# Transcriptome profiling reveals the expression and regulation of genes associated with Fusarium wilt resistance in chickpea (*Cicer arietinum* L.)

**DOI:** 10.1002/tpg2.20340

**Published:** 2023-05-21

**Authors:** Vanika Garg, Annapurna Chitikineni, Mamta Sharma, Raju Ghosh, Srinivasan Samineni, Rajeev K. Varshney, Himabindu Kudapa

**Affiliations:** ^1^ Centre for Crop and Food Innovation, WA State Agricultural Biotechnology Centre, Food Futures Institute Murdoch University Murdoch Western Australia Australia; ^2^ International Crops Research Institute for the Semi‐Arid Tropics (ICRISAT) Hyderabad India; ^3^ Crop Diversification and Genetics International Center for Biosaline Agriculture (ICBA) Dubai Uniited Arab Emirates

## Abstract

Fusarium wilt (FW) is one of the most significant biotic stresses limiting chickpea production worldwide. To dissect the molecular mechanism of FW resistance in chickpea, comparative transcriptome analyses of contrasting resistance sources of chickpea genotypes under control and *Fusarium oxysporum* f. sp. *ciceris* (*Foc*) inoculated conditions were performed. The high‐throughput transcriptome sequencing generated about 1137 million sequencing reads from 24 samples representing two resistant genotypes, two susceptible genotypes, and two near‐isogenic lines under control and stress conditions at two‐time points (7th‐ and 12th‐day post‐inoculation). The analysis identified 5182 differentially expressed genes (DEGs) between different combinations of chickpea genotypes. Functional annotation of these genes indicated their involvement in various biological processes such as defense response, cell wall biogenesis, secondary metabolism, and disease resistance. A significant number (382) of transcription factor encoding genes exhibited differential expression patterns under stress. Further, a considerable number of the identified DEGs (287) co‐localized with previously reported quantitative trait locus for FW resistance. Several resistance/susceptibility‐related genes, such as *SERINE/THREONINE PROTEIN KINASE*, *DIRIGENT*, and *MLO* exhibiting contrasting expression patterns in resistant and susceptible genotypes upon *Foc* inoculation, were identified. The results presented in the study provide valuable insights into the transcriptional dynamics associated with FW stress response in chickpea and provide candidate genes for the development of disease‐resistant chickpea cultivars.

## Core Ideas

- Transcriptome sequencing of Fusarium wilt (FW) responsive chickpea genotypes elucidates a set of 5182 differentially expressed genes (DEGs).
- Identified DEGs were involved in defense response, cell wall biogenesis, secondary metabolism, and disease resistance.
- A total of 287 DEGs co‐localized with previously reported quantitative trait loci for FW resistance.
- Near isogenic lines reduce genetic background in transcriptome studies.
- Transcriptome dataset with candidate genes is an excellent functional genomics resource for resistance breeding.

**Abbreviations**
BLAST: basic local alignment search tool
CDPKs: calcium‐dependent protein kinases
DEGs: differentially expressed genes
dpi: day post‐inoculation
FPKM: fragments per kilobase of transcript per million mapped reads
FW: Fusarium wilt
GO: gene ontology
GST: glutathione‐S‐transferase
HSPs: heat shock proteins
ICRISAT: International Crops Research Institute for the Semi‐Arid Tropics
KEGG: Kyoto Encyclopedia of Genes and Genomes
MAPK: mitogen‐activated protein kinase
NBS‐LRR: nucleotide‐binding site–leucine‐rich repeat
NCBI: National Centre for Biotechnology Information
NILs: near‐isogenic lines
PPR: pentatricopeptide repeat protein
QTL: quantitative trait locus
ROS: reactive oxygen species
SSR: simple sequence repeat
TF: transcription factor

## 1 INTRODUCTION

Chickpea (*Cicer arietinum* L.) is one of the most important grain legumes, with an annual production of 15.87 million tons (FAOSTAT, 2021). It is a self‐pollinated, diploid annual crop with a genome size of ∼740 Mb and grown predominantly in arid and semi‐arid regions of the world (Varshney et al., 2013). The importance of chickpea lies in its intrinsic potential for symbiotic nitrogen fixation and its dietary proteins, vitamins, and essential minerals. Chickpea production is essential for food security and improving the nutritional quality of diets for people living mainly in developing countries. Global chickpea production has seen a significant rise in recent years owing to the research efforts that have resulted in the development and release of improved varieties (FAOSTAT, 2021). However, meeting the growing demand calls for higher productivity gains in the chickpea crop. Further improving crop productivity will require sustainable management of devastating diseases like Fusarium wilt (FW) and Ascochyta blight (AB), which put chickpea cultivation at great risk. FW, caused by a soil‐borne fungus, *Fusarium oxysporum* f. sp. *ciceris* (*Foc*), is one of the most prevalent diseases of chickpea worldwide. FW is reported to cause yield losses varying from 10 to 100% depending on varietal susceptibility and suitable climatic conditions (Sharma et al., 2012). Since FW is a soil‐borne disease, its management through crop rotation strategies or chemical control is difficult. Therefore, using varieties resistant to FW is the most cost‐effective, promising, and environmentally sustainable strategy to manage the disease. In this direction, several quantitative trait loci (QTLs) for FW resistance have been reported to develop FW‐resistant cultivars via molecular breeding (Garg et al., 2018; Sabbavarapu et al., 2013; Varshney et al., 2014). However, genetic variability in the pathogen is high, causing varied levels of virulence and leading to the breakdown of resistance in available sources (Sharma et al., 2012). To expedite the molecular breeding process or to develop resistant varieties via gene editing approaches, it is imperative to have deeper insights into the molecular mechanism of FW resistance in chickpea.

Upon stress, plants undergo a series of physiological, morphological, molecular, and biochemical changes mediated by a network of genetic regulations. These genetic regulations mostly include modifications in gene expression at both transcriptional and post‐transcriptional levels. The identification of genes and their expression quantification have been concrete activities in molecular biology ever since the establishment of RNA's role as the fundamental mediator between the genome and proteome. The study of gene expression profiles under stress can help to pinpoint important components of resistance mechanisms. The advent of RNA‐seq has facilitated transcript identification and gene expression quantification in a robust manner. Several transcriptomic studies have successfully delineated the molecular mechanisms of drought, salinity, and AB stress in chickpea (Garg et al., 2019; Mashaki et al., 2018). Different studies using the long serial analysis of gene expression, or microarray approach were performed for only two genotypes (Ashraf et al., 2018; Upasani et al., 2017); however, high‐throughput and comprehensive analysis of genes involved in FW resistance is not yet available.

To get a deeper understanding of FW resistance in chickpea, we employed RNA‐seq to analyze the transcriptomes of roots of six contrasting chickpea genotypes for FW resistance under control and stress conditions at two different time points. We investigated these data to unravel the transcriptome dynamics associated with FW stress in chickpea and identified key differences that determine disease resistance in chickpea. The genes, transcription factors, and biological pathways showing specific and differential expression patterns between resistant and susceptible genotypes were identified. The identified genes, pathways, and molecular interactions regulating disease resistance against *F. oxysporum* in chickpea provide potential candidate targets for the development/improvement of FW‐resistant chickpea cultivars.

## 2 MATERIALS AND METHODS

### 2.1 Plant material

A total of four chickpea genotypes and two near‐isogenic lines with contrasting phenotypes for FW resistance were selected for the study. The genotype, WR 315, is a desi landrace resistant to FW (*Foc* race 1A, race 2, race 4, and race 5) (Gaur & Chaturvedi, 2004), while the genotype ICCV 05528 is a breeding line with a moderate level of disease resistance (Sharma et al., 2012). The C 214 and JG 62 are elite desi cultivars susceptible to FW (early wilter) (Pande et al., 2012). The two near‐isogenic lines (NILs)—NIL 01 (NIL 10057) and NIL 02 (NIL 10058) are obtained from introgression of *foc*1 locus conferring resistance to race 1 of FW from WR 315 into the genetic background of C 214 (Sabbavarapu et al., 2013; Varshney et al., 2014).

### 2.2 Stress treatment

For the imposition of stress, seedling raising and inoculum preparation were carried out as described earlier (Sharma et al., 2010). In brief, all the six lines selected were raised in trays filled with sterilized river sand in a greenhouse maintained at 25 ± 1°C for 10 days. *Foc* race 1 inoculum was prepared from a single conidial culture of *F. oxysporum* f. sp. *ciceris* isolated from wilt‐infected plants collected from an International Crops Research Institute for the Semi‐Arid Tropics (ICRISAT) wilt sick plot. To prepare mass inoculum, a 7‐mm disc of actively growing *F. oxysporum* f. sp. *ciceris* culture was put into a 250‐mL conical flask containing 100 mL of sterilized potato dextrose broth and incubated for 7 days in an incubator shaker (25 ± 1°C and 125 rpm). The culture was homogenized in sterilized distilled water and adjusted to 6 × 10^5^ conidia mL^–1^ using a haemocytometer for use as an inoculum (as per Sharma et al., 2010). Ten‐day‐old seedlings of each test line grown in sterilized river sand were uprooted, cleaned with tap water, and inoculated by dipping in inoculum suspension for 1–2 min to enable conidia to adhere to the roots. Inoculated seedlings were transplanted in pre‐irrigated sterile vertisol and sand (3:1) in pots and incubated in a greenhouse at 25 ± 3°C.

### 2.3 Sample collection and RNA isolation

The root tissues from both uninoculated/control and inoculated/stressed plants were harvested at 7th day post‐inoculation (dpi) and 12th dpi in sets of three biological replicates. Total RNA was isolated using the “NucleoSpin RNA Plant” kit (Macherey‐Nagel, Düren). Further, the quality of isolated RNA was assessed using Agilent 2100 Bioanalyzer (Agilent Technologies) and Nanodrop 8000 Spectrophotometer (Thermo Fisher Scientific).

### 2.4 Transcriptome sequencing and reference‐guided assembly

High‐quality total RNA (RIN ≥ 8) was subjected to library construction using TruSeq RNA Sample Prep Kit v2 (Illumina Inc.) as described previously (Garg et al., 2019). The libraries were sequenced on Illumina HiSeq 2500 platform using a 150 bp‐paired end strategy. In total, 24 samples representing six different genotypes (C 214, JG 62, ICCV 05528, WR 315, NIL 01, and NIL 02) under control and *F. oxysporum* inoculated conditions at two‐time points (7th and 12th dpi) were sequenced. The raw reads generated from transcriptome sequencing were processed to remove primer/adaptor contamination and low‐quality reads (>20% of the bases with a Phred quality score < 10) using Trimmomatic v0.35 with parameters “ILLUMINACLIP:adaptors.fa:2:30:10 SLIDINGWINDOW:10:20 MINLEN:50” (Bolger et al., 2014). The paired‐end clean reads were mapped to the chickpea reference genome Cicar.CDCFrontier_v2.0 (Garg et al., 2022) using HISAT2 v2.1.0 with default parameters (Kim et al., 2019). The mapped reads from each sample were assembled using Cufflinks v2.2.1 with default parameters to generate reference‐guided assemblies (Trapnell et al., 2012). These assemblies were merged to generate a consensus assembly using Cuffmerge with default parameters, and the assembly thus obtained was used for downstream analysis.

**Core Ideas**

- Transcriptome sequencing of Fusarium wilt (FW) responsive chickpea genotypes elucidates a set of 5182 differentially expressed genes (DEGs).
- Identified DEGs were involved in defense response, cell wall biogenesis, secondary metabolism, and disease resistance.
- A total of 287 DEGs co‐localized with previously reported quantitative trait loci for FW resistance.
- Near isogenic lines reduce genetic background in transcriptome studies.
- Transcriptome dataset with candidate genes is an excellent functional genomics resource for FW resistance breeding.

### 2.5 Expression profiling and identification of DEGs

The transcript abundance was estimated based on fragments per kilobase of transcript per million mapped reads (FPKM) using Cufflinks. Transcripts with FPKM ≥ 1 in any of the samples and quantification status as “OK” were considered to be expressed and were used for downstream analysis. The differential gene expression between different samples was estimated using Cuffdiff (Trapnell et al., 2013). Differentially expressed genes (DEGs) were identified between different samples under control (noninoculated), and *F. oxysporum* inoculated conditions at different time points. The different pairwise combinations included stress versus control samples of each genotype at 7th and 12th dpi; resistant versus susceptible genotypes under control conditions at 7th and 12th dpi; and resistant versus susceptible genotypes under FW stress conditions at both 7th and 12th dpi. The DEGs with log_2_ fold change ≥ 2 (upregulated) and/or ≤ −2 (downregulated) and an FPKM ≥ 2 for either of the samples in each pair‐wise comparison were considered to be significantly differentially expressed. The log_2_ transformed FPKM values of the DEGs were further subjected to *k*‐means clustering using pheatmap package in R/Bioconductor.

### 2.6 Annotation and GO enrichment of DEGs

To predict putative function, the identified DEGs were subjected to similarity search using BLASTX (*E*‐value cut‐off of ≤ 1E–5) against the National Center for Biotechnology Information nonredundant Viridiplantae protein database and were further assigned gene ontology (GO) terms using Blast2GO v4.1.9 (Conesa et al., 2005). To pinpoint the overrepresented functional categories in DEGs across different sample comparisons, an R package GOseq (Young et al., 2010) was used. GO terms displaying a corrected *p*‐value of ≤ 0.05 for a given set of genes were considered to be significantly enriched. The enriched GO terms were summarized and visualized using REVIGO (Supek et al., 2011).

### 2.7 Pathway and TF analysis

The identified DEGs were studied for their involvement in different pathways using the Kyoto Encyclopedia of Genes and Genomes (KEGG) Automatic Annotation Server (Moriya et al., 2007). The pathway enrichment analysis was performed based on a hypergeometric model (Boyle et al., 2004) with a significance threshold of *p*‐value of 0.05. The enriched pathways were plotted as a scatter plot using data visualization R package ggplot2 (Wickham, 2016). Further, the transcription factor encoding genes were predicted using a similarity search against the PlantTFDB 5.0 database (Tian et al., 2020) with an *E*‐value cut‐off of 1E‐10. For co‐localization studies, information about the FW resistance QTLs was obtained from previous studies (Garg et al., 2018; Patil et al., 2014; Sabbavarapu et al., 2013). The sequence similarity search using BLASTN was performed to obtain the physical locations of the markers associated with FW resistance QTLs in the chickpea reference genome. Only hits with 100% query and subject coverage were retained.

## 3 RESULTS

### 3.1 Transcriptome sequencing and reference‐guided assembly

In the present study, a total of 24 samples representing four chickpea genotypes (WR 315‐resistant, ICCV 05528‐moderately resistant, C 214‐susceptible, and JG 62‐susceptible) and two near‐isogenic lines (NIL 10057 and NIL 10058, designated as NIL 01 and NIL 02, respectively) under control and stress (*F. oxysporum* inoculated) conditions at two (7th dpi and 12th dpi) time points were subjected to transcriptome sequencing. The two‐time points identified for this study were selected based on the earlier reports in legumes that the initial symptoms begin at 7th dpi and express to the maximum at 12th dpi (Biswas et al., 2020). The NIL 01 and NIL 02 used in the present study were obtained by introgressing FW‐resistant QTLs from WR 315 into C 214 (Varshney et al., 2014). In the study, the samples are named genotype‐condition‐dpi for ease of understanding. For instance, C 214‐C‐7d represents genotype C 214 under control conditions at 7th dpi. A total of 1137 million reads were generated from the paired‐end sequencing of these 24 samples. After trimming and filtering low‐quality reads, a set of 1110 million high‐quality reads were retained and mapped on the recently available chickpea genome (Garg et al., 2022). Overall, about 95% of the high‐quality reads were mapped on the reference genome (Table 1). The sequencing data and alignment statistics reflect very high‐quality transcriptome sequencing. On average, 93.27% of the reads were mapped to the exonic region, 0.83% to the intronic region, and 5.90% to intergenic regions of the chickpea genome (Table S1). The mapped reads from all 24 samples were used to generate reference‐guided assembly using the Cufflinks‐Cuffmerge pipeline. This assembly generated 76,382 transcripts representing 27,736 gene loci, including 25,695 known and 2041 novel gene loci.

**TABLE 1:** Statistics of transcriptome sequencing data generated for 24 samples obtained from six different chickpea genotypes.

Sample	Raw reads	Filtered reads	Filtered reads (%)	Mapped reads	Mapped reads (%)
C 214‐C‐7d	42,855,890	42,018,711	98.05	41,053,642	97.70
C 214‐S‐7d	68,300,372	67,301,196	98.54	63,588,867	94.48
C 214‐C‐12d	45,760,416	44,851,746	98.01	43,952,707	98.00
C 214‐S‐12d	45,318,494	44,171,702	97.47	41,826,372	94.69
ICCV 05528‐C‐7d	41,176,344	40,161,473	97.54	38,679,310	96.31
ICCV 05528‐S‐7d	76,444,046	75,200,992	98.37	69,633,871	92.60
ICCV 05528‐C‐12d	44,135,388	43,245,710	97.98	42,451,202	98.16
ICCV 05528‐S‐12d	45,424,154	44,108,667	97.10	41,230,585	93.48
JG 62‐C‐7d	43,164,592	42,061,605	97.44	41,105,924	97.73
JG 62‐S‐7d	39,953,776	38,897,082	97.36	35,536,177	91.36
JG 62‐C‐12d	42,873,788	41,702,173	97.27	40,712,386	97.63
JG 62‐S‐12d	48,771,794	47,281,335	96.94	44,930,450	95.03
NIL 01‐C‐7d	46,350,560	45,178,442	97.47	43,648,003	96.61
NIL 01‐S‐7d	53,447,742	52,473,278	98.18	49,810,856	94.93
NIL 01‐C‐12d	39,440,102	38,178,489	96.80	37,133,220	97.26
NIL 01‐S‐12d	44,696,102	43,461,280	97.24	40,634,452	93.50
NIL 02‐C‐7d	45,465,094	44,397,549	97.65	42,286,280	95.24
NIL 02‐S‐7d	64,248,978	63,117,485	98.24	56,887,070	90.13
NIL 02‐C‐12d	43,588,388	42,633,020	97.81	41,748,412	97.93
NIL 02‐S‐12d	40,373,906	39,037,532	96.69	36,847,374	94.39
WR 315‐C‐7d	40,835,890	39,940,681	97.81	39,043,810	97.75
WR 315‐S‐7d	41,035,634	39,936,236	97.32	36,482,837	91.35
WR 315‐C‐12d	48,526,488	47,125,222	97.11	45,662,475	96.90
WR 315‐S‐12d	44,507,508	43,323,914	97.34	40,594,183	93.70

### 3.2 Global gene expression analysis

The normalized expression level (FPKM) of each gene was estimated in all the samples. The genes with very low expression values (see Section 2) in all 24 samples were filtered out. After filtering, a set of 21,609 genes exhibiting expression in at least one of the samples was obtained. The number of expressed genes varied from a minimum of 18,041 genes in WR 315‐S‐12d to a maximum of 18,872 genes in ICCV 05528‐C‐12d (Table 2). Further, based on the expression level, the genes were classified into highly expressed (FPKM ≥10) and low/moderately expressed genes (FPKM ≥1 and <10). The number of low/moderately expressed genes was less than highly expressed genes across all the samples in the study. The number of low/moderately expressed genes ranged from 7563 in JG 62‐S‐12d to 8390 in ICCV 05528‐C‐12d, whereas highly expressed genes ranged from 10,257 in ICCV 05528‐S‐12d to 10,671 in NIL 01‐C‐12d (Table 2).

**TABLE 2:** Distribution of expressed genes identified from different chickpea genotypes.

Sample ID	No. of expressed genes (FPKM ≥ 1)	No. of low/moderately expressed genes (FPKM ≥ 1 AND < 10)	No. of highly expressed genes (FPKM ≥ 10)
C 214‐C‐7d	18,651	8107	10,544
C 214‐S‐7d	18,411	7970	10,441
C 214‐C‐12d	18,753	8260	10,493
C 214‐S‐12d	18,237	7902	10,335
ICCV 05528‐C‐7d	18,531	8007	10,524
ICCV 05528‐S‐7d	18,450	8106	10,344
ICCV 05528‐C‐12d	18,872	8390	10,482
ICCV 05528‐S‐12d	18,165	7908	10,257
JG 62‐C‐7d	18,847	8293	10,554
JG 62‐S‐7d	18,141	7650	10,491
JG 62‐C‐12d	18,251	7810	10,441
JG 62‐S‐12d	18,141	7563	10,578
NIL 01‐C‐7d	18,602	8107	10,495
NIL 01‐S‐7d	18,420	8142	10,278
NIL 01‐C‐12d	18,365	7694	10,671
NIL 01‐S‐12d	18,381	8023	10,358
NIL 02‐C‐7d	18,439	7944	10,495
NIL 02‐S‐7d	18,501	8129	10,372
NIL 02‐C‐12d	18,624	8090	10,534
NIL 02‐S‐12d	18,572	8005	10,567
WR 315‐C‐7d	18,847	8279	10,568
WR 315‐S‐7d	18,111	7671	10,440
WR 315‐C‐12d	18,674	8093	10,581
WR 315‐S‐12d	18,041	7637	10,404

Abbreviation: FPKM, fragments per kilobase of transcript per million mapped reads.

### 3.3 Identification of differentially expressed genes in response to FW stress

**FIGURE 1:**
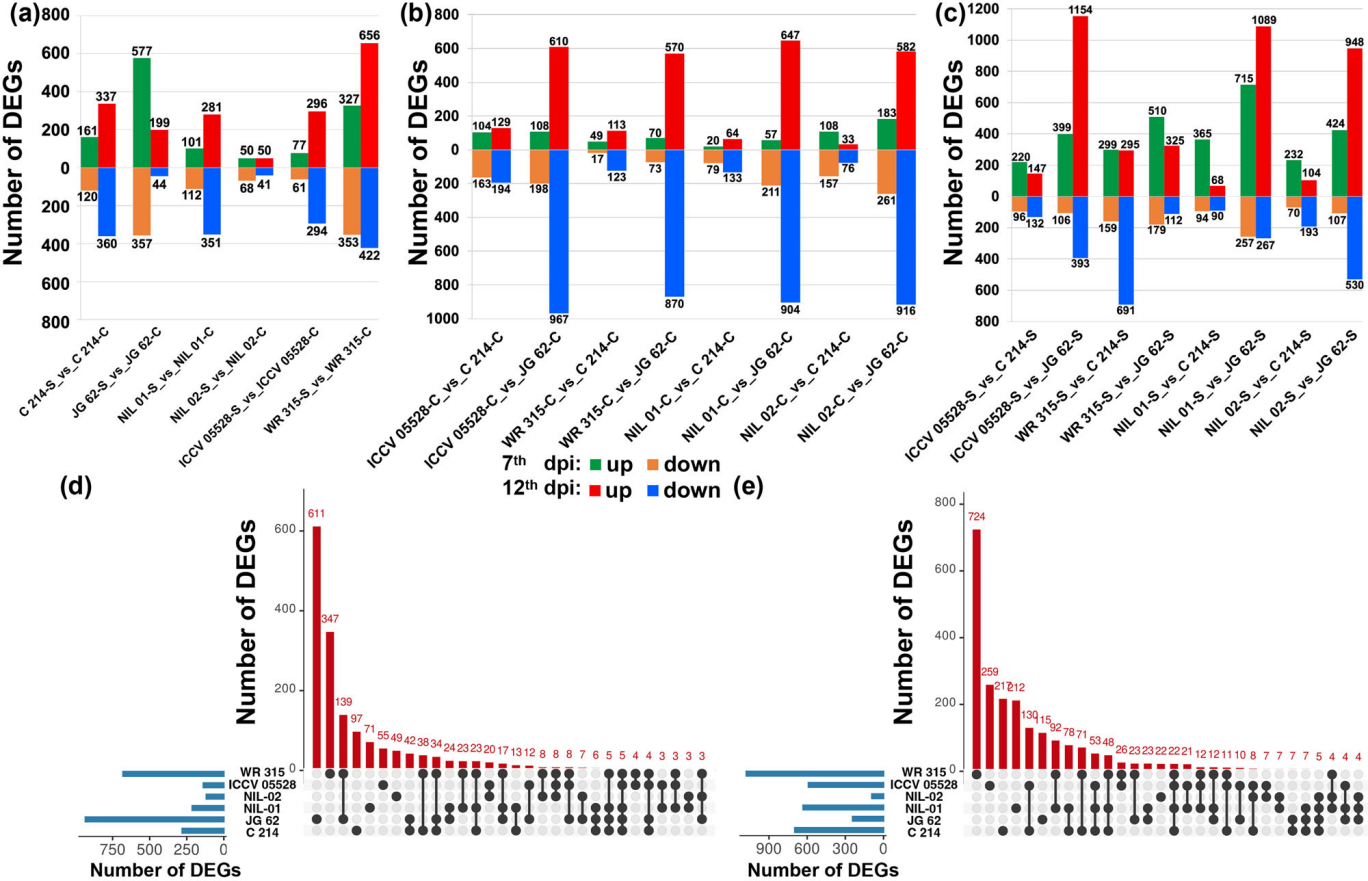
Statistics of differentially expressed genes (DEGs) identified between different combinations of chickpea genotypes in response to *Fusarium oxysporum* stress. (a) The number of DEGs in each chickpea genotype (C 214, JG 62, WR 315, ICCV 05528, NIL 01, and NIL 02) at 7th and 12th day post‐inoculation (dpi) under control and *F. oxysporum* inoculated conditions are presented in the bar graph. The number of up‐ and downregulated genes are shown in the form of bars above and below the *x*‐axis, respectively; (b) number of DEGs between different genotypes at both 7th and 12th dpi under control conditions; (c) number of DEGs between different genotypes at both 7th and 12th dpi under stress conditions. Upset plots depicting overlapping and specific response of DEGs within different chickpea genotypes under control versus stress conditions at (d) 7th dpi and (e) 12th dpi. The number of genes showing specific and overlapping response is mentioned on top of each bar. The horizontal bars represent the number of DEGs per genotype. The black circles below the vertical bars denote the genotype(s) and vertical bars represent the number of DEGs overlapping or unique to the respective genotypes.

The fold change of each gene was calculated across different pairwise combinations using Cuffdiff. A gene was considered differentially expressed if it exhibited a log_2_ fold change of ≥ 2 (upregulated) or ≤ −2 (downregulated). Using this criterion, a total of 5182 genes were differentially expressed in at least one sample combination (Table S2). The differential expression patterns between control and stress samples of each genotype were studied at both stages of infection (7th and 12th dpi). At 7th dpi, the maximum DEGs were identified in the JG 62 genotype (577 upregulated; 357 downregulated), followed by WR 315 (327 upregulated; 353 downregulated) (Figure 1a–c). At 12th dpi, the maximum transcriptional changes were observed in WR 315 genotype with differential expression of 1078 genes (656 upregulated; 422 downregulated). The minimum number of DEGs was identified between C 214‐C‐7d and WR 315‐C‐7d (63), and the maximum was identified between ICCV 05528‐S‐12d and JG 62‐S‐12d (1452). Under stress, the pattern of upregulation was more profound at the 12th dpi than the 7th dpi across all genotypes (Figure 1c).

Further, an overlap of DEGs between different genotypes under control and stress conditions at both stages of infection was studied. A significant number of DEGs were unique to each combination. In total, 1230 genes showed genotype‐specific expression patterns under stress at 7th dpi. At 12th dpi, 1549 DEGs were genotype‐specific. At 7th dpi, the maximum number of genotype‐specific DEGs were found in JG 62 (611) and the minimum in NIL 02 (49) (Figure 1d). Similarly, at 12th dpi, WR 315 (724) and NIL 02 (22) had the maximum and minimum genotype‐specific DEGs, respectively (Figure 1e). A total of 1915 genes exhibited differential expression patterns at both 7th and 12th dpi upon infection in any genotypes. These findings indicate an extensive genotype‐ and stress stage‐specific response of chickpea to *Foc* infection.

### 3.4 Genome‐wide distribution of DEGs and localization with FW QTLs

**FIGURE 2:**
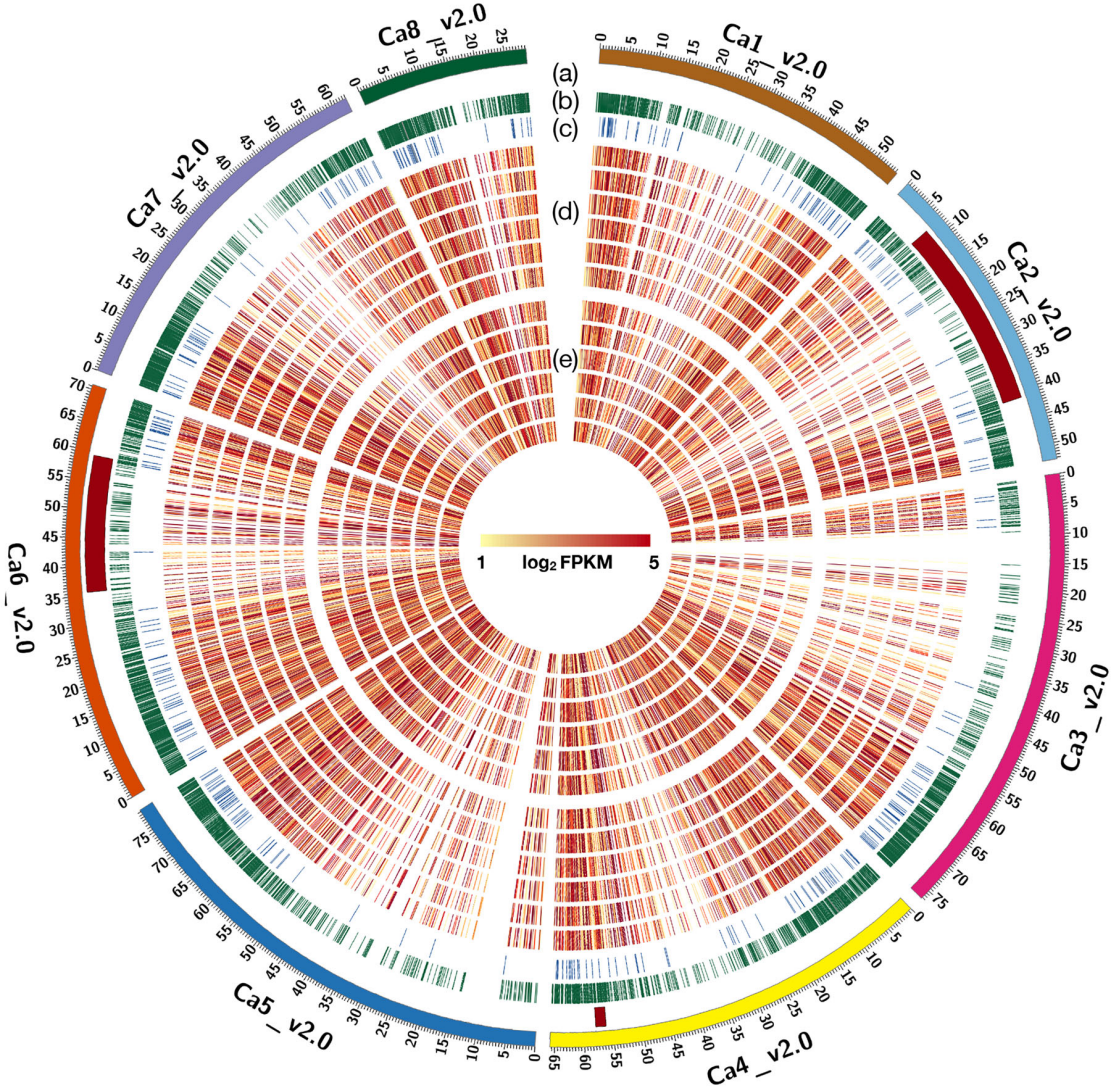
A circular plot representing genome‐wide distribution of Fusarium wilt (FW) resistance quantitative trait loci (QTLs), differentially expressed genes (DEGs), transcription factors (TFs), and expression of genes at 7th and 12th day post‐inoculation (dpi). Six different tracks (out to in) of the circular plot shows the following: (a) FW resistance QTLs; (b) DEGs; (c) differentially expressed TFs; (d) expression of genes (log_2_ fragments per kilobase of transcript per million mapped reads, FPKM) in different genotypes at 7th dpi (moving from out to in; C 214, JG 62, NIL 01, NIL 02, ICCV 05528, and WR 315); (e) expression of genes (log_2_ FPKM) in different genotypes at 12th dpi (moving from out to in; C 214, JG 62, NIL 01, NIL 02, ICCV 05528, and WR 315).

The identified DEGs were studied for their distribution in the chickpea genome. Out of 5182 DEGs, 5108 were mapped to the pseudomolecules of the chickpea genome and the remaining on scaffolds (Figure 2). The highest number of DEGs were present on pseudomolecule Ca6_v2.0 (803), followed by pseudomolecule Ca4_v2.0 (741), and the lowest was present on pseudomolecule Ca8_v2.0 (431). Several QTLs have been reported for FW resistance to date (Garg et al., 2018; Patil et al., 2014; Sabbavarapu et al., 2013). The identified DEGs were further co‐localized with previously reported FW resistance QTLs. By deploying the physical position of the simple sequence repeats (SSRs) markers on the chickpea pseudomolecules, these QTLs were mapped on pseudomolecules Ca2_v2.0, Ca4_v2.0, and Ca6_v2.0 of the chickpea reference genome (Garg et al., 2022). Further, a total of 287 out of the 5182 DEGs, were co‐localized with seven FW resistance QTLs. Of these 287 co‐localized DEGs, 39 were novel. The maximum DEGs were present in two QTLs, namely, *FW‐Q‐APR‐2‐1* (87, Garg et al., 2018) and Wilt 2 (76, Patil et al., 2014) located on pseudomolecule Ca2_v2.0 (Figure 2).

**FIGURE 3:**
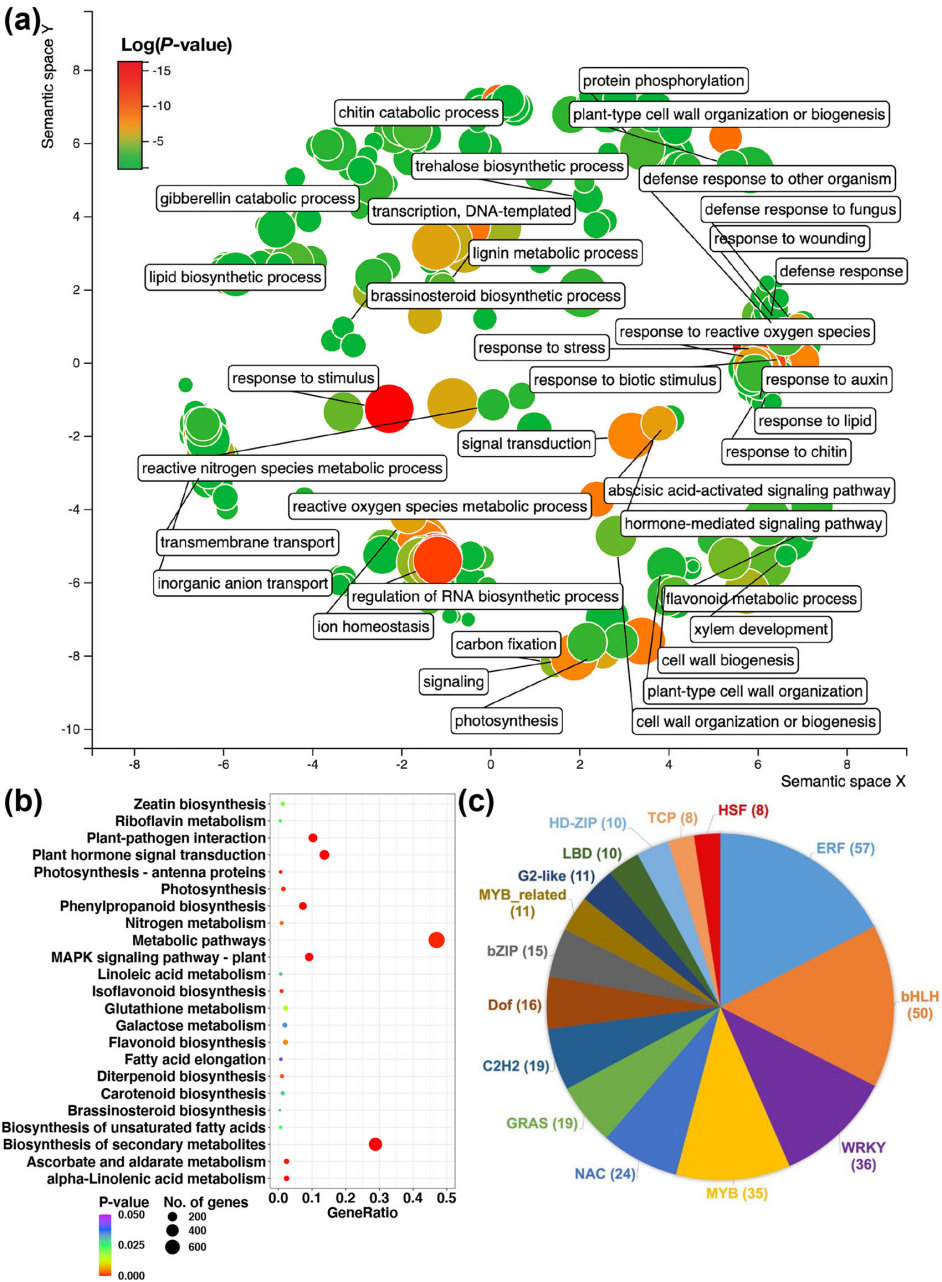
Annotation and pathway analysis of the differentially expressed genes (DEGs). (a) Gene ontology (GO) enrichment analysis of DEGs identified between different chickpea genotypes under control and stress conditions. GO terms in the biological process category are summarized and visualized using REVIGO. GO terms are represented by circles where the size of the circle is proportional to the frequency of the GO term, and the color indicates the log (*p*‐value). (b) Scatter plot of the significantly enriched Kyoto Encyclopedia of Genes and Genomes (KEGG) pathways (*p* < 0.05) during Fusarium wilt (FW) stress conditions. Gene ratio is the ratio of the number of DEGs in the pathway and the total number of DEGs with pathway annotation. (c) Pie chart showing the distribution of differentially expressed transcription factors in response to FW stress.

Further, functional annotations were assigned to the identified DEGs using BLASTX. From the total, 4767 DEGs were annotated, and 4213 of these DEGs were assigned GO terms. A total of 13,823 GO terms were retrieved for these DEGs, and these GO terms were evenly assigned to biological process (4261), molecular function (5813), and cellular component (3749) categories. GO enrichment analysis of the identified DEGs suggested the enrichment of GO terms such as defense response (GO:0006952), response to reactive oxygen species (GO:0000302), ion homeostasis (GO:0050801), signal transduction (GO:0007165), defense response to fungus (GO:0050832), cell wall biogenesis (GO:0042546), response to chitin (GO:0010200), brassinosteroid biosynthetic process (GO:0016132), abscisic acid‐activated signaling pathway (GO:0009738), and response to auxin (GO:0009733) in the biological process category (Figure 3a; Table S3). Among the enriched GO terms related to biological processes, response to chitin, defense response, lipid transport, abscisic acid‐activated signaling pathway, MAPK cascade (GO:0000165), and flavonoid biosynthesis (GO:0009813) were mainly associated with genes showing increased expression upon stress, whereas genes showing downregulation were related to circadian rhythm (GO:0007623) and response to ethylene (GO:0009723). Among the cellular component category, extracellular region (GO:0005576), plant‐type cell wall (GO:0009505), apoplast (GO:0048046), photosystem (GO:0009521), and anchored component of plasma membrane (GO:0046658) were the most overrepresented GO terms (Table S4). In the molecular function category, oxidoreductase activity (GO:0016491), transcription regulator activity (GO:0140110), protein serine/threonine kinase activity (GO:0004674), peroxidase activity (GO:0004601), hydrolase activity (GO:0016798), and monooxygenase activity (GO:0004497) were the most significantly enriched GO terms (Table S5).

Further, pathway analysis of the DEGs was performed to identify pathways enriched in response to FW stress in chickpea. We identified a total of 130 diverse pathways using the KEGG database. The enrichment analysis (*p*‐value < 0.05) indicated that genes involved in the biosynthesis of secondary metabolites (ko01110), MAPK signaling pathway (ko04016), plant‐pathogen interaction (ko04626), plant hormone signal transduction (ko04075), flavonoid biosynthesis (ko00941), and fatty acid elongation (ko00062) were significantly differentially expressed (Figure 3b).

Transcription factors (TF), being the upstream regulatory proteins, play significant roles under both abiotic and biotic stresses in plants (Bhoghireddy et al., 2020; Nuruzzaman et al., 2013). The reference‐guided transcriptome assembly identified members of 58 TF families represented by 1564 genes using the PlantTFDB database (Tian et al., 2020). Among these, 382 TF‐related genes from 39 families were found to show differential expression patterns under FW stress (Table S6). The maximum members of the ERF family (56) were differentially expressed, followed by members of bHLH (43), WRKY (35), MYB (32), and C2H2 (23) TF families. In addition, NAC (19), GRAS (17), Dof (17), and bZIP (16) TF families also exhibited differential expression patterns under stress conditions (Figure 3c). Taken together, these analyses provided an important resource for identifying specific processes, functions, and pathways associated with FW resistance in chickpea.

### 3.5 Expression trends across resistant and susceptible chickpea genotypes under FW stress

**FIGURE 4:**
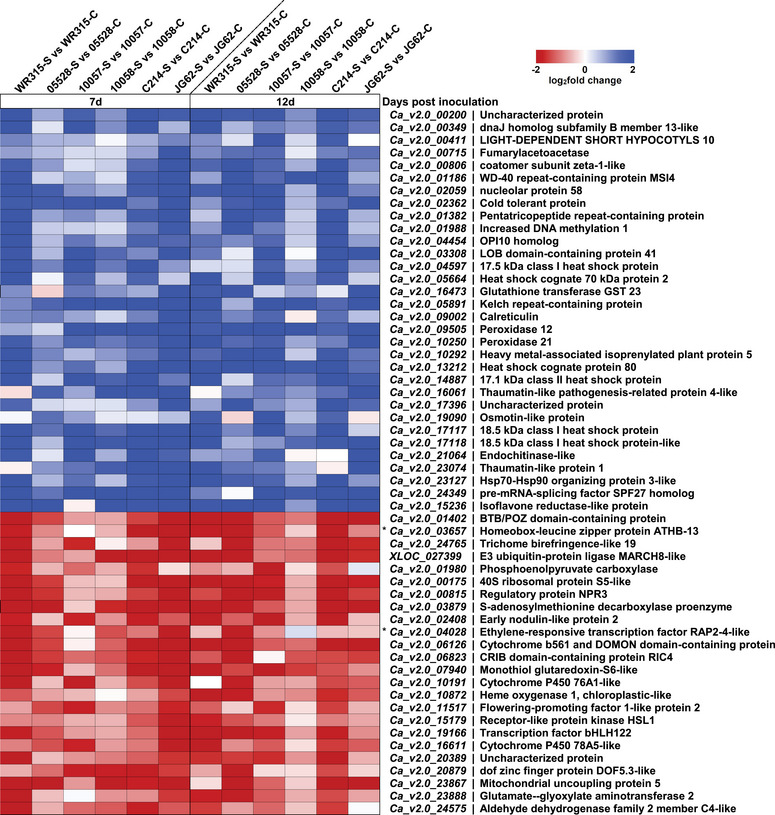
Response common to both resistant and susceptible genotypes under stress conditions. The heatmap shows the expression profiles of differentially expressed genes (DEGs) under Fusarium wilt (FW) stress conditions at 7th and 12th dpi in each chickpea genotype (C 214, JG 62, NIL 01, NIL 02, ICCV 05528, and WR 315). The genes showing similar expression trends in all genotypes upon *Fusarium oxysporum* infection are shown. The color scale at the top shows log_2_ fold change. In the heat map, blue signifies upregulation/induced expression and red signifies downregulation/repressed expression upon stress. The asterisk shows that the gene is present in one of the previously reported FW quantitative trait loci (QTLs).

To gain deeper insights into the molecular mechanisms of resistance induced by *Foc*, the identified DEGs were investigated for their expression profiles in different genotypes under stress conditions. A group of genes mainly associated with defense response, phosphorylation, redox homeostasis, and signal transduction exhibited a trend of upregulation in all the genotypes irrespective of resistance/susceptibility at both 7th and 12th dpi. Genes encoding for pathogenesis‐related proteins (*Ca_v2.0_23074* and *Ca_v2.0_19090*), endochitinase (*Ca_v2.0_21064*), glutathione‐S‐transferase (*Ca_v2.0_16473*), serine‐threonine kinase (*Ca_v2.0_17396*), peroxidases (*Ca_v2.0_09505* and *Ca_v2.0_10250*), and several classes of heat shock proteins (HSPs; *Ca_v2.0_05664*, *Ca_v2.0_13212*, *Ca_v2.0_14887*, and *Ca_v2.0_17117*) were part of this group (Figure 4). Similarly, another group of genes related to growth (*Ca_v2.0_06823*), photosynthesis (*Ca_v2.0_10872* and *Ca_v2.0_23888*), leaf development (*Ca_v2.0_01980*), chlorophyll biosynthesis (*Ca_v2.0_03879*), and flower development (*Ca_v2.0_11517*) showed a general trend of downregulation in all genotypes at both 7th and 12th dpi (Figure 4). Upon FW infection, a highly genotype‐specific response was also observed. Several stress‐responsive genes such as NBS‐LRR (*Ca_v2.0_06556*), chitinase (*Ca_v2.0_09545*), and glutathione‐S‐transferase (*Ca_v2.0_06398*) were highly upregulated specifically in WR 315 upon stress. In contrast, the expression levels of these genes were considerably lower or downregulated in the moderately resistant and susceptible genotypes.

**FIGURE 5:**
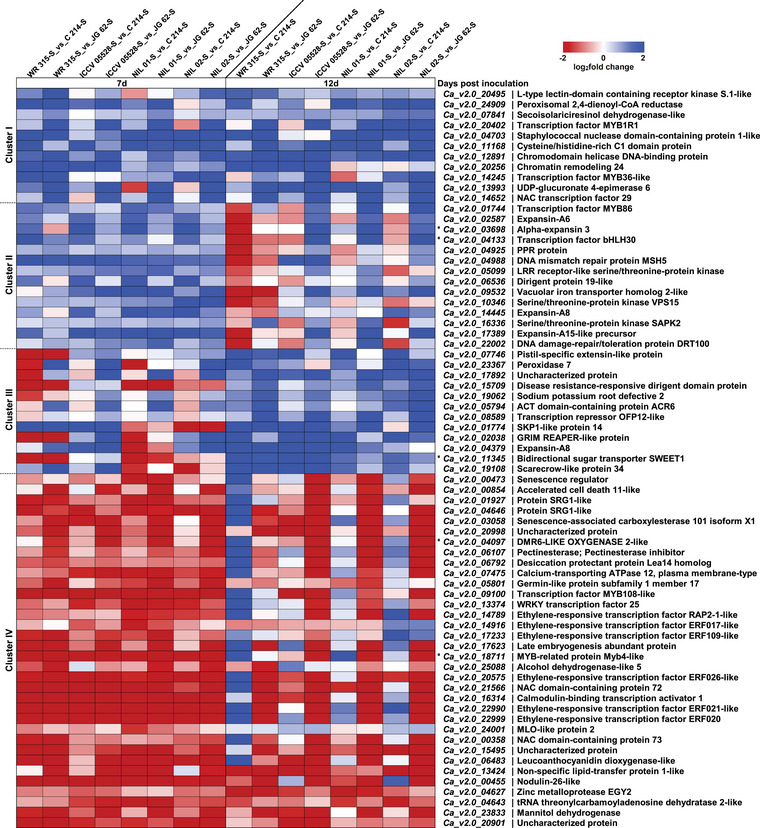
Response specific to resistant and susceptible chickpea genotypes under stress conditions. The genes with similar expression patterns have been grouped together into four clusters and selected representative genes from each cluster are shown in the heatmap. Clusters I–IV represent differentially expressed genes (DEGs) identified between resistant (including WR 315, ICCV 05528, NIL 01, and NIL 02) and susceptible genotypes (C 214 and JG 62) upon *F*oc infection at 7th and 12th dpi. The color scale at the top shows log_2_ fold change. In the heat map, blue signifies upregulation/induced expression and red signifies downregulation/repressed expression in resistant genotypes. The asterisk shows that the gene is present in one of the previously reported Fusarium wilt (FW) quantitative trait loci (QTLs).

Further, we identified key genes that might be responsible for different phenotypes of chickpea genotypes to FW stress. We investigated those genes that showed similar expression among resistant (WR 315 and ICCV 05528) and near‐isogenic lines (NIL 01 and NIL 02) but contrasting expression patterns in susceptible genotypes (C 214 and JG 62). Upon FW infection, genes (Cluster I) involved in oxidation‐reduction (*Ca_v2.0_07841*), fatty acid biosynthesis (*Ca_v2.0_24909*), and defense response (*Ca_v2.0_20495* and *Ca_v2.0_20402*) exhibited induced expression at both 7th and 12th dpi in resistant genotypes (Figure 5). Cluster II included genes showing significant up‐regulation in resistant genotypes during early infection (7th dpi). It contained genes involved in pathogen recognition such as LRR receptor‐like serine/threonine‐protein kinase (*Ca_v2.0_05099*, *Ca_v2.0_16336*, and *Ca_v2.0_10346*), ion homeostasis (*Ca_v2.0_09532*), cell wall loosening such as expansins (*Ca_v2.0_17389*, *Ca_v2.0_02587*, *Ca_v2.0_03698*, and *Ca_v2.0_14445*), DNA repair (*Ca_v2.0_04988* and *Ca_v2.0_22002*), stress‐responsive genes such as pentatricopeptide repeat proteins (PPR) (*Ca_v2.0_04925*), and Dirigent (*Ca_v2.0_06536*). Several TFs like MYB86 (*Ca_v2.0_01744*) and bHLH030 (*Ca_v2.0_04133*) were also part of this cluster. Cluster III consisted of genes showing a general trend of upregulation in all resistant genotypes during the later stage of infection (12th dpi). Several genes such as expansin‐A8 (*Ca_v2.0_04379*), SKP1‐like protein 14 (*Ca_v2.0_01774*), scarecrow‐like protein 34 (*Ca_v2.0_19108*), and GRIM REAPER‐like protein (*Ca_v2.0_02038*) were part of this cluster. Further, genes exhibiting induced expression in susceptible genotypes at either 7th or 12th dpi were grouped in Cluster IV. Genes encoding for senescence‐associated protein (*Ca_v2.0_00473* and *Ca_v2.0_03058*), accelerated cell death 11 (*Ca_v2.0_00854*), desiccation‐related protein (*Ca_v2.0_06792*), leucoanthocyanidin dioxygenase‐like protein (*Ca_v2.0_06483*), late embryogenesis abundant protein (*Ca_v2.0_17623*), pectinesterase (*Ca_v2.0_06107*), calcium‐transporting ATPase (*Ca_v2.0_07475*), downy mildew resistance 6 (DMR6; *Ca_v2.0_04097*) Mildew Locus O (MLO) like protein 2 (*Ca_v2.0_24001*), and SRG1 (*Ca_v2.0_04646* and *Ca_v2.0_01927*) showed upregulation in susceptible genotypes during early stages of infection. TFs encoding for NAC (*Ca_v2.0_00358* and *Ca_v2.0_21566*), MYB (*Ca_v2.0_18711* and *Ca_v2.0_09100*), ERF (*Ca_v2.0_14789*, *Ca_v2.0_20575*, *Ca_v2.0_14916*, *Ca_v2.0_17233*, *Ca_v2.0_22990*, and *Ca_v2.0_22999*), and WRKY25 (*Ca_v2.0_13374*) were upregulated in susceptible genotypes upon *Foc* infection (Figure 5).

## 4 DISCUSSION

Understanding the molecular basis of variability in stress response is crucial for all research designed to develop new chickpea cultivars with FW resistance. In this direction, the present study reported a comprehensive transcriptome analysis of 24 samples representing six well‐characterized chickpea genotypes (C 214, JG 62, ICCV 05528, WR 315, NIL 01, and NIL 02) under control and stress conditions at two stress stages. The data generated from these samples were used to construct a reference‐guided assembly that assembled 27,736 genes and comparison of these genes with the gene models of the chickpea reference genome led to the identification of 2041 novel genes from unannotated loci, thus demonstrating the potential of RNA‐seq in the identification of novel genes. Similar results were also reported from other transcriptome studies in chickpea (Garg et al., 2019, 2023; Kudapa et al., 2018).

An extensive transcriptional reprogramming was observed in chickpea plants under FW stress at 7th and 12th dpi. A total of 5182 genes showed differential expression patterns between contrasting stress‐responsive genotypes under control and stress conditions at different stages of infection. A significant fraction of identified DEGs were present in the QTL regions associated with FW resistance. The functional annotation, GO enrichment, and pathway analysis of these differentially expressed genes throw light on the FW resistance mechanism of chickpea. Several genes involved in the recognition of pathogens, ion homeostasis, cell wall modifications, formation of reactive oxygen species (ROS), signal transduction, and defense response were significantly enriched during FW infection. Pathogen invasion is known to affect the structure and integrity of the plant cell wall. In this study, several genes associated with “cell wall biogenesis,” “cell wall organization,” and “cell wall modification” were differentially expressed, suggesting the role of structural defense genes in FW response in chickpea similar to the observations reported from other legumes (Chang et al., 2021; Sharma et al., 2016). Several pathways related to specific physiological, metabolic, and biochemical changes were also upregulated in the present study. Transcriptome studies conducted in various plant species have reported the enrichment of similar GO terms and pathways in response to fungal stress (Garg et al., 2019; Nayak et al., 2017). Defense response involves several TFs which regulate the target gene expression by specifically binding to cis‐acting elements in the promoter regions (Park et al., 2001; Wang et al., 2009). In the present study, different classes of TFs, such as bHLH, WRKY, ERF, and bZIP exhibited differential expression patterns upon FW stress similar to those observed in previous studies (He et al., 2016; Wang et al., 2009).

When chickpea plants were challenged with *Foc*, several genes related to defense response, phosphorylation, redox homeostasis, and signal transduction exhibited significantly increased expression levels in all the genotypes. In contrast, genes involved in basic biological processes such as plant growth and photosynthesis were downregulated in all the genotypes. These genes showing similar expression in all the genotypes upon stress might be involved in the basal defense of chickpea against *F. oxysporum* infection. Under both biotic and abiotic stresses, the plant cells accumulate ROS as a part of the basal defense response. Several antioxidant enzymes such as flavonoids and peroxidases can quench the ROS generated due to infection (Gupta et al., 2019). The present study also demonstrated increased expression of peroxidases and HSPs in all genotypes upon FW infection. Genes encoding for peroxidases were also found to positively regulate defense responses against FW stress in a previous study (Ashraf et al., 2018). Various studies have reported that HSPs play an integral role in developing resistance against biotic stresses by interacting with other defense‐related proteins. The increased expression of HSP70 in this study is in concurrence with a recent report on powdery mildew (caused by *Golovinomyces orontii*) infection in sunflower (Kallamadi et al., 2018).

Several studies have reported an increase in intracellular Ca^2+^ levels in plant cells in response to both biotic and abiotic stress (Li et al., 2008; Vadassery & Oelmüller, 2009). The increased Ca^2+^levels induce expression of defense genes via the activation of ion fluxes at the plasma membrane, an oxidative burst, and MAPK activation. In the present study, several genes encoding for calcium‐dependent protein kinases (CDPKs), calcium transporting ATPase, and calmodulin‐like proteins, the major components of calcium signaling, were differentially expressed in resistant genotypes during the early stages of infection, suggesting the role of calcium signaling in FW response in chickpea. Similar observations were also reported from a previous study on FW in chickpea (Upasani et al., 2017). The increased expression of different stress‐responsive genes like serine‐threonine kinases, dirigent, and PPR protein was also seen in all resistant genotypes at different time points. Dirigent proteins are known to be implicated in the regulation of lignan biosynthesis and are important for secondary metabolism and pathogen resistance (Li et al., 2017). Their increased expression in resistant genotypes upon stress might be indicative of their involvement in FW resistance. In contrast, several genes encoding for senescence‐associated, accelerated cell death, MLO, DMR6, WRKY25, and RAP2.1 were upregulated in susceptible genotypes upon infection. Genes such as *MLO*, *DMR6*, and *WRKY25* are classified as susceptibility (S) genes and are known to mediate plant‐pathogen compatibility and facilitate pathogen infection (Henningsen et al., 2021). Loss‐of‐function mutations of *MLO* genes are reported to reduce susceptibility to fungal pathogens in various crops such as barley, tomato, pea, and wheat (Bai et al., 2008; Büschges et al., 1997; Humphry et al., 2011; Wang et al., 2014). In *Arabidopsis*, *DMR6* is known to positively affect the susceptibility to downy mildew oomycete *Hyaloperonospora parasitica* (Van Damme et al., 2008). RAP2.1 is a DREB‐type, EAR‐motif‐containing transcriptional repressor known to negatively regulate plant stress responses (Dong & Liu, 2010). The upregulation of these S genes in both the susceptible (C 214 and JG 62) genotypes indicates their involvement in the susceptibility of these genotypes to *F. oxysporum* infection. In the present study, we identified many genes exhibiting similar expression in two resistant genotypes (WR 315 and ICCV 05528) but different in NILs (NIL 01 and NIL 02). The expression of these genes in NILs was similar to their recurrent parent (C 214), demonstrating the use of NILs in distinguishing the genes that are most likely to be implicated in resistance. Similar observations are reported from other transcriptome studies where NILs helped reduce genetic background noise and identify candidate genes responsible for resistance/tolerance (Kim et al., 2011; Zhang et al., 2018).

In summary, the comparative analysis of the transcriptomes of resistant and susceptible chickpea genotypes during *F. oxysporum* f. sp. *ciceris* infection has provided insights into different genes encoding for CDPK, MLO, DMR6, WRKY25, Dirigent, NBS‐LRR, etc., and pathways that coherently induce plant defense mechanisms through basal and induced resistance. The in‐depth understanding of the molecular basis of FW resistance and the candidate genes identified in the study might contribute to the deployment of either genetic engineering or molecular breeding approaches to develop FW‐resistant chickpea varieties.

## AUTHOR CONTRIBUTIONS

**Vanika Garg**: Formal analysis, Writing‐original draft. **Annapurna Chitikineni**: Resources. **Mamta Sharma**: Methodology. **Raju Ghosh**: Methodology. **Srinivasan Samineni**: Resources. **Rajeev K. Varshney**: Conceptualization, Investigation, Writing‐review & editing. **Himabindu Kudapa**: Conceptualization, Investigation, Writing‐review & editing.

## CONFLICT OF INTEREST STATEMENT

The authors declare no conflicts of interest.

## Supporting information

Supplemental Table S1. Statistics of reads mapped on different genomic regions of the chickpea reference genome.
Supplemental Table S2. List of differentially expressed genes, their chromosomal localization, and functional annotation.
Supplemental Table S3. Functional annotation (Biological Process Category) of DEGs identified from different chickpea genotypes under control and stress conditions.
Supplemental Table S4. Functional annotation (Cellular Component Category) of DEGs identified from different chickpea genotypes under control and stress conditions.
Supplemental Table S5. Functional annotation (Molecular Function Category) of DEGs identified from different chickpea genotypes under control and stress conditions.
Supplemental Table S6. List of differentially expressed transcription factor encoding genes.

## Data Availability

The RNA‐sequencing data generated in the study are available in NCBI Sequence Read Archive (SRA, http://www.ncbi.nlm.nih.gov/sra) with the BioProject ID PRJNA761666.
