# Supplementary material for: Transcriptome profiling reveals the expression and regulation of genes associated with Fusarium wilt resistance in chickpea (Cicer arietinum L.)
Source: Plant Genome. 2023 May 21;16(3):e20340. doi: 10.1002/tpg2.20340 (PMC12807412; doi:10.1002/tpg2.20340)
Supplement: Supplementary file 1 [file TPG2-16-e20340-s001.pdf]

The Plant Genome OPEN ACCESS

Crop Science Society of America

Transcriptome profiling reveals the expression and regulation of genes associated with Fusarium wilt resistance in chickpea (*Cicer arietinum* L.)

Journal:	*The Plant Genome*
Manuscript ID	TPG-2023-01-0002.R1
Wiley - Manuscript type:	Original Article
Date Submitted by the Author:	20-Mar-2023
Complete List of Authors:	Garg, Vanika; Murdoch University, Centre for Crop & Food Innovation<br>Chitikineni, Annapurna; Murdoch University<br>Sharma, Mamta; International Crops Research Institute for the Semi-Arid Tropics<br>Ghosh, Raju; International Crops Research Institute for the Semi-Arid Tropics<br>Samineni, Srinivasan; International Crops Research Institute for the Semi-Arid Tropics<br>Varshney, Rajeev; Murdoch University, Centre for Crop & Food Innovation<br>Kudapa, Himabindu; ICRISAT, CEG
Keywords:	biotic stress, near-isogenic lines, molecular mechanism, chickpea, RNA-seq

SCHOLARONE™
Manuscripts

Core Ideas

As part of the submission process, we ask authors to prepare highlights of their article. The highlights will consist of 3 to 5 bullet points that convey the core findings of the article and emphasize the novel aspects and impacts of the research on scientific progress and environmental problem solving.

The purpose of these highlights is to give a concise summary that will be helpful in assessing the suitability of the manuscript for publication in the journal and for selecting appropriate reviewers. If the article is accepted the highlights may also be used for promoting and publicizing the research.

Core Idea 1: Transcriptome sequencing of FW responsive chickpea genotypes elucidates a set of 5182 DEGs

Core Idea 2: Identified DEGs were involved in defense response, cell wall biogenesis, secondary metabolism & disease resistance

Core Idea 3: A total of 287 DEGs co-localized with previously reported QTLs for FW resistance

Core Idea 4: NILs reduce genetic background in transcriptome studies

Core Idea 5: Transcriptome dataset with candidate genes is an excellent functional genomics resource for resistance breeding

For Review Only

Transcriptome profiling reveals the expression and regulation of genes associated with Fusarium wilt resistance in chickpea (*Cicer arietinum* L.)

Vanika Garg[1,2], Annapurna Chitikineni[1,2], Mamta Sharma[2], Raju Ghosh[2], Srinivasan Samineni[2], Rajeev K. Varshney[1,2,*], Himabindu Kudapa[2,*]

[1]Centre for Crop and Food Innovation, State Agricultural Biotechnology Centre, Food Futures Institute, Murdoch University, Murdoch, WA, Australia

[2]International Crops Research Institute for the Semi-Arid Tropics (ICRISAT), Hyderabad, India

*Correspondence: himabindu.kudapa@icrisat.org; Rajeev.Varshney@murdoch.edu.au

For Review Only

Core Ideas

- Transcriptome sequencing of FW responsive chickpea genotypes elucidates a set of 5182 DEGs
- Identified DEGs were involved in defense response, cell wall biogenesis, secondary metabolism & disease resistance
- A total of 287 DEGs co-localized with previously reported QTLs for FW resistance
- NILs reduce genetic background in transcriptome studies
- Transcriptome dataset with candidate genes is an excellent functional genomics resource for resistance breeding

For Review Only

Abbreviations

BLAST	:	Basic Local Alignment Search Tool
CDPKs	:	Calcium-dependent protein kinases
DEGs	:	Differentially expressed genes
Dpi	:	Days post inoculation
FPKM	:	Fragments Per Kilobase of transcript per Million mapped reads
FW	:	Fusarium wilt
GO	:	Gene Ontology
GST	:	Glutathione-S-transferase
HSPs	:	Heat Shock Proteins
ICRISAT	:	International Crops Research Institute for the semi-arid tropics
KEGG	:	Kyoto Encyclopedia of Genes and Genomes
MAPK	:	Mitogen-activated protein kinase
NBS-LRR	:	Nucleotide-binding site–leucine-rich repeat
NCBI	:	National Centre for Biotechnology Information
NILs	:	Near Isogenic Lines
PPR	:	Pentatricopeptide repeat protein
QTL	:	Quantitative trait locus
ROS	:	Reactive Oxygen Species
SSR	:	Simple Sequence Repeats
TF	:	Transcription factor

For Review Only

Abstract

Fusarium wilt (FW) is one of the most significant biotic stresses limiting chickpea production worldwide. To dissect the molecular mechanism of FW resistance in chickpea, comparative transcriptome analyses of contrasting resistance sources of chickpea genotypes under control and *Fusarium oxysporum* f. sp. *ciceris* (*Foc*) inoculated conditions were performed. The high-throughput transcriptome sequencing generated about 1137 million sequencing reads from 24 samples representing two resistant genotypes, two susceptible genotypes, and two near-isogenic lines under control and stress conditions at two-time points (7th and 12th-day post-inoculation). The analysis identified 5182 differentially expressed genes (DEGs) between different combinations of chickpea genotypes. Functional annotation of these genes indicated their involvement in various biological processes such as defense response, cell wall biogenesis, secondary metabolism, and disease resistance. A significant number (382) of transcription factor encoding genes exhibited differential expression patterns under stress. Further, a considerable number of the identified DEGs (287) co-localized with previously reported QTLs for FW resistance. Several resistance/susceptibility related genes, such as *SERINE/THREONINE PROTEIN KINASE*, *DIRIGENT*, *MLO* exhibiting contrasting expression patterns in resistant and susceptible genotypes upon *Foc* inoculation were identified. The results presented in the study provide valuable insights into the transcriptional dynamics associated with FW stress response in chickpea and provide candidate genes for the development of disease-resistant chickpea cultivars.

Keywords: RNA-seq, biotic stress, near-isogenic lines, chickpea, molecular mechanism

For Review Only

Introduction

Chickpea (*Cicer arietinum* L.) is one of the most important grain legumes, with an annual production of 15.87 million tons (FAOSTAT, 2021). It is a self-pollinated, diploid annual crop with a genome size of ~740 Mb and grown predominantly in arid and semi-arid regions of the world (Varshney et al., 2013). The importance of chickpea lies in its intrinsic potential for symbiotic nitrogen fixation and its dietary proteins, vitamins, and essential minerals. Chickpea production is essential for food security and improving the nutritional quality of diets for people living mainly in developing countries. Global chickpea production has seen a significant rise in recent years owing to the research efforts that have resulted in the development and release of improved varieties (FAOSTAT, 2021). However, meeting the growing demand calls for higher productivity gains in the chickpea crop. Further improving crop productivity will require sustainable management of devastating diseases like Fusarium wilt (FW), and Ascochyta blight (AB), which put chickpea cultivation at great risk. Fusarium wilt (FW), caused by a soil-borne fungus, *Fusarium oxysporum* f. sp. *ciceris* (*Foc*) is one of the most prevalent diseases of chickpea worldwide. FW is reported to cause yield losses varying from 10 to 100% depending on varietal susceptibility and suitable climatic conditions (Sharma et al., 2012). Since FW is a soil-borne disease, its management through crop rotation strategies or chemical control is difficult. Therefore, using varieties resistant to FW is the most cost-effective, promising, and environmentally sustainable strategy to manage the disease. In this direction, several QTLs for FW resistance have been reported to develop FW-resistant cultivars via molecular breeding (Sabbavarapu et al., 2013; Varshney et al., 2014; Garg et al., 2018). However, genetic variability in the pathogen is high, causing varied levels of virulence and leading to the breakdown of resistance in available sources (Sharma et al., 2012). To expedite the molecular breeding process or to develop resistant varieties via gene editing approaches, it is imperative to have deeper insights into the molecular mechanism of FW resistance in chickpea.

CSSA 5585 Guilford Rd., Madison, WI 53711

For Review Only

Upon stress, plants undergo a series of physiological, morphological, molecular, and biochemical changes mediated by a network of genetic regulations. These genetic regulations mostly include modifications in gene expression at both transcriptional and post-transcriptional levels. The identification of genes and their expression quantification have been concrete activities in molecular biology ever since the establishment of RNA's role as the fundamental mediator between the genome and proteome. The study of gene expression profiles under stress can help to pinpoint important components of resistance mechanisms. The advent of RNA-seq has facilitated transcript identification and gene expression quantification in a robust manner. Several transcriptomic studies have successfully delineated the molecular mechanisms of drought, salinity, and Ascochyta blight stress in chickpea (Mashaki et al., 2018; Garg et al., 2019). Different studies using the LongSAGE, or microarray approach were performed for only two genotypes (Upasani et al., 2017; Ashraf et al., 2018); however, high-throughput and comprehensive analysis of genes involved in FW resistance is not yet available.

To get a deeper understanding of FW resistance in chickpea, we employed RNA-seq to analyze the transcriptomes of roots of six contrasting chickpea genotypes for FW resistance under control and stress conditions at two different time points. We investigated these data to unravel transcriptome dynamics associated with FW stress in chickpea and identified key differences that determine disease resistance in chickpea. The genes, transcription factors, and biological pathways showing specific and differential expression patterns between resistant and susceptible genotypes were identified. The identified genes, pathways and molecular interactions regulating disease resistance against *F. oxysporum* in chickpea provide potential candidate targets for the development/improvement of Fusarium wilt resistance chickpea cultivars.

For Review Only

Materials and Methods

Plant material

A total of four chickpea genotypes and two near-isogenic lines with contrasting phenotypes for Fusarium wilt resistance were selected for the study. The genotype, WR 315, is a desi landrace resistant to FW (*Foc* race 1A, race 2, race 4, and race 5) (Gaur and Chaturvedi, 2004), while the genotype ICCV 05528 is a breeding line with a moderate level of disease resistance (Sharma et al., 2012). The C 214 and JG 62 are elite desi cultivars susceptible to FW (early wilter) (Pande et al., 2012). The two near-isogenic lines (NILs) - NIL 01 (NIL 10057) and NIL 02 (NIL 10058) are obtained from introgression of *foc*1 locus conferring resistance to race 1 of FW from WR 315 into the genetic background of C 214 (Sabbavarapu et al., 2013; Varshney et al., 2014).

Stress treatment

For the imposition of stress, seedling raising, and inoculum preparation were carried out as described earlier (Sharma et al., 2010). In brief, all the six lines selected were raised in trays filled with sterilized river sand in a greenhouse maintained at 25±1°C for ten days. *Foc* race 1 inoculum was prepared from a single conidial culture of *F. oxysporum* f. sp. *ciceris* isolated from wilt infected plants collected from an ICRISAT wilt sick plot. To prepare mass inoculum, a 7-mm disc of actively growing *F. oxysporum* f. sp. *ciceris* culture was put into a 250 ml conical flask containing 100 ml of sterilized potato dextrose broth and incubated for seven days in an incubator shaker (25±1°C and 125 rpm). The culture was homogenized in sterilized distilled water and adjusted to 6 x 10^5 conidia ml^{-1} using a haemocytometer for use as an inoculum (as per Sharma et al., 2010). Ten-day-old seedlings of each test line grown in sterilized river sand were uprooted, cleaned with tap water, and inoculated by dipping in inoculum suspension for 1-2 minutes to enable conidia to adhere to the roots. Inoculated

For Review Only

seedlings were transplanted in pre-irrigated sterile vertisol and sand (3:1) in pots and incubated in a greenhouse at 25±3°C.

Sample collection and RNA isolation

The root tissues from both uninoculated/control and inoculated/stressed plants were harvested at 7th days post-inoculation (dpi) and 12th dpi in sets of three biological replicates. Total RNA was isolated using the "NucleoSpin® RNA Plant" kit (Macherey-Nagel, Düren, Germany). Further, the quality of isolated RNA was assessed using Agilent 2100 Bioanalyzer (Agilent Technologies, Santa Clara, CA, USA) and Nanodrop 8000 Spectrophotometer (Thermo Fisher Scientific, Waltham, MA, USA).

Transcriptome sequencing and reference-guided assembly

High-quality total RNA (RIN ≥ 8) was subjected to library construction using TruSeq RNA Sample Prep Kit v2 (Illumina Inc., San Diego, CA, USA) as described previously (Garg et al., 2019). The libraries were sequenced on Illumina HiSeq 2500 platform using a 150 bp-paired end strategy. In total, 24 samples representing six different genotypes (C 214, JG 62, ICCV 05528, WR 315, NIL 01, NIL 02) under control and *F. oxysporum* inoculated conditions at two-time points (7th and 12th dpi) were sequenced. The raw reads generated from transcriptome sequencing were processed to remove primer/adaptor contamination and low-quality reads (>20% of the bases with a Phred quality score < 10) using Trimmomatic v0.35 with parameters "ILLUMINACLIP:adaptors.fa:2:30:10 SLIDINGWINDOW:10:20 MINLEN:50" (Bolger et al., 2014). The paired-end clean reads were mapped to the chickpea reference genome Cicar.CDCFrontier_v2.0 (Garg et al., 2022) using HISAT2 v2.1.0 with default parameters (Kim et al., 2019). The mapped reads from each sample were assembled using Cufflinks v2.2.1 with default parameters to generate reference-guided assemblies (Trapnell et al., 2012). These

For Review Only

assemblies were merged to generate a consensus assembly using Cuffmerge with default parameters, and the assembly thus obtained was used for downstream analysis.

Expression profiling and identification of DEGs

The transcript abundance was estimated based on Fragments Per Kilobase of transcript per Million mapped reads (FPKM) using Cufflinks. Transcripts with FPKM ≥1 in any of the samples and quantification status as 'OK' were considered to be expressed and were used for downstream analysis. The differential gene expression between different samples was estimated using Cuffdiff (Trapnell et al., 2013). DEGs were identified between different samples under control (non-inoculated), and *F. oxysporum* inoculated conditions at different time points. The different pairwise combinations included stress versus control samples of each genotype at 7th and 12th dpi; resistant versus susceptible genotypes under control conditions at 7th and 12th dpi; and resistant versus susceptible genotypes under FW stress conditions at both 7th and 12th dpi. The DEGs with log2 fold change ≥ 2 (up-regulated) and/ or ≤ −2 (down-regulated) and an FPKM ≥2 for either of the samples in each pair-wise comparison were considered to be significantly differentially expressed. The log2 transformed FPKM values of the DEGs were further subjected to *k*-means clustering using pheatmap package in R/Bioconductor.

Annotation and GO enrichment of DEGs

To predict putative function, the identified DEGs were subjected to similarity search using BLASTX (E-value cut-off of ≤10–5) against the National Center for Biotechnology Information non-redundant Viridiplantae protein database and were further assigned GO terms using Blast2GO v4.1.9 (Conesa et al., 2005). To pinpoint the overrepresented functional categories in DEGs across different sample comparisons, an R package GOseq (Young et al., 2010) was used. GO terms displaying a corrected *P*-value of ≤ 0.05 for a given set of genes

For Review Only

were considered to be significantly enriched. The enriched GO terms were summarised and visualized using REVIGO (Supek et al., 2011).

Pathway and TF analysis

The identified DEGs were studied for their involvement in different pathways using the Kyoto Encyclopedia of Genes and Genomes (KEGG) Automatic Annotation Server (Moriya et al., 2007). The pathway enrichment analysis was performed based on a hypergeometric model (Boyle et al., 2004) with a significance threshold of *P*-value of 0.05. The enriched pathways were plotted as a scatter plot using data visualisation R package ggplot2 (Wickham, 2016). Further, the transcription factor encoding genes were predicted using a similarity search against the PlantTFDB database (Tian et al., 2020) with an E-value cut-off of 1E-10. For co-localization studies, information about the FW resistance QTLs was obtained from previous studies (Sabbavarapu et al., 2013; Patil et al., 2014; Garg et al., 2018). The sequence similarity search using BLASTN was performed to obtain the physical locations of the markers associated with FW resistance QTLs in the chickpea reference genome. Only hits with 100% query and subject coverage were retained.

For Review Only

Results

Transcriptome sequencing and reference-guided assembly

In the present study, a total of 24 samples representing four chickpea genotypes (WR 315-resistant, ICCV 05528-moderately resistant, C 214-susceptible, JG 62-susceptible) and two near-isogenic lines (NIL 10057 and NIL 10058, designated as NIL 01 and NIL 02, respectively) under control and stress (*F. oxysporum* inoculated) conditions at two (7th dpi and 12th dpi) time points were subjected to transcriptome sequencing. The two time points identified for this study were selected based on the earlier reports in legumes that the initial symptoms begin at 7 days post-inoculation and express to the maximum at 12 days post-inoculation (Biswas et al., 2020). The near-isogenic lines (NIL 01 and NIL 02) used in the present study were obtained by introgressing FW-resistant QTLs from WR 315 into C 214 (Varshney et al., 2014). In the study, the samples are named genotype-condition-dpi for ease of understanding. For instance, C 214-C-7d represents genotype C 214 under control conditions at 7th dpi. A total of 1137 million reads were generated from the paired-end sequencing of these 24 samples. After trimming and filtering low-quality reads, a set of 1110 million high-quality reads were retained and mapped on the recently available chickpea genome (Garg et al., 2022). Overall, about 95% of the high-quality reads were mapped on the reference genome (**Table 1**). The sequencing data and alignment statistics reflect very high-quality transcriptome sequencing. On average, 93.27% of the reads were mapped to the exonic region, 0.83% to the intronic region, and 5.90% to intergenic regions of the chickpea genome (**Supplemental Table S1**). The mapped reads from all 24 samples were used to generate reference-guided assembly using the Cufflinks-Cuffmerge pipeline. This assembly generated 76,382 transcripts representing 27,736 gene loci, including 25,695 known and 2041 novel gene loci.

Global gene expression analysis

For Review Only

The normalized expression level (FPKM) of each gene was estimated in all the samples. The genes with very low expression values (see Materials and Methods) in all 24 samples were filtered out. After filtering, a set of 21,609 genes exhibiting expression in at least one of the samples was obtained. The number of expressed genes varied from a minimum of 18,041 genes in WR 315-S-12d to a maximum of 18,872 genes in ICCV 05528-C-12d (**Table 2**). Further, based on the expression level, the genes were classified into highly expressed (FPKM ≥ 10) and low/moderately expressed genes (FPKM ≥ 1 and < 10). The number of low/moderately expressed genes was less than highly expressed genes across all the samples in the study. The number of low/moderately expressed genes ranged from 7563 in JG 62-S-12d to 8390 in ICCV 05528-C-12d, whereas highly expressed genes ranged from 10,257 in ICCV 05528-S-12d to 10,671 in NIL 01-C-12d (**Table 2**).

Identification of differentially expressed genes in response to FW stress

The fold change of each gene was calculated across different pairwise combinations using Cuffdiff. A gene was considered differentially expressed if it exhibited a log_2 fold change of ≥ 2 (up-regulated) or ≤ -2 (down-regulated). Using this criterion, a total of 5182 genes were differentially expressed for at least one sample combination (**Supplemental Table S2**). The differential expression patterns between control and stress samples of each genotype were studied at both stages of infection (7th and 12th dpi). At 7th dpi, the maximum DEGs were identified in the JG 62 genotype (577 up-regulated; 357 down-regulated), followed by WR 315 (327 up-regulated; 353 down-regulated) (**Figure 1a-c**). At 12th dpi, the maximum transcriptional changes were observed in WR 315 genotype with differential expression of 1078 genes (656 up-regulated; 422 down-regulated). The minimum number of DEGs was identified between C 214-C-7d and WR 315-C-7d (63), and the maximum was identified between ICCV 05528-S-12d and JG 62-S-12d (1452). Under stress, the pattern of up-regulation was more profound at the 12th dpi than the 7th dpi across all genotypes (**Figure 1c**).

For Review Only

Further, an overlap of DEGs between different genotypes under control and stress conditions at both stages of infection was studied. A significant number of DEGs were unique to each combination. In total, 1230 genes showed genotype-specific differential expression patterns under stress at 7th dpi. At 12th dpi, 1549 DEGs were genotype-specific. At 7th dpi, the maximum number of genotype-specific DEGs were found in JG 62 (611) and the minimum in NIL 02 (49) (**Figure 1d**). Similarly, at 12th dpi, WR 315 (724) and NIL 02 (22) had the maximum and minimum genotype-specific DEGs, respectively (**Figure 1e**). A total of 1915 genes exhibited differential expression patterns at both 7th and 12th dpi upon infection in any genotypes. These findings indicate an extensive genotype- and stress stage-specific response of chickpea to *Foc* infection.

Genome-wide distribution of DEGs and localization with FW QTLs

The identified DEGs were studied for their distribution in the chickpea genome. Out of 5182 DEGs, 5108 were mapped to the pseudomolecules of the chickpea genome and the remaining on scaffolds (**Figure 2**). The highest number of DEGs were present on pseudomolecule Ca6_v2.0 (803), followed by pseudomolecule Ca4_v2.0 (741), and the lowest were present on pseudomolecule Ca8_v2.0 (431). Several QTLs have been reported for FW resistance to date (Sabbavarapu et al., 2013; Patil et al., 2014; Garg et al., 2018). The identified DEGs were further co-localized with previously reported FW resistance QTLs. By deploying the physical position of the SSR markers on the chickpea pseudomolecules, these QTLs were mapped on pseudomolecules Ca2_v2.0, Ca4_v2.0, and Ca6_v2.0 of the chickpea reference genome (Garg et al., 2022). Further, a total of 287 out of the 5182 DEGs, were co-localized with seven FW resistance QTLs. Of these 287 co-localized DEGs, 39 were novel. The maximum DEGs were

For Review Only

present in two QTLs, namely, *FW-Q-APR-2-1* (87, Garg et al., 2018) and Wilt 2 (76, Patil et al., 2014) located on pseudomolecule Ca2_v2.0 (**Figure 2**).

Further, functional annotations were assigned to the identified DEGs using BLASTX. From the total, 4767 DEGs were annotated, and 4213 of these DEGs were assigned GO terms. A total of 13,823 GO terms were retrieved for these DEGs, and these GO terms were evenly assigned to biological process (4261), molecular function (5813), and cellular component (3749) categories. GO enrichment analysis of the identified DEGs suggested the enrichment of GO terms such as defense response (GO:0006952), response to reactive oxygen species (GO:0000302), ion homeostasis (GO:0050801), signal transduction (GO:0007165), defense response to fungus (GO:0050832), cell wall biogenesis (GO:0042546), response to chitin (GO:0010200), brassinosteroid biosynthetic process (GO:0016132), abscisic acid-activated signaling pathway (GO:0009738), and response to auxin (GO:0009733) in the biological process category (**Figure 3a; Supplemental Table S3**). Among the enriched GO terms related to biological processes, response to chitin, defense response, lipid transport, abscisic acid-activated signaling pathway, MAPK cascade (GO:0000165), and flavonoid biosynthesis (GO:0009813) were mainly associated with genes showing increased expression upon stress whereas genes showing downregulation were related to circadian rhythm (GO:0007623) and response to ethylene (GO:0009723) etc. Among the cellular component category, extracellular region (GO:0005576), plant-type cell wall (GO:0009505), apoplast (GO:0048046), photosystem (GO:0009521), and anchored component of plasma membrane (GO:0046658) were the most overrepresented GO terms (**Supplemental Table S4**). In the molecular function category, oxidoreductase activity (GO:0016491), transcription regulator activity (GO:0140110), protein serine/threonine kinase activity (GO:0004674), peroxidase activity (GO:0004601), hydrolase activity (GO:0016798), and monooxygenase activity (GO:0004497) were the most significantly enriched GO terms **(Supplemental Table S5**).

For Review Only

Further, pathway analysis of the DEGs was performed to identify pathways enriched in response to FW stress in chickpea. We identified a total of 130 diverse pathways using the KEGG database. The enrichment analysis (p-value < 0.05) indicated that genes involved in the biosynthesis of secondary metabolites (ko01110), MAPK signaling pathway (ko04016), plant-pathogen interaction (ko04626), plant hormone signal transduction (ko04075), flavonoid biosynthesis (ko00941), and fatty acid elongation (ko00062) were significantly differentially expressed **(Figure 3b)**.

For Review Only

Transcription factors (TF), being the upstream regulatory proteins, play significant roles under both abiotic and biotic stresses in plants (Nuruzzaman et al., 2013; Bhoghireddy et al., 2020). The reference-guided transcriptome assembly identified members of 58 TF families represented by 1564 genes using the PlantTFDB database (Tian et al., 2020). Among these, 382 TF-related genes from 39 families were found to show differential expression patterns under FW stress (**Supplemental Table S6**). The maximum members of the ERF family (56) were differentially expressed, followed by members of bHLH (43), WRKY (35), MYB (32), and C2H2 (23) TF families. In addition, NAC (19), GRAS (17), Dof (17), and bZIP (16) TF families also exhibited differential expression patterns under stress conditions (**Figure 3c)**. Taken together, these analyses provided an important resource for identifying specific processes, functions, and pathways associated with FW resistance in chickpea.

Expression trends across resistant and susceptible chickpea genotypes under FW stress

In order to gain deeper insights into the molecular mechanisms of resistance induced by *Foc*, the identified DEGs were investigated for their expression profiles in different genotypes under stress conditions. A group of genes mainly associated with defense response, phosphorylation, redox homeostasis, and signal transduction exhibited a trend of up-regulation in all the

genotypes irrespective of resistance/susceptibility at both 7th and 12th dpi. Genes encoding for pathogenesis-related proteins (*Ca_v2.0_23074* and *Ca_v2.0_19090*), endochitinase (*Ca_v2.0_21064*), glutathione-S-transferase (*Ca_v2.0_16473*), serine-threonine kinase (*Ca_v2.0_17396*), peroxidases (*Ca_v2.0_09505* and *Ca_v2.0_10250*), and several classes of heat shock protein (HSPs; *Ca_v2.0_05664*, *Ca_v2.0_13212*, *Ca_v2.0_14887*, and *Ca_v2.0_17117*) were part of this group (**Figure 4**). Similarly, another group of genes related to growth (*Ca_v2.0_06823*), photosynthesis (*Ca_v2.0_10872* and *Ca_v2.0_23888*), leaf development (*Ca_v2.0_01980*), chlorophyll biosynthesis (*Ca_v2.0_03879*), and flower development (*Ca_v2.0_11517*) showed a general trend of down-regulation in all genotypes at both 7th and 12th dpi (**Figure 4**). Upon FW infection, a highly genotype-specific response was also observed. Several stress-responsive genes such as NBS-LRR (*Ca_v2.0_06556*), chitinase (*Ca_v2.0_09545*), and glutathione-S-transferase (*Ca_v2.0_06398*) were highly up-regulated specifically in WR 315 upon stress. In contrast, the expression levels of these genes were considerably lower or down-regulated in the moderately resistant and susceptible genotypes.

Further, we identified key genes that might be responsible for different phenotypes of chickpea genotypes to FW stress. We investigated those genes that showed similar expression among resistant (WR 315 and ICCV 05528) and near-isogenic lines (NIL 01 and NIL 02) but contrasting expression patterns in susceptible genotypes (C 214 and JG 62). Upon FW infection, genes (Cluster I) involved in oxidation-reduction (*Ca_v2.0_07841*), fatty acid biosynthesis (*Ca_v2.0_24909*), and defense response (*Ca_v2.0_20495* and *Ca_v2.0_20402*) exhibited induced expression at both 7th and 12th dpi in resistant genotypes (**Figure 5**). Cluster II included genes showing significant up-regulation in resistant genotypes during early infection (7th dpi). It contained genes involved in pathogen recognition such as LRR receptor-like serine/threonine-protein kinase (*Ca_v2.0_05099*, *Ca_v2.0_16336*, and *Ca_v2.0_10346*), ion homeostasis (*Ca_v2.0_09532*), cell wall loosening such as expansins (*Ca_v2.0_17389*,

For Review Only

Ca_v2.0_02587, *Ca_v2.0_03698*, and *Ca_v2.0_14445*), DNA repair (*Ca_v2.0_04988* and *Ca_v2.0_22002*), stress-responsive genes such as pentatricopeptide repeat proteins (PPR) (*Ca_v2.0_04925*) and Dirigent (*Ca_v2.0_06536*). Several TFs like MYB86 (*Ca_v2.0_01744*), and bHLH030 (*Ca_v2.0_04133*) were also part of this cluster. Cluster III consisted of genes showing a general trend of up-regulation in all resistant genotypes during the later stage of infection (12th dpi). Several genes such as expansin-A8 (*Ca_v2.0_04379*), SKP1-like protein 14 (*Ca_v2.0_01774*), scarecrow-like protein 34 (*Ca_v2.0_19108*), GRIM REAPER-like protein (*Ca_v2.0_02038*) were part of this cluster. Further, genes exhibiting induced expression in susceptible genotypes at either 7th or 12th dpi were grouped in Cluster IV. Genes encoding for senescence-associated protein (*Ca_v2.0_00473* and *Ca_v2.0_03058*), accelerated cell death 11 (*Ca_v2.0_00854*), desiccation-related protein (*Ca_v2.0_06792*), leucoanthocyanidin dioxygenase-like protein (*Ca_v2.0_06483*), late embryogenesis abundant protein (*Ca_v2.0_17623*), pectinesterase (*Ca_v2.0_06107*), calcium-transporting ATPase (*Ca_v2.0_07475*), downy mildew resistance 6 (DMR6; *Ca_v2.0_04097*) Mildew Locus O (MLO) like protein 2 (*Ca_v2.0_24001*) and SRG1 (*Ca_v2.0_04646*; *Ca_v2.0_01927*) showed up-regulation in susceptible genotypes during early stages of infection. TFs encoding for NAC (*Ca_v2.0_00358* and *Ca_v2.0_21566*), MYB (*Ca_v2.0_18711* and *Ca_v2.0_09100*), ERF (*Ca_v2.0_14789*, *Ca_v2.0_20575*, *Ca_v2.0_14916*, *Ca_v2.0_17233*, *Ca_v2.0_22990*, and *Ca_v2.0_22999*) and WRKY25 (*Ca_v2.0_13374*) were up-regulated in susceptible genotypes upon *Foc* infection (**Figure 5**).

For Review Only

Discussion

Understanding the molecular basis of variability in stress response is crucial for all research designed to develop new chickpea cultivars with FW resistance. In this direction, the present study reported a comprehensive transcriptome analysis of 24 samples representing six well-characterised chickpea genotypes (C 214, JG 62, ICCV 05528, WR 315, NIL 01, and NIL 02) under control and stress conditions at two stress stages. The data generated from these samples were used to construct a reference-guided assembly that assembled 27,736 genes and comparison of these genes with the gene models of the chickpea reference genome led to the identification of 2041 novel genes from unannotated loci, thus demonstrating the potential of RNA-seq in the identification of novel genes. Similar results were also reported from other transcriptome studies in chickpea (Kudapa et al., 2018; Garg et al., 2019; Kudapa et al., 2023).

An extensive transcriptional reprogramming was observed in chickpea plants under FW stress at 7th and 12th dpi. A total of 5182 genes showed differential expression patterns between contrasting stress-responsive genotypes under control and stress conditions at different stages of infection. A significant fraction of identified DEGs were present in the QTL regions associated with FW resistance. The functional annotation, GO enrichment, and pathway analysis of these differentially expressed genes throw light on the FW resistance mechanism of chickpea. Several genes involved in the recognition of pathogens, ion homeostasis, cell wall modifications, formation of reactive oxygen species (ROS), signal transduction and defense response were significantly enriched during FW infection. Pathogen invasion is known to affect the structure and integrity of the plant cell wall. In this study, several genes associated with "cell wall biogenesis", "cell wall organization", and "cell wall modification", were differentially expressed, suggesting the role of structural defense genes in FW response in chickpea similar to the observations reported from other legumes (Sharma et al., 2016; Chang et al., 2021). Several pathways related to specific physiological, metabolic, and biochemical

For Review Only

changes were also up-regulated in the present study. Transcriptome studies conducted in various plant species have reported the enrichment of similar GO terms and pathways in response to fungal stress (Nayak et al., 2017; Garg et al., 2019). Defense response involves several TFs which regulate the target gene expression by specifically binding to cis-acting elements in the promoter regions (Park et al., 2001; Wang et al., 2009). In the present study, different classes of TFs, such as bHLH, WRKY, ERF, and bZIP exhibited differential expression patterns upon FW stress similar to those observed in previous studies (Wang et al., 2009; He et al., 2016).

When chickpea plants were challenged with *Foc*, several genes related to defense response, phosphorylation, redox homeostasis, and signal transduction exhibited significantly increased expression levels in all the genotypes. In contrast, genes involved in basic biological processes such as plant growth and photosynthesis were down-regulated in all the genotypes. These genes showing similar expression in all the genotypes upon stress might be involved in the basal defense of chickpea against *F. oxysporum* infection. Under both biotic and abiotic stresses, the plant cells accumulate ROS as a part of the basal defense response. Several antioxidant enzymes such as flavonoids and peroxidases, can quench the ROS generated due to infection (Gupta et al., 2019). The present study also demonstrated increased expression of peroxidases and HSPs in all genotypes upon FW infection. Genes encoding for peroxidases were also found to positively regulate defense responses against FW stress in a previous study (Ashraf et al., 2018). Various studies have reported that HSPs play an integral role in developing resistance against biotic stresses by interacting with other defense-related proteins. The increased expression of HSP70 in this study is in concurrence with a recent report on powdery mildew (caused by *Golovinomyces orontii*) infection in sunflower (Kallamadi et al., 2018).

For Review Only

Several studies have reported an increase in intracellular Ca^{2+} levels in plant cells in response to both biotic and abiotic stress (Li et al., 2008; Vadassery and Oelmüller, 2009). The increased Ca^{2+}levels induce defense genes via the activation of ion fluxes at the plasma membrane, an oxidative burst and MAPK activation. In the present study, several genes encoding for calcium-dependent protein kinases (CDPKs), calcium transporting ATPase, and calmodulin-like proteins, the major components of calcium signalling, were differentially expressed in resistant genotypes during the early stages of infection, suggesting the role of calcium signalling in FW response in chickpea. Similar observations were also reported from a previous study on FW in chickpea (Upasani et al., 2017). The increased expression of different stress-responsive genes like serine-threonine kinases, dirigent, and PPR protein was also seen in all resistant genotypes at different time points. Dirigent proteins are known to be implicated in the regulation of lignan biosynthesis and are important for secondary metabolism and pathogen resistance (Li et al., 2017). Their increased expression in resistant genotypes upon stress might be indicative of their involvement in FW resistance. In contrast, several genes encoding for senescence-associated, accelerated cell death, MLO, DMR6, WRKY25 and RAP2.1 were up-regulated in susceptible genotypes upon infection. Genes such as *MLO*, *DMR6*, and *WRKY25* are classified as susceptibility (S) genes and are known to mediate plant-pathogen compatibility and facilitate pathogen infection (Henningsen et al., 2021). Loss-of-function mutations of *MLO* genes are reported to reduce susceptibility to fungal pathogens in various crops such as barley, tomato, pea and wheat (Büschges et al., 1997; Bai et al., 2008; Humphry et al., 2011; Wang et al., 2014). In *Arabidopsis*, *DMR6* is known to positively affect the susceptibility to downy mildew oomycete *Hyaloperonospora parasitica* (Van Damme et al., 2008). RAP2.1 is a DREB-type, EAR-motif-containing transcriptional repressor known to negatively regulate plant stress responses (Dong and Liu, 2010). The upregulation of these S genes in both the susceptible (C 214 and JG 62) genotypes indicates their involvement in the susceptibility of these genotypes to *F. oxysporum* infection. In the present study, we identified many genes exhibiting similar

For Review Only

expression in two resistant genotypes (WR 315 and ICCV 05528) but different in NILs (NIL 01 and NIL 02). The expression of these genes in NILs was similar to their recurrent parent (C 214), demonstrating the use of NILs in distinguishing the genes that are most likely to be implicated in resistance. Similar observations are reported from other transcriptome studies where NILs helped reduce genetic background noise and identify candidate genes responsible for resistance/tolerance (Kim et al., 2011; Zhang et al., 2018).

In summary, the comparative analysis of the transcriptomes of resistant and susceptible chickpea genotypes during *F. oxysporum* f. sp. *ciceris* infection has provided insights into different genes encoding for CDPK, MLO, DMR6, WRKY 25, Dirigent, NBS-LRR etc and pathways that coherently induce plant defense mechanisms through basal and induced resistance. The in-depth understanding of the molecular basis of FW resistance and the candidate genes identified in the study might contribute to the deployment of either genetic engineering or molecular breeding approaches to develop Fusarium wilt resistant chickpea varieties.

For Review Only

References

Ashraf, N., Basu, S., Narula, K., Ghosh, S., Tayal, R., Gangisetty, N., Biswas, S., Aggarwal, P. R., Chakraborty, N., & Chakraborty, S. (2018) Integrative network analyses of wilt transcriptome in chickpea reveal genotype dependent regulatory hubs in immunity and susceptibility. *Scientific Reports*, **8**, 6528

Bai, Y., Pavan, S., Zheng, Z., Zappel, N. F., Reinstädler, A., Lotti, C., de Giovanni, C., Ricciardi, L., Lindhout, P., Visser, R., Theres, K., & Panstruga, R. (2008) Naturally occurring broad-spectrum powdery mildew resistance in a Central American tomato accession is caused by loss of *Mlo* function. *Molecular Plant-Microbe Interactions*, **21**, 30-39

Bhogireddy, S., Xavier, A., Garg, V., Layland, N., Arias, R., Payton, P., Nayak, S. N., Pandey, M. K., Puppala, N., & Varshney, R. K. (2020) Genome-wide transcriptome and physiological analyses provide new insights into peanut drought response mechanisms. *Scientific Reports*, **10**, 4071

Biswas, K., Tarafdar, A., Kumar, R., Singhvi, N., Ghosh, P., Sharma, M., Pabbi, S., & Shukla, P. (2020) Molecular analysis of disease-responsive genes revealing the resistance potential against fusarium wilt (*Fusarium udum* Butler) dependent on genotype variability in the leguminous crop pigeonpea. *Frontiers in Genetics*, **11**, 862

Bolger, A. M., Lohse, M., & Usadel, B. (2014) Trimmomatic: a flexible trimmer for Illumina sequence data. *Bioinformatics*, **30**, 2114-2120

Boyle, E. I., Weng, S., Gollub, J., Jin, H., Botstein, D., Cherry, J. M., & Sherlock, G. (2004) GO::TermFinder – open source software for accessing Gene Ontology information and finding significantly enriched Gene Ontology terms associated with a list of genes. *Bioinformatics*, **12**, 3710-3715

Büschges, R., Hollricher, K., Panstruga, R., Simons, G., Wolter, M., Frijters, A., van Daelen, R., van der Lee, T., Diergaarde, P., Groenendijk, J., Töpsch, S., Vos, P., Salamini, F., &

For Review Only

Schulze-Lefert, P. (1997) The barley *Mlo* gene: a novel control element of plant pathogen resistance. *Cell*, **88**, 695-705

Chang, Y., Sun, F., Sun, S., Wang, L., Wu, J., & Zhu, Z. (2021) Transcriptome Analysis of Resistance to Fusarium Wilt in Mung Bean (*Vigna radiata* L.). *Frontiers in Plant Science*, **12**, 679629

Conesa, A., Götz, S., García-Gómez, J. M., Terol, J., Talón, M., & Robles, M. (2005) Blast2GO: a universal tool for annotation, visualization and analysis in functional genomics research. *Bioinformatics*, **21**, 3674-3676

Dong, C. J., & Liu, J. Y. (2010) The Arabidopsis EAR-motif-containing protein RAP2.1 functions as an active transcriptional repressor to keep stress responses under tight control. *BMC Plant Biology*, **10**, 47

FAOSTAT (2021) FAOSTAT, FAO (Food and Agriculture Organization of the United Nations). Available online at: https://www.fao.org/faostat.

Garg, T., Mallikarjuna, B. P., Thudi, M., Samineni, S., Singh, S., Sandhu, J. S., Kaur, L., Singh, I., Sirari, A., Basandrai, A. K., Basandrai, D., Varshney, R. K., & Gaur, P. M. (2018) Identification of QTLs for resistance to Fusarium wilt and Ascochyta blight in a recombinant inbred population of chickpea (*Cicer arietinum* L.). *Euphytica*, **214**, 45

Garg, V., Khan, A. W., Kudapa, H., Kale, S. M., Chitikineni, A., Qiwei, S., Sharma, M., Li, C., Zhang, B., Xin, L., Kishor, P. B. K., & Varshney, R. K. (2019) Integrated transcriptome, small RNA and degradome sequencing approaches provide insights into Ascochyta blight resistance in chickpea. *Plant Biotechnology Journal*, **17**, 914-931

Garg, V., Dudchenko, O., Wang, J., Khan, A. W., Gupta, S., Kaur, P., Han, K., Saxena, R. K., Kale, S. M., Pham, M., Yu, J., Chitikineni, A., Zhang, Z., Fan, G., Lui, C., Valluri, V., Meng, F., Bhandari, A., Liu, X., … Varshney, R. K. (2022) Chromosome-length genome assemblies of six legume species provide insights into genome organization, evolution, and agronomic traits for crop improvement. *Journal of Advanced Research*, **42**, 315-329

For Review Only

Gaur, P.M., & Chaturvedi, S. K. (2004) Genetic options for managing biotic stresses in pulse crops. In: *Pulses in new perspective: proceedings of the National Symposium on Crop Diversification and Natural Resource Management*, 20-22 December 2003, Indian Institute of Pulse Research, Kanpur, India

Gupta, S., Dong, Y., Dijkwel, P. P., Mueller-Roeber, B., & Gechev, T. S. (2019) Genome-wide analysis of ROS antioxidant genes in resurrection species suggest an involvement of distinct ROS detoxification systems during desiccation. *International Journal of Molecular Sciences*, **20**, 3101

He, X., Zhu, L., Xu, L., Guo, W., & Zhang, X. (2016) GhATAF1, a NAC transcription factor, confers abiotic and biotic stress responses by regulating phytohormonal signaling networks. *Plant Cell Reports*, **35**, 2167-2179

Henningsen, E. C., Omidvar, V., della Coletta, R., Michno, J.-M., Gilbert, E., Li, F., Miller, M. E., Myers, C. L., Gordon, S. P., Vogel, J. P., Steffenson, B. J., Kianian, S. F., Hirsch, C. D., & Figueroa, M. (2021) Identification of candidate susceptibility genes to *Puccinia graminis* f. sp. *tritici* in Wheat. *Frontiers in Plant Science*, **12**, 657796

Humphry, M., Reinstädler, A., Ivanov, S., Bisseling, T., & Panstruga, R. (2011) Durable broad-spectrum powdery mildew resistance in pea *er1* plants is conferred by natural loss-of-function mutations in *PsMLO1*. *Molecular Plant Pathology*, **12**, 866-878

Kallamadi, P. R., Dandu, K., Kirti, P. B., Rao, C. M., Thakur, S. S., & Mulpuri, S. (2018) An insight into powdery mildew-infected, susceptible, resistant and immune sunflower genotypes. *Proteomics*, **18**, 1700418

Kim, K. H., Kang, Y. J., Kim, D. H., Yoon, M. Y., Moon, J.-K., Kim, M. Y., Van, K., & Lee, S.-H. (2011) RNA-Seq analysis of a soybean near-isogenic line carrying bacterial leaf pustule-resistant and-susceptible alleles. *DNA Research*, **18**, 483-497

For Review Only

Kim, D., Paggi, J. M., Park, C., Bennett, C., & Salzberg, S. L. (2019) Graph-based genome alignment and genotyping with HISAT2 and HISAT-genotype. *Nature Biotechnology*, **37**, 907-915

Kudapa, H., Garg, V., Chitikineni, A., & Varshney, R. K. (2018) The RNA-Seq-based high resolution gene expression atlas of chickpea (*Cicer arietinum* L.) reveals dynamic spatio-temporal changes associated with growth and development. *Plant, Cell & Environment*, **41**, 2209-2225

Kudapa, H., Barmukh, R., Garg, V., Chitikineni, A., Samineni, S., Agarwal, G., & Varshney, R. K. (2023) Comprehensive transcriptome profiling uncovers molecular mechanisms and potential candidate genes associated with heat stress response in chickpea. *International Journal of Molecular Sciences*, **24**, 1369

Li, A., Wang, X., Leseberg, C. H., Jia, J., & Mao, L. (2008) Biotic and abiotic stress responses through calcium-dependent protein kinase (CDPK) signaling in wheat (*Triticum aestivum* L.). *Plant Signaling & Behavior*, **3**, 654-656

Li, N., Zhao, M., Liu, T., Dong, L., Cheng, Q., Wu, J., Wang, L., Chen, X., Zhang, C., Lu, W., Xu, P., & Zhang, S. (2017) A novel soybean dirigent gene GmDIR22 contributes to promotion of lignan biosynthesis and enhances resistance to *Phytophthora sojae*. *Frontiers in Plant Science*, **8**, 1185

Mashaki, K. M., Garg, V., Nasrollahnezhad Ghomi, A. A., Kudapa, H., Chitikineni, A., Zaynali Nezhad, K., Yamchi, A., Soltanloo, H., Varshney, R. K., & Thudi, M. (2018) RNA-Seq analysis revealed genes associated with drought stress response in kabuli chickpea (*Cicer arietinum* L.). *PLoS One*, **13**, e0199774

Moriya, Y., Itoh, M., Okuda, S., Yoshizawa, A. C., & Kanehisa, M. (2007) KAAS: an automatic genome annotation and pathway reconstruction server. *Nucleic Acids Research*, **35**, W182-W185

For Review Only

Nayak, S. N., Agarwal, G., Pandey, M. K., Sudini, H. K., Jayale, A. S., Purohit, S., Desai, A., Wan, L., Guo, B., Liao, B., & Varshney, R. K. (2017) *Aspergillus flavus* infection triggered immune responses and host-pathogen cross-talks in groundnut during *in-vitro* seed colonization. *Scientific Reports*, **7**, 9659

Nuruzzaman, M., Sharoni, A. M., & Kikuchi, S. (2013) Roles of NAC transcription factors in the regulation of biotic and abiotic stress responses in plants. *Frontiers in Microbiology*, **4**, 248

Pande, S., Sharma, M., Nagavardhini, A., & Rameshwar, T. (2012) High Throughput Phenotyping of Chickpea disease: Stepwise identification of host plant resistance. *Information Bulletin No. 92*. Patancheru, AP 502 324, India: International Crops Research Institute for the Semi-Arid Tropics. 56 pp

Park, J. M., Park, C.-J., Lee, S.-B., Ham, B.-K., Shin, R., & Paek, K.-H. (2001) Overexpression of the tobacco Tsi1 gene encoding an EREBP/AP2-type transcription factor enhances resistance against pathogen attack and osmotic stress in tobacco. *The Plant Cell*, **13**, 1035-1046

Patil, B. S., Ravikumar, R. L., Bhat, J. S., & Soregaon, C. D. (2014) Molecular mapping of QTLs for resistance to early and late Fusarium wilt in chickpea. *Czech Journal of Genetics and Plant Breeding*, **50**, 171-176

Sabbavarapu, M. M., Sharma, M., Chamarthi, S. K., Swapna, N., Rathore, A., Thudi, M., Gaur, P. M., Pande, S., Singh, S., Kaur, L., & Varshney, R. K. (2013) Molecular mapping of QTLs for resistance to Fusarium wilt (race 1) and Ascochyta blight in chickpea (*Cicer arietinum* L.). *Euphytica*, **193**, 121-133

Sharma, M., Pande, S., Mangala, U. N., Tripathi, S., & Gaur, P. M. (2010) Genetic resistance in desi and kabuli chickpea lines to Fusarium wilt caused by *Fusarium oxysporum* f. sp. *ciceris*. *Indian Journal of Plant Protection*, **38**, 57-62

For Review Only

Sharma, M., Babu, T. K., Gaur, P. M., Ghosh, R., Rameshwar, T., Chaudhary, R. G., Upadhyay, J. P., Gupta, O., Saxena, D. R., Kaur, L., Dubey, S. C., Anandani, V. P., Harer, P. N., Rathore, A., & Pande, S. (2012) Identification and multi-environment validation of resistance to *Fusarium oxysporum* f. sp. *ciceris* in chickpea. *Field Crops Research*, **135**, 82-88

Sharma, M., Sengupta, A., Ghosh, R., Agarwal, G., Tarafdar, A., Nagavardhini, A., Pande, S., & Varshney, R. K. (2016) Genome wide transcriptome profiling of *Fusarium oxysporum* f sp. *ciceris* conidial germination reveals new insights into infection-related genes. *Scientific Reports*, **6**, 37353

Supek, F., Bošnjak, M., Škunca, N., & Šmuc, T. (2011) REVIGO summarizes and visualizes long lists of Gene Ontology terms. *PLoS One*, **6**, e21800

Tian, F., Yang, D.-C., Meng, Y.-Q., Jin, J., & Gao, G. (2020) PlantRegMap: charting functional regulatory maps in plants. *Nucleic Acids Research*, **48**, D1104-1113

Trapnell, C., Roberts, A., Goff, L., Pertea, G., Kim, D., Kelley, D. R., Pimentel, H., Salzberg, S. L., Rinn, J. L., & Pachter, L. (2012) Differential gene and transcript expression analysis of RNA-seq experiments with TopHat and Cufflinks. *Nature Protocols*, **7**, 562-578

Trapnell, C., Hendrickson, D. G., Sauvageau, M., Goff, L., Rinn, J. L., & Pachter, L. (2013) Differential analysis of gene regulation at transcript resolution with RNA-seq. *Nature Biotechnology*, **31**, 46-53

Upasani, M. L., Limaye, B. M., Gurjar, G. S., Kasibhatla, S. M., Joshi, R. R., Kadoo, N. Y., & Gupta, V. S. (2017) Chickpea-*Fusarium oxysporum* interaction transcriptome reveals differential modulation of plant defense strategies. *Scientific Reports*, **7**, 7746

Vadassery, J., & Oelmüller, R. (2009) Calcium signaling in pathogenic and beneficial plant microbe interactions: what can we learn from the interaction between *Piriformospora indica* and *Arabidopsis thaliana*. *Plant Signaling Behavior*, **4**, 1024-1027

CSSA 5585 Guilford Rd., Madison, WI 53711

van Damme, M., Huibers, R. P., Elberse, J., & van den Ackerveken, G. (2008) Arabidopsis *DMR6* encodes a putative 2OG-Fe(II) oxygenase that is defense-associated but required for susceptibility to downy mildew. *The Plant Journal*, **54**, 785-793

Varshney, R. K., Song, C., Saxena, R. K., Azam, S., Yu, S., Sharpe, A. G., Cannon, S., Baek, J., Rosen, B. D., Tar'an, B., Millan, T., Zhang, X., Ramsay, L. D., Iwata, A., Wang, Y., Nelson, W., Farmer, A. D., Gaur, P. M., Soderlund, C., … Cook, D. R. (2013) Draft genome sequence of chickpea (*Cicer arietinum*) provides a resource for trait improvement. *Nature Biotechnology*, **31**, 240-246

Varshney, R. K., Mohan, S. M., Gaur, P. M., Chamarthi, S. K., Singh, V. K., Srinivasan, S., Swapna, N., Sharma, M., Pande, S., Singh, S., & Kaur, L. (2014) Marker-assisted backcrossing to introgress resistance to Fusarium wilt race 1 and Ascochyta blight in C 214, an elite cultivar of chickpea. *The Plant Genome*, **7**, plantgenome2013.10.0035

Wang, X., Basnayake, B. M. V. S., Zhang, H., Li, G., Li, W., Virk, N., Mengiste, T., & Song, F. (2009) The Arabidopsis ATAF1, a NAC transcription factor, is a negative regulator of defense responses against necrotrophic fungal and bacterial pathogens. *Molecular Plant-Microbe Interactions*, **22**, 1227-1238

Wang, Y., Cheng, X., Shan, Q., Zhang, Y., Liu, J., Gao, C., & Qiu, J.-L. (2014) Simultaneous editing of three homoeoalleles in hexaploid bread wheat confers heritable resistance to powdery mildew. *Nature Biotechnology*, **32**, 947-951

Wickham, H. (2016) Ggplot2: Elegant Graphics for Data Analysis, 2nd ed.; Springer International Publishing: New York, NY, USA

Young, M. D., Wakefield, M. J., Smyth, G. K., & Oshlack, A. (2010) Gene ontology analysis for RNA-seq: accounting for selection bias. *Genome Biology*, **11**, R14

Zhang, W., Sun, P., He, Q., Shu, F., & Deng, H. (2018) Transcriptome analysis of near-isogenic line provides novel insights into genes associated with panicle traits regulation in rice. *PLoS One*, **13**, e0199077

For Review Only

Supplemental Material

Six tables are provided as supplemental material to support the results presented in this study.

Data Availability

The RNA-sequencing data generated in the study are available in NCBI Sequence Read Archive (SRA, http://www.ncbi.nlm.nih.gov/sra) with the BioProject ID PRJNA761666.

Acknowledgments

Authors are thankful to Food Futures Institute, Murdoch University (Australia), for financial support. HB is thankful to the SERB of DST, Government of India, for providing the SERB Women Excellence Award (SB/WEA-01/2017). This work was supported by resources provided by the Pawsey Supercomputing Research Centre with funding from the Australian Government and the Government of Western Australia.

For Review Only

Conflict of interest

The authors declare that they have no competing interests.

Author contributions

RKV and HK conceived and designed the experiment. VG analysed the transcriptome data and wrote the manuscript. AC, SS and RKV contributed to the reagents, materials and generation of sequencing data. MS and RG conducted the phenotypic evaluation. All authors read, revised and approved the final manuscript.

Table 1. Statistics of transcriptome sequencing data generated for 24 samples obtained from six different chickpea genotypes.

Sample	Raw reads	Filtered Reads	Filtered reads (%)	Mapped reads	Mapped reads (%)
C 214-C-7d	42,855,890	42,018,711	98.05	41,053,642	97.70%
C 214-S-7d	68,300,372	67,301,196	98.54	63,588,867	94.48%
C 214-C-12d	45,760,416	44,851,746	98.01	43,952,707	98.00%
C 214-S-12d	45,318,494	44,171,702	97.47	41,826,372	94.69%
ICCV 05528-C-7d	41,176,344	40,161,473	97.54	38,679,310	96.31%
ICCV 05528-S-7d	76,444,046	75,200,992	98.37	69,633,871	92.60%
ICCV 05528-C-12d	44,135,388	43,245,710	97.98	42,451,202	98.16%
ICCV 05528-S-12d	45,424,154	44,108,667	97.10	41,230,585	93.48%
JG 62-C-7d	43,164,592	42,061,605	97.44	41,105,924	97.73%
JG 62-S-7d	39,953,776	38,897,082	97.36	35,536,177	91.36%
JG 62-C-12d	42,873,788	41,702,173	97.27	40,712,386	97.63%
JG 62-S-12d	48,771,794	47,281,335	96.94	44,930,450	95.03%
NIL 01-C-7d	46,350,560	45,178,442	97.47	43,648,003	96.61%
NIL 01-S-7d	53,447,742	52,473,278	98.18	49,810,856	94.93%
NIL 01-C-12d	39,440,102	38,178,489	96.80	37,133,220	97.26%
NIL 01-S-12d	44,696,102	43,461,280	97.24	40,634,452	93.50%
NIL 02-C-7d	45,465,094	44,397,549	97.65	42,286,280	95.24%
NIL 02-S-7d	64,248,978	63,117,485	98.24	56,887,070	90.13%
NIL 02-C-12d	43,588,388	42,633,020	97.81	41,748,412	97.93%
NIL 02-S-12d	40,373,906	39,037,532	96.69	36,847,374	94.39%
WR 315-C-7d	40,835,890	39,940,681	97.81	39,043,810	97.75%
WR 315-S-7d	41,035,634	39,936,236	97.32	36,482,837	91.35%
WR 315-C-12d	48,526,488	47,125,222	97.11	45,662,475	96.90%
WR 315-S-12d	44,507,508	43,323,914	97.34	40,594,183	93.70%

For Review Only

Table 2. Distribution of expressed genes identified from different chickpea genotypes.

Sample ID	No. of expressed genes (FPKM >= 1)	No. of low/moderately expressed genes (FPKM >= 1 & < 10)	No. of highly expressed genes (FPKM >= 10)
C 214-C-7d	18,651	8107	10,544
C 214-S-7d	18,411	7970	10,441
C 214-C-12d	18,753	8260	10,493
C 214-S-12d	18,237	7902	10,335
ICCV 05528-C-7d	18,531	8007	10,524
ICCV 05528-S-7d	18,450	8106	10,344
ICCV 05528-C-12d	18,872	8390	10,482
ICCV 05528-S-12d	18,165	7908	10,257
JG 62-C-7d	18,847	8293	10,554
JG 62-S-7d	18,141	7650	10,491
JG 62-C-12d	18,251	7810	10,441
JG 62-S-12d	18,141	7563	10,578
NIL 01-C-7d	18,602	8107	10,495
NIL 01-S-7d	18,420	8142	10,278
NIL 01-C-12d	18,365	7694	10,671
NIL 01-S-12d	18,381	8023	10,358
NIL 02-C-7d	18,439	7944	10,495
NIL 02-S-7d	18,501	8129	10,372
NIL 02-C-12d	18,624	8090	10,534
NIL 02-S-12d	18,572	8005	10,567
WR 315-C-7d	18,847	8279	10,568
WR 315-S-7d	18,111	7671	10,440
WR 315-C-12d	18,674	8093	10,581
WR 315-S-12d	18,041	7637	10,404

For Review Only

Figure Legends

Figure 1. Statistics of differentially expressed genes (DEGs) identified between different combinations of chickpea genotypes in response to *F. oxysporum* stress. (a) The number of DEGs in each chickpea genotype (C 214, JG 62, WR 315, ICCV 05528, NIL 01, and NIL 02) at 7th and 12th-day post-inoculation (dpi) under control and *F. oxysporum* inoculated conditions are presented in the bar graph. The number of up- and down-regulated genes are shown in the form of bars above and below the *x*-axis, respectively; **(b)** Number of DEGs between different genotypes at both 7th and 12th dpi under control conditions; **(c)** Number of DEGs between different genotypes at both 7th and 12th dpi under stress conditions. UpSet plots depicting overlapping and specific response of DEGs within different chickpea genotypes under control vs stress conditions at **(d)** 7th dpi and **(e)** 12th dpi. The number of genes showing specific and overlapping response is mentioned on top of each bar. The horizontal bars represent the number of DEGs per genotype. The black circles below the vertical bars denote the genotype(s) and vertical bars represent the number of DEGs overlapping or unique to the respective genotypes.

Figure 2. A circular plot representing genome-wide distribution of Fusarium wilt (FW) resistance QTLs, differentially expressed genes (DEGs), transcription factors (TFs) and expression of genes at 7th and 12th day post-inoculation (dpi). Six different tracks (out to in) of the circular plot shows following: (a) FW resistance QTLs; (b) DEGs; (c) Differentially expressed TFs; (d) Expression of genes (log_2 FPKM) in different genotypes at 7th dpi (moving from out to in; C 214, JG 62, NIL 01, NIL 02, ICCV 05528 and WR 315); (e) Expression of genes (log_2 FPKM) in different genotypes at 12th dpi (moving from out to in; C 214, JG 62, NIL 01, NIL 02, ICCV 05528 and WR 315).

Figure 3. Annotation and pathway analysis of the differentially expressed genes (DEGs). (a) Gene ontology (GO) enrichment analysis of DEGs identified between different chickpea

For Review Only

genotypes under control and stress conditions. GO terms in the biological process category are summarised and visualized using REVIGO. GO terms are represented by circles where the size of the circle is proportional to the frequency of the GO term, and the color indicates the log (*P*-value). **(b)** Scatter plot of the significantly enriched KEGG pathways (*P*-value <0.05) during FW stress conditions. GeneRatio is the ratio of the number of DEGs in the pathway, and the total number of DEGs with pathway annotation in the scatter plot. **(c)** Pie chart showing the distribution of differentially expressed transcription factors in response to FW stress.

Figure 4. Response common to both resistant and susceptible genotypes under stress conditions. The heatmap shows the expression profiles of differentially expressed genes (DEGs) under FW stress conditions at 7th and 12th dpi in each chickpea genotypes (C 214, JG 62, NIL 01, NIL 02, ICCV 05528 and WR 315). The genes showing similar expression trends in all genotypes upon *F. oxysporum* infection are shown. The color scale at the top shows $\log_2$ fold change. In the heat map, blue signifies up-regulation/induced expression and red signifies down-regulation/repressed expression. The asterisk (*) shows that the gene is present in one of the previously reported Fusarium wilt (FW) QTLs.

Figure 5. Response specific to resistant and susceptible chickpea genotypes under stress conditions. The genes with similar expression patterns have been grouped together into four clusters and selected representative genes from each cluster are shown in the heatmap. Clusters I to IV represents DEGs identified between resistant (including WR 315, ICCV 05528, NIL 01, and NIL 02) and susceptible genotypes (C 214 and JG 62) upon *F*oc infection at 7th and 12th dpi. The color scale at the top shows $\log_2$ fold change. In the heat map, blue signifies up-regulation/induced expression and red signifies down-regulation/repressed expression in resistant genotypes. The asterisk (*) shows that the gene is present in one of the previously reported Fusarium wilt (FW) QTLs.

For Review Only

Supplemental Tables

Supplemental Table S1. Statistics of reads mapped on different genomic regions of the chickpea reference genome.

Supplemental Table S2. List of differentially expressed genes, their chromosomal localization, and functional annotation.

Supplemental Table S3. Functional annotation (Biological Process Category) of DEGs identified from different chickpea genotypes under control and stress conditions.

Supplemental Table S4. Functional annotation (Cellular Component Category) of DEGs identified from different chickpea genotypes under control and stress conditions.

Supplemental Table S5. Functional annotation (Molecular Function Category) of DEGs identified from different chickpea genotypes under control and stress conditions.

Supplemental Table S6. List of differentially expressed transcription factor encoding genes.

For Review Only

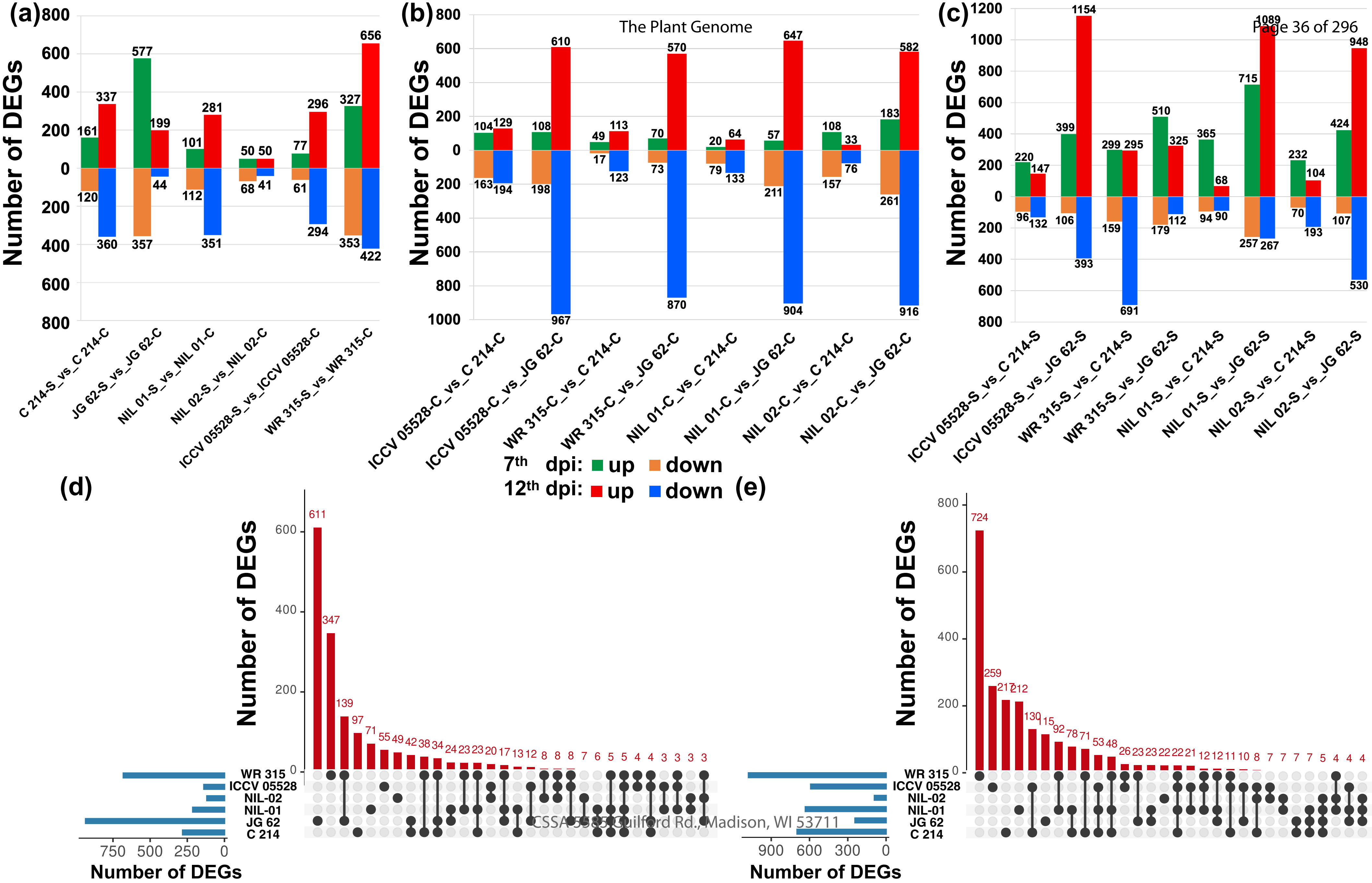
(a)
(b)
(c)
(d)
(e)
Number of DEGs
C 214-S_vs_C 214-C
JG 62-S_vs_JG 62-C
NIL 01-S_vs_NIL 01-C
NIL 02-S_vs_NIL 02-C
ICCV 05528-S_vs_ICCV 05528-C
WR 315-S_vs_WR 315-C
ICCV 05528-C_vs_C 214-C
ICCV 05528-C_vs_JG 62-C
WR 315-C_vs_C 214-C
WR 315-C_vs_JG 62-C
NIL 01-C_vs_C 214-C
NIL 01-C_vs_JG 62-C
NIL 02-C_vs_C 214-C
NIL 02-C_vs_JG 62-C
ICCV 05528-S_vs_C 214-S
ICCV 05528-S_vs_JG 62-S
WR 315-S_vs_C 214-S
WR 315-S_vs_JG 62-S
NIL 01-S_vs_C 214-S
NIL 01-S_vs_JG 62-S
NIL 02-S_vs_C 214-S
NIL 02-S_vs_JG 62-S
7th dpi: up down
12th dpi: up down
WR 315
ICCV 05528
NIL-02
NIL-01
JG 62
C 214

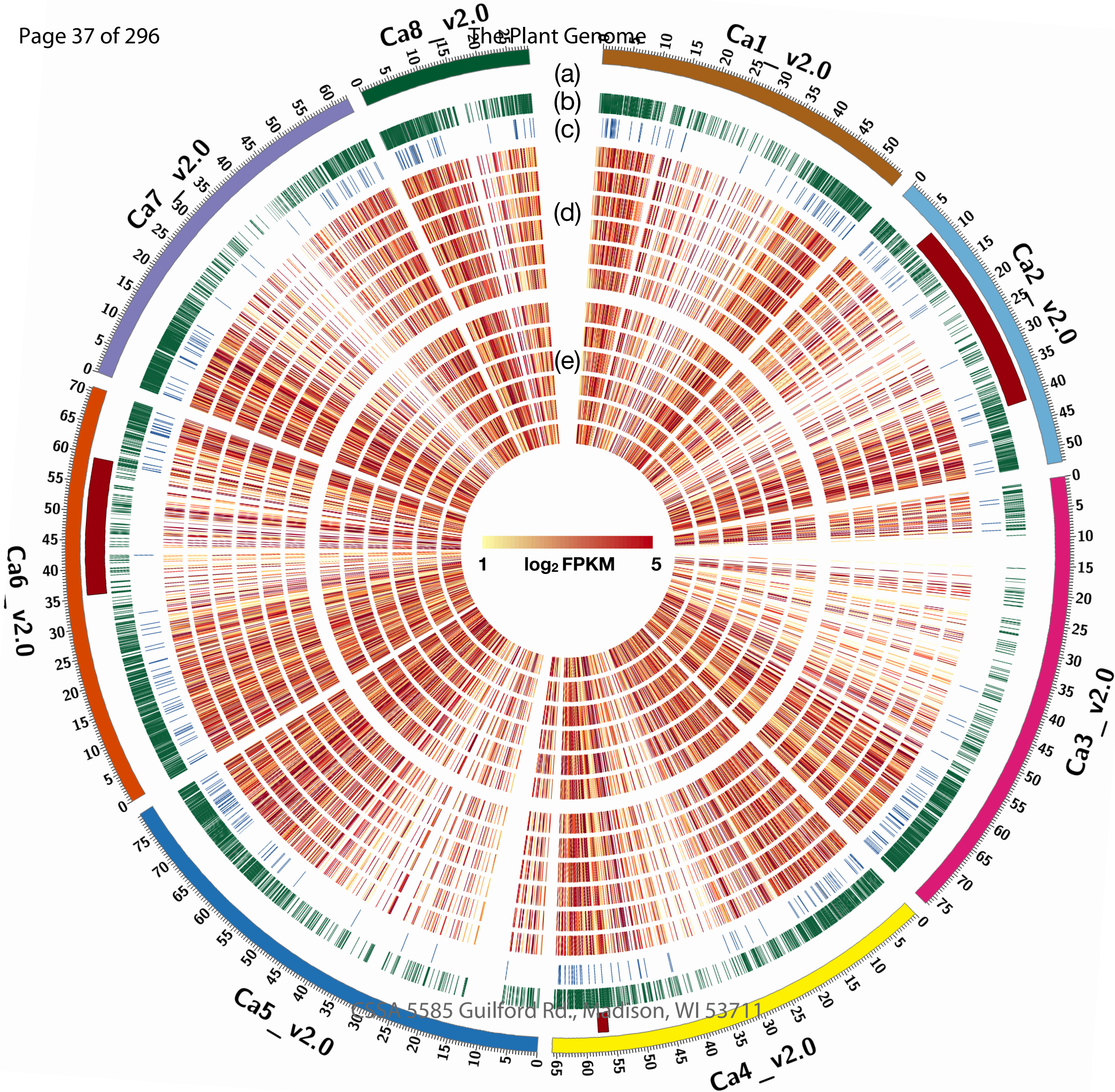

Ca1_v2.0
Ca2_v2.0
Ca3_v2.0
Ca4_v2.0
Ca5_v2.0
Ca6_v2.0
Ca7_v2.0
Ca8_v2.0
(a)
(b)
(c)
(d)
(e)
1
log2 FPKM
5

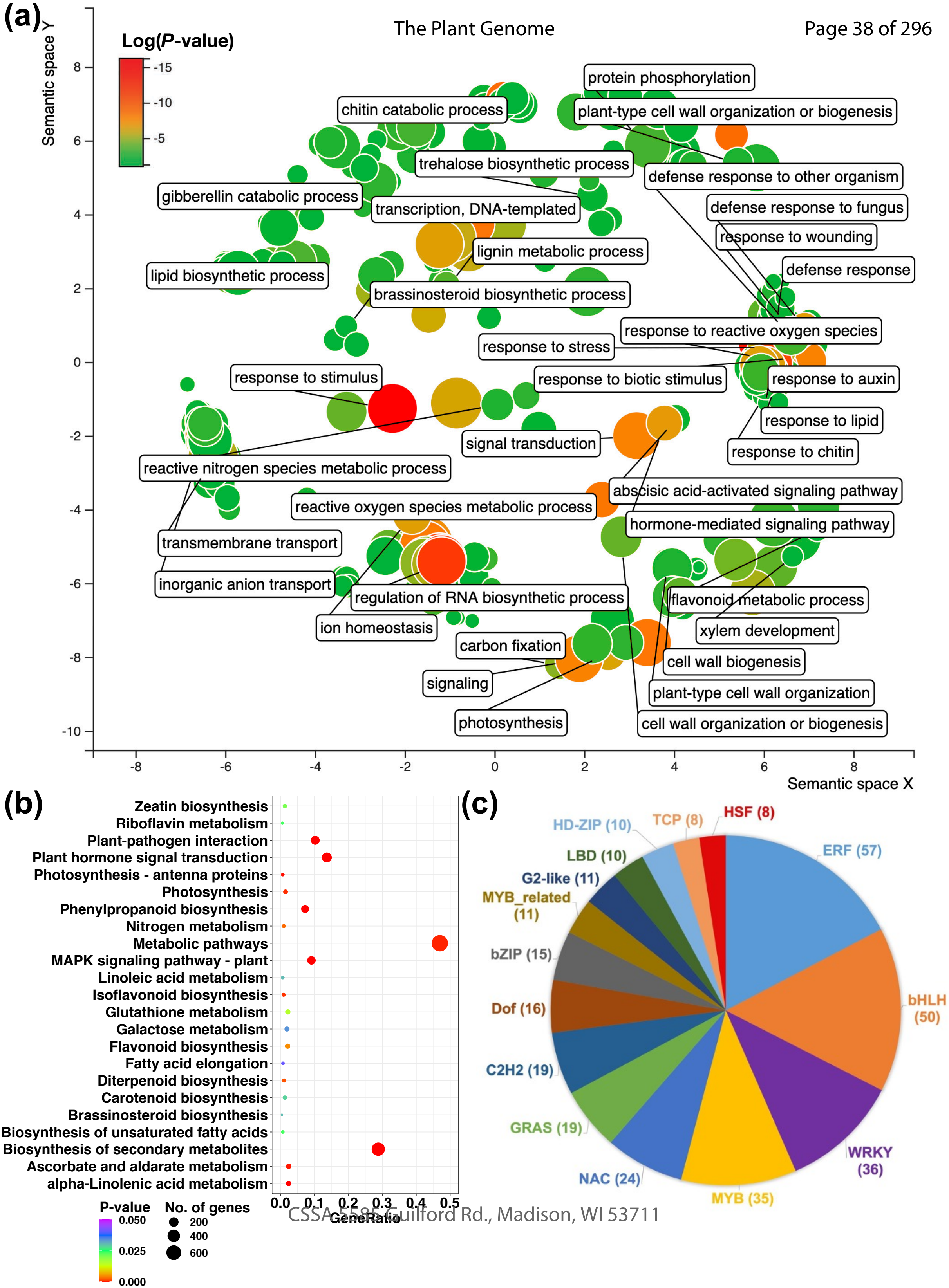
(a)
Log(P-value)
-15
-10
-5
Semantic space Y
Semantic space X
protein phosphorylation
plant-type cell wall organization or biogenesis
chitin catabolic process
trehalose biosynthetic process
defense response to other organism
gibberellin catabolic process
transcription, DNA-templated
defense response to fungus
response to wounding
lignin metabolic process
defense response
lipid biosynthetic process
brassinosteroid biosynthetic process
response to reactive oxygen species
response to stress
response to stimulus
response to biotic stimulus
response to auxin
response to lipid
signal transduction
response to chitin
reactive nitrogen species metabolic process
abscisic acid-activated signaling pathway
reactive oxygen species metabolic process
hormone-mediated signaling pathway
transmembrane transport
inorganic anion transport
regulation of RNA biosynthetic process
flavonoid metabolic process
ion homeostasis
xylem development
carbon fixation
cell wall biogenesis
signaling
plant-type cell wall organization
photosynthesis
cell wall organization or biogenesis
(b)
Zeatin biosynthesis
Riboflavin metabolism
Plant-pathogen interaction
Plant hormone signal transduction
Photosynthesis - antenna proteins
Photosynthesis
Phenylpropanoid biosynthesis
Nitrogen metabolism
Metabolic pathways
MAPK signaling pathway - plant
Linoleic acid metabolism
Isoflavonoid biosynthesis
Glutathione metabolism
Galactose metabolism
Flavonoid biosynthesis
Fatty acid elongation
Diterpenoid biosynthesis
Carotenoid biosynthesis
Brassinosteroid biosynthesis
Biosynthesis of unsaturated fatty acids
Biosynthesis of secondary metabolites
Ascorbate and aldarate metabolism
alpha-Linolenic acid metabolism
GeneRatio
P-value
0.050
0.025
0.000
No. of genes
200
400
600
(c)
HD-ZIP (10)
TCP (8)
HSF (8)
ERF (57)
LBD (10)
G2-like (11)
MYB_related (11)
bZIP (15)
bHLH (50)
Dof (16)
C2H2 (19)
GRAS (19)
WRKY (36)
NAC (24)
MYB (35)

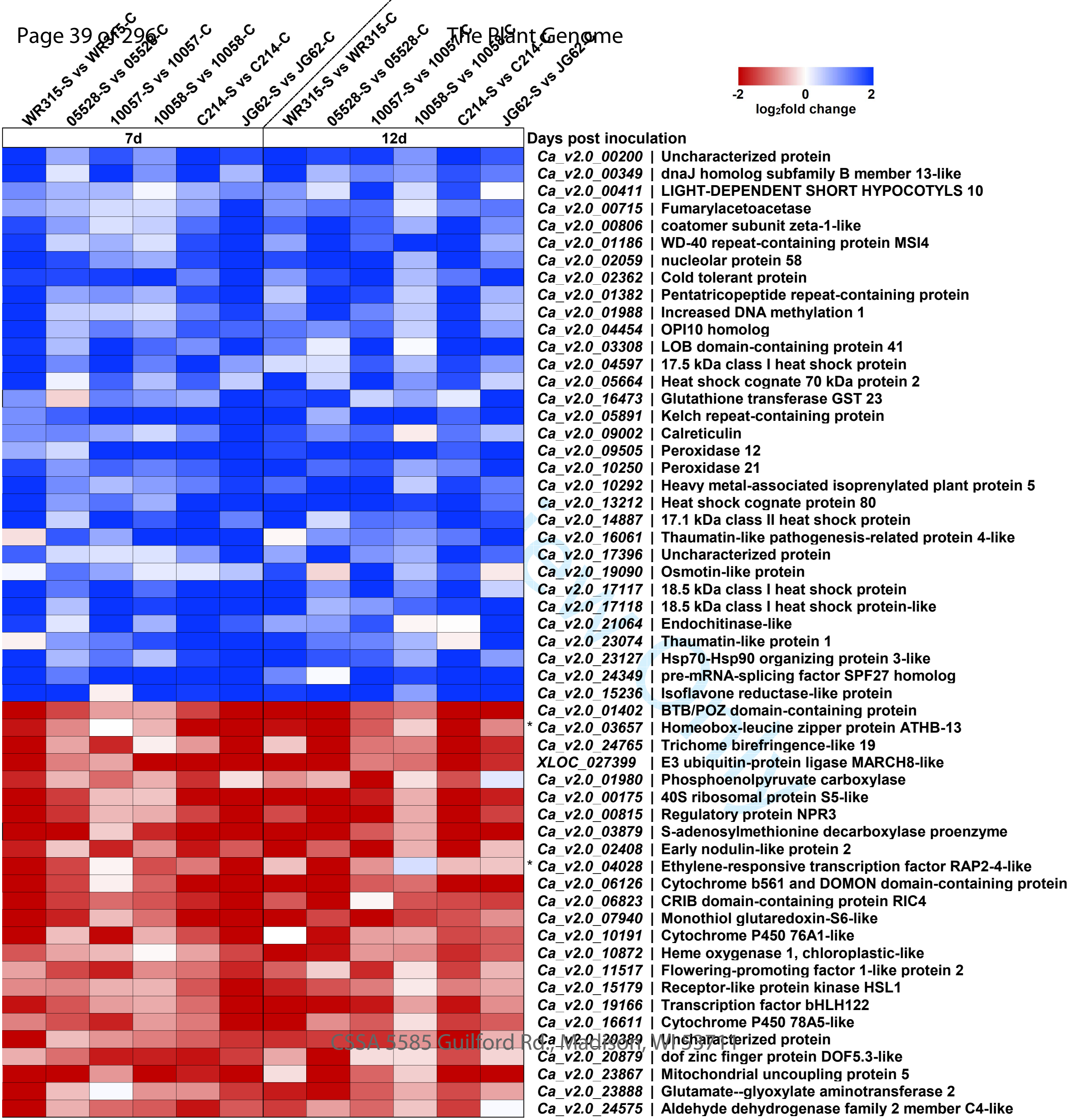
WR315-S vs WR315-C
05528-S vs 05528-C
10057-S vs 10057-C
10058-S vs 10058-C
C214-S vs C214-C
JG62-S vs JG62-C
WR315-S vs WR315-C
05528-S vs 05528-C
10057-S vs 10057-C
10058-S vs 10058-C
C214-S vs C214-C
JG62-S vs JG62-C
-2
0
2
log₂fold change
7d
12d
Days post inoculation
Ca_v2.0_00200 | Uncharacterized protein
Ca_v2.0_00349 | dnaJ homolog subfamily B member 13-like
Ca_v2.0_00411 | LIGHT-DEPENDENT SHORT HYPOCOTYLS 10
Ca_v2.0_00715 | Fumarylacetoacetase
Ca_v2.0_00806 | coatomer subunit zeta-1-like
Ca_v2.0_01186 | WD-40 repeat-containing protein MSI4
Ca_v2.0_02059 | nucleolar protein 58
Ca_v2.0_02362 | Cold tolerant protein
Ca_v2.0_01382 | Pentatricopeptide repeat-containing protein
Ca_v2.0_01988 | Increased DNA methylation 1
Ca_v2.0_04454 | OPI10 homolog
Ca_v2.0_03308 | LOB domain-containing protein 41
Ca_v2.0_04597 | 17.5 kDa class I heat shock protein
Ca_v2.0_05664 | Heat shock cognate 70 kDa protein 2
Ca_v2.0_16473 | Glutathione transferase GST 23
Ca_v2.0_05891 | Kelch repeat-containing protein
Ca_v2.0_09002 | Calreticulin
Ca_v2.0_09505 | Peroxidase 12
Ca_v2.0_10250 | Peroxidase 21
Ca_v2.0_10292 | Heavy metal-associated isoprenylated plant protein 5
Ca_v2.0_13212 | Heat shock cognate protein 80
Ca_v2.0_14887 | 17.1 kDa class II heat shock protein
Ca_v2.0_16061 | Thaumatin-like pathogenesis-related protein 4-like
Ca_v2.0_17396 | Uncharacterized protein
Ca_v2.0_19090 | Osmotin-like protein
Ca_v2.0_17117 | 18.5 kDa class I heat shock protein
Ca_v2.0_17118 | 18.5 kDa class I heat shock protein-like
Ca_v2.0_21064 | Endochitinase-like
Ca_v2.0_23074 | Thaumatin-like protein 1
Ca_v2.0_23127 | Hsp70-Hsp90 organizing protein 3-like
Ca_v2.0_24349 | pre-mRNA-splicing factor SPF27 homolog
Ca_v2.0_15236 | Isoflavone reductase-like protein
Ca_v2.0_01402 | BTB/POZ domain-containing protein
* Ca_v2.0_03657 | Homeobox-leucine zipper protein ATHB-13
Ca_v2.0_24765 | Trichome birefringence-like 19
XLOC_027399 | E3 ubiquitin-protein ligase MARCH8-like
Ca_v2.0_01980 | Phosphoenolpyruvate carboxylase
Ca_v2.0_00175 | 40S ribosomal protein S5-like
Ca_v2.0_00815 | Regulatory protein NPR3
Ca_v2.0_03879 | S-adenosylmethionine decarboxylase proenzyme
Ca_v2.0_02408 | Early nodulin-like protein 2
* Ca_v2.0_04028 | Ethylene-responsive transcription factor RAP2-4-like
Ca_v2.0_06126 | Cytochrome b561 and DOMON domain-containing protein
Ca_v2.0_06823 | CRIB domain-containing protein RIC4
Ca_v2.0_07940 | Monothiol glutaredoxin-S6-like
Ca_v2.0_10191 | Cytochrome P450 76A1-like
Ca_v2.0_10872 | Heme oxygenase 1, chloroplastic-like
Ca_v2.0_11517 | Flowering-promoting factor 1-like protein 2
Ca_v2.0_15179 | Receptor-like protein kinase HSL1
Ca_v2.0_19166 | Transcription factor bHLH122
Ca_v2.0_16611 | Cytochrome P450 78A5-like
Ca_v2.0_20389 | Uncharacterized protein
Ca_v2.0_20879 | dof zinc finger protein DOF5.3-like
Ca_v2.0_23867 | Mitochondrial uncoupling protein 5
Ca_v2.0_23888 | Glutamate--glyoxylate aminotransferase 2
Ca_v2.0_24575 | Aldehyde dehydrogenase family 2 member C4-like

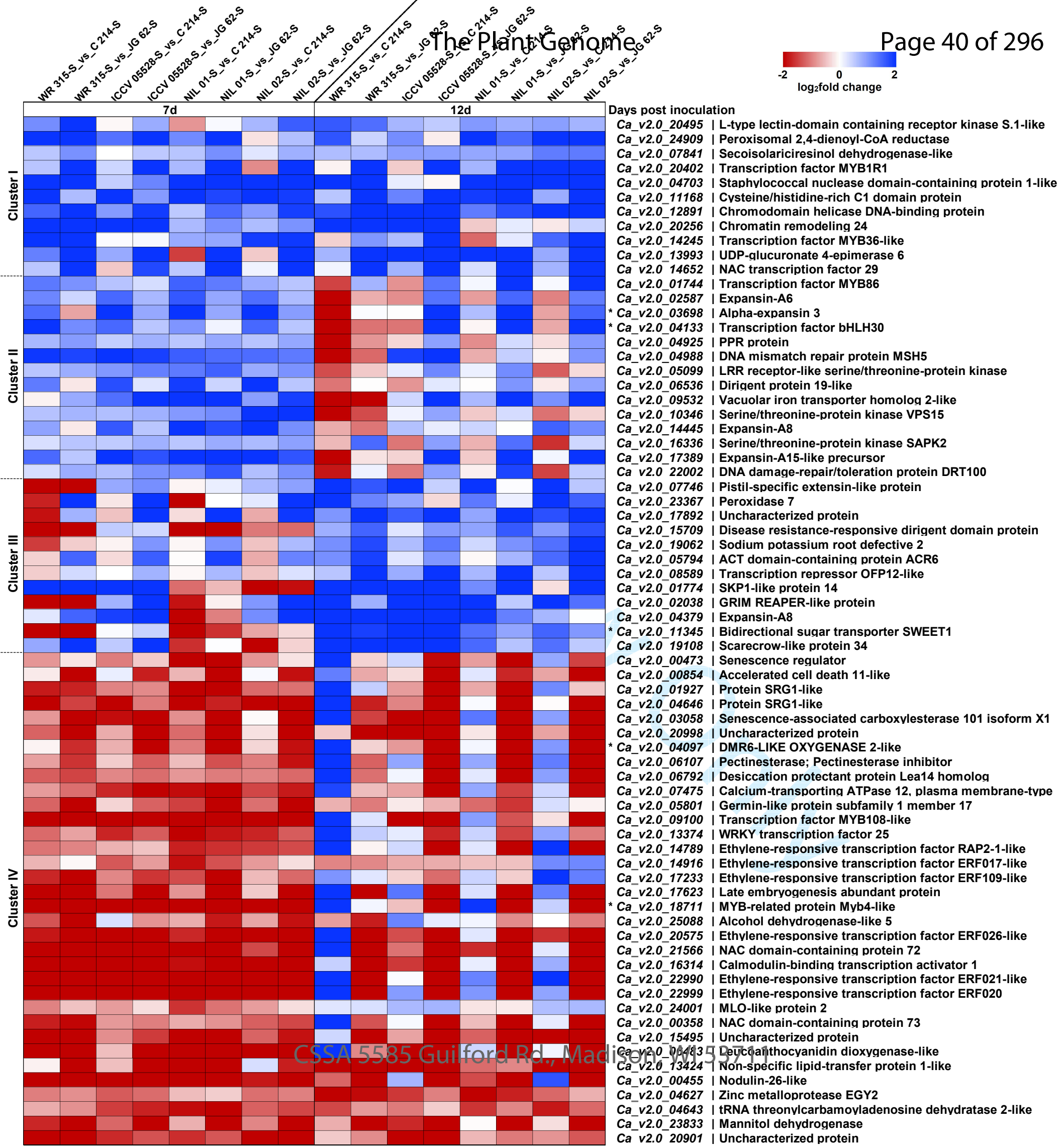

CSSA 5585 Guilford Rd., Madison, WI 53711

Supplemental Table S1. Statistics of reads mapped on different genomic regions of the chickpea refere

Sample	Total mapped reads	Number of reads mapped on exonic regions	Percentage of reads mapped on exonic regions	Number of reads mapped on intronic regions	Percentage of reads mapped on intronic regions
ICCV 05528-Control_12days	42451202	41067894	96.74%	398947	0.94%
ICCV 05528-Control_7days	38679310	37592476	97.19%	321152	0.83%
ICCV 05528-Stress_12days	41230585	39746619	96.40%	324041	0.79%
ICCV 05528-Stress_7days	69633871	60398095	86.74%	542304	0.78%
NIL 01-Control_12days	37133220	36274050	97.69%	285578	0.77%
NIL 01-Control_7days	43648003	42341844	97.01%	346785	0.79%
NIL 01-Stress_12days	40634452	19238828	47.35%	219518	0.54%
NIL 01-Stress_7days	49810856	43807946	87.95%	354177	0.71%
NIL 02-Control_12days	41748412	38217963	91.54%	350369	0.84%
NIL 02-Control_7days	42286280	40991454	96.94%	356301	0.84%
NIL 02-Stress_12days	36847374	36016742	97.75%	266927	0.72%
NIL 02-Stress_7days	56887070	54980053	96.65%	460833	0.81%
C 214-Control_12days	43952707	42942476	97.70%	453889	1.03%
C 214-Control_7days	41053642	40174638	97.86%	405652	0.99%
C 214-Stress_12days	41826372	40564889	96.98%	328445	0.79%
C 214-Stress_7days	63588867	61761833	97.13%	566549	0.89%
JG 62-Control_12days	40712386	39729021	97.58%	302044	0.74%
JG 62-Control_7days	41105924	40181258	97.75%	443691	1.08%
JG 62-Stress_12days	44930450	43100897	95.93%	340855	0.76%
JG 62-Stress_7days	35536177	34033401	95.77%	292028	0.82%
WR 315-Control_12days	45662475	41271947	90.38%	346025	0.76%
WR 315-Control_7days	39043810	38160373	97.74%	353060	0.90%
WR 315-Stress_12days	40594183	39433055	97.14%	357498	0.88%
WR 315-Stress_7days	36482837	31612235	86.65%	340760	0.93%
**Average**	-	-	93.27%	-	0.83%

For Review Only

ence genome.

Number of reads mapped on intergenic regions	Percentage of reads mapped on intergenic regions
984361	2.32%
765682	1.98%
1159925	2.81%
8693472	12.48%
573592	1.54%
959374	2.20%
21176106	52.11%
5648733	11.34%
3180080	7.62%
938525	2.22%
563705	1.53%
1446184	2.54%
556342	1.27%
473352	1.15%
933038	2.23%
1260485	1.98%
681321	1.67%
480975	1.17%
1488698	3.31%
1210748	3.41%
4044503	8.86%
530377	1.36%
803630	1.98%
4529842	12.42%
-	5.90%

For Review Only

Supplemental Table S2. List of differentially expressed genes, their chrc annotation.

XLOC ID	Ca_ID	Chromosome	Start	End
XLOC_000002	-	Ca1_v2.0	40080	49370
XLOC_000031	Ca_v2.0_00057	Ca1_v2.0	446227	465753
XLOC_000032	-	Ca1_v2.0	466563	470703
XLOC_000033	Ca_v2.0_00059	Ca1_v2.0	483097	486665
XLOC_000047	Ca_v2.0_00084	Ca1_v2.0	675500	679570
XLOC_000062	Ca_v2.0_00105	Ca1_v2.0	857441	858314
XLOC_000068	Ca_v2.0_00117	Ca1_v2.0	908072	915930
XLOC_000071	Ca_v2.0_00122	Ca1_v2.0	966295	972226
XLOC_000073	Ca_v2.0_00127	Ca1_v2.0	996631	1003275
XLOC_000080	Ca_v2.0_00139	Ca1_v2.0	1074230	1080631
XLOC_000081	Ca_v2.0_00144	Ca1_v2.0	1103712	1106257
XLOC_000085	Ca_v2.0_00150	Ca1_v2.0	1146572	1149293
XLOC_000086	Ca_v2.0_00151	Ca1_v2.0	1151316	1153532
XLOC_000087	Ca_v2.0_00152	Ca1_v2.0	1159738	1160735
XLOC_000092	Ca_v2.0_00164	Ca1_v2.0	1243555	1244222
XLOC_000100	Ca_v2.0_00182,Ca_v2.0_001	Ca1_v2.0	1373771	1397537
XLOC_000105	Ca_v2.0_00193	Ca1_v2.0	1465431	1470918
XLOC_000109	Ca_v2.0_00200	Ca1_v2.0	1530073	1548188
XLOC_000110	Ca_v2.0_00199	Ca1_v2.0	1530073	1548188
XLOC_000111	Ca_v2.0_00201	Ca1_v2.0	1552276	1556293
XLOC_000115	Ca_v2.0_00208	Ca1_v2.0	1592951	1593531
XLOC_000116	Ca_v2.0_00209	Ca1_v2.0	1604863	1605651
XLOC_000120	Ca_v2.0_00213	Ca1_v2.0	1642678	1645616
XLOC_000122	Ca_v2.0_00218	Ca1_v2.0	1670783	1672950
XLOC_000123	Ca_v2.0_00219	Ca1_v2.0	1674858	1678987
XLOC_000127	Ca_v2.0_00225	Ca1_v2.0	1713216	1716485
XLOC_000130	Ca_v2.0_00236	Ca1_v2.0	1777787	1778423
XLOC_000135	Ca_v2.0_00242,Ca_v2.0_002	Ca1_v2.0	1811848	1819411
XLOC_000137	Ca_v2.0_00248	Ca1_v2.0	1863740	1865557
XLOC_000139	Ca_v2.0_00256	Ca1_v2.0	1921125	1927808
XLOC_000145	Ca_v2.0_00264	Ca1_v2.0	1987984	1990672
XLOC_000147	Ca_v2.0_00269	Ca1_v2.0	2011418	2016136
XLOC_000149	Ca_v2.0_00271	Ca1_v2.0	2022650	2023726
XLOC_000150	Ca_v2.0_00273	Ca1_v2.0	2041458	2076179
XLOC_000156	Ca_v2.0_00288	Ca1_v2.0	2209352	2214325
XLOC_000161	Ca_v2.0_00298	Ca1_v2.0	2283050	2285477
XLOC_000163	Ca_v2.0_00301	Ca1_v2.0	2312541	2316551
XLOC_000170	Ca_v2.0_00311	Ca1_v2.0	2365001	2366243
XLOC_000171	Ca_v2.0_00312	Ca1_v2.0	2366388	2367350
XLOC_000173	Ca_v2.0_00316,Ca_v2.0_003	Ca1_v2.0	2388316	2395790
XLOC_000178	Ca_v2.0_00327	Ca1_v2.0	2509889	2514880
XLOC_000182	Ca_v2.0_00332	Ca1_v2.0	2556322	2557609

For Review Only

XLOC_000183	Ca_v2.0_00334	Ca1_v2.0	2602839	2605250
XLOC_000187	Ca_v2.0_00342	Ca1_v2.0	2684665	2686568
XLOC_000188	Ca_v2.0_00349	Ca1_v2.0	2729468	2732132
XLOC_000193	Ca_v2.0_00358	Ca1_v2.0	2800796	2804133
XLOC_000201	Ca_v2.0_00371	Ca1_v2.0	2896860	2904624
XLOC_000203	Ca_v2.0_00376	Ca1_v2.0	2948445	2949313
XLOC_000208	Ca_v2.0_00384	Ca1_v2.0	3025999	3033939
XLOC_000217	Ca_v2.0_00401	Ca1_v2.0	3140274	3142041
XLOC_000221	Ca_v2.0_00408	Ca1_v2.0	3194787	3195647
XLOC_000222	Ca_v2.0_00411	Ca1_v2.0	3224405	3229912
XLOC_000238	Ca_v2.0_00441	Ca1_v2.0	3455052	3456920
XLOC_000239	Ca_v2.0_00443	Ca1_v2.0	3470312	3471132
XLOC_000242	Ca_v2.0_00446	Ca1_v2.0	3501613	3504282
XLOC_000245	Ca_v2.0_00453	Ca1_v2.0	3564238	3566183
XLOC_000247	Ca_v2.0_00455	Ca1_v2.0	3575110	3577343
XLOC_000250	Ca_v2.0_00463	Ca1_v2.0	3589948	3600597
XLOC_000251	Ca_v2.0_00464	Ca1_v2.0	3602790	3612219
XLOC_000253	Ca_v2.0_00466	Ca1_v2.0	3617499	3624570
XLOC_000256	Ca_v2.0_00473	Ca1_v2.0	3719224	3720047
XLOC_000261	Ca_v2.0_00483	Ca1_v2.0	3832352	3837061
XLOC_000263	Ca_v2.0_00485	Ca1_v2.0	3843959	3845952
XLOC_000266	Ca_v2.0_00491	Ca1_v2.0	3901147	3908947
XLOC_000273	Ca_v2.0_00502	Ca1_v2.0	3998413	4004445
XLOC_000279	Ca_v2.0_00516	Ca1_v2.0	4123834	4124384
XLOC_000307	Ca_v2.0_00567	Ca1_v2.0	4670874	4675828
XLOC_000308	Ca_v2.0_00568	Ca1_v2.0	4676818	4682488
XLOC_000310	-	Ca1_v2.0	4707847	4708936
XLOC_000311	Ca_v2.0_00574	Ca1_v2.0	4760880	4762924
XLOC_000312	Ca_v2.0_00575	Ca1_v2.0	4766790	4767883
XLOC_000325	Ca_v2.0_00604	Ca1_v2.0	5005901	5007326
XLOC_000330	Ca_v2.0_00613	Ca1_v2.0	5093145	5097455
XLOC_000334	Ca_v2.0_00629	Ca1_v2.0	5219295	5226666
XLOC_000335	Ca_v2.0_00630	Ca1_v2.0	5227944	5229494
XLOC_000338	Ca_v2.0_00634,Ca_v2.0_006	Ca1_v2.0	5250189	5432383
XLOC_000342	Ca_v2.0_00643,Ca_v2.0_006	Ca1_v2.0	5250189	5432383
XLOC_000363	Ca_v2.0_00675	Ca1_v2.0	5637389	5639037
XLOC_000364	Ca_v2.0_00676	Ca1_v2.0	5640017	5642566
XLOC_000370	Ca_v2.0_00691	Ca1_v2.0	5745786	5751561
XLOC_000373	Ca_v2.0_00700	Ca1_v2.0	5848491	5850259
XLOC_000375	Ca_v2.0_00704	Ca1_v2.0	5917717	5927837
XLOC_000376	Ca_v2.0_00705	Ca1_v2.0	5930799	5935646
XLOC_000380	Ca_v2.0_00715	Ca1_v2.0	6044970	6050446
XLOC_000381	Ca_v2.0_00716	Ca1_v2.0	6051530	6053094
XLOC_000391	Ca_v2.0_00739	Ca1_v2.0	6295799	6298629
XLOC_000397	Ca_v2.0_00753	Ca1_v2.0	6427381	6428094
XLOC_000399	Ca_v2.0_00756	Ca1_v2.0	6453624	6454350

For Review Only

XLOC_000405	Ca_v2.0_00766	Ca1_v2.0	6563389	6568937
XLOC_000420	Ca_v2.0_00787	Ca1_v2.0	6781932	6783428
XLOC_000422	Ca_v2.0_00789	Ca1_v2.0	6799453	6801616
XLOC_000425	-	Ca1_v2.0	6862399	6867242
XLOC_000427	Ca_v2.0_00799	Ca1_v2.0	6902435	6911834
XLOC_000432	Ca_v2.0_00806	Ca1_v2.0	6937909	6940808
XLOC_000438	Ca_v2.0_00825	Ca1_v2.0	7220318	7222789
XLOC_000444	-	Ca1_v2.0	7260684	7270750
XLOC_000448	Ca_v2.0_00833	Ca1_v2.0	7320630	7323403
XLOC_000460	Ca_v2.0_00854	Ca1_v2.0	7561109	7574867
XLOC_000465	Ca_v2.0_00863	Ca1_v2.0	7664865	7666618
XLOC_000466	Ca_v2.0_00864	Ca1_v2.0	7679214	7685826
XLOC_000471	Ca_v2.0_00870	Ca1_v2.0	7722065	7780916
XLOC_000472	Ca_v2.0_00871	Ca1_v2.0	7806515	7818502
XLOC_000480	Ca_v2.0_00891	Ca1_v2.0	8033578	8038623
XLOC_000489	Ca_v2.0_00908	Ca1_v2.0	8283486	8285682
XLOC_000491	Ca_v2.0_00911	Ca1_v2.0	8373488	8374723
XLOC_000493	Ca_v2.0_00914	Ca1_v2.0	8387830	8404764
XLOC_000496	Ca_v2.0_00917	Ca1_v2.0	8457184	8457972
XLOC_000498	Ca_v2.0_00924	Ca1_v2.0	8527971	8531969
XLOC_000500	Ca_v2.0_00926	Ca1_v2.0	8550699	8551321
XLOC_000506	Ca_v2.0_00940	Ca1_v2.0	8729184	8730135
XLOC_000507	Ca_v2.0_00941	Ca1_v2.0	8740221	8741763
XLOC_000509	Ca_v2.0_00943	Ca1_v2.0	8755071	8764485
XLOC_000510	Ca_v2.0_00945	Ca1_v2.0	8782889	8786288
XLOC_000516	Ca_v2.0_00955	Ca1_v2.0	8945620	8952675
XLOC_000529	Ca_v2.0_00975	Ca1_v2.0	9108648	9109805
XLOC_000530	Ca_v2.0_00977	Ca1_v2.0	9111011	9122763
XLOC_000531	Ca_v2.0_00983	Ca1_v2.0	9240085	9241051
XLOC_000541	Ca_v2.0_00999	Ca1_v2.0	9450271	9457396
XLOC_000555	Ca_v2.0_01026	Ca1_v2.0	9778863	9780151
XLOC_000575	Ca_v2.0_01052	Ca1_v2.0	10140497	10143148
XLOC_000586	Ca_v2.0_01064	Ca1_v2.0	10319435	10321958
XLOC_000588	Ca_v2.0_01068	Ca1_v2.0	10379074	10379860
XLOC_000591	Ca_v2.0_01072	Ca1_v2.0	10504279	10516041
XLOC_000595	Ca_v2.0_01078	Ca1_v2.0	10629563	10634849
XLOC_000611	Ca_v2.0_01103	Ca1_v2.0	10995648	10996509
XLOC_000618	Ca_v2.0_01114	Ca1_v2.0	11178894	11182294
XLOC_000620	Ca_v2.0_01118	Ca1_v2.0	11249060	11259149
XLOC_000622	Ca_v2.0_01121	Ca1_v2.0	11318571	11322294
XLOC_000630	Ca_v2.0_01138	Ca1_v2.0	11654355	11662935
XLOC_000636	Ca_v2.0_01152	Ca1_v2.0	11910199	11911316
XLOC_000643	Ca_v2.0_01160	Ca1_v2.0	13581333	13585537
XLOC_000649	Ca_v2.0_01169	Ca1_v2.0	13811394	13814534
XLOC_000651	Ca_v2.0_01173	Ca1_v2.0	13879333	13881017
XLOC_000658	Ca_v2.0_01182	Ca1_v2.0	14079497	14081848

For Review Only

XLOC_000661	Ca_v2.0_01186	Ca1_v2.0	14197148	14204689
XLOC_000670	Ca_v2.0_01198	Ca1_v2.0	14539240	14544730
XLOC_000672	Ca_v2.0_01204	Ca1_v2.0	14755874	14759805
XLOC_000677	Ca_v2.0_01210	Ca1_v2.0	14848783	14855673
XLOC_000680	Ca_v2.0_01213	Ca1_v2.0	14880947	14882646
XLOC_000690	Ca_v2.0_01230	Ca1_v2.0	15169254	15173030
XLOC_000692	Ca_v2.0_01233	Ca1_v2.0	15222800	15226647
XLOC_000694	Ca_v2.0_01238	Ca1_v2.0	15296782	15297583
XLOC_000697	Ca_v2.0_01244	Ca1_v2.0	15440354	15443418
XLOC_000701	Ca_v2.0_01250	Ca1_v2.0	15541856	15550104
XLOC_000714	-	Ca1_v2.0	16883399	16885412
XLOC_000715	Ca_v2.0_01274	Ca1_v2.0	16902801	16905897
XLOC_000717	-	Ca1_v2.0	17010750	17030161
XLOC_000719	Ca_v2.0_01277	Ca1_v2.0	17050566	17051724
XLOC_000743	Ca_v2.0_01333	Ca1_v2.0	18616517	18620606
XLOC_000758	Ca_v2.0_01369	Ca1_v2.0	20599386	20601424
XLOC_000787	Ca_v2.0_01430	Ca1_v2.0	23310975	23328682
XLOC_000792	Ca_v2.0_01441	Ca1_v2.0	23624503	23626039
XLOC_000806	Ca_v2.0_01469	Ca1_v2.0	25268337	25270081
XLOC_000812	Ca_v2.0_01480	Ca1_v2.0	25813218	25816743
XLOC_000821	Ca_v2.0_01497	Ca1_v2.0	26954145	26959238
XLOC_000828	Ca_v2.0_01508	Ca1_v2.0	29227721	29231232
XLOC_000832	Ca_v2.0_01513	Ca1_v2.0	29557531	29561161
XLOC_000838	Ca_v2.0_01526	Ca1_v2.0	30613883	30617982
XLOC_000855	Ca_v2.0_01558	Ca1_v2.0	32535910	32664157
XLOC_000861	Ca_v2.0_01573	Ca1_v2.0	32932043	32935979
XLOC_000879	Ca_v2.0_01614	Ca1_v2.0	34490037	34544641
XLOC_000883	-	Ca1_v2.0	34572430	34573108
XLOC_000889	Ca_v2.0_01629	Ca1_v2.0	34851582	34856233
XLOC_000891	Ca_v2.0_01636	Ca1_v2.0	35340472	35341721
XLOC_000898	Ca_v2.0_01648	Ca1_v2.0	36081969	36084882
XLOC_000914	Ca_v2.0_01680	Ca1_v2.0	37459531	37468387
XLOC_000935	Ca_v2.0_01715	Ca1_v2.0	39144307	39151061
XLOC_000946	-	Ca1_v2.0	39904539	39971292
XLOC_000954	Ca_v2.0_01746	Ca1_v2.0	40248983	40253035
XLOC_000957	-	Ca1_v2.0	40461219	40464120
XLOC_000964	Ca_v2.0_01763	Ca1_v2.0	40730140	40732565
XLOC_000968	Ca_v2.0_01772	Ca1_v2.0	40914844	40915613
XLOC_000969	Ca_v2.0_01774	Ca1_v2.0	40935040	40935637
XLOC_000987	Ca_v2.0_01813	Ca1_v2.0	41917098	41922930
XLOC_000991	Ca_v2.0_01820	Ca1_v2.0	42056544	42058727
XLOC_000996	Ca_v2.0_01829	Ca1_v2.0	42202163	42203549
XLOC_000997	Ca_v2.0_01830	Ca1_v2.0	42211636	42212782
XLOC_001006	Ca_v2.0_01847	Ca1_v2.0	42502313	42507450
XLOC_001008	Ca_v2.0_01852	Ca1_v2.0	42587145	42590525
XLOC_001012	Ca_v2.0_01857	Ca1_v2.0	42697763	42699443

For Review Only

XLOC_001014	Ca_v2.0_01859	Ca1_v2.0	42727424	42728074
XLOC_001018	Ca_v2.0_01866	Ca1_v2.0	42802204	42803409
XLOC_001020	Ca_v2.0_01870	Ca1_v2.0	42846812	42851695
XLOC_001021	Ca_v2.0_01871	Ca1_v2.0	42853529	42858691
XLOC_001024	Ca_v2.0_01876	Ca1_v2.0	43044799	43045142
XLOC_001030	Ca_v2.0_01893	Ca1_v2.0	43227368	43248832
XLOC_001036	Ca_v2.0_01900	Ca1_v2.0	43339299	43345393
XLOC_001037	Ca_v2.0_01901	Ca1_v2.0	43350254	43354496
XLOC_001038	Ca_v2.0_01905	Ca1_v2.0	43535549	43540682
XLOC_001041	Ca_v2.0_01911	Ca1_v2.0	43558836	43634908
XLOC_001043	Ca_v2.0_01914	Ca1_v2.0	43675578	43679651
XLOC_001049	Ca_v2.0_01932	Ca1_v2.0	43959662	43963406
XLOC_001052	Ca_v2.0_01938	Ca1_v2.0	44057755	44061180
XLOC_001053	Ca_v2.0_01941	Ca1_v2.0	44115020	44117860
XLOC_001057	-	Ca1_v2.0	44267594	44268817
XLOC_001058	Ca_v2.0_01953	Ca1_v2.0	44339486	44391875
XLOC_001059	Ca_v2.0_01954	Ca1_v2.0	44439906	44443456
XLOC_001066	Ca_v2.0_01962	Ca1_v2.0	44504729	44511983
XLOC_001077	Ca_v2.0_01979	Ca1_v2.0	44871901	44873607
XLOC_001078	Ca_v2.0_01980	Ca1_v2.0	44911357	44917238
XLOC_001081	Ca_v2.0_01983	Ca1_v2.0	44940399	44942085
XLOC_001089	Ca_v2.0_01999	Ca1_v2.0	45153722	45155790
XLOC_001090	Ca_v2.0_02001	Ca1_v2.0	45171491	45175551
XLOC_001101	Ca_v2.0_02026	Ca1_v2.0	45534923	45538720
XLOC_001102	Ca_v2.0_02032	Ca1_v2.0	45589952	45597113
XLOC_001103	Ca_v2.0_02034	Ca1_v2.0	45621369	45624326
XLOC_001105	Ca_v2.0_02038	Ca1_v2.0	45680863	45681583
XLOC_001119	Ca_v2.0_02058	Ca1_v2.0	45930737	45931243
XLOC_001120	Ca_v2.0_02059	Ca1_v2.0	45936058	45940114
XLOC_001125	Ca_v2.0_02070	Ca1_v2.0	46056056	46058106
XLOC_001126	Ca_v2.0_02071	Ca1_v2.0	46067124	46072832
XLOC_001131	Ca_v2.0_02078	Ca1_v2.0	46189791	46192025
XLOC_001135	Ca_v2.0_02084	Ca1_v2.0	46292110	46297627
XLOC_001136	Ca_v2.0_02086	Ca1_v2.0	46315568	46318836
XLOC_001139	Ca_v2.0_02091	Ca1_v2.0	46349934	46351665
XLOC_001143	Ca_v2.0_02099	Ca1_v2.0	46458716	46465738
XLOC_001144	Ca_v2.0_02102	Ca1_v2.0	46476599	46479347
XLOC_001148	-	Ca1_v2.0	46539811	46544048
XLOC_001175	Ca_v2.0_02155	Ca1_v2.0	47220633	47234287
XLOC_001184	Ca_v2.0_02174	Ca1_v2.0	47479309	47484229
XLOC_001185	Ca_v2.0_02175	Ca1_v2.0	47491709	47493830
XLOC_001186	Ca_v2.0_02177	Ca1_v2.0	47532582	47533473
XLOC_001187	Ca_v2.0_02178	Ca1_v2.0	47546018	47547499
XLOC_001194	Ca_v2.0_02188	Ca1_v2.0	47634126	47639019
XLOC_001197	Ca_v2.0_02192	Ca1_v2.0	47672474	47675727
XLOC_001206	Ca_v2.0_02205	Ca1_v2.0	47847522	47850677

For Review Only

XLOC_001208	Ca_v2.0_02208	Ca1_v2.0	47882682	47884773
XLOC_001211	Ca_v2.0_02214	Ca1_v2.0	47935551	47940878
XLOC_001215	Ca_v2.0_02224	Ca1_v2.0	48068678	48069638
XLOC_001216	Ca_v2.0_02228	Ca1_v2.0	48118530	48126813
XLOC_001222	Ca_v2.0_02234	Ca1_v2.0	48172057	48176415
XLOC_001231	Ca_v2.0_02244	Ca1_v2.0	48233466	48236035
XLOC_001232	Ca_v2.0_02245	Ca1_v2.0	48238512	48240230
XLOC_001236	Ca_v2.0_02249	Ca1_v2.0	48271178	48272602
XLOC_001237	Ca_v2.0_02250	Ca1_v2.0	48275715	48279298
XLOC_001238	Ca_v2.0_02251	Ca1_v2.0	48281612	48285487
XLOC_001244	Ca_v2.0_02269	Ca1_v2.0	48439075	48447379
XLOC_001247	Ca_v2.0_02274	Ca1_v2.0	48503746	48505727
XLOC_001255	Ca_v2.0_02290	Ca1_v2.0	48671846	48673084
XLOC_001257	-	Ca1_v2.0	48716480	48722094
XLOC_001261	Ca_v2.0_02303	Ca1_v2.0	48845462	48849682
XLOC_001266	Ca_v2.0_02311	Ca1_v2.0	48915137	48916126
XLOC_001271	-	Ca1_v2.0	49049880	49052069
XLOC_001273	Ca_v2.0_02328	Ca1_v2.0	49084919	49085772
XLOC_001274	Ca_v2.0_02329	Ca1_v2.0	49089916	49094006
XLOC_001278	Ca_v2.0_02338	Ca1_v2.0	49164641	49170950
XLOC_001280	Ca_v2.0_02340	Ca1_v2.0	49208067	49210971
XLOC_001282	Ca_v2.0_02342	Ca1_v2.0	49218130	49218782
XLOC_001284	Ca_v2.0_02347	Ca1_v2.0	49286853	49289531
XLOC_001287	Ca_v2.0_02351	Ca1_v2.0	49327276	49330073
XLOC_001292	Ca_v2.0_02359	Ca1_v2.0	49415602	49416117
XLOC_001293	Ca_v2.0_02360	Ca1_v2.0	49417651	49418160
XLOC_001294	Ca_v2.0_02361	Ca1_v2.0	49420486	49420872
XLOC_001295	Ca_v2.0_02362	Ca1_v2.0	49423049	49424249
XLOC_001296	Ca_v2.0_02363	Ca1_v2.0	49436109	49436827
XLOC_001309	Ca_v2.0_02378	Ca1_v2.0	49555617	49559619
XLOC_001314	Ca_v2.0_02386	Ca1_v2.0	49625988	49628151
XLOC_001326	Ca_v2.0_02404	Ca1_v2.0	49756333	49808709
XLOC_001327	Ca_v2.0_02404	Ca1_v2.0	49756333	49808709
XLOC_001332	Ca_v2.0_02415	Ca1_v2.0	49864417	49867437
XLOC_001336	Ca_v2.0_02422	Ca1_v2.0	49980495	49981247
XLOC_001338	Ca_v2.0_02424,Ca_v2.0_024:	Ca1_v2.0	50001848	50005601
XLOC_001341	Ca_v2.0_02433	Ca1_v2.0	50080008	50082537
XLOC_001350	-	Ca1_v2.0	50280377	50283648
XLOC_001354	Ca_v2.0_02466	Ca1_v2.0	50399352	50404741
XLOC_001355	Ca_v2.0_02479	Ca1_v2.0	50473080	50561960
XLOC_001356	Ca_v2.0_02474	Ca1_v2.0	50473080	50561960
XLOC_001358	Ca_v2.0_02478	Ca1_v2.0	50473080	50561960
XLOC_001363	Ca_v2.0_02488	Ca1_v2.0	50596580	50602442
XLOC_001378	Ca_v2.0_02516	Ca1_v2.0	50898584	50900463
XLOC_001380	Ca_v2.0_02520	Ca1_v2.0	50919258	50926230
XLOC_001384	Ca_v2.0_02525	Ca1_v2.0	50990935	50997464

For Review Only

XLOC_001396	Ca_v2.0_02548	Ca1_v2.0	51204699	51205439
XLOC_001401	Ca_v2.0_02556	Ca1_v2.0	51308150	51331747
XLOC_001409	Ca_v2.0_02575	Ca1_v2.0	51460055	51462194
XLOC_001429	Ca_v2.0_02607	Ca1_v2.0	51702859	51710379
XLOC_001435	Ca_v2.0_02620	Ca1_v2.0	51804247	51812404
XLOC_001436	Ca_v2.0_02621	Ca1_v2.0	51819011	51820532
XLOC_001446	Ca_v2.0_02644	Ca1_v2.0	52049113	52055150
XLOC_001450	Ca_v2.0_02649,Ca_v2.0_026!	Ca1_v2.0	52097215	52102585
XLOC_001458	Ca_v2.0_02670	Ca1_v2.0	52204040	52206070
XLOC_001496	Ca_v2.0_02743	Ca1_v2.0	52805808	52810753
XLOC_001500	Ca_v2.0_02757	Ca1_v2.0	52958643	52964322
XLOC_001510	Ca_v2.0_02772	Ca1_v2.0	53077054	53116508
XLOC_001511	Ca_v2.0_02775	Ca1_v2.0	53077054	53116508
XLOC_001513	Ca_v2.0_02779	Ca1_v2.0	53125903	53131324
XLOC_001515	Ca_v2.0_02783	Ca1_v2.0	53143114	53146199
XLOC_001525	Ca_v2.0_02798	Ca1_v2.0	53247152	53249994
XLOC_001526	Ca_v2.0_02800	Ca1_v2.0	53276843	53279818
XLOC_001527	Ca_v2.0_02801	Ca1_v2.0	53281710	53293963
XLOC_001531	Ca_v2.0_02807	Ca1_v2.0	53338326	53343451
XLOC_001534	Ca_v2.0_02811	Ca1_v2.0	53382304	53384322
XLOC_001535	Ca_v2.0_02812	Ca1_v2.0	53393497	53411010
XLOC_001536	Ca_v2.0_02813	Ca1_v2.0	53393497	53411010
XLOC_001542	Ca_v2.0_02822	Ca1_v2.0	53485780	53488437
XLOC_001544	Ca_v2.0_02824	Ca1_v2.0	53501681	53507987
XLOC_001564	Ca_v2.0_02853	Ca1_v2.0	53692977	53726896
XLOC_001567	Ca_v2.0_02860	Ca1_v2.0	53749042	53749841
XLOC_001583	Ca_v2.0_02890	Ca1_v2.0	53965581	53969264
XLOC_001588	Ca_v2.0_02902	Ca1_v2.0	54030528	54034569
XLOC_001589	Ca_v2.0_02905	Ca1_v2.0	54065858	54067273
XLOC_001593	Ca_v2.0_02913	Ca1_v2.0	54117072	54119706
XLOC_001594	Ca_v2.0_02916	Ca1_v2.0	54140274	54154020
XLOC_001595	Ca_v2.0_02917	Ca1_v2.0	54140274	54154020
XLOC_001596	Ca_v2.0_02921	Ca1_v2.0	54164014	54167180
XLOC_001598	Ca_v2.0_02927	Ca1_v2.0	54210812	54214599
XLOC_001606	-	Ca1_v2.0	19003	21106
XLOC_001607	Ca_v2.0_00004,Ca_v2.0_000(	Ca1_v2.0	40080	49370
XLOC_001611	Ca_v2.0_00012	Ca1_v2.0	127934	130132
XLOC_001616	Ca_v2.0_00028	Ca1_v2.0	266056	267778
XLOC_001632	Ca_v2.0_00051	Ca1_v2.0	424799	425859
XLOC_001640	Ca_v2.0_00061	Ca1_v2.0	500563	506678
XLOC_001645	Ca_v2.0_00068	Ca1_v2.0	554038	584040
XLOC_001660	Ca_v2.0_00100	Ca1_v2.0	789049	811463
XLOC_001662	-	Ca1_v2.0	832399	833456
XLOC_001663	Ca_v2.0_00102	Ca1_v2.0	836256	839824
XLOC_001667	Ca_v2.0_00113	Ca1_v2.0	896330	897526
XLOC_001668	Ca_v2.0_00115	Ca1_v2.0	905009	906746

For Review Only

XLOC_001669	Ca_v2.0_00116	Ca1_v2.0	908072	915930
XLOC_001672	Ca_v2.0_00124	Ca1_v2.0	978896	979494
XLOC_001673	Ca_v2.0_00125	Ca1_v2.0	984342	986121
XLOC_001675	Ca_v2.0_00128,Ca_v2.0_001:	Ca1_v2.0	996631	1003275
XLOC_001676	Ca_v2.0_00130	Ca1_v2.0	1005521	1007603
XLOC_001681	Ca_v2.0_00137	Ca1_v2.0	1063452	1066666
XLOC_001683	Ca_v2.0_00141	Ca1_v2.0	1081837	1084054
XLOC_001694	Ca_v2.0_00163	Ca1_v2.0	1240959	1241765
XLOC_001695	Ca_v2.0_00164	Ca1_v2.0	1243555	1244222
XLOC_001700	Ca_v2.0_00175	Ca1_v2.0	1315157	1316007
XLOC_001701	Ca_v2.0_00176	Ca1_v2.0	1321744	1327713
XLOC_001708	Ca_v2.0_00190	Ca1_v2.0	1442307	1443515
XLOC_001712	-	Ca1_v2.0	1511888	1512391
XLOC_001716	Ca_v2.0_00213	Ca1_v2.0	1642678	1645616
XLOC_001727	Ca_v2.0_00230	Ca1_v2.0	1739291	1749805
XLOC_001734	Ca_v2.0_00246	Ca1_v2.0	1849427	1850807
XLOC_001736	Ca_v2.0_00248	Ca1_v2.0	1863740	1865557
XLOC_001751	Ca_v2.0_00272	Ca1_v2.0	2025398	2030072
XLOC_001755	Ca_v2.0_00277	Ca1_v2.0	2079628	2083947
XLOC_001763	Ca_v2.0_00290	Ca1_v2.0	2215439	2221242
XLOC_001765	Ca_v2.0_00292	Ca1_v2.0	2240821	2243337
XLOC_001766	Ca_v2.0_00293	Ca1_v2.0	2250661	2254438
XLOC_001770	Ca_v2.0_00304,Ca_v2.0_003(	Ca1_v2.0	2330666	2333439
XLOC_001774	Ca_v2.0_00315	Ca1_v2.0	2377765	2379948
XLOC_001776	Ca_v2.0_00319	Ca1_v2.0	2401046	2407270
XLOC_001781	Ca_v2.0_00333	Ca1_v2.0	2571268	2572652
XLOC_001783	Ca_v2.0_00336	Ca1_v2.0	2619848	2621371
XLOC_001784	Ca_v2.0_00338	Ca1_v2.0	2624956	2633053
XLOC_001796	Ca_v2.0_00360	Ca1_v2.0	2824369	2825977
XLOC_001805	Ca_v2.0_00380	Ca1_v2.0	2988781	2994176
XLOC_001811	Ca_v2.0_00395	Ca1_v2.0	3091559	3093589
XLOC_001814	Ca_v2.0_00399	Ca1_v2.0	3121204	3127092
XLOC_001817	Ca_v2.0_00404	Ca1_v2.0	3164402	3167353
XLOC_001818	Ca_v2.0_00409	Ca1_v2.0	3198352	3199940
XLOC_001821	Ca_v2.0_00413	Ca1_v2.0	3241019	3242589
XLOC_001828	Ca_v2.0_00431	Ca1_v2.0	3399221	3401062
XLOC_001829	Ca_v2.0_00432	Ca1_v2.0	3401693	3407530
XLOC_001831	Ca_v2.0_00434	Ca1_v2.0	3420101	3420964
XLOC_001832	Ca_v2.0_00436	Ca1_v2.0	3424615	3431007
XLOC_001867	Ca_v2.0_00508	Ca1_v2.0	4058263	4059247
XLOC_001871	Ca_v2.0_00519	Ca1_v2.0	4155787	4157497
XLOC_001875	Ca_v2.0_00526	Ca1_v2.0	4198071	4206352
XLOC_001876	Ca_v2.0_00528	Ca1_v2.0	4244864	4245884
XLOC_001877	Ca_v2.0_00531	Ca1_v2.0	4270562	4272778
XLOC_001891	Ca_v2.0_00557,Ca_v2.0_005!	Ca1_v2.0	4557091	4566729
XLOC_001896	Ca_v2.0_00565	Ca1_v2.0	4641670	4652479

For Review Only

XLOC_001910	Ca_v2.0_00589	Ca1_v2.0	4880024	4882077
XLOC_001923	Ca_v2.0_00608	Ca1_v2.0	5063678	5064311
XLOC_001926	Ca_v2.0_00614	Ca1_v2.0	5093145	5097455
XLOC_001927	Ca_v2.0_00615	Ca1_v2.0	5101276	5104482
XLOC_001934	Ca_v2.0_00624	Ca1_v2.0	5156336	5157492
XLOC_001937	Ca_v2.0_00627	Ca1_v2.0	5197056	5201552
XLOC_001938	Ca_v2.0_00628	Ca1_v2.0	5202416	5211945
XLOC_001939	Ca_v2.0_00631	Ca1_v2.0	5235798	5237422
XLOC_001940	Ca_v2.0_00636,Ca_v2.0_006	Ca1_v2.0	5250189	5432383
XLOC_001943	Ca_v2.0_00641	Ca1_v2.0	5250189	5432383
XLOC_001952	Ca_v2.0_00667	Ca1_v2.0	5592043	5595008
XLOC_001953	Ca_v2.0_00672	Ca1_v2.0	5619666	5621212
XLOC_001954	Ca_v2.0_00673	Ca1_v2.0	5628543	5630057
XLOC_001962	Ca_v2.0_00688	Ca1_v2.0	5718141	5726152
XLOC_001980	Ca_v2.0_00717	Ca1_v2.0	6054428	6057836
XLOC_001986	Ca_v2.0_00727	Ca1_v2.0	6161154	6163741
XLOC_001990	Ca_v2.0_00732	Ca1_v2.0	6247318	6251780
XLOC_002004	Ca_v2.0_00751	Ca1_v2.0	6406729	6408594
XLOC_002010	Ca_v2.0_00770	Ca1_v2.0	6597954	6611534
XLOC_002012	Ca_v2.0_00773,Ca_v2.0_007	Ca1_v2.0	6627885	6634261
XLOC_002015	Ca_v2.0_00783	Ca1_v2.0	6750967	6754626
XLOC_002035	Ca_v2.0_00815	Ca1_v2.0	7062448	7070120
XLOC_002043	Ca_v2.0_00829	Ca1_v2.0	7260684	7270750
XLOC_002058	Ca_v2.0_00870	Ca1_v2.0	7722065	7780916
XLOC_002059	-	Ca1_v2.0	7791051	7791932
XLOC_002063	Ca_v2.0_00875	Ca1_v2.0	7846281	7848189
XLOC_002079	Ca_v2.0_00901	Ca1_v2.0	8137676	8145623
XLOC_002086	Ca_v2.0_00918	Ca1_v2.0	8464435	8467720
XLOC_002088	Ca_v2.0_00921	Ca1_v2.0	8490693	8494292
XLOC_002093	Ca_v2.0_00930	Ca1_v2.0	8614686	8621385
XLOC_002098	Ca_v2.0_00938	Ca1_v2.0	8693200	8701562
XLOC_002099	Ca_v2.0_00941	Ca1_v2.0	8740221	8741763
XLOC_002103	Ca_v2.0_00949	Ca1_v2.0	8843237	8873071
XLOC_002109	Ca_v2.0_00962	Ca1_v2.0	9019297	9019802
XLOC_002110	Ca_v2.0_00965	Ca1_v2.0	9037217	9039040
XLOC_002114	Ca_v2.0_00976,Ca_v2.0_009	Ca1_v2.0	9111011	9122763
XLOC_002116	Ca_v2.0_00980	Ca1_v2.0	9137612	9141519
XLOC_002119	Ca_v2.0_00985	Ca1_v2.0	9250292	9258471
XLOC_002131	Ca_v2.0_01012	Ca1_v2.0	9598507	9607832
XLOC_002132	Ca_v2.0_01014	Ca1_v2.0	9626040	9638544
XLOC_002149	Ca_v2.0_01072	Ca1_v2.0	10504279	10516041
XLOC_002157	Ca_v2.0_01085	Ca1_v2.0	10751146	10752151
XLOC_002189	Ca_v2.0_01155	Ca1_v2.0	11950528	11961995
XLOC_002205	Ca_v2.0_01195	Ca1_v2.0	14477697	14483134
XLOC_002211	Ca_v2.0_01207	Ca1_v2.0	14799835	14810031
XLOC_002220	Ca_v2.0_01231	Ca1_v2.0	15181747	15182581

For Review Only

XLOC_002229	Ca_v2.0_01253	Ca1_v2.0	15594613	15599127
XLOC_002242	-	Ca1_v2.0	17010750	17030161
XLOC_002244	Ca_v2.0_01276	Ca1_v2.0	17042035	17044089
XLOC_002246	Ca_v2.0_01277	Ca1_v2.0	17050566	17051724
XLOC_002247	Ca_v2.0_01278	Ca1_v2.0	17054783	17111502
XLOC_002255	Ca_v2.0_01292	Ca1_v2.0	17516452	17517520
XLOC_002264	Ca_v2.0_01310	Ca1_v2.0	18068483	18074718
XLOC_002268	-	Ca1_v2.0	18246455	18247503
XLOC_002275	Ca_v2.0_01324	Ca1_v2.0	18379366	18382838
XLOC_002277	Ca_v2.0_01327	Ca1_v2.0	18439567	18440212
XLOC_002279	Ca_v2.0_01329	Ca1_v2.0	18478500	18479499
XLOC_002282	Ca_v2.0_01339	Ca1_v2.0	18928668	18932576
XLOC_002286	Ca_v2.0_01346	Ca1_v2.0	19175394	19176180
XLOC_002288	Ca_v2.0_01349	Ca1_v2.0	19322544	19325090
XLOC_002289	Ca_v2.0_01350	Ca1_v2.0	19342775	19350227
XLOC_002290	Ca_v2.0_01351	Ca1_v2.0	19450584	19452278
XLOC_002297	Ca_v2.0_01362	Ca1_v2.0	19826444	19828084
XLOC_002302	Ca_v2.0_01371,Ca_v2.0_013	Ca1_v2.0	20911165	20914839
XLOC_002306	Ca_v2.0_01382	Ca1_v2.0	21155734	21158663
XLOC_002314	Ca_v2.0_01398	Ca1_v2.0	21546471	21566159
XLOC_002317	Ca_v2.0_01402	Ca1_v2.0	21628389	21632786
XLOC_002319	Ca_v2.0_01409	Ca1_v2.0	21986919	22002905
XLOC_002328	Ca_v2.0_01420	Ca1_v2.0	22428342	22434819
XLOC_002337	Ca_v2.0_01435	Ca1_v2.0	23408883	23413524
XLOC_002341	Ca_v2.0_01441	Ca1_v2.0	23624503	23626039
XLOC_002346	Ca_v2.0_01447	Ca1_v2.0	24067383	24068047
XLOC_002350	Ca_v2.0_01451	Ca1_v2.0	24303938	24317387
XLOC_002375	Ca_v2.0_01493	Ca1_v2.0	26745188	26753727
XLOC_002377	Ca_v2.0_01497	Ca1_v2.0	26954145	26959238
XLOC_002378	Ca_v2.0_01498	Ca1_v2.0	26988424	26992462
XLOC_002380	Ca_v2.0_01506	Ca1_v2.0	27664894	27669194
XLOC_002382	Ca_v2.0_01514	Ca1_v2.0	29633021	29635390
XLOC_002405	Ca_v2.0_01546	Ca1_v2.0	31871557	31874336
XLOC_002408	-	Ca1_v2.0	32532913	32535303
XLOC_002418	Ca_v2.0_01572	Ca1_v2.0	32884321	32917951
XLOC_002427	Ca_v2.0_01582	Ca1_v2.0	33365648	33369468
XLOC_002430	Ca_v2.0_01587	Ca1_v2.0	33519930	33520391
XLOC_002444	Ca_v2.0_01614,Ca_v2.0_016	Ca1_v2.0	34490037	34544641
XLOC_002445	Ca_v2.0_01615	Ca1_v2.0	34490037	34544641
XLOC_002449	Ca_v2.0_01625	Ca1_v2.0	34623216	34626198
XLOC_002456	Ca_v2.0_01637	Ca1_v2.0	35491096	35500249
XLOC_002463	Ca_v2.0_01648	Ca1_v2.0	36081969	36084882
XLOC_002474	Ca_v2.0_01671	Ca1_v2.0	37177845	37234330
XLOC_002481	Ca_v2.0_01683	Ca1_v2.0	37708440	37709445
XLOC_002482	Ca_v2.0_01684	Ca1_v2.0	37749852	37750478
XLOC_002488	Ca_v2.0_01691	Ca1_v2.0	37922545	37923338

For Review Only

XLOC_002489	Ca_v2.0_01694	Ca1_v2.0	37993931	37997794
XLOC_002502	Ca_v2.0_01722	Ca1_v2.0	39469971	39474595
XLOC_002511	Ca_v2.0_01738	Ca1_v2.0	40066060	40067742
XLOC_002514	Ca_v2.0_01744	Ca1_v2.0	40207866	40210141
XLOC_002517	Ca_v2.0_01748	Ca1_v2.0	40304517	40306805
XLOC_002520	Ca_v2.0_01753	Ca1_v2.0	40401214	40403989
XLOC_002521	Ca_v2.0_01754	Ca1_v2.0	40436914	40438473
XLOC_002533	Ca_v2.0_01779	Ca1_v2.0	41028384	41036580
XLOC_002548	Ca_v2.0_01802	Ca1_v2.0	41642450	41643498
XLOC_002553	Ca_v2.0_01817	Ca1_v2.0	41974895	41978449
XLOC_002556	Ca_v2.0_01822	Ca1_v2.0	42080999	42083919
XLOC_002557	Ca_v2.0_01823	Ca1_v2.0	42090524	42137696
XLOC_002567	Ca_v2.0_01844	Ca1_v2.0	42441473	42444227
XLOC_002569	Ca_v2.0_01850	Ca1_v2.0	42557886	42560250
XLOC_002573	Ca_v2.0_01862,Ca_v2.0_018(	Ca1_v2.0	42763186	42783087
XLOC_002574	Ca_v2.0_01867	Ca1_v2.0	42806213	42815057
XLOC_002577	Ca_v2.0_01872	Ca1_v2.0	42887963	42890374
XLOC_002580	Ca_v2.0_01881	Ca1_v2.0	43107950	43110682
XLOC_002583	-	Ca1_v2.0	43182548	43184663
XLOC_002584	Ca_v2.0_01884	Ca1_v2.0	43188590	43189483
XLOC_002585	Ca_v2.0_01885	Ca1_v2.0	43199721	43200316
XLOC_002586	Ca_v2.0_01886	Ca1_v2.0	43210032	43212156
XLOC_002588	Ca_v2.0_01897	Ca1_v2.0	43292552	43298739
XLOC_002590	-	Ca1_v2.0	43455618	43455957
XLOC_002592	Ca_v2.0_01904	Ca1_v2.0	43497393	43498765
XLOC_002598	-	Ca1_v2.0	43666630	43668130
XLOC_002604	Ca_v2.0_01923	Ca1_v2.0	43796713	43799414
XLOC_002606	Ca_v2.0_01927	Ca1_v2.0	43821425	43825637
XLOC_002607	Ca_v2.0_01928	Ca1_v2.0	43848190	43849090
XLOC_002610	Ca_v2.0_01931	Ca1_v2.0	43957182	43958339
XLOC_002612	Ca_v2.0_01933	Ca1_v2.0	43966776	43970928
XLOC_002618	Ca_v2.0_01943	Ca1_v2.0	44127009	44132690
XLOC_002625	Ca_v2.0_01951	Ca1_v2.0	44316035	44320540
XLOC_002627	-	Ca1_v2.0	44339486	44391875
XLOC_002628	Ca_v2.0_01953	Ca1_v2.0	44339486	44391875
XLOC_002630	-	Ca1_v2.0	44339486	44391875
XLOC_002631	-	Ca1_v2.0	44424364	44425290
XLOC_002638	Ca_v2.0_01978	Ca1_v2.0	44842606	44846193
XLOC_002639	Ca_v2.0_01980	Ca1_v2.0	44911357	44917238
XLOC_002642	Ca_v2.0_01988	Ca1_v2.0	45020730	45030425
XLOC_002644	Ca_v2.0_01990	Ca1_v2.0	45052082	45056315
XLOC_002651	Ca_v2.0_02002	Ca1_v2.0	45177490	45183713
XLOC_002658	Ca_v2.0_02014	Ca1_v2.0	45382691	45387553
XLOC_002666	Ca_v2.0_02028	Ca1_v2.0	45548421	45549243
XLOC_002671	Ca_v2.0_02034	Ca1_v2.0	45621369	45624326
XLOC_002684	Ca_v2.0_02067	Ca1_v2.0	46004443	46007449

For Review Only

XLOC_002686	Ca_v2.0_02070	Ca1_v2.0	46056056	46058106
XLOC_002691	Ca_v2.0_02078	Ca1_v2.0	46189791	46192025
XLOC_002693	Ca_v2.0_02083	Ca1_v2.0	46278844	46282115
XLOC_002694	Ca_v2.0_02084	Ca1_v2.0	46292110	46297627
XLOC_002696	Ca_v2.0_02087	Ca1_v2.0	46319716	46321442
XLOC_002698	Ca_v2.0_02091	Ca1_v2.0	46349934	46351665
XLOC_002701	Ca_v2.0_02096	Ca1_v2.0	46404194	46405574
XLOC_002704	Ca_v2.0_02100	Ca1_v2.0	46469035	46469825
XLOC_002705	Ca_v2.0_02101	Ca1_v2.0	46472532	46473097
XLOC_002709	Ca_v2.0_02114	Ca1_v2.0	46655581	46659656
XLOC_002713	Ca_v2.0_02119	Ca1_v2.0	46788272	46791406
XLOC_002719	Ca_v2.0_02139	Ca1_v2.0	46981635	46982479
XLOC_002725	-	Ca1_v2.0	47113008	47115438
XLOC_002726	Ca_v2.0_02151,Ca_v2.0_021!	Ca1_v2.0	47150570	47200270
XLOC_002727	-	Ca1_v2.0	47150570	47200270
XLOC_002728	Ca_v2.0_02152	Ca1_v2.0	47150570	47200270
XLOC_002737	Ca_v2.0_02167	Ca1_v2.0	47365607	47368699
XLOC_002738	Ca_v2.0_02168	Ca1_v2.0	47375848	47379111
XLOC_002744	Ca_v2.0_02188	Ca1_v2.0	47634126	47639019
XLOC_002753	Ca_v2.0_02205	Ca1_v2.0	47847522	47850677
XLOC_002754	-	Ca1_v2.0	47847522	47850677
XLOC_002755	Ca_v2.0_02206	Ca1_v2.0	47855995	47864456
XLOC_002756	Ca_v2.0_02209	Ca1_v2.0	47892556	47893990
XLOC_002761	Ca_v2.0_02217	Ca1_v2.0	47968704	47970470
XLOC_002770	Ca_v2.0_02230	Ca1_v2.0	48135197	48139953
XLOC_002772	Ca_v2.0_02233	Ca1_v2.0	48161720	48164534
XLOC_002783	Ca_v2.0_02263	Ca1_v2.0	48381191	48385446
XLOC_002788	Ca_v2.0_02268	Ca1_v2.0	48427853	48429273
XLOC_002790	Ca_v2.0_02273	Ca1_v2.0	48491370	48495340
XLOC_002799	Ca_v2.0_02291	Ca1_v2.0	48674841	48676507
XLOC_002800	Ca_v2.0_02292	Ca1_v2.0	48686417	48690135
XLOC_002801	Ca_v2.0_02293	Ca1_v2.0	48691293	48698774
XLOC_002806	Ca_v2.0_02299	Ca1_v2.0	48795722	48796397
XLOC_002810	Ca_v2.0_02310	Ca1_v2.0	48904154	48908003
XLOC_002811	Ca_v2.0_02311	Ca1_v2.0	48915137	48916126
XLOC_002824	Ca_v2.0_02326	Ca1_v2.0	49077508	49078428
XLOC_002826	Ca_v2.0_02330	Ca1_v2.0	49089916	49094006
XLOC_002827	Ca_v2.0_02331	Ca1_v2.0	49100450	49101545
XLOC_002831	Ca_v2.0_02344	Ca1_v2.0	49227688	49228401
XLOC_002841	-	Ca1_v2.0	49511061	49511841
XLOC_002845	Ca_v2.0_02380	Ca1_v2.0	49563239	49567530
XLOC_002849	Ca_v2.0_02398	Ca1_v2.0	49704565	49705474
XLOC_002854	Ca_v2.0_02408	Ca1_v2.0	49756333	49808709
XLOC_002861	Ca_v2.0_02428	Ca1_v2.0	50019260	50021854
XLOC_002862	Ca_v2.0_02429	Ca1_v2.0	50046567	50047319
XLOC_002864	Ca_v2.0_02431	Ca1_v2.0	50060543	50061737

For Review Only

XLOC_002869	Ca_v2.0_02439	Ca1_v2.0	50105512	50106552
XLOC_002870	Ca_v2.0_02440	Ca1_v2.0	50109850	50112653
XLOC_002873	Ca_v2.0_02446	Ca1_v2.0	50161703	50165409
XLOC_002878	Ca_v2.0_02453	Ca1_v2.0	50217549	50222314
XLOC_002880	Ca_v2.0_02455	Ca1_v2.0	50228683	50230402
XLOC_002883	Ca_v2.0_02458	Ca1_v2.0	50251952	50258389
XLOC_002898	Ca_v2.0_02480	Ca1_v2.0	50473080	50561960
XLOC_002900	Ca_v2.0_02485	Ca1_v2.0	50584616	50585808
XLOC_002904	Ca_v2.0_02492	Ca1_v2.0	50626944	50632514
XLOC_002914	Ca_v2.0_02507	Ca1_v2.0	50787216	50791986
XLOC_002925	Ca_v2.0_02521	Ca1_v2.0	50957330	50960095
XLOC_002936	Ca_v2.0_02544	Ca1_v2.0	51174895	51175892
XLOC_002941	Ca_v2.0_02553	Ca1_v2.0	51254112	51256296
XLOC_002943	Ca_v2.0_02557	Ca1_v2.0	51308150	51331747
XLOC_002944	Ca_v2.0_02558	Ca1_v2.0	51308150	51331747
XLOC_002953	Ca_v2.0_02572	Ca1_v2.0	51423953	51429519
XLOC_002958	Ca_v2.0_02587	Ca1_v2.0	51556762	51558744
XLOC_002962	Ca_v2.0_02595	Ca1_v2.0	51623262	51625592
XLOC_002968	Ca_v2.0_02606	Ca1_v2.0	51701627	51702215
XLOC_002975	Ca_v2.0_02617	Ca1_v2.0	51790068	51791991
XLOC_002976	Ca_v2.0_02618	Ca1_v2.0	51793146	51796441
XLOC_002979	Ca_v2.0_02623	Ca1_v2.0	51836488	51837135
XLOC_002982	Ca_v2.0_02628	Ca1_v2.0	51893540	51895094
XLOC_003003	Ca_v2.0_02669	Ca1_v2.0	52194875	52199554
XLOC_003011	-	Ca1_v2.0	52268065	52294562
XLOC_003020	Ca_v2.0_02701	Ca1_v2.0	52485397	52489636
XLOC_003021	Ca_v2.0_02703	Ca1_v2.0	52498392	52508406
XLOC_003032	Ca_v2.0_02722	Ca1_v2.0	52650586	52653338
XLOC_003036	Ca_v2.0_02728	Ca1_v2.0	52693804	52694304
XLOC_003041	Ca_v2.0_02734	Ca1_v2.0	52729613	52734053
XLOC_003045	Ca_v2.0_02744	Ca1_v2.0	52825859	52829793
XLOC_003046	Ca_v2.0_02746	Ca1_v2.0	52843757	52849600
XLOC_003047	Ca_v2.0_02748	Ca1_v2.0	52861218	52862955
XLOC_003050	Ca_v2.0_02752	Ca1_v2.0	52903449	52910719
XLOC_003052	Ca_v2.0_02753	Ca1_v2.0	52928559	52929426
XLOC_003059	Ca_v2.0_02770	Ca1_v2.0	53058519	53060301
XLOC_003064	Ca_v2.0_02782	Ca1_v2.0	53139474	53140551
XLOC_003072	Ca_v2.0_02799	Ca1_v2.0	53258773	53266153
XLOC_003076	Ca_v2.0_02812,Ca_v2.0_028	Ca1_v2.0	53393497	53411010
XLOC_003081	Ca_v2.0_02829	Ca1_v2.0	53526383	53529264
XLOC_003082	Ca_v2.0_02842	Ca1_v2.0	53622444	53631767
XLOC_003085	Ca_v2.0_02847	Ca1_v2.0	53666543	53668758
XLOC_003091	Ca_v2.0_02855	Ca1_v2.0	53692977	53726896
XLOC_003092	Ca_v2.0_02856	Ca1_v2.0	53692977	53726896
XLOC_003105	Ca_v2.0_02883	Ca1_v2.0	53903755	53905458
XLOC_003107	Ca_v2.0_02892	Ca1_v2.0	53979854	53984344

For Review Only

XLOC_003111	Ca_v2.0_02898	Ca1_v2.0	54008878	54009880
XLOC_003115	Ca_v2.0_02904	Ca1_v2.0	54053546	54055736
XLOC_003122	Ca_v2.0_02918	Ca1_v2.0	54154281	54156498
XLOC_003124	Ca_v2.0_02920	Ca1_v2.0	54164014	54167180
XLOC_003134	-	Ca1_v2.0	386622	386844
XLOC_003136	-	Ca1_v2.0	1524610	1524725
XLOC_003146	-	Ca1_v2.0	5196193	5196526
XLOC_003150	-	Ca1_v2.0	6353986	6354941
XLOC_003154	-	Ca1_v2.0	9315957	9316933
XLOC_003155	-	Ca1_v2.0	9337769	9338590
XLOC_003179	-	Ca1_v2.0	34072833	34073675
XLOC_003180	-	Ca1_v2.0	34199535	34201247
XLOC_003189	-	Ca1_v2.0	42153957	42154693
XLOC_003190	-	Ca1_v2.0	42192152	42192448
XLOC_003196	-	Ca1_v2.0	43725374	43739783
XLOC_003202	-	Ca1_v2.0	45586544	45587357
XLOC_003203	-	Ca1_v2.0	47562377	47563198
XLOC_003204	-	Ca1_v2.0	49409518	49410505
XLOC_003215	-	Ca1_v2.0	53414818	53415727
XLOC_003224	Ca_v2.0_02953	Ca2_v2.0	195932	213018
XLOC_003233	Ca_v2.0_02967	Ca2_v2.0	309981	311749
XLOC_003243	Ca_v2.0_02988	Ca2_v2.0	486480	487657
XLOC_003256	Ca_v2.0_03011	Ca2_v2.0	682700	690746
XLOC_003261	Ca_v2.0_03018	Ca2_v2.0	775905	778385
XLOC_003273	Ca_v2.0_03041,Ca_v2.0_030	Ca2_v2.0	1002566	1010018
XLOC_003277	Ca_v2.0_03047	Ca2_v2.0	1045176	1052614
XLOC_003281	Ca_v2.0_03052	Ca2_v2.0	1112210	1120650
XLOC_003282	-	Ca2_v2.0	1134674	1137324
XLOC_003283	Ca_v2.0_03054	Ca2_v2.0	1154897	1158637
XLOC_003286	Ca_v2.0_03057	Ca2_v2.0	1173912	1176283
XLOC_003287	Ca_v2.0_03058	Ca2_v2.0	1178229	1187711
XLOC_003289	Ca_v2.0_03061	Ca2_v2.0	1192178	1193726
XLOC_003290	Ca_v2.0_03063	Ca2_v2.0	1217647	1218997
XLOC_003291	Ca_v2.0_03067	Ca2_v2.0	1248014	1250249
XLOC_003305	Ca_v2.0_03097	Ca2_v2.0	1510985	1533235
XLOC_003307	Ca_v2.0_03098	Ca2_v2.0	1534649	1536372
XLOC_003315	Ca_v2.0_03113	Ca2_v2.0	1686703	1691102
XLOC_003326	Ca_v2.0_03134	Ca2_v2.0	2007280	2009958
XLOC_003327	Ca_v2.0_03135	Ca2_v2.0	2016043	2021661
XLOC_003332	Ca_v2.0_03145	Ca2_v2.0	2118657	2122284
XLOC_003346	Ca_v2.0_03171	Ca2_v2.0	2356064	2359638
XLOC_003348	Ca_v2.0_03177	Ca2_v2.0	2399624	2427066
XLOC_003349	Ca_v2.0_03178	Ca2_v2.0	2399624	2427066
XLOC_003350	Ca_v2.0_03180	Ca2_v2.0	2429643	2439741
XLOC_003353	Ca_v2.0_03185	Ca2_v2.0	2490944	2491504
XLOC_003360	Ca_v2.0_03198	Ca2_v2.0	2612958	2618381

For Review Only

XLOC_003365	-	Ca2_v2.0	2691701	2693615
XLOC_003366	Ca_v2.0_03203	Ca2_v2.0	2705096	2708997
XLOC_003367	Ca_v2.0_03204,Ca_v2.0_032(	Ca2_v2.0	2713703	2731229
XLOC_003373	Ca_v2.0_03217	Ca2_v2.0	2893826	2895539
XLOC_003377	Ca_v2.0_03222	Ca2_v2.0	2984915	2988378
XLOC_003383	Ca_v2.0_03230	Ca2_v2.0	3115637	3116441
XLOC_003393	Ca_v2.0_03246	Ca2_v2.0	3258517	3263922
XLOC_003395	Ca_v2.0_03251	Ca2_v2.0	3317963	3320430
XLOC_003404	Ca_v2.0_03266	Ca2_v2.0	3486091	3499498
XLOC_003433	Ca_v2.0_03313	Ca2_v2.0	4197448	4200710
XLOC_003434	Ca_v2.0_03316	Ca2_v2.0	4225949	4229311
XLOC_003438	Ca_v2.0_03321	Ca2_v2.0	4304781	4307474
XLOC_003446	Ca_v2.0_03335	Ca2_v2.0	4459164	4459675
XLOC_003447	Ca_v2.0_03336	Ca2_v2.0	4469701	4470382
XLOC_003450	Ca_v2.0_03341	Ca2_v2.0	4498965	4499800
XLOC_003458	Ca_v2.0_03358	Ca2_v2.0	4665294	4669191
XLOC_003459	Ca_v2.0_03359	Ca2_v2.0	4675162	4680774
XLOC_003460	Ca_v2.0_03360	Ca2_v2.0	4685292	4691563
XLOC_003465	Ca_v2.0_03370	Ca2_v2.0	4904708	4908628
XLOC_003468	Ca_v2.0_03374	Ca2_v2.0	4931296	4954745
XLOC_003486	Ca_v2.0_03397	Ca2_v2.0	5430629	5431029
XLOC_003490	Ca_v2.0_03409	Ca2_v2.0	5539782	5540481
XLOC_003499	Ca_v2.0_03432	Ca2_v2.0	5833063	5833923
XLOC_003510	Ca_v2.0_03448	Ca2_v2.0	5992618	5993707
XLOC_003513	Ca_v2.0_03453	Ca2_v2.0	6150756	6152631
XLOC_003514	Ca_v2.0_03456	Ca2_v2.0	6177552	6182236
XLOC_003521	Ca_v2.0_03463	Ca2_v2.0	6246365	6251678
XLOC_003527	Ca_v2.0_03480	Ca2_v2.0	6497841	6499840
XLOC_003533	Ca_v2.0_03492	Ca2_v2.0	6590599	6608553
XLOC_003536	-	Ca2_v2.0	6671293	6672555
XLOC_003554	Ca_v2.0_03530	Ca2_v2.0	7090634	7094754
XLOC_003557	Ca_v2.0_03534	Ca2_v2.0	7117258	7121850
XLOC_003560	Ca_v2.0_03537	Ca2_v2.0	7171481	7174873
XLOC_003565	Ca_v2.0_03546	Ca2_v2.0	7709133	7715423
XLOC_003570	Ca_v2.0_03557	Ca2_v2.0	7894356	7896513
XLOC_003577	Ca_v2.0_03567,Ca_v2.0_035(	Ca2_v2.0	8078950	8088191
XLOC_003585	Ca_v2.0_03584	Ca2_v2.0	8278497	8284862
XLOC_003590	Ca_v2.0_03598	Ca2_v2.0	8511860	8513959
XLOC_003591	Ca_v2.0_03600	Ca2_v2.0	8544390	8552374
XLOC_003602	Ca_v2.0_03616	Ca2_v2.0	8766084	8775913
XLOC_003618	-	Ca2_v2.0	9437162	9440069
XLOC_003619	Ca_v2.0_03653	Ca2_v2.0	9553054	9557446
XLOC_003620	Ca_v2.0_03662	Ca2_v2.0	9731240	9734097
XLOC_003621	Ca_v2.0_03663	Ca2_v2.0	9796015	9797118
XLOC_003631	Ca_v2.0_03686	Ca2_v2.0	10321678	10360032
XLOC_003632	Ca_v2.0_03687	Ca2_v2.0	10406527	10408523

For Review Only

XLOC_003637	Ca_v2.0_03701	Ca2_v2.0	10683418	10685986
XLOC_003638	Ca_v2.0_03703	Ca2_v2.0	10690603	10697307
XLOC_003646	Ca_v2.0_03718	Ca2_v2.0	11085543	11090888
XLOC_003655	Ca_v2.0_03738	Ca2_v2.0	11460233	11465800
XLOC_003657	Ca_v2.0_03746	Ca2_v2.0	11517257	11522423
XLOC_003663	Ca_v2.0_03754	Ca2_v2.0	11705138	11711574
XLOC_003665	Ca_v2.0_03757	Ca2_v2.0	11723084	11727352
XLOC_003667	Ca_v2.0_03760	Ca2_v2.0	11813183	11816344
XLOC_003672	Ca_v2.0_03772	Ca2_v2.0	12263719	12278887
XLOC_003673	Ca_v2.0_03773	Ca2_v2.0	12291204	12294823
XLOC_003679	Ca_v2.0_03784	Ca2_v2.0	12460453	12461501
XLOC_003680	Ca_v2.0_03785	Ca2_v2.0	12489854	12491887
XLOC_003688	Ca_v2.0_03798	Ca2_v2.0	12799291	12801021
XLOC_003689	Ca_v2.0_03799	Ca2_v2.0	12848678	12852487
XLOC_003690	Ca_v2.0_03800	Ca2_v2.0	12915258	12922165
XLOC_003694	-	Ca2_v2.0	13131983	13140913
XLOC_003697	Ca_v2.0_03807	Ca2_v2.0	13297989	13299535
XLOC_003708	Ca_v2.0_03833,Ca_v2.0_038:	Ca2_v2.0	14050983	14102688
XLOC_003710	Ca_v2.0_03840	Ca2_v2.0	14488668	14496692
XLOC_003715	Ca_v2.0_03847	Ca2_v2.0	14675756	14677987
XLOC_003716	Ca_v2.0_03848	Ca2_v2.0	14682074	14689499
XLOC_003720	Ca_v2.0_03854	Ca2_v2.0	14951967	14954875
XLOC_003721	Ca_v2.0_03855	Ca2_v2.0	14961456	14961982
XLOC_003723	Ca_v2.0_03858	Ca2_v2.0	15130089	15131005
XLOC_003727	-	Ca2_v2.0	15290958	15292194
XLOC_003734	Ca_v2.0_03874	Ca2_v2.0	15754390	15757384
XLOC_003736	Ca_v2.0_03876	Ca2_v2.0	15814684	15821061
XLOC_003737	Ca_v2.0_03879	Ca2_v2.0	15890558	15895331
XLOC_003741	Ca_v2.0_03896	Ca2_v2.0	16534082	16536929
XLOC_003751	Ca_v2.0_03913	Ca2_v2.0	17493878	17496199
XLOC_003753	Ca_v2.0_03918	Ca2_v2.0	17820598	17824487
XLOC_003754	Ca_v2.0_03920	Ca2_v2.0	17930565	17932657
XLOC_003763	Ca_v2.0_03933	Ca2_v2.0	18842919	18848179
XLOC_003771	Ca_v2.0_03958	Ca2_v2.0	20724482	20729197
XLOC_003775	Ca_v2.0_03967	Ca2_v2.0	22370721	22371932
XLOC_003776	Ca_v2.0_03968	Ca2_v2.0	22666649	22673273
XLOC_003783	-	Ca2_v2.0	23909522	23923840
XLOC_003790	Ca_v2.0_03990	Ca2_v2.0	25302723	25311675
XLOC_003797	Ca_v2.0_04000	Ca2_v2.0	26651214	26678776
XLOC_003798	Ca_v2.0_04001	Ca2_v2.0	26706399	26712817
XLOC_003807	Ca_v2.0_04014	Ca2_v2.0	28288707	28328800
XLOC_003808	Ca_v2.0_04016	Ca2_v2.0	28484765	28492741
XLOC_003809	Ca_v2.0_04018	Ca2_v2.0	28569383	28573947
XLOC_003813	Ca_v2.0_04022	Ca2_v2.0	29019552	29020210
XLOC_003817	Ca_v2.0_04030	Ca2_v2.0	29832985	29836737
XLOC_003820	-	Ca2_v2.0	30213276	30219566

For Review Only

XLOC_003823	Ca_v2.0_04039	Ca2_v2.0	30406581	30407805
XLOC_003825	Ca_v2.0_04042	Ca2_v2.0	30697351	30700178
XLOC_003826	-	Ca2_v2.0	30898806	30942220
XLOC_003827	Ca_v2.0_04046	Ca2_v2.0	31327760	31330798
XLOC_003840	Ca_v2.0_04077	Ca2_v2.0	33231104	33234466
XLOC_003841	Ca_v2.0_04079	Ca2_v2.0	33287190	33361212
XLOC_003845	-	Ca2_v2.0	33691365	33711279
XLOC_003846	-	Ca2_v2.0	33739697	33741082
XLOC_003847	-	Ca2_v2.0	33763195	33764165
XLOC_003854	Ca_v2.0_04097	Ca2_v2.0	34722233	34729992
XLOC_003873	Ca_v2.0_04138	Ca2_v2.0	36381791	36385258
XLOC_003880	Ca_v2.0_04154	Ca2_v2.0	36811468	36832558
XLOC_003890	Ca_v2.0_04168	Ca2_v2.0	37301835	37303305
XLOC_003898	Ca_v2.0_04190	Ca2_v2.0	37809581	37815425
XLOC_003899	Ca_v2.0_04191	Ca2_v2.0	37828463	37833788
XLOC_003906	Ca_v2.0_04204	Ca2_v2.0	38207074	38211949
XLOC_003917	Ca_v2.0_04220	Ca2_v2.0	38444372	38445271
XLOC_003921	Ca_v2.0_04226	Ca2_v2.0	38617207	38624682
XLOC_003925	Ca_v2.0_04232	Ca2_v2.0	38746404	38749660
XLOC_003929	Ca_v2.0_04240	Ca2_v2.0	39020668	39026325
XLOC_003940	Ca_v2.0_04260	Ca2_v2.0	39247080	39249000
XLOC_003952	Ca_v2.0_04277	Ca2_v2.0	39492873	39498599
XLOC_003960	Ca_v2.0_04293	Ca2_v2.0	39796864	39799931
XLOC_003969	Ca_v2.0_04309	Ca2_v2.0	40060594	40061756
XLOC_003971	Ca_v2.0_04312	Ca2_v2.0	40096767	40100766
XLOC_003975	Ca_v2.0_04316	Ca2_v2.0	40416019	40418682
XLOC_003976	Ca_v2.0_04317	Ca2_v2.0	40423250	40432171
XLOC_003979	Ca_v2.0_04321	Ca2_v2.0	40465626	40494104
XLOC_003988	Ca_v2.0_04335,Ca_v2.0_043:	Ca2_v2.0	40649539	40679928
XLOC_003994	Ca_v2.0_04347	Ca2_v2.0	40800390	40801324
XLOC_003995	Ca_v2.0_04354	Ca2_v2.0	40903727	40905354
XLOC_003997	Ca_v2.0_04356	Ca2_v2.0	40944365	40951222
XLOC_004006	Ca_v2.0_04366	Ca2_v2.0	41090264	41094244
XLOC_004007	Ca_v2.0_04367	Ca2_v2.0	41114407	41119854
XLOC_004008	Ca_v2.0_04370	Ca2_v2.0	41179669	41181957
XLOC_004010	Ca_v2.0_04376	Ca2_v2.0	41274307	41275808
XLOC_004012	Ca_v2.0_04387	Ca2_v2.0	41537306	41543190
XLOC_004018	Ca_v2.0_04397	Ca2_v2.0	41721895	41726532
XLOC_004020	Ca_v2.0_04402	Ca2_v2.0	41777601	41781068
XLOC_004021	-	Ca2_v2.0	41799507	41804172
XLOC_004023	Ca_v2.0_04411	Ca2_v2.0	41898879	41900381
XLOC_004025	Ca_v2.0_04413	Ca2_v2.0	41919676	41921389
XLOC_004034	Ca_v2.0_04433	Ca2_v2.0	42226435	42230272
XLOC_004039	Ca_v2.0_04445	Ca2_v2.0	42334633	42340085
XLOC_004042	Ca_v2.0_04450	Ca2_v2.0	42429945	42431671
XLOC_004044	Ca_v2.0_04454	Ca2_v2.0	42477811	42479060

For Review Only

XLOC_004046	Ca_v2.0_04456	Ca2_v2.0	42512589	42516035
XLOC_004047	Ca_v2.0_04457	Ca2_v2.0	42539689	42541718
XLOC_004049	Ca_v2.0_04461	Ca2_v2.0	42707055	42708142
XLOC_004052	Ca_v2.0_04468	Ca2_v2.0	42938603	42941284
XLOC_004053	Ca_v2.0_04471	Ca2_v2.0	43038164	43039942
XLOC_004054	-	Ca2_v2.0	43071156	43072313
XLOC_004055	Ca_v2.0_04474	Ca2_v2.0	43073726	43075973
XLOC_004075	Ca_v2.0_04513	Ca2_v2.0	43418416	43522629
XLOC_004076	Ca_v2.0_04515	Ca2_v2.0	43418416	43522629
XLOC_004079	Ca_v2.0_04523	Ca2_v2.0	43597680	43599628
XLOC_004080	Ca_v2.0_04525	Ca2_v2.0	43626194	43630174
XLOC_004082	Ca_v2.0_04528	Ca2_v2.0	43683797	43685685
XLOC_004087	Ca_v2.0_04533	Ca2_v2.0	43706450	43709554
XLOC_004095	Ca_v2.0_04556	Ca2_v2.0	43970249	43971937
XLOC_004107	Ca_v2.0_04576	Ca2_v2.0	44725920	44868394
XLOC_004112	Ca_v2.0_04586	Ca2_v2.0	45279132	45279680
XLOC_004118	-	Ca2_v2.0	45407874	45409648
XLOC_004124	Ca_v2.0_04603	Ca2_v2.0	45651726	45655725
XLOC_004127	Ca_v2.0_04607	Ca2_v2.0	45670276	45673779
XLOC_004130	Ca_v2.0_04614	Ca2_v2.0	45746000	45747858
XLOC_004138	Ca_v2.0_04638	Ca2_v2.0	46037912	46042848
XLOC_004141	Ca_v2.0_04647,Ca_v2.0_046	Ca2_v2.0	46171273	46174888
XLOC_004149	Ca_v2.0_04664	Ca2_v2.0	46378024	46382706
XLOC_004152	Ca_v2.0_04671	Ca2_v2.0	46439976	46444970
XLOC_004153	Ca_v2.0_04673	Ca2_v2.0	46457612	46465657
XLOC_004154	Ca_v2.0_04675	Ca2_v2.0	46492283	46496085
XLOC_004160	Ca_v2.0_04685	Ca2_v2.0	46609970	46613099
XLOC_004161	Ca_v2.0_04688	Ca2_v2.0	46629344	46633038
XLOC_004167	Ca_v2.0_04696	Ca2_v2.0	46691993	46693918
XLOC_004168	Ca_v2.0_04700	Ca2_v2.0	46731780	46735015
XLOC_004171	Ca_v2.0_04710	Ca2_v2.0	47645389	47647597
XLOC_004181	Ca_v2.0_04736	Ca2_v2.0	48066550	48070896
XLOC_004192	Ca_v2.0_04751	Ca2_v2.0	48294793	48297709
XLOC_004193	Ca_v2.0_04752	Ca2_v2.0	48299965	48305333
XLOC_004194	Ca_v2.0_04758	Ca2_v2.0	48378540	48380881
XLOC_004196	Ca_v2.0_04760	Ca2_v2.0	48388263	48392047
XLOC_004198	-	Ca2_v2.0	48462121	48465369
XLOC_004199	Ca_v2.0_04765	Ca2_v2.0	48467688	48472085
XLOC_004209	Ca_v2.0_04778	Ca2_v2.0	48694985	48695922
XLOC_004228	Ca_v2.0_04815	Ca2_v2.0	49092674	49095087
XLOC_004229	Ca_v2.0_04817	Ca2_v2.0	49102430	49103002
XLOC_004238	Ca_v2.0_04834	Ca2_v2.0	49225845	49228094
XLOC_004244	Ca_v2.0_04849	Ca2_v2.0	49361231	49447960
XLOC_004256	Ca_v2.0_04869	Ca2_v2.0	49655886	49673846
XLOC_004259	Ca_v2.0_04875	Ca2_v2.0	49741367	49743794
XLOC_004262	Ca_v2.0_04888	Ca2_v2.0	49857736	49860536

For Review Only

XLOC_004271	Ca_v2.0_04902	Ca2_v2.0	50173275	50175581
XLOC_004273	Ca_v2.0_04904	Ca2_v2.0	50185082	50188177
XLOC_004275	Ca_v2.0_04907	Ca2_v2.0	50213638	50216279
XLOC_004285	Ca_v2.0_04921	Ca2_v2.0	50337981	50342260
XLOC_004296	Ca_v2.0_04943	Ca2_v2.0	50492399	50501035
XLOC_004301	Ca_v2.0_04950	Ca2_v2.0	50601318	50605409
XLOC_004303	Ca_v2.0_04953	Ca2_v2.0	50650994	50654964
XLOC_004306	Ca_v2.0_04961	Ca2_v2.0	50681271	50685520
XLOC_004309	Ca_v2.0_04967	Ca2_v2.0	50772636	50775001
XLOC_004313	Ca_v2.0_04978	Ca2_v2.0	50880586	50892321
XLOC_004316	Ca_v2.0_04987	Ca2_v2.0	50949592	50953622
XLOC_004325	Ca_v2.0_05003	Ca2_v2.0	51091144	51091931
XLOC_004333	Ca_v2.0_05024	Ca2_v2.0	51243528	51247569
XLOC_004334	Ca_v2.0_05026	Ca2_v2.0	51250753	51255952
XLOC_004335	Ca_v2.0_05027	Ca2_v2.0	51260088	51265287
XLOC_004338	Ca_v2.0_05033	Ca2_v2.0	51297048	51300070
XLOC_004346	Ca_v2.0_05050	Ca2_v2.0	51519524	51520329
XLOC_004353	Ca_v2.0_05066	Ca2_v2.0	51614000	51616970
XLOC_004355	Ca_v2.0_05070	Ca2_v2.0	51629435	51631532
XLOC_004356	Ca_v2.0_05071,Ca_v2.0_050	Ca2_v2.0	51633613	51644206
XLOC_004358	Ca_v2.0_05074	Ca2_v2.0	51650759	51651857
XLOC_004362	Ca_v2.0_05081	Ca2_v2.0	51681279	51681806
XLOC_004373	Ca_v2.0_05098	Ca2_v2.0	51816376	51820416
XLOC_004374	Ca_v2.0_05099	Ca2_v2.0	51838188	51841915
XLOC_004376	Ca_v2.0_05105	Ca2_v2.0	51873739	51876151
XLOC_004384	Ca_v2.0_05118	Ca2_v2.0	52004630	52007740
XLOC_004385	Ca_v2.0_05119	Ca2_v2.0	52022963	52027930
XLOC_004386	-	Ca2_v2.0	52032955	52040377
XLOC_004392	Ca_v2.0_05132	Ca2_v2.0	52126115	52128583
XLOC_004395	Ca_v2.0_05138	Ca2_v2.0	52162837	52164568
XLOC_004407	Ca_v2.0_05161	Ca2_v2.0	52335672	52340675
XLOC_004431	Ca_v2.0_02944	Ca2_v2.0	119886	122797
XLOC_004437	Ca_v2.0_02958	Ca2_v2.0	240749	244215
XLOC_004444	Ca_v2.0_02975	Ca2_v2.0	371966	376833
XLOC_004447	Ca_v2.0_02979	Ca2_v2.0	420046	423183
XLOC_004451	Ca_v2.0_02987	Ca2_v2.0	483739	484826
XLOC_004457	Ca_v2.0_03006	Ca2_v2.0	629281	633231
XLOC_004458	Ca_v2.0_03007	Ca2_v2.0	637795	642658
XLOC_004475	Ca_v2.0_03041	Ca2_v2.0	1002566	1010018
XLOC_004486	Ca_v2.0_03070	Ca2_v2.0	1266594	1271690
XLOC_004488	Ca_v2.0_03074	Ca2_v2.0	1304305	1305813
XLOC_004491	Ca_v2.0_03077	Ca2_v2.0	1342978	1350534
XLOC_004492	Ca_v2.0_03078	Ca2_v2.0	1365792	1373107
XLOC_004494	Ca_v2.0_03080	Ca2_v2.0	1380966	1390414
XLOC_004495	Ca_v2.0_03082	Ca2_v2.0	1404526	1406898
XLOC_004502	Ca_v2.0_03095,Ca_v2.0_030	Ca2_v2.0	1510985	1533235

For Review Only

XLOC_004512	Ca_v2.0_03119	Ca2_v2.0	1739670	1743073
XLOC_004514	Ca_v2.0_03122	Ca2_v2.0	1802118	1811539
XLOC_004519	Ca_v2.0_03133	Ca2_v2.0	1988583	1990060
XLOC_004521	Ca_v2.0_03136	Ca2_v2.0	2025576	2027008
XLOC_004526	Ca_v2.0_03151	Ca2_v2.0	2179373	2181870
XLOC_004531	Ca_v2.0_03160	Ca2_v2.0	2268762	2275272
XLOC_004534	Ca_v2.0_03165	Ca2_v2.0	2297560	2299669
XLOC_004541	Ca_v2.0_03177,Ca_v2.0_031	Ca2_v2.0	2399624	2427066
XLOC_004561	-	Ca2_v2.0	2928850	2930661
XLOC_004562	Ca_v2.0_03220	Ca2_v2.0	2939742	2944258
XLOC_004563	Ca_v2.0_03223	Ca2_v2.0	2993838	3000861
XLOC_004565	Ca_v2.0_03229	Ca2_v2.0	3090566	3093485
XLOC_004566	Ca_v2.0_03233	Ca2_v2.0	3131970	3135424
XLOC_004567	Ca_v2.0_03235	Ca2_v2.0	3142879	3147319
XLOC_004572	Ca_v2.0_03249	Ca2_v2.0	3302575	3306441
XLOC_004574	Ca_v2.0_03256	Ca2_v2.0	3355273	3357206
XLOC_004575	Ca_v2.0_03257	Ca2_v2.0	3394758	3399338
XLOC_004580	Ca_v2.0_03266	Ca2_v2.0	3486091	3499498
XLOC_004582	-	Ca2_v2.0	3486091	3499498
XLOC_004597	Ca_v2.0_03308	Ca2_v2.0	4131884	4133565
XLOC_004611	Ca_v2.0_03340	Ca2_v2.0	4484604	4487989
XLOC_004621	Ca_v2.0_03361	Ca2_v2.0	4720778	4723528
XLOC_004628	-	Ca2_v2.0	4909202	4910823
XLOC_004630	Ca_v2.0_03372	Ca2_v2.0	4931296	4954745
XLOC_004634	Ca_v2.0_03392	Ca2_v2.0	5389540	5398225
XLOC_004646	Ca_v2.0_03414	Ca2_v2.0	5602575	5604596
XLOC_004647	Ca_v2.0_03416	Ca2_v2.0	5635802	5652520
XLOC_004661	Ca_v2.0_03445	Ca2_v2.0	5974465	5977614
XLOC_004666	Ca_v2.0_03455	Ca2_v2.0	6173330	6174737
XLOC_004667	Ca_v2.0_03463	Ca2_v2.0	6246365	6251678
XLOC_004668	Ca_v2.0_03466	Ca2_v2.0	6312356	6316751
XLOC_004675	Ca_v2.0_03476,Ca_v2.0_034	Ca2_v2.0	6439796	6446899
XLOC_004676	Ca_v2.0_03478	Ca2_v2.0	6451503	6460510
XLOC_004681	Ca_v2.0_03485	Ca2_v2.0	6548854	6551453
XLOC_004691	Ca_v2.0_03508	Ca2_v2.0	6826712	6840818
XLOC_004710	Ca_v2.0_03552,Ca_v2.0_035	Ca2_v2.0	7766180	7802056
XLOC_004711	Ca_v2.0_03554	Ca2_v2.0	7814298	7816258
XLOC_004721	Ca_v2.0_03571	Ca2_v2.0	8138993	8140205
XLOC_004727	Ca_v2.0_03578	Ca2_v2.0	8211157	8212557
XLOC_004730	Ca_v2.0_03585	Ca2_v2.0	8278497	8284862
XLOC_004739	Ca_v2.0_03599	Ca2_v2.0	8515301	8517106
XLOC_004740	Ca_v2.0_03604	Ca2_v2.0	8582663	8605934
XLOC_004745	Ca_v2.0_03618	Ca2_v2.0	8819909	8821518
XLOC_004746	Ca_v2.0_03619	Ca2_v2.0	8826128	8828009
XLOC_004747	Ca_v2.0_03620	Ca2_v2.0	8829747	8831693
XLOC_004748	Ca_v2.0_03622	Ca2_v2.0	8885350	8886616

For Review Only

For Review Only

XLOC_004758	Ca_v2.0_03640	Ca2_v2.0	9346162	9349742
XLOC_004764	Ca_v2.0_03652	Ca2_v2.0	9532204	9536011
XLOC_004767	Ca_v2.0_03656	Ca2_v2.0	9613909	9614073
XLOC_004768	Ca_v2.0_03657	Ca2_v2.0	9614310	9616344
XLOC_004771	Ca_v2.0_03660	Ca2_v2.0	9656727	9657895
XLOC_004789	Ca_v2.0_03692	Ca2_v2.0	10458490	10460339
XLOC_004794	Ca_v2.0_03698	Ca2_v2.0	10583938	10587219
XLOC_004798	Ca_v2.0_03704	Ca2_v2.0	10700151	10702004
XLOC_004812	Ca_v2.0_03732	Ca2_v2.0	11368619	11372100
XLOC_004825	Ca_v2.0_03751	Ca2_v2.0	11655853	11660852
XLOC_004828	Ca_v2.0_03759	Ca2_v2.0	11745908	11747296
XLOC_004833	-	Ca2_v2.0	11889660	11890632
XLOC_004837	-	Ca2_v2.0	12082191	12082587
XLOC_004842	Ca_v2.0_03781	Ca2_v2.0	12392861	12396504
XLOC_004845	Ca_v2.0_03788	Ca2_v2.0	12572210	12575650
XLOC_004852	Ca_v2.0_03804	Ca2_v2.0	13108866	13111645
XLOC_004860	Ca_v2.0_03815	Ca2_v2.0	13450772	13453862
XLOC_004863	Ca_v2.0_03822	Ca2_v2.0	13776858	13780503
XLOC_004875	Ca_v2.0_03840	Ca2_v2.0	14488668	14496692
XLOC_004877	Ca_v2.0_03846	Ca2_v2.0	14634101	14642010
XLOC_004889	Ca_v2.0_03876	Ca2_v2.0	15814684	15821061
XLOC_004893	Ca_v2.0_03881	Ca2_v2.0	16073425	16075420
XLOC_004896	Ca_v2.0_03886	Ca2_v2.0	16259211	16259836
XLOC_004913	Ca_v2.0_03913	Ca2_v2.0	17493878	17496199
XLOC_004915	Ca_v2.0_03915	Ca2_v2.0	17586350	17588009
XLOC_004920	Ca_v2.0_03926	Ca2_v2.0	18353140	18354335
XLOC_004924	Ca_v2.0_03933	Ca2_v2.0	18842919	18848179
XLOC_004950	-	Ca2_v2.0	22890330	22891336
XLOC_004959	Ca_v2.0_03986	Ca2_v2.0	24818757	24821453
XLOC_004965	Ca_v2.0_04002	Ca2_v2.0	26841097	26842168
XLOC_004968	Ca_v2.0_04012	Ca2_v2.0	28201049	28203535
XLOC_004969	Ca_v2.0_04014,Ca_v2.0_040	Ca2_v2.0	28288707	28328800
XLOC_004970	Ca_v2.0_04016,Ca_v2.0_040	Ca2_v2.0	28484765	28492741
XLOC_004973	Ca_v2.0_04028	Ca2_v2.0	29759984	29761049
XLOC_004986	Ca_v2.0_04051	Ca2_v2.0	31481249	31481801
XLOC_005001	Ca_v2.0_04078,Ca_v2.0_040	Ca2_v2.0	33287190	33361212
XLOC_005016	Ca_v2.0_04101	Ca2_v2.0	34824502	34830744
XLOC_005019	Ca_v2.0_04107,Ca_v2.0_041	Ca2_v2.0	35063540	35067444
XLOC_005022	Ca_v2.0_04113	Ca2_v2.0	35272195	35273857
XLOC_005032	Ca_v2.0_04132	Ca2_v2.0	36169821	36174515
XLOC_005033	Ca_v2.0_04133	Ca2_v2.0	36182744	36184419
XLOC_005036	Ca_v2.0_04137	Ca2_v2.0	36339117	36349318
XLOC_005037	Ca_v2.0_04138	Ca2_v2.0	36381791	36385258
XLOC_005049	Ca_v2.0_04165	Ca2_v2.0	37136163	37137926
XLOC_005050	Ca_v2.0_04167	Ca2_v2.0	37258957	37264808
XLOC_005056	Ca_v2.0_04175	Ca2_v2.0	37522313	37524544

XLOC_005064	Ca_v2.0_04189	Ca2_v2.0	37773534	37778504
XLOC_005074	Ca_v2.0_04204	Ca2_v2.0	38207074	38211949
XLOC_005098	Ca_v2.0_04259	Ca2_v2.0	39233982	39235168
XLOC_005100	Ca_v2.0_04262	Ca2_v2.0	39269877	39273334
XLOC_005105	Ca_v2.0_04271	Ca2_v2.0	39345207	39357972
XLOC_005115	Ca_v2.0_04292	Ca2_v2.0	39785879	39789286
XLOC_005118	Ca_v2.0_04303	Ca2_v2.0	39978054	39980999
XLOC_005127	Ca_v2.0_04321	Ca2_v2.0	40465626	40494104
XLOC_005131	Ca_v2.0_04329	Ca2_v2.0	40600317	40601948
XLOC_005136	Ca_v2.0_04346	Ca2_v2.0	40777926	40780652
XLOC_005138	Ca_v2.0_04349	Ca2_v2.0	40831253	40835113
XLOC_005140	Ca_v2.0_04351	Ca2_v2.0	40861029	40861832
XLOC_005142	Ca_v2.0_04353	Ca2_v2.0	40888224	40889845
XLOC_005146	Ca_v2.0_04365	Ca2_v2.0	41084950	41087855
XLOC_005148	Ca_v2.0_04368	Ca2_v2.0	41128549	41130520
XLOC_005152	Ca_v2.0_04373	Ca2_v2.0	41225420	41230558
XLOC_005153	Ca_v2.0_04375	Ca2_v2.0	41267761	41270954
XLOC_005156	Ca_v2.0_04379	Ca2_v2.0	41333179	41335702
XLOC_005160	Ca_v2.0_04383	Ca2_v2.0	41366966	41369786
XLOC_005164	Ca_v2.0_04391	Ca2_v2.0	41622089	41626014
XLOC_005170	Ca_v2.0_04398	Ca2_v2.0	41746108	41754097
XLOC_005172	Ca_v2.0_04400	Ca2_v2.0	41763306	41767150
XLOC_005174	Ca_v2.0_04402	Ca2_v2.0	41777601	41781068
XLOC_005182	Ca_v2.0_04411	Ca2_v2.0	41898879	41900381
XLOC_005194	Ca_v2.0_04432	Ca2_v2.0	42214164	42218615
XLOC_005200	Ca_v2.0_04442	Ca2_v2.0	42288849	42292875
XLOC_005225	Ca_v2.0_04481	Ca2_v2.0	43154055	43155300
XLOC_005231	Ca_v2.0_04492	Ca2_v2.0	43251990	43254804
XLOC_005235	Ca_v2.0_04503	Ca2_v2.0	43390440	43392559
XLOC_005236	Ca_v2.0_04505	Ca2_v2.0	43412428	43417995
XLOC_005241	Ca_v2.0_04514	Ca2_v2.0	43418416	43522629
XLOC_005255	Ca_v2.0_04541	Ca2_v2.0	43866832	43869450
XLOC_005258	Ca_v2.0_04544	Ca2_v2.0	43881023	43884120
XLOC_005259	Ca_v2.0_04545,Ca_v2.0_045	Ca2_v2.0	43886716	43891589
XLOC_005267	-	Ca2_v2.0	44225956	44227403
XLOC_005274	Ca_v2.0_04574	Ca2_v2.0	44725920	44868394
XLOC_005282	Ca_v2.0_04590	Ca2_v2.0	45312776	45315730
XLOC_005283	-	Ca2_v2.0	45413754	45417512
XLOC_005286	Ca_v2.0_04597	Ca2_v2.0	45547566	45548524
XLOC_005288	Ca_v2.0_04599	Ca2_v2.0	45567096	45570055
XLOC_005291	Ca_v2.0_04605	Ca2_v2.0	45661460	45664295
XLOC_005294	Ca_v2.0_04612	Ca2_v2.0	45719334	45722649
XLOC_005296	Ca_v2.0_04615	Ca2_v2.0	45756372	45757528
XLOC_005301	Ca_v2.0_04621	Ca2_v2.0	45816666	45819305
XLOC_005302	Ca_v2.0_04622	Ca2_v2.0	45823839	45826077
XLOC_005304	Ca_v2.0_04627	Ca2_v2.0	45883857	45891261

For Review Only

XLOC_005311	Ca_v2.0_04636	Ca2_v2.0	46011825	46016267
XLOC_005312	Ca_v2.0_04637	Ca2_v2.0	46016390	46022342
XLOC_005315	Ca_v2.0_04643	Ca2_v2.0	46063864	46070301
XLOC_005318	Ca_v2.0_04646	Ca2_v2.0	46149043	46152920
XLOC_005330	Ca_v2.0_04667	Ca2_v2.0	46397401	46400408
XLOC_005333	Ca_v2.0_04672	Ca2_v2.0	46450331	46451716
XLOC_005335	Ca_v2.0_04674	Ca2_v2.0	46471753	46474042
XLOC_005340	Ca_v2.0_04684	Ca2_v2.0	46607966	46608862
XLOC_005344	Ca_v2.0_04694	Ca2_v2.0	46682430	46683602
XLOC_005346	Ca_v2.0_04697	Ca2_v2.0	46696668	46714409
XLOC_005349	Ca_v2.0_04703	Ca2_v2.0	47002012	47005441
XLOC_005352	-	Ca2_v2.0	47319873	47321522
XLOC_005357	Ca_v2.0_04711	Ca2_v2.0	47671486	47672046
XLOC_005362	Ca_v2.0_04717	Ca2_v2.0	47778668	47781073
XLOC_005363	-	Ca2_v2.0	47845650	47847411
XLOC_005376	Ca_v2.0_04741	Ca2_v2.0	48151097	48157118
XLOC_005382	Ca_v2.0_04753	Ca2_v2.0	48299965	48305333
XLOC_005386	Ca_v2.0_04757	Ca2_v2.0	48348645	48353299
XLOC_005390	Ca_v2.0_04765	Ca2_v2.0	48467688	48472085
XLOC_005402	Ca_v2.0_04797	Ca2_v2.0	48897012	48898843
XLOC_005409	Ca_v2.0_04808	Ca2_v2.0	49012904	49013229
XLOC_005412	Ca_v2.0_04814	Ca2_v2.0	49064741	49071222
XLOC_005416	Ca_v2.0_04826	Ca2_v2.0	49155435	49160236
XLOC_005428	Ca_v2.0_04847	Ca2_v2.0	49361231	49447960
XLOC_005439	Ca_v2.0_04870	Ca2_v2.0	49684301	49685146
XLOC_005441	-	Ca2_v2.0	49734351	49735302
XLOC_005455	Ca_v2.0_04893	Ca2_v2.0	49899994	49986710
XLOC_005467	Ca_v2.0_04925	Ca2_v2.0	50351341	50358748
XLOC_005476	Ca_v2.0_04943	Ca2_v2.0	50492399	50501035
XLOC_005486	Ca_v2.0_04962	Ca2_v2.0	50685874	50688504
XLOC_005487	Ca_v2.0_04963	Ca2_v2.0	50691002	50692735
XLOC_005501	Ca_v2.0_04986	Ca2_v2.0	50936308	50942724
XLOC_005502	Ca_v2.0_04988	Ca2_v2.0	50955179	50968679
XLOC_005511	Ca_v2.0_05006	Ca2_v2.0	51127373	51129775
XLOC_005517	Ca_v2.0_05017	Ca2_v2.0	51201162	51208448
XLOC_005519	Ca_v2.0_05019	Ca2_v2.0	51217136	51220426
XLOC_005521	Ca_v2.0_05021	Ca2_v2.0	51224936	51226256
XLOC_005527	Ca_v2.0_05031,Ca_v2.0_050:	Ca2_v2.0	51283214	51289090
XLOC_005528	Ca_v2.0_05033	Ca2_v2.0	51297048	51300070
XLOC_005533	Ca_v2.0_05039	Ca2_v2.0	51439873	51442386
XLOC_005534	Ca_v2.0_05043	Ca2_v2.0	51470998	51474270
XLOC_005536	Ca_v2.0_05046	Ca2_v2.0	51493918	51495013
XLOC_005558	Ca_v2.0_05099	Ca2_v2.0	51838188	51841915
XLOC_005565	Ca_v2.0_05113	Ca2_v2.0	51944754	51946022
XLOC_005574	Ca_v2.0_05131	Ca2_v2.0	52118991	52123268
XLOC_005577	Ca_v2.0_05135	Ca2_v2.0	52131962	52147342

For Review Only

XLOC_005578	Ca_v2.0_05135	Ca2_v2.0	52131962	52147342
XLOC_005580	Ca_v2.0_05140,Ca_v2.0_051	Ca2_v2.0	52175382	52184012
XLOC_005581	Ca_v2.0_05145	Ca2_v2.0	52213432	52214492
XLOC_005587	-	Ca2_v2.0	52280400	52281808
XLOC_005590	Ca_v2.0_05160	Ca2_v2.0	52326726	52332063
XLOC_005592	Ca_v2.0_05162	Ca2_v2.0	52342439	52344313
XLOC_005595	Ca_v2.0_05167	Ca2_v2.0	52368573	52369468
XLOC_005600	Ca_v2.0_05173	Ca2_v2.0	52423110	52424684
XLOC_005603	Ca_v2.0_05178	Ca2_v2.0	52445353	52448316
XLOC_005605	Ca_v2.0_05179	Ca2_v2.0	52450759	52452369
XLOC_005611	Ca_v2.0_05191	Ca2_v2.0	52581467	52581850
XLOC_005615	-	Ca2_v2.0	190914	191756
XLOC_005618	-	Ca2_v2.0	1448282	1449673
XLOC_005620	-	Ca2_v2.0	2080835	2081519
XLOC_005624	-	Ca2_v2.0	4736446	4736823
XLOC_005628	-	Ca2_v2.0	6161156	6161779
XLOC_005629	-	Ca2_v2.0	6170120	6171046
XLOC_005632	-	Ca2_v2.0	6853922	6854241
XLOC_005642	-	Ca2_v2.0	12735832	12736047
XLOC_005643	-	Ca2_v2.0	13131983	13140913
XLOC_005644	-	Ca2_v2.0	13742737	13743358
XLOC_005649	-	Ca2_v2.0	15995924	15997176
XLOC_005662	-	Ca2_v2.0	30167871	30168620
XLOC_005664	-	Ca2_v2.0	31733498	31733906
XLOC_005668	-	Ca2_v2.0	34231981	34233083
XLOC_005674	-	Ca2_v2.0	38761807	38762475
XLOC_005676	-	Ca2_v2.0	39695019	39696812
XLOC_005686	-	Ca2_v2.0	43003794	43003990
XLOC_005687	-	Ca2_v2.0	43029722	43030197
XLOC_005692	-	Ca2_v2.0	44072703	44073161
XLOC_005698	-	Ca2_v2.0	48083234	48083834
XLOC_005699	-	Ca2_v2.0	48448780	48450113
XLOC_005702	-	Ca2_v2.0	49574157	49575010
XLOC_005711	-	Ca3_v2.0	266323	267273
XLOC_005714	Ca_v2.0_05214	Ca3_v2.0	429453	431217
XLOC_005715	Ca_v2.0_05215	Ca3_v2.0	448169	450707
XLOC_005728	Ca_v2.0_05247	Ca3_v2.0	1201937	1211432
XLOC_005732	Ca_v2.0_05254	Ca3_v2.0	1415223	1419226
XLOC_005735	Ca_v2.0_05259	Ca3_v2.0	1494597	1500351
XLOC_005736	Ca_v2.0_05260	Ca3_v2.0	1539196	1542205
XLOC_005738	Ca_v2.0_05262	Ca3_v2.0	1637691	1639457
XLOC_005739	Ca_v2.0_05263	Ca3_v2.0	1647236	1649004
XLOC_005760	Ca_v2.0_05303	Ca3_v2.0	2228423	2235841
XLOC_005765	Ca_v2.0_05314	Ca3_v2.0	2497378	2500001
XLOC_005771	Ca_v2.0_05325	Ca3_v2.0	2785571	2787816
XLOC_005774	Ca_v2.0_05333,Ca_v2.0_053	Ca3_v2.0	2977145	2986424

For Review Only

XLOC_005777	Ca_v2.0_05345	Ca3_v2.0	3288916	3291880
XLOC_005781	Ca_v2.0_05352	Ca3_v2.0	3370313	3373273
XLOC_005786	Ca_v2.0_05358	Ca3_v2.0	3587340	3591908
XLOC_005793	Ca_v2.0_05381	Ca3_v2.0	4081135	4131655
XLOC_005805	Ca_v2.0_05423	Ca3_v2.0	5018365	5020375
XLOC_005806	Ca_v2.0_05424	Ca3_v2.0	5082491	5084524
XLOC_005813	Ca_v2.0_05441	Ca3_v2.0	5765062	5767089
XLOC_005815	Ca_v2.0_05443	Ca3_v2.0	5845891	5850203
XLOC_005819	Ca_v2.0_05450	Ca3_v2.0	6064455	6068583
XLOC_005849	Ca_v2.0_05503	Ca3_v2.0	7016686	7021929
XLOC_005862	Ca_v2.0_05527	Ca3_v2.0	7832347	7840297
XLOC_005869	Ca_v2.0_05538	Ca3_v2.0	8207829	8212719
XLOC_005873	Ca_v2.0_05544	Ca3_v2.0	8362795	8367773
XLOC_005893	Ca_v2.0_05598	Ca3_v2.0	17440523	17443737
XLOC_005894	Ca_v2.0_05599	Ca3_v2.0	17505334	17509557
XLOC_005929	Ca_v2.0_05664	Ca3_v2.0	19872437	19875548
XLOC_005935	Ca_v2.0_05677	Ca3_v2.0	20522487	20524722
XLOC_005936	Ca_v2.0_05678	Ca3_v2.0	20551080	20560247
XLOC_005937	Ca_v2.0_05691	Ca3_v2.0	21002922	21007133
XLOC_005940	Ca_v2.0_05697	Ca3_v2.0	21114840	21118268
XLOC_005945	Ca_v2.0_05710	Ca3_v2.0	21692811	21698164
XLOC_005952	Ca_v2.0_05720	Ca3_v2.0	22505655	22505999
XLOC_005973	Ca_v2.0_05766	Ca3_v2.0	26637845	26644422
XLOC_005980	-	Ca3_v2.0	27441633	27442891
XLOC_005984	Ca_v2.0_05780	Ca3_v2.0	27562938	27563571
XLOC_005985	-	Ca3_v2.0	27684925	27685555
XLOC_005995	Ca_v2.0_05794	Ca3_v2.0	29126283	29131382
XLOC_005999	Ca_v2.0_05801	Ca3_v2.0	29915541	29916537
XLOC_006000	Ca_v2.0_05803	Ca3_v2.0	29971569	29972511
XLOC_006001	Ca_v2.0_05806	Ca3_v2.0	30336901	30337384
XLOC_006011	Ca_v2.0_05821	Ca3_v2.0	31294573	31303822
XLOC_006017	Ca_v2.0_05838	Ca3_v2.0	33035196	33053194
XLOC_006034	Ca_v2.0_05879	Ca3_v2.0	34612744	34621012
XLOC_006035	Ca_v2.0_05884	Ca3_v2.0	35241818	35242259
XLOC_006038	Ca_v2.0_05891	Ca3_v2.0	35987199	35993239
XLOC_006064	Ca_v2.0_05930	Ca3_v2.0	38731831	38736090
XLOC_006076	Ca_v2.0_05948	Ca3_v2.0	39615393	39616473
XLOC_006093	Ca_v2.0_05987	Ca3_v2.0	41681816	41684938
XLOC_006098	-	Ca3_v2.0	43005314	43008452
XLOC_006103	Ca_v2.0_06009	Ca3_v2.0	43636624	43638185
XLOC_006109	Ca_v2.0_06016,Ca_v2.0_060	Ca3_v2.0	44069469	44114337
XLOC_006111	Ca_v2.0_06025	Ca3_v2.0	44437607	44477449
XLOC_006113	-	Ca3_v2.0	44437607	44477449
XLOC_006117	Ca_v2.0_06033	Ca3_v2.0	44772816	44774472
XLOC_006127	Ca_v2.0_06053	Ca3_v2.0	45833248	45835511
XLOC_006149	Ca_v2.0_06104	Ca3_v2.0	48155337	48156385

For Review Only

XLOC_006151	-	Ca3_v2.0	48297695	48300462
XLOC_006157	Ca_v2.0_06120	Ca3_v2.0	48669702	48670664
XLOC_006161	Ca_v2.0_06126	Ca3_v2.0	48790395	48794272
XLOC_006176	Ca_v2.0_06155	Ca3_v2.0	49529836	49532309
XLOC_006177	Ca_v2.0_06156	Ca3_v2.0	49564100	49570538
XLOC_006180	Ca_v2.0_06160	Ca3_v2.0	49695609	49699451
XLOC_006190	-	Ca3_v2.0	50048313	50050203
XLOC_006200	Ca_v2.0_06200	Ca3_v2.0	50444036	50446996
XLOC_006207	Ca_v2.0_06216	Ca3_v2.0	50833824	50836360
XLOC_006221	Ca_v2.0_06240	Ca3_v2.0	51385402	51386134
XLOC_006227	Ca_v2.0_06251	Ca3_v2.0	51630976	51633760
XLOC_006229	Ca_v2.0_06253	Ca3_v2.0	51673313	51676174
XLOC_006234	Ca_v2.0_06260	Ca3_v2.0	51888878	51893827
XLOC_006236	Ca_v2.0_06262	Ca3_v2.0	51922831	51928959
XLOC_006247	Ca_v2.0_06280	Ca3_v2.0	52351963	52358649
XLOC_006273	Ca_v2.0_06333	Ca3_v2.0	53670273	53674803
XLOC_006274	Ca_v2.0_06339	Ca3_v2.0	53875278	53880566
XLOC_006296	Ca_v2.0_06392	Ca3_v2.0	55629367	55632114
XLOC_006301	Ca_v2.0_06398	Ca3_v2.0	55703404	55707761
XLOC_006302	Ca_v2.0_06400	Ca3_v2.0	55784408	55787345
XLOC_006305	Ca_v2.0_06407	Ca3_v2.0	55875468	55879848
XLOC_006307	Ca_v2.0_06410	Ca3_v2.0	55959962	55964235
XLOC_006311	Ca_v2.0_06416	Ca3_v2.0	56047642	56050904
XLOC_006312	Ca_v2.0_06419	Ca3_v2.0	56156246	56178290
XLOC_006316	Ca_v2.0_06426	Ca3_v2.0	56245864	56250433
XLOC_006327	-	Ca3_v2.0	56655429	56683149
XLOC_006336	Ca_v2.0_06456	Ca3_v2.0	56983837	57003239
XLOC_006341	Ca_v2.0_06473	Ca3_v2.0	57264751	57276801
XLOC_006342	Ca_v2.0_06477	Ca3_v2.0	57424963	57428536
XLOC_006347	Ca_v2.0_06483	Ca3_v2.0	57587589	57593297
XLOC_006348	Ca_v2.0_06485	Ca3_v2.0	57653025	57654830
XLOC_006363	Ca_v2.0_06513	Ca3_v2.0	58072160	58074123
XLOC_006368	Ca_v2.0_06521	Ca3_v2.0	58133628	58137734
XLOC_006370	Ca_v2.0_06524	Ca3_v2.0	58187343	58191076
XLOC_006378	Ca_v2.0_06535	Ca3_v2.0	58328413	58340688
XLOC_006379	Ca_v2.0_06536	Ca3_v2.0	58364695	58365649
XLOC_006384	Ca_v2.0_06544	Ca3_v2.0	58498374	58503134
XLOC_006386	Ca_v2.0_06546	Ca3_v2.0	58512362	58533301
XLOC_006393	Ca_v2.0_06560	Ca3_v2.0	58643510	58644744
XLOC_006395	Ca_v2.0_06562	Ca3_v2.0	58704283	58705239
XLOC_006396	-	Ca3_v2.0	58795449	58798894
XLOC_006401	Ca_v2.0_06575	Ca3_v2.0	58986558	58989119
XLOC_006402	Ca_v2.0_06576	Ca3_v2.0	59000690	59002756
XLOC_006403	Ca_v2.0_06577	Ca3_v2.0	59005383	59013795
XLOC_006406	Ca_v2.0_06582	Ca3_v2.0	59078339	59083938
XLOC_006410	Ca_v2.0_06588	Ca3_v2.0	59169381	59170845

For Review Only

XLOC_006413	-	Ca3_v2.0	59224400	59226443
XLOC_006414	Ca_v2.0_06593	Ca3_v2.0	59234561	59235848
XLOC_006416	Ca_v2.0_06596	Ca3_v2.0	59253930	59255539
XLOC_006419	Ca_v2.0_06602	Ca3_v2.0	59300401	59310409
XLOC_006421	Ca_v2.0_06604	Ca3_v2.0	59328225	59334361
XLOC_006427	Ca_v2.0_06616	Ca3_v2.0	59504218	59507733
XLOC_006431	Ca_v2.0_06625	Ca3_v2.0	59583299	59591250
XLOC_006443	Ca_v2.0_06648	Ca3_v2.0	59811579	59815223
XLOC_006445	-	Ca3_v2.0	59859700	59861965
XLOC_006446	-	Ca3_v2.0	59868475	59882303
XLOC_006458	Ca_v2.0_06676	Ca3_v2.0	60265163	60277542
XLOC_006459	Ca_v2.0_06677	Ca3_v2.0	60281346	60284781
XLOC_006463	Ca_v2.0_06684	Ca3_v2.0	60388189	60399129
XLOC_006468	Ca_v2.0_06697	Ca3_v2.0	60529199	60554297
XLOC_006476	Ca_v2.0_06711	Ca3_v2.0	60695643	60705134
XLOC_006481	Ca_v2.0_06720	Ca3_v2.0	60761893	60769689
XLOC_006486	Ca_v2.0_06733	Ca3_v2.0	60900636	60905426
XLOC_006492	Ca_v2.0_06743	Ca3_v2.0	61010514	61020004
XLOC_006493	Ca_v2.0_06746	Ca3_v2.0	61053190	61054747
XLOC_006496	Ca_v2.0_06752	Ca3_v2.0	61161907	61167257
XLOC_006501	Ca_v2.0_06760	Ca3_v2.0	61270865	61276495
XLOC_006502	Ca_v2.0_06763	Ca3_v2.0	61294097	61297887
XLOC_006508	Ca_v2.0_06776	Ca3_v2.0	61488812	61495362
XLOC_006509	Ca_v2.0_06782	Ca3_v2.0	61546628	61556649
XLOC_006510	Ca_v2.0_06783	Ca3_v2.0	61573048	61587024
XLOC_006511	Ca_v2.0_06785	Ca3_v2.0	61618138	61621088
XLOC_006512	Ca_v2.0_06787	Ca3_v2.0	61660138	61661933
XLOC_006517	Ca_v2.0_06792	Ca3_v2.0	61686898	61689080
XLOC_006525	Ca_v2.0_06810	Ca3_v2.0	61856579	61862302
XLOC_006530	Ca_v2.0_06821	Ca3_v2.0	61954363	61958067
XLOC_006532	Ca_v2.0_06823	Ca3_v2.0	61965742	61970359
XLOC_006537	Ca_v2.0_06833	Ca3_v2.0	62076279	62080691
XLOC_006544	Ca_v2.0_06848	Ca3_v2.0	62196296	62203021
XLOC_006550	-	Ca3_v2.0	62345993	62347699
XLOC_006552	Ca_v2.0_06864	Ca3_v2.0	62372090	62375328
XLOC_006558	Ca_v2.0_06872	Ca3_v2.0	62453961	62458159
XLOC_006565	Ca_v2.0_06895	Ca3_v2.0	62696673	62704216
XLOC_006566	-	Ca3_v2.0	62730697	62732339
XLOC_006572	-	Ca3_v2.0	62979378	62981868
XLOC_006573	Ca_v2.0_06909	Ca3_v2.0	62989471	63006985
XLOC_006584	Ca_v2.0_06939	Ca3_v2.0	63217208	63221987
XLOC_006588	Ca_v2.0_06945,Ca_v2.0_069	Ca3_v2.0	63302271	63313007
XLOC_006595	Ca_v2.0_06966	Ca3_v2.0	63564941	63566908
XLOC_006623	Ca_v2.0_07021	Ca3_v2.0	64306658	64315284
XLOC_006635	Ca_v2.0_07038	Ca3_v2.0	64514895	64518658
XLOC_006636	Ca_v2.0_07039	Ca3_v2.0	64543838	64546219

For Review Only

XLOC_006638	Ca_v2.0_07041	Ca3_v2.0	64580610	64582735
XLOC_006647	Ca_v2.0_07056	Ca3_v2.0	64710266	64718106
XLOC_006651	Ca_v2.0_07067	Ca3_v2.0	64730104	64813491
XLOC_006653	Ca_v2.0_07070	Ca3_v2.0	64852503	64864073
XLOC_006661	Ca_v2.0_07091	Ca3_v2.0	65018424	65021779
XLOC_006664	Ca_v2.0_07095	Ca3_v2.0	65057640	65062742
XLOC_006671	Ca_v2.0_07102	Ca3_v2.0	65122763	65139331
XLOC_006681	Ca_v2.0_07119	Ca3_v2.0	65276178	65276919
XLOC_006688	Ca_v2.0_07130	Ca3_v2.0	65378502	65381741
XLOC_006694	Ca_v2.0_07140	Ca3_v2.0	65455557	65459663
XLOC_006708	Ca_v2.0_07173	Ca3_v2.0	65799816	65863674
XLOC_006711	Ca_v2.0_07178	Ca3_v2.0	65799816	65863674
XLOC_006722	Ca_v2.0_07215	Ca3_v2.0	66325835	66328949
XLOC_006723	Ca_v2.0_07217	Ca3_v2.0	66346376	66349575
XLOC_006730	Ca_v2.0_07227	Ca3_v2.0	66480704	66485761
XLOC_006733	Ca_v2.0_07232	Ca3_v2.0	66534310	66535647
XLOC_006736	Ca_v2.0_07235	Ca3_v2.0	66547380	66596414
XLOC_006749	Ca_v2.0_07252	Ca3_v2.0	66762501	66764530
XLOC_006750	Ca_v2.0_07255	Ca3_v2.0	66821218	66826450
XLOC_006752	Ca_v2.0_07257	Ca3_v2.0	66830442	66834983
XLOC_006753	Ca_v2.0_07258	Ca3_v2.0	66838264	66839033
XLOC_006754	Ca_v2.0_07261	Ca3_v2.0	66875109	66876930
XLOC_006756	-	Ca3_v2.0	66882207	66912171
XLOC_006771	Ca_v2.0_07288	Ca3_v2.0	67118051	67120871
XLOC_006773	Ca_v2.0_07291	Ca3_v2.0	67150692	67153102
XLOC_006774	Ca_v2.0_07295	Ca3_v2.0	67213437	67215427
XLOC_006775	Ca_v2.0_07301	Ca3_v2.0	67276122	67300723
XLOC_006779	Ca_v2.0_07306	Ca3_v2.0	67325535	67329592
XLOC_006780	-	Ca3_v2.0	67338006	67343595
XLOC_006782	Ca_v2.0_07316	Ca3_v2.0	67428829	67432189
XLOC_006785	Ca_v2.0_07321	Ca3_v2.0	67483152	67483757
XLOC_006789	Ca_v2.0_07327	Ca3_v2.0	67526348	67528983
XLOC_006795	Ca_v2.0_07340	Ca3_v2.0	67652740	67655231
XLOC_006796	Ca_v2.0_07341	Ca3_v2.0	67665339	67666927
XLOC_006797	Ca_v2.0_07342	Ca3_v2.0	67682224	67684717
XLOC_006818	Ca_v2.0_07384	Ca3_v2.0	67969591	67971433
XLOC_006829	Ca_v2.0_07406	Ca3_v2.0	68167208	68169569
XLOC_006838	Ca_v2.0_07422	Ca3_v2.0	68249017	68253844
XLOC_006864	Ca_v2.0_07477	Ca3_v2.0	68794036	68798201
XLOC_006865	Ca_v2.0_07480	Ca3_v2.0	68813721	68815417
XLOC_006866	Ca_v2.0_07481	Ca3_v2.0	68821141	68821501
XLOC_006871	Ca_v2.0_07488	Ca3_v2.0	68876298	68880194
XLOC_006874	Ca_v2.0_07494	Ca3_v2.0	68933729	68940852
XLOC_006876	Ca_v2.0_07500	Ca3_v2.0	68997027	68999171
XLOC_006878	Ca_v2.0_07503	Ca3_v2.0	69041218	69050892
XLOC_006882	Ca_v2.0_07510	Ca3_v2.0	69100860	69103558

For Review Only

XLOC_006885	Ca_v2.0_07517	Ca3_v2.0	69155053	69157891
XLOC_006887	Ca_v2.0_07520	Ca3_v2.0	69175288	69178037
XLOC_006888	Ca_v2.0_07521	Ca3_v2.0	69184111	69186701
XLOC_006892	Ca_v2.0_07530	Ca3_v2.0	69229114	69231208
XLOC_006894	Ca_v2.0_07536	Ca3_v2.0	69306827	69314186
XLOC_006907	Ca_v2.0_07560	Ca3_v2.0	69523866	69532346
XLOC_006915	Ca_v2.0_07575	Ca3_v2.0	69694317	69694772
XLOC_006927	Ca_v2.0_07590	Ca3_v2.0	69863262	69868786
XLOC_006929	Ca_v2.0_07595	Ca3_v2.0	69890250	69900198
XLOC_006932	Ca_v2.0_07599	Ca3_v2.0	69948156	69950375
XLOC_006937	Ca_v2.0_07614	Ca3_v2.0	70071532	70079089
XLOC_006938	Ca_v2.0_07619	Ca3_v2.0	70105134	70106736
XLOC_006941	Ca_v2.0_07623	Ca3_v2.0	70139998	70143347
XLOC_006946	Ca_v2.0_07635	Ca3_v2.0	70233260	70236711
XLOC_006947	Ca_v2.0_07639	Ca3_v2.0	70253605	70258090
XLOC_006957	Ca_v2.0_07660	Ca3_v2.0	70418228	70420377
XLOC_006963	Ca_v2.0_07672	Ca3_v2.0	70513035	70515475
XLOC_006964	Ca_v2.0_07675	Ca3_v2.0	70547039	70547975
XLOC_006967	Ca_v2.0_07679	Ca3_v2.0	70615305	70638700
XLOC_006973	Ca_v2.0_07691	Ca3_v2.0	70751464	70754662
XLOC_006986	Ca_v2.0_07706	Ca3_v2.0	71005711	71007480
XLOC_006987	Ca_v2.0_07707	Ca3_v2.0	71010454	71014460
XLOC_006988	Ca_v2.0_07711	Ca3_v2.0	71043110	71045711
XLOC_006990	Ca_v2.0_07714	Ca3_v2.0	71067109	71070229
XLOC_006991	Ca_v2.0_07717	Ca3_v2.0	71083029	71093560
XLOC_006999	Ca_v2.0_07731	Ca3_v2.0	71244131	71246082
XLOC_007002	Ca_v2.0_07734	Ca3_v2.0	71296557	71298488
XLOC_007003	Ca_v2.0_07735	Ca3_v2.0	71303275	71305314
XLOC_007019	Ca_v2.0_07765	Ca3_v2.0	71526109	71528144
XLOC_007020	Ca_v2.0_07767	Ca3_v2.0	71538541	71539318
XLOC_007026	Ca_v2.0_07775	Ca3_v2.0	71609655	71611755
XLOC_007031	Ca_v2.0_07782	Ca3_v2.0	71704051	71707935
XLOC_007038	Ca_v2.0_07799	Ca3_v2.0	71916432	71920651
XLOC_007045	Ca_v2.0_07820	Ca3_v2.0	71986206	72047343
XLOC_007047	Ca_v2.0_07812	Ca3_v2.0	71986206	72047343
XLOC_007051	Ca_v2.0_07823	Ca3_v2.0	72079140	72081344
XLOC_007054	Ca_v2.0_07830	Ca3_v2.0	72122604	72129255
XLOC_007061	Ca_v2.0_07839,Ca_v2.0_078	Ca3_v2.0	72198069	72206637
XLOC_007062	Ca_v2.0_07841	Ca3_v2.0	72212237	72213479
XLOC_007069	Ca_v2.0_07852	Ca3_v2.0	72310111	72311985
XLOC_007073	Ca_v2.0_07870	Ca3_v2.0	72472551	72474554
XLOC_007077	Ca_v2.0_07876	Ca3_v2.0	72535768	72537148
XLOC_007094	Ca_v2.0_07916	Ca3_v2.0	72844024	72845745
XLOC_007103	Ca_v2.0_07940	Ca3_v2.0	72987263	72987581
XLOC_007111	Ca_v2.0_07955	Ca3_v2.0	73130414	73132632
XLOC_007114	Ca_v2.0_07958	Ca3_v2.0	73143460	73144014

For Review Only

XLOC_007117	Ca_v2.0_07962	Ca3_v2.0	73163740	73168869
XLOC_007119	Ca_v2.0_07967	Ca3_v2.0	73194223	73199504
XLOC_007121	Ca_v2.0_07974	Ca3_v2.0	73258468	73259302
XLOC_007122	Ca_v2.0_07977	Ca3_v2.0	73277070	73279291
XLOC_007132	Ca_v2.0_07999	Ca3_v2.0	73444648	73450174
XLOC_007137	Ca_v2.0_08013	Ca3_v2.0	73564535	73568440
XLOC_007138	Ca_v2.0_08015	Ca3_v2.0	73580519	73583768
XLOC_007139	Ca_v2.0_08017	Ca3_v2.0	73590521	73593281
XLOC_007144	Ca_v2.0_08024	Ca3_v2.0	73646345	73650324
XLOC_007145	Ca_v2.0_08025	Ca3_v2.0	73664000	73666170
XLOC_007151	Ca_v2.0_08038	Ca3_v2.0	73792086	73794631
XLOC_007165	Ca_v2.0_08058	Ca3_v2.0	73945327	73947576
XLOC_007167	Ca_v2.0_08061	Ca3_v2.0	73970319	73972075
XLOC_007171	Ca_v2.0_08073	Ca3_v2.0	74078529	74079080
XLOC_007173	-	Ca3_v2.0	74082637	74096677
XLOC_007178	-	Ca3_v2.0	74150569	74151844
XLOC_007179	Ca_v2.0_08081	Ca3_v2.0	74156453	74158356
XLOC_007185	Ca_v2.0_08096	Ca3_v2.0	74270151	74270877
XLOC_007198	Ca_v2.0_08120	Ca3_v2.0	74487036	74488919
XLOC_007209	Ca_v2.0_08135	Ca3_v2.0	74629075	74632437
XLOC_007219	Ca_v2.0_08150	Ca3_v2.0	74726513	74729019
XLOC_007220	Ca_v2.0_08163	Ca3_v2.0	74818221	74828374
XLOC_007223	Ca_v2.0_08172	Ca3_v2.0	74932882	74940217
XLOC_007225	Ca_v2.0_08180	Ca3_v2.0	74984927	74988517
XLOC_007229	Ca_v2.0_08193	Ca3_v2.0	75048532	75050520
XLOC_007230	Ca_v2.0_08194	Ca3_v2.0	75063301	75065140
XLOC_007234	Ca_v2.0_08204	Ca3_v2.0	75149264	75150978
XLOC_007240	Ca_v2.0_08214	Ca3_v2.0	75221488	75232762
XLOC_007241	Ca_v2.0_08219	Ca3_v2.0	75269170	75277444
XLOC_007244	Ca_v2.0_08224,Ca_v2.0_082	Ca3_v2.0	75309239	75316216
XLOC_007245	Ca_v2.0_08228,Ca_v2.0_082	Ca3_v2.0	75331861	75345605
XLOC_007247	Ca_v2.0_08237	Ca3_v2.0	75399650	75401281
XLOC_007252	Ca_v2.0_08246	Ca3_v2.0	75451589	75454982
XLOC_007255	Ca_v2.0_08251	Ca3_v2.0	75493874	75497366
XLOC_007258	Ca_v2.0_08256	Ca3_v2.0	75535712	75536623
XLOC_007263	Ca_v2.0_08264	Ca3_v2.0	75592168	75592770
XLOC_007272	Ca_v2.0_08280	Ca3_v2.0	75734058	75735441
XLOC_007286	Ca_v2.0_08303,Ca_v2.0_083	Ca3_v2.0	75985435	75990101
XLOC_007292	Ca_v2.0_08313	Ca3_v2.0	76042367	76049867
XLOC_007305	Ca_v2.0_08333	Ca3_v2.0	76197517	76199304
XLOC_007306	Ca_v2.0_08334	Ca3_v2.0	76200681	76203717
XLOC_007310	Ca_v2.0_08342	Ca3_v2.0	76280595	76281298
XLOC_007311	Ca_v2.0_08343	Ca3_v2.0	76288373	76290021
XLOC_007312	Ca_v2.0_08347	Ca3_v2.0	76307003	76308187
XLOC_007333	Ca_v2.0_08387,Ca_v2.0_083	Ca3_v2.0	76514277	76523013
XLOC_007336	Ca_v2.0_08392	Ca3_v2.0	76560023	76566282

For Review Only

XLOC_007339	Ca_v2.0_08397	Ca3_v2.0	76609338	76610409
XLOC_007346	Ca_v2.0_05207	Ca3_v2.0	272929	278502
XLOC_007352	Ca_v2.0_05217	Ca3_v2.0	489506	493210
XLOC_007368	Ca_v2.0_05245	Ca3_v2.0	1142774	1159454
XLOC_007369	Ca_v2.0_05246	Ca3_v2.0	1166669	1170533
XLOC_007371	Ca_v2.0_05249	Ca3_v2.0	1277106	1282443
XLOC_007376	Ca_v2.0_05260	Ca3_v2.0	1539196	1542205
XLOC_007378	Ca_v2.0_05268	Ca3_v2.0	1677598	1682414
XLOC_007391	Ca_v2.0_05295	Ca3_v2.0	2021382	2025876
XLOC_007396	Ca_v2.0_05303	Ca3_v2.0	2228423	2235841
XLOC_007399	Ca_v2.0_05306	Ca3_v2.0	2257931	2261971
XLOC_007413	Ca_v2.0_05332	Ca3_v2.0	2934336	2944412
XLOC_007423	Ca_v2.0_05350	Ca3_v2.0	3334594	3337247
XLOC_007430	Ca_v2.0_05367	Ca3_v2.0	3808355	3823074
XLOC_007447	Ca_v2.0_05389	Ca3_v2.0	4349654	4352562
XLOC_007453	Ca_v2.0_05401	Ca3_v2.0	4553355	4555528
XLOC_007456	Ca_v2.0_05404	Ca3_v2.0	4617126	4617825
XLOC_007458	Ca_v2.0_05406	Ca3_v2.0	4638320	4638996
XLOC_007459	Ca_v2.0_05407	Ca3_v2.0	4647490	4653369
XLOC_007465	Ca_v2.0_05415	Ca3_v2.0	4865851	4877171
XLOC_007467	Ca_v2.0_05419	Ca3_v2.0	4971533	4975551
XLOC_007468	Ca_v2.0_05420	Ca3_v2.0	4983820	4990863
XLOC_007469	Ca_v2.0_05421	Ca3_v2.0	4992422	4998293
XLOC_007471	Ca_v2.0_05423	Ca3_v2.0	5018365	5020375
XLOC_007472	Ca_v2.0_05424	Ca3_v2.0	5082491	5084524
XLOC_007479	Ca_v2.0_05432	Ca3_v2.0	5470051	5474288
XLOC_007483	Ca_v2.0_05439	Ca3_v2.0	5723527	5731549
XLOC_007484	Ca_v2.0_05440	Ca3_v2.0	5734124	5738746
XLOC_007485	Ca_v2.0_05444	Ca3_v2.0	5871984	5877267
XLOC_007491	Ca_v2.0_05467	Ca3_v2.0	6164387	6169140
XLOC_007500	Ca_v2.0_05487	Ca3_v2.0	6601137	6609643
XLOC_007508	Ca_v2.0_05501	Ca3_v2.0	6983495	6984327
XLOC_007511	Ca_v2.0_05506	Ca3_v2.0	7071698	7073957
XLOC_007520	Ca_v2.0_05519	Ca3_v2.0	7541389	7544372
XLOC_007521	Ca_v2.0_05524	Ca3_v2.0	7658339	7659043
XLOC_007527	Ca_v2.0_05540	Ca3_v2.0	8256186	8258777
XLOC_007529	Ca_v2.0_05542	Ca3_v2.0	8279444	8283478
XLOC_007531	Ca_v2.0_05547	Ca3_v2.0	8416762	8422971
XLOC_007534	Ca_v2.0_05553,Ca_v2.0_055!	Ca3_v2.0	9464575	9471520
XLOC_007579	Ca_v2.0_05638	Ca3_v2.0	19191143	19191931
XLOC_007595	Ca_v2.0_05661	Ca3_v2.0	19802102	19803755
XLOC_007600	Ca_v2.0_05671	Ca3_v2.0	20063894	20068308
XLOC_007603	Ca_v2.0_05679	Ca3_v2.0	20551080	20560247
XLOC_007626	Ca_v2.0_05711	Ca3_v2.0	21743744	21750497
XLOC_007629	Ca_v2.0_05718	Ca3_v2.0	22406967	22409075
XLOC_007650	Ca_v2.0_05750	Ca3_v2.0	25405444	25410446

For Review Only

XLOC_007654	Ca_v2.0_05756,Ca_v2.0_057!	Ca3_v2.0	25800759	25804503
XLOC_007661	Ca_v2.0_05766	Ca3_v2.0	26637845	26644422
XLOC_007669	Ca_v2.0_05782	Ca3_v2.0	27695089	27703133
XLOC_007677	Ca_v2.0_05799	Ca3_v2.0	29571005	29588945
XLOC_007683	Ca_v2.0_05814	Ca3_v2.0	30766523	30771451
XLOC_007685	Ca_v2.0_05820	Ca3_v2.0	31284360	31285030
XLOC_007709	Ca_v2.0_05854,Ca_v2.0_058!	Ca3_v2.0	34316059	34317939
XLOC_007727	Ca_v2.0_05887	Ca3_v2.0	35687875	35696780
XLOC_007732	-	Ca3_v2.0	36119912	36121248
XLOC_007737	Ca_v2.0_05898	Ca3_v2.0	36158933	36170315
XLOC_007741	Ca_v2.0_05902	Ca3_v2.0	36388101	36396519
XLOC_007761	Ca_v2.0_05951	Ca3_v2.0	39753906	39755540
XLOC_007766	Ca_v2.0_05958	Ca3_v2.0	40059166	40066088
XLOC_007768	Ca_v2.0_05960	Ca3_v2.0	40158716	40169287
XLOC_007785	Ca_v2.0_05988	Ca3_v2.0	41754274	41759340
XLOC_007787	Ca_v2.0_05992,Ca_v2.0_059!	Ca3_v2.0	42199096	42207194
XLOC_007799	Ca_v2.0_06016	Ca3_v2.0	44069469	44114337
XLOC_007803	Ca_v2.0_06022	Ca3_v2.0	44404521	44408308
XLOC_007819	Ca_v2.0_06048	Ca3_v2.0	45253379	45256020
XLOC_007822	Ca_v2.0_06054	Ca3_v2.0	45962722	45969701
XLOC_007823	Ca_v2.0_06056	Ca3_v2.0	46122043	46123461
XLOC_007832	Ca_v2.0_06068	Ca3_v2.0	46623468	46630236
XLOC_007835	Ca_v2.0_06075	Ca3_v2.0	47010272	47011301
XLOC_007838	Ca_v2.0_06083	Ca3_v2.0	47279749	47281508
XLOC_007845	Ca_v2.0_06091	Ca3_v2.0	47664452	47671129
XLOC_007850	Ca_v2.0_06098	Ca3_v2.0	47982789	48030610
XLOC_007854	Ca_v2.0_06107	Ca3_v2.0	48277079	48279669
XLOC_007855	-	Ca3_v2.0	48297695	48300462
XLOC_007874	Ca_v2.0_06140	Ca3_v2.0	49232844	49233669
XLOC_007881	Ca_v2.0_06155	Ca3_v2.0	49529836	49532309
XLOC_007883	Ca_v2.0_06157	Ca3_v2.0	49594393	49595611
XLOC_007885	-	Ca3_v2.0	49797998	49799182
XLOC_007891	Ca_v2.0_06172	Ca3_v2.0	49889541	49893457
XLOC_007897	Ca_v2.0_06180	Ca3_v2.0	50027245	50030094
XLOC_007898	Ca_v2.0_06181	Ca3_v2.0	50036894	50038664
XLOC_007910	Ca_v2.0_06203	Ca3_v2.0	50534689	50537999
XLOC_007975	Ca_v2.0_06330	Ca3_v2.0	53591210	53594566
XLOC_007977	Ca_v2.0_06332	Ca3_v2.0	53656287	53658299
XLOC_007984	Ca_v2.0_06341	Ca3_v2.0	53898479	53902051
XLOC_007994	Ca_v2.0_06356	Ca3_v2.0	54172020	54178540
XLOC_008001	Ca_v2.0_06368	Ca3_v2.0	54410556	54410829
XLOC_008005	Ca_v2.0_06374	Ca3_v2.0	54793015	54793680
XLOC_008016	Ca_v2.0_06389	Ca3_v2.0	55531538	55539769
XLOC_008019	Ca_v2.0_06395	Ca3_v2.0	55663925	55666356
XLOC_008020	-	Ca3_v2.0	55668875	55670350
XLOC_008022	-	Ca3_v2.0	55688888	55691087

For Review Only

XLOC_008024	Ca_v2.0_06397	Ca3_v2.0	55701108	55702388
XLOC_008025	Ca_v2.0_06399	Ca3_v2.0	55703404	55707761
XLOC_008029	Ca_v2.0_06405	Ca3_v2.0	55846534	55850153
XLOC_008030	Ca_v2.0_06408	Ca3_v2.0	55889729	55893887
XLOC_008035	Ca_v2.0_06418	Ca3_v2.0	56156246	56178290
XLOC_008037	Ca_v2.0_06423	Ca3_v2.0	56202176	56202872
XLOC_008038	Ca_v2.0_06424	Ca3_v2.0	56210185	56211823
XLOC_008039	Ca_v2.0_06425	Ca3_v2.0	56221130	56229194
XLOC_008051	Ca_v2.0_06455	Ca3_v2.0	56983837	57003239
XLOC_008054	Ca_v2.0_06459	Ca3_v2.0	57051084	57058256
XLOC_008055	Ca_v2.0_06460,Ca_v2.0_064(	Ca3_v2.0	57063969	57068630
XLOC_008058	Ca_v2.0_06465	Ca3_v2.0	57121392	57124645
XLOC_008062	Ca_v2.0_06470	Ca3_v2.0	57188920	57193141
XLOC_008063	Ca_v2.0_06472	Ca3_v2.0	57236191	57241698
XLOC_008064	Ca_v2.0_06473	Ca3_v2.0	57264751	57276801
XLOC_008065	Ca_v2.0_06474	Ca3_v2.0	57264751	57276801
XLOC_008067	-	Ca3_v2.0	57300677	57303677
XLOC_008073	Ca_v2.0_06488	Ca3_v2.0	57712720	57716149
XLOC_008081	Ca_v2.0_06503	Ca3_v2.0	57899337	57928414
XLOC_008083	Ca_v2.0_06508	Ca3_v2.0	58011291	58016149
XLOC_008084	Ca_v2.0_06509	Ca3_v2.0	58021007	58023511
XLOC_008086	Ca_v2.0_06516	Ca3_v2.0	58108417	58109485
XLOC_008087	Ca_v2.0_06520	Ca3_v2.0	58133628	58137734
XLOC_008096	Ca_v2.0_06544	Ca3_v2.0	58498374	58503134
XLOC_008097	Ca_v2.0_06546	Ca3_v2.0	58512362	58533301
XLOC_008098	Ca_v2.0_06550,Ca_v2.0_065!	Ca3_v2.0	58539139	58545372
XLOC_008099	Ca_v2.0_06554	Ca3_v2.0	58568199	58571419
XLOC_008100	Ca_v2.0_06556	Ca3_v2.0	58584111	58586503
XLOC_008107	Ca_v2.0_06566	Ca3_v2.0	58807192	58815622
XLOC_008112	Ca_v2.0_06573	Ca3_v2.0	58914673	58923164
XLOC_008115	Ca_v2.0_06582	Ca3_v2.0	59078339	59083938
XLOC_008120	Ca_v2.0_06591	Ca3_v2.0	59209794	59215624
XLOC_008127	Ca_v2.0_06608	Ca3_v2.0	59401766	59403953
XLOC_008134	Ca_v2.0_06618	Ca3_v2.0	59521948	59525720
XLOC_008143	Ca_v2.0_06636	Ca3_v2.0	59684134	59688531
XLOC_008144	Ca_v2.0_06637	Ca3_v2.0	59692021	59700750
XLOC_008145	Ca_v2.0_06638	Ca3_v2.0	59703780	59705227
XLOC_008146	Ca_v2.0_06639	Ca3_v2.0	59708867	59709503
XLOC_008158	Ca_v2.0_06665	Ca3_v2.0	60089979	60091153
XLOC_008171	Ca_v2.0_06690	Ca3_v2.0	60434447	60437695
XLOC_008177	Ca_v2.0_06698	Ca3_v2.0	60529199	60554297
XLOC_008196	Ca_v2.0_06735	Ca3_v2.0	60922925	60923668
XLOC_008199	Ca_v2.0_06740	Ca3_v2.0	60960978	60967355
XLOC_008216	Ca_v2.0_06769	Ca3_v2.0	61394378	61395536
XLOC_008219	Ca_v2.0_06777	Ca3_v2.0	61488812	61495362
XLOC_008224	Ca_v2.0_06784	Ca3_v2.0	61603196	61608700

For Review Only

XLOC_008225	Ca_v2.0_06786	Ca3_v2.0	61635032	61639126
XLOC_008226	Ca_v2.0_06789	Ca3_v2.0	61671429	61678432
XLOC_008229	Ca_v2.0_06800	Ca3_v2.0	61766945	61772481
XLOC_008230	Ca_v2.0_06802	Ca3_v2.0	61801568	61808303
XLOC_008234	Ca_v2.0_06809	Ca3_v2.0	61850655	61853680
XLOC_008235	Ca_v2.0_06811	Ca3_v2.0	61862619	61865832
XLOC_008239	Ca_v2.0_06824	Ca3_v2.0	61965742	61970359
XLOC_008243	Ca_v2.0_06830	Ca3_v2.0	62034150	62041472
XLOC_008249	Ca_v2.0_06843	Ca3_v2.0	62151096	62156400
XLOC_008260	Ca_v2.0_06861	Ca3_v2.0	62342716	62345923
XLOC_008262	Ca_v2.0_06863	Ca3_v2.0	62361774	62365266
XLOC_008279	Ca_v2.0_06894	Ca3_v2.0	62674763	62682242
XLOC_008280	-	Ca3_v2.0	62696673	62704216
XLOC_008291	Ca_v2.0_06909	Ca3_v2.0	62989471	63006985
XLOC_008295	Ca_v2.0_06916	Ca3_v2.0	63069571	63074649
XLOC_008299	Ca_v2.0_06923	Ca3_v2.0	63103917	63108469
XLOC_008300	Ca_v2.0_06924	Ca3_v2.0	63112923	63116497
XLOC_008302	Ca_v2.0_06926	Ca3_v2.0	63127038	63135331
XLOC_008305	Ca_v2.0_06929	Ca3_v2.0	63150427	63152882
XLOC_008306	Ca_v2.0_06930	Ca3_v2.0	63168624	63169426
XLOC_008309	Ca_v2.0_06937,Ca_v2.0_069:	Ca3_v2.0	63209846	63215817
XLOC_008311	Ca_v2.0_06944	Ca3_v2.0	63290364	63291423
XLOC_008316	Ca_v2.0_06954	Ca3_v2.0	63381052	63383266
XLOC_008322	Ca_v2.0_06961	Ca3_v2.0	63499851	63502685
XLOC_008325	Ca_v2.0_06964	Ca3_v2.0	63542539	63548965
XLOC_008326	Ca_v2.0_06966	Ca3_v2.0	63564941	63566908
XLOC_008330	Ca_v2.0_06974	Ca3_v2.0	63647347	63650942
XLOC_008334	Ca_v2.0_06983	Ca3_v2.0	63729320	63730649
XLOC_008353	Ca_v2.0_07008	Ca3_v2.0	64218158	64228266
XLOC_008357	Ca_v2.0_07017	Ca3_v2.0	64275124	64276226
XLOC_008358	Ca_v2.0_07021	Ca3_v2.0	64306658	64315284
XLOC_008371	Ca_v2.0_07055	Ca3_v2.0	64697005	64701025
XLOC_008373	Ca_v2.0_07066	Ca3_v2.0	64730104	64813491
XLOC_008378	Ca_v2.0_07065	Ca3_v2.0	64730104	64813491
XLOC_008381	Ca_v2.0_07069	Ca3_v2.0	64852503	64864073
XLOC_008391	Ca_v2.0_07082	Ca3_v2.0	64965136	64968319
XLOC_008392	Ca_v2.0_07083	Ca3_v2.0	64969945	64971570
XLOC_008399	Ca_v2.0_07105	Ca3_v2.0	65160287	65162948
XLOC_008401	Ca_v2.0_07110	Ca3_v2.0	65215469	65217048
XLOC_008408	Ca_v2.0_07126	Ca3_v2.0	65332200	65334784
XLOC_008412	Ca_v2.0_07137	Ca3_v2.0	65432800	65435764
XLOC_008422	Ca_v2.0_07153	Ca3_v2.0	65630206	65636266
XLOC_008427	Ca_v2.0_07161	Ca3_v2.0	65711074	65713707
XLOC_008448	Ca_v2.0_07195	Ca3_v2.0	66034791	66037464
XLOC_008458	Ca_v2.0_07209	Ca3_v2.0	66261672	66266055
XLOC_008467	Ca_v2.0_07227	Ca3_v2.0	66480704	66485761

For Review Only

XLOC_008470	Ca_v2.0_07231	Ca3_v2.0	66527378	66533835
XLOC_008471	Ca_v2.0_07234	Ca3_v2.0	66547380	66596414
XLOC_008476	Ca_v2.0_07245	Ca3_v2.0	66677647	66679612
XLOC_008479	Ca_v2.0_07248	Ca3_v2.0	66716082	66718212
XLOC_008512	Ca_v2.0_07307	Ca3_v2.0	67330816	67331663
XLOC_008517	Ca_v2.0_07313	Ca3_v2.0	67385309	67390192
XLOC_008518	Ca_v2.0_07314	Ca3_v2.0	67396322	67400933
XLOC_008519	Ca_v2.0_07315	Ca3_v2.0	67411775	67422179
XLOC_008521	Ca_v2.0_07319	Ca3_v2.0	67461921	67481917
XLOC_008529	Ca_v2.0_07331	Ca3_v2.0	67577521	67583379
XLOC_008535	Ca_v2.0_07347	Ca3_v2.0	67709065	67710352
XLOC_008536	Ca_v2.0_07348	Ca3_v2.0	67723367	67724630
XLOC_008537	Ca_v2.0_07349	Ca3_v2.0	67728128	67735137
XLOC_008548	Ca_v2.0_07365	Ca3_v2.0	67842424	67843220
XLOC_008549	Ca_v2.0_07369	Ca3_v2.0	67866099	67867228
XLOC_008550	Ca_v2.0_07370	Ca3_v2.0	67870457	67872869
XLOC_008553	Ca_v2.0_07385	Ca3_v2.0	67973116	67976581
XLOC_008554	Ca_v2.0_07388	Ca3_v2.0	68000406	68002737
XLOC_008559	Ca_v2.0_07402	Ca3_v2.0	68123858	68124750
XLOC_008560	Ca_v2.0_07403	Ca3_v2.0	68146960	68148507
XLOC_008562	Ca_v2.0_07405	Ca3_v2.0	68159276	68160019
XLOC_008563	Ca_v2.0_07407	Ca3_v2.0	68174188	68175943
XLOC_008566	Ca_v2.0_07416	Ca3_v2.0	68219892	68220450
XLOC_008567	Ca_v2.0_07417	Ca3_v2.0	68223659	68224406
XLOC_008580	Ca_v2.0_07443	Ca3_v2.0	68431731	68432853
XLOC_008584	Ca_v2.0_07448	Ca3_v2.0	68511014	68512688
XLOC_008590	Ca_v2.0_07463	Ca3_v2.0	68638898	68645837
XLOC_008593	Ca_v2.0_07468	Ca3_v2.0	68684174	68685857
XLOC_008594	Ca_v2.0_07469	Ca3_v2.0	68686977	68694630
XLOC_008596	Ca_v2.0_07475	Ca3_v2.0	68767533	68771247
XLOC_008600	Ca_v2.0_07487	Ca3_v2.0	68862179	68863769
XLOC_008604	Ca_v2.0_07496	Ca3_v2.0	68951143	68953026
XLOC_008608	Ca_v2.0_07505	Ca3_v2.0	69062224	69063159
XLOC_008612	Ca_v2.0_07511	Ca3_v2.0	69100860	69103558
XLOC_008618	Ca_v2.0_07524	Ca3_v2.0	69194738	69195573
XLOC_008629	Ca_v2.0_07539	Ca3_v2.0	69330926	69332441
XLOC_008632	Ca_v2.0_07546	Ca3_v2.0	69388142	69399978
XLOC_008641	Ca_v2.0_07560	Ca3_v2.0	69523866	69532346
XLOC_008642	Ca_v2.0_07558	Ca3_v2.0	69523866	69532346
XLOC_008645	Ca_v2.0_07565	Ca3_v2.0	69570873	69578458
XLOC_008653	Ca_v2.0_07584	Ca3_v2.0	69788764	69790269
XLOC_008658	Ca_v2.0_07596	Ca3_v2.0	69906197	69925044
XLOC_008668	Ca_v2.0_07612	Ca3_v2.0	70044327	70047556
XLOC_008670	Ca_v2.0_07616	Ca3_v2.0	70087690	70088402
XLOC_008671	Ca_v2.0_07617	Ca3_v2.0	70093116	70093909
XLOC_008673	Ca_v2.0_07621	Ca3_v2.0	70124101	70126294

For Review Only

XLOC_008679	Ca_v2.0_07633	Ca3_v2.0	70213568	70215042
XLOC_008693	Ca_v2.0_07657	Ca3_v2.0	70393246	70396882
XLOC_008716	Ca_v2.0_07706	Ca3_v2.0	71005711	71007480
XLOC_008726	Ca_v2.0_07723	Ca3_v2.0	71147828	71151355
XLOC_008731	Ca_v2.0_07734	Ca3_v2.0	71296557	71298488
XLOC_008737	Ca_v2.0_07743	Ca3_v2.0	71360780	71363655
XLOC_008739	Ca_v2.0_07746	Ca3_v2.0	71378293	71379806
XLOC_008740	Ca_v2.0_07750	Ca3_v2.0	71411442	71413230
XLOC_008748	Ca_v2.0_07764	Ca3_v2.0	71513489	71516378
XLOC_008754	Ca_v2.0_07780	Ca3_v2.0	71654020	71657661
XLOC_008762	Ca_v2.0_07790	Ca3_v2.0	71829190	71829484
XLOC_008765	Ca_v2.0_07794	Ca3_v2.0	71851123	71855106
XLOC_008773	Ca_v2.0_07810	Ca3_v2.0	71973862	71981530
XLOC_008777	Ca_v2.0_07818	Ca3_v2.0	71986206	72047343
XLOC_008787	Ca_v2.0_07841	Ca3_v2.0	72212237	72213479
XLOC_008790	Ca_v2.0_07849	Ca3_v2.0	72292069	72295604
XLOC_008791	Ca_v2.0_07853	Ca3_v2.0	72326952	72328149
XLOC_008792	Ca_v2.0_07854,Ca_v2.0_078	Ca3_v2.0	72331326	72335521
XLOC_008802	Ca_v2.0_07868,Ca_v2.0_078	Ca3_v2.0	72450474	72460772
XLOC_008807	Ca_v2.0_07886	Ca3_v2.0	72604243	72607244
XLOC_008817	Ca_v2.0_07898	Ca3_v2.0	72690009	72695955
XLOC_008819	Ca_v2.0_07900	Ca3_v2.0	72716414	72721925
XLOC_008820	Ca_v2.0_07905	Ca3_v2.0	72742449	72747111
XLOC_008821	Ca_v2.0_07906	Ca3_v2.0	72748177	72750901
XLOC_008825	Ca_v2.0_07911	Ca3_v2.0	72791085	72805231
XLOC_008832	Ca_v2.0_07920	Ca3_v2.0	72870085	72871827
XLOC_008833	Ca_v2.0_07922	Ca3_v2.0	72881390	72882018
XLOC_008841	Ca_v2.0_07933	Ca3_v2.0	72950217	72953813
XLOC_008843	Ca_v2.0_07935	Ca3_v2.0	72961949	72964470
XLOC_008844	Ca_v2.0_07936	Ca3_v2.0	72968104	72968922
XLOC_008851	Ca_v2.0_07950	Ca3_v2.0	73110348	73115021
XLOC_008855	Ca_v2.0_07954	Ca3_v2.0	73126396	73129286
XLOC_008856	Ca_v2.0_07957	Ca3_v2.0	73138387	73142320
XLOC_008859	Ca_v2.0_07961	Ca3_v2.0	73158751	73161472
XLOC_008863	Ca_v2.0_07968	Ca3_v2.0	73194223	73199504
XLOC_008868	Ca_v2.0_07975	Ca3_v2.0	73261318	73263012
XLOC_008871	Ca_v2.0_07981	Ca3_v2.0	73301578	73304385
XLOC_008880	Ca_v2.0_07995	Ca3_v2.0	73419173	73424449
XLOC_008883	Ca_v2.0_08001	Ca3_v2.0	73465829	73469511
XLOC_008886	Ca_v2.0_08004	Ca3_v2.0	73501238	73502087
XLOC_008890	Ca_v2.0_08011	Ca3_v2.0	73548660	73551300
XLOC_008893	Ca_v2.0_08017	Ca3_v2.0	73590521	73593281
XLOC_008904	Ca_v2.0_08037	Ca3_v2.0	73764649	73773117
XLOC_008905	Ca_v2.0_08041	Ca3_v2.0	73823063	73827763
XLOC_008908	Ca_v2.0_08049	Ca3_v2.0	73878957	73881965
XLOC_008911	Ca_v2.0_08057	Ca3_v2.0	73931030	73932084

For Review Only

XLOC_008912	Ca_v2.0_08059	Ca3_v2.0	73948507	73952117
XLOC_008917	Ca_v2.0_08066	Ca3_v2.0	74000144	74012125
XLOC_008918	Ca_v2.0_08067	Ca3_v2.0	74021175	74027043
XLOC_008922	Ca_v2.0_08072	Ca3_v2.0	74064574	74071337
XLOC_008924	Ca_v2.0_08078	Ca3_v2.0	74122518	74124114
XLOC_008926	Ca_v2.0_08082	Ca3_v2.0	74166342	74166741
XLOC_008927	Ca_v2.0_08084	Ca3_v2.0	74175292	74179860
XLOC_008935	Ca_v2.0_08099	Ca3_v2.0	74289049	74291149
XLOC_008936	Ca_v2.0_08100	Ca3_v2.0	74298113	74301120
XLOC_008938	Ca_v2.0_08104	Ca3_v2.0	74317190	74317923
XLOC_008941	Ca_v2.0_08107	Ca3_v2.0	74342458	74346269
XLOC_008948	Ca_v2.0_08127	Ca3_v2.0	74564332	74568661
XLOC_008952	Ca_v2.0_08144	Ca3_v2.0	74674971	74690934
XLOC_008957	Ca_v2.0_08150	Ca3_v2.0	74726513	74729019
XLOC_008967	Ca_v2.0_08160	Ca3_v2.0	74803455	74804150
XLOC_008969	Ca_v2.0_08162	Ca3_v2.0	74814692	74817125
XLOC_008970	Ca_v2.0_08163	Ca3_v2.0	74818221	74828374
XLOC_008974	Ca_v2.0_08169	Ca3_v2.0	74884452	74888706
XLOC_008975	Ca_v2.0_08170	Ca3_v2.0	74889818	74913932
XLOC_008986	Ca_v2.0_08189	Ca3_v2.0	75021184	75029419
XLOC_008995	Ca_v2.0_08202	Ca3_v2.0	75125238	75126575
XLOC_008996	Ca_v2.0_08203	Ca3_v2.0	75128426	75138816
XLOC_009009	Ca_v2.0_08222,Ca_v2.0_082:	Ca3_v2.0	75304209	75307963
XLOC_009013	Ca_v2.0_08227	Ca3_v2.0	75324674	75327997
XLOC_009026	Ca_v2.0_08250	Ca3_v2.0	75476419	75480651
XLOC_009031	Ca_v2.0_08261	Ca3_v2.0	75562464	75567320
XLOC_009034	-	Ca3_v2.0	75634666	75643256
XLOC_009041	Ca_v2.0_08277	Ca3_v2.0	75713233	75722284
XLOC_009049	Ca_v2.0_08298	Ca3_v2.0	75955722	75958117
XLOC_009055	Ca_v2.0_08312	Ca3_v2.0	76035285	76037726
XLOC_009060	Ca_v2.0_08324	Ca3_v2.0	76127323	76129105
XLOC_009061	Ca_v2.0_08325	Ca3_v2.0	76137870	76141470
XLOC_009070	Ca_v2.0_08341	Ca3_v2.0	76262968	76265474
XLOC_009072	Ca_v2.0_08344	Ca3_v2.0	76293436	76296811
XLOC_009078	Ca_v2.0_08351	Ca3_v2.0	76323696	76330052
XLOC_009079	Ca_v2.0_08352	Ca3_v2.0	76330891	76333052
XLOC_009083	Ca_v2.0_08365	Ca3_v2.0	76406465	76408564
XLOC_009086	Ca_v2.0_08369	Ca3_v2.0	76428210	76432981
XLOC_009098	Ca_v2.0_08398	Ca3_v2.0	76611635	76634961
XLOC_009102	-	Ca3_v2.0	5034456	5036081
XLOC_009109	-	Ca3_v2.0	8156103	8156677
XLOC_009110	-	Ca3_v2.0	8288103	8288817
XLOC_009113	-	Ca3_v2.0	15512124	15513013
XLOC_009114	-	Ca3_v2.0	15533458	15533865
XLOC_009117	-	Ca3_v2.0	18099237	18099704
XLOC_009120	-	Ca3_v2.0	19587514	19588242

For Review Only

XLOC_009125	-	Ca3_v2.0	24965296	24965582
XLOC_009127	-	Ca3_v2.0	25561946	25562212
XLOC_009143	-	Ca3_v2.0	37896531	37896759
XLOC_009144	-	Ca3_v2.0	38093489	38093725
XLOC_009146	-	Ca3_v2.0	39003345	39006698
XLOC_009147	-	Ca3_v2.0	39443622	39444414
XLOC_009150	-	Ca3_v2.0	43751284	43751862
XLOC_009161	-	Ca3_v2.0	49847976	49848264
XLOC_009162	-	Ca3_v2.0	50266656	50274255
XLOC_009167	-	Ca3_v2.0	54260334	54261792
XLOC_009183	-	Ca3_v2.0	58767027	58767326
XLOC_009187	-	Ca3_v2.0	60689017	60689618
XLOC_009188	-	Ca3_v2.0	61903037	61903582
XLOC_009198	-	Ca3_v2.0	66726017	66726305
XLOC_009199	-	Ca3_v2.0	66775909	66776774
XLOC_009204	-	Ca3_v2.0	69007309	69007886
XLOC_009205	-	Ca3_v2.0	69020820	69021720
XLOC_009206	-	Ca3_v2.0	69026669	69027411
XLOC_009224	-	Ca3_v2.0	75076346	75076974
XLOC_009234	Ca_v2.0_08411	Ca4_v2.0	86219	93059
XLOC_009236	Ca_v2.0_08413	Ca4_v2.0	115661	116908
XLOC_009240	Ca_v2.0_08421	Ca4_v2.0	219274	219885
XLOC_009243	Ca_v2.0_08426	Ca4_v2.0	250800	254497
XLOC_009246	Ca_v2.0_08432	Ca4_v2.0	314887	318872
XLOC_009251	Ca_v2.0_08439	Ca4_v2.0	382733	387989
XLOC_009258	Ca_v2.0_08456	Ca4_v2.0	581038	585469
XLOC_009263	Ca_v2.0_08471	Ca4_v2.0	741420	749687
XLOC_009265	Ca_v2.0_08473,Ca_v2.0_084	Ca4_v2.0	758388	761323
XLOC_009270	Ca_v2.0_08484	Ca4_v2.0	830369	835184
XLOC_009271	Ca_v2.0_08489	Ca4_v2.0	861803	873288
XLOC_009278	Ca_v2.0_08504	Ca4_v2.0	990695	1003298
XLOC_009279	Ca_v2.0_08505	Ca4_v2.0	1019796	1025144
XLOC_009285	Ca_v2.0_08517	Ca4_v2.0	1149495	1150172
XLOC_009286	Ca_v2.0_08518	Ca4_v2.0	1155034	1161578
XLOC_009289	Ca_v2.0_08525	Ca4_v2.0	1221215	1227371
XLOC_009291	Ca_v2.0_08528	Ca4_v2.0	1257186	1258613
XLOC_009302	Ca_v2.0_08547	Ca4_v2.0	1449915	1451891
XLOC_009305	Ca_v2.0_08551	Ca4_v2.0	1483039	1484408
XLOC_009308	Ca_v2.0_08554	Ca4_v2.0	1528479	1530249
XLOC_009309	Ca_v2.0_08557	Ca4_v2.0	1550173	1553042
XLOC_009311	Ca_v2.0_08559	Ca4_v2.0	1563800	1569955
XLOC_009316	Ca_v2.0_08571	Ca4_v2.0	1749335	1751617
XLOC_009320	Ca_v2.0_08582	Ca4_v2.0	1865861	1866895
XLOC_009321	Ca_v2.0_08583	Ca4_v2.0	1879537	1881436
XLOC_009326	Ca_v2.0_08587	Ca4_v2.0	1890009	1973190
XLOC_009328	Ca_v2.0_08589	Ca4_v2.0	1890009	1973190

For Review Only

XLOC_009332	Ca_v2.0_08593	Ca4_v2.0	1890009	1973190
XLOC_009335	Ca_v2.0_08600	Ca4_v2.0	2036000	2042910
XLOC_009337	Ca_v2.0_08603	Ca4_v2.0	2068831	2080030
XLOC_009349	Ca_v2.0_08635	Ca4_v2.0	2469501	2479840
XLOC_009353	Ca_v2.0_08641	Ca4_v2.0	2523695	2527923
XLOC_009355	Ca_v2.0_08643	Ca4_v2.0	2549221	2551642
XLOC_009359	Ca_v2.0_08653	Ca4_v2.0	2616545	2621050
XLOC_009363	Ca_v2.0_08664	Ca4_v2.0	2740167	2740634
XLOC_009365	Ca_v2.0_08666	Ca4_v2.0	2764144	2765216
XLOC_009369	Ca_v2.0_08671	Ca4_v2.0	2826861	2830694
XLOC_009374	Ca_v2.0_08677	Ca4_v2.0	2881802	2885694
XLOC_009376	Ca_v2.0_08681	Ca4_v2.0	2917200	2922359
XLOC_009387	Ca_v2.0_08701	Ca4_v2.0	3103945	3106382
XLOC_009395	Ca_v2.0_08731	Ca4_v2.0	3394916	3399617
XLOC_009400	Ca_v2.0_08739	Ca4_v2.0	3490785	3494010
XLOC_009402	Ca_v2.0_08741	Ca4_v2.0	3504687	3506033
XLOC_009411	Ca_v2.0_08752	Ca4_v2.0	3635120	3639460
XLOC_009414	Ca_v2.0_08757	Ca4_v2.0	3664779	3668706
XLOC_009418	Ca_v2.0_08769	Ca4_v2.0	3749343	3753454
XLOC_009421	Ca_v2.0_08775	Ca4_v2.0	3809138	3813819
XLOC_009422	Ca_v2.0_08776	Ca4_v2.0	3816124	3826507
XLOC_009429	Ca_v2.0_08786	Ca4_v2.0	3926880	3929088
XLOC_009431	Ca_v2.0_08790,Ca_v2.0_087	Ca4_v2.0	3977840	3988448
XLOC_009436	Ca_v2.0_08803	Ca4_v2.0	4136125	4139217
XLOC_009451	Ca_v2.0_08831	Ca4_v2.0	4416739	4421161
XLOC_009453	Ca_v2.0_08833	Ca4_v2.0	4432350	4434160
XLOC_009469	Ca_v2.0_08876,Ca_v2.0_088	Ca4_v2.0	4869988	4873792
XLOC_009483	Ca_v2.0_08903	Ca4_v2.0	5099147	5104558
XLOC_009496	Ca_v2.0_08922	Ca4_v2.0	5273212	5274903
XLOC_009524	Ca_v2.0_08965	Ca4_v2.0	5638028	5645089
XLOC_009529	Ca_v2.0_08973	Ca4_v2.0	5737333	5739448
XLOC_009531	Ca_v2.0_08975	Ca4_v2.0	5747838	5750960
XLOC_009540	Ca_v2.0_08994	Ca4_v2.0	5906338	5916093
XLOC_009543	Ca_v2.0_08997	Ca4_v2.0	5932315	5934364
XLOC_009545	Ca_v2.0_09001	Ca4_v2.0	5953354	5954697
XLOC_009547	Ca_v2.0_09007	Ca4_v2.0	6002491	6005261
XLOC_009548	Ca_v2.0_09008	Ca4_v2.0	6012421	6013600
XLOC_009550	Ca_v2.0_09014	Ca4_v2.0	6068599	6074501
XLOC_009555	Ca_v2.0_09022	Ca4_v2.0	6164553	6166477
XLOC_009562	Ca_v2.0_09040	Ca4_v2.0	6308352	6315956
XLOC_009563	Ca_v2.0_09041	Ca4_v2.0	6308352	6315956
XLOC_009564	Ca_v2.0_09042	Ca4_v2.0	6308352	6315956
XLOC_009565	Ca_v2.0_09043	Ca4_v2.0	6320255	6320522
XLOC_009567	Ca_v2.0_09046	Ca4_v2.0	6344425	6356876
XLOC_009568	Ca_v2.0_09047	Ca4_v2.0	6383228	6385315
XLOC_009573	Ca_v2.0_09056	Ca4_v2.0	6458385	6506238

For Review Only

XLOC_009585	Ca_v2.0_09078	Ca4_v2.0	6659270	6660983
XLOC_009592	Ca_v2.0_09090	Ca4_v2.0	6768909	6770487
XLOC_009595	Ca_v2.0_09096	Ca4_v2.0	6797767	6800456
XLOC_009596	Ca_v2.0_09097	Ca4_v2.0	6810023	6813296
XLOC_009597	Ca_v2.0_09098	Ca4_v2.0	6818340	6823789
XLOC_009598	Ca_v2.0_09099	Ca4_v2.0	6828115	6830435
XLOC_009599	Ca_v2.0_09100	Ca4_v2.0	6841111	6842421
XLOC_009607	Ca_v2.0_09113	Ca4_v2.0	6983586	6986255
XLOC_009614	Ca_v2.0_09120	Ca4_v2.0	7043913	7045011
XLOC_009620	Ca_v2.0_09129	Ca4_v2.0	7152174	7154536
XLOC_009628	Ca_v2.0_09145	Ca4_v2.0	7288341	7289171
XLOC_009631	Ca_v2.0_09148	Ca4_v2.0	7332393	7338614
XLOC_009634	Ca_v2.0_09152	Ca4_v2.0	7386600	7401081
XLOC_009635	Ca_v2.0_09154	Ca4_v2.0	7415363	7418504
XLOC_009636	Ca_v2.0_09155	Ca4_v2.0	7420595	7422184
XLOC_009646	Ca_v2.0_09174	Ca4_v2.0	7639975	7644304
XLOC_009651	Ca_v2.0_09182	Ca4_v2.0	7704984	7708055
XLOC_009656	Ca_v2.0_09194	Ca4_v2.0	7843831	7844646
XLOC_009660	Ca_v2.0_09203	Ca4_v2.0	7934683	7935758
XLOC_009663	Ca_v2.0_09209	Ca4_v2.0	7987662	7992671
XLOC_009668	Ca_v2.0_09221	Ca4_v2.0	8123081	8127736
XLOC_009689	Ca_v2.0_09266	Ca4_v2.0	8477652	8478804
XLOC_009691	Ca_v2.0_09271	Ca4_v2.0	8493083	8517832
XLOC_009702	Ca_v2.0_09282	Ca4_v2.0	8621064	8624135
XLOC_009704	Ca_v2.0_09285	Ca4_v2.0	8639934	8642303
XLOC_009711	Ca_v2.0_09311	Ca4_v2.0	8952071	8955336
XLOC_009713	-	Ca4_v2.0	8970299	8985933
XLOC_009723	Ca_v2.0_09326	Ca4_v2.0	9159812	9161947
XLOC_009727	Ca_v2.0_09336	Ca4_v2.0	9280940	9284690
XLOC_009734	Ca_v2.0_09352	Ca4_v2.0	9504653	9513516
XLOC_009735	Ca_v2.0_09353	Ca4_v2.0	9537382	9541641
XLOC_009736	Ca_v2.0_09354	Ca4_v2.0	9543314	9545461
XLOC_009742	Ca_v2.0_09362	Ca4_v2.0	9626796	9628340
XLOC_009749	Ca_v2.0_09370	Ca4_v2.0	9712809	9714646
XLOC_009762	Ca_v2.0_09393	Ca4_v2.0	9882089	10147454
XLOC_009763	Ca_v2.0_09394	Ca4_v2.0	9882089	10147454
XLOC_009764	Ca_v2.0_09397	Ca4_v2.0	9882089	10147454
XLOC_009766	Ca_v2.0_09399	Ca4_v2.0	9882089	10147454
XLOC_009773	Ca_v2.0_09417	Ca4_v2.0	10300858	10301683
XLOC_009779	Ca_v2.0_09431	Ca4_v2.0	10453188	10461183
XLOC_009784	Ca_v2.0_09439	Ca4_v2.0	10524032	10526865
XLOC_009792	Ca_v2.0_09451	Ca4_v2.0	10653607	10655776
XLOC_009795	Ca_v2.0_09468	Ca4_v2.0	10827247	10831883
XLOC_009813	Ca_v2.0_09506	Ca4_v2.0	11166829	11167474
XLOC_009817	Ca_v2.0_09515	Ca4_v2.0	11234795	11237958
XLOC_009819	Ca_v2.0_09520	Ca4_v2.0	11289837	11291931

For Review Only

XLOC_009821	Ca_v2.0_09524	Ca4_v2.0	11390709	11393676
XLOC_009829	Ca_v2.0_09543	Ca4_v2.0	11674115	11681265
XLOC_009830	Ca_v2.0_09544	Ca4_v2.0	11686034	11687300
XLOC_009831	Ca_v2.0_09545	Ca4_v2.0	11690464	11692408
XLOC_009836	Ca_v2.0_09552	Ca4_v2.0	11744960	11746572
XLOC_009845	-	Ca4_v2.0	11931805	11934292
XLOC_009849	Ca_v2.0_09574	Ca4_v2.0	12007877	12010515
XLOC_009861	-	Ca4_v2.0	12228174	12228865
XLOC_009862	Ca_v2.0_09594	Ca4_v2.0	12269369	12271224
XLOC_009870	Ca_v2.0_09608	Ca4_v2.0	12369524	12374472
XLOC_009876	Ca_v2.0_09617	Ca4_v2.0	12437237	12437742
XLOC_009878	Ca_v2.0_09620	Ca4_v2.0	12456645	12457428
XLOC_009879	Ca_v2.0_09621	Ca4_v2.0	12473043	12474498
XLOC_009886	Ca_v2.0_09636	Ca4_v2.0	12665833	12670062
XLOC_009887	Ca_v2.0_09638	Ca4_v2.0	12680125	12681523
XLOC_009888	Ca_v2.0_09639	Ca4_v2.0	12692960	12708871
XLOC_009892	Ca_v2.0_09644	Ca4_v2.0	12733151	12735448
XLOC_009895	Ca_v2.0_09646	Ca4_v2.0	12748599	12750657
XLOC_009911	Ca_v2.0_09682	Ca4_v2.0	13121762	13126374
XLOC_009912	Ca_v2.0_09683	Ca4_v2.0	13127532	13128870
XLOC_009916	Ca_v2.0_09694	Ca4_v2.0	13259410	13261499
XLOC_009919	Ca_v2.0_09698	Ca4_v2.0	13322069	13326300
XLOC_009920	Ca_v2.0_09699	Ca4_v2.0	13339630	13345329
XLOC_009924	Ca_v2.0_09705	Ca4_v2.0	13421908	13424523
XLOC_009926	Ca_v2.0_09707	Ca4_v2.0	13455227	13456304
XLOC_009930	Ca_v2.0_09713	Ca4_v2.0	13487527	13528241
XLOC_009941	Ca_v2.0_09732	Ca4_v2.0	13636154	13638327
XLOC_009964	Ca_v2.0_09774	Ca4_v2.0	13977721	13978749
XLOC_009966	Ca_v2.0_09776	Ca4_v2.0	13985020	13994133
XLOC_009968	Ca_v2.0_09778	Ca4_v2.0	14009720	14014324
XLOC_009969	-	Ca4_v2.0	14015144	14016886
XLOC_009971	Ca_v2.0_09779	Ca4_v2.0	14023829	14042044
XLOC_009994	Ca_v2.0_09825	Ca4_v2.0	14488322	14489714
XLOC_010001	Ca_v2.0_09839	Ca4_v2.0	14646395	14647916
XLOC_010006	Ca_v2.0_09851,Ca_v2.0_098!	Ca4_v2.0	14731119	14751776
XLOC_010008	Ca_v2.0_09853	Ca4_v2.0	14731119	14751776
XLOC_010015	Ca_v2.0_09865	Ca4_v2.0	14845591	14849164
XLOC_010017	Ca_v2.0_09868	Ca4_v2.0	14882759	14886076
XLOC_010024	Ca_v2.0_09887	Ca4_v2.0	15053849	15054994
XLOC_010025	Ca_v2.0_09888	Ca4_v2.0	15059546	15060234
XLOC_010026	Ca_v2.0_09890	Ca4_v2.0	15097427	15101041
XLOC_010027	Ca_v2.0_09891	Ca4_v2.0	15101760	15103327
XLOC_010029	Ca_v2.0_09894,Ca_v2.0_098!	Ca4_v2.0	15129377	15133452
XLOC_010030	Ca_v2.0_09896	Ca4_v2.0	15133674	15138755
XLOC_010032	Ca_v2.0_09899	Ca4_v2.0	15177303	15187654
XLOC_010035	Ca_v2.0_09904	Ca4_v2.0	15228869	15237453

For Review Only

XLOC_010036	Ca_v2.0_09906	Ca4_v2.0	15239426	15243309
XLOC_010040	Ca_v2.0_09911	Ca4_v2.0	15267657	15273045
XLOC_010046	Ca_v2.0_09922	Ca4_v2.0	15446803	15466514
XLOC_010054	Ca_v2.0_09942	Ca4_v2.0	15737451	15740370
XLOC_010056	Ca_v2.0_09944	Ca4_v2.0	15760508	15764865
XLOC_010067	Ca_v2.0_09961	Ca4_v2.0	15957613	15958792
XLOC_010069	-	Ca4_v2.0	16045478	16048761
XLOC_010071	Ca_v2.0_09968	Ca4_v2.0	16083790	16085919
XLOC_010079	Ca_v2.0_09983	Ca4_v2.0	16318263	16323415
XLOC_010082	Ca_v2.0_09986	Ca4_v2.0	16361303	16363284
XLOC_010083	Ca_v2.0_09987	Ca4_v2.0	16365175	16367132
XLOC_010086	Ca_v2.0_09990	Ca4_v2.0	16390033	16393742
XLOC_010088	Ca_v2.0_09995	Ca4_v2.0	16430810	16434951
XLOC_010090	Ca_v2.0_09998	Ca4_v2.0	16455572	16457966
XLOC_010095	Ca_v2.0_10007	Ca4_v2.0	16586622	16588687
XLOC_010105	Ca_v2.0_10024	Ca4_v2.0	16745518	16746663
XLOC_010127	Ca_v2.0_10074	Ca4_v2.0	17332834	17335811
XLOC_010128	Ca_v2.0_10075	Ca4_v2.0	17339184	17349390
XLOC_010131	Ca_v2.0_10081	Ca4_v2.0	17394284	17398601
XLOC_010136	Ca_v2.0_10088	Ca4_v2.0	17490600	17501574
XLOC_010139	Ca_v2.0_10091	Ca4_v2.0	17511487	17515034
XLOC_010146	Ca_v2.0_10101	Ca4_v2.0	17566821	17568122
XLOC_010147	Ca_v2.0_10102	Ca4_v2.0	17578966	17582205
XLOC_010150	Ca_v2.0_10111	Ca4_v2.0	17699656	17701640
XLOC_010155	Ca_v2.0_10117	Ca4_v2.0	17741885	17742883
XLOC_010161	Ca_v2.0_10136	Ca4_v2.0	17874329	17876029
XLOC_010165	Ca_v2.0_10143	Ca4_v2.0	17977027	17984878
XLOC_010170	Ca_v2.0_10155	Ca4_v2.0	18375882	18381204
XLOC_010191	Ca_v2.0_10191	Ca4_v2.0	18893113	18896720
XLOC_010192	Ca_v2.0_10196	Ca4_v2.0	18917888	18924245
XLOC_010204	Ca_v2.0_10219	Ca4_v2.0	19230351	19285067
XLOC_010211	Ca_v2.0_10228	Ca4_v2.0	19403029	19406738
XLOC_010227	Ca_v2.0_10257	Ca4_v2.0	19834436	19838140
XLOC_010232	Ca_v2.0_10268	Ca4_v2.0	20101785	20106499
XLOC_010233	Ca_v2.0_10272	Ca4_v2.0	20127515	20131537
XLOC_010234	Ca_v2.0_10273	Ca4_v2.0	20206281	20210091
XLOC_010236	Ca_v2.0_10275	Ca4_v2.0	20248746	20252408
XLOC_010244	Ca_v2.0_10292	Ca4_v2.0	20518629	20520919
XLOC_010247	Ca_v2.0_10300	Ca4_v2.0	20669538	20678832
XLOC_010256	Ca_v2.0_10313	Ca4_v2.0	20993458	20998941
XLOC_010257	Ca_v2.0_10315	Ca4_v2.0	21022202	21023321
XLOC_010260	Ca_v2.0_10324	Ca4_v2.0	21485317	21498688
XLOC_010268	Ca_v2.0_10340	Ca4_v2.0	22454406	22458532
XLOC_010276	Ca_v2.0_10360	Ca4_v2.0	23247016	23252923
XLOC_010287	Ca_v2.0_10379	Ca4_v2.0	23601631	23604782
XLOC_010288	Ca_v2.0_10384	Ca4_v2.0	23646800	23648268

For Review Only

XLOC_010289	Ca_v2.0_10385	Ca4_v2.0	23659269	23662500
XLOC_010292	Ca_v2.0_10395	Ca4_v2.0	23877631	23881918
XLOC_010293	Ca_v2.0_10397	Ca4_v2.0	24082134	24099917
XLOC_010295	Ca_v2.0_10404	Ca4_v2.0	24222607	24224461
XLOC_010310	-	Ca4_v2.0	25132461	25133998
XLOC_010311	Ca_v2.0_10441	Ca4_v2.0	25137043	25138595
XLOC_010318	Ca_v2.0_10454	Ca4_v2.0	25972810	25976348
XLOC_010328	Ca_v2.0_10467	Ca4_v2.0	26273664	26277694
XLOC_010335	Ca_v2.0_10482	Ca4_v2.0	26624499	26629601
XLOC_010338	Ca_v2.0_10485	Ca4_v2.0	26707069	26708824
XLOC_010340	Ca_v2.0_10492	Ca4_v2.0	27008059	27011361
XLOC_010341	Ca_v2.0_10493	Ca4_v2.0	27022009	27024699
XLOC_010344	Ca_v2.0_10501,Ca_v2.0_105(	Ca4_v2.0	27227227	27236474
XLOC_010349	Ca_v2.0_10512	Ca4_v2.0	27368882	27375242
XLOC_010350	Ca_v2.0_10513	Ca4_v2.0	27375294	27382475
XLOC_010354	Ca_v2.0_10524	Ca4_v2.0	27656623	27663001
XLOC_010355	Ca_v2.0_10531	Ca4_v2.0	28004976	28008133
XLOC_010364	-	Ca4_v2.0	29120597	29123186
XLOC_010366	-	Ca4_v2.0	29212263	29224221
XLOC_010368	Ca_v2.0_10558	Ca4_v2.0	29241134	29244100
XLOC_010370	Ca_v2.0_10562	Ca4_v2.0	29391404	29392385
XLOC_010371	Ca_v2.0_10565	Ca4_v2.0	29467020	29468368
XLOC_010374	Ca_v2.0_10569	Ca4_v2.0	29592219	29595224
XLOC_010377	Ca_v2.0_10572	Ca4_v2.0	29637987	29638711
XLOC_010379	Ca_v2.0_10574	Ca4_v2.0	29658010	29658802
XLOC_010384	Ca_v2.0_10584	Ca4_v2.0	29981959	29992642
XLOC_010387	Ca_v2.0_10597	Ca4_v2.0	30358537	30361867
XLOC_010394	Ca_v2.0_10610	Ca4_v2.0	30898824	30899683
XLOC_010400	Ca_v2.0_10628,Ca_v2.0_106:	Ca4_v2.0	31552458	31558521
XLOC_010403	Ca_v2.0_10635	Ca4_v2.0	31993639	31996546
XLOC_010434	Ca_v2.0_10708	Ca4_v2.0	34876636	34884719
XLOC_010446	Ca_v2.0_10730	Ca4_v2.0	36703847	36730470
XLOC_010461	Ca_v2.0_10762	Ca4_v2.0	37968400	37974277
XLOC_010464	Ca_v2.0_10769	Ca4_v2.0	38220295	38222701
XLOC_010472	-	Ca4_v2.0	38832135	38838974
XLOC_010475	Ca_v2.0_10791	Ca4_v2.0	40362586	40365227
XLOC_010477	Ca_v2.0_10800	Ca4_v2.0	40577213	40585424
XLOC_010480	Ca_v2.0_10807	Ca4_v2.0	41268391	41269024
XLOC_010491	-	Ca4_v2.0	42423435	42424471
XLOC_010494	Ca_v2.0_10833	Ca4_v2.0	42691518	42692519
XLOC_010497	Ca_v2.0_10837	Ca4_v2.0	42940980	42942943
XLOC_010499	Ca_v2.0_10841	Ca4_v2.0	43135081	43137754
XLOC_010500	Ca_v2.0_10842	Ca4_v2.0	43175612	43181127
XLOC_010507	Ca_v2.0_10857	Ca4_v2.0	43834925	43839475
XLOC_010508	Ca_v2.0_10858,Ca_v2.0_108!	Ca4_v2.0	43849120	43857535
XLOC_010514	Ca_v2.0_10867	Ca4_v2.0	44003334	44009655

For Review Only

XLOC_010519	Ca_v2.0_10872	Ca4_v2.0	44122921	44127397
XLOC_010550	Ca_v2.0_10930	Ca4_v2.0	46882106	46885078
XLOC_010551	Ca_v2.0_10932	Ca4_v2.0	46957512	46959411
XLOC_010553	Ca_v2.0_10934	Ca4_v2.0	46995597	46997309
XLOC_010565	Ca_v2.0_10958	Ca4_v2.0	47815725	47823803
XLOC_010572	Ca_v2.0_10968	Ca4_v2.0	48210753	48229125
XLOC_010579	-	Ca4_v2.0	48857868	48859596
XLOC_010581	Ca_v2.0_10985	Ca4_v2.0	49063141	49063917
XLOC_010582	Ca_v2.0_10987	Ca4_v2.0	49139444	49143243
XLOC_010583	Ca_v2.0_10988	Ca4_v2.0	49157269	49167113
XLOC_010584	Ca_v2.0_10990	Ca4_v2.0	49220830	49223046
XLOC_010601	Ca_v2.0_11015	Ca4_v2.0	49936271	49947543
XLOC_010618	Ca_v2.0_11051	Ca4_v2.0	50684671	50687065
XLOC_010620	Ca_v2.0_11056	Ca4_v2.0	50858215	50860116
XLOC_010624	Ca_v2.0_11065	Ca4_v2.0	50998499	51005497
XLOC_010625	Ca_v2.0_11066	Ca4_v2.0	51074568	51077552
XLOC_010628	Ca_v2.0_11069	Ca4_v2.0	51135617	51138254
XLOC_010629	Ca_v2.0_11071	Ca4_v2.0	51169797	51216738
XLOC_010633	Ca_v2.0_11076	Ca4_v2.0	51452952	51461337
XLOC_010642	Ca_v2.0_11095	Ca4_v2.0	51930794	51935349
XLOC_010644	Ca_v2.0_11098,Ca_v2.0_110!	Ca4_v2.0	52007261	52015610
XLOC_010655	Ca_v2.0_11117	Ca4_v2.0	52451561	52453346
XLOC_010660	Ca_v2.0_11127	Ca4_v2.0	52741420	52745865
XLOC_010661	Ca_v2.0_11128	Ca4_v2.0	52777502	52880401
XLOC_010676	Ca_v2.0_11156	Ca4_v2.0	53465701	53469007
XLOC_010684	Ca_v2.0_11168	Ca4_v2.0	53679700	53681485
XLOC_010686	Ca_v2.0_11171	Ca4_v2.0	53745119	53750794
XLOC_010698	Ca_v2.0_11197	Ca4_v2.0	54026113	54041420
XLOC_010726	Ca_v2.0_11254	Ca4_v2.0	55336127	55338857
XLOC_010727	Ca_v2.0_11255	Ca4_v2.0	55347466	55351002
XLOC_010728	Ca_v2.0_11257	Ca4_v2.0	55366990	55370952
XLOC_010734	Ca_v2.0_11268	Ca4_v2.0	55559745	55566949
XLOC_010739	Ca_v2.0_11276	Ca4_v2.0	55633523	55646579
XLOC_010741	Ca_v2.0_11280	Ca4_v2.0	55669876	55671700
XLOC_010742	Ca_v2.0_11282	Ca4_v2.0	55681527	55685999
XLOC_010744	Ca_v2.0_11285	Ca4_v2.0	55718514	55727545
XLOC_010751	Ca_v2.0_11301	Ca4_v2.0	56029948	56033479
XLOC_010759	Ca_v2.0_11313	Ca4_v2.0	56173922	56178564
XLOC_010762	Ca_v2.0_11317	Ca4_v2.0	56250126	56255613
XLOC_010765	Ca_v2.0_11320	Ca4_v2.0	56308028	56313810
XLOC_010768	Ca_v2.0_11332	Ca4_v2.0	56458545	56466727
XLOC_010771	Ca_v2.0_11337	Ca4_v2.0	56536331	56544896
XLOC_010772	Ca_v2.0_11341	Ca4_v2.0	56588866	56590671
XLOC_010777	-	Ca4_v2.0	56845148	56846344
XLOC_010785	Ca_v2.0_11370	Ca4_v2.0	57010710	57020020
XLOC_010788	Ca_v2.0_11377	Ca4_v2.0	57088021	57091903

For Review Only

XLOC_010791	Ca_v2.0_11382	Ca4_v2.0	57193744	57200047
XLOC_010792	Ca_v2.0_11384	Ca4_v2.0	57241635	57247859
XLOC_010793	Ca_v2.0_11388	Ca4_v2.0	57305552	57334080
XLOC_010797	Ca_v2.0_11394	Ca4_v2.0	57628740	57634504
XLOC_010799	Ca_v2.0_11398	Ca4_v2.0	57709148	57713689
XLOC_010804	Ca_v2.0_11404	Ca4_v2.0	57783323	57785093
XLOC_010809	Ca_v2.0_11410	Ca4_v2.0	57884382	57888184
XLOC_010810	Ca_v2.0_11411	Ca4_v2.0	57892572	57897033
XLOC_010811	Ca_v2.0_11412	Ca4_v2.0	57900589	57903110
XLOC_010812	Ca_v2.0_11413	Ca4_v2.0	57939838	57940869
XLOC_010813	Ca_v2.0_11416	Ca4_v2.0	58026355	58028298
XLOC_010831	Ca_v2.0_11450	Ca4_v2.0	58400257	58402754
XLOC_010832	Ca_v2.0_11451	Ca4_v2.0	58406466	58415687
XLOC_010837	Ca_v2.0_11461	Ca4_v2.0	58579897	58582744
XLOC_010843	Ca_v2.0_11471	Ca4_v2.0	58702955	58706271
XLOC_010852	Ca_v2.0_11485	Ca4_v2.0	58954110	58959163
XLOC_010858	Ca_v2.0_11497	Ca4_v2.0	59078875	59081995
XLOC_010864	Ca_v2.0_11506	Ca4_v2.0	59174449	59178758
XLOC_010866	Ca_v2.0_11508	Ca4_v2.0	59195370	59205899
XLOC_010881	Ca_v2.0_11538	Ca4_v2.0	59515371	59544496
XLOC_010882	Ca_v2.0_11542	Ca4_v2.0	59558294	59560818
XLOC_010887	Ca_v2.0_11550	Ca4_v2.0	59681909	59683917
XLOC_010890	Ca_v2.0_11555	Ca4_v2.0	59716282	59718697
XLOC_010891	Ca_v2.0_11559	Ca4_v2.0	59825326	59855200
XLOC_010892	Ca_v2.0_11562	Ca4_v2.0	59866928	59870134
XLOC_010904	Ca_v2.0_11586	Ca4_v2.0	60171063	60174733
XLOC_010911	Ca_v2.0_11598	Ca4_v2.0	60316735	60320338
XLOC_010921	Ca_v2.0_11614	Ca4_v2.0	60488528	60489225
XLOC_010923	Ca_v2.0_11617	Ca4_v2.0	60529615	60531086
XLOC_010926	Ca_v2.0_11621	Ca4_v2.0	60586562	60590028
XLOC_010940	Ca_v2.0_11654	Ca4_v2.0	60935065	60938459
XLOC_010943	Ca_v2.0_11657	Ca4_v2.0	60953181	60957964
XLOC_010947	Ca_v2.0_11662	Ca4_v2.0	61054754	61056574
XLOC_010954	Ca_v2.0_11675	Ca4_v2.0	61162065	61163576
XLOC_010956	Ca_v2.0_11679	Ca4_v2.0	61228143	61233180
XLOC_010964	Ca_v2.0_11694	Ca4_v2.0	61437377	61438497
XLOC_010966	Ca_v2.0_11698	Ca4_v2.0	61462686	61465950
XLOC_010972	Ca_v2.0_11707	Ca4_v2.0	61578317	61586763
XLOC_010986	Ca_v2.0_11734	Ca4_v2.0	61749211	61758187
XLOC_010991	Ca_v2.0_11746	Ca4_v2.0	61911737	61917680
XLOC_010992	Ca_v2.0_11748	Ca4_v2.0	61941800	61943518
XLOC_011005	Ca_v2.0_11770	Ca4_v2.0	62193918	62194901
XLOC_011007	Ca_v2.0_11772	Ca4_v2.0	62211855	62214035
XLOC_011011	Ca_v2.0_11780	Ca4_v2.0	62329142	62331485
XLOC_011015	Ca_v2.0_11788	Ca4_v2.0	62407061	62413748
XLOC_011017	Ca_v2.0_11793	Ca4_v2.0	62470335	62472048

XLOC_011022	Ca_v2.0_11800	Ca4_v2.0	62630614	62633073
XLOC_011031	Ca_v2.0_11816	Ca4_v2.0	62824297	62825661
XLOC_011039	Ca_v2.0_11827	Ca4_v2.0	62954433	62956733
XLOC_011050	Ca_v2.0_11846	Ca4_v2.0	63122866	63123207
XLOC_011051	Ca_v2.0_11847	Ca4_v2.0	63128731	63129283
XLOC_011052	Ca_v2.0_11848	Ca4_v2.0	63142370	63150248
XLOC_011053	Ca_v2.0_11853	Ca4_v2.0	63179711	63188114
XLOC_011055	Ca_v2.0_11855	Ca4_v2.0	63191743	63196604
XLOC_011059	Ca_v2.0_11862	Ca4_v2.0	63258243	63268968
XLOC_011061	Ca_v2.0_11865	Ca4_v2.0	63686491	63688799
XLOC_011066	-	Ca4_v2.0	63743904	63746302
XLOC_011068	Ca_v2.0_11874	Ca4_v2.0	63769249	63773842
XLOC_011071	Ca_v2.0_11878	Ca4_v2.0	63821580	63823775
XLOC_011073	Ca_v2.0_11884	Ca4_v2.0	63935046	63949777
XLOC_011074	Ca_v2.0_11887	Ca4_v2.0	63987698	63989347
XLOC_011096	Ca_v2.0_11926	Ca4_v2.0	64418531	64420020
XLOC_011108	Ca_v2.0_11950	Ca4_v2.0	64637105	64644616
XLOC_011118	Ca_v2.0_11969	Ca4_v2.0	64797372	64828310
XLOC_011123	Ca_v2.0_11977	Ca4_v2.0	64926262	64927403
XLOC_011127	Ca_v2.0_11983	Ca4_v2.0	64992121	64995566
XLOC_011128	Ca_v2.0_11985	Ca4_v2.0	65013927	65019442
XLOC_011134	Ca_v2.0_11993	Ca4_v2.0	65130916	65141974
XLOC_011146	Ca_v2.0_12016	Ca4_v2.0	65582675	65583927
XLOC_011150	Ca_v2.0_12023	Ca4_v2.0	65662277	65676690
XLOC_011151	Ca_v2.0_12026	Ca4_v2.0	65691693	65697046
XLOC_011152	Ca_v2.0_12027	Ca4_v2.0	65702338	65704974
XLOC_011167	Ca_v2.0_08423	Ca4_v2.0	223160	227322
XLOC_011191	Ca_v2.0_08461	Ca4_v2.0	617504	637333
XLOC_011192	Ca_v2.0_08462	Ca4_v2.0	652561	658774
XLOC_011193	Ca_v2.0_08463	Ca4_v2.0	659011	664440
XLOC_011195	Ca_v2.0_08467	Ca4_v2.0	699669	700703
XLOC_011199	Ca_v2.0_08475	Ca4_v2.0	761752	763209
XLOC_011209	Ca_v2.0_08490	Ca4_v2.0	888776	893967
XLOC_011224	Ca_v2.0_08516	Ca4_v2.0	1141382	1143593
XLOC_011225	Ca_v2.0_08518	Ca4_v2.0	1155034	1161578
XLOC_011226	Ca_v2.0_08519	Ca4_v2.0	1172928	1175607
XLOC_011234	Ca_v2.0_08535,Ca_v2.0_085:	Ca4_v2.0	1334853	1346910
XLOC_011250	Ca_v2.0_08568	Ca4_v2.0	1693693	1700227
XLOC_011254	Ca_v2.0_08574	Ca4_v2.0	1764925	1769481
XLOC_011266	Ca_v2.0_08605	Ca4_v2.0	2089009	2092031
XLOC_011268	Ca_v2.0_08607	Ca4_v2.0	2100661	2102514
XLOC_011269	Ca_v2.0_08608	Ca4_v2.0	2109534	2111206
XLOC_011274	Ca_v2.0_08614	Ca4_v2.0	2156611	2168028
XLOC_011283	Ca_v2.0_08626	Ca4_v2.0	2369702	2373857
XLOC_011288	Ca_v2.0_08633	Ca4_v2.0	2453840	2455275
XLOC_011290	Ca_v2.0_08637	Ca4_v2.0	2481561	2485395

For Review Only

XLOC_011292	Ca_v2.0_08639	Ca4_v2.0	2502385	2504568
XLOC_011299	Ca_v2.0_08650	Ca4_v2.0	2587444	2591443
XLOC_011300	Ca_v2.0_08652	Ca4_v2.0	2600133	2600748
XLOC_011303	Ca_v2.0_08656	Ca4_v2.0	2651773	2653587
XLOC_011306	Ca_v2.0_08662	Ca4_v2.0	2706589	2713442
XLOC_011307	Ca_v2.0_08663	Ca4_v2.0	2718356	2720001
XLOC_011310	Ca_v2.0_08668	Ca4_v2.0	2810655	2814242
XLOC_011319	Ca_v2.0_08687	Ca4_v2.0	2969314	2977118
XLOC_011322	Ca_v2.0_08692	Ca4_v2.0	3013247	3017340
XLOC_011337	-	Ca4_v2.0	3206171	3207382
XLOC_011338	Ca_v2.0_08712	Ca4_v2.0	3211787	3219088
XLOC_011341	Ca_v2.0_08719	Ca4_v2.0	3266180	3268432
XLOC_011345	Ca_v2.0_08724	Ca4_v2.0	3309114	3312092
XLOC_011352	Ca_v2.0_08734	Ca4_v2.0	3444956	3446959
XLOC_011355	Ca_v2.0_08742	Ca4_v2.0	3513743	3515758
XLOC_011358	Ca_v2.0_08746	Ca4_v2.0	3553304	3559370
XLOC_011359	Ca_v2.0_08748	Ca4_v2.0	3569940	3576161
XLOC_011361	Ca_v2.0_08754	Ca4_v2.0	3644431	3645294
XLOC_011368	-	Ca4_v2.0	3706824	3712687
XLOC_011375	Ca_v2.0_08777	Ca4_v2.0	3816124	3826507
XLOC_011378	Ca_v2.0_08788	Ca4_v2.0	3939687	3942967
XLOC_011389	Ca_v2.0_08811	Ca4_v2.0	4224831	4228941
XLOC_011399	Ca_v2.0_08826	Ca4_v2.0	4373327	4375532
XLOC_011403	Ca_v2.0_08833	Ca4_v2.0	4432350	4434160
XLOC_011404	Ca_v2.0_08835	Ca4_v2.0	4447169	4449558
XLOC_011407	Ca_v2.0_08840	Ca4_v2.0	4499343	4500738
XLOC_011410	Ca_v2.0_08843	Ca4_v2.0	4537488	4539084
XLOC_011412	Ca_v2.0_08847	Ca4_v2.0	4575242	4579759
XLOC_011415	Ca_v2.0_08851	Ca4_v2.0	4619249	4622925
XLOC_011421	Ca_v2.0_08859	Ca4_v2.0	4734547	4737198
XLOC_011429	Ca_v2.0_08871	Ca4_v2.0	4821342	4822176
XLOC_011435	Ca_v2.0_08879	Ca4_v2.0	4888962	4890834
XLOC_011442	Ca_v2.0_08892	Ca4_v2.0	4988868	4990571
XLOC_011446	Ca_v2.0_08899	Ca4_v2.0	5035285	5070681
XLOC_011458	Ca_v2.0_08931	Ca4_v2.0	5332117	5334049
XLOC_011471	Ca_v2.0_08969	Ca4_v2.0	5673428	5675356
XLOC_011477	Ca_v2.0_08982	Ca4_v2.0	5795345	5799311
XLOC_011478	-	Ca4_v2.0	5801768	5802450
XLOC_011487	Ca_v2.0_09002	Ca4_v2.0	5955973	5959909
XLOC_011506	Ca_v2.0_09035	Ca4_v2.0	6265974	6267793
XLOC_011510	Ca_v2.0_09039	Ca4_v2.0	6298379	6303241
XLOC_011511	Ca_v2.0_09040	Ca4_v2.0	6308352	6315956
XLOC_011513	Ca_v2.0_09046	Ca4_v2.0	6344425	6356876
XLOC_011515	Ca_v2.0_09051	Ca4_v2.0	6428602	6432896
XLOC_011532	Ca_v2.0_09084	Ca4_v2.0	6717756	6719221
XLOC_011538	Ca_v2.0_09095	Ca4_v2.0	6791296	6795313

For Review Only

XLOC_011540	Ca_v2.0_09102	Ca4_v2.0	6852121	6857706
XLOC_011547	Ca_v2.0_09121	Ca4_v2.0	7050236	7051405
XLOC_011551	Ca_v2.0_09132	Ca4_v2.0	7186827	7190751
XLOC_011553	Ca_v2.0_09137	Ca4_v2.0	7228450	7236112
XLOC_011554	Ca_v2.0_09139	Ca4_v2.0	7257203	7258245
XLOC_011560	Ca_v2.0_09152,Ca_v2.0_091!	Ca4_v2.0	7386600	7401081
XLOC_011563	Ca_v2.0_09162	Ca4_v2.0	7544271	7545772
XLOC_011567	Ca_v2.0_09171	Ca4_v2.0	7617244	7621272
XLOC_011580	Ca_v2.0_09189	Ca4_v2.0	7787215	7787644
XLOC_011585	Ca_v2.0_09199	Ca4_v2.0	7902126	7902900
XLOC_011590	Ca_v2.0_09208	Ca4_v2.0	7961724	7973897
XLOC_011596	Ca_v2.0_09215	Ca4_v2.0	8042070	8045665
XLOC_011597	Ca_v2.0_09217	Ca4_v2.0	8061094	8064642
XLOC_011609	Ca_v2.0_09236	Ca4_v2.0	8217390	8320225
XLOC_011611	Ca_v2.0_09240	Ca4_v2.0	8217390	8320225
XLOC_011612	-	Ca4_v2.0	8217390	8320225
XLOC_011613	Ca_v2.0_09242	Ca4_v2.0	8217390	8320225
XLOC_011626	Ca_v2.0_09272	Ca4_v2.0	8493083	8517832
XLOC_011627	Ca_v2.0_09273	Ca4_v2.0	8520273	8525559
XLOC_011644	Ca_v2.0_09299	Ca4_v2.0	8784599	8789348
XLOC_011647	Ca_v2.0_09304	Ca4_v2.0	8832164	8837248
XLOC_011652	Ca_v2.0_09310	Ca4_v2.0	8928693	8949349
XLOC_011655	Ca_v2.0_09314	Ca4_v2.0	8987349	8990898
XLOC_011657	Ca_v2.0_09321	Ca4_v2.0	9129124	9131741
XLOC_011666	Ca_v2.0_09336	Ca4_v2.0	9280940	9284690
XLOC_011667	Ca_v2.0_09337	Ca4_v2.0	9288639	9291124
XLOC_011668	Ca_v2.0_09339	Ca4_v2.0	9309469	9311332
XLOC_011671	Ca_v2.0_09343	Ca4_v2.0	9370050	9381149
XLOC_011676	Ca_v2.0_09352	Ca4_v2.0	9504653	9513516
XLOC_011681	Ca_v2.0_09373	Ca4_v2.0	9735930	9745582
XLOC_011688	Ca_v2.0_09385	Ca4_v2.0	9882089	10147454
XLOC_011696	-	Ca4_v2.0	10151849	10155113
XLOC_011699	Ca_v2.0_09409	Ca4_v2.0	10220964	10222636
XLOC_011706	Ca_v2.0_09422	Ca4_v2.0	10332814	10334484
XLOC_011709	Ca_v2.0_09427	Ca4_v2.0	10402782	10403497
XLOC_011710	Ca_v2.0_09428	Ca4_v2.0	10415162	10420634
XLOC_011714	Ca_v2.0_09437	Ca4_v2.0	10503197	10510776
XLOC_011718	Ca_v2.0_09448	Ca4_v2.0	10595111	10595969
XLOC_011749	Ca_v2.0_09490	Ca4_v2.0	11043540	11053833
XLOC_011751	Ca_v2.0_09493	Ca4_v2.0	11065795	11075184
XLOC_011753	Ca_v2.0_09496	Ca4_v2.0	11091171	11098170
XLOC_011755	Ca_v2.0_09504	Ca4_v2.0	11149994	11156343
XLOC_011756	Ca_v2.0_09505	Ca4_v2.0	11163862	11165574
XLOC_011759	Ca_v2.0_09512	Ca4_v2.0	11200709	11203469
XLOC_011762	Ca_v2.0_09516	Ca4_v2.0	11245127	11247332
XLOC_011764	Ca_v2.0_09518	Ca4_v2.0	11257607	11279092

For Review Only

XLOC_011766	Ca_v2.0_09522	Ca4_v2.0	11303324	11305318
XLOC_011772	Ca_v2.0_09532	Ca4_v2.0	11497071	11497805
XLOC_011774	Ca_v2.0_09536	Ca4_v2.0	11561833	11564219
XLOC_011789	Ca_v2.0_09566	Ca4_v2.0	11850668	11852332
XLOC_011797	-	Ca4_v2.0	12057514	12059369
XLOC_011803	Ca_v2.0_09585	Ca4_v2.0	12141013	12145654
XLOC_011811	Ca_v2.0_09596	Ca4_v2.0	12285367	12288495
XLOC_011814	Ca_v2.0_09600	Ca4_v2.0	12321295	12326719
XLOC_011815	Ca_v2.0_09602	Ca4_v2.0	12326829	12331903
XLOC_011825	Ca_v2.0_09629	Ca4_v2.0	12573633	12579689
XLOC_011840	Ca_v2.0_09658	Ca4_v2.0	12863115	12866238
XLOC_011841	Ca_v2.0_09659	Ca4_v2.0	12874664	12877367
XLOC_011842	Ca_v2.0_09661	Ca4_v2.0	12889605	12891883
XLOC_011845	Ca_v2.0_09665	Ca4_v2.0	12935661	12942922
XLOC_011848	Ca_v2.0_09669	Ca4_v2.0	12965460	12967469
XLOC_011849	Ca_v2.0_09672	Ca4_v2.0	13000612	13010112
XLOC_011852	Ca_v2.0_09677	Ca4_v2.0	13067791	13068851
XLOC_011879	-	Ca4_v2.0	13607379	13608318
XLOC_011880	Ca_v2.0_09731	Ca4_v2.0	13627540	13632552
XLOC_011884	Ca_v2.0_09742	Ca4_v2.0	13761732	13768991
XLOC_011885	Ca_v2.0_09747	Ca4_v2.0	13799747	13801129
XLOC_011925	Ca_v2.0_09816	Ca4_v2.0	14419951	14425325
XLOC_011934	Ca_v2.0_09829	Ca4_v2.0	14544267	14549115
XLOC_011937	Ca_v2.0_09832	Ca4_v2.0	14562561	14567811
XLOC_011938	-	Ca4_v2.0	14568556	14571146
XLOC_011951	Ca_v2.0_09858	Ca4_v2.0	14786011	14787497
XLOC_011958	Ca_v2.0_09875	Ca4_v2.0	14957722	14960638
XLOC_011968	-	Ca4_v2.0	15072916	15074378
XLOC_011969	Ca_v2.0_09891	Ca4_v2.0	15101760	15103327
XLOC_011972	Ca_v2.0_09898	Ca4_v2.0	15171750	15173034
XLOC_011982	Ca_v2.0_09918	Ca4_v2.0	15400066	15404384
XLOC_011986	Ca_v2.0_09922	Ca4_v2.0	15446803	15466514
XLOC_011989	Ca_v2.0_09925	Ca4_v2.0	15515381	15521858
XLOC_011993	Ca_v2.0_09933	Ca4_v2.0	15596090	15606477
XLOC_011996	Ca_v2.0_09936	Ca4_v2.0	15632423	15634227
XLOC_011998	Ca_v2.0_09939	Ca4_v2.0	15683770	15687558
XLOC_012000	Ca_v2.0_09948	Ca4_v2.0	15782914	15785159
XLOC_012004	Ca_v2.0_09955	Ca4_v2.0	15892450	15894423
XLOC_012006	Ca_v2.0_09960	Ca4_v2.0	15937506	15939834
XLOC_012007	Ca_v2.0_09962	Ca4_v2.0	15965197	15966174
XLOC_012032	Ca_v2.0_10025	Ca4_v2.0	16747181	16750119
XLOC_012033	Ca_v2.0_10026	Ca4_v2.0	16754125	16760706
XLOC_012035	Ca_v2.0_10029	Ca4_v2.0	16785306	16792637
XLOC_012036	Ca_v2.0_10031	Ca4_v2.0	16833656	16837253
XLOC_012052	Ca_v2.0_10058	Ca4_v2.0	17099784	17101801
XLOC_012057	Ca_v2.0_10065	Ca4_v2.0	17176783	17182444

For Review Only

XLOC_012059	Ca_v2.0_10067	Ca4_v2.0	17219282	17227673
XLOC_012062	Ca_v2.0_10071	Ca4_v2.0	17309368	17313426
XLOC_012063	Ca_v2.0_10073	Ca4_v2.0	17324645	17330364
XLOC_012065	Ca_v2.0_10075	Ca4_v2.0	17339184	17349390
XLOC_012070	Ca_v2.0_10094	Ca4_v2.0	17528254	17531982
XLOC_012071	Ca_v2.0_10095	Ca4_v2.0	17534683	17536403
XLOC_012079	Ca_v2.0_10110	Ca4_v2.0	17682257	17683176
XLOC_012080	Ca_v2.0_10111	Ca4_v2.0	17699656	17701640
XLOC_012097	Ca_v2.0_10134	Ca4_v2.0	17848421	17849249
XLOC_012103	Ca_v2.0_10144	Ca4_v2.0	17986183	17989708
XLOC_012104	Ca_v2.0_10145	Ca4_v2.0	18023613	18025723
XLOC_012128	Ca_v2.0_10191	Ca4_v2.0	18893113	18896720
XLOC_012144	Ca_v2.0_10214	Ca4_v2.0	19202765	19204079
XLOC_012151	Ca_v2.0_10232	Ca4_v2.0	19445731	19452229
XLOC_012152	-	Ca4_v2.0	19461768	19462946
XLOC_012156	Ca_v2.0_10238	Ca4_v2.0	19552528	19562855
XLOC_012163	Ca_v2.0_10250	Ca4_v2.0	19730505	19734658
XLOC_012164	Ca_v2.0_10251	Ca4_v2.0	19742850	19746536
XLOC_012167	-	Ca4_v2.0	19869486	19872750
XLOC_012173	Ca_v2.0_10268	Ca4_v2.0	20101785	20106499
XLOC_012183	Ca_v2.0_10288	Ca4_v2.0	20405054	20405791
XLOC_012186	Ca_v2.0_10292	Ca4_v2.0	20518629	20520919
XLOC_012193	Ca_v2.0_10311	Ca4_v2.0	20941188	20944244
XLOC_012198	Ca_v2.0_10319	Ca4_v2.0	21160468	21165364
XLOC_012207	-	Ca4_v2.0	22213611	22214487
XLOC_012221	Ca_v2.0_10346	Ca4_v2.0	22761481	22763495
XLOC_012226	Ca_v2.0_10354	Ca4_v2.0	22885794	22892209
XLOC_012228	Ca_v2.0_10356	Ca4_v2.0	23043089	23048437
XLOC_012234	Ca_v2.0_10367	Ca4_v2.0	23334250	23340785
XLOC_012235	Ca_v2.0_10369	Ca4_v2.0	23355395	23363528
XLOC_012238	Ca_v2.0_10374	Ca4_v2.0	23507181	23514763
XLOC_012246	Ca_v2.0_10388	Ca4_v2.0	23715759	23716830
XLOC_012247	Ca_v2.0_10389	Ca4_v2.0	23725517	23731220
XLOC_012252	Ca_v2.0_10398	Ca4_v2.0	24102557	24107232
XLOC_012255	Ca_v2.0_10403	Ca4_v2.0	24181887	24186796
XLOC_012263	Ca_v2.0_10418	Ca4_v2.0	24423412	24424910
XLOC_012265	Ca_v2.0_10422,Ca_v2.0_104:	Ca4_v2.0	24468676	24472850
XLOC_012281	Ca_v2.0_10448	Ca4_v2.0	25787913	25789341
XLOC_012291	Ca_v2.0_10475	Ca4_v2.0	26441846	26442462
XLOC_012301	Ca_v2.0_10491	Ca4_v2.0	26978771	26990712
XLOC_012305	Ca_v2.0_10499	Ca4_v2.0	27129212	27133032
XLOC_012309	Ca_v2.0_10507	Ca4_v2.0	27316172	27320356
XLOC_012311	Ca_v2.0_10509	Ca4_v2.0	27332741	27333852
XLOC_012314	Ca_v2.0_10513	Ca4_v2.0	27375294	27382475
XLOC_012316	Ca_v2.0_10514,Ca_v2.0_105	Ca4_v2.0	27448921	27454614
XLOC_012318	Ca_v2.0_10519	Ca4_v2.0	27599558	27601789

For Review Only

XLOC_012340	Ca_v2.0_10550	Ca4_v2.0	28805822	28832589
XLOC_012342	Ca_v2.0_10553	Ca4_v2.0	28909164	28911593
XLOC_012344	Ca_v2.0_10554	Ca4_v2.0	29055110	29057230
XLOC_012347	Ca_v2.0_10560	Ca4_v2.0	29341045	29341961
XLOC_012350	-	Ca4_v2.0	29422258	29424716
XLOC_012353	Ca_v2.0_10567	Ca4_v2.0	29513230	29518735
XLOC_012357	Ca_v2.0_10584	Ca4_v2.0	29981959	29992642
XLOC_012366	Ca_v2.0_10595	Ca4_v2.0	30328899	30332942
XLOC_012374	Ca_v2.0_10609	Ca4_v2.0	30877160	30885173
XLOC_012386	Ca_v2.0_10626,Ca_v2.0_106	Ca4_v2.0	31474924	31482105
XLOC_012410	Ca_v2.0_10668	Ca4_v2.0	33037573	33041429
XLOC_012418	Ca_v2.0_10679	Ca4_v2.0	33491042	33492685
XLOC_012457	-	Ca4_v2.0	37333183	37359326
XLOC_012466	Ca_v2.0_10760	Ca4_v2.0	37919530	37921356
XLOC_012505	-	Ca4_v2.0	42378029	42379631
XLOC_012509	Ca_v2.0_10833	Ca4_v2.0	42691518	42692519
XLOC_012519	Ca_v2.0_10852	Ca4_v2.0	43488866	43491698
XLOC_012535	Ca_v2.0_10893	Ca4_v2.0	45573047	45579395
XLOC_012536	Ca_v2.0_10894	Ca4_v2.0	45731852	45747732
XLOC_012539	-	Ca4_v2.0	45793345	45796310
XLOC_012542	Ca_v2.0_10904	Ca4_v2.0	45926515	45939392
XLOC_012548	Ca_v2.0_10915	Ca4_v2.0	46220058	46221139
XLOC_012564	Ca_v2.0_10950	Ca4_v2.0	47689706	47694142
XLOC_012569	Ca_v2.0_10961	Ca4_v2.0	47912593	47914877
XLOC_012570	Ca_v2.0_10962	Ca4_v2.0	47982276	47983976
XLOC_012571	Ca_v2.0_10967	Ca4_v2.0	48201122	48203077
XLOC_012572	Ca_v2.0_10969	Ca4_v2.0	48210753	48229125
XLOC_012574	Ca_v2.0_10972	Ca4_v2.0	48357378	48357799
XLOC_012580	Ca_v2.0_10980	Ca4_v2.0	48921511	48922131
XLOC_012581	Ca_v2.0_10981	Ca4_v2.0	48941249	48947763
XLOC_012611	Ca_v2.0_11042	Ca4_v2.0	50509122	50511033
XLOC_012616	Ca_v2.0_11052	Ca4_v2.0	50713642	50725505
XLOC_012625	Ca_v2.0_11066	Ca4_v2.0	51074568	51077552
XLOC_012642	Ca_v2.0_11103	Ca4_v2.0	52082353	52084300
XLOC_012644	Ca_v2.0_11109	Ca4_v2.0	52264462	52268459
XLOC_012661	Ca_v2.0_11144	Ca4_v2.0	53186521	53189327
XLOC_012669	Ca_v2.0_11158	Ca4_v2.0	53483044	53488584
XLOC_012673	Ca_v2.0_11168	Ca4_v2.0	53679700	53681485
XLOC_012674	Ca_v2.0_11170	Ca4_v2.0	53717812	53721045
XLOC_012675	Ca_v2.0_11172	Ca4_v2.0	53777503	53779207
XLOC_012684	Ca_v2.0_11189	Ca4_v2.0	53947551	53954353
XLOC_012699	Ca_v2.0_11213	Ca4_v2.0	54634770	54638691
XLOC_012705	Ca_v2.0_11224	Ca4_v2.0	54783075	54827327
XLOC_012710	Ca_v2.0_11237	Ca4_v2.0	55093443	55102843
XLOC_012715	Ca_v2.0_11243	Ca4_v2.0	55188277	55200524
XLOC_012722	Ca_v2.0_11256	Ca4_v2.0	55352399	55354898

For Review Only

XLOC_012733	Ca_v2.0_11281	Ca4_v2.0	55673917	55676234
XLOC_012734	Ca_v2.0_11284	Ca4_v2.0	55710656	55713348
XLOC_012737	Ca_v2.0_11291	Ca4_v2.0	55835874	55836984
XLOC_012739	Ca_v2.0_11293	Ca4_v2.0	55864667	55871704
XLOC_012741	Ca_v2.0_11296	Ca4_v2.0	55941411	55943833
XLOC_012745	Ca_v2.0_11300	Ca4_v2.0	55982852	55985465
XLOC_012749	Ca_v2.0_11312	Ca4_v2.0	56167121	56171544
XLOC_012761	Ca_v2.0_11334	Ca4_v2.0	56478709	56480762
XLOC_012769	Ca_v2.0_11345	Ca4_v2.0	56639119	56642312
XLOC_012773	Ca_v2.0_11350	Ca4_v2.0	56700127	56702002
XLOC_012774	Ca_v2.0_11351	Ca4_v2.0	56711467	56712415
XLOC_012779	Ca_v2.0_11358	Ca4_v2.0	56797409	56801642
XLOC_012788	Ca_v2.0_11376	Ca4_v2.0	57080626	57083264
XLOC_012790	-	Ca4_v2.0	57120483	57121258
XLOC_012792	Ca_v2.0_11383	Ca4_v2.0	57193744	57200047
XLOC_012795	Ca_v2.0_11387	Ca4_v2.0	57291835	57294821
XLOC_012801	Ca_v2.0_11393	Ca4_v2.0	57626234	57628421
XLOC_012802	Ca_v2.0_11396	Ca4_v2.0	57644486	57657519
XLOC_012806	Ca_v2.0_11411	Ca4_v2.0	57892572	57897033
XLOC_012809	Ca_v2.0_11415	Ca4_v2.0	57999304	58002820
XLOC_012815	Ca_v2.0_11433	Ca4_v2.0	58211905	58289748
XLOC_012822	Ca_v2.0_11444	Ca4_v2.0	58322529	58329004
XLOC_012824	Ca_v2.0_11451	Ca4_v2.0	58406466	58415687
XLOC_012825	Ca_v2.0_11452	Ca4_v2.0	58418977	58422968
XLOC_012826	Ca_v2.0_11453	Ca4_v2.0	58427484	58430613
XLOC_012836	Ca_v2.0_11474	Ca4_v2.0	58749613	58754194
XLOC_012840	-	Ca4_v2.0	58929226	58934134
XLOC_012841	Ca_v2.0_11486	Ca4_v2.0	58954110	58959163
XLOC_012842	Ca_v2.0_11487	Ca4_v2.0	58966185	58969136
XLOC_012847	Ca_v2.0_11501	Ca4_v2.0	59127769	59130542
XLOC_012848	Ca_v2.0_11503,Ca_v2.0_115(	Ca4_v2.0	59141838	59145922
XLOC_012849	Ca_v2.0_11509	Ca4_v2.0	59195370	59205899
XLOC_012852	Ca_v2.0_11515	Ca4_v2.0	59247218	59256510
XLOC_012853	Ca_v2.0_11517	Ca4_v2.0	59266813	59268227
XLOC_012861	Ca_v2.0_11533	Ca4_v2.0	59442970	59457747
XLOC_012864	Ca_v2.0_11539,Ca_v2.0_115	Ca4_v2.0	59515371	59544496
XLOC_012878	Ca_v2.0_11563	Ca4_v2.0	59873645	59876245
XLOC_012889	Ca_v2.0_11583	Ca4_v2.0	60120421	60124232
XLOC_012897	Ca_v2.0_11600	Ca4_v2.0	60335893	60338559
XLOC_012904	Ca_v2.0_11617	Ca4_v2.0	60529615	60531086
XLOC_012906	Ca_v2.0_11625	Ca4_v2.0	60642815	60644882
XLOC_012911	Ca_v2.0_11633	Ca4_v2.0	60682693	60683440
XLOC_012913	Ca_v2.0_11636	Ca4_v2.0	60695735	60700184
XLOC_012917	Ca_v2.0_11643	Ca4_v2.0	60769993	60771756
XLOC_012919	Ca_v2.0_11645	Ca4_v2.0	60811960	60813976
XLOC_012920	-	Ca4_v2.0	60832994	60836404

For Review Only

XLOC_012922	Ca_v2.0_11647	Ca4_v2.0	60854830	60860860
XLOC_012926	Ca_v2.0_11651	Ca4_v2.0	60899143	60899983
XLOC_012931	-	Ca4_v2.0	61035781	61037131
XLOC_012932	Ca_v2.0_11662	Ca4_v2.0	61054754	61056574
XLOC_012934	Ca_v2.0_11665	Ca4_v2.0	61073409	61076764
XLOC_012935	Ca_v2.0_11668	Ca4_v2.0	61100536	61102660
XLOC_012941	Ca_v2.0_11681	Ca4_v2.0	61258509	61258955
XLOC_012946	-	Ca4_v2.0	61370083	61371979
XLOC_012950	Ca_v2.0_11696,Ca_v2.0_116	Ca4_v2.0	61446838	61455440
XLOC_012952	Ca_v2.0_11702	Ca4_v2.0	61501806	61506668
XLOC_012960	Ca_v2.0_11725	Ca4_v2.0	61685248	61689240
XLOC_012965	Ca_v2.0_11734	Ca4_v2.0	61749211	61758187
XLOC_012971	Ca_v2.0_11746	Ca4_v2.0	61911737	61917680
XLOC_012982	Ca_v2.0_11773	Ca4_v2.0	62232346	62235307
XLOC_012990	Ca_v2.0_11787	Ca4_v2.0	62402809	62405441
XLOC_012996	Ca_v2.0_11802	Ca4_v2.0	62635197	62641986
XLOC_012998	Ca_v2.0_11805	Ca4_v2.0	62661906	62663276
XLOC_012999	Ca_v2.0_11810	Ca4_v2.0	62715869	62718243
XLOC_013003	Ca_v2.0_11820	Ca4_v2.0	62871343	62874317
XLOC_013013	Ca_v2.0_11850	Ca4_v2.0	63156038	63156650
XLOC_013014	Ca_v2.0_11851,Ca_v2.0_118	Ca4_v2.0	63161603	63167542
XLOC_013016	Ca_v2.0_11857	Ca4_v2.0	63199812	63203379
XLOC_013017	Ca_v2.0_11861	Ca4_v2.0	63235411	63244483
XLOC_013018	Ca_v2.0_11863	Ca4_v2.0	63258243	63268968
XLOC_013019	Ca_v2.0_11865	Ca4_v2.0	63686491	63688799
XLOC_013024	Ca_v2.0_11878	Ca4_v2.0	63821580	63823775
XLOC_013026	-	Ca4_v2.0	63875951	63887046
XLOC_013033	Ca_v2.0_11885	Ca4_v2.0	63960898	63962525
XLOC_013046	Ca_v2.0_11909	Ca4_v2.0	64279110	64281627
XLOC_013049	Ca_v2.0_11916	Ca4_v2.0	64328821	64333765
XLOC_013050	Ca_v2.0_11920	Ca4_v2.0	64359691	64364142
XLOC_013066	Ca_v2.0_11957	Ca4_v2.0	64697007	64698615
XLOC_013070	Ca_v2.0_11964,Ca_v2.0_119	Ca4_v2.0	64762386	64773918
XLOC_013072	Ca_v2.0_11971	Ca4_v2.0	64797372	64828310
XLOC_013079	Ca_v2.0_11984	Ca4_v2.0	64996716	65003314
XLOC_013081	Ca_v2.0_11986	Ca4_v2.0	65071058	65072190
XLOC_013083	Ca_v2.0_11991	Ca4_v2.0	65095974	65096963
XLOC_013084	Ca_v2.0_11994	Ca4_v2.0	65149071	65154320
XLOC_013086	Ca_v2.0_11997	Ca4_v2.0	65183083	65187623
XLOC_013088	Ca_v2.0_11999	Ca4_v2.0	65216193	65218585
XLOC_013092	Ca_v2.0_12009	Ca4_v2.0	65538900	65541069
XLOC_013100	Ca_v2.0_12022	Ca4_v2.0	65651078	65654169
XLOC_013102	Ca_v2.0_12025	Ca4_v2.0	65682539	65686255
XLOC_013111	-	Ca4_v2.0	1693693	1700227
XLOC_013127	-	Ca4_v2.0	4488266	4488756
XLOC_013128	-	Ca4_v2.0	4690284	4691149

For Review Only

XLOC_013133	-	Ca4_v2.0	7041627	7041945
XLOC_013139	-	Ca4_v2.0	9353472	9354256
XLOC_013143	-	Ca4_v2.0	9882089	10147454
XLOC_013154	-	Ca4_v2.0	13118734	13118982
XLOC_013158	-	Ca4_v2.0	14726243	14726743
XLOC_013160	-	Ca4_v2.0	15364282	15365130
XLOC_013168	-	Ca4_v2.0	16268354	16268747
XLOC_013178	-	Ca4_v2.0	19455604	19456157
XLOC_013180	-	Ca4_v2.0	19931373	19933444
XLOC_013187	-	Ca4_v2.0	22928461	22929217
XLOC_013209	-	Ca4_v2.0	35369359	35369579
XLOC_013211	-	Ca4_v2.0	38611859	38612348
XLOC_013214	-	Ca4_v2.0	42388323	42388502
XLOC_013215	-	Ca4_v2.0	42600036	42600184
XLOC_013217	-	Ca4_v2.0	44671147	44672105
XLOC_013219	-	Ca4_v2.0	46399742	46400260
XLOC_013235	-	Ca4_v2.0	54183751	54184941
XLOC_013238	-	Ca4_v2.0	55919977	55920669
XLOC_013243	-	Ca4_v2.0	58911944	58912436
XLOC_013244	-	Ca4_v2.0	58923719	58924647
XLOC_013245	-	Ca4_v2.0	59112829	59113628
XLOC_013248	-	Ca4_v2.0	61370083	61371979
XLOC_013250	-	Ca4_v2.0	61788243	61790139
XLOC_013256	-	Ca4_v2.0	64573628	64575249
XLOC_013268	Ca_v2.0_12043	Ca5_v2.0	335369	352317
XLOC_013269	Ca_v2.0_12046	Ca5_v2.0	449156	454982
XLOC_013271	Ca_v2.0_12053	Ca5_v2.0	597837	609917
XLOC_013280	Ca_v2.0_12071	Ca5_v2.0	1389972	1484934
XLOC_013281	Ca_v2.0_12074	Ca5_v2.0	1389972	1484934
XLOC_013291	Ca_v2.0_12084	Ca5_v2.0	1689550	1691057
XLOC_013293	Ca_v2.0_12085	Ca5_v2.0	1751338	1753756
XLOC_013295	Ca_v2.0_12088	Ca5_v2.0	1779554	1780342
XLOC_013297	Ca_v2.0_12091	Ca5_v2.0	1809242	1814931
XLOC_013298	Ca_v2.0_12092	Ca5_v2.0	1853051	1855026
XLOC_013306	Ca_v2.0_12111	Ca5_v2.0	2220927	2229271
XLOC_013307	Ca_v2.0_12112	Ca5_v2.0	2250184	2251095
XLOC_013311	Ca_v2.0_12122	Ca5_v2.0	2333008	2338587
XLOC_013312	Ca_v2.0_12129	Ca5_v2.0	2457606	2462616
XLOC_013332	Ca_v2.0_12160	Ca5_v2.0	2991256	2999930
XLOC_013336	Ca_v2.0_12167	Ca5_v2.0	3211004	3215591
XLOC_013349	Ca_v2.0_12189	Ca5_v2.0	3583919	3589394
XLOC_013350	Ca_v2.0_12190,Ca_v2.0_121!	Ca5_v2.0	3603196	3609614
XLOC_013358	Ca_v2.0_12206	Ca5_v2.0	4111377	4115959
XLOC_013359	Ca_v2.0_12208	Ca5_v2.0	4318862	4320159
XLOC_013364	Ca_v2.0_12214	Ca5_v2.0	4448143	4454541
XLOC_013373	Ca_v2.0_12237	Ca5_v2.0	5008554	5020770

For Review Only

XLOC_013375	Ca_v2.0_12242	Ca5_v2.0	5187169	5200559
XLOC_013376	Ca_v2.0_12243	Ca5_v2.0	5205191	5220041
XLOC_013389	Ca_v2.0_12271,Ca_v2.0_1227	Ca5_v2.0	5726714	5737431
XLOC_013400	Ca_v2.0_12303	Ca5_v2.0	6640958	6649642
XLOC_013401	Ca_v2.0_12305	Ca5_v2.0	6675355	6680069
XLOC_013403	Ca_v2.0_12307	Ca5_v2.0	6859670	6862668
XLOC_013404	Ca_v2.0_12309	Ca5_v2.0	6920014	6922353
XLOC_013408	-	Ca5_v2.0	14046124	14047662
XLOC_013409	Ca_v2.0_12320	Ca5_v2.0	14210810	14220363
XLOC_013410	Ca_v2.0_12322	Ca5_v2.0	14344486	14350993
XLOC_013417	Ca_v2.0_12341,Ca_v2.0_1234	Ca5_v2.0	15714599	15717731
XLOC_013421	Ca_v2.0_12349	Ca5_v2.0	15737176	15744354
XLOC_013443	Ca_v2.0_12390	Ca5_v2.0	17402585	17420244
XLOC_013451	Ca_v2.0_12399	Ca5_v2.0	18129415	18133838
XLOC_013457	Ca_v2.0_12412	Ca5_v2.0	18611351	18614184
XLOC_013462	Ca_v2.0_12418	Ca5_v2.0	18770313	18771153
XLOC_013467	Ca_v2.0_12428	Ca5_v2.0	19514368	19517935
XLOC_013479	Ca_v2.0_12452	Ca5_v2.0	20511975	20513137
XLOC_013488	Ca_v2.0_12464	Ca5_v2.0	21462128	21464280
XLOC_013489	Ca_v2.0_12465	Ca5_v2.0	21549204	21555154
XLOC_013490	Ca_v2.0_12467	Ca5_v2.0	21646142	21652022
XLOC_013499	Ca_v2.0_12477	Ca5_v2.0	22615920	22616826
XLOC_013511	Ca_v2.0_12498	Ca5_v2.0	23117414	23120681
XLOC_013514	Ca_v2.0_12504	Ca5_v2.0	23508462	23510521
XLOC_013522	Ca_v2.0_12521	Ca5_v2.0	24277992	24281201
XLOC_013532	Ca_v2.0_12537	Ca5_v2.0	24606968	24607643
XLOC_013552	Ca_v2.0_12569	Ca5_v2.0	25660348	25661720
XLOC_013560	Ca_v2.0_12586	Ca5_v2.0	27663904	27665854
XLOC_013561	Ca_v2.0_12587	Ca5_v2.0	27842645	27844110
XLOC_013588	Ca_v2.0_12641	Ca5_v2.0	32879910	32880501
XLOC_013590	Ca_v2.0_12644	Ca5_v2.0	33119280	33121739
XLOC_013596	Ca_v2.0_12654	Ca5_v2.0	34296028	34304471
XLOC_013598	-	Ca5_v2.0	34341412	34345616
XLOC_013619	Ca_v2.0_12688	Ca5_v2.0	35905884	35907612
XLOC_013625	Ca_v2.0_12703	Ca5_v2.0	37735797	37741609
XLOC_013626	Ca_v2.0_12705	Ca5_v2.0	37825538	37828488
XLOC_013628	-	Ca5_v2.0	37868220	37868747
XLOC_013632	Ca_v2.0_12711	Ca5_v2.0	38198036	38200790
XLOC_013633	Ca_v2.0_12712	Ca5_v2.0	38295368	38298449
XLOC_013636	Ca_v2.0_12715	Ca5_v2.0	38433983	38554971
XLOC_013641	Ca_v2.0_12724	Ca5_v2.0	38857401	38862041
XLOC_013646	Ca_v2.0_12735	Ca5_v2.0	39230505	39240847
XLOC_013661	Ca_v2.0_12763	Ca5_v2.0	41439641	41449254
XLOC_013676	Ca_v2.0_12784	Ca5_v2.0	42405292	42418704
XLOC_013679	Ca_v2.0_12787	Ca5_v2.0	42465466	42488530
XLOC_013684	Ca_v2.0_12795	Ca5_v2.0	42791878	42793961

For Review Only

XLOC_013686	Ca_v2.0_12798	Ca5_v2.0	42892002	42896030
XLOC_013700	Ca_v2.0_12828	Ca5_v2.0	44254318	44269886
XLOC_013705	Ca_v2.0_12842	Ca5_v2.0	44555634	44560893
XLOC_013712	Ca_v2.0_12858	Ca5_v2.0	44962954	45015682
XLOC_013726	Ca_v2.0_12878	Ca5_v2.0	45632031	45637001
XLOC_013732	Ca_v2.0_12888,Ca_v2.0_128	Ca5_v2.0	46410103	46413176
XLOC_013736	Ca_v2.0_12895	Ca5_v2.0	46823909	46828812
XLOC_013742	Ca_v2.0_12906	Ca5_v2.0	47211756	47213948
XLOC_013747	Ca_v2.0_12916	Ca5_v2.0	47582175	47583339
XLOC_013748	Ca_v2.0_12917	Ca5_v2.0	47626341	47631606
XLOC_013750	Ca_v2.0_12923	Ca5_v2.0	47963032	47966454
XLOC_013763	Ca_v2.0_12938	Ca5_v2.0	48242886	48246981
XLOC_013764	Ca_v2.0_12939	Ca5_v2.0	48258760	48259887
XLOC_013769	Ca_v2.0_12947	Ca5_v2.0	48490468	48491289
XLOC_013771	Ca_v2.0_12949	Ca5_v2.0	48569586	48581595
XLOC_013784	Ca_v2.0_12970	Ca5_v2.0	49468431	49470217
XLOC_013796	Ca_v2.0_12996	Ca5_v2.0	50186154	50190932
XLOC_013802	Ca_v2.0_13010	Ca5_v2.0	50401933	50402754
XLOC_013804	Ca_v2.0_13014	Ca5_v2.0	50527114	50532410
XLOC_013805	Ca_v2.0_13016	Ca5_v2.0	50584716	50594072
XLOC_013809	Ca_v2.0_13022	Ca5_v2.0	50697137	50698783
XLOC_013814	Ca_v2.0_13045	Ca5_v2.0	50886881	50892322
XLOC_013819	Ca_v2.0_13061	Ca5_v2.0	51228404	51229306
XLOC_013823	Ca_v2.0_13069	Ca5_v2.0	51470834	51477920
XLOC_013829	Ca_v2.0_13077	Ca5_v2.0	51746500	51748409
XLOC_013846	Ca_v2.0_13100	Ca5_v2.0	52269919	52367966
XLOC_013847	Ca_v2.0_13103	Ca5_v2.0	52269919	52367966
XLOC_013852	Ca_v2.0_13113,Ca_v2.0_131	Ca5_v2.0	52432482	52436041
XLOC_013870	Ca_v2.0_13159	Ca5_v2.0	53603927	53609599
XLOC_013871	Ca_v2.0_13160	Ca5_v2.0	53627734	53628722
XLOC_013874	Ca_v2.0_13163	Ca5_v2.0	53668715	53673704
XLOC_013883	Ca_v2.0_13178	Ca5_v2.0	54055868	54060486
XLOC_013894	Ca_v2.0_13212	Ca5_v2.0	54553251	54617127
XLOC_013895	Ca_v2.0_13206	Ca5_v2.0	54553251	54617127
XLOC_013896	Ca_v2.0_13208	Ca5_v2.0	54553251	54617127
XLOC_013902	Ca_v2.0_13217	Ca5_v2.0	54644259	54648392
XLOC_013904	Ca_v2.0_13222	Ca5_v2.0	54778433	54781074
XLOC_013917	Ca_v2.0_13248	Ca5_v2.0	55359275	55362945
XLOC_013921	Ca_v2.0_13256	Ca5_v2.0	55428859	55430828
XLOC_013922	Ca_v2.0_13258	Ca5_v2.0	55501192	55504385
XLOC_013926	Ca_v2.0_13270	Ca5_v2.0	55631519	55634336
XLOC_013931	Ca_v2.0_13279	Ca5_v2.0	55810634	55812608
XLOC_013939	Ca_v2.0_13291	Ca5_v2.0	56027458	56031812
XLOC_013940	-	Ca5_v2.0	56034665	56035531
XLOC_013947	Ca_v2.0_13303	Ca5_v2.0	56300082	56306602
XLOC_013951	Ca_v2.0_13314	Ca5_v2.0	56480318	56482765

For Review Only

XLOC_013966	Ca_v2.0_13339	Ca5_v2.0	56938107	56947788
XLOC_013968	Ca_v2.0_13344	Ca5_v2.0	57004067	57012561
XLOC_013970	Ca_v2.0_13347	Ca5_v2.0	57062886	57064777
XLOC_013973	Ca_v2.0_13352	Ca5_v2.0	57123908	57126912
XLOC_013974	Ca_v2.0_13353	Ca5_v2.0	57137834	57139846
XLOC_013979	Ca_v2.0_13361,Ca_v2.0_133(	Ca5_v2.0	57209761	57215069
XLOC_013984	Ca_v2.0_13370	Ca5_v2.0	57325506	57326419
XLOC_013992	Ca_v2.0_13385	Ca5_v2.0	57490680	57493694
XLOC_013995	Ca_v2.0_13392	Ca5_v2.0	57552414	57557270
XLOC_013996	Ca_v2.0_13393	Ca5_v2.0	57559182	57561772
XLOC_013997	Ca_v2.0_13397	Ca5_v2.0	57582039	57584151
XLOC_014002	Ca_v2.0_13424	Ca5_v2.0	58030245	58031353
XLOC_014005	Ca_v2.0_13432,Ca_v2.0_134:	Ca5_v2.0	58102118	58113302
XLOC_014011	Ca_v2.0_13448	Ca5_v2.0	58359867	58371950
XLOC_014013	Ca_v2.0_13450	Ca5_v2.0	58401029	58404724
XLOC_014014	Ca_v2.0_13451	Ca5_v2.0	58408281	58412350
XLOC_014015	-	Ca5_v2.0	58487620	58489870
XLOC_014018	Ca_v2.0_13460	Ca5_v2.0	58531879	58537618
XLOC_014023	Ca_v2.0_13470	Ca5_v2.0	58628231	58631681
XLOC_014030	Ca_v2.0_13484	Ca5_v2.0	58764710	58770843
XLOC_014031	Ca_v2.0_13485	Ca5_v2.0	58786599	58791821
XLOC_014033	Ca_v2.0_13489	Ca5_v2.0	58823187	58831678
XLOC_014034	Ca_v2.0_13492	Ca5_v2.0	58851410	58855933
XLOC_014039	Ca_v2.0_13499	Ca5_v2.0	58929403	58934938
XLOC_014043	Ca_v2.0_13505	Ca5_v2.0	58987436	58990782
XLOC_014053	Ca_v2.0_13516	Ca5_v2.0	59155255	59156836
XLOC_014055	Ca_v2.0_13527	Ca5_v2.0	59262446	59262939
XLOC_014071	Ca_v2.0_13565	Ca5_v2.0	59737654	59801877
XLOC_014075	Ca_v2.0_13571	Ca5_v2.0	59881190	59882301
XLOC_014078	Ca_v2.0_13579	Ca5_v2.0	59998557	60004343
XLOC_014082	Ca_v2.0_13585	Ca5_v2.0	60057938	60060796
XLOC_014085	Ca_v2.0_13589,Ca_v2.0_135(	Ca5_v2.0	60101344	60107793
XLOC_014095	Ca_v2.0_13606	Ca5_v2.0	60342072	60345910
XLOC_014100	Ca_v2.0_13614	Ca5_v2.0	60433152	60438031
XLOC_014102	Ca_v2.0_13618	Ca5_v2.0	60482436	60483717
XLOC_014114	Ca_v2.0_13637	Ca5_v2.0	60759120	60763301
XLOC_014116	Ca_v2.0_13641	Ca5_v2.0	60821310	60824940
XLOC_014119	Ca_v2.0_13646	Ca5_v2.0	60879368	60881204
XLOC_014122	Ca_v2.0_13655	Ca5_v2.0	60986895	60989222
XLOC_014125	Ca_v2.0_13658	Ca5_v2.0	61044015	61050830
XLOC_014157	Ca_v2.0_13729	Ca5_v2.0	61792236	61794590
XLOC_014164	Ca_v2.0_13740	Ca5_v2.0	62000144	62004418
XLOC_014183	Ca_v2.0_13767	Ca5_v2.0	62446633	62450031
XLOC_014186	Ca_v2.0_13776	Ca5_v2.0	62566661	62569573
XLOC_014191	Ca_v2.0_13783	Ca5_v2.0	62635148	62641034
XLOC_014195	Ca_v2.0_13787	Ca5_v2.0	62675311	62681138

For Review Only

XLOC_014196	Ca_v2.0_13788	Ca5_v2.0	62690881	62692472
XLOC_014199	Ca_v2.0_13795	Ca5_v2.0	62764013	62769530
XLOC_014206	Ca_v2.0_13804	Ca5_v2.0	62826211	62828339
XLOC_014209	Ca_v2.0_13808	Ca5_v2.0	62864574	62871990
XLOC_014211	Ca_v2.0_13810	Ca5_v2.0	62934243	62935333
XLOC_014232	Ca_v2.0_13864	Ca5_v2.0	63476537	63482459
XLOC_014241	Ca_v2.0_13877	Ca5_v2.0	63620199	63622874
XLOC_014242	Ca_v2.0_13880	Ca5_v2.0	63647971	63657042
XLOC_014245	Ca_v2.0_13888	Ca5_v2.0	63745737	63749055
XLOC_014246	Ca_v2.0_13889	Ca5_v2.0	63761655	63765768
XLOC_014256	Ca_v2.0_13906	Ca5_v2.0	63911922	63914323
XLOC_014262	Ca_v2.0_13916	Ca5_v2.0	63999569	64016051
XLOC_014269	Ca_v2.0_13938	Ca5_v2.0	64161179	64427584
XLOC_014282	Ca_v2.0_13954	Ca5_v2.0	64485128	64517421
XLOC_014286	Ca_v2.0_13960	Ca5_v2.0	64557805	64559463
XLOC_014294	Ca_v2.0_13975	Ca5_v2.0	64736428	64739118
XLOC_014305	Ca_v2.0_13996,Ca_v2.0_139	Ca5_v2.0	64946614	64968540
XLOC_014316	Ca_v2.0_14017,Ca_v2.0_140	Ca5_v2.0	65205326	65227101
XLOC_014330	Ca_v2.0_14042	Ca5_v2.0	65416346	65417987
XLOC_014333	Ca_v2.0_14046	Ca5_v2.0	65456694	65458129
XLOC_014338	Ca_v2.0_14059,Ca_v2.0_140	Ca5_v2.0	65552275	65555362
XLOC_014347	Ca_v2.0_14077	Ca5_v2.0	65715005	65716529
XLOC_014350	Ca_v2.0_14081	Ca5_v2.0	65759698	65765864
XLOC_014360	Ca_v2.0_14092	Ca5_v2.0	65854163	65858813
XLOC_014366	Ca_v2.0_14101	Ca5_v2.0	65926982	65981442
XLOC_014378	Ca_v2.0_14116	Ca5_v2.0	66091520	66094602
XLOC_014380	Ca_v2.0_14118	Ca5_v2.0	66105959	66107233
XLOC_014382	Ca_v2.0_14124	Ca5_v2.0	66142962	66151357
XLOC_014383	Ca_v2.0_14126	Ca5_v2.0	66164584	66168136
XLOC_014384	Ca_v2.0_14127	Ca5_v2.0	66192008	66203248
XLOC_014386	Ca_v2.0_14130	Ca5_v2.0	66213788	66217312
XLOC_014398	Ca_v2.0_14149	Ca5_v2.0	66353502	66362179
XLOC_014399	Ca_v2.0_14150	Ca5_v2.0	66353502	66362179
XLOC_014404	Ca_v2.0_14162	Ca5_v2.0	66522091	66531509
XLOC_014407	Ca_v2.0_14170	Ca5_v2.0	66609997	66615253
XLOC_014408	Ca_v2.0_14171	Ca5_v2.0	66621292	66622191
XLOC_014410	Ca_v2.0_14173,Ca_v2.0_141	Ca5_v2.0	66640389	66648480
XLOC_014417	Ca_v2.0_14185	Ca5_v2.0	66759752	66762400
XLOC_014421	Ca_v2.0_14192	Ca5_v2.0	66878429	66882006
XLOC_014422	Ca_v2.0_14194	Ca5_v2.0	66904084	66919010
XLOC_014425	Ca_v2.0_14199	Ca5_v2.0	66943147	66951223
XLOC_014427	Ca_v2.0_14202	Ca5_v2.0	66971471	66978329
XLOC_014430	Ca_v2.0_14210	Ca5_v2.0	67033334	67035945
XLOC_014441	Ca_v2.0_14225	Ca5_v2.0	67196957	67201214
XLOC_014444	Ca_v2.0_14228	Ca5_v2.0	67218665	67219817
XLOC_014460	Ca_v2.0_14250	Ca5_v2.0	67411123	67417157

For Review Only

XLOC_014461	Ca_v2.0_14251	Ca5_v2.0	67417899	67426297
XLOC_014463	Ca_v2.0_14254	Ca5_v2.0	67432565	67437737
XLOC_014467	Ca_v2.0_14260	Ca5_v2.0	67507221	67510463
XLOC_014472	Ca_v2.0_14267	Ca5_v2.0	67579123	67596075
XLOC_014473	-	Ca5_v2.0	67579123	67596075
XLOC_014480	Ca_v2.0_14282	Ca5_v2.0	67682359	67684999
XLOC_014481	Ca_v2.0_14285	Ca5_v2.0	67713079	67714287
XLOC_014484	Ca_v2.0_14291	Ca5_v2.0	67770444	67772247
XLOC_014486	Ca_v2.0_14293	Ca5_v2.0	67811356	67814615
XLOC_014497	Ca_v2.0_14313	Ca5_v2.0	68039602	68040125
XLOC_014499	Ca_v2.0_14317	Ca5_v2.0	68085409	68090262
XLOC_014509	Ca_v2.0_14338	Ca5_v2.0	68258875	68262856
XLOC_014512	Ca_v2.0_14345	Ca5_v2.0	68340198	68342577
XLOC_014517	Ca_v2.0_14353	Ca5_v2.0	68420887	68425653
XLOC_014518	Ca_v2.0_14355	Ca5_v2.0	68433493	68436709
XLOC_014529	Ca_v2.0_14377	Ca5_v2.0	68679557	68680547
XLOC_014534	Ca_v2.0_14386	Ca5_v2.0	68781472	68788262
XLOC_014535	Ca_v2.0_14388	Ca5_v2.0	68795104	68799481
XLOC_014537	Ca_v2.0_14390	Ca5_v2.0	68805410	68811600
XLOC_014538	Ca_v2.0_14392	Ca5_v2.0	68822083	68826354
XLOC_014539	Ca_v2.0_14393	Ca5_v2.0	68831096	68831926
XLOC_014540	Ca_v2.0_14396	Ca5_v2.0	68832099	68897501
XLOC_014549	Ca_v2.0_14414	Ca5_v2.0	68988217	68990121
XLOC_014555	Ca_v2.0_14427	Ca5_v2.0	69113243	69115212
XLOC_014561	Ca_v2.0_14437	Ca5_v2.0	69180428	69184078
XLOC_014564	Ca_v2.0_14443	Ca5_v2.0	69238954	69241604
XLOC_014566	Ca_v2.0_14445	Ca5_v2.0	69271654	69273817
XLOC_014567	Ca_v2.0_14448	Ca5_v2.0	69282249	69287386
XLOC_014568	Ca_v2.0_14450	Ca5_v2.0	69326042	69360151
XLOC_014573	Ca_v2.0_14461	Ca5_v2.0	69415542	69418062
XLOC_014574	Ca_v2.0_14464	Ca5_v2.0	69445637	69446429
XLOC_014585	Ca_v2.0_14482	Ca5_v2.0	69577953	69579803
XLOC_014593	Ca_v2.0_14495	Ca5_v2.0	69782373	69785325
XLOC_014597	Ca_v2.0_14503	Ca5_v2.0	69837513	69839555
XLOC_014598	Ca_v2.0_14504	Ca5_v2.0	69840764	69844379
XLOC_014612	Ca_v2.0_14528	Ca5_v2.0	70103005	70108197
XLOC_014621	Ca_v2.0_14544	Ca5_v2.0	70184109	70185341
XLOC_014622	Ca_v2.0_14546	Ca5_v2.0	70229252	70234923
XLOC_014625	Ca_v2.0_14551	Ca5_v2.0	70285345	70289622
XLOC_014636	Ca_v2.0_14570	Ca5_v2.0	70481391	70483262
XLOC_014643	Ca_v2.0_14590	Ca5_v2.0	70673745	70679234
XLOC_014646	Ca_v2.0_14597	Ca5_v2.0	70709990	70710858
XLOC_014653	Ca_v2.0_14616	Ca5_v2.0	70874761	70879394
XLOC_014664	Ca_v2.0_14641	Ca5_v2.0	71166284	71169551
XLOC_014667	Ca_v2.0_14645	Ca5_v2.0	71211777	71217843
XLOC_014670	Ca_v2.0_14649	Ca5_v2.0	71236234	71241856

For Review Only

XLOC_014674	Ca_v2.0_14660	Ca5_v2.0	71316634	71317488
XLOC_014687	Ca_v2.0_14682	Ca5_v2.0	71494995	71511117
XLOC_014688	Ca_v2.0_14683	Ca5_v2.0	71512236	71514346
XLOC_014689	Ca_v2.0_14686	Ca5_v2.0	71535734	71539061
XLOC_014692	Ca_v2.0_14691	Ca5_v2.0	71578940	71579991
XLOC_014695	Ca_v2.0_14699	Ca5_v2.0	71667715	71668513
XLOC_014702	Ca_v2.0_14708	Ca5_v2.0	71811561	71813657
XLOC_014703	Ca_v2.0_14709	Ca5_v2.0	71817062	71826767
XLOC_014707	Ca_v2.0_14717	Ca5_v2.0	71952045	71959248
XLOC_014712	Ca_v2.0_14726	Ca5_v2.0	72052611	72060010
XLOC_014716	Ca_v2.0_14729	Ca5_v2.0	72092393	72094127
XLOC_014717	Ca_v2.0_14731	Ca5_v2.0	72120325	72122337
XLOC_014719	Ca_v2.0_14737	Ca5_v2.0	72155596	72158473
XLOC_014723	Ca_v2.0_14755	Ca5_v2.0	72281203	72350398
XLOC_014731	Ca_v2.0_14757	Ca5_v2.0	72281203	72350398
XLOC_014733	Ca_v2.0_14765	Ca5_v2.0	72428388	72430270
XLOC_014737	Ca_v2.0_14769,Ca_v2.0_147	Ca5_v2.0	72462747	72478064
XLOC_014745	Ca_v2.0_14782	Ca5_v2.0	72566494	72572060
XLOC_014746	Ca_v2.0_14790	Ca5_v2.0	72673131	72675793
XLOC_014750	Ca_v2.0_14795	Ca5_v2.0	72750867	72753203
XLOC_014751	Ca_v2.0_14797	Ca5_v2.0	72768193	72770910
XLOC_014757	Ca_v2.0_14810,Ca_v2.0_148	Ca5_v2.0	72887259	72892749
XLOC_014759	Ca_v2.0_14813	Ca5_v2.0	72898954	72899936
XLOC_014766	Ca_v2.0_14825	Ca5_v2.0	73004392	73009508
XLOC_014772	Ca_v2.0_14836	Ca5_v2.0	73101980	73104816
XLOC_014778	Ca_v2.0_14842	Ca5_v2.0	73140271	73142067
XLOC_014780	Ca_v2.0_14844	Ca5_v2.0	73177021	73181109
XLOC_014782	Ca_v2.0_14845	Ca5_v2.0	73204866	73209091
XLOC_014785	Ca_v2.0_14849	Ca5_v2.0	73248232	73256296
XLOC_014786	Ca_v2.0_14850	Ca5_v2.0	73270025	73272306
XLOC_014787	Ca_v2.0_14852	Ca5_v2.0	73287400	73289427
XLOC_014792	Ca_v2.0_14870	Ca5_v2.0	73438286	73442293
XLOC_014793	Ca_v2.0_14872	Ca5_v2.0	73457694	73460096
XLOC_014794	Ca_v2.0_14873	Ca5_v2.0	73466801	73471776
XLOC_014795	-	Ca5_v2.0	73500236	73504636
XLOC_014805	Ca_v2.0_14896	Ca5_v2.0	73629814	73630640
XLOC_014808	Ca_v2.0_14902	Ca5_v2.0	73653843	73666727
XLOC_014810	Ca_v2.0_14908	Ca5_v2.0	73726536	73728934
XLOC_014820	Ca_v2.0_14925	Ca5_v2.0	73918224	73918909
XLOC_014827	Ca_v2.0_14939	Ca5_v2.0	74028067	74041487
XLOC_014839	Ca_v2.0_14961	Ca5_v2.0	74305531	74355526
XLOC_014843	Ca_v2.0_14969	Ca5_v2.0	74359212	74360879
XLOC_014848	Ca_v2.0_14980	Ca5_v2.0	74460678	74463242
XLOC_014850	Ca_v2.0_14984	Ca5_v2.0	74490911	74493239
XLOC_014854	Ca_v2.0_14993	Ca5_v2.0	74575626	74579890
XLOC_014859	Ca_v2.0_15001	Ca5_v2.0	74636918	74639931

For Review Only

XLOC_014864	Ca_v2.0_15016	Ca5_v2.0	74764898	74768913
XLOC_014867	Ca_v2.0_15019	Ca5_v2.0	74783071	74793860
XLOC_014879	Ca_v2.0_15038	Ca5_v2.0	74906531	74935102
XLOC_014883	Ca_v2.0_15047	Ca5_v2.0	75003644	75011078
XLOC_014889	Ca_v2.0_15059	Ca5_v2.0	75139721	75142266
XLOC_014895	Ca_v2.0_15068	Ca5_v2.0	75235444	75237380
XLOC_014896	Ca_v2.0_15071	Ca5_v2.0	75255697	75258417
XLOC_014897	Ca_v2.0_15074	Ca5_v2.0	75272585	75280028
XLOC_014901	Ca_v2.0_15084	Ca5_v2.0	75352025	75357544
XLOC_014906	Ca_v2.0_15095	Ca5_v2.0	75439318	75446378
XLOC_014909	Ca_v2.0_15101	Ca5_v2.0	75483350	75484510
XLOC_014922	Ca_v2.0_15125	Ca5_v2.0	75724974	75728691
XLOC_014923	Ca_v2.0_15126	Ca5_v2.0	75732172	75744663
XLOC_014929	Ca_v2.0_15136	Ca5_v2.0	75842447	75853783
XLOC_014932	Ca_v2.0_15145	Ca5_v2.0	75935234	75940528
XLOC_014933	Ca_v2.0_15148	Ca5_v2.0	75966655	75969996
XLOC_014935	Ca_v2.0_15154	Ca5_v2.0	76019976	76025751
XLOC_014939	Ca_v2.0_15159	Ca5_v2.0	76073450	76083791
XLOC_014959	Ca_v2.0_15200	Ca5_v2.0	76600784	76601498
XLOC_014962	Ca_v2.0_15208	Ca5_v2.0	76665493	76666939
XLOC_014972	Ca_v2.0_15230	Ca5_v2.0	76828164	76830316
XLOC_014975	Ca_v2.0_15235	Ca5_v2.0	76842033	76844562
XLOC_014989	Ca_v2.0_15260	Ca5_v2.0	77051721	77055819
XLOC_015004	Ca_v2.0_15291	Ca5_v2.0	77370338	77372691
XLOC_015007	Ca_v2.0_15303,Ca_v2.0_153(	Ca5_v2.0	77464844	77471028
XLOC_015008	Ca_v2.0_15305	Ca5_v2.0	77472372	77475497
XLOC_015010	Ca_v2.0_15308	Ca5_v2.0	77505172	77507250
XLOC_015021	Ca_v2.0_15326	Ca5_v2.0	77657701	77658830
XLOC_015024	-	Ca5_v2.0	77735371	77738172
XLOC_015036	Ca_v2.0_15355	Ca5_v2.0	77952375	77957267
XLOC_015041	Ca_v2.0_15367	Ca5_v2.0	78082843	78084788
XLOC_015056	Ca_v2.0_15388	Ca5_v2.0	78222545	78224296
XLOC_015062	Ca_v2.0_15406	Ca5_v2.0	78271478	78364579
XLOC_015067	Ca_v2.0_15418	Ca5_v2.0	78461878	78463928
XLOC_015070	Ca_v2.0_15424	Ca5_v2.0	78497607	78498835
XLOC_015071	Ca_v2.0_15425	Ca5_v2.0	78504958	78507228
XLOC_015080	Ca_v2.0_15440	Ca5_v2.0	78605723	78608057
XLOC_015092	Ca_v2.0_15458	Ca5_v2.0	78792079	78796540
XLOC_015102	Ca_v2.0_15476	Ca5_v2.0	78939707	78941274
XLOC_015117	Ca_v2.0_15495	Ca5_v2.0	79118076	79121960
XLOC_015118	Ca_v2.0_15497	Ca5_v2.0	79130726	79136987
XLOC_015119	Ca_v2.0_15499	Ca5_v2.0	79149800	79158692
XLOC_015120	Ca_v2.0_15500	Ca5_v2.0	79167684	79179645
XLOC_015122	Ca_v2.0_15504	Ca5_v2.0	79254203	79255666
XLOC_015124	Ca_v2.0_15506	Ca5_v2.0	79260218	79265890
XLOC_015144	Ca_v2.0_12068,Ca_v2.0_120(	Ca5_v2.0	1283119	1289283

For Review Only

XLOC_015146	Ca_v2.0_12073	Ca5_v2.0	1389972	1484934
XLOC_015149	Ca_v2.0_12076	Ca5_v2.0	1389972	1484934
XLOC_015151	-	Ca5_v2.0	1695867	1699634
XLOC_015154	Ca_v2.0_12091	Ca5_v2.0	1809242	1814931
XLOC_015155	Ca_v2.0_12092	Ca5_v2.0	1853051	1855026
XLOC_015160	Ca_v2.0_12099	Ca5_v2.0	1960476	1977480
XLOC_015168	-	Ca5_v2.0	2277191	2278314
XLOC_015179	Ca_v2.0_12128	Ca5_v2.0	2448334	2453496
XLOC_015203	Ca_v2.0_12180	Ca5_v2.0	3454950	3461802
XLOC_015244	Ca_v2.0_12244	Ca5_v2.0	5205191	5220041
XLOC_015257	Ca_v2.0_12265	Ca5_v2.0	5599977	5602556
XLOC_015269	Ca_v2.0_12286	Ca5_v2.0	6148628	6149657
XLOC_015284	Ca_v2.0_12304	Ca5_v2.0	6650891	6651905
XLOC_015287	Ca_v2.0_12310	Ca5_v2.0	6949876	6954249
XLOC_015295	-	Ca5_v2.0	14172912	14174034
XLOC_015296	-	Ca5_v2.0	14188640	14189852
XLOC_015311	Ca_v2.0_12346	Ca5_v2.0	15729287	15733146
XLOC_015313	Ca_v2.0_12348,Ca_v2.0_123!	Ca5_v2.0	15737176	15744354
XLOC_015320	Ca_v2.0_12366	Ca5_v2.0	16715724	16741460
XLOC_015366	-	Ca5_v2.0	21438979	21440006
XLOC_015378	Ca_v2.0_12496	Ca5_v2.0	23064063	23085743
XLOC_015386	Ca_v2.0_12507	Ca5_v2.0	23673075	23685168
XLOC_015393	Ca_v2.0_12522	Ca5_v2.0	24320348	24330765
XLOC_015403	Ca_v2.0_12550	Ca5_v2.0	25000853	25008186
XLOC_015407	Ca_v2.0_12561	Ca5_v2.0	25166948	25192529
XLOC_015420	Ca_v2.0_12582	Ca5_v2.0	27345086	27346635
XLOC_015421	Ca_v2.0_12583	Ca5_v2.0	27456195	27460234
XLOC_015447	Ca_v2.0_12639	Ca5_v2.0	32620231	32623803
XLOC_015451	Ca_v2.0_12647	Ca5_v2.0	33273986	33278091
XLOC_015483	-	Ca5_v2.0	37773643	37774749
XLOC_015484	-	Ca5_v2.0	37802581	37808739
XLOC_015487	Ca_v2.0_12716	Ca5_v2.0	38433983	38554971
XLOC_015493	Ca_v2.0_12727	Ca5_v2.0	38930714	38930990
XLOC_015509	-	Ca5_v2.0	40538826	40540093
XLOC_015515	Ca_v2.0_12763	Ca5_v2.0	41439641	41449254
XLOC_015531	Ca_v2.0_12806	Ca5_v2.0	43116405	43178821
XLOC_015542	Ca_v2.0_12823	Ca5_v2.0	44136873	44161377
XLOC_015543	Ca_v2.0_12826	Ca5_v2.0	44212451	44214045
XLOC_015554	Ca_v2.0_12843	Ca5_v2.0	44561322	44567088
XLOC_015560	Ca_v2.0_12855	Ca5_v2.0	44872825	44877629
XLOC_015563	Ca_v2.0_12858	Ca5_v2.0	44962954	45015682
XLOC_015566	Ca_v2.0_12868	Ca5_v2.0	45329986	45331615
XLOC_015567	Ca_v2.0_12869	Ca5_v2.0	45349881	45350948
XLOC_015569	Ca_v2.0_12874	Ca5_v2.0	45576473	45614281
XLOC_015576	Ca_v2.0_12890	Ca5_v2.0	46447892	46454339
XLOC_015577	Ca_v2.0_12891	Ca5_v2.0	46460511	46464859

For Review Only

XLOC_015580	-	Ca5_v2.0	46587279	46589957
XLOC_015603	Ca_v2.0_12942	Ca5_v2.0	48316388	48322164
XLOC_015615	Ca_v2.0_12974	Ca5_v2.0	49524630	49525318
XLOC_015635	Ca_v2.0_13012	Ca5_v2.0	50471055	50474413
XLOC_015647	Ca_v2.0_13031,Ca_v2.0_130:	Ca5_v2.0	50792756	50805399
XLOC_015670	-	Ca5_v2.0	51666231	51667726
XLOC_015671	Ca_v2.0_13080	Ca5_v2.0	51874787	51880927
XLOC_015672	Ca_v2.0_13081	Ca5_v2.0	51891549	51896478
XLOC_015673	Ca_v2.0_13085	Ca5_v2.0	52016211	52018965
XLOC_015674	Ca_v2.0_13086	Ca5_v2.0	52027270	52033061
XLOC_015678	Ca_v2.0_13101	Ca5_v2.0	52269919	52367966
XLOC_015685	Ca_v2.0_13111	Ca5_v2.0	52384898	52389991
XLOC_015688	Ca_v2.0_13119	Ca5_v2.0	52493506	52499107
XLOC_015697	Ca_v2.0_13134	Ca5_v2.0	52832854	52835664
XLOC_015698	Ca_v2.0_13135	Ca5_v2.0	52857716	52861336
XLOC_015699	Ca_v2.0_13136	Ca5_v2.0	52869878	52872115
XLOC_015701	Ca_v2.0_13139	Ca5_v2.0	52950597	52959986
XLOC_015704	Ca_v2.0_13142	Ca5_v2.0	53023536	53025323
XLOC_015705	Ca_v2.0_13143	Ca5_v2.0	53088085	53090651
XLOC_015712	Ca_v2.0_13152,Ca_v2.0_131!	Ca5_v2.0	53473407	53484125
XLOC_015715	Ca_v2.0_13163	Ca5_v2.0	53668715	53673704
XLOC_015717	Ca_v2.0_13168	Ca5_v2.0	53754207	53766743
XLOC_015730	-	Ca5_v2.0	54278031	54279173
XLOC_015732	Ca_v2.0_13196	Ca5_v2.0	54339023	54341603
XLOC_015734	Ca_v2.0_13197	Ca5_v2.0	54413780	54415241
XLOC_015740	Ca_v2.0_13203	Ca5_v2.0	54530456	54533085
XLOC_015744	Ca_v2.0_13214	Ca5_v2.0	54553251	54617127
XLOC_015748	Ca_v2.0_13223	Ca5_v2.0	54789429	54795042
XLOC_015750	Ca_v2.0_13227	Ca5_v2.0	54863201	54864096
XLOC_015751	Ca_v2.0_13228	Ca5_v2.0	54900497	54901334
XLOC_015758	Ca_v2.0_13244	Ca5_v2.0	55317248	55318345
XLOC_015766	Ca_v2.0_13260	Ca5_v2.0	55518393	55522246
XLOC_015768	Ca_v2.0_13262,Ca_v2.0_132(	Ca5_v2.0	55526219	55545413
XLOC_015772	Ca_v2.0_13268	Ca5_v2.0	55579621	55583978
XLOC_015781	Ca_v2.0_13293	Ca5_v2.0	56038078	56040574
XLOC_015790	Ca_v2.0_13311	Ca5_v2.0	56393632	56397406
XLOC_015801	Ca_v2.0_13340	Ca5_v2.0	56938107	56947788
XLOC_015802	Ca_v2.0_13341	Ca5_v2.0	56951710	56956485
XLOC_015808	Ca_v2.0_13356	Ca5_v2.0	57163744	57191750
XLOC_015809	Ca_v2.0_13360	Ca5_v2.0	57208699	57209474
XLOC_015816	Ca_v2.0_13374	Ca5_v2.0	57342671	57346255
XLOC_015834	Ca_v2.0_13406	Ca5_v2.0	57718522	57721144
XLOC_015848	Ca_v2.0_13418	Ca5_v2.0	57914895	57915133
XLOC_015849	Ca_v2.0_13419	Ca5_v2.0	57915332	57915950
XLOC_015856	Ca_v2.0_13431	Ca5_v2.0	58084784	58099303
XLOC_015864	Ca_v2.0_13445	Ca5_v2.0	58249790	58252257

For Review Only

XLOC_015876	Ca_v2.0_13462	Ca5_v2.0	58549438	58565534
XLOC_015878	Ca_v2.0_13464	Ca5_v2.0	58584476	58586323
XLOC_015886	-	Ca5_v2.0	58704935	58707383
XLOC_015890	Ca_v2.0_13487	Ca5_v2.0	58800335	58809541
XLOC_015893	Ca_v2.0_13491	Ca5_v2.0	58842132	58845596
XLOC_015897	Ca_v2.0_13504	Ca5_v2.0	58961968	58964666
XLOC_015898	Ca_v2.0_13505	Ca5_v2.0	58987436	58990782
XLOC_015899	Ca_v2.0_13507	Ca5_v2.0	59034144	59037937
XLOC_015902	Ca_v2.0_13517	Ca5_v2.0	59169109	59170024
XLOC_015903	Ca_v2.0_13518	Ca5_v2.0	59172337	59184035
XLOC_015906	Ca_v2.0_13522	Ca5_v2.0	59200004	59204064
XLOC_015907	Ca_v2.0_13523	Ca5_v2.0	59219827	59220635
XLOC_015913	Ca_v2.0_13530	Ca5_v2.0	59283450	59283714
XLOC_015914	Ca_v2.0_13533	Ca5_v2.0	59302442	59337638
XLOC_015915	Ca_v2.0_13534	Ca5_v2.0	59353563	59354836
XLOC_015917	Ca_v2.0_13538	Ca5_v2.0	59423753	59428323
XLOC_015921	Ca_v2.0_13547	Ca5_v2.0	59527655	59529149
XLOC_015925	Ca_v2.0_13552	Ca5_v2.0	59598754	59604220
XLOC_015926	Ca_v2.0_13553	Ca5_v2.0	59609636	59610502
XLOC_015931	Ca_v2.0_13559	Ca5_v2.0	59672403	59675490
XLOC_015936	Ca_v2.0_13567	Ca5_v2.0	59819352	59821525
XLOC_015937	Ca_v2.0_13568	Ca5_v2.0	59832800	59836338
XLOC_015941	Ca_v2.0_13574	Ca5_v2.0	59931778	59933977
XLOC_015943	Ca_v2.0_13576	Ca5_v2.0	59959724	59961496
XLOC_015947	Ca_v2.0_13585	Ca5_v2.0	60057938	60060796
XLOC_015949	Ca_v2.0_13590	Ca5_v2.0	60101344	60107793
XLOC_015958	Ca_v2.0_13606	Ca5_v2.0	60342072	60345910
XLOC_015959	Ca_v2.0_13610	Ca5_v2.0	60379091	60381399
XLOC_015965	Ca_v2.0_13621	Ca5_v2.0	60521733	60527805
XLOC_015968	-	Ca5_v2.0	60592333	60594362
XLOC_015969	Ca_v2.0_13625	Ca5_v2.0	60594887	60596329
XLOC_015974	Ca_v2.0_13633	Ca5_v2.0	60689901	60693180
XLOC_015976	Ca_v2.0_13637	Ca5_v2.0	60759120	60763301
XLOC_015978	Ca_v2.0_13641	Ca5_v2.0	60821310	60824940
XLOC_015980	Ca_v2.0_13645	Ca5_v2.0	60859288	60860536
XLOC_015988	Ca_v2.0_13663	Ca5_v2.0	61094965	61095945
XLOC_015992	Ca_v2.0_13668	Ca5_v2.0	61123553	61128509
XLOC_016005	Ca_v2.0_13695,Ca_v2.0_136!	Ca5_v2.0	61489773	61495767
XLOC_016006	Ca_v2.0_13698	Ca5_v2.0	61508191	61510693
XLOC_016007	Ca_v2.0_13699	Ca5_v2.0	61532490	61534620
XLOC_016008	Ca_v2.0_13700	Ca5_v2.0	61537569	61539908
XLOC_016013	Ca_v2.0_13705	Ca5_v2.0	61580018	61584270
XLOC_016016	Ca_v2.0_13709	Ca5_v2.0	61607496	61612144
XLOC_016022	-	Ca5_v2.0	61718441	61722260
XLOC_016025	Ca_v2.0_13724	Ca5_v2.0	61752938	61753720
XLOC_016029	Ca_v2.0_13729	Ca5_v2.0	61792236	61794590

For Review Only

XLOC_016032	Ca_v2.0_13738	Ca5_v2.0	61983248	61989465
XLOC_016033	Ca_v2.0_13739	Ca5_v2.0	61992956	61996481
XLOC_016039	Ca_v2.0_13753	Ca5_v2.0	62171468	62176952
XLOC_016055	Ca_v2.0_13796	Ca5_v2.0	62772975	62781232
XLOC_016059	-	Ca5_v2.0	62854439	62856737
XLOC_016080	Ca_v2.0_13844	Ca5_v2.0	63293440	63299034
XLOC_016081	Ca_v2.0_13845	Ca5_v2.0	63303256	63310593
XLOC_016089	Ca_v2.0_13855	Ca5_v2.0	63404453	63407029
XLOC_016100	Ca_v2.0_13879	Ca5_v2.0	63636168	63638263
XLOC_016107	Ca_v2.0_13890	Ca5_v2.0	63766698	63776076
XLOC_016109	Ca_v2.0_13892	Ca5_v2.0	63794405	63796194
XLOC_016113	Ca_v2.0_13901	Ca5_v2.0	63859592	63865915
XLOC_016117	Ca_v2.0_13914	Ca5_v2.0	63969616	63971366
XLOC_016119	-	Ca5_v2.0	63999569	64016051
XLOC_016122	Ca_v2.0_13921	Ca5_v2.0	64060147	64063710
XLOC_016131	Ca_v2.0_13933	Ca5_v2.0	64161179	64427584
XLOC_016138	Ca_v2.0_13945	Ca5_v2.0	64161179	64427584
XLOC_016141	Ca_v2.0_13950	Ca5_v2.0	64466042	64468283
XLOC_016148	-	Ca5_v2.0	64607161	64608903
XLOC_016153	Ca_v2.0_13972	Ca5_v2.0	64698270	64701180
XLOC_016157	Ca_v2.0_13979	Ca5_v2.0	64777130	64779705
XLOC_016159	Ca_v2.0_13982	Ca5_v2.0	64793252	64799461
XLOC_016160	Ca_v2.0_13986	Ca5_v2.0	64824277	64832898
XLOC_016173	Ca_v2.0_14010	Ca5_v2.0	65144391	65148783
XLOC_016175	Ca_v2.0_14012	Ca5_v2.0	65167976	65169459
XLOC_016180	Ca_v2.0_14028	Ca5_v2.0	65288613	65290664
XLOC_016181	Ca_v2.0_14030	Ca5_v2.0	65292582	65297149
XLOC_016187	Ca_v2.0_14041	Ca5_v2.0	65401063	65404693
XLOC_016189	Ca_v2.0_14047	Ca5_v2.0	65463370	65466115
XLOC_016203	Ca_v2.0_14075	Ca5_v2.0	65689499	65692614
XLOC_016207	Ca_v2.0_14094	Ca5_v2.0	65868662	65869859
XLOC_016218	Ca_v2.0_14121	Ca5_v2.0	66119555	66121003
XLOC_016221	Ca_v2.0_14126	Ca5_v2.0	66164584	66168136
XLOC_016226	Ca_v2.0_14142	Ca5_v2.0	66307585	66317610
XLOC_016229	Ca_v2.0_14147	Ca5_v2.0	66342668	66352971
XLOC_016230	Ca_v2.0_14148,Ca_v2.0_141!	Ca5_v2.0	66353502	66362179
XLOC_016231	Ca_v2.0_14149	Ca5_v2.0	66353502	66362179
XLOC_016232	Ca_v2.0_14151	Ca5_v2.0	66365025	66379901
XLOC_016237	Ca_v2.0_14158	Ca5_v2.0	66479320	66480326
XLOC_016238	Ca_v2.0_14160	Ca5_v2.0	66506769	66507756
XLOC_016240	Ca_v2.0_14164	Ca5_v2.0	66538994	66540288
XLOC_016243	Ca_v2.0_14167	Ca5_v2.0	66587199	66591601
XLOC_016247	Ca_v2.0_14175	Ca5_v2.0	66640389	66648480
XLOC_016249	Ca_v2.0_14179	Ca5_v2.0	66687551	66689459
XLOC_016253	Ca_v2.0_14189	Ca5_v2.0	66811172	66827697
XLOC_016254	Ca_v2.0_14193	Ca5_v2.0	66888162	66890362

For Review Only

XLOC_016255	Ca_v2.0_14195	Ca5_v2.0	66904084	66919010
XLOC_016259	Ca_v2.0_14203	Ca5_v2.0	66971471	66978329
XLOC_016261	Ca_v2.0_14205	Ca5_v2.0	66998676	66999711
XLOC_016272	Ca_v2.0_14237	Ca5_v2.0	67304387	67311875
XLOC_016274	Ca_v2.0_14241	Ca5_v2.0	67356775	67359939
XLOC_016275	Ca_v2.0_14245	Ca5_v2.0	67376322	67378029
XLOC_016276	Ca_v2.0_14246	Ca5_v2.0	67385975	67387636
XLOC_016278	Ca_v2.0_14252	Ca5_v2.0	67417899	67426297
XLOC_016280	Ca_v2.0_14258	Ca5_v2.0	67482440	67484133
XLOC_016291	Ca_v2.0_14279	Ca5_v2.0	67665077	67670388
XLOC_016297	Ca_v2.0_14289	Ca5_v2.0	67738027	67742162
XLOC_016299	Ca_v2.0_14294	Ca5_v2.0	67815276	67817206
XLOC_016304	Ca_v2.0_14304	Ca5_v2.0	67948150	67954005
XLOC_016312	Ca_v2.0_14320	Ca5_v2.0	68115047	68124066
XLOC_016318	Ca_v2.0_14334	Ca5_v2.0	68228284	68231748
XLOC_016321	Ca_v2.0_14339	Ca5_v2.0	68264815	68265806
XLOC_016325	Ca_v2.0_14346	Ca5_v2.0	68346769	68350828
XLOC_016327	Ca_v2.0_14352	Ca5_v2.0	68407259	68412379
XLOC_016329	Ca_v2.0_14355	Ca5_v2.0	68433493	68436709
XLOC_016336	Ca_v2.0_14366	Ca5_v2.0	68513601	68515318
XLOC_016337	Ca_v2.0_14368	Ca5_v2.0	68555765	68562360
XLOC_016342	Ca_v2.0_14378	Ca5_v2.0	68687280	68687973
XLOC_016343	-	Ca5_v2.0	68700332	68705202
XLOC_016348	Ca_v2.0_14387	Ca5_v2.0	68781472	68788262
XLOC_016350	Ca_v2.0_14392	Ca5_v2.0	68822083	68826354
XLOC_016352	Ca_v2.0_14394	Ca5_v2.0	68832099	68897501
XLOC_016353	Ca_v2.0_14395	Ca5_v2.0	68832099	68897501
XLOC_016355	Ca_v2.0_14402	Ca5_v2.0	68832099	68897501
XLOC_016370	Ca_v2.0_14431	Ca5_v2.0	69129778	69133747
XLOC_016373	-	Ca5_v2.0	69163070	69165156
XLOC_016380	Ca_v2.0_14449	Ca5_v2.0	69320701	69321135
XLOC_016384	Ca_v2.0_14456	Ca5_v2.0	69361416	69365880
XLOC_016392	Ca_v2.0_14470	Ca5_v2.0	69492704	69495739
XLOC_016393	Ca_v2.0_14476	Ca5_v2.0	69518278	69529562
XLOC_016394	Ca_v2.0_14479	Ca5_v2.0	69552964	69555301
XLOC_016397	Ca_v2.0_14483	Ca5_v2.0	69603019	69606595
XLOC_016401	Ca_v2.0_14491	Ca5_v2.0	69719957	69728467
XLOC_016403	Ca_v2.0_14498	Ca5_v2.0	69796776	69802850
XLOC_016405	-	Ca5_v2.0	69820761	69821896
XLOC_016406	Ca_v2.0_14501	Ca5_v2.0	69828434	69829429
XLOC_016409	Ca_v2.0_14512	Ca5_v2.0	69953742	69960250
XLOC_016410	Ca_v2.0_14513	Ca5_v2.0	69953742	69960250
XLOC_016411	Ca_v2.0_14516	Ca5_v2.0	69977847	69978863
XLOC_016422	Ca_v2.0_14536	Ca5_v2.0	70136935	70140236
XLOC_016423	Ca_v2.0_14538	Ca5_v2.0	70154389	70155586
XLOC_016437	Ca_v2.0_14567	Ca5_v2.0	70441849	70449745

For Review Only

XLOC_016438	Ca_v2.0_14568	Ca5_v2.0	70451309	70459614
XLOC_016471	Ca_v2.0_14621	Ca5_v2.0	70923671	70925231
XLOC_016472	Ca_v2.0_14624	Ca5_v2.0	70950432	70952078
XLOC_016485	Ca_v2.0_14645,Ca_v2.0_146	Ca5_v2.0	71211777	71217843
XLOC_016489	Ca_v2.0_14652	Ca5_v2.0	71253763	71254872
XLOC_016498	Ca_v2.0_14675	Ca5_v2.0	71430607	71435917
XLOC_016499	Ca_v2.0_14676,Ca_v2.0_146	Ca5_v2.0	71439576	71445231
XLOC_016501	Ca_v2.0_14683	Ca5_v2.0	71512236	71514346
XLOC_016505	Ca_v2.0_14689	Ca5_v2.0	71556165	71556950
XLOC_016508	Ca_v2.0_14694	Ca5_v2.0	71609483	71611266
XLOC_016520	Ca_v2.0_14714	Ca5_v2.0	71892356	71896568
XLOC_016531	Ca_v2.0_14739	Ca5_v2.0	72170276	72172419
XLOC_016539	Ca_v2.0_14751	Ca5_v2.0	72281203	72350398
XLOC_016540	Ca_v2.0_14758	Ca5_v2.0	72281203	72350398
XLOC_016541	Ca_v2.0_14759	Ca5_v2.0	72372661	72375173
XLOC_016544	Ca_v2.0_14762	Ca5_v2.0	72400209	72406732
XLOC_016550	Ca_v2.0_14779	Ca5_v2.0	72538047	72541914
XLOC_016553	Ca_v2.0_14784	Ca5_v2.0	72606542	72608196
XLOC_016554	-	Ca5_v2.0	72631719	72634878
XLOC_016555	Ca_v2.0_14785	Ca5_v2.0	72640484	72642180
XLOC_016559	Ca_v2.0_14789	Ca5_v2.0	72665481	72666549
XLOC_016561	Ca_v2.0_14796	Ca5_v2.0	72761656	72765321
XLOC_016575	Ca_v2.0_14816	Ca5_v2.0	72906515	72912305
XLOC_016577	Ca_v2.0_14818	Ca5_v2.0	72935949	72937073
XLOC_016580	Ca_v2.0_14828	Ca5_v2.0	73027956	73029479
XLOC_016581	Ca_v2.0_14829	Ca5_v2.0	73030919	73031868
XLOC_016588	Ca_v2.0_14852	Ca5_v2.0	73287400	73289427
XLOC_016599	Ca_v2.0_14865	Ca5_v2.0	73392777	73395581
XLOC_016603	Ca_v2.0_14871	Ca5_v2.0	73449312	73450464
XLOC_016612	Ca_v2.0_14885	Ca5_v2.0	73565461	73569851
XLOC_016613	Ca_v2.0_14887	Ca5_v2.0	73577526	73578511
XLOC_016615	Ca_v2.0_14889	Ca5_v2.0	73585698	73587995
XLOC_016624	Ca_v2.0_14907	Ca5_v2.0	73715394	73718470
XLOC_016626	Ca_v2.0_14910	Ca5_v2.0	73756676	73758759
XLOC_016629	Ca_v2.0_14916	Ca5_v2.0	73822352	73823495
XLOC_016639	Ca_v2.0_14937	Ca5_v2.0	74014923	74020583
XLOC_016644	Ca_v2.0_14943	Ca5_v2.0	74061652	74064592
XLOC_016647	Ca_v2.0_14947	Ca5_v2.0	74123319	74128151
XLOC_016650	Ca_v2.0_14951	Ca5_v2.0	74147344	74155943
XLOC_016653	Ca_v2.0_14957	Ca5_v2.0	74240332	74241373
XLOC_016657	Ca_v2.0_14968	Ca5_v2.0	74305531	74355526
XLOC_016676	Ca_v2.0_15004,Ca_v2.0_150	Ca5_v2.0	74658707	74660130
XLOC_016683	Ca_v2.0_15014	Ca5_v2.0	74744816	74748928
XLOC_016685	Ca_v2.0_15020	Ca5_v2.0	74783071	74793860
XLOC_016696	Ca_v2.0_15042	Ca5_v2.0	74963398	74965363
XLOC_016698	Ca_v2.0_15048	Ca5_v2.0	75003644	75011078

For Review Only

XLOC_016710	Ca_v2.0_15072	Ca5_v2.0	75267454	75269283
XLOC_016728	Ca_v2.0_15102	Ca5_v2.0	75486128	75487217
XLOC_016735	Ca_v2.0_15118	Ca5_v2.0	75652128	75656451
XLOC_016737	Ca_v2.0_15121	Ca5_v2.0	75691783	75692966
XLOC_016739	Ca_v2.0_15124	Ca5_v2.0	75713460	75720029
XLOC_016743	Ca_v2.0_15130	Ca5_v2.0	75756694	75761094
XLOC_016754	Ca_v2.0_15149	Ca5_v2.0	75980058	75980784
XLOC_016757	Ca_v2.0_15153	Ca5_v2.0	76007089	76013957
XLOC_016758	Ca_v2.0_15154	Ca5_v2.0	76019976	76025751
XLOC_016761	Ca_v2.0_15162	Ca5_v2.0	76123295	76126186
XLOC_016766	Ca_v2.0_15174	Ca5_v2.0	76274704	76280538
XLOC_016769	Ca_v2.0_15179	Ca5_v2.0	76321653	76325445
XLOC_016773	Ca_v2.0_15188	Ca5_v2.0	76469378	76470270
XLOC_016774	Ca_v2.0_15189	Ca5_v2.0	76479638	76481619
XLOC_016786	Ca_v2.0_15207	Ca5_v2.0	76653306	76655099
XLOC_016789	Ca_v2.0_15212	Ca5_v2.0	76695939	76696352
XLOC_016800	Ca_v2.0_15232	Ca5_v2.0	76832812	76833762
XLOC_016802	Ca_v2.0_15236	Ca5_v2.0	76851667	76854083
XLOC_016803	Ca_v2.0_15237	Ca5_v2.0	76855825	76856981
XLOC_016809	Ca_v2.0_15249	Ca5_v2.0	76921131	76924662
XLOC_016811	Ca_v2.0_15252	Ca5_v2.0	76950969	76954805
XLOC_016817	Ca_v2.0_15258	Ca5_v2.0	77037964	77041676
XLOC_016818	Ca_v2.0_15259	Ca5_v2.0	77047298	77049155
XLOC_016841	Ca_v2.0_15304	Ca5_v2.0	77464844	77471028
XLOC_016844	Ca_v2.0_15313	Ca5_v2.0	77556396	77575869
XLOC_016849	-	Ca5_v2.0	77635413	77636305
XLOC_016850	Ca_v2.0_15322	Ca5_v2.0	77640911	77641241
XLOC_016852	Ca_v2.0_15327	Ca5_v2.0	77662259	77663924
XLOC_016853	Ca_v2.0_15330	Ca5_v2.0	77686198	77695497
XLOC_016854	Ca_v2.0_15331	Ca5_v2.0	77703067	77704674
XLOC_016858	Ca_v2.0_15336	Ca5_v2.0	77757556	77765037
XLOC_016862	Ca_v2.0_15342	Ca5_v2.0	77809058	77810567
XLOC_016870	Ca_v2.0_15357	Ca5_v2.0	77994830	77999040
XLOC_016878	Ca_v2.0_15374	Ca5_v2.0	78124990	78134017
XLOC_016893	Ca_v2.0_15405	Ca5_v2.0	78271478	78364579
XLOC_016894	Ca_v2.0_15406	Ca5_v2.0	78271478	78364579
XLOC_016896	Ca_v2.0_15410	Ca5_v2.0	78394518	78399256
XLOC_016928	Ca_v2.0_15473	Ca5_v2.0	78912557	78913835
XLOC_016930	Ca_v2.0_15477	Ca5_v2.0	78950108	78952916
XLOC_016932	Ca_v2.0_15479	Ca5_v2.0	78961853	78981676
XLOC_016934	Ca_v2.0_15486	Ca5_v2.0	79019081	79023790
XLOC_016937	Ca_v2.0_15495	Ca5_v2.0	79118076	79121960
XLOC_016938	Ca_v2.0_15496	Ca5_v2.0	79126781	79128185
XLOC_016947	-	Ca5_v2.0	289739	289969
XLOC_016973	-	Ca5_v2.0	17881634	17881770
XLOC_016974	-	Ca5_v2.0	18473050	18473775

For Review Only

XLOC_016980	-	Ca5_v2.0	21680448	21680700
XLOC_016983	-	Ca5_v2.0	23846377	23846834
XLOC_016984	-	Ca5_v2.0	29027941	29028219
XLOC_016986	-	Ca5_v2.0	29474824	29475692
XLOC_016987	-	Ca5_v2.0	29532214	29532972
XLOC_016990	-	Ca5_v2.0	29642324	29643161
XLOC_016991	-	Ca5_v2.0	32758573	32760224
XLOC_016993	-	Ca5_v2.0	32957490	32959062
XLOC_016994	-	Ca5_v2.0	32959478	32960285
XLOC_017011	-	Ca5_v2.0	40760871	40761101
XLOC_017022	-	Ca5_v2.0	45658741	45659784
XLOC_017027	-	Ca5_v2.0	47292958	47293091
XLOC_017028	-	Ca5_v2.0	47995269	47995893
XLOC_017041	-	Ca5_v2.0	51220847	51221132
XLOC_017044	-	Ca5_v2.0	52059655	52060250
XLOC_017046	-	Ca5_v2.0	52542442	52543012
XLOC_017048	-	Ca5_v2.0	53084389	53084963
XLOC_017075	-	Ca5_v2.0	62439211	62440224
XLOC_017080	Ca_v2.0_13993	Ca5_v2.0	64905988	64906977
XLOC_017084	-	Ca5_v2.0	66751252	66751549
XLOC_017096	-	Ca5_v2.0	69898595	69898955
XLOC_017103	-	Ca5_v2.0	72533384	72533737
XLOC_017105	-	Ca5_v2.0	72807778	72809228
XLOC_017107	-	Ca5_v2.0	72996449	72996752
XLOC_017122	-	Ca5_v2.0	77428937	77442063
XLOC_017123	-	Ca5_v2.0	78778656	78780243
XLOC_017128	Ca_v2.0_15517	Ca6_v2.0	68506	69823
XLOC_017129	Ca_v2.0_15518	Ca6_v2.0	88016	97703
XLOC_017132	Ca_v2.0_15524	Ca6_v2.0	176165	178487
XLOC_017138	Ca_v2.0_15531	Ca6_v2.0	255211	263844
XLOC_017145	Ca_v2.0_15543	Ca6_v2.0	424123	425832
XLOC_017148	Ca_v2.0_15548	Ca6_v2.0	465451	467225
XLOC_017150	Ca_v2.0_15553	Ca6_v2.0	552803	554966
XLOC_017152	-	Ca6_v2.0	577234	579064
XLOC_017154	Ca_v2.0_15556	Ca6_v2.0	600966	604526
XLOC_017159	Ca_v2.0_15564	Ca6_v2.0	675238	677748
XLOC_017168	Ca_v2.0_15582	Ca6_v2.0	948673	956030
XLOC_017175	Ca_v2.0_15600	Ca6_v2.0	1122105	1124568
XLOC_017177	Ca_v2.0_15605	Ca6_v2.0	1171229	1177476
XLOC_017179	Ca_v2.0_15607	Ca6_v2.0	1188332	1189163
XLOC_017181	Ca_v2.0_15609	Ca6_v2.0	1198012	1213012
XLOC_017184	Ca_v2.0_15615	Ca6_v2.0	1315712	1319242
XLOC_017202	Ca_v2.0_15648	Ca6_v2.0	1711550	1713687
XLOC_017206	Ca_v2.0_15659	Ca6_v2.0	1794501	1795220
XLOC_017212	Ca_v2.0_15673	Ca6_v2.0	1882982	1884361
XLOC_017213	Ca_v2.0_15674	Ca6_v2.0	1895904	1896800

For Review Only

XLOC_017215	Ca_v2.0_15676	Ca6_v2.0	1912524	1920715
XLOC_017232	Ca_v2.0_15704	Ca6_v2.0	2138539	2140712
XLOC_017234	Ca_v2.0_15706	Ca6_v2.0	2146316	2152507
XLOC_017235	Ca_v2.0_15707	Ca6_v2.0	2154000	2156398
XLOC_017237	Ca_v2.0_15709	Ca6_v2.0	2175823	2176615
XLOC_017254	Ca_v2.0_15734	Ca6_v2.0	2394505	2397598
XLOC_017255	Ca_v2.0_15735	Ca6_v2.0	2402052	2434545
XLOC_017256	Ca_v2.0_15735	Ca6_v2.0	2402052	2434545
XLOC_017262	Ca_v2.0_15750	Ca6_v2.0	2513851	2515688
XLOC_017268	Ca_v2.0_15756	Ca6_v2.0	2562917	2566495
XLOC_017270	Ca_v2.0_15761	Ca6_v2.0	2589620	2592525
XLOC_017271	Ca_v2.0_15762	Ca6_v2.0	2596226	2600926
XLOC_017272	Ca_v2.0_15763	Ca6_v2.0	2606010	2613661
XLOC_017274	Ca_v2.0_15767	Ca6_v2.0	2662476	2668494
XLOC_017283	Ca_v2.0_15777	Ca6_v2.0	2845567	2846577
XLOC_017292	Ca_v2.0_15796	Ca6_v2.0	3169161	3170292
XLOC_017293	Ca_v2.0_15797	Ca6_v2.0	3172495	3172919
XLOC_017315	Ca_v2.0_15841	Ca6_v2.0	3544402	3546343
XLOC_017318	Ca_v2.0_15845	Ca6_v2.0	3573513	3582342
XLOC_017324	Ca_v2.0_15864	Ca6_v2.0	3705290	3711622
XLOC_017325	Ca_v2.0_15865	Ca6_v2.0	3719036	3724153
XLOC_017326	Ca_v2.0_15867	Ca6_v2.0	3729669	3741458
XLOC_017329	Ca_v2.0_15871	Ca6_v2.0	3787676	3791735
XLOC_017338	Ca_v2.0_15892	Ca6_v2.0	3967877	3968730
XLOC_017340	Ca_v2.0_15894	Ca6_v2.0	4001353	4002589
XLOC_017348	Ca_v2.0_15912	Ca6_v2.0	4215496	4217103
XLOC_017351	Ca_v2.0_15917	Ca6_v2.0	4262479	4266302
XLOC_017356	Ca_v2.0_15925	Ca6_v2.0	4329933	4331071
XLOC_017361	Ca_v2.0_15932	Ca6_v2.0	4379243	4382057
XLOC_017368	Ca_v2.0_15944,Ca_v2.0_159	Ca6_v2.0	4464404	4467458
XLOC_017370	Ca_v2.0_15956	Ca6_v2.0	4526487	4545139
XLOC_017374	Ca_v2.0_15960	Ca6_v2.0	4567191	4578579
XLOC_017375	Ca_v2.0_15963	Ca6_v2.0	4627074	4628165
XLOC_017377	Ca_v2.0_15966	Ca6_v2.0	4639673	4640703
XLOC_017378	Ca_v2.0_15967	Ca6_v2.0	4642664	4646398
XLOC_017385	Ca_v2.0_15985	Ca6_v2.0	4782341	4783173
XLOC_017393	Ca_v2.0_15996	Ca6_v2.0	4918623	4920327
XLOC_017397	Ca_v2.0_16003	Ca6_v2.0	4988547	4991272
XLOC_017403	Ca_v2.0_16014	Ca6_v2.0	5110061	5110885
XLOC_017410	Ca_v2.0_16025	Ca6_v2.0	5252124	5253495
XLOC_017413	Ca_v2.0_16032	Ca6_v2.0	5319049	5322235
XLOC_017420	Ca_v2.0_16041	Ca6_v2.0	5413414	5416297
XLOC_017426	Ca_v2.0_16051	Ca6_v2.0	5484210	5485449
XLOC_017430	Ca_v2.0_16056	Ca6_v2.0	5527499	5531155
XLOC_017432	Ca_v2.0_16058	Ca6_v2.0	5539007	5543510
XLOC_017434	Ca_v2.0_16061	Ca6_v2.0	5569304	5570208

For Review Only

XLOC_017436	Ca_v2.0_16063	Ca6_v2.0	5586928	5590806
XLOC_017444	Ca_v2.0_16076	Ca6_v2.0	5694593	5695475
XLOC_017449	Ca_v2.0_16087	Ca6_v2.0	5842920	5844575
XLOC_017453	Ca_v2.0_16096	Ca6_v2.0	5931963	5935173
XLOC_017458	-	Ca6_v2.0	6063427	6064545
XLOC_017466	Ca_v2.0_16124	Ca6_v2.0	6194560	6195042
XLOC_017476	Ca_v2.0_16145	Ca6_v2.0	6389916	6393229
XLOC_017491	Ca_v2.0_16170	Ca6_v2.0	6652526	6657411
XLOC_017495	Ca_v2.0_16177	Ca6_v2.0	6695886	6700599
XLOC_017503	Ca_v2.0_16191	Ca6_v2.0	6832566	6834406
XLOC_017509	-	Ca6_v2.0	6995954	7002053
XLOC_017510	Ca_v2.0_16210	Ca6_v2.0	7014065	7017671
XLOC_017511	Ca_v2.0_16211	Ca6_v2.0	7023770	7025875
XLOC_017517	Ca_v2.0_16224,Ca_v2.0_162	Ca6_v2.0	7135788	7149597
XLOC_017518	Ca_v2.0_16225	Ca6_v2.0	7135788	7149597
XLOC_017522	Ca_v2.0_16232	Ca6_v2.0	7198758	7202487
XLOC_017525	Ca_v2.0_16235	Ca6_v2.0	7235947	7236556
XLOC_017527	Ca_v2.0_16238	Ca6_v2.0	7263845	7265045
XLOC_017528	Ca_v2.0_16240	Ca6_v2.0	7274333	7280465
XLOC_017531	-	Ca6_v2.0	7341964	7345501
XLOC_017551	Ca_v2.0_16282	Ca6_v2.0	7656240	7667282
XLOC_017558	Ca_v2.0_16294	Ca6_v2.0	7751974	7755609
XLOC_017561	Ca_v2.0_16298	Ca6_v2.0	7784937	7786461
XLOC_017562	Ca_v2.0_16299	Ca6_v2.0	7811730	7814446
XLOC_017563	Ca_v2.0_16301	Ca6_v2.0	7826149	7827241
XLOC_017564	Ca_v2.0_16303	Ca6_v2.0	7836071	7839356
XLOC_017567	Ca_v2.0_16306	Ca6_v2.0	7864278	7867335
XLOC_017571	Ca_v2.0_16312	Ca6_v2.0	7903972	7907430
XLOC_017573	Ca_v2.0_16314	Ca6_v2.0	7915346	7926931
XLOC_017576	Ca_v2.0_16318	Ca6_v2.0	7939395	7940265
XLOC_017577	Ca_v2.0_16320	Ca6_v2.0	7960783	7971175
XLOC_017580	-	Ca6_v2.0	8003665	8006768
XLOC_017583	Ca_v2.0_16336	Ca6_v2.0	8087038	8091581
XLOC_017586	Ca_v2.0_16346	Ca6_v2.0	8227715	8230586
XLOC_017591	Ca_v2.0_16359	Ca6_v2.0	8332330	8335834
XLOC_017601	Ca_v2.0_16375,Ca_v2.0_163	Ca6_v2.0	8462069	8476784
XLOC_017610	Ca_v2.0_16392	Ca6_v2.0	8603656	8604931
XLOC_017617	Ca_v2.0_16402	Ca6_v2.0	8698665	8700196
XLOC_017619	Ca_v2.0_16405	Ca6_v2.0	8719752	8720668
XLOC_017630	Ca_v2.0_16429	Ca6_v2.0	9018569	9019381
XLOC_017637	Ca_v2.0_16438	Ca6_v2.0	9074749	9082612
XLOC_017640	Ca_v2.0_16444	Ca6_v2.0	9131105	9182010
XLOC_017646	Ca_v2.0_16454	Ca6_v2.0	9251280	9254374
XLOC_017652	Ca_v2.0_16470	Ca6_v2.0	9369182	9370406
XLOC_017657	Ca_v2.0_16482	Ca6_v2.0	9474020	9475034
XLOC_017659	Ca_v2.0_16485,Ca_v2.0_164	Ca6_v2.0	9505724	9514099

For Review Only

XLOC_017660	-	Ca6_v2.0	9565481	9567409
XLOC_017666	Ca_v2.0_16506	Ca6_v2.0	9694241	9699036
XLOC_017667	Ca_v2.0_16509	Ca6_v2.0	9719399	9726129
XLOC_017669	Ca_v2.0_16513	Ca6_v2.0	9774237	9786134
XLOC_017677	Ca_v2.0_16523	Ca6_v2.0	9868450	9869744
XLOC_017685	Ca_v2.0_16545	Ca6_v2.0	10057387	10064807
XLOC_017700	Ca_v2.0_16586	Ca6_v2.0	10426321	10436711
XLOC_017706	Ca_v2.0_16594	Ca6_v2.0	10471485	10474047
XLOC_017707	Ca_v2.0_16595	Ca6_v2.0	10474759	10482237
XLOC_017720	Ca_v2.0_16618,Ca_v2.0_166	Ca6_v2.0	10756983	10761816
XLOC_017737	Ca_v2.0_16655	Ca6_v2.0	11162799	11171165
XLOC_017743	Ca_v2.0_16663	Ca6_v2.0	11272998	11285040
XLOC_017747	Ca_v2.0_16672	Ca6_v2.0	11409539	11411133
XLOC_017748	Ca_v2.0_16675	Ca6_v2.0	11464420	11467592
XLOC_017753	Ca_v2.0_16683	Ca6_v2.0	11554958	11559432
XLOC_017757	Ca_v2.0_16690	Ca6_v2.0	11627350	11630628
XLOC_017762	Ca_v2.0_16698	Ca6_v2.0	11693663	11699404
XLOC_017766	Ca_v2.0_16703	Ca6_v2.0	11740882	11743231
XLOC_017767	Ca_v2.0_16704,Ca_v2.0_167(	Ca6_v2.0	11758639	11762966
XLOC_017772	Ca_v2.0_16710	Ca6_v2.0	11764917	11827974
XLOC_017777	Ca_v2.0_16727	Ca6_v2.0	11997148	11998665
XLOC_017783	Ca_v2.0_16739	Ca6_v2.0	12174682	12176071
XLOC_017784	Ca_v2.0_16740	Ca6_v2.0	12176850	12187366
XLOC_017790	Ca_v2.0_16750	Ca6_v2.0	12234774	12235215
XLOC_017791	Ca_v2.0_16751	Ca6_v2.0	12246109	12248297
XLOC_017806	Ca_v2.0_16781	Ca6_v2.0	12596856	12599553
XLOC_017813	Ca_v2.0_16797	Ca6_v2.0	12719491	12724687
XLOC_017817	Ca_v2.0_16810	Ca6_v2.0	12814511	12829324
XLOC_017822	Ca_v2.0_16816	Ca6_v2.0	12876624	12882719
XLOC_017842	Ca_v2.0_16873	Ca6_v2.0	13356509	13358013
XLOC_017851	Ca_v2.0_16892	Ca6_v2.0	13514480	13523414
XLOC_017852	Ca_v2.0_16894	Ca6_v2.0	13536549	13542414
XLOC_017853	Ca_v2.0_16895	Ca6_v2.0	13543296	13552097
XLOC_017854	Ca_v2.0_16898	Ca6_v2.0	13570128	13572910
XLOC_017855	Ca_v2.0_16899	Ca6_v2.0	13588252	13590480
XLOC_017859	Ca_v2.0_16905	Ca6_v2.0	13653536	13659530
XLOC_017860	Ca_v2.0_16906	Ca6_v2.0	13665048	13667848
XLOC_017862	Ca_v2.0_16909	Ca6_v2.0	13672073	13675224
XLOC_017864	Ca_v2.0_16912	Ca6_v2.0	13694870	13696037
XLOC_017865	Ca_v2.0_16917	Ca6_v2.0	13715450	13726102
XLOC_017875	Ca_v2.0_16932	Ca6_v2.0	13835034	13838014
XLOC_017876	Ca_v2.0_16935	Ca6_v2.0	13858394	13880552
XLOC_017891	Ca_v2.0_16966	Ca6_v2.0	14063908	14068420
XLOC_017897	Ca_v2.0_16978	Ca6_v2.0	14205231	14208309
XLOC_017912	Ca_v2.0_17003	Ca6_v2.0	14546121	14551960
XLOC_017926	Ca_v2.0_17049	Ca6_v2.0	15092961	15093901

For Review Only

XLOC_017927	Ca_v2.0_17052	Ca6_v2.0	15118864	15123102
XLOC_017947	Ca_v2.0_17089	Ca6_v2.0	15559972	15562357
XLOC_017963	Ca_v2.0_17116	Ca6_v2.0	15790750	15881517
XLOC_017964	Ca_v2.0_17118	Ca6_v2.0	15790750	15881517
XLOC_017965	Ca_v2.0_17119	Ca6_v2.0	15790750	15881517
XLOC_017969	Ca_v2.0_17133	Ca6_v2.0	16046062	16047352
XLOC_017970	Ca_v2.0_17134	Ca6_v2.0	16051090	16054383
XLOC_017971	Ca_v2.0_17139	Ca6_v2.0	16118763	16121038
XLOC_017973	Ca_v2.0_17142	Ca6_v2.0	16149807	16151805
XLOC_017976	Ca_v2.0_17147	Ca6_v2.0	16186267	16189495
XLOC_017978	Ca_v2.0_17155	Ca6_v2.0	16249039	16252113
XLOC_017992	Ca_v2.0_17180	Ca6_v2.0	16460920	16463991
XLOC_017997	Ca_v2.0_17194	Ca6_v2.0	16612006	16614268
XLOC_018004	Ca_v2.0_17203	Ca6_v2.0	16675535	16694623
XLOC_018008	Ca_v2.0_17208	Ca6_v2.0	16754521	16757154
XLOC_018009	Ca_v2.0_17209	Ca6_v2.0	16761651	16762357
XLOC_018011	Ca_v2.0_17211	Ca6_v2.0	16775944	16777181
XLOC_018014	Ca_v2.0_17217	Ca6_v2.0	16832222	16837152
XLOC_018017	Ca_v2.0_17220	Ca6_v2.0	16860394	16861645
XLOC_018018	Ca_v2.0_17221	Ca6_v2.0	16870867	16872319
XLOC_018019	Ca_v2.0_17222	Ca6_v2.0	16890593	16892577
XLOC_018020	Ca_v2.0_17223	Ca6_v2.0	16900647	16905463
XLOC_018022	Ca_v2.0_17227	Ca6_v2.0	16957312	16960000
XLOC_018026	Ca_v2.0_17232	Ca6_v2.0	16987952	16991457
XLOC_018033	Ca_v2.0_17250	Ca6_v2.0	17207730	17211652
XLOC_018038	Ca_v2.0_17258	Ca6_v2.0	17309781	17310003
XLOC_018041	Ca_v2.0_17262	Ca6_v2.0	17341749	17351292
XLOC_018043	Ca_v2.0_17264,Ca_v2.0_172(	Ca6_v2.0	17371322	17376218
XLOC_018051	Ca_v2.0_17276	Ca6_v2.0	17499542	17502545
XLOC_018052	Ca_v2.0_17277	Ca6_v2.0	17509855	17510763
XLOC_018054	-	Ca6_v2.0	17522873	17523997
XLOC_018058	Ca_v2.0_17285	Ca6_v2.0	17617687	17634828
XLOC_018059	Ca_v2.0_17288	Ca6_v2.0	17652268	17660880
XLOC_018061	Ca_v2.0_17291	Ca6_v2.0	17685408	17686413
XLOC_018063	Ca_v2.0_17294	Ca6_v2.0	17706422	17710099
XLOC_018066	Ca_v2.0_17301	Ca6_v2.0	17774124	17776391
XLOC_018078	Ca_v2.0_17317	Ca6_v2.0	17915785	17921241
XLOC_018081	Ca_v2.0_17322	Ca6_v2.0	17972967	17977792
XLOC_018082	Ca_v2.0_17325	Ca6_v2.0	18002888	18018685
XLOC_018084	Ca_v2.0_17328	Ca6_v2.0	18060027	18061410
XLOC_018103	Ca_v2.0_17367	Ca6_v2.0	18451552	18453405
XLOC_018104	Ca_v2.0_17370	Ca6_v2.0	18504607	18507897
XLOC_018108	Ca_v2.0_17378,Ca_v2.0_173	Ca6_v2.0	18583723	18590824
XLOC_018111	Ca_v2.0_17387	Ca6_v2.0	18721424	18723404
XLOC_018113	Ca_v2.0_17389	Ca6_v2.0	18765024	18766772
XLOC_018115	Ca_v2.0_17395	Ca6_v2.0	18838960	18841308

For Review Only

XLOC_018116	Ca_v2.0_17396,Ca_v2.0_173!	Ca6_v2.0	18842116	18851106
XLOC_018120	Ca_v2.0_17403	Ca6_v2.0	18898512	18900885
XLOC_018121	Ca_v2.0_17408	Ca6_v2.0	18923378	18929387
XLOC_018123	Ca_v2.0_17412	Ca6_v2.0	18966130	18968774
XLOC_018135	Ca_v2.0_17435	Ca6_v2.0	19242096	19243873
XLOC_018138	Ca_v2.0_17439	Ca6_v2.0	19262788	19264391
XLOC_018141	Ca_v2.0_17443	Ca6_v2.0	19280704	19285762
XLOC_018142	Ca_v2.0_17446	Ca6_v2.0	19312174	19318429
XLOC_018145	Ca_v2.0_17450	Ca6_v2.0	19391140	19392928
XLOC_018148	Ca_v2.0_17454	Ca6_v2.0	19432162	19451294
XLOC_018149	Ca_v2.0_17457	Ca6_v2.0	19466288	19467105
XLOC_018151	Ca_v2.0_17460	Ca6_v2.0	19528505	19534858
XLOC_018153	Ca_v2.0_17465,Ca_v2.0_174(	Ca6_v2.0	19568034	19572073
XLOC_018159	Ca_v2.0_17476	Ca6_v2.0	19633069	19636876
XLOC_018163	Ca_v2.0_17485	Ca6_v2.0	19718592	19722644
XLOC_018167	Ca_v2.0_17492	Ca6_v2.0	19795472	19797701
XLOC_018173	Ca_v2.0_17501	Ca6_v2.0	19882077	19888079
XLOC_018174	Ca_v2.0_17502	Ca6_v2.0	19899138	19901901
XLOC_018200	Ca_v2.0_17541	Ca6_v2.0	20290219	20292724
XLOC_018207	Ca_v2.0_17553,Ca_v2.0_175!	Ca6_v2.0	20379933	20392030
XLOC_018210	Ca_v2.0_17558	Ca6_v2.0	20427033	20428993
XLOC_018212	Ca_v2.0_17560	Ca6_v2.0	20440090	20445757
XLOC_018224	Ca_v2.0_17585	Ca6_v2.0	20757235	20761438
XLOC_018230	Ca_v2.0_17601	Ca6_v2.0	21309485	21357400
XLOC_018232	Ca_v2.0_17608	Ca6_v2.0	21383617	21391657
XLOC_018241	Ca_v2.0_17625	Ca6_v2.0	21613457	21618252
XLOC_018249	Ca_v2.0_17637	Ca6_v2.0	21684940	21688337
XLOC_018250	Ca_v2.0_17638	Ca6_v2.0	21705388	21707615
XLOC_018256	Ca_v2.0_17646	Ca6_v2.0	21776403	21787792
XLOC_018277	Ca_v2.0_17690	Ca6_v2.0	22272094	22274390
XLOC_018281	Ca_v2.0_17693,Ca_v2.0_176!	Ca6_v2.0	22302113	22312890
XLOC_018283	Ca_v2.0_17697	Ca6_v2.0	22338113	22341056
XLOC_018285	Ca_v2.0_17699	Ca6_v2.0	22381456	22382170
XLOC_018289	Ca_v2.0_17703	Ca6_v2.0	22410855	22413981
XLOC_018299	Ca_v2.0_17718	Ca6_v2.0	22643218	22646242
XLOC_018300	Ca_v2.0_17719	Ca6_v2.0	22654733	22656651
XLOC_018305	Ca_v2.0_17728	Ca6_v2.0	22789657	22794309
XLOC_018323	Ca_v2.0_17767	Ca6_v2.0	23392782	23405707
XLOC_018326	Ca_v2.0_17774	Ca6_v2.0	23523427	23525381
XLOC_018351	Ca_v2.0_17823	Ca6_v2.0	24488893	24492180
XLOC_018354	Ca_v2.0_17827	Ca6_v2.0	24597261	24601604
XLOC_018356	Ca_v2.0_17831	Ca6_v2.0	24735649	24737580
XLOC_018361	Ca_v2.0_17839	Ca6_v2.0	24983642	24985133
XLOC_018363	Ca_v2.0_17847,Ca_v2.0_178	Ca6_v2.0	25149095	25159020
XLOC_018366	Ca_v2.0_17852	Ca6_v2.0	25306113	25308349
XLOC_018368	Ca_v2.0_17855	Ca6_v2.0	25326103	25329455

For Review Only

XLOC_018370	Ca_v2.0_17861	Ca6_v2.0	25459164	25460694
XLOC_018374	Ca_v2.0_17868	Ca6_v2.0	25634114	25636643
XLOC_018375	Ca_v2.0_17870	Ca6_v2.0	25644414	25649352
XLOC_018386	Ca_v2.0_17890	Ca6_v2.0	25917210	25919152
XLOC_018388	Ca_v2.0_17892	Ca6_v2.0	25928098	25936244
XLOC_018390	Ca_v2.0_17894	Ca6_v2.0	25958557	25968640
XLOC_018391	-	Ca6_v2.0	25996511	25998659
XLOC_018393	Ca_v2.0_17897	Ca6_v2.0	26073844	26081355
XLOC_018397	Ca_v2.0_17912	Ca6_v2.0	26428528	26431262
XLOC_018398	Ca_v2.0_17913	Ca6_v2.0	26461804	26468317
XLOC_018402	Ca_v2.0_17930	Ca6_v2.0	26750250	26752910
XLOC_018411	Ca_v2.0_17944	Ca6_v2.0	26919530	26927654
XLOC_018414	Ca_v2.0_17947	Ca6_v2.0	26966956	26971908
XLOC_018420	Ca_v2.0_17961	Ca6_v2.0	27273007	27275308
XLOC_018422	Ca_v2.0_17963	Ca6_v2.0	27311519	27313261
XLOC_018423	Ca_v2.0_17964	Ca6_v2.0	27332465	27335373
XLOC_018431	Ca_v2.0_17976	Ca6_v2.0	27656867	27660270
XLOC_018437	Ca_v2.0_17990	Ca6_v2.0	27990309	27995328
XLOC_018439	Ca_v2.0_17993	Ca6_v2.0	28012561	28014539
XLOC_018441	Ca_v2.0_17996	Ca6_v2.0	28148917	28151911
XLOC_018451	Ca_v2.0_18016	Ca6_v2.0	29024427	29030007
XLOC_018456	Ca_v2.0_18023	Ca6_v2.0	29252075	29256355
XLOC_018460	Ca_v2.0_18029	Ca6_v2.0	29388114	29392299
XLOC_018469	Ca_v2.0_18046	Ca6_v2.0	29662322	29669972
XLOC_018470	Ca_v2.0_18048,Ca_v2.0_180	Ca6_v2.0	29724840	29769049
XLOC_018471	Ca_v2.0_18050	Ca6_v2.0	29802840	29806660
XLOC_018474	-	Ca6_v2.0	29905185	29910867
XLOC_018477	Ca_v2.0_18058	Ca6_v2.0	30049130	30052980
XLOC_018495	Ca_v2.0_18097	Ca6_v2.0	31080158	31086103
XLOC_018496	Ca_v2.0_18100	Ca6_v2.0	31244896	31249556
XLOC_018506	Ca_v2.0_18123	Ca6_v2.0	32026647	32028811
XLOC_018507	Ca_v2.0_18124	Ca6_v2.0	32031186	32033486
XLOC_018513	Ca_v2.0_18135	Ca6_v2.0	32456969	32463643
XLOC_018518	-	Ca6_v2.0	32770193	32772070
XLOC_018520	Ca_v2.0_18143	Ca6_v2.0	32851224	32857090
XLOC_018523	Ca_v2.0_18148	Ca6_v2.0	33114932	33116171
XLOC_018528	Ca_v2.0_18168	Ca6_v2.0	34139268	34143619
XLOC_018542	Ca_v2.0_18201	Ca6_v2.0	35562964	35565741
XLOC_018544	Ca_v2.0_18206	Ca6_v2.0	35704090	35705633
XLOC_018558	Ca_v2.0_18227	Ca6_v2.0	36499217	36503347
XLOC_018573	Ca_v2.0_18252	Ca6_v2.0	37494047	37497610
XLOC_018576	Ca_v2.0_18256	Ca6_v2.0	37615503	37675086
XLOC_018585	-	Ca6_v2.0	39299601	39300556
XLOC_018596	Ca_v2.0_18301	Ca6_v2.0	40122952	40124428
XLOC_018609	Ca_v2.0_18326	Ca6_v2.0	41058499	41061054
XLOC_018611	-	Ca6_v2.0	41439848	41441619

For Review Only

XLOC_018612	Ca_v2.0_18332	Ca6_v2.0	41562724	41564441
XLOC_018614	Ca_v2.0_18343	Ca6_v2.0	42145232	42147739
XLOC_018615	Ca_v2.0_18345	Ca6_v2.0	42384982	42392056
XLOC_018616	Ca_v2.0_18346	Ca6_v2.0	42395440	42406255
XLOC_018624	Ca_v2.0_18358	Ca6_v2.0	43980309	43997559
XLOC_018642	Ca_v2.0_18391	Ca6_v2.0	45537066	45538904
XLOC_018654	Ca_v2.0_18415	Ca6_v2.0	46512356	46513656
XLOC_018664	Ca_v2.0_18425	Ca6_v2.0	47115452	47117345
XLOC_018665	Ca_v2.0_18426	Ca6_v2.0	47156781	47159261
XLOC_018666	Ca_v2.0_18427	Ca6_v2.0	47169225	47172358
XLOC_018671	Ca_v2.0_18434	Ca6_v2.0	47581363	47582978
XLOC_018675	Ca_v2.0_18440	Ca6_v2.0	48068001	48068927
XLOC_018677	Ca_v2.0_18444	Ca6_v2.0	48199621	48205517
XLOC_018678	Ca_v2.0_18445	Ca6_v2.0	48226730	48228878
XLOC_018699	Ca_v2.0_18469	Ca6_v2.0	49144847	49146017
XLOC_018703	Ca_v2.0_18473	Ca6_v2.0	49962190	49963620
XLOC_018714	Ca_v2.0_18494	Ca6_v2.0	50680069	50683468
XLOC_018716	-	Ca6_v2.0	51017412	51017876
XLOC_018717	Ca_v2.0_18500	Ca6_v2.0	51018149	51018575
XLOC_018726	Ca_v2.0_18514	Ca6_v2.0	51433737	51437995
XLOC_018730	Ca_v2.0_18524	Ca6_v2.0	51720564	51722943
XLOC_018733	Ca_v2.0_18528	Ca6_v2.0	51849792	51858335
XLOC_018737	Ca_v2.0_18535	Ca6_v2.0	52129419	52139624
XLOC_018742	Ca_v2.0_18543	Ca6_v2.0	52504220	52548041
XLOC_018755	Ca_v2.0_18566	Ca6_v2.0	53293361	53299190
XLOC_018758	Ca_v2.0_18573	Ca6_v2.0	53493817	53501052
XLOC_018765	Ca_v2.0_18587	Ca6_v2.0	54704944	54705764
XLOC_018772	-	Ca6_v2.0	54982043	54984157
XLOC_018774	-	Ca6_v2.0	55100072	55100753
XLOC_018775	-	Ca6_v2.0	55108147	55108862
XLOC_018776	-	Ca6_v2.0	55139929	55141417
XLOC_018796	Ca_v2.0_18630	Ca6_v2.0	56613445	56614567
XLOC_018797	Ca_v2.0_18631	Ca6_v2.0	56621463	56622676
XLOC_018799	Ca_v2.0_18639	Ca6_v2.0	56917968	56920968
XLOC_018805	Ca_v2.0_18651	Ca6_v2.0	57474512	57477356
XLOC_018828	Ca_v2.0_18704	Ca6_v2.0	58871908	58874295
XLOC_018831	Ca_v2.0_18713	Ca6_v2.0	59173457	59174966
XLOC_018832	Ca_v2.0_18719	Ca6_v2.0	59295756	59296618
XLOC_018834	Ca_v2.0_18724	Ca6_v2.0	59341065	59348255
XLOC_018835	Ca_v2.0_18725	Ca6_v2.0	59341065	59348255
XLOC_018839	Ca_v2.0_18732	Ca6_v2.0	59611880	59615448
XLOC_018841	Ca_v2.0_18737	Ca6_v2.0	59668520	59687714
XLOC_018849	Ca_v2.0_18748	Ca6_v2.0	60009129	60011261
XLOC_018878	Ca_v2.0_18799	Ca6_v2.0	61548383	61549563
XLOC_018881	Ca_v2.0_18807	Ca6_v2.0	61711955	61723256
XLOC_018886	-	Ca6_v2.0	62058420	62059308

For Review Only

XLOC_018887	Ca_v2.0_18818	Ca6_v2.0	62093807	62100565
XLOC_018893	Ca_v2.0_18826	Ca6_v2.0	62222849	62223706
XLOC_018896	Ca_v2.0_18831	Ca6_v2.0	62290653	62291839
XLOC_018907	Ca_v2.0_18855	Ca6_v2.0	62767610	62768546
XLOC_018914	Ca_v2.0_18872	Ca6_v2.0	62972557	62977323
XLOC_018916	Ca_v2.0_18876	Ca6_v2.0	63043750	63047492
XLOC_018926	Ca_v2.0_18892	Ca6_v2.0	63314248	63315950
XLOC_018931	Ca_v2.0_18898	Ca6_v2.0	63384630	63385603
XLOC_018938	Ca_v2.0_18908	Ca6_v2.0	63631552	63638047
XLOC_018941	Ca_v2.0_18914	Ca6_v2.0	63894305	63901430
XLOC_018950	Ca_v2.0_18931	Ca6_v2.0	64259549	64265611
XLOC_018953	Ca_v2.0_18937	Ca6_v2.0	64345643	64349321
XLOC_018955	Ca_v2.0_18940	Ca6_v2.0	64372080	64375634
XLOC_018957	Ca_v2.0_18944	Ca6_v2.0	64447877	64450794
XLOC_018960	Ca_v2.0_18948	Ca6_v2.0	64536831	64540927
XLOC_018961	-	Ca6_v2.0	64550776	64553150
XLOC_018965	Ca_v2.0_18953	Ca6_v2.0	64627992	64630002
XLOC_018971	-	Ca6_v2.0	64917279	64919717
XLOC_018972	Ca_v2.0_18965	Ca6_v2.0	64933437	64936528
XLOC_018974	Ca_v2.0_18967	Ca6_v2.0	64971007	64975761
XLOC_018983	Ca_v2.0_18978	Ca6_v2.0	65119633	65121919
XLOC_018990	Ca_v2.0_18988	Ca6_v2.0	65501000	65503078
XLOC_018996	Ca_v2.0_19004	Ca6_v2.0	65840148	65842324
XLOC_018999	Ca_v2.0_19009	Ca6_v2.0	65879954	65882908
XLOC_019000	Ca_v2.0_19010	Ca6_v2.0	65885475	65886665
XLOC_019001	Ca_v2.0_19011	Ca6_v2.0	65890756	65891989
XLOC_019003	Ca_v2.0_19013	Ca6_v2.0	65912295	65921169
XLOC_019005	Ca_v2.0_19018	Ca6_v2.0	66011217	66017725
XLOC_019014	Ca_v2.0_19037	Ca6_v2.0	66303698	66307199
XLOC_019028	Ca_v2.0_19062	Ca6_v2.0	66765690	66767656
XLOC_019029	Ca_v2.0_19067	Ca6_v2.0	66770100	66801383
XLOC_019036	Ca_v2.0_19073	Ca6_v2.0	66864931	66867220
XLOC_019037	Ca_v2.0_19075	Ca6_v2.0	66873459	66876823
XLOC_019039	Ca_v2.0_19077	Ca6_v2.0	66905162	66908938
XLOC_019045	Ca_v2.0_19089	Ca6_v2.0	67015404	67015776
XLOC_019046	Ca_v2.0_19090	Ca6_v2.0	67015853	67016183
XLOC_019047	Ca_v2.0_19091	Ca6_v2.0	67023566	67033796
XLOC_019048	Ca_v2.0_19093	Ca6_v2.0	67043974	67047073
XLOC_019053	Ca_v2.0_19102	Ca6_v2.0	67256852	67260757
XLOC_019057	Ca_v2.0_19108	Ca6_v2.0	67330864	67352374
XLOC_019060	Ca_v2.0_19115	Ca6_v2.0	67368138	67369967
XLOC_019061	Ca_v2.0_19116	Ca6_v2.0	67371738	67386126
XLOC_019063	Ca_v2.0_19121	Ca6_v2.0	67447615	67449661
XLOC_019066	Ca_v2.0_19124	Ca6_v2.0	67472694	67475653
XLOC_019075	Ca_v2.0_19138	Ca6_v2.0	67568175	67570312
XLOC_019077	Ca_v2.0_19141	Ca6_v2.0	67610244	67614110

For Review Only

XLOC_019084	Ca_v2.0_19152,Ca_v2.0_191!	Ca6_v2.0	67741297	67784011
XLOC_019087	Ca_v2.0_19162	Ca6_v2.0	67896311	67897754
XLOC_019089	Ca_v2.0_19166	Ca6_v2.0	67955893	67959695
XLOC_019096	Ca_v2.0_19182	Ca6_v2.0	68242152	68249335
XLOC_019097	Ca_v2.0_19184,Ca_v2.0_191!	Ca6_v2.0	68380172	68465765
XLOC_019098	Ca_v2.0_19188	Ca6_v2.0	68521230	68529343
XLOC_019103	Ca_v2.0_19200	Ca6_v2.0	68675382	68680932
XLOC_019113	Ca_v2.0_19226	Ca6_v2.0	68859893	68861201
XLOC_019114	Ca_v2.0_19227	Ca6_v2.0	68864295	68867689
XLOC_019115	Ca_v2.0_19228	Ca6_v2.0	68868746	68871855
XLOC_019117	-	Ca6_v2.0	68929268	68930095
XLOC_019119	Ca_v2.0_19239	Ca6_v2.0	68958317	68959780
XLOC_019120	Ca_v2.0_19242	Ca6_v2.0	68977177	68979881
XLOC_019124	Ca_v2.0_19249	Ca6_v2.0	69036007	69038169
XLOC_019126	Ca_v2.0_19252	Ca6_v2.0	69046546	69051998
XLOC_019131	Ca_v2.0_19270	Ca6_v2.0	69209055	69209930
XLOC_019132	Ca_v2.0_19272	Ca6_v2.0	69234931	69236875
XLOC_019137	Ca_v2.0_19282	Ca6_v2.0	69380268	69386919
XLOC_019142	Ca_v2.0_19288	Ca6_v2.0	69513858	69517084
XLOC_019149	Ca_v2.0_19300	Ca6_v2.0	69646564	69649213
XLOC_019155	Ca_v2.0_19311	Ca6_v2.0	69714925	69719196
XLOC_019168	Ca_v2.0_19334	Ca6_v2.0	69910387	69919254
XLOC_019173	Ca_v2.0_19342	Ca6_v2.0	70024014	70027174
XLOC_019175	Ca_v2.0_19347	Ca6_v2.0	70075686	70079281
XLOC_019182	Ca_v2.0_19359	Ca6_v2.0	70265777	70270346
XLOC_019191	Ca_v2.0_19375	Ca6_v2.0	70388663	70423858
XLOC_019193	Ca_v2.0_19377	Ca6_v2.0	70441736	70446053
XLOC_019197	Ca_v2.0_19384	Ca6_v2.0	70570292	70571018
XLOC_019202	Ca_v2.0_19398	Ca6_v2.0	70707958	70717043
XLOC_019206	Ca_v2.0_15515	Ca6_v2.0	42400	50260
XLOC_019209	Ca_v2.0_15521	Ca6_v2.0	145931	146631
XLOC_019210	Ca_v2.0_15522	Ca6_v2.0	147421	155796
XLOC_019215	Ca_v2.0_15534	Ca6_v2.0	286625	289883
XLOC_019230	Ca_v2.0_15565	Ca6_v2.0	697324	699251
XLOC_019235	Ca_v2.0_15573	Ca6_v2.0	814740	820796
XLOC_019237	Ca_v2.0_15577	Ca6_v2.0	876195	881251
XLOC_019241	Ca_v2.0_15583	Ca6_v2.0	962225	964114
XLOC_019244	Ca_v2.0_15588	Ca6_v2.0	983946	991220
XLOC_019245	Ca_v2.0_15589	Ca6_v2.0	1002117	1005939
XLOC_019249	Ca_v2.0_15593	Ca6_v2.0	1043771	1045196
XLOC_019250	Ca_v2.0_15595,Ca_v2.0_155!	Ca6_v2.0	1069369	1075998
XLOC_019263	Ca_v2.0_15621	Ca6_v2.0	1377382	1381559
XLOC_019268	Ca_v2.0_15629	Ca6_v2.0	1484947	1488774
XLOC_019274	Ca_v2.0_15639	Ca6_v2.0	1590027	1592208
XLOC_019280	Ca_v2.0_15651,Ca_v2.0_156!	Ca6_v2.0	1733229	1747340
XLOC_019286	Ca_v2.0_15663	Ca6_v2.0	1819550	1826164

For Review Only

XLOC_019305	Ca_v2.0_15711	Ca6_v2.0	2183639	2188100
XLOC_019322	Ca_v2.0_15749	Ca6_v2.0	2505149	2505754
XLOC_019325	Ca_v2.0_15759	Ca6_v2.0	2574136	2575568
XLOC_019326	Ca_v2.0_15760	Ca6_v2.0	2579669	2581025
XLOC_019327	Ca_v2.0_15763	Ca6_v2.0	2606010	2613661
XLOC_019331	-	Ca6_v2.0	2655461	2659347
XLOC_019335	Ca_v2.0_15781	Ca6_v2.0	2954783	2968131
XLOC_019351	Ca_v2.0_15806	Ca6_v2.0	3246017	3247566
XLOC_019353	-	Ca6_v2.0	3261914	3263828
XLOC_019371	Ca_v2.0_15839	Ca6_v2.0	3514846	3519042
XLOC_019378	Ca_v2.0_15849	Ca6_v2.0	3591363	3597855
XLOC_019381	Ca_v2.0_15854	Ca6_v2.0	3636697	3639090
XLOC_019392	Ca_v2.0_15868	Ca6_v2.0	3748011	3751765
XLOC_019399	Ca_v2.0_15882	Ca6_v2.0	3879268	3883415
XLOC_019418	Ca_v2.0_15915	Ca6_v2.0	4236258	4239301
XLOC_019421	Ca_v2.0_15919	Ca6_v2.0	4266986	4271002
XLOC_019422	Ca_v2.0_15921	Ca6_v2.0	4284705	4288968
XLOC_019431	Ca_v2.0_15949	Ca6_v2.0	4495161	4501383
XLOC_019433	Ca_v2.0_15951	Ca6_v2.0	4514356	4516915
XLOC_019436	Ca_v2.0_15954	Ca6_v2.0	4526487	4545139
XLOC_019442	Ca_v2.0_15969	Ca6_v2.0	4650240	4651155
XLOC_019452	Ca_v2.0_15982	Ca6_v2.0	4749985	4752293
XLOC_019455	Ca_v2.0_15994	Ca6_v2.0	4869092	4870020
XLOC_019456	Ca_v2.0_15995	Ca6_v2.0	4885912	4895510
XLOC_019458	Ca_v2.0_16001	Ca6_v2.0	4959431	4961318
XLOC_019460	Ca_v2.0_16004	Ca6_v2.0	4997203	5000001
XLOC_019463	Ca_v2.0_16012	Ca6_v2.0	5063111	5066876
XLOC_019471	Ca_v2.0_16029	Ca6_v2.0	5292491	5294173
XLOC_019472	Ca_v2.0_16030,Ca_v2.0_160:	Ca6_v2.0	5306188	5311026
XLOC_019478	Ca_v2.0_16045	Ca6_v2.0	5435143	5438495
XLOC_019484	Ca_v2.0_16064	Ca6_v2.0	5591926	5593991
XLOC_019486	Ca_v2.0_16067	Ca6_v2.0	5608417	5611993
XLOC_019490	Ca_v2.0_16081	Ca6_v2.0	5738191	5738944
XLOC_019492	Ca_v2.0_16084	Ca6_v2.0	5793805	5796372
XLOC_019528	Ca_v2.0_16140	Ca6_v2.0	6350822	6354921
XLOC_019533	Ca_v2.0_16150	Ca6_v2.0	6444617	6450404
XLOC_019535	Ca_v2.0_16157	Ca6_v2.0	6530352	6538486
XLOC_019545	Ca_v2.0_16173	Ca6_v2.0	6670202	6672042
XLOC_019552	Ca_v2.0_16186	Ca6_v2.0	6780161	6787401
XLOC_019558	Ca_v2.0_16200	Ca6_v2.0	6915840	6917716
XLOC_019563	Ca_v2.0_16205	Ca6_v2.0	6949754	6956326
XLOC_019564	Ca_v2.0_16207	Ca6_v2.0	6962342	6972172
XLOC_019573	Ca_v2.0_16220	Ca6_v2.0	7107152	7108916
XLOC_019576	Ca_v2.0_16231	Ca6_v2.0	7171367	7180507
XLOC_019587	Ca_v2.0_16255	Ca6_v2.0	7445735	7449603
XLOC_019593	Ca_v2.0_16268	Ca6_v2.0	7534685	7535881

For Review Only

XLOC_019597	Ca_v2.0_16274	Ca6_v2.0	7580274	7582422
XLOC_019604	Ca_v2.0_16286	Ca6_v2.0	7681134	7684882
XLOC_019606	Ca_v2.0_16290	Ca6_v2.0	7722624	7727175
XLOC_019608	Ca_v2.0_16295	Ca6_v2.0	7756508	7760717
XLOC_019611	Ca_v2.0_16306	Ca6_v2.0	7864278	7867335
XLOC_019612	Ca_v2.0_16307	Ca6_v2.0	7869762	7872441
XLOC_019618	Ca_v2.0_16322,Ca_v2.0_163:	Ca6_v2.0	7978203	7980487
XLOC_019620	Ca_v2.0_16328	Ca6_v2.0	8022799	8028509
XLOC_019626	Ca_v2.0_16336	Ca6_v2.0	8087038	8091581
XLOC_019630	Ca_v2.0_16341	Ca6_v2.0	8127300	8137290
XLOC_019634	Ca_v2.0_16345	Ca6_v2.0	8224461	8226336
XLOC_019635	Ca_v2.0_16348	Ca6_v2.0	8236763	8241826
XLOC_019643	Ca_v2.0_16360	Ca6_v2.0	8340159	8344111
XLOC_019649	Ca_v2.0_16372	Ca6_v2.0	8432142	8436574
XLOC_019651	Ca_v2.0_16375	Ca6_v2.0	8462069	8476784
XLOC_019652	Ca_v2.0_16380	Ca6_v2.0	8482327	8483421
XLOC_019661	Ca_v2.0_16398	Ca6_v2.0	8658146	8660514
XLOC_019669	Ca_v2.0_16419	Ca6_v2.0	8917776	8920802
XLOC_019673	Ca_v2.0_16426	Ca6_v2.0	8978446	8983569
XLOC_019679	Ca_v2.0_16439	Ca6_v2.0	9088910	9092307
XLOC_019689	Ca_v2.0_16458	Ca6_v2.0	9282402	9285317
XLOC_019690	Ca_v2.0_16460	Ca6_v2.0	9295870	9304274
XLOC_019691	Ca_v2.0_16462	Ca6_v2.0	9317605	9319775
XLOC_019692	Ca_v2.0_16463	Ca6_v2.0	9326972	9329131
XLOC_019695	Ca_v2.0_16467	Ca6_v2.0	9353977	9356416
XLOC_019700	Ca_v2.0_16473	Ca6_v2.0	9381541	9384752
XLOC_019702	Ca_v2.0_16475	Ca6_v2.0	9390881	9392932
XLOC_019704	Ca_v2.0_16479	Ca6_v2.0	9415311	9416525
XLOC_019706	Ca_v2.0_16481	Ca6_v2.0	9436216	9437147
XLOC_019714	Ca_v2.0_16492	Ca6_v2.0	9562110	9563816
XLOC_019715	Ca_v2.0_16495	Ca6_v2.0	9588611	9593937
XLOC_019716	Ca_v2.0_16497	Ca6_v2.0	9609657	9611603
XLOC_019719	Ca_v2.0_16503	Ca6_v2.0	9678118	9686260
XLOC_019720	Ca_v2.0_16504	Ca6_v2.0	9687941	9689485
XLOC_019723	Ca_v2.0_16507	Ca6_v2.0	9702069	9704761
XLOC_019730	Ca_v2.0_16525	Ca6_v2.0	9880877	9882348
XLOC_019732	Ca_v2.0_16531	Ca6_v2.0	9931319	9937354
XLOC_019748	Ca_v2.0_16557	Ca6_v2.0	10166618	10168367
XLOC_019750	Ca_v2.0_16559	Ca6_v2.0	10174813	10180238
XLOC_019752	Ca_v2.0_16564,Ca_v2.0_165(	Ca6_v2.0	10233426	10242325
XLOC_019754	Ca_v2.0_16568	Ca6_v2.0	10255398	10258708
XLOC_019758	Ca_v2.0_16575	Ca6_v2.0	10312320	10316937
XLOC_019759	Ca_v2.0_16576,Ca_v2.0_165	Ca6_v2.0	10317909	10327424
XLOC_019761	Ca_v2.0_16580	Ca6_v2.0	10361876	10366475
XLOC_019763	Ca_v2.0_16583	Ca6_v2.0	10390477	10392990
XLOC_019766	Ca_v2.0_16591	Ca6_v2.0	10452092	10457008

For Review Only

XLOC_019770	Ca_v2.0_16601	Ca6_v2.0	10554106	10556439
XLOC_019774	Ca_v2.0_16608	Ca6_v2.0	10656321	10658792
XLOC_019776	Ca_v2.0_16611	Ca6_v2.0	10689213	10691712
XLOC_019779	Ca_v2.0_16615	Ca6_v2.0	10716265	10736041
XLOC_019784	Ca_v2.0_16622	Ca6_v2.0	10789240	10799194
XLOC_019790	Ca_v2.0_16634,Ca_v2.0_166:	Ca6_v2.0	10913600	10921256
XLOC_019795	-	Ca6_v2.0	10977089	10978822
XLOC_019801	Ca_v2.0_16654	Ca6_v2.0	11150424	11154368
XLOC_019802	Ca_v2.0_16655	Ca6_v2.0	11162799	11171165
XLOC_019808	Ca_v2.0_16669	Ca6_v2.0	11333544	11336380
XLOC_019819	Ca_v2.0_16696	Ca6_v2.0	11681591	11686306
XLOC_019821	Ca_v2.0_16701	Ca6_v2.0	11715680	11717045
XLOC_019822	Ca_v2.0_16708	Ca6_v2.0	11764917	11827974
XLOC_019823	Ca_v2.0_16711	Ca6_v2.0	11764917	11827974
XLOC_019827	Ca_v2.0_16716	Ca6_v2.0	11880779	11883018
XLOC_019829	Ca_v2.0_16718	Ca6_v2.0	11890670	11900106
XLOC_019832	Ca_v2.0_16723	Ca6_v2.0	11953614	11959666
XLOC_019841	Ca_v2.0_16736	Ca6_v2.0	12121842	12123889
XLOC_019846	Ca_v2.0_16752	Ca6_v2.0	12249534	12252740
XLOC_019856	-	Ca6_v2.0	12542160	12542588
XLOC_019868	Ca_v2.0_16794	Ca6_v2.0	12637449	12702996
XLOC_019870	Ca_v2.0_16796	Ca6_v2.0	12637449	12702996
XLOC_019871	Ca_v2.0_16798	Ca6_v2.0	12719491	12724687
XLOC_019872	Ca_v2.0_16799	Ca6_v2.0	12725057	12725858
XLOC_019876	Ca_v2.0_16806	Ca6_v2.0	12772614	12774462
XLOC_019883	Ca_v2.0_16819	Ca6_v2.0	12899797	12901420
XLOC_019890	Ca_v2.0_16830	Ca6_v2.0	12979437	12980442
XLOC_019892	Ca_v2.0_16836	Ca6_v2.0	13011813	13013248
XLOC_019894	-	Ca6_v2.0	13023800	13026622
XLOC_019910	Ca_v2.0_16860	Ca6_v2.0	13217906	13218733
XLOC_019917	Ca_v2.0_16871	Ca6_v2.0	13330027	13334659
XLOC_019918	Ca_v2.0_16873	Ca6_v2.0	13356509	13358013
XLOC_019928	Ca_v2.0_16892	Ca6_v2.0	13514480	13523414
XLOC_019929	Ca_v2.0_16893	Ca6_v2.0	13525953	13529330
XLOC_019934	Ca_v2.0_16907	Ca6_v2.0	13665048	13667848
XLOC_019950	Ca_v2.0_16938	Ca6_v2.0	13892161	13893011
XLOC_019961	Ca_v2.0_16958	Ca6_v2.0	13999561	14005807
XLOC_019979	Ca_v2.0_16997	Ca6_v2.0	14491131	14496623
XLOC_019989	Ca_v2.0_17013	Ca6_v2.0	14650684	14656759
XLOC_020000	Ca_v2.0_17025,Ca_v2.0_170:	Ca6_v2.0	14795706	14798344
XLOC_020004	Ca_v2.0_17031	Ca6_v2.0	14849790	14855462
XLOC_020008	Ca_v2.0_17038	Ca6_v2.0	14897082	14900906
XLOC_020010	Ca_v2.0_17044	Ca6_v2.0	15001042	15003860
XLOC_020014	Ca_v2.0_17050	Ca6_v2.0	15097073	15100650
XLOC_020019	Ca_v2.0_17058	Ca6_v2.0	15195254	15197322
XLOC_020020	Ca_v2.0_17060	Ca6_v2.0	15241582	15257935

For Review Only

XLOC_020023	Ca_v2.0_17066	Ca6_v2.0	15313501	15316097
XLOC_020026	Ca_v2.0_17074	Ca6_v2.0	15383719	15392814
XLOC_020028	Ca_v2.0_17080	Ca6_v2.0	15430786	15433002
XLOC_020033	Ca_v2.0_17087	Ca6_v2.0	15500559	15502215
XLOC_020047	Ca_v2.0_17117	Ca6_v2.0	15790750	15881517
XLOC_020048	Ca_v2.0_17118	Ca6_v2.0	15790750	15881517
XLOC_020052	Ca_v2.0_17125	Ca6_v2.0	15916082	15916433
XLOC_020056	Ca_v2.0_17129	Ca6_v2.0	15977350	15982289
XLOC_020062	Ca_v2.0_17140	Ca6_v2.0	16129998	16132537
XLOC_020063	Ca_v2.0_17141	Ca6_v2.0	16144925	16147349
XLOC_020065	Ca_v2.0_17145	Ca6_v2.0	16171458	16172199
XLOC_020077	Ca_v2.0_17171	Ca6_v2.0	16384639	16390953
XLOC_020082	Ca_v2.0_17180	Ca6_v2.0	16460920	16463991
XLOC_020094	Ca_v2.0_17200,Ca_v2.0_172(	Ca6_v2.0	16634746	16641385
XLOC_020098	Ca_v2.0_17215	Ca6_v2.0	16814541	16815708
XLOC_020106	Ca_v2.0_17233	Ca6_v2.0	17001847	17002883
XLOC_020109	Ca_v2.0_17236	Ca6_v2.0	17080045	17083247
XLOC_020124	Ca_v2.0_17270	Ca6_v2.0	17426584	17428004
XLOC_020125	Ca_v2.0_17271	Ca6_v2.0	17431076	17432489
XLOC_020131	Ca_v2.0_17283	Ca6_v2.0	17578610	17604054
XLOC_020135	Ca_v2.0_17289	Ca6_v2.0	17652268	17660880
XLOC_020146	-	Ca6_v2.0	17935415	17937727
XLOC_020149	Ca_v2.0_17322	Ca6_v2.0	17972967	17977792
XLOC_020152	Ca_v2.0_17327	Ca6_v2.0	18036150	18039152
XLOC_020157	Ca_v2.0_17336	Ca6_v2.0	18131260	18134852
XLOC_020186	Ca_v2.0_17394	Ca6_v2.0	18821922	18825412
XLOC_020188	Ca_v2.0_17396	Ca6_v2.0	18842116	18851106
XLOC_020211	Ca_v2.0_17441	Ca6_v2.0	19271309	19272537
XLOC_020218	Ca_v2.0_17454	Ca6_v2.0	19432162	19451294
XLOC_020219	Ca_v2.0_17455	Ca6_v2.0	19432162	19451294
XLOC_020220	Ca_v2.0_17456	Ca6_v2.0	19456697	19458462
XLOC_020221	Ca_v2.0_17459	Ca6_v2.0	19494164	19498990
XLOC_020234	Ca_v2.0_17485	Ca6_v2.0	19718592	19722644
XLOC_020244	Ca_v2.0_17498	Ca6_v2.0	19858817	19860774
XLOC_020247	Ca_v2.0_17506	Ca6_v2.0	19916720	19934911
XLOC_020254	Ca_v2.0_17526	Ca6_v2.0	20136330	20141970
XLOC_020257	Ca_v2.0_17534	Ca6_v2.0	20209893	20211382
XLOC_020258	Ca_v2.0_17535	Ca6_v2.0	20222317	20228349
XLOC_020266	Ca_v2.0_17553	Ca6_v2.0	20379933	20392030
XLOC_020270	Ca_v2.0_17566	Ca6_v2.0	20475990	20484777
XLOC_020278	Ca_v2.0_17580	Ca6_v2.0	20628373	20633319
XLOC_020279	-	Ca6_v2.0	20671859	20674995
XLOC_020280	Ca_v2.0_17583	Ca6_v2.0	20739558	20741633
XLOC_020296	Ca_v2.0_17605	Ca6_v2.0	21309485	21357400
XLOC_020301	Ca_v2.0_17612	Ca6_v2.0	21433041	21435334
XLOC_020304	Ca_v2.0_17618	Ca6_v2.0	21525668	21528597

For Review Only

XLOC_020305	Ca_v2.0_17619	Ca6_v2.0	21538772	21540932
XLOC_020306	Ca_v2.0_17623	Ca6_v2.0	21592169	21596466
XLOC_020315	Ca_v2.0_17646	Ca6_v2.0	21776403	21787792
XLOC_020331	Ca_v2.0_17678	Ca6_v2.0	22155630	22162537
XLOC_020341	Ca_v2.0_17693	Ca6_v2.0	22302113	22312890
XLOC_020348	Ca_v2.0_17713	Ca6_v2.0	22565412	22568828
XLOC_020363	Ca_v2.0_17746	Ca6_v2.0	23050369	23066364
XLOC_020367	Ca_v2.0_17751	Ca6_v2.0	23122019	23126680
XLOC_020369	Ca_v2.0_17754	Ca6_v2.0	23167659	23174117
XLOC_020373	Ca_v2.0_17759	Ca6_v2.0	23242563	23247393
XLOC_020375	Ca_v2.0_17762	Ca6_v2.0	23277292	23279623
XLOC_020380	Ca_v2.0_17769	Ca6_v2.0	23460716	23469530
XLOC_020393	Ca_v2.0_17793	Ca6_v2.0	23942080	23944739
XLOC_020394	Ca_v2.0_17795	Ca6_v2.0	24006949	24011843
XLOC_020410	Ca_v2.0_17829	Ca6_v2.0	24665529	24671093
XLOC_020411	Ca_v2.0_17830	Ca6_v2.0	24726632	24729389
XLOC_020412	Ca_v2.0_17831	Ca6_v2.0	24735649	24737580
XLOC_020413	-	Ca6_v2.0	24737679	24737942
XLOC_020414	Ca_v2.0_17833	Ca6_v2.0	24875380	24876890
XLOC_020415	Ca_v2.0_17835,Ca_v2.0_178	Ca6_v2.0	24933569	24943421
XLOC_020423	Ca_v2.0_17849	Ca6_v2.0	25149095	25159020
XLOC_020426	Ca_v2.0_17855	Ca6_v2.0	25326103	25329455
XLOC_020427	Ca_v2.0_17856	Ca6_v2.0	25359888	25362166
XLOC_020429	Ca_v2.0_17859,Ca_v2.0_178	Ca6_v2.0	25422125	25427220
XLOC_020433	Ca_v2.0_17869	Ca6_v2.0	25639389	25639995
XLOC_020434	Ca_v2.0_17870	Ca6_v2.0	25644414	25649352
XLOC_020435	Ca_v2.0_17871	Ca6_v2.0	25651909	25652740
XLOC_020438	Ca_v2.0_17876	Ca6_v2.0	25703880	25706161
XLOC_020450	Ca_v2.0_17903	Ca6_v2.0	26230505	26235039
XLOC_020464	Ca_v2.0_17920	Ca6_v2.0	26566011	26566572
XLOC_020465	Ca_v2.0_17921	Ca6_v2.0	26577558	26578247
XLOC_020480	Ca_v2.0_17952	Ca6_v2.0	27063293	27067413
XLOC_020481	Ca_v2.0_17953	Ca6_v2.0	27121843	27122698
XLOC_020484	Ca_v2.0_17957	Ca6_v2.0	27222791	27223455
XLOC_020485	Ca_v2.0_17958	Ca6_v2.0	27242159	27242928
XLOC_020504	Ca_v2.0_17997	Ca6_v2.0	28205558	28211449
XLOC_020507	-	Ca6_v2.0	28484345	28486414
XLOC_020508	-	Ca6_v2.0	28496654	28498253
XLOC_020509	Ca_v2.0_18007	Ca6_v2.0	28645882	28648257
XLOC_020511	Ca_v2.0_18009	Ca6_v2.0	28661935	28665300
XLOC_020515	Ca_v2.0_18015	Ca6_v2.0	28925352	28928529
XLOC_020542	Ca_v2.0_18081	Ca6_v2.0	30685185	30687686
XLOC_020546	Ca_v2.0_18089	Ca6_v2.0	30788480	30800353
XLOC_020549	Ca_v2.0_18093	Ca6_v2.0	30914910	30920463
XLOC_020561	Ca_v2.0_18110	Ca6_v2.0	31705452	31711739
XLOC_020574	Ca_v2.0_18135	Ca6_v2.0	32456969	32463643

For Review Only

XLOC_020577	Ca_v2.0_18145	Ca6_v2.0	32970257	32975052
XLOC_020579	Ca_v2.0_18149	Ca6_v2.0	33153923	33155889
XLOC_020590	Ca_v2.0_18161	Ca6_v2.0	33564215	33571657
XLOC_020608	Ca_v2.0_18184	Ca6_v2.0	34727041	34732389
XLOC_020617	Ca_v2.0_18202	Ca6_v2.0	35565883	35572163
XLOC_020618	Ca_v2.0_18203	Ca6_v2.0	35612862	35617663
XLOC_020619	Ca_v2.0_18204	Ca6_v2.0	35632348	35636213
XLOC_020624	Ca_v2.0_18214	Ca6_v2.0	35823620	35829397
XLOC_020631	Ca_v2.0_18228	Ca6_v2.0	36536860	36542439
XLOC_020633	Ca_v2.0_18230	Ca6_v2.0	36588987	36593187
XLOC_020634	Ca_v2.0_18231	Ca6_v2.0	36616765	36643732
XLOC_020649	Ca_v2.0_18255	Ca6_v2.0	37615503	37675086
XLOC_020659	Ca_v2.0_18271,Ca_v2.0_182	Ca6_v2.0	38849461	38851589
XLOC_020666	Ca_v2.0_18280	Ca6_v2.0	39493834	39498582
XLOC_020668	-	Ca6_v2.0	39656268	39657276
XLOC_020677	Ca_v2.0_18296	Ca6_v2.0	40009286	40010077
XLOC_020697	-	Ca6_v2.0	41895032	41896042
XLOC_020740	Ca_v2.0_18412	Ca6_v2.0	46314171	46338606
XLOC_020742	Ca_v2.0_18413	Ca6_v2.0	46377374	46379496
XLOC_020747	Ca_v2.0_18434	Ca6_v2.0	47581363	47582978
XLOC_020761	Ca_v2.0_18484	Ca6_v2.0	50355555	50359240
XLOC_020767	Ca_v2.0_18499	Ca6_v2.0	50977307	50980217
XLOC_020771	Ca_v2.0_18512	Ca6_v2.0	51399058	51410131
XLOC_020777	Ca_v2.0_18523	Ca6_v2.0	51634116	51635488
XLOC_020779	Ca_v2.0_18528	Ca6_v2.0	51849792	51858335
XLOC_020792	Ca_v2.0_18551	Ca6_v2.0	52884372	52885692
XLOC_020808	Ca_v2.0_18585	Ca6_v2.0	54623413	54626391
XLOC_020834	Ca_v2.0_18637	Ca6_v2.0	56881292	56882712
XLOC_020845	Ca_v2.0_18656	Ca6_v2.0	57522451	57526176
XLOC_020864	Ca_v2.0_18683	Ca6_v2.0	58350094	58351241
XLOC_020870	Ca_v2.0_18696	Ca6_v2.0	58726394	58727542
XLOC_020881	Ca_v2.0_18709	Ca6_v2.0	58946275	58948756
XLOC_020882	Ca_v2.0_18710	Ca6_v2.0	58954646	58956936
XLOC_020883	Ca_v2.0_18711	Ca6_v2.0	59070336	59071987
XLOC_020890	Ca_v2.0_18721	Ca6_v2.0	59308008	59309216
XLOC_020892	Ca_v2.0_18723	Ca6_v2.0	59326589	59329077
XLOC_020898	Ca_v2.0_18734	Ca6_v2.0	59642041	59651472
XLOC_020901	Ca_v2.0_18745	Ca6_v2.0	59941574	59943985
XLOC_020903	-	Ca6_v2.0	60004544	60006300
XLOC_020905	Ca_v2.0_18756	Ca6_v2.0	60159182	60160925
XLOC_020911	Ca_v2.0_18767	Ca6_v2.0	60377470	60378114
XLOC_020922	Ca_v2.0_18791,Ca_v2.0_187	Ca6_v2.0	61420919	61424649
XLOC_020927	Ca_v2.0_18803	Ca6_v2.0	61646329	61650836
XLOC_020939	Ca_v2.0_18834	Ca6_v2.0	62332210	62332720
XLOC_020942	Ca_v2.0_18837	Ca6_v2.0	62347740	62349623
XLOC_020945	Ca_v2.0_18842	Ca6_v2.0	62411000	62413526

For Review Only

XLOC_020946	Ca_v2.0_18843	Ca6_v2.0	62419611	62420978
XLOC_020985	Ca_v2.0_18924	Ca6_v2.0	64124163	64125340
XLOC_020990	Ca_v2.0_18934	Ca6_v2.0	64312728	64313862
XLOC_020997	Ca_v2.0_18944	Ca6_v2.0	64447877	64450794
XLOC_021002	Ca_v2.0_18957	Ca6_v2.0	64675076	64677584
XLOC_021012	Ca_v2.0_18989	Ca6_v2.0	65530999	65536173
XLOC_021018	Ca_v2.0_19000	Ca6_v2.0	65792465	65795883
XLOC_021025	Ca_v2.0_19013	Ca6_v2.0	65912295	65921169
XLOC_021026	Ca_v2.0_19014	Ca6_v2.0	65912295	65921169
XLOC_021031	Ca_v2.0_19021	Ca6_v2.0	66048992	66049769
XLOC_021037	Ca_v2.0_19028	Ca6_v2.0	66157215	66159173
XLOC_021038	Ca_v2.0_19029	Ca6_v2.0	66169706	66174107
XLOC_021039	Ca_v2.0_19032	Ca6_v2.0	66220395	66222881
XLOC_021041	Ca_v2.0_19037	Ca6_v2.0	66303698	66307199
XLOC_021042	Ca_v2.0_19038	Ca6_v2.0	66320049	66323649
XLOC_021043	Ca_v2.0_19040	Ca6_v2.0	66371245	66376181
XLOC_021045	Ca_v2.0_19047	Ca6_v2.0	66473564	66485865
XLOC_021047	Ca_v2.0_19052	Ca6_v2.0	66582805	66587116
XLOC_021055	Ca_v2.0_19063	Ca6_v2.0	66770100	66801383
XLOC_021060	Ca_v2.0_19069	Ca6_v2.0	66808884	66812134
XLOC_021062	Ca_v2.0_19074	Ca6_v2.0	66867348	66870341
XLOC_021063	Ca_v2.0_19075	Ca6_v2.0	66873459	66876823
XLOC_021065	Ca_v2.0_19080	Ca6_v2.0	66934104	66949740
XLOC_021076	Ca_v2.0_19104	Ca6_v2.0	67277623	67280565
XLOC_021077	Ca_v2.0_19112	Ca6_v2.0	67330864	67352374
XLOC_021080	Ca_v2.0_19113	Ca6_v2.0	67357661	67360400
XLOC_021082	Ca_v2.0_19116,Ca_v2.0_191	Ca6_v2.0	67371738	67386126
XLOC_021089	Ca_v2.0_19134	Ca6_v2.0	67551087	67553403
XLOC_021093	Ca_v2.0_19142	Ca6_v2.0	67610244	67614110
XLOC_021097	Ca_v2.0_19152	Ca6_v2.0	67741297	67784011
XLOC_021098	Ca_v2.0_19153	Ca6_v2.0	67741297	67784011
XLOC_021099	Ca_v2.0_19154	Ca6_v2.0	67741297	67784011
XLOC_021106	Ca_v2.0_19165	Ca6_v2.0	67928488	67929797
XLOC_021109	Ca_v2.0_19170	Ca6_v2.0	67996803	68006513
XLOC_021115	-	Ca6_v2.0	68123731	68126384
XLOC_021118	Ca_v2.0_19179	Ca6_v2.0	68175464	68178927
XLOC_021119	Ca_v2.0_19180	Ca6_v2.0	68198126	68206478
XLOC_021121	Ca_v2.0_19182	Ca6_v2.0	68242152	68249335
XLOC_021124	Ca_v2.0_19185	Ca6_v2.0	68380172	68465765
XLOC_021125	Ca_v2.0_19185	Ca6_v2.0	68380172	68465765
XLOC_021126	-	Ca6_v2.0	68380172	68465765
XLOC_021128	Ca_v2.0_19187	Ca6_v2.0	68488815	68495186
XLOC_021135	Ca_v2.0_19201	Ca6_v2.0	68675382	68680932
XLOC_021143	Ca_v2.0_19213	Ca6_v2.0	68768149	68774113
XLOC_021146	Ca_v2.0_19216	Ca6_v2.0	68788461	68791979
XLOC_021151	Ca_v2.0_19230	Ca6_v2.0	68877230	68878428

For Review Only

XLOC_021154	Ca_v2.0_19237	Ca6_v2.0	68934499	68936096
XLOC_021159	Ca_v2.0_19248	Ca6_v2.0	69036007	69038169
XLOC_021160	Ca_v2.0_19250	Ca6_v2.0	69039316	69041964
XLOC_021161	Ca_v2.0_19253	Ca6_v2.0	69046546	69051998
XLOC_021165	Ca_v2.0_19258	Ca6_v2.0	69087001	69090217
XLOC_021168	Ca_v2.0_19263,Ca_v2.0_192(	Ca6_v2.0	69132515	69138627
XLOC_021169	Ca_v2.0_19265	Ca6_v2.0	69140951	69146459
XLOC_021173	Ca_v2.0_19271	Ca6_v2.0	69226227	69227515
XLOC_021174	Ca_v2.0_19272	Ca6_v2.0	69234931	69236875
XLOC_021176	Ca_v2.0_19276	Ca6_v2.0	69318732	69324054
XLOC_021206	Ca_v2.0_19339	Ca6_v2.0	69976573	69979389
XLOC_021211	Ca_v2.0_19349	Ca6_v2.0	70126422	70127099
XLOC_021223	Ca_v2.0_19365	Ca6_v2.0	70336241	70340951
XLOC_021246	-	Ca6_v2.0	847338	849581
XLOC_021248	-	Ca6_v2.0	1011829	1012811
XLOC_021253	-	Ca6_v2.0	2061096	2062274
XLOC_021258	-	Ca6_v2.0	4870619	4870892
XLOC_021261	-	Ca6_v2.0	5109718	5109966
XLOC_021268	-	Ca6_v2.0	7621420	7622153
XLOC_021278	-	Ca6_v2.0	9443875	9444858
XLOC_021279	-	Ca6_v2.0	9463396	9464121
XLOC_021282	-	Ca6_v2.0	10524714	10525289
XLOC_021287	-	Ca6_v2.0	11873748	11874702
XLOC_021292	-	Ca6_v2.0	12921975	12922615
XLOC_021305	-	Ca6_v2.0	18347982	18348428
XLOC_021307	-	Ca6_v2.0	18438478	18439656
XLOC_021313	-	Ca6_v2.0	19503735	19504541
XLOC_021318	-	Ca6_v2.0	21139395	21140040
XLOC_021323	-	Ca6_v2.0	22951602	22952828
XLOC_021333	-	Ca6_v2.0	25998767	25998968
XLOC_021334	-	Ca6_v2.0	26569567	26570323
XLOC_021337	-	Ca6_v2.0	27434600	27435040
XLOC_021340	-	Ca6_v2.0	29921986	29923450
XLOC_021346	-	Ca6_v2.0	33020164	33021240
XLOC_021353	-	Ca6_v2.0	44367355	44367539
XLOC_021354	-	Ca6_v2.0	44923346	44923678
XLOC_021359	-	Ca6_v2.0	46671265	46671779
XLOC_021361	-	Ca6_v2.0	46876012	46876195
XLOC_021365	Ca_v2.0_18488	Ca6_v2.0	50493445	50517938
XLOC_021369	-	Ca6_v2.0	51664291	51666322
XLOC_021382	-	Ca6_v2.0	58087043	58087212
XLOC_021386	-	Ca6_v2.0	59024519	59025652
XLOC_021390	-	Ca6_v2.0	59807009	59808245
XLOC_021394	-	Ca6_v2.0	61108055	61109240
XLOC_021408	-	Ca6_v2.0	66927147	66928024
XLOC_021410	-	Ca6_v2.0	68846577	68847563

For Review Only

XLOC_021411	-	Ca6_v2.0	68924803	68925667
XLOC_021423	Ca_v2.0_19412,Ca_v2.0_194	Ca7_v2.0	164577	172758
XLOC_021427	Ca_v2.0_19418	Ca7_v2.0	210846	214999
XLOC_021430	Ca_v2.0_19423	Ca7_v2.0	260786	270129
XLOC_021434	Ca_v2.0_19433	Ca7_v2.0	364866	366705
XLOC_021440	Ca_v2.0_19451	Ca7_v2.0	523279	531213
XLOC_021441	Ca_v2.0_19456	Ca7_v2.0	570280	577100
XLOC_021442	Ca_v2.0_19458	Ca7_v2.0	582461	587751
XLOC_021447	Ca_v2.0_19474	Ca7_v2.0	713314	718291
XLOC_021448	Ca_v2.0_19475	Ca7_v2.0	725585	727677
XLOC_021449	Ca_v2.0_19476	Ca7_v2.0	730930	731795
XLOC_021463	Ca_v2.0_19505	Ca7_v2.0	945944	952694
XLOC_021467	Ca_v2.0_19511	Ca7_v2.0	993024	995510
XLOC_021472	Ca_v2.0_19515	Ca7_v2.0	1039090	1040499
XLOC_021473	Ca_v2.0_19516	Ca7_v2.0	1050131	1051580
XLOC_021476	Ca_v2.0_19524	Ca7_v2.0	1098350	1103816
XLOC_021480	Ca_v2.0_19530	Ca7_v2.0	1145472	1147655
XLOC_021481	Ca_v2.0_19534	Ca7_v2.0	1185108	1185892
XLOC_021482	Ca_v2.0_19536	Ca7_v2.0	1190434	1196801
XLOC_021492	Ca_v2.0_19561	Ca7_v2.0	1366500	1370580
XLOC_021495	Ca_v2.0_19568	Ca7_v2.0	1433720	1443330
XLOC_021505	Ca_v2.0_19588	Ca7_v2.0	1593897	1596382
XLOC_021507	Ca_v2.0_19590	Ca7_v2.0	1613295	1622419
XLOC_021513	Ca_v2.0_19603	Ca7_v2.0	1712055	1712804
XLOC_021516	Ca_v2.0_19606	Ca7_v2.0	1744506	1747834
XLOC_021517	Ca_v2.0_19607,Ca_v2.0_196(	Ca7_v2.0	1756437	1761837
XLOC_021518	Ca_v2.0_19609	Ca7_v2.0	1770756	1773930
XLOC_021519	Ca_v2.0_19611	Ca7_v2.0	1792080	1797721
XLOC_021521	Ca_v2.0_19613,Ca_v2.0_196	Ca7_v2.0	1833668	1835820
XLOC_021522	Ca_v2.0_19615	Ca7_v2.0	1842540	1845504
XLOC_021527	-	Ca7_v2.0	1892421	1892984
XLOC_021530	Ca_v2.0_19624	Ca7_v2.0	1917733	1921271
XLOC_021533	-	Ca7_v2.0	2007134	2007690
XLOC_021538	Ca_v2.0_19638	Ca7_v2.0	2095469	2096415
XLOC_021539	Ca_v2.0_19639	Ca7_v2.0	2097716	2098593
XLOC_021544	Ca_v2.0_19645	Ca7_v2.0	2156624	2165520
XLOC_021545	Ca_v2.0_19647	Ca7_v2.0	2167571	2175151
XLOC_021550	Ca_v2.0_19652	Ca7_v2.0	2214992	2215862
XLOC_021560	Ca_v2.0_19668	Ca7_v2.0	2373134	2377446
XLOC_021562	Ca_v2.0_19670	Ca7_v2.0	2415790	2422090
XLOC_021568	Ca_v2.0_19679	Ca7_v2.0	2521311	2523543
XLOC_021571	Ca_v2.0_19686	Ca7_v2.0	2571302	2580062
XLOC_021583	Ca_v2.0_19709	Ca7_v2.0	2768126	2770088
XLOC_021586	Ca_v2.0_19714	Ca7_v2.0	2828106	2829761
XLOC_021588	Ca_v2.0_19718	Ca7_v2.0	2865940	2868101
XLOC_021590	Ca_v2.0_19726	Ca7_v2.0	2927024	2928432

For Review Only

XLOC_021594	Ca_v2.0_19734	Ca7_v2.0	2988557	2989019
XLOC_021595	Ca_v2.0_19735	Ca7_v2.0	2993676	2997076
XLOC_021597	Ca_v2.0_19742	Ca7_v2.0	3044789	3050022
XLOC_021600	Ca_v2.0_19747	Ca7_v2.0	3068016	3070470
XLOC_021603	Ca_v2.0_19752	Ca7_v2.0	3111834	3115371
XLOC_021608	Ca_v2.0_19762	Ca7_v2.0	3185967	3186857
XLOC_021613	Ca_v2.0_19770	Ca7_v2.0	3242176	3245384
XLOC_021615	Ca_v2.0_19778	Ca7_v2.0	3292322	3295279
XLOC_021616	Ca_v2.0_19779	Ca7_v2.0	3321738	3324469
XLOC_021618	Ca_v2.0_19783	Ca7_v2.0	3356306	3363980
XLOC_021620	Ca_v2.0_19788	Ca7_v2.0	3384867	3387022
XLOC_021626	Ca_v2.0_19796	Ca7_v2.0	3449967	3456609
XLOC_021629	Ca_v2.0_19802	Ca7_v2.0	3491733	3493563
XLOC_021631	Ca_v2.0_19805	Ca7_v2.0	3544032	3549407
XLOC_021638	Ca_v2.0_19816	Ca7_v2.0	3651978	3654469
XLOC_021639	Ca_v2.0_19819	Ca7_v2.0	3680731	3692321
XLOC_021647	Ca_v2.0_19836	Ca7_v2.0	3828453	3831850
XLOC_021653	Ca_v2.0_19850	Ca7_v2.0	3932891	3942947
XLOC_021664	Ca_v2.0_19870	Ca7_v2.0	4160838	4163522
XLOC_021666	Ca_v2.0_19873	Ca7_v2.0	4189526	4191599
XLOC_021667	Ca_v2.0_19874	Ca7_v2.0	4198410	4205663
XLOC_021670	Ca_v2.0_19878	Ca7_v2.0	4226906	4232517
XLOC_021672	Ca_v2.0_19884	Ca7_v2.0	4252487	4279355
XLOC_021673	Ca_v2.0_19881	Ca7_v2.0	4252487	4279355
XLOC_021675	Ca_v2.0_19883	Ca7_v2.0	4252487	4279355
XLOC_021676	Ca_v2.0_19884	Ca7_v2.0	4252487	4279355
XLOC_021693	Ca_v2.0_19917	Ca7_v2.0	4534390	4537383
XLOC_021698	Ca_v2.0_19925	Ca7_v2.0	4621019	4624580
XLOC_021702	Ca_v2.0_19931	Ca7_v2.0	4674774	4677515
XLOC_021703	Ca_v2.0_19934	Ca7_v2.0	4714806	4715845
XLOC_021705	Ca_v2.0_19938,Ca_v2.0_199:	Ca7_v2.0	4732340	4735829
XLOC_021719	Ca_v2.0_19978	Ca7_v2.0	5001613	5003840
XLOC_021734	Ca_v2.0_20007	Ca7_v2.0	5283842	5286260
XLOC_021743	Ca_v2.0_20022	Ca7_v2.0	5388116	5392498
XLOC_021745	Ca_v2.0_20024	Ca7_v2.0	5404328	5405449
XLOC_021746	Ca_v2.0_20025	Ca7_v2.0	5407846	5408705
XLOC_021751	Ca_v2.0_20034	Ca7_v2.0	5476808	5478902
XLOC_021752	Ca_v2.0_20035	Ca7_v2.0	5482021	5484117
XLOC_021754	Ca_v2.0_20037	Ca7_v2.0	5485000	5495898
XLOC_021756	Ca_v2.0_20041	Ca7_v2.0	5529368	5535173
XLOC_021758	Ca_v2.0_20047	Ca7_v2.0	5593016	5597003
XLOC_021764	Ca_v2.0_20056	Ca7_v2.0	5685539	5688158
XLOC_021765	Ca_v2.0_20058	Ca7_v2.0	5708500	5710106
XLOC_021766	Ca_v2.0_20059	Ca7_v2.0	5726061	5730587
XLOC_021772	Ca_v2.0_20071	Ca7_v2.0	5849546	5862470
XLOC_021775	Ca_v2.0_20077	Ca7_v2.0	5909787	5910798

For Review Only

XLOC_021779	Ca_v2.0_20087	Ca7_v2.0	5996594	5997489
XLOC_021788	Ca_v2.0_20104	Ca7_v2.0	6149686	6153581
XLOC_021796	Ca_v2.0_20117	Ca7_v2.0	6228795	6233747
XLOC_021800	Ca_v2.0_20122	Ca7_v2.0	6280673	6283421
XLOC_021801	Ca_v2.0_20123	Ca7_v2.0	6285451	6287276
XLOC_021805	Ca_v2.0_20128	Ca7_v2.0	6341110	6343747
XLOC_021808	-	Ca7_v2.0	6402137	6405211
XLOC_021809	Ca_v2.0_20134	Ca7_v2.0	6436949	6439468
XLOC_021818	Ca_v2.0_20144	Ca7_v2.0	6590247	6591994
XLOC_021826	Ca_v2.0_20163	Ca7_v2.0	6764289	6767742
XLOC_021830	Ca_v2.0_20168	Ca7_v2.0	6790663	6792751
XLOC_021833	Ca_v2.0_20180	Ca7_v2.0	6889329	6890547
XLOC_021850	-	Ca7_v2.0	7185811	7186769
XLOC_021851	Ca_v2.0_20215	Ca7_v2.0	7205165	7208571
XLOC_021856	Ca_v2.0_20227	Ca7_v2.0	7355650	7356480
XLOC_021863	Ca_v2.0_20240	Ca7_v2.0	7457460	7463576
XLOC_021874	Ca_v2.0_20256	Ca7_v2.0	7529482	7558125
XLOC_021902	Ca_v2.0_20312	Ca7_v2.0	8060051	8063557
XLOC_021904	Ca_v2.0_20318	Ca7_v2.0	8112436	8114480
XLOC_021913	Ca_v2.0_20341	Ca7_v2.0	8292779	8293598
XLOC_021917	Ca_v2.0_20356	Ca7_v2.0	8410184	8414547
XLOC_021918	Ca_v2.0_20357	Ca7_v2.0	8421367	8423996
XLOC_021934	Ca_v2.0_20389	Ca7_v2.0	8704790	8718230
XLOC_021942	Ca_v2.0_20401	Ca7_v2.0	8836740	8837462
XLOC_021943	Ca_v2.0_20402	Ca7_v2.0	8844342	8846875
XLOC_021950	Ca_v2.0_20413	Ca7_v2.0	8952115	8953671
XLOC_021956	Ca_v2.0_20418	Ca7_v2.0	9010635	9017292
XLOC_021959	-	Ca7_v2.0	9085899	9099303
XLOC_021963	Ca_v2.0_20430	Ca7_v2.0	9162507	9164139
XLOC_021964	Ca_v2.0_20431	Ca7_v2.0	9170412	9175218
XLOC_021965	-	Ca7_v2.0	9175709	9176315
XLOC_021967	Ca_v2.0_20435	Ca7_v2.0	9207150	9216734
XLOC_021969	Ca_v2.0_20438,Ca_v2.0_204:	Ca7_v2.0	9226017	9233642
XLOC_021973	Ca_v2.0_20446	Ca7_v2.0	9255860	9261259
XLOC_021974	Ca_v2.0_20447	Ca7_v2.0	9265391	9267899
XLOC_021993	Ca_v2.0_20477	Ca7_v2.0	9516152	9519851
XLOC_021998	Ca_v2.0_20484	Ca7_v2.0	9633601	9644349
XLOC_022003	Ca_v2.0_20495	Ca7_v2.0	9714823	9727309
XLOC_022004	Ca_v2.0_20496	Ca7_v2.0	9736586	9738096
XLOC_022006	Ca_v2.0_20499	Ca7_v2.0	9747812	9748443
XLOC_022009	Ca_v2.0_20505	Ca7_v2.0	9797420	9799423
XLOC_022011	Ca_v2.0_20512	Ca7_v2.0	9866930	9871215
XLOC_022032	Ca_v2.0_20548	Ca7_v2.0	10226439	10227302
XLOC_022037	Ca_v2.0_20556	Ca7_v2.0	10298248	10303467
XLOC_022043	Ca_v2.0_20564	Ca7_v2.0	10432594	10435031
XLOC_022050	Ca_v2.0_20575	Ca7_v2.0	10519469	10520672

For Review Only

XLOC_022051	Ca_v2.0_20576	Ca7_v2.0	10523682	10524535
XLOC_022060	Ca_v2.0_20590	Ca7_v2.0	10658102	10662137
XLOC_022063	Ca_v2.0_20594	Ca7_v2.0	10692300	10693907
XLOC_022067	Ca_v2.0_20606	Ca7_v2.0	10822865	10826818
XLOC_022072	Ca_v2.0_20613	Ca7_v2.0	10883977	10886900
XLOC_022080	Ca_v2.0_20627	Ca7_v2.0	11120481	11121505
XLOC_022081	Ca_v2.0_20629	Ca7_v2.0	11137363	11138357
XLOC_022082	Ca_v2.0_20631	Ca7_v2.0	11147159	11152700
XLOC_022088	Ca_v2.0_20645	Ca7_v2.0	11260507	11261400
XLOC_022097	Ca_v2.0_20664	Ca7_v2.0	11443842	11444867
XLOC_022101	Ca_v2.0_20672	Ca7_v2.0	11545361	11548309
XLOC_022102	Ca_v2.0_20675	Ca7_v2.0	11591409	11592776
XLOC_022104	Ca_v2.0_20680	Ca7_v2.0	11653192	11655064
XLOC_022107	Ca_v2.0_20685	Ca7_v2.0	11684790	11686860
XLOC_022111	Ca_v2.0_20695	Ca7_v2.0	11762773	11842905
XLOC_022120	Ca_v2.0_20705	Ca7_v2.0	11910699	11914136
XLOC_022127	Ca_v2.0_20713	Ca7_v2.0	12048355	12049470
XLOC_022132	Ca_v2.0_20719	Ca7_v2.0	12095231	12097399
XLOC_022134	Ca_v2.0_20724	Ca7_v2.0	12138242	12144236
XLOC_022136	Ca_v2.0_20727	Ca7_v2.0	12180056	12186039
XLOC_022141	Ca_v2.0_20739	Ca7_v2.0	12328311	12330718
XLOC_022146	Ca_v2.0_20746	Ca7_v2.0	12444320	12447372
XLOC_022148	Ca_v2.0_20748	Ca7_v2.0	12461531	12465972
XLOC_022160	Ca_v2.0_20769	Ca7_v2.0	12761664	12763474
XLOC_022166	Ca_v2.0_20785	Ca7_v2.0	12923878	12925328
XLOC_022167	Ca_v2.0_20786	Ca7_v2.0	12952683	12955459
XLOC_022169	Ca_v2.0_20789	Ca7_v2.0	12991619	12994211
XLOC_022176	Ca_v2.0_20799	Ca7_v2.0	13100982	13103061
XLOC_022178	Ca_v2.0_20807	Ca7_v2.0	13165601	13167613
XLOC_022182	Ca_v2.0_20812	Ca7_v2.0	13227207	13227997
XLOC_022192	Ca_v2.0_20829	Ca7_v2.0	13499827	13503476
XLOC_022195	Ca_v2.0_20833	Ca7_v2.0	13554739	13556394
XLOC_022213	Ca_v2.0_20858	Ca7_v2.0	13885391	13886045
XLOC_022214	Ca_v2.0_20859	Ca7_v2.0	13898124	13898809
XLOC_022224	Ca_v2.0_20877	Ca7_v2.0	14169084	14173468
XLOC_022225	Ca_v2.0_20878	Ca7_v2.0	14181485	14185758
XLOC_022227	Ca_v2.0_20881	Ca7_v2.0	14221923	14232683
XLOC_022229	Ca_v2.0_20885	Ca7_v2.0	14256400	14258858
XLOC_022236	Ca_v2.0_20895	Ca7_v2.0	14335259	14427177
XLOC_022240	Ca_v2.0_20899	Ca7_v2.0	14552656	14554971
XLOC_022242	Ca_v2.0_20901,Ca_v2.0_209(	Ca7_v2.0	14567615	14570467
XLOC_022243	Ca_v2.0_20903	Ca7_v2.0	14590882	14591734
XLOC_022250	Ca_v2.0_20916	Ca7_v2.0	14844291	14845507
XLOC_022262	Ca_v2.0_20936	Ca7_v2.0	15116973	15120887
XLOC_022268	Ca_v2.0_20947	Ca7_v2.0	15239130	15247216
XLOC_022273	Ca_v2.0_20957	Ca7_v2.0	15338523	15342559

For Review Only

XLOC_022275	Ca_v2.0_20960	Ca7_v2.0	15384056	15385179
XLOC_022286	Ca_v2.0_20973	Ca7_v2.0	15572018	15574680
XLOC_022287	Ca_v2.0_20974	Ca7_v2.0	15576129	15576465
XLOC_022296	Ca_v2.0_20991	Ca7_v2.0	15904688	15908085
XLOC_022299	Ca_v2.0_20998	Ca7_v2.0	15975808	15976941
XLOC_022307	Ca_v2.0_21012	Ca7_v2.0	16145849	16151586
XLOC_022311	Ca_v2.0_21017	Ca7_v2.0	16232301	16234186
XLOC_022312	Ca_v2.0_21020	Ca7_v2.0	16271142	16274061
XLOC_022324	Ca_v2.0_21040	Ca7_v2.0	16492554	16505188
XLOC_022336	Ca_v2.0_21082	Ca7_v2.0	17094916	17100150
XLOC_022344	Ca_v2.0_21098	Ca7_v2.0	17291789	17298872
XLOC_022347	Ca_v2.0_21106	Ca7_v2.0	17441143	17442282
XLOC_022352	Ca_v2.0_21112	Ca7_v2.0	17543065	17546764
XLOC_022356	Ca_v2.0_21125	Ca7_v2.0	17681725	17685014
XLOC_022358	Ca_v2.0_21128	Ca7_v2.0	17708745	17711204
XLOC_022359	Ca_v2.0_21130	Ca7_v2.0	17750510	17753830
XLOC_022360	Ca_v2.0_21132	Ca7_v2.0	17757140	17769564
XLOC_022372	Ca_v2.0_21157	Ca7_v2.0	18101428	18109948
XLOC_022375	Ca_v2.0_21162	Ca7_v2.0	18183015	18190219
XLOC_022376	Ca_v2.0_21164	Ca7_v2.0	18197208	18214061
XLOC_022387	Ca_v2.0_21187	Ca7_v2.0	18603774	18613326
XLOC_022394	Ca_v2.0_21201	Ca7_v2.0	18844679	18845739
XLOC_022400	Ca_v2.0_21208	Ca7_v2.0	19048424	19051585
XLOC_022403	Ca_v2.0_21215	Ca7_v2.0	19141685	19149141
XLOC_022405	Ca_v2.0_21217	Ca7_v2.0	19191951	19200321
XLOC_022412	Ca_v2.0_21231	Ca7_v2.0	19452252	19472459
XLOC_022419	Ca_v2.0_21247	Ca7_v2.0	20389487	20390482
XLOC_022429	Ca_v2.0_21268	Ca7_v2.0	20903563	20905019
XLOC_022431	Ca_v2.0_21276	Ca7_v2.0	21063122	21065241
XLOC_022442	Ca_v2.0_21300	Ca7_v2.0	21411009	21413507
XLOC_022446	Ca_v2.0_21309	Ca7_v2.0	21584477	21589618
XLOC_022450	Ca_v2.0_21314	Ca7_v2.0	21749949	21752732
XLOC_022468	Ca_v2.0_21351	Ca7_v2.0	22401328	22406573
XLOC_022469	Ca_v2.0_21354	Ca7_v2.0	22422323	22431468
XLOC_022480	Ca_v2.0_21375	Ca7_v2.0	22937821	22940053
XLOC_022483	Ca_v2.0_21378	Ca7_v2.0	23077154	23082060
XLOC_022485	-	Ca7_v2.0	23161816	23162596
XLOC_022495	Ca_v2.0_21399	Ca7_v2.0	23399883	23406553
XLOC_022498	Ca_v2.0_21403	Ca7_v2.0	23473534	23479763
XLOC_022507	Ca_v2.0_21422	Ca7_v2.0	23947045	23960824
XLOC_022528	-	Ca7_v2.0	24902982	24903720
XLOC_022544	-	Ca7_v2.0	25558086	25561814
XLOC_022556	Ca_v2.0_21503	Ca7_v2.0	26358624	26361710
XLOC_022557	Ca_v2.0_21504	Ca7_v2.0	26365430	26390292
XLOC_022560	Ca_v2.0_21507	Ca7_v2.0	26471549	26473892
XLOC_022565	Ca_v2.0_21513	Ca7_v2.0	26568281	26573850

For Review Only

XLOC_022572	Ca_v2.0_21520	Ca7_v2.0	26704401	26717818
XLOC_022575	Ca_v2.0_21528	Ca7_v2.0	27122679	27125189
XLOC_022576	Ca_v2.0_21530	Ca7_v2.0	27190379	27197925
XLOC_022589	Ca_v2.0_21546	Ca7_v2.0	28123422	28126745
XLOC_022591	Ca_v2.0_21549	Ca7_v2.0	28415525	28415992
XLOC_022592	Ca_v2.0_21550	Ca7_v2.0	28494566	28514496
XLOC_022593	Ca_v2.0_21552	Ca7_v2.0	28636241	28666851
XLOC_022604	-	Ca7_v2.0	30834525	30838652
XLOC_022607	-	Ca7_v2.0	30949888	30950790
XLOC_022613	Ca_v2.0_21606	Ca7_v2.0	31511912	31517322
XLOC_022615	Ca_v2.0_21612	Ca7_v2.0	31696275	31701132
XLOC_022620	Ca_v2.0_21624,Ca_v2.0_216	Ca7_v2.0	32243282	32262192
XLOC_022652	Ca_v2.0_21687	Ca7_v2.0	35316270	35325366
XLOC_022661	Ca_v2.0_21706,Ca_v2.0_217	Ca7_v2.0	36524305	36534859
XLOC_022677	-	Ca7_v2.0	40208085	40215314
XLOC_022689	Ca_v2.0_21750	Ca7_v2.0	41175344	41179909
XLOC_022690	Ca_v2.0_21751	Ca7_v2.0	41181603	41184005
XLOC_022695	Ca_v2.0_21760	Ca7_v2.0	41683951	41695298
XLOC_022705	-	Ca7_v2.0	42490917	42499665
XLOC_022706	-	Ca7_v2.0	42490917	42499665
XLOC_022712	Ca_v2.0_21790	Ca7_v2.0	43068158	43073988
XLOC_022714	Ca_v2.0_21794	Ca7_v2.0	43212043	43224676
XLOC_022716	-	Ca7_v2.0	43356537	43358864
XLOC_022722	Ca_v2.0_21806	Ca7_v2.0	44025461	44028052
XLOC_022723	Ca_v2.0_21807	Ca7_v2.0	44047379	44059880
XLOC_022725	Ca_v2.0_21811	Ca7_v2.0	44214115	44217228
XLOC_022732	Ca_v2.0_21830	Ca7_v2.0	44673922	44680174
XLOC_022743	Ca_v2.0_21849	Ca7_v2.0	45383782	45394157
XLOC_022745	Ca_v2.0_21855,Ca_v2.0_218	Ca7_v2.0	45530346	45647104
XLOC_022750	-	Ca7_v2.0	46186109	46200391
XLOC_022763	Ca_v2.0_21901	Ca7_v2.0	47024225	47025945
XLOC_022772	Ca_v2.0_21925	Ca7_v2.0	47771965	47772329
XLOC_022778	Ca_v2.0_21940	Ca7_v2.0	48269603	48271464
XLOC_022787	Ca_v2.0_21962	Ca7_v2.0	48844714	48848240
XLOC_022793	Ca_v2.0_21971	Ca7_v2.0	49011376	49024653
XLOC_022795	Ca_v2.0_21973	Ca7_v2.0	49045058	49046718
XLOC_022803	Ca_v2.0_21985	Ca7_v2.0	49277650	49286123
XLOC_022806	Ca_v2.0_21988	Ca7_v2.0	49318495	49319652
XLOC_022815	Ca_v2.0_22005	Ca7_v2.0	49765741	49769758
XLOC_022827	Ca_v2.0_22023	Ca7_v2.0	50271887	50276770
XLOC_022830	Ca_v2.0_22034	Ca7_v2.0	50362384	50437306
XLOC_022833	Ca_v2.0_22028	Ca7_v2.0	50362384	50437306
XLOC_022844	Ca_v2.0_22048	Ca7_v2.0	50730319	50731097
XLOC_022853	Ca_v2.0_22058	Ca7_v2.0	50958978	50961602
XLOC_022855	Ca_v2.0_22063	Ca7_v2.0	51109312	51127203
XLOC_022858	Ca_v2.0_22068	Ca7_v2.0	51293489	51294794

For Review Only

XLOC_022869	Ca_v2.0_22086	Ca7_v2.0	51615803	51617462
XLOC_022876	Ca_v2.0_22096	Ca7_v2.0	51826665	51832216
XLOC_022883	Ca_v2.0_22107	Ca7_v2.0	52019552	52020789
XLOC_022897	Ca_v2.0_22133	Ca7_v2.0	52487969	52493292
XLOC_022899	Ca_v2.0_22139	Ca7_v2.0	52576458	52581530
XLOC_022905	Ca_v2.0_22151	Ca7_v2.0	52703484	52705178
XLOC_022917	Ca_v2.0_22176	Ca7_v2.0	53261401	53272377
XLOC_022920	Ca_v2.0_22187	Ca7_v2.0	53408646	53412533
XLOC_022921	Ca_v2.0_22188	Ca7_v2.0	53426813	53429671
XLOC_022923	Ca_v2.0_22192	Ca7_v2.0	53533428	53536030
XLOC_022928	Ca_v2.0_22198	Ca7_v2.0	53575634	53577377
XLOC_022939	Ca_v2.0_22219	Ca7_v2.0	53789170	53792315
XLOC_022940	Ca_v2.0_22220	Ca7_v2.0	53812402	53816375
XLOC_022944	Ca_v2.0_22226	Ca7_v2.0	53829743	53831527
XLOC_022952	Ca_v2.0_22240	Ca7_v2.0	53936288	53949379
XLOC_022953	Ca_v2.0_22241	Ca7_v2.0	53936288	53949379
XLOC_022954	Ca_v2.0_22246	Ca7_v2.0	54030843	54034472
XLOC_022957	Ca_v2.0_22252,Ca_v2.0_222!	Ca7_v2.0	54130767	54168806
XLOC_022960	Ca_v2.0_22256	Ca7_v2.0	54217714	54218272
XLOC_022967	Ca_v2.0_22269	Ca7_v2.0	54500688	54505575
XLOC_022980	Ca_v2.0_22299	Ca7_v2.0	54964721	54967665
XLOC_022983	Ca_v2.0_22304	Ca7_v2.0	55010866	55012405
XLOC_022990	Ca_v2.0_22315	Ca7_v2.0	55338513	55340818
XLOC_022991	Ca_v2.0_22317	Ca7_v2.0	55374035	55384958
XLOC_022992	Ca_v2.0_22318	Ca7_v2.0	55411823	55416817
XLOC_023002	Ca_v2.0_22335	Ca7_v2.0	55821195	55825468
XLOC_023007	Ca_v2.0_22345	Ca7_v2.0	55956864	55959783
XLOC_023009	Ca_v2.0_22350	Ca7_v2.0	55996960	56000164
XLOC_023012	Ca_v2.0_22355	Ca7_v2.0	56031141	56037670
XLOC_023015	Ca_v2.0_22361	Ca7_v2.0	56141243	56142814
XLOC_023017	Ca_v2.0_22365	Ca7_v2.0	56184283	56190142
XLOC_023018	Ca_v2.0_22366	Ca7_v2.0	56194922	56198665
XLOC_023021	Ca_v2.0_22370	Ca7_v2.0	56237803	56239061
XLOC_023022	Ca_v2.0_22372	Ca7_v2.0	56266377	56268052
XLOC_023027	Ca_v2.0_22378	Ca7_v2.0	56326063	56327341
XLOC_023030	Ca_v2.0_22381	Ca7_v2.0	56399207	56411068
XLOC_023034	Ca_v2.0_22387	Ca7_v2.0	56494744	56495776
XLOC_023036	Ca_v2.0_22390	Ca7_v2.0	56520370	56524910
XLOC_023037	Ca_v2.0_22392	Ca7_v2.0	56547414	56549273
XLOC_023038	Ca_v2.0_22393	Ca7_v2.0	56551402	56553027
XLOC_023055	Ca_v2.0_22421	Ca7_v2.0	56972357	56973063
XLOC_023056	Ca_v2.0_22422	Ca7_v2.0	57000306	57009679
XLOC_023057	Ca_v2.0_22423	Ca7_v2.0	57025748	57029517
XLOC_023059	Ca_v2.0_22427	Ca7_v2.0	57084501	57088891
XLOC_023066	Ca_v2.0_22448,Ca_v2.0_224	Ca7_v2.0	57312053	57320495
XLOC_023067	Ca_v2.0_22450	Ca7_v2.0	57361355	57434033

For Review Only

XLOC_023069	Ca_v2.0_22455,Ca_v2.0_224!	Ca7_v2.0	57525030	57545716
XLOC_023072	Ca_v2.0_22459	Ca7_v2.0	57565711	57567542
XLOC_023079	Ca_v2.0_22475	Ca7_v2.0	57789546	57799804
XLOC_023089	Ca_v2.0_22488	Ca7_v2.0	57988854	57992492
XLOC_023096	Ca_v2.0_22500	Ca7_v2.0	58137662	58141015
XLOC_023109	Ca_v2.0_22527	Ca7_v2.0	58441820	58443447
XLOC_023111	Ca_v2.0_22530	Ca7_v2.0	58491335	58493959
XLOC_023117	Ca_v2.0_22541	Ca7_v2.0	58599871	58600282
XLOC_023120	Ca_v2.0_22547	Ca7_v2.0	58667061	58669297
XLOC_023128	Ca_v2.0_22557	Ca7_v2.0	58746555	58749612
XLOC_023131	Ca_v2.0_22560	Ca7_v2.0	58819550	58822677
XLOC_023137	Ca_v2.0_22579	Ca7_v2.0	59297516	59299202
XLOC_023139	Ca_v2.0_22581	Ca7_v2.0	59327962	59329991
XLOC_023143	Ca_v2.0_22590	Ca7_v2.0	59475293	59477222
XLOC_023145	Ca_v2.0_22592	Ca7_v2.0	59493227	59494339
XLOC_023148	Ca_v2.0_22595	Ca7_v2.0	59555847	59559025
XLOC_023159	Ca_v2.0_22608	Ca7_v2.0	59686839	59692220
XLOC_023164	Ca_v2.0_22613	Ca7_v2.0	59735905	59743486
XLOC_023165	Ca_v2.0_22615	Ca7_v2.0	59746813	59747439
XLOC_023167	Ca_v2.0_22616	Ca7_v2.0	59749948	59752837
XLOC_023172	Ca_v2.0_22633	Ca7_v2.0	59915503	59920757
XLOC_023174	Ca_v2.0_22637	Ca7_v2.0	59939136	59939520
XLOC_023175	Ca_v2.0_22638	Ca7_v2.0	59944827	59949260
XLOC_023176	Ca_v2.0_22639	Ca7_v2.0	59954934	59957572
XLOC_023177	Ca_v2.0_22642	Ca7_v2.0	60000907	60002477
XLOC_023184	Ca_v2.0_22653	Ca7_v2.0	60115859	60117524
XLOC_023202	Ca_v2.0_22676	Ca7_v2.0	60317205	60321815
XLOC_023205	-	Ca7_v2.0	60472956	60474000
XLOC_023207	Ca_v2.0_22685	Ca7_v2.0	60489929	60491365
XLOC_023208	-	Ca7_v2.0	60496043	60497024
XLOC_023219	Ca_v2.0_22702	Ca7_v2.0	60794802	60800388
XLOC_023224	Ca_v2.0_22713	Ca7_v2.0	60878814	60891405
XLOC_023227	Ca_v2.0_22717	Ca7_v2.0	60967161	60976102
XLOC_023230	Ca_v2.0_22720	Ca7_v2.0	61022295	61026240
XLOC_023232	Ca_v2.0_22724	Ca7_v2.0	61061646	61064711
XLOC_023241	Ca_v2.0_22739	Ca7_v2.0	61321788	61322994
XLOC_023243	Ca_v2.0_22743	Ca7_v2.0	61402271	61432783
XLOC_023266	Ca_v2.0_22788	Ca7_v2.0	61970491	61971290
XLOC_023267	Ca_v2.0_22789	Ca7_v2.0	61972390	61976163
XLOC_023270	Ca_v2.0_22794	Ca7_v2.0	62037455	62043252
XLOC_023271	Ca_v2.0_22797	Ca7_v2.0	62052127	62053843
XLOC_023275	Ca_v2.0_22802	Ca7_v2.0	62086936	62088844
XLOC_023277	-	Ca7_v2.0	62107885	62108990
XLOC_023278	Ca_v2.0_22803	Ca7_v2.0	62111097	62111773
XLOC_023281	Ca_v2.0_22807	Ca7_v2.0	62139196	62143034
XLOC_023289	Ca_v2.0_22818	Ca7_v2.0	62211545	62223819

For Review Only

XLOC_023295	Ca_v2.0_22835	Ca7_v2.0	62402152	62409611
XLOC_023304	Ca_v2.0_19419	Ca7_v2.0	215299	216375
XLOC_023307	Ca_v2.0_19426	Ca7_v2.0	288662	289512
XLOC_023311	Ca_v2.0_19432	Ca7_v2.0	353628	360670
XLOC_023322	Ca_v2.0_19449	Ca7_v2.0	516314	518928
XLOC_023323	Ca_v2.0_19450	Ca7_v2.0	520413	521964
XLOC_023328	Ca_v2.0_19457	Ca7_v2.0	578167	578975
XLOC_023330	Ca_v2.0_19461	Ca7_v2.0	594144	599046
XLOC_023342	Ca_v2.0_19483	Ca7_v2.0	775145	783457
XLOC_023352	Ca_v2.0_19501	Ca7_v2.0	895875	896929
XLOC_023353	Ca_v2.0_19502	Ca7_v2.0	898516	901404
XLOC_023355	Ca_v2.0_19506	Ca7_v2.0	956421	962647
XLOC_023361	Ca_v2.0_19520	Ca7_v2.0	1074595	1079344
XLOC_023378	Ca_v2.0_19543	Ca7_v2.0	1249195	1255618
XLOC_023392	Ca_v2.0_19569	Ca7_v2.0	1433720	1443330
XLOC_023403	Ca_v2.0_19589	Ca7_v2.0	1604767	1611722
XLOC_023407	Ca_v2.0_19597	Ca7_v2.0	1674605	1678842
XLOC_023414	Ca_v2.0_19610	Ca7_v2.0	1776829	1779201
XLOC_023420	Ca_v2.0_19632	Ca7_v2.0	2046682	2048416
XLOC_023425	Ca_v2.0_19639	Ca7_v2.0	2097716	2098593
XLOC_023429	Ca_v2.0_19646	Ca7_v2.0	2156624	2165520
XLOC_023430	Ca_v2.0_19647	Ca7_v2.0	2167571	2175151
XLOC_023432	Ca_v2.0_19653	Ca7_v2.0	2218522	2220401
XLOC_023433	Ca_v2.0_19656	Ca7_v2.0	2249000	2250779
XLOC_023434	Ca_v2.0_19660	Ca7_v2.0	2292985	2294314
XLOC_023437	Ca_v2.0_19665	Ca7_v2.0	2345124	2350961
XLOC_023442	Ca_v2.0_19680	Ca7_v2.0	2524069	2525591
XLOC_023451	Ca_v2.0_19695	Ca7_v2.0	2640574	2644204
XLOC_023453	Ca_v2.0_19700	Ca7_v2.0	2695766	2699899
XLOC_023454	Ca_v2.0_19701	Ca7_v2.0	2705699	2708026
XLOC_023458	Ca_v2.0_19708	Ca7_v2.0	2757893	2760958
XLOC_023459	Ca_v2.0_19710	Ca7_v2.0	2787750	2791724
XLOC_023461	Ca_v2.0_19713	Ca7_v2.0	2811568	2819389
XLOC_023469	Ca_v2.0_19724	Ca7_v2.0	2908717	2914729
XLOC_023474	Ca_v2.0_19732	Ca7_v2.0	2967292	2979208
XLOC_023475	Ca_v2.0_19737	Ca7_v2.0	3008526	3010500
XLOC_023479	Ca_v2.0_19741	Ca7_v2.0	3039891	3042675
XLOC_023480	Ca_v2.0_19744	Ca7_v2.0	3054533	3056860
XLOC_023485	Ca_v2.0_19757	Ca7_v2.0	3157350	3160210
XLOC_023497	Ca_v2.0_19777	Ca7_v2.0	3282139	3285705
XLOC_023503	Ca_v2.0_19790	Ca7_v2.0	3396640	3398156
XLOC_023504	Ca_v2.0_19791	Ca7_v2.0	3403278	3405402
XLOC_023508	Ca_v2.0_19797	Ca7_v2.0	3459357	3460097
XLOC_023511	Ca_v2.0_19803	Ca7_v2.0	3508925	3510115
XLOC_023519	Ca_v2.0_19817	Ca7_v2.0	3655452	3657948
XLOC_023520	Ca_v2.0_19818	Ca7_v2.0	3660585	3663745

For Review Only

XLOC_023525	Ca_v2.0_19825	Ca7_v2.0	3746294	3748659
XLOC_023529	Ca_v2.0_19834	Ca7_v2.0	3810160	3816326
XLOC_023540	Ca_v2.0_19851	Ca7_v2.0	3932891	3942947
XLOC_023541	Ca_v2.0_19853	Ca7_v2.0	3948966	3951866
XLOC_023542	Ca_v2.0_19855	Ca7_v2.0	3966551	3969738
XLOC_023544	Ca_v2.0_19858,Ca_v2.0_198!	Ca7_v2.0	3988027	3990539
XLOC_023548	Ca_v2.0_19875	Ca7_v2.0	4205818	4209835
XLOC_023553	Ca_v2.0_19881	Ca7_v2.0	4252487	4279355
XLOC_023555	Ca_v2.0_19889	Ca7_v2.0	4295554	4298397
XLOC_023560	Ca_v2.0_19898,Ca_v2.0_198!	Ca7_v2.0	4376911	4383998
XLOC_023562	Ca_v2.0_19902	Ca7_v2.0	4407250	4408225
XLOC_023567	Ca_v2.0_19909	Ca7_v2.0	4464028	4465215
XLOC_023579	Ca_v2.0_19934	Ca7_v2.0	4714806	4715845
XLOC_023580	Ca_v2.0_19935	Ca7_v2.0	4717902	4718854
XLOC_023585	Ca_v2.0_19944	Ca7_v2.0	4778116	4784873
XLOC_023588	Ca_v2.0_19948	Ca7_v2.0	4803511	4806805
XLOC_023591	Ca_v2.0_19951	Ca7_v2.0	4816498	4817085
XLOC_023592	Ca_v2.0_19953	Ca7_v2.0	4828217	4829036
XLOC_023596	Ca_v2.0_19961	Ca7_v2.0	4889149	4890651
XLOC_023609	Ca_v2.0_19981	Ca7_v2.0	5047228	5050911
XLOC_023611	Ca_v2.0_19983	Ca7_v2.0	5061212	5062114
XLOC_023617	Ca_v2.0_19999	Ca7_v2.0	5217001	5222575
XLOC_023623	Ca_v2.0_20008	Ca7_v2.0	5286733	5289182
XLOC_023625	Ca_v2.0_20016	Ca7_v2.0	5347778	5352686
XLOC_023627	Ca_v2.0_20018	Ca7_v2.0	5362321	5366464
XLOC_023636	Ca_v2.0_20032	Ca7_v2.0	5472838	5474557
XLOC_023640	Ca_v2.0_20036	Ca7_v2.0	5485000	5495898
XLOC_023642	Ca_v2.0_20042	Ca7_v2.0	5529368	5535173
XLOC_023653	Ca_v2.0_20066	Ca7_v2.0	5773955	5795781
XLOC_023658	Ca_v2.0_20074	Ca7_v2.0	5884518	5885510
XLOC_023660	Ca_v2.0_20076	Ca7_v2.0	5900529	5901217
XLOC_023670	Ca_v2.0_20093	Ca7_v2.0	6045896	6058711
XLOC_023682	Ca_v2.0_20125	Ca7_v2.0	6308484	6315001
XLOC_023688	Ca_v2.0_20133	Ca7_v2.0	6385710	6390286
XLOC_023689	-	Ca7_v2.0	6410411	6411862
XLOC_023690	-	Ca7_v2.0	6456911	6459302
XLOC_023693	Ca_v2.0_20144	Ca7_v2.0	6590247	6591994
XLOC_023694	Ca_v2.0_20145	Ca7_v2.0	6595491	6597124
XLOC_023703	Ca_v2.0_20159	Ca7_v2.0	6736344	6737420
XLOC_023709	Ca_v2.0_20174	Ca7_v2.0	6826277	6831155
XLOC_023715	Ca_v2.0_20182	Ca7_v2.0	6899524	6902600
XLOC_023720	Ca_v2.0_20189	Ca7_v2.0	6937395	6940971
XLOC_023723	Ca_v2.0_20195	Ca7_v2.0	6983672	6985266
XLOC_023724	Ca_v2.0_20197	Ca7_v2.0	7016426	7020672
XLOC_023732	Ca_v2.0_20210	Ca7_v2.0	7137370	7138941
XLOC_023747	Ca_v2.0_20237	Ca7_v2.0	7426163	7427653

For Review Only

XLOC_023748	Ca_v2.0_20238	Ca7_v2.0	7428577	7433174
XLOC_023750	Ca_v2.0_20242	Ca7_v2.0	7471997	7472824
XLOC_023751	Ca_v2.0_20244	Ca7_v2.0	7488194	7488930
XLOC_023780	Ca_v2.0_20301	Ca7_v2.0	7981671	7985796
XLOC_023787	Ca_v2.0_20316	Ca7_v2.0	8101373	8105162
XLOC_023792	Ca_v2.0_20328	Ca7_v2.0	8199180	8201394
XLOC_023795	Ca_v2.0_20332	Ca7_v2.0	8214625	8220149
XLOC_023801	Ca_v2.0_20340	Ca7_v2.0	8286663	8288715
XLOC_023820	Ca_v2.0_20370	Ca7_v2.0	8519791	8524266
XLOC_023822	Ca_v2.0_20375	Ca7_v2.0	8560282	8568317
XLOC_023828	Ca_v2.0_20389	Ca7_v2.0	8704790	8718230
XLOC_023835	Ca_v2.0_20404	Ca7_v2.0	8873152	8878227
XLOC_023838	Ca_v2.0_20409	Ca7_v2.0	8925876	8928666
XLOC_023840	Ca_v2.0_20413	Ca7_v2.0	8952115	8953671
XLOC_023844	-	Ca7_v2.0	9007860	9008886
XLOC_023854	Ca_v2.0_20433	Ca7_v2.0	9183320	9184915
XLOC_023857	Ca_v2.0_20442	Ca7_v2.0	9242905	9244120
XLOC_023862	Ca_v2.0_20459	Ca7_v2.0	9358548	9362043
XLOC_023866	Ca_v2.0_20471	Ca7_v2.0	9464956	9468493
XLOC_023868	Ca_v2.0_20473	Ca7_v2.0	9480308	9486606
XLOC_023872	Ca_v2.0_20481	Ca7_v2.0	9591435	9594809
XLOC_023876	Ca_v2.0_20490	Ca7_v2.0	9697365	9700199
XLOC_023882	Ca_v2.0_20502	Ca7_v2.0	9760385	9762555
XLOC_023887	Ca_v2.0_20510	Ca7_v2.0	9831015	9833626
XLOC_023888	-	Ca7_v2.0	9840858	9841554
XLOC_023891	-	Ca7_v2.0	9884181	9887060
XLOC_023895	Ca_v2.0_20521	Ca7_v2.0	9936918	9940588
XLOC_023896	Ca_v2.0_20522	Ca7_v2.0	9979503	9981924
XLOC_023898	Ca_v2.0_20534	Ca7_v2.0	10079872	10085012
XLOC_023901	Ca_v2.0_20540	Ca7_v2.0	10152313	10157213
XLOC_023903	Ca_v2.0_20543	Ca7_v2.0	10175175	10185597
XLOC_023922	Ca_v2.0_20585	Ca7_v2.0	10608958	10611500
XLOC_023933	Ca_v2.0_20610	Ca7_v2.0	10868281	10869993
XLOC_023934	Ca_v2.0_20612	Ca7_v2.0	10878840	10883326
XLOC_023935	Ca_v2.0_20613	Ca7_v2.0	10883977	10886900
XLOC_023936	Ca_v2.0_20616	Ca7_v2.0	10920973	10928529
XLOC_023937	-	Ca7_v2.0	10934531	10935950
XLOC_023938	Ca_v2.0_20618	Ca7_v2.0	10962321	10967282
XLOC_023941	Ca_v2.0_20621	Ca7_v2.0	11012108	11015733
XLOC_023944	Ca_v2.0_20626	Ca7_v2.0	11089587	11097129
XLOC_023945	Ca_v2.0_20628	Ca7_v2.0	11123045	11123707
XLOC_023947	Ca_v2.0_20631	Ca7_v2.0	11147159	11152700
XLOC_023949	Ca_v2.0_20633	Ca7_v2.0	11161915	11162630
XLOC_023957	Ca_v2.0_20650	Ca7_v2.0	11300257	11311987
XLOC_023965	Ca_v2.0_20661	Ca7_v2.0	11418406	11420184
XLOC_023966	Ca_v2.0_20662	Ca7_v2.0	11422242	11427443

For Review Only

XLOC_023972	Ca_v2.0_20669	Ca7_v2.0	11526583	11528834
XLOC_023973	Ca_v2.0_20670	Ca7_v2.0	11532718	11534285
XLOC_023979	Ca_v2.0_20679	Ca7_v2.0	11635414	11637216
XLOC_023987	Ca_v2.0_20700	Ca7_v2.0	11864341	11884340
XLOC_023989	Ca_v2.0_20706	Ca7_v2.0	11916820	11921441
XLOC_023991	Ca_v2.0_20719	Ca7_v2.0	12095231	12097399
XLOC_023993	Ca_v2.0_20721,Ca_v2.0_207:	Ca7_v2.0	12110748	12116701
XLOC_023996	Ca_v2.0_20729	Ca7_v2.0	12212004	12217737
XLOC_024002	Ca_v2.0_20745	Ca7_v2.0	12383966	12385651
XLOC_024004	Ca_v2.0_20750	Ca7_v2.0	12491346	12495671
XLOC_024010	Ca_v2.0_20761	Ca7_v2.0	12662972	12666755
XLOC_024011	Ca_v2.0_20762	Ca7_v2.0	12670526	12671534
XLOC_024013	Ca_v2.0_20765	Ca7_v2.0	12701875	12703706
XLOC_024015	Ca_v2.0_20767	Ca7_v2.0	12723308	12724204
XLOC_024016	Ca_v2.0_20770	Ca7_v2.0	12772344	12774044
XLOC_024024	Ca_v2.0_20782	Ca7_v2.0	12874073	12875940
XLOC_024031	Ca_v2.0_20795	Ca7_v2.0	13062676	13066523
XLOC_024034	Ca_v2.0_20802	Ca7_v2.0	13118107	13121963
XLOC_024035	Ca_v2.0_20803	Ca7_v2.0	13129812	13132496
XLOC_024044	Ca_v2.0_20825	Ca7_v2.0	13422824	13424625
XLOC_024045	Ca_v2.0_20827	Ca7_v2.0	13449488	13460643
XLOC_024049	Ca_v2.0_20840	Ca7_v2.0	13644156	13645214
XLOC_024055	Ca_v2.0_20854	Ca7_v2.0	13846080	13857793
XLOC_024056	Ca_v2.0_20862	Ca7_v2.0	13991210	14010266
XLOC_024059	-	Ca7_v2.0	14060810	14069321
XLOC_024065	Ca_v2.0_20876	Ca7_v2.0	14158119	14164373
XLOC_024066	Ca_v2.0_20879	Ca7_v2.0	14189999	14191825
XLOC_024068	Ca_v2.0_20885	Ca7_v2.0	14256400	14258858
XLOC_024074	Ca_v2.0_20893	Ca7_v2.0	14335259	14427177
XLOC_024075	Ca_v2.0_20895	Ca7_v2.0	14335259	14427177
XLOC_024083	Ca_v2.0_20914	Ca7_v2.0	14782775	14785059
XLOC_024084	Ca_v2.0_20917	Ca7_v2.0	14857370	14864091
XLOC_024091	Ca_v2.0_20938	Ca7_v2.0	15138671	15139615
XLOC_024103	Ca_v2.0_20967	Ca7_v2.0	15505424	15515711
XLOC_024105	Ca_v2.0_20978	Ca7_v2.0	15597599	15600487
XLOC_024108	Ca_v2.0_20983	Ca7_v2.0	15721104	15727438
XLOC_024112	Ca_v2.0_20991	Ca7_v2.0	15904688	15908085
XLOC_024118	Ca_v2.0_21001	Ca7_v2.0	16001628	16009809
XLOC_024123	Ca_v2.0_21013	Ca7_v2.0	16153395	16156053
XLOC_024124	Ca_v2.0_21014	Ca7_v2.0	16160526	16163112
XLOC_024138	Ca_v2.0_21045	Ca7_v2.0	16526623	16535259
XLOC_024147	Ca_v2.0_21059	Ca7_v2.0	16686321	16689507
XLOC_024148	Ca_v2.0_21060	Ca7_v2.0	16725129	16726267
XLOC_024149	Ca_v2.0_21061	Ca7_v2.0	16736569	16737513
XLOC_024150	Ca_v2.0_21062	Ca7_v2.0	16751240	16752366
XLOC_024152	Ca_v2.0_21064	Ca7_v2.0	16774911	16778552

For Review Only

XLOC_024157	Ca_v2.0_21071	Ca7_v2.0	16945256	16948114
XLOC_024164	Ca_v2.0_21080	Ca7_v2.0	17084125	17084980
XLOC_024165	Ca_v2.0_21081	Ca7_v2.0	17089968	17092721
XLOC_024177	Ca_v2.0_21103	Ca7_v2.0	17372319	17379638
XLOC_024185	Ca_v2.0_21118	Ca7_v2.0	17593182	17603397
XLOC_024204	Ca_v2.0_21145	Ca7_v2.0	17988281	17993913
XLOC_024214	Ca_v2.0_21167	Ca7_v2.0	18237725	18251375
XLOC_024227	Ca_v2.0_21187	Ca7_v2.0	18603774	18613326
XLOC_024237	Ca_v2.0_21211	Ca7_v2.0	19071154	19072517
XLOC_024248	Ca_v2.0_21230	Ca7_v2.0	19423209	19427429
XLOC_024251	Ca_v2.0_21232	Ca7_v2.0	19474541	19475633
XLOC_024257	Ca_v2.0_21242,Ca_v2.0_212	Ca7_v2.0	20300306	20303721
XLOC_024261	Ca_v2.0_21249	Ca7_v2.0	20439988	20447910
XLOC_024272	Ca_v2.0_21267	Ca7_v2.0	20864223	20866521
XLOC_024296	Ca_v2.0_21308	Ca7_v2.0	21537904	21540503
XLOC_024298	Ca_v2.0_21313	Ca7_v2.0	21685846	21687218
XLOC_024316	Ca_v2.0_21339	Ca7_v2.0	22171359	22180039
XLOC_024353	Ca_v2.0_21418	Ca7_v2.0	23905694	23907198
XLOC_024368	Ca_v2.0_21450	Ca7_v2.0	24601457	24601718
XLOC_024370	Ca_v2.0_21452	Ca7_v2.0	24703535	24707348
XLOC_024375	Ca_v2.0_21463	Ca7_v2.0	24980191	24990729
XLOC_024378	Ca_v2.0_21467	Ca7_v2.0	25049238	25060483
XLOC_024381	Ca_v2.0_21468	Ca7_v2.0	25095661	25096525
XLOC_024391	Ca_v2.0_21486	Ca7_v2.0	25550104	25555462
XLOC_024400	Ca_v2.0_21510	Ca7_v2.0	26514119	26521672
XLOC_024406	Ca_v2.0_21526	Ca7_v2.0	27058470	27062535
XLOC_024408	Ca_v2.0_21530	Ca7_v2.0	27190379	27197925
XLOC_024418	Ca_v2.0_21561	Ca7_v2.0	29203680	29205050
XLOC_024421	Ca_v2.0_21566	Ca7_v2.0	29673794	29675840
XLOC_024443	Ca_v2.0_21590	Ca7_v2.0	30545734	30547891
XLOC_024447	Ca_v2.0_21597	Ca7_v2.0	31137714	31142556
XLOC_024453	Ca_v2.0_21605	Ca7_v2.0	31511912	31517322
XLOC_024460	Ca_v2.0_21614	Ca7_v2.0	31759564	31761671
XLOC_024461	-	Ca7_v2.0	31783554	31784274
XLOC_024465	Ca_v2.0_21621	Ca7_v2.0	32054047	32056894
XLOC_024466	Ca_v2.0_21622	Ca7_v2.0	32209619	32214181
XLOC_024475	Ca_v2.0_21638	Ca7_v2.0	32668472	32671760
XLOC_024481	Ca_v2.0_21651	Ca7_v2.0	33657607	33661004
XLOC_024506	Ca_v2.0_21709	Ca7_v2.0	36644130	36649970
XLOC_024508	-	Ca7_v2.0	36821098	36824581
XLOC_024511	Ca_v2.0_21724	Ca7_v2.0	38100845	38103277
XLOC_024519	Ca_v2.0_21741	Ca7_v2.0	40781417	40783937
XLOC_024529	Ca_v2.0_21767	Ca7_v2.0	42019246	42023211
XLOC_024540	Ca_v2.0_21784	Ca7_v2.0	42714497	42716491
XLOC_024555	Ca_v2.0_21808	Ca7_v2.0	44172270	44178638
XLOC_024558	Ca_v2.0_21811	Ca7_v2.0	44214115	44217228

For Review Only

XLOC_024563	Ca_v2.0_21820	Ca7_v2.0	44429958	44440917
XLOC_024567	Ca_v2.0_21826	Ca7_v2.0	44576668	44579878
XLOC_024572	Ca_v2.0_21835	Ca7_v2.0	44823229	44828261
XLOC_024574	Ca_v2.0_21837	Ca7_v2.0	44889401	44894213
XLOC_024576	Ca_v2.0_21841	Ca7_v2.0	44974171	44974830
XLOC_024583	Ca_v2.0_21859	Ca7_v2.0	45751113	45752613
XLOC_024587	Ca_v2.0_21865,Ca_v2.0_218(	Ca7_v2.0	46126274	46131695
XLOC_024595	Ca_v2.0_21874	Ca7_v2.0	46361048	46367750
XLOC_024596	Ca_v2.0_21875	Ca7_v2.0	46391447	46398065
XLOC_024599	Ca_v2.0_21879	Ca7_v2.0	46436086	46441605
XLOC_024606	Ca_v2.0_21890	Ca7_v2.0	46757683	46761381
XLOC_024616	Ca_v2.0_21905,Ca_v2.0_219(	Ca7_v2.0	47091578	47096198
XLOC_024625	Ca_v2.0_21921	Ca7_v2.0	47650629	47651255
XLOC_024628	Ca_v2.0_21924	Ca7_v2.0	47735483	47741040
XLOC_024629	Ca_v2.0_21926	Ca7_v2.0	47817569	47819074
XLOC_024637	Ca_v2.0_21941,Ca_v2.0_219	Ca7_v2.0	48271729	48287976
XLOC_024640	Ca_v2.0_21950	Ca7_v2.0	48404908	48409623
XLOC_024649	Ca_v2.0_21964	Ca7_v2.0	48867371	48873230
XLOC_024658	Ca_v2.0_21990	Ca7_v2.0	49351129	49363361
XLOC_024662	Ca_v2.0_21997	Ca7_v2.0	49588005	49590086
XLOC_024664	Ca_v2.0_22002	Ca7_v2.0	49671150	49672797
XLOC_024671	Ca_v2.0_22022	Ca7_v2.0	50229516	50234506
XLOC_024672	Ca_v2.0_22030	Ca7_v2.0	50362384	50437306
XLOC_024683	Ca_v2.0_22063	Ca7_v2.0	51109312	51127203
XLOC_024686	Ca_v2.0_22068	Ca7_v2.0	51293489	51294794
XLOC_024688	Ca_v2.0_22071	Ca7_v2.0	51354489	51356142
XLOC_024695	Ca_v2.0_22092	Ca7_v2.0	51728054	51744069
XLOC_024696	Ca_v2.0_22097	Ca7_v2.0	51834445	51835386
XLOC_024697	Ca_v2.0_22098	Ca7_v2.0	51904975	51905957
XLOC_024721	Ca_v2.0_22149	Ca7_v2.0	52674609	52685005
XLOC_024722	Ca_v2.0_22154	Ca7_v2.0	52726996	52729351
XLOC_024738	Ca_v2.0_22179	Ca7_v2.0	53327214	53336351
XLOC_024740	Ca_v2.0_22183	Ca7_v2.0	53359974	53365303
XLOC_024753	Ca_v2.0_22211	Ca7_v2.0	53693831	53697737
XLOC_024754	Ca_v2.0_22212	Ca7_v2.0	53701116	53709156
XLOC_024773	Ca_v2.0_22260	Ca7_v2.0	54289558	54292121
XLOC_024774	Ca_v2.0_22261	Ca7_v2.0	54329570	54335564
XLOC_024779	Ca_v2.0_22269	Ca7_v2.0	54500688	54505575
XLOC_024780	Ca_v2.0_22271	Ca7_v2.0	54509376	54517594
XLOC_024787	Ca_v2.0_22280	Ca7_v2.0	54673956	54676922
XLOC_024792	Ca_v2.0_22292	Ca7_v2.0	54863774	54865091
XLOC_024803	Ca_v2.0_22310	Ca7_v2.0	55126046	55128377
XLOC_024806	Ca_v2.0_22316	Ca7_v2.0	55370541	55371708
XLOC_024808	-	Ca7_v2.0	55396249	55397405
XLOC_024815	Ca_v2.0_22326	Ca7_v2.0	55641059	55641879
XLOC_024817	Ca_v2.0_22335	Ca7_v2.0	55821195	55825468

For Review Only

XLOC_024821	Ca_v2.0_22342	Ca7_v2.0	55932564	55936873
XLOC_024822	Ca_v2.0_22343	Ca7_v2.0	55941106	55944890
XLOC_024829	Ca_v2.0_22355	Ca7_v2.0	56031141	56037670
XLOC_024831	Ca_v2.0_22357	Ca7_v2.0	56044972	56047995
XLOC_024834	Ca_v2.0_22362	Ca7_v2.0	56145699	56147700
XLOC_024836	Ca_v2.0_22365	Ca7_v2.0	56184283	56190142
XLOC_024842	Ca_v2.0_22380	Ca7_v2.0	56389867	56393251
XLOC_024844	Ca_v2.0_22383	Ca7_v2.0	56445532	56457829
XLOC_024845	Ca_v2.0_22385	Ca7_v2.0	56459438	56468032
XLOC_024852	Ca_v2.0_22404	Ca7_v2.0	56732938	56736062
XLOC_024853	Ca_v2.0_22405,Ca_v2.0_224(	Ca7_v2.0	56745384	56753021
XLOC_024861	Ca_v2.0_22424	Ca7_v2.0	57042092	57044862
XLOC_024862	Ca_v2.0_22426	Ca7_v2.0	57067861	57074256
XLOC_024868	Ca_v2.0_22435	Ca7_v2.0	57147253	57148056
XLOC_024879	-	Ca7_v2.0	57358609	57359781
XLOC_024882	-	Ca7_v2.0	57470995	57471856
XLOC_024887	Ca_v2.0_22459	Ca7_v2.0	57565711	57567542
XLOC_024890	Ca_v2.0_22461	Ca7_v2.0	57596077	57601860
XLOC_024892	Ca_v2.0_22464	Ca7_v2.0	57627475	57634468
XLOC_024894	Ca_v2.0_22469	Ca7_v2.0	57697303	57698304
XLOC_024895	Ca_v2.0_22470	Ca7_v2.0	57708201	57708998
XLOC_024898	Ca_v2.0_22476	Ca7_v2.0	57789546	57799804
XLOC_024900	Ca_v2.0_22479	Ca7_v2.0	57874877	57907029
XLOC_024903	Ca_v2.0_22489	Ca7_v2.0	58019071	58027307
XLOC_024907	Ca_v2.0_22497	Ca7_v2.0	58099200	58106806
XLOC_024910	Ca_v2.0_22506	Ca7_v2.0	58187582	58190307
XLOC_024924	Ca_v2.0_22528	Ca7_v2.0	58445041	58447385
XLOC_024932	Ca_v2.0_22551	Ca7_v2.0	58690267	58693467
XLOC_024935	Ca_v2.0_22559	Ca7_v2.0	58805659	58817550
XLOC_024944	Ca_v2.0_22571	Ca7_v2.0	59113771	59134901
XLOC_024946	Ca_v2.0_22574	Ca7_v2.0	59194392	59195426
XLOC_024955	-	Ca7_v2.0	59508848	59510536
XLOC_024960	Ca_v2.0_22614	Ca7_v2.0	59735905	59743486
XLOC_024970	Ca_v2.0_22628	Ca7_v2.0	59848199	59869383
XLOC_024972	Ca_v2.0_22631	Ca7_v2.0	59883677	59885942
XLOC_024973	Ca_v2.0_22634	Ca7_v2.0	59920998	59924456
XLOC_024976	Ca_v2.0_22641	Ca7_v2.0	59975233	59979267
XLOC_024990	Ca_v2.0_22677	Ca7_v2.0	60331859	60332539
XLOC_024997	Ca_v2.0_22686	Ca7_v2.0	60501121	60506317
XLOC_024998	Ca_v2.0_22687	Ca7_v2.0	60510961	60520562
XLOC_025004	Ca_v2.0_22698	Ca7_v2.0	60759019	60762890
XLOC_025010	Ca_v2.0_22707	Ca7_v2.0	60846282	60850553
XLOC_025013	Ca_v2.0_22712	Ca7_v2.0	60875971	60878079
XLOC_025016	Ca_v2.0_22718	Ca7_v2.0	60976475	60982954
XLOC_025018	Ca_v2.0_22721	Ca7_v2.0	61045280	61045885
XLOC_025029	-	Ca7_v2.0	61470089	61473432

For Review Only

XLOC_025033	Ca_v2.0_22758	Ca7_v2.0	61602515	61604561
XLOC_025038	Ca_v2.0_22769	Ca7_v2.0	61707307	61711850
XLOC_025041	Ca_v2.0_22776	Ca7_v2.0	61827871	61829532
XLOC_025049	Ca_v2.0_22787	Ca7_v2.0	61961556	61962299
XLOC_025051	Ca_v2.0_22792	Ca7_v2.0	62013304	62024985
XLOC_025055	Ca_v2.0_22797	Ca7_v2.0	62052127	62053843
XLOC_025070	Ca_v2.0_22824	Ca7_v2.0	62301808	62303920
XLOC_025071	Ca_v2.0_22825	Ca7_v2.0	62308147	62321399
XLOC_025076	Ca_v2.0_22832	Ca7_v2.0	62376267	62382740
XLOC_025084	-	Ca7_v2.0	718879	720090
XLOC_025086	-	Ca7_v2.0	920661	921842
XLOC_025089	-	Ca7_v2.0	1730222	1730436
XLOC_025091	-	Ca7_v2.0	1815359	1816320
XLOC_025099	-	Ca7_v2.0	4474032	4474468
XLOC_025102	-	Ca7_v2.0	5868380	5869195
XLOC_025103	-	Ca7_v2.0	5926569	5927126
XLOC_025106	-	Ca7_v2.0	6758045	6758812
XLOC_025118	-	Ca7_v2.0	10375202	10375736
XLOC_025119	-	Ca7_v2.0	10548723	10549950
XLOC_025132	-	Ca7_v2.0	15335769	15337044
XLOC_025133	-	Ca7_v2.0	16027151	16028143
XLOC_025134	-	Ca7_v2.0	16262630	16264066
XLOC_025139	-	Ca7_v2.0	21759211	21760664
XLOC_025141	-	Ca7_v2.0	21771791	21773298
XLOC_025149	-	Ca7_v2.0	24029533	24030564
XLOC_025155	-	Ca7_v2.0	27393465	27394159
XLOC_025158	-	Ca7_v2.0	31306097	31345735
XLOC_025164	-	Ca7_v2.0	42490917	42499665
XLOC_025167	-	Ca7_v2.0	44211368	44211616
XLOC_025173	-	Ca7_v2.0	47082608	47082876
XLOC_025174	-	Ca7_v2.0	47120866	47122014
XLOC_025183	-	Ca7_v2.0	54545959	54546499
XLOC_025188	-	Ca7_v2.0	56923511	56923789
XLOC_025199	-	Ca7_v2.0	61036265	61037337
XLOC_025203	-	Ca7_v2.0	61335379	61335533
XLOC_025213	Ca_v2.0_22851	Ca8_v2.0	161762	164961
XLOC_025217	Ca_v2.0_22861	Ca8_v2.0	214998	230464
XLOC_025218	Ca_v2.0_22863	Ca8_v2.0	235899	237310
XLOC_025221	Ca_v2.0_22867	Ca8_v2.0	253404	255638
XLOC_025224	Ca_v2.0_22873	Ca8_v2.0	288071	292081
XLOC_025226	Ca_v2.0_22883	Ca8_v2.0	381065	382838
XLOC_025233	Ca_v2.0_22894	Ca8_v2.0	451865	453573
XLOC_025234	Ca_v2.0_22896	Ca8_v2.0	481857	536023
XLOC_025242	Ca_v2.0_22909	Ca8_v2.0	603267	605757
XLOC_025253	Ca_v2.0_22933	Ca8_v2.0	726048	732048
XLOC_025255	Ca_v2.0_22936	Ca8_v2.0	742004	747510

For Review Only

XLOC_025263	Ca_v2.0_22949	Ca8_v2.0	829310	831692
XLOC_025270	Ca_v2.0_22960	Ca8_v2.0	914836	921425
XLOC_025272	Ca_v2.0_22963	Ca8_v2.0	927817	932678
XLOC_025274	Ca_v2.0_22965	Ca8_v2.0	973108	990500
XLOC_025279	Ca_v2.0_22976	Ca8_v2.0	1081301	1084255
XLOC_025286	Ca_v2.0_22988	Ca8_v2.0	1147088	1150337
XLOC_025287	Ca_v2.0_22990	Ca8_v2.0	1165740	1167047
XLOC_025295	Ca_v2.0_22999	Ca8_v2.0	1231754	1235024
XLOC_025299	Ca_v2.0_23007	Ca8_v2.0	1284835	1289820
XLOC_025307	Ca_v2.0_23024	Ca8_v2.0	1429537	1430697
XLOC_025311	-	Ca8_v2.0	1530395	1531309
XLOC_025314	Ca_v2.0_23041	Ca8_v2.0	1558193	1572609
XLOC_025317	Ca_v2.0_23045	Ca8_v2.0	1622962	1627307
XLOC_025323	Ca_v2.0_23052	Ca8_v2.0	1662583	1665230
XLOC_025324	-	Ca8_v2.0	1665995	1667122
XLOC_025327	Ca_v2.0_23056	Ca8_v2.0	1682222	1682836
XLOC_025336	Ca_v2.0_23076	Ca8_v2.0	1862311	1869630
XLOC_025338	Ca_v2.0_23081	Ca8_v2.0	1903674	1907421
XLOC_025339	Ca_v2.0_23082	Ca8_v2.0	1917122	1919223
XLOC_025340	Ca_v2.0_23084	Ca8_v2.0	1954577	1955345
XLOC_025344	Ca_v2.0_23088	Ca8_v2.0	1998202	2002805
XLOC_025352	Ca_v2.0_23106,Ca_v2.0_231(	Ca8_v2.0	2109233	2113740
XLOC_025365	Ca_v2.0_23145	Ca8_v2.0	2392024	2398095
XLOC_025368	Ca_v2.0_23152	Ca8_v2.0	2478266	2480289
XLOC_025369	Ca_v2.0_23153	Ca8_v2.0	2480843	2483659
XLOC_025373	Ca_v2.0_23162	Ca8_v2.0	2552220	2554932
XLOC_025375	Ca_v2.0_23166	Ca8_v2.0	2576127	2577441
XLOC_025378	Ca_v2.0_23172	Ca8_v2.0	2613704	2615496
XLOC_025386	Ca_v2.0_23185	Ca8_v2.0	2698898	2704607
XLOC_025390	Ca_v2.0_23192	Ca8_v2.0	2736480	2739887
XLOC_025392	Ca_v2.0_23195	Ca8_v2.0	2798399	2800222
XLOC_025396	Ca_v2.0_23201	Ca8_v2.0	2937329	2942313
XLOC_025402	Ca_v2.0_23209	Ca8_v2.0	2998674	3003249
XLOC_025408	Ca_v2.0_23223	Ca8_v2.0	3092744	3093609
XLOC_025412	Ca_v2.0_23230	Ca8_v2.0	3150593	3153128
XLOC_025414	Ca_v2.0_23232	Ca8_v2.0	3169662	3171339
XLOC_025416	Ca_v2.0_23234	Ca8_v2.0	3203965	3207516
XLOC_025417	Ca_v2.0_23238	Ca8_v2.0	3228355	3232651
XLOC_025424	Ca_v2.0_23250	Ca8_v2.0	3380197	3380837
XLOC_025426	Ca_v2.0_23256	Ca8_v2.0	3418295	3421746
XLOC_025428	Ca_v2.0_23260	Ca8_v2.0	3449866	3458523
XLOC_025433	Ca_v2.0_23271	Ca8_v2.0	3554943	3563924
XLOC_025435	Ca_v2.0_23277	Ca8_v2.0	3601151	3606975
XLOC_025436	Ca_v2.0_23278	Ca8_v2.0	3616257	3618077
XLOC_025438	Ca_v2.0_23282	Ca8_v2.0	3658958	3663738
XLOC_025441	Ca_v2.0_23288	Ca8_v2.0	3756026	3764661

For Review Only

XLOC_025445	Ca_v2.0_23303	Ca8_v2.0	3880549	3884021
XLOC_025448	Ca_v2.0_23309	Ca8_v2.0	3897538	3995462
XLOC_025451	Ca_v2.0_23315	Ca8_v2.0	3897538	3995462
XLOC_025456	Ca_v2.0_23321	Ca8_v2.0	4016016	4020277
XLOC_025465	Ca_v2.0_23336	Ca8_v2.0	4149879	4156200
XLOC_025467	Ca_v2.0_23339	Ca8_v2.0	4168441	4174768
XLOC_025469	Ca_v2.0_23343	Ca8_v2.0	4222226	4224175
XLOC_025474	Ca_v2.0_23348	Ca8_v2.0	4247098	4248485
XLOC_025475	Ca_v2.0_23350	Ca8_v2.0	4266859	4274393
XLOC_025476	Ca_v2.0_23352	Ca8_v2.0	4299267	4303601
XLOC_025477	-	Ca8_v2.0	4303973	4308110
XLOC_025480	Ca_v2.0_23360	Ca8_v2.0	4401923	4405574
XLOC_025482	Ca_v2.0_23364	Ca8_v2.0	4452244	4457308
XLOC_025484	Ca_v2.0_23367	Ca8_v2.0	4467463	4480796
XLOC_025485	Ca_v2.0_23371	Ca8_v2.0	4487447	4492032
XLOC_025491	Ca_v2.0_23380	Ca8_v2.0	4542278	4542835
XLOC_025494	Ca_v2.0_23386	Ca8_v2.0	4563494	4563967
XLOC_025496	Ca_v2.0_23388	Ca8_v2.0	4576552	4577351
XLOC_025497	Ca_v2.0_23389	Ca8_v2.0	4584491	4587365
XLOC_025501	Ca_v2.0_23396	Ca8_v2.0	4683031	4984382
XLOC_025502	Ca_v2.0_23419	Ca8_v2.0	4683031	4984382
XLOC_025503	Ca_v2.0_23397	Ca8_v2.0	4683031	4984382
XLOC_025505	Ca_v2.0_23399	Ca8_v2.0	4683031	4984382
XLOC_025506	Ca_v2.0_23402	Ca8_v2.0	4683031	4984382
XLOC_025508	Ca_v2.0_23410	Ca8_v2.0	4683031	4984382
XLOC_025509	Ca_v2.0_23411	Ca8_v2.0	4683031	4984382
XLOC_025510	Ca_v2.0_23414	Ca8_v2.0	4683031	4984382
XLOC_025517	Ca_v2.0_23424	Ca8_v2.0	4683031	4984382
XLOC_025523	Ca_v2.0_23432	Ca8_v2.0	5024012	5030245
XLOC_025532	Ca_v2.0_23452	Ca8_v2.0	5245370	5250889
XLOC_025533	Ca_v2.0_23454	Ca8_v2.0	5254370	5257506
XLOC_025535	Ca_v2.0_23460	Ca8_v2.0	5310666	5312621
XLOC_025553	Ca_v2.0_23484	Ca8_v2.0	5484497	5486534
XLOC_025561	Ca_v2.0_23500	Ca8_v2.0	5688551	5695395
XLOC_025567	Ca_v2.0_23511	Ca8_v2.0	5828230	5834148
XLOC_025570	Ca_v2.0_23514	Ca8_v2.0	5866749	5870329
XLOC_025571	Ca_v2.0_23515	Ca8_v2.0	5877658	5880595
XLOC_025572	Ca_v2.0_23516	Ca8_v2.0	5884824	5887526
XLOC_025574	Ca_v2.0_23520	Ca8_v2.0	5910973	5912743
XLOC_025578	Ca_v2.0_23524	Ca8_v2.0	5943310	5952971
XLOC_025581	Ca_v2.0_23531	Ca8_v2.0	5979458	5982214
XLOC_025593	Ca_v2.0_23552	Ca8_v2.0	6185820	6186365
XLOC_025601	Ca_v2.0_23561	Ca8_v2.0	6305385	6307041
XLOC_025619	Ca_v2.0_23585	Ca8_v2.0	6559144	6567247
XLOC_025622	Ca_v2.0_23592	Ca8_v2.0	6613676	6615130
XLOC_025629	Ca_v2.0_23608	Ca8_v2.0	6733951	6734982

For Review Only

XLOC_025634	Ca_v2.0_23620	Ca8_v2.0	6918773	6922195
XLOC_025635	Ca_v2.0_23622	Ca8_v2.0	6932454	6934253
XLOC_025636	Ca_v2.0_23624	Ca8_v2.0	6946747	6950544
XLOC_025638	Ca_v2.0_23627	Ca8_v2.0	6980902	6985593
XLOC_025644	Ca_v2.0_23636	Ca8_v2.0	7047553	7049834
XLOC_025647	Ca_v2.0_23641	Ca8_v2.0	7119780	7123132
XLOC_025654	Ca_v2.0_23654	Ca8_v2.0	7217954	7220875
XLOC_025659	Ca_v2.0_23662	Ca8_v2.0	7311906	7315737
XLOC_025664	Ca_v2.0_23673	Ca8_v2.0	7464286	7465768
XLOC_025667	Ca_v2.0_23679	Ca8_v2.0	7488987	7494943
XLOC_025673	Ca_v2.0_23693	Ca8_v2.0	7656677	7660450
XLOC_025675	Ca_v2.0_23697	Ca8_v2.0	7685976	7688482
XLOC_025678	Ca_v2.0_23701	Ca8_v2.0	7707851	7713022
XLOC_025682	Ca_v2.0_23706	Ca8_v2.0	7750245	7755074
XLOC_025696	-	Ca8_v2.0	8105380	8106924
XLOC_025708	Ca_v2.0_23765	Ca8_v2.0	8381643	8389219
XLOC_025719	Ca_v2.0_23782	Ca8_v2.0	8635877	8638263
XLOC_025721	Ca_v2.0_23788	Ca8_v2.0	8690829	8694725
XLOC_025722	Ca_v2.0_23791	Ca8_v2.0	8717501	8719526
XLOC_025724	Ca_v2.0_23795	Ca8_v2.0	8799802	8801753
XLOC_025730	Ca_v2.0_23813	Ca8_v2.0	9051808	9052941
XLOC_025740	Ca_v2.0_23830	Ca8_v2.0	9247607	9250760
XLOC_025748	Ca_v2.0_23846	Ca8_v2.0	9434843	9440356
XLOC_025766	Ca_v2.0_23877	Ca8_v2.0	9947945	9951753
XLOC_025771	Ca_v2.0_23891	Ca8_v2.0	10115105	10120010
XLOC_025788	Ca_v2.0_23920	Ca8_v2.0	10572676	10573654
XLOC_025792	Ca_v2.0_23928	Ca8_v2.0	10736076	10741586
XLOC_025795	Ca_v2.0_23931	Ca8_v2.0	10795226	10798055
XLOC_025800	Ca_v2.0_23940	Ca8_v2.0	10991234	10993622
XLOC_025805	Ca_v2.0_23952	Ca8_v2.0	11400897	11403797
XLOC_025809	Ca_v2.0_23962	Ca8_v2.0	11552795	11556364
XLOC_025819	Ca_v2.0_23980	Ca8_v2.0	12016977	12022228
XLOC_025822	Ca_v2.0_23984	Ca8_v2.0	12128018	12129905
XLOC_025823	Ca_v2.0_23988	Ca8_v2.0	12214158	12216289
XLOC_025824	Ca_v2.0_23989	Ca8_v2.0	12220125	12221564
XLOC_025826	Ca_v2.0_23993	Ca8_v2.0	12247273	12432556
XLOC_025828	Ca_v2.0_23998	Ca8_v2.0	12459234	12460527
XLOC_025829	Ca_v2.0_24001	Ca8_v2.0	12559804	12568283
XLOC_025830	Ca_v2.0_24002	Ca8_v2.0	12569684	12571401
XLOC_025839	Ca_v2.0_24019	Ca8_v2.0	13121903	13186446
XLOC_025841	Ca_v2.0_24023	Ca8_v2.0	13238027	13240244
XLOC_025842	Ca_v2.0_24029	Ca8_v2.0	13408006	13411852
XLOC_025855	Ca_v2.0_24051	Ca8_v2.0	14067404	14069220
XLOC_025862	Ca_v2.0_24068	Ca8_v2.0	16358006	16362862
XLOC_025863	Ca_v2.0_24072	Ca8_v2.0	16562086	16562744
XLOC_025876	Ca_v2.0_24096	Ca8_v2.0	17191452	17195933

For Review Only

XLOC_025887	Ca_v2.0_24115	Ca8_v2.0	18265795	18268871
XLOC_025893	Ca_v2.0_24127	Ca8_v2.0	18866107	18879321
XLOC_025895	Ca_v2.0_24131	Ca8_v2.0	18923665	18987918
XLOC_025904	Ca_v2.0_24144	Ca8_v2.0	19551949	19553013
XLOC_025911	Ca_v2.0_24161	Ca8_v2.0	19782847	19788008
XLOC_025913	Ca_v2.0_24165	Ca8_v2.0	19853437	19856649
XLOC_025915	Ca_v2.0_24167	Ca8_v2.0	19990519	19993012
XLOC_025921	Ca_v2.0_24184,Ca_v2.0_241	Ca8_v2.0	20794271	20800223
XLOC_025922	Ca_v2.0_24188	Ca8_v2.0	20819419	20822949
XLOC_025936	Ca_v2.0_24217	Ca8_v2.0	21884294	21886290
XLOC_025945	-	Ca8_v2.0	22190432	22191456
XLOC_025960	Ca_v2.0_24266	Ca8_v2.0	22832451	22835726
XLOC_025963	Ca_v2.0_24272	Ca8_v2.0	22948780	22953279
XLOC_025972	Ca_v2.0_24289	Ca8_v2.0	23172211	23178309
XLOC_025977	Ca_v2.0_24298	Ca8_v2.0	23238893	23240331
XLOC_025989	Ca_v2.0_24313	Ca8_v2.0	23539027	23546321
XLOC_025995	Ca_v2.0_24326	Ca8_v2.0	23737735	23784022
XLOC_026005	Ca_v2.0_24337	Ca8_v2.0	23906491	23915493
XLOC_026006	Ca_v2.0_24339	Ca8_v2.0	23927449	23930076
XLOC_026012	Ca_v2.0_24354	Ca8_v2.0	24086920	24089543
XLOC_026023	Ca_v2.0_24375	Ca8_v2.0	24416448	24425364
XLOC_026031	Ca_v2.0_24389	Ca8_v2.0	24555248	24559728
XLOC_026032	Ca_v2.0_24390	Ca8_v2.0	24561743	24562118
XLOC_026041	Ca_v2.0_24413	Ca8_v2.0	24923105	24927450
XLOC_026045	Ca_v2.0_24419	Ca8_v2.0	25013496	25118860
XLOC_026046	Ca_v2.0_24418	Ca8_v2.0	25013496	25118860
XLOC_026048	Ca_v2.0_24420	Ca8_v2.0	25013496	25118860
XLOC_026059	Ca_v2.0_24438	Ca8_v2.0	25443774	25445635
XLOC_026061	Ca_v2.0_24441	Ca8_v2.0	25477593	25478837
XLOC_026067	-	Ca8_v2.0	25609016	25612624
XLOC_026070	Ca_v2.0_24454	Ca8_v2.0	25655224	25662845
XLOC_026073	Ca_v2.0_24460	Ca8_v2.0	25690840	25711329
XLOC_026097	-	Ca8_v2.0	26637734	26639496
XLOC_026100	Ca_v2.0_24525	Ca8_v2.0	26664074	26665030
XLOC_026105	Ca_v2.0_24531	Ca8_v2.0	26723659	26731924
XLOC_026113	Ca_v2.0_24542	Ca8_v2.0	26839066	26849770
XLOC_026115	-	Ca8_v2.0	26873552	26876333
XLOC_026124	Ca_v2.0_24567	Ca8_v2.0	27064416	27073475
XLOC_026127	Ca_v2.0_24576	Ca8_v2.0	27190161	27199757
XLOC_026129	Ca_v2.0_24578	Ca8_v2.0	27220571	27220990
XLOC_026134	Ca_v2.0_24590	Ca8_v2.0	27390985	27395165
XLOC_026137	Ca_v2.0_24595	Ca8_v2.0	27461283	27464138
XLOC_026148	-	Ca8_v2.0	27672448	27687585
XLOC_026149	Ca_v2.0_24616	Ca8_v2.0	27704646	27707955
XLOC_026154	-	Ca8_v2.0	27768642	27769785
XLOC_026157	Ca_v2.0_24625	Ca8_v2.0	27802032	27807493

For Review Only

XLOC_026158	Ca_v2.0_24626	Ca8_v2.0	27814237	27815443
XLOC_026159	Ca_v2.0_24627	Ca8_v2.0	27840221	28014920
XLOC_026160	Ca_v2.0_24629	Ca8_v2.0	27840221	28014920
XLOC_026163	-	Ca8_v2.0	27840221	28014920
XLOC_026173	Ca_v2.0_24647	Ca8_v2.0	28063816	28065604
XLOC_026177	Ca_v2.0_24659	Ca8_v2.0	28219530	28225061
XLOC_026180	Ca_v2.0_24666	Ca8_v2.0	28297317	28299552
XLOC_026189	Ca_v2.0_24685	Ca8_v2.0	28437472	28439676
XLOC_026190	Ca_v2.0_24686	Ca8_v2.0	28449814	28451631
XLOC_026201	-	Ca8_v2.0	12279	16622
XLOC_026207	Ca_v2.0_22857,Ca_v2.0_228!	Ca8_v2.0	192811	204257
XLOC_026211	Ca_v2.0_22863	Ca8_v2.0	235899	237310
XLOC_026212	Ca_v2.0_22866	Ca8_v2.0	249879	250491
XLOC_026220	Ca_v2.0_22877	Ca8_v2.0	312955	316397
XLOC_026225	Ca_v2.0_22884	Ca8_v2.0	390524	394692
XLOC_026226	Ca_v2.0_22886	Ca8_v2.0	396894	403582
XLOC_026232	Ca_v2.0_22902	Ca8_v2.0	544373	545723
XLOC_026234	Ca_v2.0_22905	Ca8_v2.0	564249	575000
XLOC_026236	Ca_v2.0_22907	Ca8_v2.0	592434	596600
XLOC_026238	Ca_v2.0_22912	Ca8_v2.0	615639	618363
XLOC_026239	Ca_v2.0_22914	Ca8_v2.0	623964	625655
XLOC_026259	Ca_v2.0_22957	Ca8_v2.0	890220	895020
XLOC_026260	Ca_v2.0_22958	Ca8_v2.0	897153	901231
XLOC_026262	Ca_v2.0_22964	Ca8_v2.0	934357	971208
XLOC_026265	Ca_v2.0_22969	Ca8_v2.0	1007797	1025792
XLOC_026281	Ca_v2.0_23006	Ca8_v2.0	1270241	1276614
XLOC_026283	Ca_v2.0_23010	Ca8_v2.0	1304545	1304890
XLOC_026292	Ca_v2.0_23026	Ca8_v2.0	1438632	1439580
XLOC_026297	Ca_v2.0_23033	Ca8_v2.0	1499310	1502591
XLOC_026303	Ca_v2.0_23047	Ca8_v2.0	1628876	1637141
XLOC_026307	Ca_v2.0_23059	Ca8_v2.0	1714205	1716736
XLOC_026311	Ca_v2.0_23068	Ca8_v2.0	1791150	1797476
XLOC_026315	Ca_v2.0_23074	Ca8_v2.0	1850604	1851740
XLOC_026316	Ca_v2.0_23075	Ca8_v2.0	1855947	1856940
XLOC_026317	Ca_v2.0_23076	Ca8_v2.0	1862311	1869630
XLOC_026323	Ca_v2.0_23089	Ca8_v2.0	2003372	2006916
XLOC_026336	Ca_v2.0_23117	Ca8_v2.0	2180807	2193121
XLOC_026340	Ca_v2.0_23122	Ca8_v2.0	2212989	2216186
XLOC_026341	Ca_v2.0_23125	Ca8_v2.0	2225846	2227905
XLOC_026342	Ca_v2.0_23127	Ca8_v2.0	2241690	2245741
XLOC_026348	Ca_v2.0_23136	Ca8_v2.0	2322849	2324291
XLOC_026353	Ca_v2.0_23141	Ca8_v2.0	2356294	2358232
XLOC_026360	Ca_v2.0_23149	Ca8_v2.0	2435064	2436628
XLOC_026363	Ca_v2.0_23154	Ca8_v2.0	2483896	2486943
XLOC_026366	Ca_v2.0_23160	Ca8_v2.0	2522695	2528524
XLOC_026368	Ca_v2.0_23162	Ca8_v2.0	2552220	2554932

For Review Only

XLOC_026378	Ca_v2.0_23177	Ca8_v2.0	2637513	2640046
XLOC_026379	Ca_v2.0_23182	Ca8_v2.0	2674878	2677565
XLOC_026382	Ca_v2.0_23186	Ca8_v2.0	2698898	2704607
XLOC_026385	Ca_v2.0_23193	Ca8_v2.0	2740718	2743646
XLOC_026386	Ca_v2.0_23195	Ca8_v2.0	2798399	2800222
XLOC_026387	Ca_v2.0_23197	Ca8_v2.0	2882168	2891252
XLOC_026393	Ca_v2.0_23207	Ca8_v2.0	2970229	2976296
XLOC_026394	Ca_v2.0_23211	Ca8_v2.0	3007125	3008714
XLOC_026395	Ca_v2.0_23213	Ca8_v2.0	3009642	3021389
XLOC_026397	Ca_v2.0_23216	Ca8_v2.0	3043955	3048266
XLOC_026404	Ca_v2.0_23237	Ca8_v2.0	3217470	3222702
XLOC_026408	Ca_v2.0_23245	Ca8_v2.0	3317195	3319451
XLOC_026411	Ca_v2.0_23248	Ca8_v2.0	3363092	3363947
XLOC_026412	Ca_v2.0_23251	Ca8_v2.0	3382430	3384344
XLOC_026413	Ca_v2.0_23254	Ca8_v2.0	3386214	3397583
XLOC_026421	Ca_v2.0_23267	Ca8_v2.0	3531901	3536159
XLOC_026423	Ca_v2.0_23270	Ca8_v2.0	3547386	3551427
XLOC_026424	Ca_v2.0_23272	Ca8_v2.0	3554943	3563924
XLOC_026430	Ca_v2.0_23284	Ca8_v2.0	3693944	3696738
XLOC_026441	Ca_v2.0_23299	Ca8_v2.0	3838692	3841546
XLOC_026446	Ca_v2.0_23310	Ca8_v2.0	3897538	3995462
XLOC_026452	Ca_v2.0_23317	Ca8_v2.0	3897538	3995462
XLOC_026466	-	Ca8_v2.0	4286222	4288229
XLOC_026470	Ca_v2.0_23358	Ca8_v2.0	4371998	4374630
XLOC_026478	Ca_v2.0_23372	Ca8_v2.0	4492196	4497117
XLOC_026483	Ca_v2.0_23384	Ca8_v2.0	4557954	4558783
XLOC_026485	Ca_v2.0_23393	Ca8_v2.0	4619387	4625622
XLOC_026487	Ca_v2.0_23412	Ca8_v2.0	4683031	4984382
XLOC_026489	-	Ca8_v2.0	4683031	4984382
XLOC_026491	Ca_v2.0_23403	Ca8_v2.0	4683031	4984382
XLOC_026495	Ca_v2.0_23407	Ca8_v2.0	4683031	4984382
XLOC_026501	Ca_v2.0_23422	Ca8_v2.0	4683031	4984382
XLOC_026503	Ca_v2.0_23428	Ca8_v2.0	4683031	4984382
XLOC_026505	Ca_v2.0_23434	Ca8_v2.0	5050769	5052155
XLOC_026506	Ca_v2.0_23436	Ca8_v2.0	5090427	5090971
XLOC_026511	Ca_v2.0_23444	Ca8_v2.0	5180562	5184563
XLOC_026515	Ca_v2.0_23449	Ca8_v2.0	5209777	5210831
XLOC_026518	Ca_v2.0_23454	Ca8_v2.0	5254370	5257506
XLOC_026523	Ca_v2.0_23467	Ca8_v2.0	5368402	5372545
XLOC_026527	Ca_v2.0_23476	Ca8_v2.0	5426562	5432166
XLOC_026528	Ca_v2.0_23478	Ca8_v2.0	5435619	5441852
XLOC_026532	Ca_v2.0_23488	Ca8_v2.0	5523477	5527874
XLOC_026534	Ca_v2.0_23490	Ca8_v2.0	5544510	5549101
XLOC_026537	Ca_v2.0_23493	Ca8_v2.0	5594580	5597161
XLOC_026538	Ca_v2.0_23494	Ca8_v2.0	5607697	5609204
XLOC_026540	Ca_v2.0_23499	Ca8_v2.0	5683480	5684466

For Review Only

XLOC_026545	Ca_v2.0_23509	Ca8_v2.0	5818394	5820266
XLOC_026546	Ca_v2.0_23516	Ca8_v2.0	5884824	5887526
XLOC_026547	Ca_v2.0_23518	Ca8_v2.0	5899391	5900364
XLOC_026548	Ca_v2.0_23519	Ca8_v2.0	5906654	5909768
XLOC_026549	Ca_v2.0_23522	Ca8_v2.0	5922499	5929757
XLOC_026550	Ca_v2.0_23525	Ca8_v2.0	5943310	5952971
XLOC_026554	Ca_v2.0_23536	Ca8_v2.0	6029128	6036808
XLOC_026559	Ca_v2.0_23544	Ca8_v2.0	6110647	6120766
XLOC_026563	Ca_v2.0_23557	Ca8_v2.0	6218269	6224018
XLOC_026564	Ca_v2.0_23559	Ca8_v2.0	6269290	6276719
XLOC_026565	Ca_v2.0_23559	Ca8_v2.0	6269290	6276719
XLOC_026566	-	Ca8_v2.0	6325076	6330638
XLOC_026569	Ca_v2.0_23563	Ca8_v2.0	6356784	6360312
XLOC_026581	Ca_v2.0_23586	Ca8_v2.0	6559144	6567247
XLOC_026587	Ca_v2.0_23596	Ca8_v2.0	6653509	6661414
XLOC_026594	Ca_v2.0_23605	Ca8_v2.0	6708377	6711648
XLOC_026596	Ca_v2.0_23609	Ca8_v2.0	6742805	6747120
XLOC_026609	Ca_v2.0_23626	Ca8_v2.0	6964143	6973266
XLOC_026610	Ca_v2.0_23628	Ca8_v2.0	6986367	6987597
XLOC_026611	Ca_v2.0_23630	Ca8_v2.0	7004880	7013894
XLOC_026612	Ca_v2.0_23632	Ca8_v2.0	7028276	7029659
XLOC_026627	Ca_v2.0_23660	Ca8_v2.0	7301333	7304655
XLOC_026633	Ca_v2.0_23670	Ca8_v2.0	7425155	7431425
XLOC_026636	Ca_v2.0_23676	Ca8_v2.0	7477321	7481145
XLOC_026637	Ca_v2.0_23677	Ca8_v2.0	7482948	7483464
XLOC_026638	Ca_v2.0_23679	Ca8_v2.0	7488987	7494943
XLOC_026657	Ca_v2.0_23715	Ca8_v2.0	7806019	7808911
XLOC_026668	Ca_v2.0_23729	Ca8_v2.0	7958719	7962009
XLOC_026680	Ca_v2.0_23754	Ca8_v2.0	8228588	8232782
XLOC_026690	Ca_v2.0_23773	Ca8_v2.0	8491612	8493067
XLOC_026696	Ca_v2.0_23781	Ca8_v2.0	8630088	8632322
XLOC_026698	Ca_v2.0_23785	Ca8_v2.0	8666498	8667716
XLOC_026701	Ca_v2.0_23788	Ca8_v2.0	8690829	8694725
XLOC_026707	Ca_v2.0_23798	Ca8_v2.0	8811442	8814761
XLOC_026712	Ca_v2.0_23806	Ca8_v2.0	8883250	8890406
XLOC_026716	Ca_v2.0_23810	Ca8_v2.0	8928244	8935794
XLOC_026717	-	Ca8_v2.0	8951002	8952828
XLOC_026720	Ca_v2.0_23815	Ca8_v2.0	9071659	9074178
XLOC_026725	Ca_v2.0_23824	Ca8_v2.0	9164760	9170000
XLOC_026726	Ca_v2.0_23825	Ca8_v2.0	9189511	9198226
XLOC_026727	Ca_v2.0_23829	Ca8_v2.0	9236754	9245626
XLOC_026728	Ca_v2.0_23831	Ca8_v2.0	9259708	9263344
XLOC_026729	Ca_v2.0_23833	Ca8_v2.0	9283947	9286440
XLOC_026738	Ca_v2.0_23850	Ca8_v2.0	9479040	9483902
XLOC_026741	Ca_v2.0_23855	Ca8_v2.0	9555219	9558044
XLOC_026742	Ca_v2.0_23858	Ca8_v2.0	9594862	9599424

For Review Only

XLOC_026747	Ca_v2.0_23867	Ca8_v2.0	9733226	9735486
XLOC_026750	Ca_v2.0_23873	Ca8_v2.0	9865234	9866387
XLOC_026751	Ca_v2.0_23878	Ca8_v2.0	9952850	9954010
XLOC_026753	Ca_v2.0_23882	Ca8_v2.0	10027427	10028953
XLOC_026757	Ca_v2.0_23888	Ca8_v2.0	10085111	10091447
XLOC_026761	Ca_v2.0_23895	Ca8_v2.0	10150816	10155387
XLOC_026766	Ca_v2.0_23903	Ca8_v2.0	10298651	10323181
XLOC_026790	Ca_v2.0_23953,Ca_v2.0_239	Ca8_v2.0	11409030	11411412
XLOC_026799	Ca_v2.0_23967	Ca8_v2.0	11670426	11671796
XLOC_026807	Ca_v2.0_23985	Ca8_v2.0	12190638	12193310
XLOC_026822	Ca_v2.0_24013,Ca_v2.0_240	Ca8_v2.0	12999551	13057096
XLOC_026823	Ca_v2.0_24019,Ca_v2.0_240	Ca8_v2.0	13121903	13186446
XLOC_026825	Ca_v2.0_24023	Ca8_v2.0	13238027	13240244
XLOC_026844	Ca_v2.0_24050	Ca8_v2.0	14036945	14038623
XLOC_026845	Ca_v2.0_24052,Ca_v2.0_240	Ca8_v2.0	14089309	14175214
XLOC_026846	Ca_v2.0_24053	Ca8_v2.0	14089309	14175214
XLOC_026847	Ca_v2.0_24055	Ca8_v2.0	14222223	14223477
XLOC_026848	Ca_v2.0_24058	Ca8_v2.0	14273872	14277142
XLOC_026849	Ca_v2.0_24060	Ca8_v2.0	14300501	14308050
XLOC_026871	Ca_v2.0_24096	Ca8_v2.0	17191452	17195933
XLOC_026898	Ca_v2.0_24150	Ca8_v2.0	19651311	19654748
XLOC_026901	Ca_v2.0_24154	Ca8_v2.0	19705471	19707444
XLOC_026913	Ca_v2.0_24174	Ca8_v2.0	20237041	20248791
XLOC_026918	Ca_v2.0_24183	Ca8_v2.0	20787961	20794085
XLOC_026925	Ca_v2.0_24195	Ca8_v2.0	21109463	21111336
XLOC_026929	Ca_v2.0_24201	Ca8_v2.0	21246761	21253011
XLOC_026933	-	Ca8_v2.0	21306842	21308183
XLOC_026937	Ca_v2.0_24214	Ca8_v2.0	21677327	21682819
XLOC_026943	Ca_v2.0_24223	Ca8_v2.0	22057626	22060071
XLOC_026950	Ca_v2.0_24238	Ca8_v2.0	22263698	22265940
XLOC_026965	Ca_v2.0_24263	Ca8_v2.0	22788853	22789570
XLOC_026967	Ca_v2.0_24269	Ca8_v2.0	22919079	22922922
XLOC_026970	Ca_v2.0_24278	Ca8_v2.0	23007614	23008564
XLOC_026975	Ca_v2.0_24284	Ca8_v2.0	23105848	23106790
XLOC_026979	Ca_v2.0_24292	Ca8_v2.0	23194100	23198396
XLOC_027004	Ca_v2.0_24349	Ca8_v2.0	24037322	24038190
XLOC_027011	Ca_v2.0_24364	Ca8_v2.0	24268598	24277774
XLOC_027016	Ca_v2.0_24369	Ca8_v2.0	24323442	24334527
XLOC_027017	Ca_v2.0_24370	Ca8_v2.0	24336436	24344090
XLOC_027018	Ca_v2.0_24371	Ca8_v2.0	24360390	24366842
XLOC_027022	Ca_v2.0_24374	Ca8_v2.0	24406849	24407598
XLOC_027023	Ca_v2.0_24375,Ca_v2.0_243	Ca8_v2.0	24416448	24425364
XLOC_027024	Ca_v2.0_24376	Ca8_v2.0	24416448	24425364
XLOC_027034	Ca_v2.0_24401	Ca8_v2.0	24720614	24725297
XLOC_027036	Ca_v2.0_24403	Ca8_v2.0	24803023	24809673
XLOC_027038	Ca_v2.0_24405,Ca_v2.0_244	Ca8_v2.0	24831683	24843217

For Review Only

XLOC_027040	Ca_v2.0_24411	Ca8_v2.0	24910374	24912790
XLOC_027042	Ca_v2.0_24420	Ca8_v2.0	25013496	25118860
XLOC_027046	Ca_v2.0_24430	Ca8_v2.0	25348041	25351203
XLOC_027049	Ca_v2.0_24435	Ca8_v2.0	25410387	25414845
XLOC_027054	Ca_v2.0_24449	Ca8_v2.0	25596233	25597066
XLOC_027061	Ca_v2.0_24463	Ca8_v2.0	25751854	25757647
XLOC_027065	Ca_v2.0_24469	Ca8_v2.0	25957825	25963613
XLOC_027072	Ca_v2.0_24479	Ca8_v2.0	26111329	26115305
XLOC_027074	Ca_v2.0_24482	Ca8_v2.0	26146242	26151183
XLOC_027077	Ca_v2.0_24487	Ca8_v2.0	26280100	26293408
XLOC_027086	-	Ca8_v2.0	26410480	26411259
XLOC_027093	Ca_v2.0_24509	Ca8_v2.0	26515462	26517593
XLOC_027096	Ca_v2.0_24513	Ca8_v2.0	26547668	26551053
XLOC_027099	Ca_v2.0_24518	Ca8_v2.0	26592750	26596510
XLOC_027102	Ca_v2.0_24521	Ca8_v2.0	26623918	26626381
XLOC_027104	Ca_v2.0_24525	Ca8_v2.0	26664074	26665030
XLOC_027114	Ca_v2.0_24551	Ca8_v2.0	26929258	26934240
XLOC_027116	-	Ca8_v2.0	26943496	26948239
XLOC_027117	Ca_v2.0_24553	Ca8_v2.0	26949570	26952500
XLOC_027122	Ca_v2.0_24562	Ca8_v2.0	27019262	27022956
XLOC_027129	Ca_v2.0_24569	Ca8_v2.0	27098102	27101742
XLOC_027130	Ca_v2.0_24570	Ca8_v2.0	27133394	27140537
XLOC_027134	Ca_v2.0_24575	Ca8_v2.0	27190161	27199757
XLOC_027136	-	Ca8_v2.0	27210609	27211349
XLOC_027141	Ca_v2.0_24586	Ca8_v2.0	27337903	27339805
XLOC_027155	Ca_v2.0_24612	Ca8_v2.0	27659781	27660920
XLOC_027157	Ca_v2.0_24620	Ca8_v2.0	27748605	27749121
XLOC_027160	Ca_v2.0_24627	Ca8_v2.0	27840221	28014920
XLOC_027161	Ca_v2.0_24628	Ca8_v2.0	27840221	28014920
XLOC_027163	Ca_v2.0_24635	Ca8_v2.0	27840221	28014920
XLOC_027164	Ca_v2.0_24636	Ca8_v2.0	27840221	28014920
XLOC_027173	Ca_v2.0_24652	Ca8_v2.0	28149041	28149684
XLOC_027174	Ca_v2.0_24654	Ca8_v2.0	28193103	28194322
XLOC_027179	Ca_v2.0_24661	Ca8_v2.0	28231147	28233774
XLOC_027187	Ca_v2.0_24672	Ca8_v2.0	28344814	28347986
XLOC_027188	Ca_v2.0_24673	Ca8_v2.0	28348755	28353814
XLOC_027189	Ca_v2.0_24675	Ca8_v2.0	28362726	28366728
XLOC_027210	-	Ca8_v2.0	1837381	1838042
XLOC_027215	-	Ca8_v2.0	3357047	3357274
XLOC_027217	-	Ca8_v2.0	3890864	3891951
XLOC_027218	-	Ca8_v2.0	4221380	4221686
XLOC_027220	-	Ca8_v2.0	4683031	4984382
XLOC_027221	-	Ca8_v2.0	4683031	4984382
XLOC_027232	-	Ca8_v2.0	8953210	8953617
XLOC_027236	-	Ca8_v2.0	11583684	11584919
XLOC_027241	-	Ca8_v2.0	13463936	13464173

For Review Only

XLOC_027243	-	Ca8_v2.0	14278199	14278468
XLOC_027253	-	Ca8_v2.0	21317194	21317602
XLOC_027265	-	Ca8_v2.0	25556980	25558163
XLOC_027269	-	Ca8_v2.0	26366295	26366725
XLOC_027272	-	Ca8_v2.0	27552982	27554140
XLOC_027275	-	Ca8_v2.0	27767546	27768534
XLOC_027279	-	Ca_scaffold_141	10	533
XLOC_027291	-	Ca_scaffold_193	1935	3416
XLOC_027298	Ca_v2.0_24739	Ca_scaffold_230	46	2587
XLOC_027308	-	Ca_scaffold_250	5432	6394
XLOC_027312	-	Ca_scaffold_282	0	320
XLOC_027313	-	Ca_scaffold_283	1324	2278
XLOC_027314	-	Ca_scaffold_293	117	1052
XLOC_027315	-	Ca_scaffold_297	908	1653
XLOC_027320	Ca_v2.0_24716	Ca_scaffold_335	3784	4268
XLOC_027326	Ca_v2.0_24757	Ca_scaffold_362	4932	9929
XLOC_027328	Ca_v2.0_24759	Ca_scaffold_382	1551	4909
XLOC_027329	-	Ca_scaffold_388	965	1313
XLOC_027330	Ca_v2.0_24760	Ca_scaffold_394	8120	8692
XLOC_027336	Ca_v2.0_24765	Ca_scaffold_406	749	1451
XLOC_027362	Ca_v2.0_24782	Ca_scaffold_507	376	655
XLOC_027364	Ca_v2.0_24784	Ca_scaffold_508	7943	11683
XLOC_027365	-	Ca_scaffold_509	7	1007
XLOC_027371	-	Ca_scaffold_513	0	348
XLOC_027372	-	Ca_scaffold_513	69	568
XLOC_027375	-	Ca_scaffold_515	422	629
XLOC_027377	Ca_v2.0_24791	Ca_scaffold_516	411	754
XLOC_027388	Ca_v2.0_24800	Ca_scaffold_521	13	1174
XLOC_027390	Ca_v2.0_24802	Ca_scaffold_522	152	1164
XLOC_027399	-	Ca_scaffold_527	389	1256
XLOC_027403	-	Ca_scaffold_529	0	1297
XLOC_027414	Ca_v2.0_24816	Ca_scaffold_539	280	1392
XLOC_027415	Ca_v2.0_24816	Ca_scaffold_539	280	1392
XLOC_027426	Ca_v2.0_24825	Ca_scaffold_544	3	1295
XLOC_027429	Ca_v2.0_24828	Ca_scaffold_544	1	1663
XLOC_027434	-	Ca_scaffold_548	651	1601
XLOC_027437	-	Ca_scaffold_549	1533	2123
XLOC_027443	Ca_v2.0_24724	Ca_scaffold_550	12062	13795
XLOC_027444	Ca_v2.0_24724	Ca_scaffold_550	12062	13795
XLOC_027448	Ca_v2.0_24837	Ca_scaffold_551	5	2126
XLOC_027470	Ca_v2.0_24855	Ca_scaffold_562	2616	3624
XLOC_027483	Ca_v2.0_24867	Ca_scaffold_565	230	3453
XLOC_027495	Ca_v2.0_24878	Ca_scaffold_568	1900	3970
XLOC_027502	Ca_v2.0_24885	Ca_scaffold_569	105	3605
XLOC_027503	Ca_v2.0_24886	Ca_scaffold_570	2346	5263
XLOC_027508	Ca_v2.0_24891	Ca_scaffold_570	0	2843

For Review Only

XLOC_027525	Ca_v2.0_24909	Ca_scaffold_572	151	5595
XLOC_027540	Ca_v2.0_24922	Ca_scaffold_574	1455	8913
XLOC_027541	Ca_v2.0_24923	Ca_scaffold_574	2479	8871
XLOC_027547	Ca_v2.0_24929	Ca_scaffold_575	5744	10208
XLOC_027548	Ca_v2.0_24930	Ca_scaffold_575	675	2960
XLOC_027549	Ca_v2.0_24931	Ca_scaffold_575	8135	11595
XLOC_027560	Ca_v2.0_24942	Ca_scaffold_576	4183	4948
XLOC_027570	Ca_v2.0_24954,Ca_v2.0_249	Ca_scaffold_576	14921	24265
XLOC_027572	Ca_v2.0_24965,Ca_v2.0_249	Ca_scaffold_576	54764	61964
XLOC_027580	Ca_v2.0_24955	Ca_scaffold_576	14921	24265
XLOC_027583	Ca_v2.0_24968,Ca_v2.0_249	Ca_scaffold_576	65327	77432
XLOC_027588	Ca_v2.0_24981	Ca_scaffold_576	97199	97648
XLOC_027593	Ca_v2.0_24992	Ca_scaffold_577	19266	20134
XLOC_027604	-	Ca_scaffold_578	212978	213188
XLOC_027608	Ca_v2.0_25002,Ca_v2.0_250	Ca_scaffold_578	27017	34064
XLOC_027620	-	Ca_scaffold_581	42336	42551
XLOC_027621	Ca_v2.0_25012	Ca_scaffold_582	436	20341
XLOC_027624	Ca_v2.0_25015	Ca_scaffold_583	21694	31445
XLOC_027629	Ca_v2.0_25018	Ca_scaffold_586	12644	14000
XLOC_027631	Ca_v2.0_25020	Ca_scaffold_586	12901	14757
XLOC_027648	Ca_v2.0_25035	Ca_scaffold_595	35050	35525
XLOC_027654	-	Ca_scaffold_599	13557	13818
XLOC_027663	Ca_v2.0_25048	Ca_scaffold_606	1462	2006
XLOC_027682	Ca_v2.0_25066	Ca_scaffold_612	39983	41694
XLOC_027684	-	Ca_scaffold_612	48013	49460
XLOC_027689	Ca_v2.0_25066	Ca_scaffold_612	39983	41694
XLOC_027706	Ca_v2.0_25082	Ca_scaffold_623	9209	11458
XLOC_027708	Ca_v2.0_25085	Ca_scaffold_623	16600	19382
XLOC_027709	Ca_v2.0_25084	Ca_scaffold_623	9091	9679
XLOC_027712	Ca_v2.0_25088	Ca_scaffold_624	277	3780
XLOC_027715	Ca_v2.0_25091	Ca_scaffold_631	311	479
XLOC_027720	Ca_v2.0_25096	Ca_scaffold_634	11200	11898
XLOC_027733	-	Ca_scaffold_692	1205	1500
XLOC_027736	Ca_v2.0_24727	Ca_scaffold_988	30	1646

For Review Only

ɔmosomal localization, and functional

Annotation
small acidic protein 1-like
---NA---
AES63246.2 T-complex protein
serine hydroxymethyltransferase 7
patatin-like phospholipase domain-containing protein 2
vacuolar iron transporter homolog 4
soyasaponin III rhamnosyltransferase
high affinity sulfate transporter 2-like isoform X1
chlorophyll a-b binding protein CP24 10A, chloroplastic
uncharacterized protein LOC101515692
protein EXORDIUM-like 2
bifunctional riboflavin biosynthesis protein RIBA 1, chloroplastic
classical arabinogalactan protein 1-like
---NA---
protein PELPK1-like
MADS-box transcription factor 23-like isoform X3
protein RMD5 homolog isoform X1
uncharacterized protein LOC101507431
transcription factor bHLH19-like
receptor-like protein kinase HAIKU2
basic form of pathogenesis-related protein 1-like
pathogenesis-related protein 1B-like
sulfite exporter TauE/SafE family protein 5-like
AES63635.1 C2H2-like zinc finger protein
uncharacterized protein LOC101514496
transcription factor bHLH112-like isoform X2
B-box domain protein 30-like
Equilibrative nucleoside transporter
KEH36510.1 receptor-interacting protein, putative
---NA---
transmembrane protein 184A
lysine histidine transporter-like 8
putative glycine-rich cell wall structural protein 1
putative disease resistance protein At3g14460
COP1-interacting protein 7
stem-specific protein TSJT1-like
clathrin light chain 1-like
uncharacterized protein LOC101492576
enoyl-CoA delta isomerase 2, peroxisomal-like
callose synthase 11-like
protein SMG7L isoform X2
dof zinc finger protein DOF5.3-like

For Review Only

squamosa promoter-binding-like protein 3
AES63882.1 nucleic acid-binding, OB-fold-like protein
dnaJ homolog subfamily B member 13-like
NAC domain-containing protein 73
probable leucine-rich repeat receptor-like protein kinase At5g49770
pathogenesis-related genes transcriptional activator PTI5-like
protein IQ-DOMAIN 1
cysteine protease
olee1-like protein
protein LIGHT-DEPENDENT SHORT HYPOCOTYLS 10-like
probable WRKY transcription factor 65
precursor of CEP14-like
U-box domain-containing protein 17
pyridoxal 5'-phosphate synthase subunit PDX1.3
AES64037.1 major intrinsic protein (MIP) family transporter
26S proteasome non-ATPase regulatory subunit 14 homolog
hypothetical protein DVH24_017397
integrin-linked protein kinase 1
KEH36680.1 senescence regulator
serine carboxypeptidase-like 11
uncharacterized protein LOC11443845 isoform X1
CSC1-like protein ERD4
transcription factor TCP2
AES64169.1 VQ motif protein
sugar transporter ERD6-like 16
LEC14B homolog
pyrimidine-specific ribonucleoside hydrolase rihA
uncharacterized protein LOC101505806
protein DMP2-like
transmembrane protein 45A-like
probable serine/threonine-protein kinase PBL23
dihydroxy-acid dehydratase, chloroplastic
basic 7S globulin
uncharacterized protein LOC101500992
adenylate isopentenyltransferase 5, chloroplastic-like
probable F-box protein At4g22030
B-cell receptor-associated protein 29-like
protein STRUBBELIG-RECEPTOR FAMILY 8
E3 ubiquitin-protein ligase RNF123
RPM1-interacting protein 4
EH domain-containing protein 2-like
ABD33010.1 Fumarylacetoacetase
BAG family molecular chaperone regulator 5, mitochondrial
CASP-like protein 2B1
nuclear transcription factor Y subunit B-3-like
14 kDa proline-rich protein DC2.15-like precursor

For Review Only

farnesyl pyrophosphate synthase 1

photosystem I reaction center subunit III, chloroplastic

uncharacterized protein LOC101507534

transmembrane protein 64

probable leucine-rich repeat receptor-like protein kinase At5g49770 isoform X1

coatomer subunit zeta-1-like

patatin-like protein 7

peroxidase

peroxidase

accelerated cell death 11-like

AES64849.1 transmembrane protein, putative

dihydroceramide fatty acyl 2-hydroxylase FAH1-like isoform X1

berberine bridge enzyme-like 21

wall-associated receptor kinase-like 8 isoform X1

UDP-arabinose 4-epimerase 1

transcription factor MYB108-like

F-box protein At5g49610 isoform X1

Ribosomal protein S6

cell wall / vacuolar inhibitor of fructosidase 1-like

GDSL esterase/lipase At4g16230-like isoform X2

transcription factor bHLH147-like

insoluble matrix shell protein 4-like

insoluble matrix shell protein 4-like

uncharacterized protein LOC101488230

glucan endo-1,3-beta-glucosidase precursor

beta-D-xylosidase 1

pathogenesis-related protein STH-2-like

pathogenesis-related protein STH-2-like

pathogenesis-related protein 1

peroxisomal fatty acid beta-oxidation multifunctional protein MFP2-like

protein SRC2-like

protein SRG1

fasciclin-like arabinogalactan protein 4

hypothetical protein MtrunA17_Chr2g0296631

isoflavone 3'-hydroxylase-like

mitochondrial amidoxime reducing component 2-like

ethylene-responsive transcription factor ERF096-like

PR5-like receptor kinase

plasma membrane ATPase 4

uncharacterized protein LOC101495820

AES65296.1 DUF4228 domain protein

protein CHLOROPLAST IMPORT APPARATUS 2-like

protein SMAX1-LIKE 4

plant intracellular Ras-group-related LRR protein 6-like

probable WRKY transcription factor 11

probable xyloglucan endotransglucosylase/hydrolase protein 23

For Review Only

---NA---
putative transcription factor bHLH041
IQ domain-containing protein IQM1
peroxidase N-like
protein ALP1-like
vacuolar protein sorting-associated protein 2 homolog 1
replication protein A 70 kDa DNA-binding subunit A
stress-response A/B barrel domain-containing protein UP3-like
MOB kinase activator 1A
tubulin--tyrosine ligase-like protein 12 isoform X2
---NA---
protein TIFY 10A-like
NADH-dependent glutamate synthase
ethylene-responsive transcription factor ERF017-like
guanosine deaminase-like isoform X1
uncharacterized protein LOC101493681
CBL-interacting serine/threonine-protein kinase 23 isoform X2
uncharacterized protein LOC101489452
mitochondrial import inner membrane translocase subunit TIM50
leucine-rich repeat receptor-like protein kinase TDR isoform X1
calmodulin-binding protein 60 E-like isoform X4
KEH37975.1 F-box protein
DELLA protein RHT-1-like
heme oxygenase 1, chloroplastic-like isoform X2
nudix hydrolase 19, chloroplastic
serine carboxypeptidase-like 45 isoform X2
uridine 5'-monophosphate synthase
---NA---
INO80 complex subunit C-like isoform X2
dof zinc finger protein DOF5.4-like
UDP-arabinopyranose mutase 3-like
---NA---
---NA---
auxin-responsive protein IAA30
KEH26102.1 plant/T8K14-16 protein
hypothetical protein VIGAN_07205400
cytochrome P450 CYP736A12-like
SKP1-like protein 14
SKP1-like protein 14
G-type lectin S-receptor-like serine/threonine-protein kinase RLK1 isoform X1
transcription factor JUNGBRUNNEN 1
cysteine-rich TM module stress tolerance protein
cysteine-rich and transmembrane domain-containing protein B-like
NAC domain-containing protein 21/22
cold-responsive protein kinase 1-like
serine/threonine-protein kinase WAG1

For Review Only

hypothetical protein C4D60_Mb01t29200

nicotianamine synthase-like

peroxisomal (S)-2-hydroxy-acid oxidase isoform X2

ruBisCO large subunit-binding protein subunit beta, chloroplastic

VQ motif-containing protein 1

putative callose synthase 8

---NA---

cytochrome P450 72A68-like

11-oxo-beta-amyrin 30-oxidase-like

fatty-acid-binding protein 3, chloroplastic

F-box/FBD/LRR-repeat protein At4g26340-like

probable sodium/metabolite cotransporter BASS1, chloroplastic

B-box zinc finger protein 22

geraniol 8-hydroxylase-like

lysosomal Pro-X carboxypeptidase

---NA---

WRKY DNA-binding transcription factor 70-like

protein GRAVITROPIC IN THE LIGHT 1-like

epidermis-specific secreted glycoprotein EP1

phosphoenolpyruvate carboxylase

AAA-ATPase At3g50940-like

transcription factor TCP4-like

QBM11794.1 SPOROCYTELESS-like EAR-containing protein

probable protein phosphatase 2C 39

protein DETOXIFICATION 19-like

proline-rich extensin-like protein EPR1

protein GRIM REAPER-like

defensin AFP precursor

vicilin-like seed storage protein At2g18540

stem-specific protein TSJT1

lanC-like protein GCR2

NAC family transcription factor 4

probable protein phosphatase 2C 12

receptor-like protein kinase THESEUS 1

AES66730.1 cotton fiber

unknown

transcription factor MYB41

---NA---

UDP-glycosyltransferase 79B30-like

protein UPSTREAM OF FLC-like isoform X2

transcription factor FER-LIKE IRON DEFICIENCY-INDUCED TRANSCRIPTION FACTOR-like isoform X1

zinc finger protein ZAT5-like

protein SENSITIVE TO PROTON RHIZOTOXICITY 2

aspartic proteinase-like protein 2

GDSL esterase/lipase At5g22810

arogenate dehydratase 3, chloroplastic-like

For Review Only

KEH38957.1 DUF868 family protein
serine/threonine-protein kinase D6PKL2
zinc finger A20 and AN1 domain-containing stress-associated protein 3
pentatricopeptide repeat-containing protein At5g48910-like
glucan endo-1,3-beta-glucosidase 14 isoform X1
neoxanthin synthase, chloroplastic-like
casparian strip membrane protein 4
F-box protein At2g27310
interactor of constitutive active ROPs 4
histone-lysine N-methyltransferase, H3 lysine-79 specific-like isoform X1
protein FAF-like, chloroplastic
dnaJ protein homolog
mitogen-activated protein kinase kinase kinase 18-like
---NA---
protein DETOXIFICATION 40-like
vascular-related unknown protein 1-like
---NA---
cysteine-rich repeat secretory protein 38-like
uclacyanin 1
probable terpene synthase 11
xyloglucan endotransglucosylase/hydrolase protein 9-like
hypothetical protein TSUD_105500
G-type lectin S-receptor-like serine/threonine-protein kinase
cytochrome b6-f complex iron-sulfur subunit, chloroplastic
KEH39042.1 wound-responsive family protein
KEH39052.1 wound-responsive family protein
uncharacterized protein LOC101509446
cold tolerant protein
cold tolerant protein
protein SYM1 isoform X3
ethylene-responsive transcription factor ERF054
early nodulin-like protein 2
probable serine/threonine-protein kinase At1g54610
probable aminotransferase TAT2
signaling peptide TAXIMIN 2-like
receptor-like protein kinase HSL1
ras-related protein RABA1f-like
protein LIGHT-DEPENDENT SHORT HYPOCOTYLS 10-like
protein NRT1/ PTR FAMILY 2.6-like
auxin response factor 4
putative Zinc finger, SWIM-type, interactor of constitutive active ROPs
protein disulfide-isomerase 5-1
beta-galactosidase 8-like
hypothetical protein MTR_2g095040
P-loop NTPase domain-containing protein LPA1 homolog 1-like isoform X1
dolichol kinase EVAN

For Review Only

uncharacterized protein LOC101493563
transcription factor MYB48-like
dof zinc finger protein DOF1.6-like isoform X1
---NA---
outer envelope protein 80, chloroplastic
zinc finger protein WIP2-like
beta-glucosidase 12-like
histidine methyltransferase-like protein
60S ribosomal protein L38-like
pyridoxal phosphate phosphatase phospho2
probable inactive ATP-dependent zinc metalloprotease FTSHI 2, chloroplastic
protein NRT1/ PTR FAMILY 1.2-like
homeobox-leucine zipper protein GLABRA 2
photosystem I reaction center subunit VI-2, chloroplastic
AES68001.1 late embryogenesis abundant protein
FCS-Like Zinc finger 10
coatomer subunit beta'-2-like
coatomer subunit beta'-2-like
D-glycerate 3-kinase, chloroplastic isoform X1
gibberellin 3-beta-dioxygenase 1
pleiotropic drug resistance protein 1-like
---NA---
AES68094.1 plant/F18G18-200 protein
acyl-protein thioesterase 2
---NA---
uncharacterized protein LOC101513413
transcription factor bHLH18-like isoform X2
cinnamoyl-CoA reductase 1
probable WRKY transcription factor 40
putative receptor-like protein kinase At1g80870
ent-kaurene oxidase, chloroplastic-like
ent-kaurene oxidase, chloroplastic-like
fasciclin-like arabinogalactan protein 14
receptor-like protein kinase
---NA---
kinesin-like protein KIN-5C
protein NRT1/ PTR FAMILY 4.3-like
ABE79561.1 Octicosapeptide/Phox/Bem1p
ferredoxin I
potassium channel AKT2/3 isoform X1
---NA---
WAT1-related protein At1g68170-like
uncharacterized protein LOC101500249
fructose-1,6-bisphosphatase, cytosolic
NAC domain-containing protein 104
soyasaponin III rhamnosyltransferase

For Review Only

beta-xylosidase/alpha-L-arabinofuranosidase 1 isoform X2
chaperone protein dnaJ 20, chloroplastic-like
non-specific lipid-transfer protein-like protein At5g64080
cationic peroxidase 2-like
probable inactive receptor kinase At2g26730
ribosome biogenesis regulatory protein homolog
metacaspase-3-like isoform X1
protein PELPK1-like
protein PELPK1-like
40S ribosomal protein S5-like
gamma-glutamyl peptidase 5-like
---NA---
uncharacterized protein LOC101495983 isoform X2
sulfite exporter TauE/SafE family protein 5-like
receptor-like serine/threonine-protein kinase SD1-8
transcription factor MYB41
KEH36507.1 wound-responsive family protein
secretory carrier-associated membrane protein-like
AES63728.2 lysine ketoglutarate reductase trans-splicing-like protein
polyphenol oxidase A1, chloroplastic-like
sphingolipid delta(4)-desaturase DES1-like
E3 ubiquitin-protein ligase AIRP2-like
probable polyol transporter 4 isoform X1
E3 ubiquitin-protein ligase RMA1H1-like
probable ubiquitin-conjugating enzyme E2 24
dof zinc finger protein DOF1.4
dormancy-associated protein 1-like
pinin isoform X1
ABN08731.1 Thioredoxin fold
glutamate receptor 2.7
phospholipase A1-II 1-like
protein trichome birefringence-like 38
putative transcription factor bHLH107
cyclic dof factor 4
probable phosphatidylinositol 4-phosphate 5-kinase MSS4
receptor-like protein 51
cellulose synthase A catalytic subunit 8 [UDP-forming]-like isoform X1
hypothetical protein GLYMA_15G084900
target of rapamycin complex subunit LST8
AES64152.1 transmembrane protein, putative
gibberellin 2-beta-dioxygenase-like
potassium channel KAT1-like
probable F-box protein At4g22030
cytochrome P450 710A11-like
serine/threonine-protein kinase RUNKEL
probable 1-deoxy-D-xylulose-5-phosphate synthase, chloroplastic

For Review Only

F-box/kelch-repeat protein At3g23880-like
uncharacterized protein LOC101488548
probable serine/threonine-protein kinase PBL23
adenosine deaminase-like protein
uncharacterized protein LOC101493769
ATP-dependent (S)-NAD(P)H-hydrate dehydratase
stem-specific protein TSJT1-like
basic 7S globulin-like
uncharacterized protein LOC101500677
KEH36766.1 RING zinc finger protein, putative
5'-adenylylsulfate reductase 3, chloroplastic
probable F-box protein At4g22030
putative petal formation-expressed
chitin elicitor receptor kinase 1-like
acyl-CoA--sterol O-acyltransferase 1
cytochrome b561 and DOMON domain-containing protein At4g17280-like
heterogeneous nuclear ribonucleoprotein 1
pollen-specific leucine-rich repeat extensin-like protein 1
BTB/POZ domain-containing protein At5g47800-like isoform X1
uncharacterized protein At4g26485-like isoform X3
---NA---
regulatory protein NPR3 isoform X1
peroxidase
berberine bridge enzyme-like 21
---NA---
berberine bridge enzyme-like 8
polygalacturonase At1g48100-like
uncharacterized protein LOC101495367 isoform X2
WRKY transcription factor 23-like
ATP-citrate synthase beta chain protein 1
NADH dehydrogenase [ubiquinone] 1 alpha subcomplex subunit 2-like
insoluble matrix shell protein 4-like
uncharacterized protein LOC101490977 isoform X2
hypothetical protein MtrunA17_Chr2g0294851
---NA---
protein LIR18B-like
probable polygalacturonase
uncharacterized protein LOC101504309
ABC transporter C family member 14-like
isoleucine--tRNA ligase, chloroplastic/mitochondrial
isoflavone 3'-hydroxylase-like
calmodulin-binding protein 25
transcription factor bHLH35 isoform X2
cytokinin dehydrogenase 3-like
diacylglycerol O-acyltransferase 1C-like isoform X2
RING-H2 finger protein ATL52-like

For Review Only

serine/threonine-protein kinase RIPK-like
---NA---
ethylene-responsive transcription factor ERF017-like
ethylene-responsive transcription factor ERF017-like
ethylene-responsive transcription factor ERF017-like
KEH37635.1 transmembrane protein, putative
RHN73746.1 putative transmembrane protein
---NA---
---NA---
uncharacterized protein LOC101488468
uncharacterized protein LOC101489465
pyruvate dehydrogenase (acetyl-transferring) kinase, mitochondrial-like
KEH37723.1 DUF538 family protein
S-adenosylmethionine synthase-like
subtilisin-like protease SBT2.2
UDP-glycosyltransferase 43
UDP-glycosyltransferase 43
RNA cytidine acetyltransferase 1 isoform X2
pentatricopeptide repeat-containing protein At1g03540
regulator of nonsense transcripts UPF2 isoform X2
BTB/POZ domain-containing protein At1g30440
CTD small phosphatase-like protein 2 isoform X1
protein MEI2-like 5 isoform X1
heparanase-like protein 3
uncharacterized protein LOC101489452
hypothetical protein E2542_SST19178
probable flavin-containing monooxygenase 1 isoform X1
uncharacterized protein LOC101509174
calmodulin-binding protein 60 E-like isoform X1
uncharacterized protein LOC101515377
serine carboxypeptidase-like 45 isoform X2
cucumber peeling cupredoxin-like
3-ketoacyl-CoA synthase 11-like
---NA---
2-alkenal reductase (NADP(+)-dependent)-like
F-box/FBD/LRR-repeat protein At3g14710-like isoform X1
transmembrane protein, putative
uridine 5'-monophosphate synthase
---NA---
flavonol 3-O-glucosyltransferase UGT89B1
---NA---
UDP-arabinopyranose mutase 3-like
cytochrome P450 714A2-like isoform X2
VQ motif-containing protein 25-like
KEH38183.1 transmembrane protein, putative
uncharacterized protein LOC101492031

For Review Only

AES66081.2 DUF966 domain protein
ATP-citrate synthase alpha chain protein 2-like
peroxidase 7-like
transcription factor MYB41 isoform X1
putative vacuolar protein sorting-associated protein
zinc transporter 1
plant/F3O9-12 protein
kinase TKL-Pl-6 family
auxin-responsive protein SAUR72
LOB domain-containing protein 4
nitrile-specifier protein 5-like
elongation factor 2
rho GTPase-activating protein gacF
photosystem II 10 kDa polypeptide, chloroplastic
probable glutathione S-transferase
ubiquitin carboxyl-terminal hydrolase 25 isoform X2
9-cis-epoxycarotenoid dioxygenase
gibberellin 2-beta-dioxygenase 1 isoform X1
---NA---
uncharacterized protein LOC101496473
uncharacterized protein LOC101496808
pentatricopeptide repeat-containing protein mitochondrial-like
---NA---
---NA---
chitinase 2-like
---NA---
ribosomal protein S4 (mitochondrion)
protein SRG1-like
AES60756.1 albumin-2 protein
alpha-amylase/subtilisin inhibitor-like
bidirectional sugar transporter SWEET2
protein trichome birefringence-like 41
BES1/BZR1 homolog protein 4-like
hypothetical protein MTR_2g075490
hypothetical protein MTR_2g075520
hypothetical protein MTR_2g075490
---NA---
protein LIGHT-DEPENDENT SHORT HYPOCOTYLS 10-like
phosphoenolpyruvate carboxylase
increased DNA methylation 1
putative nucleolar GTP-binding protein
sodium/calcium exchanger NCL
uncharacterized protein LOC101512525
chilling-induced protein
---NA---
CBS domain-containing protein CBSX5

For Review Only

stem-specific protein TSJT1

---NA---

KEH38800.1 S-adenosylmethionine-dependent methyltransferase, putative

probable protein phosphatase 2C 12

KEH38807.1 root cap/late embryogenesis-like protein

uncharacterized protein LOC101502389

chlorophyll a-b binding protein 13, chloroplastic

KEH38819.1 wound-responsive family protein

uncharacterized protein LOC101497455

transcription factor bHLH123-like isoform X4

scarecrow-like protein 21

protein NDR1-like

vascular-related unknown protein 4-like isoform X1

UDP-glycosyltransferase 79B30-like

UDP-glycosyltransferase 79B30

UDP-glycosyltransferase 79B30

peroxidase P7

probable serine/threonine-protein kinase PBL7

aspartic proteinase-like protein 2

arogenate dehydratase 3, chloroplastic-like

---NA---

nucleobase-ascorbate transporter 12-like

stress-response A/B barrel domain-containing protein At5g22580

cytochrome P450 94C1-like

transcription factor TGA9-like isoform X2

---NA---

receptor protein kinase TMK1-like isoform X1

E3 ubiquitin-protein ligase PUB23-like

core-2 i-branching beta-n-acetylglucosaminyltransferase family protein

B-cell receptor-associated protein 31

L-ascorbate oxidase homolog

---NA---

rhomboid family member

hypothetical protein TEA_005098

---NA---

cysteine-rich repeat secretory protein 38-like

transcription factor bHLH115-like isoform X1

neural Wiskott-Aldrich syndrome protein

calcium-binding protein PBP1-like

uncharacterized protein LOC101504633

probable protein phosphatase 2C 52

isoform X1

early nodulin-like protein 2

zinc finger protein MAGPIE

monothiol glutaredoxin-S9

fasciclin-like arabinogalactan protein 13

For Review Only

RING-H2 finger protein ATL40-like
transcription factor TCP4-like
protein trichome birefringence-like 39
bifunctional riboflavin kinase/FMN phosphatase-like
heavy metal-associated isoprenylated plant protein 28
phosphoenolpyruvate carboxylase 2-like
auxin response factor 4
fasciclin-like arabinogalactan protein 12
---NA---
UDP-xylose transporter 1-like
SNF1-related protein kinase regulatory subunit beta-3
ruBisCO-associated protein-like
exocyst complex component EXO70H1
AES67575.1 DUF1635 family protein
transcription factor MYB48-like
LOW QUALITY PROTEIN: uncharacterized protein LOC101504850
KEH39447.1 expansin A10
AES67633.1 haloacid dehalogenase-like hydrolase
hypothetical protein MtrDRAFT_AC157503g25v2
extensin-2-like
ADP,ATP carrier protein, mitochondrial-like
hypothetical protein L195_g032453
DNA damage-repair/toleration protein DRT100
KEH39487.1 DUF936 family protein
endochitinase PR4
beta-fructofuranosidase, cell wall isozyme-like
protein indeterminate-domain 7-like
inorganic pyrophosphatase 2-like
uncharacterized protein LOC101502273
IAA-amino acid hydrolase ILR1-like 4
auxin-responsive protein IAA26-like
clavaminate synthase-like protein At3g21360
histidine-containing phosphotransfer protein 4
ABC transporter G family member 36
uncharacterized protein LOC101493777
auxin-responsive protein IAA27-like isoform X1
probable dynein light chain
probable sulfate transporter 3.4
pleiotropic drug resistance protein 1-like
ABD28387.1 Asp/Glu racemase
nuclear pore complex protein NUP93A-like
uncharacterized protein LOC101490521
probable polygalacturonase At3g15720
embryo-specific protein ATS3A-like
probable serine/threonine-protein kinase fhkB
uncharacterized protein LOC101500888

For Review Only

zinc finger protein 1-like
CBL-interacting serine/threonine-protein kinase 25-like
tubby-like protein 8
fasciclin-like arabinogalactan protein 14
---NA---
---NA---
transmembrane protein, putative
hypothetical protein MtrunA17_Chr2g0290401
hypothetical protein MtrunA17_Chr2g0295461
hypothetical protein TSUD_289410
---NA---
---NA---
---NA---
---NA---
---NA---
---NA---
---NA---
wound-responsive family protein
---NA---
lisH domain and HEAT repeat-containing protein KIAA1468 isoform X2
alkane hydroxylase MAH1-like
putative germin-like protein 2-1
probable phospholipid-transporting ATPase 8
2-methylene-furan-3-one reductase-like
potassium transporter 8-like isoform X4
alpha-dioxygenase 1
KEH24902.1 BAH domain-containing protein
---NA---
xyloglucan glycosyltransferase 4
gibberellin-regulated protein 14
senescence-associated carboxylesterase 101 isoform X1
peroxidase 63-like protein
zinc finger AN1 domain-containing stress-associated protein 12
cytochrome P450 82A1-like
AAA-ATPase ASD, mitochondrial
AAA-ATPase ASD, mitochondrial
F-box/WD repeat-containing protein 7-like
gibberellin-regulated protein 4-like
granule-bound starch synthase 2, chloroplastic/amyloplastic
transcription factor MYB106
KEH25033.1 plant calmodulin-binding-like protein
7-deoxyloganetin glucosyltransferase
7-deoxyloganetin glucosyltransferase
alpha-1,4 glucan phosphorylase L isozyme, chloroplastic/amyloplastic
transcription factor UPBEAT1
protein SPIRAL1-like 2

For Review Only

TMV resistance protein N-like

TMV resistance protein N isoform X1

malonyl-coenzyme A:anthocyanin 3-O-glucoside-6''-O-malonyltransferase-like

proline-rich protein 4

putative non-specific serine/threonine protein kinase

basic leucine zipper 43-like

proline dehydrogenase 2, mitochondrial-like

KEH25243.1 DUF1262 family protein

probable endo-1,3(4)-beta-glucanase ARB_01444

protein ABA DEFICIENT 4, chloroplastic-like isoform X2

uncharacterized protein LOC101503941 isoform X1

uncharacterized protein LOC101511386

---NA---

hypothetical protein TSUD_277870

class I heat shock protein-like

death-associated protein kinase dapk-1

lysine histidine transporter 1-like

lysine histidine transporter 1-like isoform X2

uncharacterized protein At3g27210-like

F-box/kelch-repeat protein At3g27150

hypothetical protein MTR_6g026820

uncharacterized protein LOC112422581

protein SRC1

ethylene-responsive transcription factor RAP2-11

LOW QUALITY PROTEIN: uncharacterized protein LOC101508606

probable glutamate dehydrogenase 3

glucan endo-1,3-beta-glucosidase 8

ethylene-responsive transcription factor ERF110-like

ruBisCO-associated protein-like

ruBisCO-associated protein-like

spermidine synthase 1

calcium-dependent protein kinase 24-like

AES96227.2 NHL repeat protein

WAT1-related protein At3g28070-like isoform X2

uncharacterized protein LOC101515721

uncharacterized protein LOC101513818

probable galacturonosyltransferase-like 9

glycosyltransferase family 92 protein RCOM_0530710

transcription factor MYB1R1-like

AES96473.2 actin cross-linking protein

---NA---

UDP-galactose/UDP-glucose transporter 3

F-box/kelch-repeat protein At1g26930-like

uncharacterized protein LOC101512365

ribonuclease 1-like precursor

ribonuclease 3-like

For Review Only

casparian strip membrane protein 1
uncharacterized endoplasmic reticulum membrane protein C16E8.02-like
protein argonaute 7
AES96945.2 DUF863 family protein
---NA---
probable leucine-rich repeat receptor-like protein kinase At1g35710
remorin isoform X2
beta-glucosidase 24-like
ureide permease 1
pentatricopeptide repeat-containing protein At5g50280, chloroplastic
hypothetical protein TSUD_219230
cytochrome P450 78A5
UDP-glycosyltransferase 73D1
probable UDP-arabinopyranose mutase 1
salicylate carboxymethyltransferase
---NA---
universal stress protein PHOS32
probable terpene synthase 2
heat shock protein 90-5, chloroplastic
protein SRG1-like isoform X3
protein SRG1-like isoform X1
probable indole-3-acetic acid-amido synthetase GH3.1
hypothetical protein GLYMA_01G069000
basic leucine zipper 43
---NA---
F-box/FBD/LRR-repeat protein At1g13570-like
protein NSP-INTERACTING KINASE 2 isoform X2
S-adenosylmethionine decarboxylase proenzyme
probable transmembrane ascorbate ferrireductase 4
allene oxide cyclase, chloroplastic-like
RHN55963.1 putative nucleic acid-binding protein
nodulation-signaling pathway 2 protein-like
alpha-xylosidase 1-like
endonuclease 2
zinc finger protein GIS3-like
AES97518.1 core-2/I-branching enzyme
heavy metal-associated isoprenylated plant protein 20-like
lysine histidine transporter-like 6 isoform X2
mitogen-activated protein kinase kinase kinase 1-like
nitrate reductase [NADH]-like
---NA---
sulfate transporter 1.3
low affinity sulfate transporter 3-like
uncharacterized protein LOC113848277
AT-rich interactive domain-containing protein 6-like
hypothetical protein TSUD_219090

For Review Only

uncharacterized protein LOC113848277
probable trehalose-phosphate phosphatase C
---NA---
protein spinster homolog 3-like
hypothetical protein GW17_00051536
protein EXORDIUM-like
uncharacterized protein LOC101491122
---NA---
---NA---
protein DMR6-LIKE OXYGENASE 2-like
uncharacterized protein LOC101504435
MADS-box transcription factor 23-like isoform X3
AES98345.1 transmembrane protein, putative
---NA---
---NA---
cucumisin-like
uncharacterized protein LOC101505835
putative bark agglutinin LECRPA3
heavy metal-associated isoprenylated plant protein 9-like
zinc finger protein CONSTANS-LIKE 9
cytochrome P450 86A1
protein SMAX1-LIKE 6-like
probable protein phosphatase 2C 25
protein LIGHT-DEPENDENT SHORT HYPOCOTYLS 6-like
serine carboxypeptidase-like 42 isoform X2
UDP-glycosyltransferase 87A1
UDP-glycosyltransferase 87A1
cytochrome P450 83B1
AES98854.2 exocyst subunit exo70 family protein
uncharacterized protein LOC101504436
probable O-methyltransferase 3
probable O-methyltransferase 3
---NA---
peroxidase 4-like
CBL-interacting serine/threonine-protein kinase 2-like
transcription repressor OFP4
kinesin-like protein KIN-14N isoform X1
root phototropism protein 2-like
KH domain-containing protein At2g38610 isoform X3
---NA---
NDR1/HIN1-like protein 6
probable acyl-activating enzyme 6
protein SET DOMAIN GROUP 40 isoform X2
isocitrate dehydrogenase [NADP], chloroplastic
---NA---
protein OPI10 homolog

For Review Only

receptor protein kinase TMK1
---NA---
calcium-binding protein CML24
uncharacterized protein LOC101496603
HMG-Y-related protein A-like
protein RSI-1
GDSL esterase/lipase At5g37690
AES99342.1 plastid movement impaired protein
transmembrane protein, putative
AT-hook motif nuclear-localized protein 15-like
ribulose bisphosphate carboxylase/oxygenase activase, chloroplastic
glycerol-3-phosphate acyltransferase 5
---NA---
QAX32714.1 leghemoglobin
folylpolyglutamate synthase
uncharacterized protein LOC101499991
---NA---
probable sugar phosphate/phosphate translocator At3g11320
RHN57075.1 putative laccase
uncharacterized acetyltransferase At3g50280-like
phosphoinositide phospholipase C 2-like
zinc finger CCCH domain-containing protein 29
probable methyltransferase PMT23
protein trichome birefringence-like 33
---NA---
laccase 90a
staphylococcal-like nuclease CAN2
amino acid transporter AVT6C-like
peroxidase P7
WEB family protein At2g40480-like
protein transport protein SEC61 subunit gamma
probable tyrosine-protein phosphatase DSP4
protein LYK5
protein NRT1/ PTR FAMILY 6.4
putative phosphatidylglycerol/phosphatidylinositol transfer protein DDB_G0282179
receptor-like protein EIX1 isoform X2
lysM domain receptor-like kinase 3 isoform X2
putative LysM receptor kinase K1A
22.7 kDa class IV heat shock protein
probable serine/threonine-protein kinase PBL3
uncharacterized protein LOC101492272
blue copper protein
protein CLT3, chloroplastic isoform X2
---NA---
protein PATRONUS 2
oxygen-evolving enhancer protein 2, chloroplastic-like

For Review Only

glutathione S-transferase F9-like
transcription factor JUNGBRUNNEN 1
4-hydroxyphenylpyruvate dioxygenase
uncharacterized protein LOC101508299 isoform X1
probable LRR receptor-like serine/threonine-protein kinase At1g07650 isoform X2
---NA---
chloroplastic lipocalin isoform X1
AET00448.1 plant/F10M23-360 protein
probable disease resistance protein At1g52660
calcium-dependent protein kinase 2-like
GDSL esterase/lipase At5g55050-like
AET00563.1 DUF4408 domain protein
pentatricopeptide repeat-containing protein At4g39952, mitochondrial
glycerophosphodiester phosphodiesterase GDPDL3-like
glycerophosphodiester phosphodiesterase GDPDL4-like
AET00645.1 calcium-dependent lipid-binding (CaLB domain) family protein
heavy metal-associated isoprenylated plant protein 47-like isoform X2
two-pore potassium channel 1
uncharacterized protein LOC101512599
golgin subfamily A member 6-like protein 22
protein TIC 20-v, chloroplastic
AET00794.1 DUF2775 family protein
protein transport protein SFT2-like
LRR receptor-like serine/threonine-protein kinase
fructose-bisphosphate aldolase, cytoplasmic isozyme 1
ADP-glucose pyrophosphorylase small subunit CagpS2
transcriptional regulator STERILE APETALA
---NA---
chlorophyll a-b binding protein 215, chloroplastic
glucan endo-1,3-beta-glucosidase 4
nudix hydrolase 7-like
pyruvate kinase, cytosolic isozyme
glucuronokinase 1
galactinol--sucrose galactosyltransferase
Heterogeneous nuclear ribonucleoprotein 1
putative germin-like protein 2-1
uncharacterized protein LOC101496036
cinnamoyl-CoA reductase-like SNL6
potassium transporter 8-like isoform X2
protein NRT1/ PTR FAMILY 5.8-like
probable galacturonosyltransferase-like 9
ABC transporter B family member 15-like
type IV inositol polyphosphate 5-phosphatase 7-like
glucose-6-phosphate isomerase, cytosolic
CLAVATA3/ESR (CLE)-related protein 25
AAA-ATPase ASD, mitochondrial

For Review Only

homeobox-leucine zipper protein HAT7-like isoform X1
ABC transporter B family member 19
zinc-finger homeodomain protein 9-like
uncharacterized protein At5g19025
uncharacterized protein At5g39570
purple acid phosphatase 23
lamin-like protein
7-deoxyloganetin glucosyltransferase
---NA---
Calcium/calmodulin-regulated receptor-like kinase 2
ubinuclein-1-like isoform X1
E3 ubiquitin-protein ligase cip8-like protein
putative calcium-transporting ATPase 13, plasma membrane-type
protein NSP-INTERACTING KINASE 1-like
proline dehydrogenase 2, mitochondrial-like
protein trichome birefringence-like 19
protein trichome birefringence-like 19
probable endo-1,3(4)-beta-glucanase ARB_01444
KEH24966.1 RNase P Rpr2/Rpp21 subunit domain protein
LOB domain-containing protein 41-like isoform X1
probable pectate lyase 5
uncharacterized protein LOC101497905
short-chain alcohol dehydrogenase
F-box/kelch-repeat protein At3g27150-like
DNA-directed RNA polymerase II subunit RPB7
cyclic dof factor 1-like
transcription factor IIIB 90 kDa subunit-like isoform X2
---NA---
AES75184.1 methyltransferase-like protein
glucan endo-1,3-beta-glucosidase 8
myb-related protein 2-like isoform X1
OTU domain-containing protein DDB_G0284757 isoform X1
probable magnesium transporter NIPA8
UDP-glycosyltransferase 74G1
type IV inositol polyphosphate 5-phosphatase 3-like isoform X2
protein ALTERED XYLOGLUCAN 4-like
protein ALTERED XYLOGLUCAN 4
zinc finger protein 5
metalloendoproteinase 2-MMP
probable galacturonosyltransferase-like 9
protein COFACTOR ASSEMBLY OF COMPLEX C SUBUNIT B CCB4, chloroplastic
phospholipid-transporting ATPase 3-like
TMV resistance protein N-like
TMV resistance protein N-like
toll/interleukin-1 receptor-like protein
thioredoxin H2

For Review Only

AES96549.1 plant/T7N9-9 protein

probable serine/threonine-protein kinase PBL3

homeobox-leucine zipper protein ATHB-13

homeobox-leucine zipper protein ATHB-13

transcription factor PRE6

myb family transcription factor PHL8-like isoform X1

alpha-expansin 3 precursor

NAC family transcription factor 3

uncharacterized protein At5g01610

probable WRKY transcription factor 57

glycine-rich cell wall structural protein 1.8-like

---NA---

anthranilate synthase alpha subunit 1, chloroplastic-like

ninja-family protein AFP1

AES97085.2 plant/T31B5-30 protein

protein BIG GRAIN 1-like E

clavaminate synthase-like protein At3g21360

phosphatidylinositol 4-kinase gamma 5-like

heat shock protein 90-5, chloroplastic

protein SRG1-like

protein NSP-INTERACTING KINASE 2

KEH26721.1 DUF1262 family protein

zinc finger, C3HC4 type (RING finger) protein

allene oxide cyclase, chloroplastic-like

AP2/ERF and B3 domain-containing transcription factor RAV1-like

ethylene-responsive transcription factor ERF014-like

alpha-xylosidase 1-like

---NA---

pathogenesis-related protein 5

nudix hydrolase 1

---NA---

---NA---

sulfate transporter 1.3

ethylene-responsive transcription factor RAP2-4-like

RING-H2 finger protein ATL47-like

protein EXORDIUM-like

esterase AGAP003155

transport inhibitor response 1-like protein

UDP-glucose flavonoid 3-O-glucosyltransferase 6

beta carbonic anhydrase 5, chloroplastic isoform X4

transcription factor bHLH30-like

kinesin-like protein KIN-4C isoform X1

uncharacterized protein LOC101504435

PB1 domain protein

phosphoglycerate mutase-like protein 1

protein DETOXIFICATION 48-like

For Review Only

protein ENHANCED DISEASE RESISTANCE 4-like
cucumisin-like
QCD78030.1 Thylakoid soluble phosphoprotein TSP9
probable NAD(P)H dehydrogenase (quinone) FQR1-like 3 isoform X2
elongator complex protein 5 isoform X1
U11/U12 small nuclear ribonucleoprotein 31 kDa protein
zinc transporter 6, chloroplastic
cytochrome P450 83B1
Calmodulin-binding transcription activator 5
GDSL esterase/lipase At2g30310-like
WRKY transcription factor WRKY24
senescence associated gene 20-like
protein LURP-one-related 7 isoform X1
cationic peroxidase 1-like
LOB domain-containing protein 13 isoform X2
EPIDERMAL PATTERNING FACTOR-like protein 5
protein GRAVITROPIC IN THE LIGHT 1
XP_024639442.1 expansin-A8
uncharacterized protein LOC101507870
protein NETWORKED 4A-like
probable LRR receptor-like serine/threonine-protein kinase At1g06840
metal tolerance protein 4-like
KH domain-containing protein At3g08620-like isoform X1
NDR1/HIN1-like protein 6
phytoene synthase 2, chloroplastic isoform X1
protein REVEILLE 2 isoform X1
15.7 kDa heat shock protein, peroxisomal isoform X1
aspartic proteinase PCS1-like
U-box domain-containing protein 21-like
phosphatidylinositol 4-kinase beta 1-like isoform X3
AES99341.1 transmembrane protein, putative
16 kDa phloem protein 2-like
receptor-like cytoplasmic kinase 176
protein AAR2 homolog isoform X1
uncharacterized protein LOC101500940 isoform X2
---NA---
microtubule-associated protein RP/EB family member 1C-like
---NA---
17.5 kDa class I heat shock protein-like
AES99497.1 RING/FYVE/PHD zinc finger protein
AES99515.1 acid phosphatase-like protein
probable signal peptidase complex subunit 2
AES99537.1 fasciclin domain protein
RHN57099.1 putative tetratricopeptide-like helical domain-containing protein
nematode resistance protein-like HSPRO2
probable zinc metalloprotease EGY2, chloroplastic isoform X2

For Review Only

phosphoinositide phospholipase C 6-like isoform X1
phosphoinositide phospholipase C 6-like isoform X2
tRNA threonylcarbamoyladenosine dehydratase 2-like
probable 2-oxoglutarate-dependent dioxygenase At5g05600
ferritin-4, chloroplastic
abscisic acid receptor PYL4-like
dehydration-responsive element-binding protein 2C
heavy metal-associated isoprenylated plant protein 33
uncharacterized protein LOC101494235
probable leucine-rich repeat receptor-like serine/threonine-protein kinase At3g14840
ribonuclease TUDOR 1-like
uncharacterized protein LOC113786764
uncharacterized protein LOC101504124
glycine-rich protein 2 precursor
glycine-rich protein 1 precursor
TMV resistance protein N-like
protein NRT1/ PTR FAMILY 6.4
receptor-like protein EIX2 isoform X1
---NA---
NDR1/HIN1-like protein 13
hypothetical protein MTR_5g089380
CBL-interacting serine/threonine-protein kinase 3-like
AET00142.2 ubiquitin-associated/TS-N domain protein, putative
hypothetical protein DEO72_LG3g2861
AET00234.1 DUF4228 domain protein
---NA---
anthocyanidin 3-O-glucosyltransferase 2
pentatricopeptide repeat-containing protein At2g42920, chloroplastic
probable LRR receptor-like serine/threonine-protein kinase At1g07650 isoform X3
cytochrome P450 716B1-like
protein FATTY ACID EXPORT 5
Bromodomain and PHD finger-containing protein 3
DNA mismatch repair protein MSH5 isoform X1
cytochrome b561 and DOMON domain-containing protein At3g25290-like
putative BPI/LBP family protein At1g04970
syntaxin-52-like isoform X1
probable long-chain-alcohol O-fatty-acyltransferase 5
kinesin-like protein KIN-12F
AET00645.1 calcium-dependent lipid-binding (CaLB domain) family protein
probable serine/threonine-protein kinase WNK11
scarecrow-like protein 21
AET00668.1 transmembrane protein, putative
LRR receptor-like serine/threonine-protein kinase
cyclin-dependent protein kinase inhibitor SMR13-like
calcium-transporting ATPase 12, plasma membrane-type
uncharacterized protein LOC101505296

For Review Only

heat shock protein 90-6, mitochondrial
WRKY transcription factor 1
cytochrome b561 and DOMON domain-containing protein At2g04850-like
uncharacterized protein LOC101514326
acetyl-CoA acetyltransferase, cytosolic 1
fasciclin-like arabinogalactan protein 2
uncharacterized protein LOC101492055
chlorophyll a-b binding protein 3, chloroplastic
putative UDP-glucose flavonoid 3-O-glucosyltransferase 3
UDP-glycosyltransferase 82A1
uncharacterized protein LOC101499844
---NA---
hypothetical protein glysoja_033381
uncharacterized protein LOC101492811
hypothetical protein EJD97_025590
---NA---
---NA---
---NA---
---NA---
RNA polymerase beta subunit
hypothetical protein MtrunA17_Chr1g0154861
IDA 1
hypothetical protein TSUD_219090
hypothetical protein MtrunA17_Chr5g0426211
uncharacterized protein LOC101507869
hypothetical protein MtrunA17_Chr5g0429141
---NA---
---NA---
---NA---
NKAP family protein-like
1-aminocyclopropane-1-carboxylate oxidase isoform X1
Clavata3/ESR (CLE) gene family member MtCLE17
---NA---
olee1-like protein
bidirectional sugar transporter N3-like
bidirectional sugar transporter N3
heparan-alpha-glucosaminide N-acetyltransferase
KYP59477.1 IST1-like protein
cytokinin hydroxylase
LOB domain-containing protein 41-like
ribulose bisphosphate carboxylase small chain 3A, chloroplastic
ribulose bisphosphate carboxylase small chain 3A, chloroplastic
uncharacterized protein LOC101489357
myb-related protein 306-like
gibberellin-regulated protein 4
amino acid transporter AVT1I isoform X1

For Review Only

cyclic dof factor 2

cytochrome P450 71A1-like

cystathionine gamma-synthase, chloroplastic-like

GMP synthase

isoflavone-7-O-methyltransferase 9

phenolic glucoside malonyltransferase 1

hypothetical protein CR513_42363, partial

protein DOWNY MILDEW RESISTANCE 6-like

AES77743.2 DSBA oxidoreductase family protein

WPP domain-interacting tail-anchored protein 1

---NA---

KEH21810.1 LuxR family transcriptional regulator, putative

ethylene-responsive transcription factor ERF113

uncharacterized protein LOC101497365

60S acidic ribosomal protein p0-like

heat shock cognate 70 kDa protein 2-like

CASP-like protein 4D1

transcription initiation factor TFIID subunit 6

heat shock cognate 70 kDa protein-like

heat shock cognate 70 kDa protein-like

glucan endo-1,3-beta-glucosidase

---NA---

peroxidase 55

---NA---

hypothetical protein TSUD_166290

---NA---

ACT domain-containing protein ACR6

germin-like protein subfamily 1 member 17

germin-like protein subfamily 1 member 20

18.2 kDa class I heat shock protein

probable adenylate kinase 7, mitochondrial

ABC transporter B family member 26, chloroplastic-like isoform X2

lysine histidine transporter 1-like

---NA---

kelch repeat-containing protein At3g27220-like isoform X1

zinc finger protein CONSTANS-LIKE 8

KEH41462.1 Hop-interacting protein THI031, putative

2-methylene-furan-3-one reductase-like

---NA---

DNA mismatch repair protein MSH1, mitochondrial-like

uncharacterized protein LOC101507264

putative viral movement protein

---NA---

UDP-glycosyltransferase 73C3

RHN45458.1 putative cyclin

uncharacterized protein LOC101489905 isoform X1

For Review Only

pectin methylesterase
uncharacterized protein LOC101495409
cytochrome b561 and DOMON domain-containing protein At3g25290 isoform X1
U-box domain-containing protein 9-like
---NA---
transcription factor bHLH62 isoform X1
---NA---
beta-amyrin 24-hydroxylase-like
1-aminocyclopropane-1-carboxylate oxidase homolog 1-like
albumin-1 D-like
F-box/kelch-repeat protein At3g24760
protein SULFUR DEFICIENCY-INDUCED 1-like
CLAVATA3/ESR (CLE)-related protein TDIF-like
putative receptor protein kinase ZmPK1
probable pectate lyase 8
KEH22909.1 laccase/diphenol oxidase family protein
auxin response factor 9
AES79378.2 plant/T7H20-70 protein
probable glutathione S-transferase
hypothetical protein TSUD_177570
phosphatidylinositol:ceramide inositolphosphotransferase 2 isoform X1
putative copper ion-binding laccase
L-type lectin-domain containing receptor kinase VIII.2
---NA---
mitochondrial uncoupling protein 1
---NA---
T-complex protein 1 subunit beta
GDSL esterase/lipase CPRD49-like
GDSL esterase/lipase CPRD49-like
probable 2-oxoglutarate-dependent dioxygenase At3g111800
trihelix transcription factor GT-3b-like
scarecrow-like protein 26
hypothetical protein MTR_7g069970
protein trichome birefringence-like 34
---NA---
dirigent protein 19-like
uncharacterized protein UGT73P7
soyasapogenol B glucuronide galactosyltransferase
short-chain dehydrogenase reductase 2a-like
senescence associated gene 20-like
---NA---
transcription factor SCREAM2-like
cationic peroxidase 1-like
cationic peroxidase 1
CBL-interacting protein kinase 18-like
transcription repressor OFP2-like

For Review Only

non-specific lipid-transfer protein 1-like
non-specific lipid-transfer protein 1-like
RING-H2 finger protein ATL74-like
uncharacterized protein LOC101497036 isoform X1
protein FATTY ACID EXPORT 3, chloroplastic isoform X4
receptor-like protein kinase FERONIA
probable LRR receptor-like serine/threonine-protein kinase At1g06840 isoform X1
protein SCARECROW
vestitone reductase-like
vestitone reductase
AES80016.2 C2 calcium/lipid-binding and GRAM domain protein
glyoxylate/hydroxypyruvate reductase HPR3-like
phospholipase D gamma 1-like isoform X1
F-box protein At4g00755-like
SPX domain-containing membrane protein At4g22990-like
NAD-dependent malic enzyme 59 kDa isoform, mitochondrial
FT-interacting protein 7-like
---NA---
gibberellin 20-oxidase-like protein
U-box domain-containing protein 33 isoform X2
RHN46970.1 putative HAD-like domain-containing protein
bifunctional L-3-cyanoalanine synthase/cysteine synthase 1, mitochondrial
probable polygalacturonase
uncharacterized protein LOC101492836 isoform X1
F-box/kelch-repeat protein At3g23880-like isoform X1
heavy metal-associated isoprenylated plant protein 39
WRKY transcription factor 22
desiccation protectant protein Lea14 homolog isoform X2
protein TRIGALACTOSYLDIACYLGLYCEROL 3, chloroplastic isoform X1
GDT1-like protein 3
CRIB domain-containing protein RIC4
probable protein phosphatase 2C 33
phospholipase A(1) LCAT3 isoform X2
uncharacterized protein LOC101494553
transcription factor bHLH18-like isoform X2
formin-like protein 1
MDIS1-interacting receptor like kinase 2-like
MDIS1-interacting receptor like kinase 2-like isoform X1
auxin response factor 9
ethylene-responsive transcription factor 1-like
F-box/LRR-repeat protein
eukaryotic translation initiation factor 5-like
GEM-like protein 5 isoform X3
xyloglucan endotransglucosylase/hydrolase precursor
nucleoside-triphosphatase-like isoform X1
NAC transcription factor 47

For Review Only

NAC domain-containing protein 87
glutamate dehydrogenase 1
probable glycosyltransferase At5g03795 isoform X1
spermidine hydroxycinnamoyl transferase-like
plant cysteine oxidase 2-like
ABD28531.1 protein F1K23.6 -, putative
---NA---
inorganic phosphate transporter 1-4-like
p21-activated protein kinase-interacting protein 1-like
protein DETOXIFICATION 21-like
calmodulin-binding protein 25
protein sym-1-like
---NA---
probable auxin efflux carrier component 1b
AES81209.1 family 31 glycosyl hydrolase, alpha-glucosidase
AES81222.1 transmembrane protein, putative
regulatory protein NPR5-like
transcription factor ORG2
calmodulin binding protein PICBP
probable protein phosphatase 2C 43
uncharacterized protein LOC101515199
protein EARLY RESPONSIVE TO DEHYDRATION 15-like
mediator of RNA polymerase II transcription subunit 10b-like protein
AES81346.1 glycosyltransferase family 61 protein
heat stress transcription factor B-3
AES81362.1 transmembrane protein, putative
insulin-degrading enzyme-like 1, peroxisomal isoform X1
leucine-rich repeat receptor-like tyrosine-protein kinase PXC3
dihydroxy-acid dehydratase-like protein
guanylate kinase 2
DIS3-exonuclease-like protein
zinc finger protein WIP2
transcription factor bHLH130-like isoform X1
protein GIGAS CELL1
3,9-dihydroxypterocarpan 6A-monooxygenase
homeobox-leucine zipper protein HOX11
gibberellin receptor GID1B-like
sm-like protein LSM2
protein LONGIFOLIA 1-like
uncharacterized protein LOC101501263
---NA---
auxin-induced protein AUX28-like
E3 ubiquitin protein ligase DRIP2-like isoform X1
cytochrome P450 94A1
poly [ADP-ribose] polymerase 1
ethylene-responsive transcription factor ERF098-like

For Review Only

inorganic phosphate transporter 1-4-like
inorganic phosphate transporter 1-4
transcription factor MYB14
protein LIGHT-DEPENDENT SHORT HYPOCOTYLS 4-like
serine/threonine-protein kinase tricorner
Tetratricopeptide repeat-like superfamily protein, putative isoform 2
uncharacterized protein LOC101492959
transcription factor PIF1-like isoform X3
heat shock cognate 70 kDa protein 2-like isoform X3
cation/H(+) antiporter 20-like
WAT1-related protein At2g37460-like isoform X1
RHN48380.1 putative angiotensin-converting enzyme 2
ankyrin repeat-containing protein At5g02620
probable protein phosphatase 2C 38
glutathione S-transferase L3-like
AES81811.1 protamine P1 family protein
AES81838.1 peptide upstream ORF protein, putative
AES81850.1 senescence regulator
putative phenylalanine ammonia-lyase
heavy metal-associated isoprenylated plant protein 6-like
transcription factor RAX2-like
S-adenosylmethionine synthase 3
probable xyloglucan endotransglucosylase/hydrolase protein 32
ACT domain-containing protein ACR10-like
heparan-alpha-glucosaminide N-acetyltransferase-like isoform X3
thaumatin-like protein 1b
UDP-glycosyltransferase 73C2
UDP-glycosyltransferase 73C1
cytochrome P450 76A1
uncharacterized protein LOC101495759
PAN domain-containing protein At5g03700
growth-regulating factor 4
protein ABSCISIC ACID-INSENSITIVE 5 isoform X1
auxin-responsive protein SAUR15
AES82051.1 glutaredoxin (GRX) family protein
1-aminocyclopropane-1-carboxylate oxidase homolog 4-like
mitochondrial fission protein ELM1
putative pentatricopeptide repeat-containing protein At5g37570
secoisolariciresinol dehydrogenase-like
probable aspartyl protease At4g16563
E3 ubiquitin-protein ligase PUB22
KEH24258.1 glutaredoxin (GRX) family protein
60S ribosomal protein L21-1-like
monothiol glutaredoxin-S6
heavy metal-associated isoprenylated plant protein 36-like
rapid alkalinization factor

For Review Only

thiamine pyrophosphokinase 1 isoform X1
universal stress protein MJ0531-like protein
indole-3-acetic acid-induced protein ARG2
60S ribosomal protein L18-2
dnaJ protein ERDJ3B
protein trichome birefringence-like 6 isoform X1
DELLA protein RGL2-like
probable WRKY transcription factor 69
stachyose synthase
WRKY transcription factor 23
short-chain dehydrogenase reductase 3b-like isoform X1
transcription factor PHYTOCHROME INTERACTING FACTOR-LIKE 15
uncharacterized protein LOC101492848
hypothetical protein MtrDRAFT_AC149038g21v2
hypothetical protein MtrDRAFT_AC149038g24v2
hydrophobic protein LTI6B
hydrophobic protein LTI6B-like
---NA---
dnaJ homolog subfamily B member 6
F-box protein PP2-B15-like isoform X2
---NA---
respiratory burst oxidase homolog protein B-like
aminopeptidase P2
protein YIPF1 homolog
E3 ubiquitin-protein ligase ATL6-like
transcription factor bHLH94-like
histidine-containing phosphotransfer protein 1-like
---NA---
acyltransferase-like protein At1g54570, chloroplastic
aldehyde oxidase GLOX
5'-3' exoribonuclease 4-like
AES82631.1 plant/T24G3-80 protein
probable aldo-keto reductase 1
light-inducible protein CPRF2
---NA---
hypothetical protein glysoja_035587
transcription factor MYB4-like
uncharacterized protein LOC101495764
common plant regulatory factor 1
KEH24632.1 acid phosphatase/vanadium-dependent haloperoxidase
phosphatidylinositol 4-kinase gamma 4-like
ethylene-responsive transcription factor ERF023
KEH24687.1 DUF688 family protein
dof zinc finger protein DOF4.1-like
protein LHY isoform X1
probable RNA-binding protein ARP1 isoform X1

For Review Only

RING-H2 finger protein ATL3-like
CDP-diacylglycerol--glycerol-3-phosphate 3-phosphatidyltransferase 1, chloroplastic-like
NAD(P)-binding Rossmann-fold superfamily protein
uncharacterized protein LOC101504964
AES77287.1 SNARE associated family protein
2-alkenal reductase (NADP(+)-dependent)
LOB domain-containing protein 41
mannose-6-phosphate isomerase 1-like
outer plastidial membrane protein porin
uncharacterized protein LOC101489357
O-acyltransferase WSD1-like isoform X2
aquaporin NIP2-1
alpha-expansin precursor
ent-copalyl diphosphate synthase, chloroplastic-like isoform X1
UDP-glucose 6-dehydrogenase 1
UDP-glycosyltransferase 83A1 isoform X1
histone H3.2
histone H3.2
copper transport protein CCH-like
serine carboxypeptidase-like 25
ninja-family protein AFP3
putative G3BP-like protein isoform X1
phenolic glucoside malonyltransferase 1
isoflavone-7-O-methyltransferase 9
phenolic glucoside malonyltransferase 1-like
putative methylesterase 11, chloroplastic
COBRA-like protein 4
UDP-arabinopyranose mutase 2
protein DOWNY MILDEW RESISTANCE 6-like
E3 ubiquitin-protein ligase At3g02290
AES77801.1 transmembrane protein, putative
basic leucine zipper 43-like
Protein GLUTAMINE DUMPER 2
proline dehydrogenase 2, mitochondrial
hypothetical protein MTR_7g019160
inactive leucine-rich repeat receptor-like protein kinase CORYNE isoform X2
trihelix transcription factor ASR3
serine carboxypeptidase-like 45
cytochrome b (mitochondrion)
zinc finger protein ZAT11-like
enoyl-CoA delta isomerase 1, peroxisomal-like
heat shock cognate 70 kDa protein 2-like
transcription initiation factor TFIID subunit 6
uncharacterized protein LOC101488939 isoform X2
omega-hydroxypalmitate O-feruloyl transferase
merozoite surface protein CMZ-8-like

For Review Only

cullin-1 isoform X1
peroxidase 55
ubiquitin carboxyl-terminal hydrolase 31-like
mitochondrial pyruvate carrier 1-like
uncharacterized protein At3g27210
putative calcium-binding protein CML19
hypothetical protein TanjilG_08301
transcription factor GTE9-like
---NA---
mediator of RNA polymerase II transcription subunit 15a-like protein
kelch repeat-containing protein At3g27220-like isoform X1
myb-related protein 315
---NA---
U4/U6 small nuclear ribonucleoprotein Prp31 homolog isoform X1
CASP-like protein 4A1 isoform X1
E3 ubiquitin-protein ligase listerin isoform X2
putative leucine-rich repeat domain, L domain-containing protein
cysteine-rich repeat secretory protein 15
putative expansin-B2
transcription factor UNE10
KEH22364.1 zinc ion-binding protein
carotenoid cleavage dioxygenase 7 O isoform
ethylene-responsive transcription factor ERF034
hydroquinone glucosyltransferase
RHN45586.1 putative phosphoglycerate/bisphosphoglycerate mutase, active, histidine phosphatase superfamily
eukaryotic translation initiation factor 4G-like
ABD33414.1 Pectinesterase; Pectinesterase inhibitor
AES78906.1 pectinesterase/pectinesterase inhibitor
putative lipid-transfer protein DIR1
U-box domain-containing protein 9-like
uncharacterized protein LOC101514428
metallothionein-like protein 4B
GTP cyclohydrolase 1-like
galacturonosyltransferase 8
AES79074.1 transmembrane protein, putative
1-aminocyclopropane-1-carboxylate oxidase homolog 1-like
suppressor of disruption of TFIIS
cyclin-dependent protein kinase inhibitor SMR4
protein SRG1-like
transcription factor bHLH155-like
---NA---
hypothetical protein MTR_7g064050
protein NRT1/ PTR FAMILY 5.6-like
probable glutathione S-transferase
probable glutathione S-transferase
probable glutathione S-transferase

For Review Only

glutathione s-transferase 2
universal stress protein PHOS32-like
putative transposase, Ptta/En/Spm, plant
queuine tRNA-ribosyltransferase subunit QTRTD1
protein SSUH2 homolog
Werner Syndrome-like exonuclease
Werner Syndrome-like exonuclease
receptor-like protein 13 isoform X1
T-complex protein 1 subunit beta-like isoform X2
putative serine/threonine-protein kinase isoform X2
ABD33312.1 GCN5-related N-acetyltransferase; BRCT
---NA---
vacuolar cation/proton exchanger 3
GDSL esterase/lipase CPRD49-like
GDSL esterase/lipase CPRD49-like
GDSL esterase/lipase CPRD49-like isoform X4
GDSL esterase/lipase CPRD49-like
omega-3 fatty acid desaturase, chloroplastic-like
importin-11-like isoform X2
ammonium transporter 2
putative Myb family transcription factor At1g14600 isoform X1
uncharacterized protein LOC101501670
ferritin-1, chloroplastic
uncharacterized protein UGT73P7
soyasapogenol B glucuronide galactosyltransferase
probable WRKY transcription factor 33
RHN38382.1 putative tetratricopeptide-like helical domain-containing protein
TIR-NBS-LRR resistance protein
protein LURP-one-related 10
alanine--glyoxylate aminotransferase 2 homolog 3, mitochondrial-like
CBL-interacting protein kinase 18-like
---NA---
probable WRKY transcription factor 70
ubiquitin-conjugation enzyme
---NA---
protein PIN-LIKES 6
calcium-binding allergen Bet v 3-like
AES79885.1 late embryogenesis abundant protein
protein LITTLE ZIPPER 2
E3 ubiquitin-protein ligase At1g12760-like
F-box protein At4g00755-like
putative ripening-related protein 1
serine/threonine-protein kinase BSK3-like
probable E3 ubiquitin-protein ligase RHA1A
mitogen-activated protein kinase homolog MMK2
C-terminal binding protein AN

For Review Only

1-aminocyclopropane-1-carboxylate synthase 1-like
sphingoid long-chain bases kinase 2, mitochondrial-like
chromatin remodeling complex subunit
protein LUTEIN DEFICIENT 5, chloroplastic isoform X1
flavin mononucleotide hydrolase 1, chloroplatic
receptor-like protein CLAVATA2
photosystem I reaction center subunit XI, chloroplastic
isopentenyl-diphosphate Delta-isomerase I
---NA---
transmembrane protein, putative
acyl carrier protein 1, mitochondrial
subtilisin-like protease Glyma18g48580
subtilisin-like protease Glyma18g48580
ethylene-responsive transcription factor 1-like
uncharacterized protein LOC101513998
receptor-like kinase TMK4
LEAF RUST 10 DISEASE-RESISTANCE LOCUS RECEPTOR-LIKE PROTEIN KINASE-like 2.4 isoform X1
LEAF RUST 10 DISEASE-RESISTANCE LOCUS RECEPTOR-LIKE PROTEIN KINASE-like 1.4
putative glutamine amidotransferase GAT1_2.1
non-specific lipid-transfer protein 2
uncharacterized protein LOC101494758
AES80631.1 transmembrane protein, putative
CASP-like protein 1F2
glycerophosphodiester phosphodiesterase GDPD1, chloroplastic-like
zinc finger protein CONSTANS-LIKE 15-like isoform X2
GEM-like protein 5 isoform X2
mitochondrial phosphate carrier protein 3, mitochondrial
light-regulated protein 1, chloroplastic
---NA---
ABD33170.1 disease resistance gene, putative
xyloglucan endotransglucosylase/hydrolase precursor
dnaJ homolog subfamily B member 7
DNA ligase 1
ethylene-responsive transcription factor ERN1-like
spermidine hydroxycinnamoyl transferase-like
---NA---
drought-responsive protein
cation/calcium exchanger 2-like
AES80942.1 transmembrane protein, putative
transcription factor MYB54 isoform X1
GDSL esterase/lipase At3g26430-like
uncharacterized protein LOC101488507
protein PHYTOCHROME KINASE SUBSTRATE 4-like
WAT1-related protein At5g07050-like
probable mediator of RNA polymerase II transcription subunit 26b
AES81209.1 family 31 glycosyl hydrolase, alpha-glucosidase

For Review Only

amino acid transporter AVT1A-like

---NA---

glutaredoxin family protein

two-component response regulator ARR9

uncharacterized protein LOC101509910

probable galactinol--sucrose galactosyltransferase 2

calcium-dependent calmodulin-independent protein kinase isoform 2

remorin 4.1-like

---NA---

zinc finger A20 and AN1 domain-containing stress-associated protein 8-like

polygalacturonase inhibitor-like

polygalacturonase inhibitor-like

bZIP transcription factor TGA10-like isoform X2

CLAVATA3/ESR-related 39

KEH30599.1 alpha 1,4-glycosyltransferase family protein, putative

seed maturation protein LEA 4

protein trichome birefringence

activator of 90 kDa heat shock protein ATPase homolog 1

dirigent protein 2-like

dirigent protein 22-like

histone H2AX-like

receptor-like protein 31

hypothetical protein MtrunA17_Chr7g0259351

hypothetical protein MTR_7g094270

protein FANTASTIC FOUR 1

probable inactive patatin-like protein 9

F-box/kelch-repeat protein SKIP30

protein ANTAGONIST OF LIKE HETEROCHROMATIN PROTEIN 1

sulfate transporter 3.1-like

calcium-transporting ATPase 12, plasma membrane-type

auxin-induced protein 22E-like

ethylene-responsive transcription factor 8-like

LOB domain-containing protein 29-like

F-box protein At2g26160

pollen Ole e I family allergen

CRIB domain-containing protein RIC7

phospholipid--sterol O-acyltransferase isoform X2

uncharacterized protein LOC101502107

MDIS1-interacting receptor like kinase 1-like

Subtilisin-like protease SBT1.2

photosystem I reaction center subunit IV, chloroplastic-like

uncharacterized protein LOC101503165

uncharacterized protein LOC101499143

zinc finger protein ZAT11-like

zinc finger protein ZAT11-like

protein SODIUM POTASSIUM ROOT DEFECTIVE 2-like

For Review Only

protein ALUMINUM SENSITIVE 3
probable beta-1,4-xylosyltransferase IRX9
transcription factor RAX2-like
probable pectate lyase 12
UDP-glycosyltransferase 73C2
purine-uracil permease NCS1-like
pistil-specific extensin-like protein
wall-associated receptor kinase-like 20 isoform X1
leucine-rich repeat receptor-like protein kinase PXC1
uncharacterized protein LOC101499859
putative LysM domain-containing protein
purple acid phosphatase 22
uncharacterized protein LOC101511310
rho GTPase-activating protein 5
secoisolariciresinol dehydrogenase-like
uncharacterized protein LOC101498714
NDR1/HIN1-like protein 10
NDR1/HIN1-like protein 10
BTB/POZ domain-containing protein At3g05675
fasciclin-like arabinogalactan protein 16
probable carboxylesterase 15
probable E3 ubiquitin-protein ligase BAH1-like
uncharacterized protein LOC101489930
ubiquitin carboxyl-terminal hydrolase family protein
pre-mRNA-splicing factor prp12 isoform X1
peroxidase 3-like
uncharacterized protein LOC101494570
zinc finger protein CONSTANS-LIKE 13
---NA---
monothiol glutaredoxin-S11
wee1-like protein kinase
dephospho-CoA kinase
protein LOW PSII ACCUMULATION 1, chloroplastic
xyloglucan 6-xylosyltransferase 2
phytochrome A-associated F-box protein
uncharacterized protein At5g01610-like
probable pectinesterase/pectinesterase inhibitor 7
protein NDR1-like
DnaJ heat shock n-terminal domain-containing protein
AES82177.2 DUF4228 domain protein
calvin cycle protein CP12-2, chloroplastic-like
probable WRKY transcription factor 69
polygalacturonase At1g48100
s-adenosylmethionine synthetase
two-pore potassium channel 5
auxin-responsive protein IAA30

For Review Only

AES82299.1 charged multivesicular body protein

paladin-like isoform X2

cathepsin B-like protease 2

---NA---

transcription factor MYB61-like

hypothetical protein MtrDRAFT_AC157894g34v2

aspartic proteinase isoform X1

galactinol synthase 2-like

chloroplast stem-loop binding protein of 41 kDa b, chloroplastic

KEH24470.1 plant/F25P12-18 protein

calmodulin-binding protein 60 G-like

wall-associated receptor kinase-like 20

AES82458.2 epoxide hydrolase

dormancy-associated protein homolog 3 isoform X1

protein RALF-like 33

heavy metal-associated isoprenylated plant protein 35-like

respiratory burst oxidase homolog protein B-like

uncharacterized protein LOC101502432 isoform X2

DNA polymerase zeta catalytic subunit

cation/calcium exchanger 5

protein FANTASTIC FOUR 1-like

sugar transporter ERD6-like 5

AES82595.2 acetyltransferase (GNAT) domain protein

proteasome subunit beta type-4-like isoform X1

GDP-mannose 3,5-epimerase 2

protein WVD2-like 1 isoform X1

pentatricopeptide repeat-containing protein At3g61520, mitochondrial-like

ankyrin repeat domain-containing protein 13C

uncharacterized protein LOC101493502 isoform X2

GDSL esterase/lipase At4g01130 isoform X1

hydroquinone glucosyltransferase

phospholipase A1 PLIP1, chloroplastic isoform X1

transcription factor bHLH13-like

uncharacterized protein LOC101514667

U-box domain-containing protein 44-like

ATP synthase delta chain, chloroplastic

3-ketoacyl-CoA synthase 1-like isoform X1

probable L-gulonolactone oxidase 6

XP_012569670.1 exportin-T

---NA---

uncharacterized protein LOC101507158

---NA---

NADH-quinone oxidoreductase protein

transmembrane protein, putative

uncharacterized protein LOC101499348

---NA---

For Review Only

---NA---
---NA---
ABC transporter A family member 4-like
---NA---
---NA---
---NA---
hypothetical protein L195_g052424
---NA---
---NA---
KEH22993.1 legume-specific protein
---NA---
---NA---
KEH20845.1 MFS transporter
---NA---
---NA---
transmembrane protein, putative
transmembrane protein, putative
transmembrane protein, putative
hypothetical protein MtrDRAFT_AC150244g1v2
Phosphatidate phosphatase PAH1
probable membrane-associated kinase regulator 3
zinc finger protein ZAT11
probable receptor-like protein kinase At2g47060 isoform X2
soluble inorganic pyrophosphatase 4 isoform X2
uncharacterized protein LOC101502221
photosystem I reaction center subunit IV A, chloroplastic-like
ABC transporter C family member 5
xyloglucan galactosyltransferase MUR3-like
cellulose synthase A catalytic subunit 4 [UDP-forming]
uncharacterized protein LOC101493183
serine/threonine-protein kinase 19 isoform X2
protein CUP-SHAPED COTYLEDON 3-like
ethylene-responsive transcription factor ERF098
ethylene-responsive transcription factor 1B
transcription factor EMB1444
LOB domain-containing protein 29
PRA1 family protein D-like
photosystem II reaction center W protein, chloroplastic-like
auxin-induced protein 22D-like isoform X2
uncharacterized protein LOC101488851
beta-carotene isomerase D27, chloroplastic-like
protein LIFEGUARD 2-like
non-specific lipid-transfer protein-like protein At2g13820
FCS-Like Zinc finger 8-like
KEH42637.1 heat shock 22 kDa protein, putative
transcription repressor OFP12-like

For Review Only

uncharacterized protein LOC101503605
probable transmembrane GTPase FZO-like, chloroplastic
uncharacterized protein LOC101507063
3-isopropylmalate dehydratase large subunit, chloroplastic-like isoform X2
myelin gene regulatory factor-like A isoform X3
acid phosphatase 1-like protein
ethylene receptor 2
ethylene-responsive transcription factor ERF096-like
ethylene-responsive transcription factor 1B
probable inorganic phosphate transporter 1-3
protein SIEVE ELEMENT OCCLUSION B-like isoform X2
uncharacterized protein LOC101495639
phosphatase 2C-like protein 44
U-box domain-containing protein 44-like
guanine nucleotide-binding protein subunit gamma 2
calmodulin-like protein 11
reticuline oxidase-like
peroxidase 47
KEH42859.1 plant/F12B17-70 protein
uncharacterized protein LOC101512294 isoform X2
testis-expressed protein 2-like isoform X2
aluminum-activated malate transporter 10 isoform X4
metal-nicotianamine transporter YSL1
heavy metal-associated isoprenylated plant protein 30-like
ethylene receptor like-protein
AT-hook motif nuclear-localized protein 19
GDP-mannose 3,5-epimerase 2-like
kinesin-like protein KIN-14U isoform X2
phosphoglycerate mutase-like protein AT74
respiratory burst oxidase homolog protein B-like isoform X2
uncharacterized protein LOC101493941
dormancy-associated protein homolog 3 isoform X2
RHN80837.1 putative DNA helicase
uncharacterized protein LOC101513908
universal stress protein A-like protein
uncharacterized protein LOC101510246
nicotianamine synthase-like
---NA---
galactinol synthase 2
uncharacterized protein LOC113786344
uncharacterized protein LOC113786344
uncharacterized protein LOC113786344
hypothetical protein MTR_0187s0020
enolase-phosphatase E1-like isoform X2
transcription factor MYB61
hypothetical protein MTR_1g085190

For Review Only

auxin-responsive protein IAA30-like
peroxidase 5-like
polygalacturonase At1g48100
protein NIM1-INTERACTING 1-like
ADP ribosylation factor
transcription factor MYB78-like
transcription factor MYB108-like
beta-carotene isomerase D27, chloroplastic isoform X1
calvin cycle protein CP12-2, chloroplastic-like
AES61673.1 DNAJ heat shock amino-terminal domain protein
uncharacterized protein LOC101494577
nicotinamide adenine dinucleotide transporter 1, chloroplastic isoform X2
ethylene-responsive transcription factor ERF073-like
AES61737.2 pollen Ole e I family allergen
proline-rich protein 3-like
---NA---
AES61800.2 CS domain protein
glutaredoxin-c11-like protein
probable ATP-dependent RNA helicase ddx56
ankyrin repeat domain-containing protein 50-like
auxin-binding protein T85 isoform X1
KEH43300.1 DUF1635 family protein
uncharacterized protein LOC101495324
4-coumarate--CoA ligase-like 5
---NA---
AES61996.2 plant/T32M21-140 protein
uncharacterized protein LOC101510792
---NA---
Succinate dehydrogenase subunit 7B, mitochondrial isoform B
phosphoenolpyruvate carboxylase 4
ABC transporter G family member 21
---NA---
hypothetical protein TSUD_39670
uncharacterized protein LOC101503057
GATA transcription factor 8
putative transcription factor ARF family
aquaporin PIP2-1-like
patatin-like protein 6
hypothetical protein TSUD_41220
embryogenesis-associated protein EMB8-like
isoliquiritigenin 2'-O-methyltransferase
uncharacterized protein LOC101494056
somatic embryogenesis receptor kinase 2
---NA---
60S ribosomal protein L3-1
putative ABA/WDS induced protein

For Review Only

trihelix transcription factor GT-2-like
ABC transporter C family member 10-like
acidic endochitinase precursor
putative chitinase
probable membrane-associated kinase regulator 6
beta-carotene hydroxylase 2, chloroplastic
protein PATRONUS 2-like isoform X1
uncharacterized protein LOC113786378
AES62534.1 PAR1 protein
transcription factor MYB41-like isoform X1
Clavata3/ESR (CLE) gene family member MtCLE05
3-hexulose-6-phosphate isomerase-like protein
uncharacterized protein LOC101504477
PvSR7 protein
ethylene-responsive transcription factor ERF027-like
dehydration-responsive element-binding protein 1A
molybdenum cofactor sulfurase-like protein
peroxidase 66
perakine reductase-like
early light-induced protein, chloroplastic-like
leucine-rich repeat extensin-like protein 6 isoform X2
homocysteine S-methyltransferase 3-like
type IV inositol polyphosphate 5-phosphatase 7
FCS-Like Zinc finger 8-like isoform X1
uncharacterized protein LOC101514017
---NA---
haloacid dehalogenase superfamily protein
NDR1/HIN1-like protein 26
cysteine-rich receptor-like protein kinase 25
cysteine-rich receptor-like protein kinase 10 isoform X1
Cysteine-rich receptor protein kinase 25
cysteine-rich receptor-like protein kinase 10 isoform X5
zinc finger protein ZAT10-like
KEH44041.1 gibberellin-regulated family protein
methylthioribose kinase-like
KEH44066.1 plant basic secretory protein (BSP) family protein
receptor protein kinase-like protein At4g34220
D-3-phosphoglycerate dehydrogenase 1, chloroplastic
uncharacterized protein LOC101492975
uncharacterized protein LOC101493310
probable ribose-5-phosphate isomerase 2
exonuclease V, chloroplastic-like
E3 ubiquitin-protein ligase MARCH2
sulfhydryl oxidase 2 isoform X1
tetratricopeptide repeat protein 5-like isoform X2
ATP synthase subunit beta, mitochondrial

For Review Only

---NA---
origin of replication complex subunit 4
amino-acid permease BAT1 homolog
LEAF RUST 10 DISEASE-RESISTANCE LOCUS RECEPTOR-LIKE PROTEIN KINASE-like 1.1
probable protein phosphatase 2C 15
AES62900.1 transmembrane protein, putative
IST1 homolog isoform X3
uncharacterized protein LOC101498397
dihydrolipoyllysine-residue acetyltransferase component 4 of pyruvate dehydrogenase complex, chloroplastic
KEH44246.1 regulator of Vps4 activity in the MVB pathway protein
cytochrome P450 CYP73A100-like
uncharacterized protein LOC101505214
KEH44263.1 P-loop nucleoside triphosphate hydrolase superfamily protein
putative major facilitator superfamily domain-containing protein
transcription factor SRM1
germin-like protein subfamily 3 member 2
SNW/SKI-interacting protein A-like
dolichyl-diphosphooligosaccharide--protein glycosyltransferase subunit STT3A
transmembrane protein, putative
myosin-J heavy chain-like protein
transmembrane protein 53
probable glutathione S-transferase
protein ASPARTIC PROTEASE IN GUARD CELL 2
CASP-like protein 4C2
zinc finger protein ZAT11-like
60S ribosomal protein l35-like
Chalcone--flavonone isomerase 1B-1
---NA---
cytochrome P450 76A1-like
putative Extensin domain-containing protein
---NA---
probable terpene synthase 2
putative serine/threonine-protein kinase isoform X1
cytokinin riboside 5'-monophosphate phosphoribohydrolase LOG3 isoform X1
protein NUCLEAR FUSION DEFECTIVE 4 isoform X1
phenylalanine ammonia-lyase 1-like
adenosine kinase 2
heavy metal-associated isoprenylated plant protein 5
protein PTST homolog 3, chloroplastic isoform X2
Alpha carbonic anhydrase 7
zinc finger protein ZAT5
phosphatidylinositol 4-phosphate 5-kinase 9 isoform X1
peptidyl-prolyl cis-trans isomerase FKBP16-4, chloroplastic
threonine dehydratase biosynthetic, chloroplastic-like
heat stress transcription factor A-3
peptidyl-prolyl cis-trans isomerase FKBP17-1, chloroplastic

For Review Only

fructose-bisphosphate aldolase 6, cytosolic isoform X2
IQ domain-containing protein IQM3
cytochrome P450 94C1-like
protein LITTLE ZIPPER 3
casparian strip membrane protein 1
casparian strip membrane protein 2
uncharacterized protein LOC101512833
protein SMAX1-LIKE 3
BEL1-like homeodomain protein 1
E3 ubiquitin-protein ligase PUB23-like
syntaxin-121-like protein
AES60579.1 syntaxin of plants 122 protein
KEH41854.1 START-2 domain protein
prolycopene isomerase, chloroplastic isoform X1
ABC transporter G family member 22-like isoform X2
protein FLOWERING LOCUS D
serine/arginine-rich SC35-like splicing factor SCL33
---NA---
---NA---
XP_012570483.1 actin-97
11 kDa late embryogenesis abundant protein-like
uncharacterized protein LOC101509942
myb family transcription factor PHL5
uncharacterized protein LOC101492980
uncharacterized protein LOC101492980
bZIP transcription factor TGA10
internal alternative NAD(P)H-ubiquinone oxidoreductase A2, mitochondrial-like
transmembrane protein, putative
protein DETOXIFICATION 42-like
remorin 4.2-like
tubulin-folding cofactor B isoform X1
---NA---
---NA---
geraniol 8-hydroxylase
---NA---
protein trichome birefringence-like 36
KEH41383.1 hemerythrin HHE cation-binding domain protein
protein TAPETUM DETERMINANT 1
---NA---
linker histone H1 and h5 family protein
transcription factor ORG2-like
ethylene-responsive transcription factor ERF1
uncharacterized protein LOC101507395 isoform X2
putative glycosyl transferase, family 14
DUF688 family protein
DNA mismatch repair protein MSH1, mitochondrial isoform X1

For Review Only

heme oxygenase 1, chloroplastic-like
alpha-1,6-mannosyl-glycoprotein 2-beta-N-acetylglucosaminyltransferase
golgin subfamily A member 6-like protein 7
2-alkenal reductase (NADP(+)-dependent)-like
splicing factor 3B subunit 6-like protein
actin-related protein 2/3 complex subunit 2B
---NA---
ethylene-responsive transcription factor 1B-like
serine/threonine-protein phosphatase PP1-like isoform X2
probable inorganic phosphate transporter 1-3
transcription factor MYB14-like
phosphate transporter PHO1-like
KEH41039.1 DUF506 family protein
CBL-interacting serine/threonine-protein kinase 4-like
serine/threonine receptor-like kinase NFP
auxin-induced protein IAA4
transcription factor bHLH92 isoform X1
benzyl alcohol O-benzoyltransferase-like
methylenetetrahydrofolate reductase 2-like
type IV inositol polyphosphate 5-phosphatase 9-like isoform X1
65-kDa microtubule-associated protein 6
KEH40918.1 transmembrane protein, putative
isoliquiritigenin 2'-O-methyltransferase-like
isoliquiritigenin 2'-O-methyltransferase-like
AES59929.1 cysteine/histidine-rich C1 domain protein
KEH40771.1 cysteine/histidine-rich C1 domain protein
extra-large guanine nucleotide-binding protein 1-like isoform X1
RNA demethylase ALKBH10B-like isoform X1
uncharacterized protein LOC101515113
KEH40586.1 translocation protein TolB
zinc finger protein VAR3, chloroplastic
exosome complex component RRP42-like
mitochondrial cytochrome c oxidase subunit II (mitochondrion)
protein C2-DOMAIN ABA-RELATED 9-like
uncharacterized protein LOC101498273
rust resistance kinase Lr10-like isoform X3
WAT1-related protein At4g08290
uncharacterized protein At4g08330, chloroplastic-like
aldehyde dehydrogenase family 3 member H1
amino acid permease 3-like
NAD kinase 2, chloroplastic-like isoform X1
F-box protein SKP2B
scarecrow-like protein 1
arabinogalactan peptide-like protein
cyclic nucleotide-gated ion channel 1-like protein
rust resistance kinase Lr10-like isoform X1

For Review Only

rust resistance kinase Lr10
putative wall-associated receptor kinase-like 16
receptor-like protein kinase
LEAF RUST 10 DISEASE-RESISTANCE LOCUS RECEPTOR-LIKE PROTEIN KINASE-like 2.1
sulfite exporter TauE/SafE family protein 3-like isoform X2
photosystem I reaction center subunit psaK, chloroplastic
aquaporin TIP4-1
probable glycosyltransferase At5g03795
uncharacterized protein LOC109799781
dehydration-responsive element-binding protein 3-like
uncharacterized protein LOC101502451
AES58745.1 transmembrane protein, putative
AES58749.1 alpha/beta fold hydrolase
protein BOBBER 1
BURP domain protein RD22
protein NRT1/ PTR FAMILY 1.2-like
probable WRKY transcription factor 17
pentatricopeptide repeat-containing protein At4g32430, mitochondrial-like isoform X1
peroxidase 45-like
FCS-Like Zinc finger 10-like
molybdate transporter 1
serine/threonine protein phosphatase 2A 57 kDa regulatory subunit B' kappa isoform-like
gibberellin 3-beta-dioxygenase 1-like
pleiotropic drug resistance protein 1-like isoform X1
V-type proton ATPase subunit c''2
EIN3-binding F-box protein 1
protein MEMO1
ethylene-responsive transcription factor ERF003-like
zinc finger protein 3
U-box domain-containing protein 6
tocopherol O-methyltransferase, chloroplastic
RNA demethylase ALKBH9B
KEH39908.1 DUF1677 family protein
protein BIG GRAIN 1-like B
cytokinin dehydrogenase 7
glutaredoxin-c6-like protein
protein indeterminate-domain 7-like
glycine dehydrogenase (decarboxylating), mitochondrial isoform X1
vacuolar-processing enzyme-like isoform X2
homeotic protein knotted-1 isoform X1
transcription factor RAX3-like isoform X2
phytosulfokine precursor protein
uncharacterized protein LOC101508556
F-box protein At3g12350
beta-galactosidase-like precursor
zinc finger protein ZAT10

For Review Only

subtilisin-like protease SBT1.7
uncharacterized protein At4g06744
transcription factor SPATULA-like isoform X3
putative calcium-binding protein CML10-like
EF hand protein
EF-hand pair protein
AES59472.1 plant/F17M5-140 protein
uncharacterized protein LOC101511335
polyadenylate-binding protein RBP47C-like
AES59499.1 DUF1645 family protein
B3 domain-containing protein REM16-like
probable transcription factor KAN4
allene oxide synthase 1, chloroplastic
lysM domain receptor-like kinase 4
elongation of fatty acids protein 3-like
uncharacterized protein LOC101489629
beta-galactosidase 5-like precursor
protein BASIC PENTACYSTEINE6
auxin-induced protein 6B-like
HVA22-like protein i
AES59723.1 C2H2-like zinc finger protein
uncharacterized protein LOC101496544 isoform X1
syringolide-induced protein 14-1-1
---NA---
short-chain dehydrogenase/reductase family 42E member 1
cell division control protein 2 homolog D
WAT1-related protein At2g37460-like
RHN79956.1 putative myosin ATPase
ketohexokinase isoform X1
ribokinase-like isoform X1
uncharacterized protein LOC101511860
photosystem II reaction center PSB28 protein, chloroplastic
---NA---
ethylene-responsive transcription factor 1B-like
ethylene-responsive transcription factor 1B
protein DOWNY MILDEW RESISTANCE 6-like
cytochrome P450 94A2-like
heat stress transcription factor A-6b
serine/threonine-protein kinase Nek4
F-box protein At2g27310-like
basic 7S globulin-like
basic 7S globulin
uncharacterized protein LOC113786164
uncharacterized protein LOC101490378
transcription factor WER-like
---NA---

For Review Only

cysteine-rich repeat secretory protein 3
cellulose synthase-like protein G2
hypothetical protein MTR_1g073770
uncharacterized protein At4g14450, chloroplastic
electron transfer flavoprotein subunit beta, mitochondrial
RING-H2 finger protein ATL16
serine/threonine-protein phosphatase PP1
protein NRT1/ PTR FAMILY 5.2-like
peroxisomal membrane protein PMP22
protein LIGHT-DEPENDENT SHORT HYPOCOTYLS 3 isoform X1
putative E3 ubiquitin-protein ligase XBAT34
basic 7S globulin
auxin-responsive protein IAA14-like
uncharacterized protein LOC101515752
DUF3475 domain protein
---NA---
---NA---
22.7 kDa class IV heat shock protein
---NA---
alpha-galactosidase isoform X2
KEH42900.1 plastocyanin-like domain protein
ubiquitin carboxyl-terminal hydrolase 2-like
ammonium transporter 1 member 2
AT-hook motif nuclear-localized protein 19
rubisco accumulation factor 1.1, chloroplastic
phosphoenolpyruvate carboxylase kinase 1-like
protein LIGHT-DEPENDENT SHORT HYPOCOTYLS 1
AES61227.1 coatomer protein
uncharacterized protein LOC101489287
GRF1-interacting factor 1
pathogenesis-related protein PR-4 precursor
uncharacterized protein LOC101496636 isoform X2
guanosine deaminase
---NA---
transcription factor bHLH94
translationally-controlled tumor protein homolog
F-box protein PP2-B15-like
---NA---
calreticulin-like protein
probable L-cysteine desulfhydrase, chloroplastic
aspartic proteinase-like isoform X2
uncharacterized protein LOC113786344
enolase-phosphatase E1
protein NRT1/ PTR FAMILY 4.5
protein RADIALIS-like 3
---NA---

For Review Only

gibberellin 2-beta-dioxygenase 2
bZIP transcription factor
phospholipase A1 PLIP2, chloroplastic
uncharacterized protein LOC101508750
AES61705.1 DUF761 domain protein
ethylene-responsive transcription factor ERF073-like
indole-3-acetic acid-induced protein ARG2-like
UDP-glucuronic acid decarboxylase 2
nuclear transcription factor Y subunit B-5
classical arabinogalactan protein 26
serine/threonine-protein phosphatase BSL1
receptor-like cytosolic serine/threonine-protein kinase RBK2
endonuclease/exonuclease/phosphatase family protein
ethylene-responsive transcription factor ERF062
Oleosin 1
---NA---
ethylene-responsive transcription factor ERF107
uncharacterized protein LOC101495324
putative disease resistance RPP13-like protein 2
SEC12-like protein 1
glycosyltransferase family protein 64 protein C5
pyruvate kinase 1, cytosolic-like
GABA transporter 1-like
cytochrome P450 77A3-like
Myb/SANT-like DNA-binding domain protein
transcription factor HHO3-like isoform X1
AP2/ERF and B3 domain-containing transcription repressor TEM1
AES62082.2 transmembrane fragile-X-F-associated protein
phosphoenolpyruvate carboxylase 4
vacuolar protein-sorting-associated protein 33 homolog
phenylalanine ammonia-lyase 2
---NA---
tetraspanin-6-like
LOB domain-containing protein 1-like
---NA---
alkaline/neutral invertase CINV2
probable LRR receptor-like serine/threonine-protein kinase At1g34110
transcription factor UPBEAT1-like
type IV inositol polyphosphate 5-phosphatase 3-like
probable choline kinase 1
carboxypeptidase SOL1 isoform X2
hypothetical protein LR48_Vigan02g200400
peroxidase 12
ethylene-responsive transcription factor ERF110-like isoform X2
uncharacterized protein LOC101490484
thioredoxin Y1 chloroplastic-like

For Review Only

AES62351.1 plant/F1M20-13 protein
vacuolar iron transporter homolog 2-like
XP_004496490.1 extensin-3
heavy metal-associated isoprenylated plant protein 39
---NA---
calmodulin-binding heat-shock protein
---NA---
receptor-like protein EIX1
pentatricopeptide repeat-containing protein At1g55630-like
protein IQ-DOMAIN 14-like
PTI1-like tyrosine-protein kinase At3g15890
transcription factor bHLH36-like
receptor-like serine/threonine-protein kinase At4g25390
alpha-L-fucosidase 1-like
xyloglucan galactosyltransferase XLT2
KEH43760.1 hydroxyproline-rich glycoprotein family protein
Dehydration-responsive element-binding protein 2D
hypothetical protein D0Y65_028056
primary amine oxidase 2-like
probable LRR receptor-like serine/threonine-protein kinase At1g67720
2-hydroxyisoflavanone dehydratase-like
pre-mRNA-splicing factor 38B-like
PXMP2/4 family protein 4-like
protein FAR-RED IMPAIRED RESPONSE 1 isoform X1
probable prolyl 4-hydroxylase 9
UDP-glycosyltransferase 84B2-like
cyclin D3
---NA---
exonuclease V, chloroplastic-like
AES62821.1 DUF4228 domain protein
zinc finger CCCH domain-containing protein 15 isoform X1
amino-acid permease BAT1 homolog
lipid phosphate phosphatase 2-like
uncharacterized protein LOC101512519 isoform X1
reticulon-like protein B14
AES62858.1 DUF789 family protein
KEH44200.1 core-2/I-branching enzyme
uncharacterized protein LOC101506517
endonuclease 2
VQ motif-containing protein 8, chloroplastic
protein trichome birefringence-like 36
diphosphomevalonate decarboxylase MVD2, peroxisomal
protein indeterminate-domain 5, chloroplastic isoform X1
nucleotide-diphospho-sugar transferase family protein
serine/threonine protein phosphatase 2A 57 kDa regulatory subunit B' beta isoform-like isoform X1
mechanosensitive ion channel protein 6-like

For Review Only

serine/threonine-protein kinase STY46 isoform X1
CAX-interacting protein 4
---NA---
---NA---
putative GEM-like protein 8
GEM-like protein 4
RING-H2 finger protein ATL18
CASP-like protein 4C2
dirigent protein 25-like
chalcone--flavonone isomerase 2
cationic peroxidase 1-like
cytochrome P450 76A1-like
uncharacterized protein LOC101507812
---NA---
---NA---
---NA---
peroxidase 21
---NA---
---NA---
XP_028183191.1 cytokinin riboside 5'-monophosphate phosphoribohydrolase LOG3-like isoform X1
auxin-responsive protein SAUR32
heavy metal-associated isoprenylated plant protein 5
probable pectate lyase 12
aldehyde dehydrogenase family 3 member H1-like isoform X2
---NA---
serine/threonine-protein kinase VPS15
protein DMR6-LIKE OXYGENASE 1-like isoform X2
aspartic proteinase-like protein 2
pyruvate kinase 1, cytosolic
leucine-rich repeat receptor-like protein kinase PXC1
glucomannan 4-beta-mannosyltransferase 9-like
dehydration-responsive element-binding protein 1B-like
uncharacterized protein LOC101492207
GDSL esterase/lipase At5g03610-like
purple acid phosphatase 22-like
RHN79545.1 putative calcium/calmodulin-dependent protein kinase
putative pentatricopeptide repeat-containing protein At3g11460, mitochondrial
probable F-box protein At2g36090
transmembrane protein, putative
DExH-box ATP-dependent RNA helicase DExH1 isoform X1
AES60550.1 EEIG1/EHBP1 protein amino-terminal domain protein
protein WVD2-like 4
uncharacterized protein LOC101495448
ABC transporter G family member 22-like isoform X1
calcium-dependent protein kinase 20
MATH domain and coiled-coil domain-containing protein At3g58410 isoform X1

For Review Only

kinesin-like protein KIN-7O isoform X2
homeobox-leucine zipper protein HOX11-like
peroxidase A2-like
VQ motif-containing protein 25-like
---NA---
DUF761 domain protein
bZIP transcription factor TGA10
receptor protein kinase-like protein ZAR1
serine/threonine-protein kinase Nek5-like isoform X2
uncharacterized protein LOC101508857
protein MIZU-KUSSEI 1
zeatin O-glucosyltransferase
---NA---
two-component response regulator ARR9-like isoform X1
uncharacterized protein LOC101502600
linker histone H1 and h5 family protein
DNA mismatch repair protein MSH1, mitochondrial-like
probable pectate lyase 1
---NA---
zinc finger BED domain-containing protein RICESLEEPER 1-like
KEH41204.1 ABC1/COQ8 Serine/Threonine kinase
dirigent protein 18-like
oxidation resistance protein 1-like isoform X1
ammonium transporter 1 member 1-like
transcription factor MYB24-like
protein PROTON GRADIENT REGULATION 5, chloroplastic
actin-related protein 2/3 complex subunit 2B
transmembrane protein, putative
uncharacterized protein At4g14450, chloroplastic-like
DNA repair protein RAD5B
calmodulin-like protein 11
armadillo repeat-containing protein LFR
auxin-induced protein IAA4
peroxidase 5-like
WAT1-related protein At3g30340-like isoform X1
B3 domain-containing transcription factor VRN1-like
AES59936.1 transmembrane protein, putative
KEH40771.1 cysteine/histidine-rich C1 domain protein
allene oxide synthase 3
KEH40761.1 cytochrome b6f complex subunit (PetM), putative
tobamovirus multiplication protein 3
zinc finger protein 4-like
pollen Ole e I family allergen
probable RNA-dependent RNA polymerase 1
dynamin-related protein 1C-like
nudix hydrolase 17, mitochondrial-like

For Review Only

MLP-like protein 328
rust resistance kinase Lr10
leucine-rich repeat extensin-like protein 6
MLP-like protein 34
KEH40549.1 plastid movement impaired protein
heavy metal-associated isoprenylated plant protein 22-like
RHN77924.1 putative alpha-L-fucosidase
probable receptor-like protein kinase At1g11050
bidirectional sugar transporter SWEET1
stearoyl-[acyl-carrier-protein] 9-desaturase 6, chloroplastic
proline-rich receptor-like protein kinase PERK10
glycolipid transfer protein 2
rust resistance kinase Lr10-like
---NA---
LEAF RUST 10 DISEASE-RESISTANCE LOCUS RECEPTOR-LIKE PROTEIN KINASE-like 2.5
protein DMR6-LIKE OXYGENASE 2-like
rust resistance kinase Lr10-like isoform X1
glutamate synthase [NADH], amyloplastic isoform X1
probable glycosyltransferase At5g03795
RCC1 domain-containing protein RUG3, mitochondrial-like
protein PEP-RELATED DEVELOPMENT ARRESTED 1, chloroplastic
exonuclease mut-7 homolog isoform X1
alpha/beta fold hydrolase
protein ABIL2-like isoform X2
uncharacterized protein At4g00950-like
KEH39742.1 transmembrane protein, putative
60S ribosomal protein l23
protein NRT1/ PTR FAMILY 1.2-like
ribosomal RNA small subunit methyltransferase-like
myb-like protein X
AES58911.1 transcriptional regulator of RNA polII, SAGA, subunit
plasma membrane ATPase 4
protein ACTIVITY OF BC1 COMPLEX KINASE 1, chloroplastic
flowering-promoting factor 1-like protein 2
protein arginine N-methyltransferase 1.5-like isoform X1
probable E3 ubiquitin-protein ligase RZFP34
AES58954.1 FAD/NAD(P)-binding oxidoreductase family protein
SPX domain-containing protein 2-like
uncharacterized protein LOC101491982
zinc finger protein 3
L-type lectin-domain containing receptor kinase IX.1-like
early nodulin-like protein 3
WAT1-related protein At5g40240-like isoform X1
probable WRKY transcription factor 40
CBL-interacting serine/threonine-protein kinase 25
RNA polymerase beta' subunit

For Review Only

probable monogalactosyldiacylglycerol synthase, chloroplastic
protein GLUTAMINE DUMPER 5-like
---NA---
KEH39908.1 DUF1677 family protein
serine/threonine-protein kinase RIPK
protein ALP1-like
uncharacterized protein LOC101503399
---NA---
beta-fructofuranosidase, insoluble isoenzyme CWINV3-like
pathogen-associated molecular patterns-induced protein A70
protein NDL2
vacuolar-processing enzyme-like isoform X1
homeotic protein knotted-1 isoform X1
ethylene-responsive transcription factor ABR1-like isoform X1
U-box domain-containing protein 4-like
3-oxo-Delta(4,5)-steroid 5-beta-reductase
ABD33278.1 Gibberellin regulated protein
probable isoaspartyl peptidase/L-asparaginase 2
AES59401.1 SPla/RYanodine receptor (SPRY) domain protein
BRI1 kinase inhibitor 1-like
probable protein phosphatase 2C 12
patatin-like protein 2
calcineurin-like metallo-phosphoesterase superfamily protein
serine carboxypeptidase II-3-like isoform X3
AES59499.1 DUF1645 family protein
allene oxide synthase 1, chloroplastic
---NA---
probable galacturonosyltransferase-like 1
KEH40130.1 erythronate-4-phosphate dehydrogenase family protein
probable LRR receptor-like serine/threonine-protein kinase At2g16250
sugar transporter ERD6-like 6
protein SHI RELATED SEQUENCE 5-like
lysine-specific histone demethylase 1 homolog 3 isoform X1
cytochrome P450 71A1-like
tRNA (adenine(58)-N(1))-methyltransferase non-catalytic subunit trm6
heavy metal-associated isoprenylated plant protein 39-like isoform X4
gibberellin-regulated protein 1-like precursor
endoplasmin homolog isoform X1
synaptotagmin-2-like isoform X1
DNA ligase 1-like isoform X1
expansin-A11
L-ascorbate oxidase homolog
4-coumarate--CoA ligase-like 5
---NA---
---NA---
---NA---

For Review Only

---NA---

uncharacterized protein LOC101492970

unknown

---NA---

hypothetical protein MtrunA17_Chr5g0393601

---NA---

orf109 (mitochondrion)

hypothetical protein TSUD_317270

aspartic proteinase-like protein 2 isoform X2

NADH dehydrogenase subunit 2 (mitochondrion)

---NA---

Ribosomal protein S10

---NA---

---NA---

---NA---

---NA---

---NA---

hypothetical protein MtrunA17_Chr1g0160311

---NA---

auxin-responsive protein SAUR50-like

uncharacterized protein LOC101511026

---NA---

hypothetical protein MtrunA17_Chr1g0151831

bZIP transcription factor 11-like

putative methylesterase 12, chloroplastic isoform X1

cellulose synthase-like protein G1

putative methyltransferase At1g22800, mitochondrial

endoglucanase 9-like

---NA---

cytosolic sulfotransferase 15-like

GPI-anchored protein LLG1-like

photosystem I reaction center subunit V, chloroplastic

WPP domain-interacting protein 2-like

hydroquinone glucosyltransferase-like

probable glucan 1,3-beta-glucosidase A

21 kDa protein-like

Monocopper oxidase protein SKS1

calcium permeable stress-gated cation channel 1-like

U-box domain-containing protein 35-like isoform X1

eugenol synthase 1-like isoform X1

glycine--tRNA ligase, mitochondrial 1-like

CTD small phosphatase-like protein 2

probable pectinesterase/pectinesterase inhibitor 25

AES68616.1 transmembrane protein, putative

disease resistance protein RPM1-like

---NA---

For Review Only

cytochrome P450 734A1-like isoform X1

probable diphthine methyl ester synthase isoform X2

glycerophosphodiester phosphodiesterase GDPDL4-like

transcription factor TGA2.2 isoform X6

AES68714.1 50S ribosomal protein L27

---NA---

trihelix transcription factor ASIL2 isoform X1

---NA---

putative disease resistance protein RGA4

chloride channel protein CLC-c-like

ribosomal protein S12C

Cytochrome c biogenesis CcmF C-terminal-like mitochondrial protein

TMV resistance protein N-like isoform X1

protein SUPPRESSOR OF npr1-1, CONSTITUTIVE 1-like

dirigent protein 22-like

dirigent protein 21

protein EARLY RESPONSIVE TO DEHYDRATION 15-like isoform X2

---NA---

basic leucine zipper 24

zinc finger CCCH domain-containing protein 53 isoform X1

pentatricopeptide repeat-containing protein At4g18975, chloroplastic isoform X4

triosephosphate isomerase 1 precursor

uncharacterized protein LOC101504502 isoform X1

short-chain dehydrogenase TIC 32, chloroplastic-like isoform X1

KEH17090.1 DUF642 family protein

photosystem II 5 kDa protein, chloroplastic-like

DIS3-exonuclease-like protein

myb family transcription factor PHL8-like isoform X1

phosphatidylinositol/phosphatidylcholine transfer protein SFH12-like isoform X1

transmembrane protein, putative

protein EMBRYO DEFECTIVE 514-like

PLASMODESMATA CALLOSE-BINDING PROTEIN 3-like isoform X2

pentatricopeptide repeat-containing protein At1g10910, chloroplastic-like

uncharacterized protein LOC101500340

GDSL esterase/lipase At1g74460-like

short-chain dehydrogenase TIC 32, chloroplastic-like isoform X2

---NA---

chlorophyll a-b binding protein P4, chloroplastic

cyclic dof factor 3-like

putative bark agglutinin LECRPA3

peptidyl-prolyl cis-trans isomerase NIMA-interacting 4

LON peptidase N-terminal domain and RING finger protein 1

---NA---

ABC transporter A family member 7-like

nudix hydrolase 3

hypothetical protein ES319_D08G073700v1

For Review Only

E3 ubiquitin-protein ligase MARCH7-like
stomatal closure-related actin-binding protein 1-like
F-box protein At2g26850-like
G-type lectin S-receptor-like serine/threonine-protein kinase At4g27290 isoform X1
serine carboxypeptidase-like 45
probable terpene synthase 2
integrator complex subunit 7 homolog isoform X2
putative photosystem II oxygen-evolving complex protein PsbP-1
2-hydroxyisoflavanone dehydratase-like
protein JASON-like isoform X2
cytochrome b561 domain-containing protein At2g30890-like
glucan endo-1,3-beta-glucosidase 12-like
AES70126.1 peptidase
---NA---
probable galacturonosyltransferase 15 isoform X1
spermidine hydroxycinnamoyl transferase-like
probable folate-biopterin transporter 4
AES70332.1 DUF4228 domain protein
calcium-dependent protein kinase 2-like
protein CLT3, chloroplastic-like
Senescence regulator S40
small heat shock protein, chloroplastic-like
hypothetical protein TSUD_198970
---NA---
ethylene-responsive transcription factor 7-like
putative lipid-transfer protein DIR1
glycerophosphodiester phosphodiesterase GDPDL3
disease resistance protein RPM1
Glycine-rich RNA-binding protein
classical arabinogalactan protein 9-like
nudix hydrolase 10-like
LEAF RUST 10 DISEASE-RESISTANCE LOCUS RECEPTOR-LIKE PROTEIN KINASE-like 1.4
heat shock cognate protein 80
ribosomal protein S4 (mitochondrion)
hypothetical protein (mitochondrion)
LRR receptor-like serine/threonine-protein kinase
heavy metal-associated isoprenylated plant protein 41-like
chaperonin-like RbcX protein 2, chloroplastic
uncharacterized protein LOC101509734
serine/arginine-rich splicing factor SR45a
transcription factor DIVARICATA-like
zinc finger family protein
non-specific phospholipase C1
hypothetical protein TSUD_126650
protein SMAX1-LIKE 6 isoform X1
gibberellin 2-beta-dioxygenase 8

For Review Only

ethylene-overproduction protein 1
ubiquitin-activating enzyme E1 1-like
protein LIGHT-DEPENDENT SHORT HYPOCOTYLS 5
uncharacterized protein LOC101514590
LOB domain-containing protein 12
probable protein phosphatase 2C 26 isoform X2
AES69531.1 DUF4228 domain protein
cationic peroxidase 1-like
phosphatidate cytidylyltransferase 4, chloroplastic-like
CBL-interacting serine/threonine-protein kinase 10
CBL-interacting serine/threonine-protein kinase 11-like
non-specific lipid-transfer protein 1-like
probable glutathione S-transferase parA
probable LRR receptor-like serine/threonine-protein kinase At1g06840 isoform X2
60S ribosomal protein L10-like
metal tolerance protein 4-like isoform X1
---NA---
bifunctional bis(5'-adenosyl)-triphosphatase/adenylylsulfatase FHIT-like
pentatricopeptide repeat-containing protein At4g25270, chloroplastic
cysteine-rich receptor-like protein kinase 3
cold-responsive protein kinase 1-like
cytochrome P450 90A1-like
probable galactinol--sucrose galactosyltransferase 1
protein EARLY FLOWERING 4
CTD nuclear envelope phosphatase 1 homolog
taxadien-5-alpha-ol O-acetyltransferase
LOB domain-containing protein 12-like
---NA---
ethylene-responsive transcription factor ERF014
flavonol synthase/flavanone 3-hydroxylase
nucleic acid binding protein
oxysterol-binding protein-related protein 1C-like
putative galactosylxylosylprotein 3-beta-galactosyltransferase
uncharacterized protein LOC101511967
heavy metal-associated isoprenylated plant protein 24
inactive TPR repeat-containing thioredoxin TTL3
sugar transport protein 5
transcription factor MYB93
protein EXORDIUM-like
nucleotide pyrophosphatase/phosphodiesterase
---NA---
uncharacterized protein LOC101514803 isoform X3
probable inactive poly [ADP-ribose] polymerase SRO2
polygalacturonase 1 beta-like protein 3
probable alpha,alpha-trehalose-phosphate synthase [UDP-forming] 9 isoform X1
nucleobase-ascorbate transporter 6

For Review Only

uncharacterized protein LOC101505135
putative alpha-N-acetylglucosaminidase
putative transferase At4g12130, mitochondrial
L-ascorbate oxidase
NDR1/HIN1-like protein 13
putative plastidic glucose transporter 1-like protein
---NA---
oligopeptide transporter 7-like
B2 protein-like
OTU domain-containing protein DDB_G0284757
repetitive proline-rich cell wall protein
---NA---
uncharacterized protein LOC101494494 isoform X1
putative E3 ubiquitin-protein ligase XBAT31
NAD(P)H-dependent 6'-deoxychalcone synthase-like
phytoene synthase 2, chloroplastic-like isoform X1
DNA repair protein RAD50
protein transport protein SEC23
probable methyltransferase At1g27930
F-box protein At1g67340-like
F-box/kelch-repeat protein
uncharacterized protein LOC101491577
serine/threonine protein phosphatase 2A 57 kDa regulatory subunit B' theta isoform
very-long-chain 3-oxoacyl-CoA reductase 1-like
putative nitronate monooxygenase-like protein
sufE-like protein 2, chloroplastic
probable E3 ubiquitin-protein ligase XERICO
ATP-dependent RNA helicase DEAH11, chloroplastic
homeobox-leucine zipper protein ATHB-6-like
LMBR1 domain-containing protein 2 homolog A
lanC-like protein GCL1
uncharacterized protein LOC101495892
uncharacterized protein LOC101495563
sulfate transporter 2.1-like isoform X1
hypothetical protein glysoja_022994
probable E3 ubiquitin-protein ligase XERICO
transcription factor TGA4-like isoform X1
NAC family transcription factor 5
protein disulfide-isomerase
delta-aminolevulinic acid dehydratase, chloroplastic
beta-galactosidase 16-like
AES72307.1 proteasome assembly chaperone-like protein
chloroplast processing peptidase
probable receptor-like serine/threonine-protein kinase At5g57670
probable calcium-binding protein CML44
2-oxoisovalerate dehydrogenase subunit alpha 2, mitochondrial-like

For Review Only

proline-rich receptor-like protein kinase PERK14 isoform X2
ACT domain-containing protein ACR3
scarecrow-like protein 1
alcohol dehydrogenase 1
alcohol dehydrogenase 1-like
oxidoreductase protein
protein MKS1
pathogenesis-related genes transcriptional activator PTI6-like
transcription factor SPEECHLESS
uncharacterized protein LOC101491306
probable pectinesterase/pectinesterase inhibitor 34
protein phosphatase 2C 57 isoform X1
dof zinc finger protein DOF4.6-like isoform X1
rhodanese-like domain-containing protein 14, chloroplastic
AAA-ATPase At2g46620-like
small heat shock protein, chloroplastic
StAR-related lipid transfer protein
uncharacterized protein LOC101513070
transcription termination factor MTERF8, chloroplastic
two-component response regulator-like APRR5 isoform X1
NDR1/HIN1-like protein 26
protein DOWNY MILDEW RESISTANCE 6
uncharacterized protein LOC101494813
60S ribosomal protein L10
putative F-box protein At3g16210
probable chalcone--flavonone isomerase 3
Expansin-A8 protein
potassium transporter 2 isoform X1
hypothetical protein glysoja_038315
peroxidase P7-like
transmembrane protein, putative
probable WRKY transcription factor 13
WAT1-related protein At5g64700
serine/threonine-protein kinase ATG1t
AES72809.1 TLD-domain nucleolar protein
mitogen-activated protein kinase kinase kinase 3
uncharacterized protein LOC101504183 isoform X1
probable methyltransferase PMT26
proline-rich receptor-like protein kinase PERK14
NAC domain-containing protein 2
TOM1-like protein 5
defensin-like protein 1 isoform X1
RZC07830.1 Calnexin-like isoform A
transcription factor bHLH137
metal tolerance protein 4 isoform X2
probable serine/threonine-protein kinase At1g54610

For Review Only

SAUR-like auxin-responsive family protein
F-box protein At1g78280
14-3-3-like protein isoform X1
alcohol dehydrogenase-like 2
classical arabinogalactan protein 9
leucine-rich repeat extensin-like protein 3
putative F-box/FBD/LRR-repeat protein At1g78760
---NA---
myb-like DNA-binding domain protein
MADS-box protein SVP-like isoform X1
AT-hook motif nuclear-localized protein 17
aspartyl protease AED3
14-3-3-like protein B
transcription factor MYB1-like
RHN70256.1 putative cupredoxin
patatin-like protein 2
probable protein phosphatase 2C 12 isoform X2
THO complex subunit 5A
AES73331.1 SPla/RYanodine receptor (SPRY) domain protein
probable isoaspartyl peptidase/L-asparaginase 2
putative receptor protein kinase ZmPK1
hypothetical protein DVH24_012528
cytochrome b5
interferon-related developmental regulator 1
lecithin-cholesterol acyltransferase-like 1
KEH35844.1 DUF4378 domain protein
AP2-like ethylene-responsive transcription factor ANT
probable xyloglucan glycosyltransferase 12
branched-chain-amino-acid aminotransferase 2, chloroplastic isoform X1
homeobox-leucine zipper protein HAT9-like
zinc finger CCCH domain-containing protein 20-like
probable ADP,ATP carrier protein At5g56450
heat shock factor protein HSF30
cytochrome P450 711A1-like
---NA---
putative photosystem II
copper-transporting ATPase PAA2, chloroplastic
serine/threonine-protein kinase RIPK-like isoform X2
protein GLUTAMINE DUMPER 5
phospholipase D Z-like
kunitz-type elastase inhibitor BrEI-like precursor
hypothetical protein L195_g031169
auxin-responsive protein IAA29
G-type lectin S-receptor-like serine/threonine-protein kinase SD2-5
SPX domain-containing protein 2-like isoform X1
protein OVEREXPRESSOR OF CATIONIC PEROXIDASE 3

For Review Only

EG45-like domain containing protein
pleiotropic drug resistance protein 1-like
protein starmaker-like
potassium channel KAT3 isoform X2
protein FAM32A-like
F-box protein AFR
uncharacterized protein LOC101494693
probable sarcosine oxidase
auxin-responsive protein SAUR71-like
bZIP transcription factor RISBZ4-like
two-on-two hemoglobin-3-like
bark storage protein A-like
Protein kinase
carotenoid cleavage dioxygenase 8 homolog B, chloroplastic isoform X1
homeobox-leucine zipper protein ATHB-8 isoform X1
monoglyceride lipase
NADP-dependent malic enzyme
receptor-like protein kinase HSL1
hypothetical protein MtrunA17_Chr3g0141221
elongation of fatty acids protein 3-like
KEH36160.1 fatty acyl-CoA synthetase family protein
uncharacterized protein LOC101501386
ubiquitin-protein ligase
cytokinin riboside 5'-monophosphate phosphoribohydrolase LOG3-like
AES74020.1 DUF668 family protein
thaumatin-like protein 1b isoform X3
uncharacterized protein LOC101503743
AES74086.1 syringolide-induced protein 14-1-1
---NA---
protein NLP4 isoform X1
WUSCHEL-related homeobox 8-like isoform X1
cysteine protease XCP2-like precursor
MATH and LRR domain-containing protein PFE0570w
isoflavone reductase homolog PCBER-like
AES74211.1 transmembrane protein, putative
theobromine synthase 2-like
B-box zinc finger protein 20-like
AES74273.1 calcium-binding EF-hand protein
aquaporin PIP2-2
uncharacterized protein LOC101512317 isoform X1
uncharacterized protein LOC101513800
protein LAZ1
Alpha-1,3-mannosyl-glycoprotein 2-beta-N-acetylglucosaminyltransferase
chlorophyll a-b binding protein CP29.3, chloroplastic
arginyl-tRNA--protein transferase 2-like
SUN domain-containing protein 4-like

For Review Only

endoglucanase 9-like
zinc finger SWIM domain-containing protein 7-like
uncharacterized protein LOC101497082
WPP domain-interacting protein 2-like
hydroquinone glucosyltransferase-like
UDP-glycosyltransferase 88F5
---NA---
trehalose-phosphate phosphatase A-like isoform X1
phospholipid-transporting ATPase 1-like
probable diphthine methyl ester synthase isoform X2
NAD(P)H-dependent 6'-deoxychalcone synthase-like
cytosolic sulfotransferase 15-like
VQ motif protein
putative nepenthesin
---NA---
transmembrane protein, putative
NADH dehydrogenase subunit 6 (mitochondrion)
Cytochrome c biogenesis CcmF C-terminal-like mitochondrial protein
protein LNK2 isoform X3
---NA---
---NA---
polyamine oxidase 1
ent-kaurenoic acid oxidase 2
protein DETOXIFICATION 43-like
poly(ADP-ribose) glycohydrolase 1-like
DNA-damage-repair/toleration protein DRT100-like precursor
Signal peptidase complex subunit 3B
ADP,ATP carrier protein 1, mitochondrial-like
probable arabinosyltransferase ARAD1
transmembrane protein, putative
transmembrane protein, putative
putative bark agglutinin LECRPA3
putative pentatricopeptide repeat-containing protein At1g74400
---NA---
plant intracellular Ras-group-related LRR protein 7
---NA---
multicystatin-like
F-box/kelch-repeat protein At5g26960
Holliday junction resolvase MOC1, chloroplastic isoform X2
non-specific phospholipase C2
G-type lectin S-receptor-like serine/threonine-protein kinase At4g27290 isoform X1
probable polyamine transporter At1g31830
KEH33548.1 DUF4228 domain protein
---NA---
serine--glyoxylate aminotransferase isoform X2
chromodomain helicase DNA-binding protein, putative

For Review Only

integrator complex subunit 7 homolog isoform X2
ribose-phosphate pyrophosphokinase 4
hypothetical protein MtrunA17_Chr3g0097921
reactive Intermediate Deaminase A, chloroplastic isoform X2
cytochrome c biogenesis C (mitochondrion)
uncharacterized protein LOC105852272
---NA---
KEH33913.1 extracellular ligand-gated ion channel protein
uncharacterized protein LOC101500757
indole-3-acetate O-methyltransferase 1
---NA---
ribonuclease S-F11-like
3-ketoacyl-CoA thiolase 2, peroxisomal-like
cytochrome P450 71D10-like
tabersonine 16-hydroxylase 1-like
cytochrome P450 71D9
cytochrome P450 71D11-like
serine acetyltransferase 1, chloroplastic-like
30S ribosomal protein S10, chloroplastic
TMV resistance protein N-like isoform X1
nudix hydrolase 10-like
---NA---
---NA---
protein SUPPRESSOR OF GENE SILENCING 3 homolog isoform X2
BURP domain protein USPL1-like
lysM domain-containing GPI-anchored protein 1-like
heat shock cognate protein 80
BTB/POZ domain-containing protein At1g55760
chaperone protein dnaJ 11, chloroplastic-like
chaperone protein dnaJ 11, chloroplastic-like
cyclin-dependent protein kinase inhibitor SMR9
calmodulin-binding receptor-like cytoplasmic kinase 2
PREDICTED: uncharacterized protein LOC107874440
protein NRT1/ PTR FAMILY 8.2-like
E3 ubiquitin-protein ligase AIRP2 isoform X1
protein PSY1
putative LRR receptor-like protein kinase
amino acid transporter AVT1I
putative receptor protein kinase ZmPK1
uncharacterized protein LOC101489310
probable WRKY transcription factor 26
alpha-expansin 2 precursor
NADH dehydrogenase subunit 5 (mitochondrion)
ribosomal protein S1 (mitochondrion)
uncharacterized protein LOC101506096 isoform X1
probable inactive receptor kinase At5g58300

For Review Only

calcium uniporter protein 5, mitochondrial-like isoform X3
aspartyl protease family protein 2-like
CRM-domain containing factor CFM3, chloroplastic/mitochondrial-like isoform X2
probable helicase MAGATAMA 3
AES71131.1 alpha/beta hydrolase-like protein
putative serine/threonine-protein kinase-like protein CCR3
CTD nuclear envelope phosphatase 1 homolog
probable 2-oxoglutarate-dependent dioxygenase At5g05600
LOB domain-containing protein 21-like
UTP--glucose-1-phosphate uridylyltransferase 3, chloroplastic
inactive receptor-like serine/threonine-protein kinase At2g40270
glutaredoxin domain-containing cysteine-rich protein
DEAD-box ATP-dependent RNA helicase 18
protein trichome birefringence-like 33
abscisic acid receptor PYL4-like
putative laccase
senescence associated gene 20
BTB/POZ domain-containing protein At3g56230
AES71277.1 hypoxia-responsive family protein
WEB family protein At2g40480
WAT1-related protein At4g08290-like isoform X1
WAT1-related protein At4g08290-like
MORN repeat-containing protein 1-like
GRAS family protein
AES71342.1 nucleic acid-binding protein
oxysterol-binding protein-related protein 1C-like
beta-1,3-galactosyltransferase 7 isoform X1
uncharacterized protein LOC101513911
low affinity sulfate transporter 3-like
---NA---
FCS-Like Zinc finger 5-like
ethylene-responsive transcription factor RAP2-4
inactive TPR repeat-containing thioredoxin TTL3
sugar transport protein 5-like
signal peptidase complex-like protein DTM1
calcium-binding protein CML38-like
probable inactive leucine-rich repeat receptor-like protein kinase At3g03770 isoform X2
BTB/POZ and TAZ domain-containing protein 1-like
probable pectate lyase 5
peptide methionine sulfoxide reductase-like
KEH35007.1 KDEL-tailed cysteine endopeptidase CEP1
leucine-rich repeat receptor-like serine/threonine/tyrosine-protein kinase SOBIR1 isoform X1
probable galactinol--sucrose galactosyltransferase 2
protein DEHYDRATION-INDUCED 19 homolog 2-like isoform X2
phloem specific protein
uncharacterized protein LOC101511445

For Review Only

tryptophan aminotransferase-related protein 2-like
galactinol--sucrose galactosyltransferase-like
hypothetical protein PHAVU_005G172700g
---NA---
---NA---
protein SUPPRESSOR OF npr1-1, CONSTITUTIVE 1
disease resistance protein RML1A-like
metal tolerance protein 10-like
methionine gamma-lyase-like
heat shock 70 kDa protein 17
protein TIFY 5B-like
choline-phosphate cytidylyltransferase 2
heavy metal-associated isoprenylated plant protein 16-like
---NA---
zinc finger protein CONSTANS-LIKE 9
uncharacterized protein LOC101500977
NAD(P)H-dependent 6'-deoxychalcone synthase-like
1-aminocyclopropane-1-carboxylate oxidase
titin-like
IQ domain-containing protein IQM2-like
protein YLS3
purple acid phosphatase 15-like isoform X1
chalcone synthase 1B
receptor-like kinase TMK4
brassinosteroid-regulated protein bru1-like protein
rho GDP-dissociation inhibitor 1-like
amino acid transporter AVT1H
CAG14984.1 putative lipid transfer protein GPI-anchored
REF/SRPP-like protein At1g67360
E3 ubiquitin-protein ligase mib1-like
photosystem II core complex proteins psbY, chloroplastic-like
putative clathrin assembly protein At4g40080
homeobox-leucine zipper protein ATHB-6-like
Ras-related protein RABH1d
uncharacterized protein LOC101495892
uncharacterized protein LOC101495242
uncharacterized protein LOC101495563
ABC transporter D family member 1
protein DOWNSTREAM OF FLC
FCS-Like Zinc finger 7
heavy metal-associated isoprenylated plant protein 23-like
actin cytoskeleton-regulatory complex protein PAN1-like
glutathione hydrolase 1
photosystem II 22 kDa protein, chloroplastic
aldehyde dehydrogenase family 3 member H1-like isoform X1
two-component response regulator ARR17-like

For Review Only

IAA-amino acid hydrolase ILR1-like 6
metal transporter Nramp5
uncharacterized protein LOC101496117
probable methyltransferase PMT25 isoform X1
meiotic nuclear division protein 1 homolog isoform X2
transcription factor MYB36-like
uncharacterized protein LOC101509525
ribosome biogenesis protein NSA2 homolog
basic blue protein-like
glycolipid transfer protein 2-like
probable inactive receptor kinase At4g23740
probable WRKY transcription factor 53
spermine synthase-like isoform X1
calcineurin B-like protein 7 isoform X1
AES72528.1 transmembrane protein, putative
KEH35466.1 transmembrane protein, putative
protein yippee-like At4g27745
molybdate-anion transporter-like
AAA-ATPase At2g46620-like
homeobox-leucine zipper protein ATHB-12-like
CDPK-related kinase 4-like
AES72604.1 DUF4228 domain protein
polyadenylate-binding protein-interacting protein 12-like
AES72623.1 membrane-related protein CP5, putative
two-component response regulator-like APRR9 isoform X3
uncharacterized protein LOC101488650
protein TRANSPARENT TESTA GLABRA 1
peroxidase 5-like
methylesterase 17
uncharacterized protein LOC101505139
transmembrane protein, putative
nucleotide/sugar transporter family protein
uncharacterized protein LOC101491685 isoform X2
hypothetical protein, partial
probable WRKY transcription factor 43
brassinosteroid LRR receptor kinase
cellulose synthase A catalytic subunit 2 [UDP-forming]-like
AES72792.1 transmembrane protein, putative
photosystem I reaction center subunit N, chloroplastic
RING-H2 finger protein ATL78
UDP-glucuronate:xylan alpha-glucuronosyltransferase 1 isoform X2
probable pectinesterase/pectinesterase inhibitor 51
cell wall / vacuolar inhibitor of fructosidase 2
probable polygalacturonase
U-box domain-containing protein 25-like
transmembrane protein, putative

For Review Only

beta-galactosidase 10
AES72991.1 electron transporter
ethylene-responsive transcription factor ERF114-like
metal tolerance protein 4-like isoform X2
NAC domain-containing protein 83
RHN70124.1 putative molybdenum cofactor sulfurtransferase
TITAN-like protein isoform X2
14-3-3-like protein isoform X1
hypothetical protein TSUD_317170
transcription factor bHLH96-like isoform X1
double-stranded RNA-binding protein 1-like
zinc-finger homeodomain protein 2-like
protein NRT1/ PTR FAMILY 4.5
transcription factor MYB1-like
protein MIZU-KUSSEI 1
uroporphyrinogen-III synthase, chloroplastic
coenzyme Q-binding protein COQ10 homolog B, mitochondrial
heat stress transcription factor B-4
uncharacterized acetyltransferase At3g50280
uncharacterized acetyltransferase At3g50280-like
ethylene-responsive transcription factor RAP2-1-like
probable carbohydrate esterase At4g34215
subtilisin-like protease SBT1.7
zinc finger protein ZF1
---NA---
KEH35826.1 transmembrane protein, putative
zinc finger CCCH domain-containing protein 20-like
zinc transporter 4, chloroplastic-like isoform X1
KYP70326.1 Glutaredoxin-C6
probable WRKY transcription factor 50 isoform X1
heat shock protein HSP17.6-II-like
solute carrier family 25 member 44
AES73480.1 alpha/beta fold hydrolase
uncharacterized protein LOC101497093
ethylene-responsive transcription factor ERF017-like
early nodulin-like protein 1
probable WRKY transcription factor 40
26S proteasome non-ATPase regulatory subunit 12 homolog A-like
chaperone protein ClpB4, mitochondrial
protein PPLZ02-like
kunitz-type elastase inhibitor BrEI-like precursor
zinc finger Ran-binding domain-containing protein 2-like
probable boron transporter 7 isoform X1
probable protein phosphatase 2C 22
molybdate transporter 1
potassium channel KAT3 isoform X1

For Review Only

probable WRKY transcription factor 17
uncharacterized protein LOC101510277
RHN70844.1 putative alpha/Beta hydrolase
RHN70850.1 putative aminoacyltransferase, E1 ubiquitin-activating enzyme
tocopherol cyclase, chloroplastic
zeaxanthin 7,8(7',8')-cleavage dioxygenase, chromoplastic-like isoform X1
lachrymatory-factor synthase-like
class V chitinase
NADP-dependent malic enzyme-like
serine/threonine-protein kinase Aurora-1 isoform X2
amino acid permease 6-like
receptor protein-tyrosine kinase CEPR1
uncharacterized protein At5g65660-like
transcription factor bHLH93-like
probable galacturonosyltransferase-like 1
hypothetical protein MTR_3g111650
protein RADIALIS-like 4
isoflavone reductase-like protein
KEH36169.1 transmembrane protein, putative
abscisic acid 8'-hydroxylase 4
bifunctional nuclease 1
mitogen-activated protein kinase homolog NTF3
---NA---
uncharacterized protein LOC101505557 isoform X1
CO(2)-response secreted protease-like
---NA---
ribonuclease H
24-methylenesterol C-methyltransferase 2
plastocyanin B'/B''-like
ornithine decarboxylase
L-ascorbate oxidase homolog
uncharacterized protein LOC101490725
probable serine/threonine-protein kinase PIX13 isoform X1
glyceraldehyde-3-phosphate dehydrogenase B, chloroplastic
BURP domain protein USPL1-like
MATH and LRR domain-containing protein PFE0570w
glucan endo-1,3-beta-D-glucosidase
heavy metal-associated isoprenylated plant protein 26
plant phospholipase-like protein
exportin-7-B isoform X1
MLO-like protein 1
uncharacterized protein LOC101512317 isoform X2
class I chitinase precursor
---NA---
---NA---
---NA---

For Review Only

uncharacterized protein LOC101489324

---NA---

---NA---

hypothetical protein TanjilG_14324

hypothetical protein MTR_3g027030

hypothetical protein MtrunA17_Chr3g0087341

hypothetical protein MtrunA17_Chr3g0087911

---NA---

hypothetical protein L195_g031969, partial

---NA---

---NA---

---NA---

hypothetical protein MtrunA17_Chr3g0096231

conserved hypothetical protein

---NA---

PREDICTED: uncharacterized protein LOC109122063

---NA---

uncharacterized protein LOC101492457 isoform X4

---NA---

---NA---

---NA---

---NA---

hypothetical protein MtrunA17_Chr3g0134371

---NA---

---NA---

auxin-induced protein 15A-like

uncharacterized protein DDB_G0271670-like

probable polyamine oxidase 4

expansin-a13-like protein

glutamate decarboxylase 1-like

U-box domain-containing protein 21

aspartic proteinase PCS1

nudix hydrolase 18, mitochondrial-like

transcription factor MYB20-like

AES70969.2 calmodulin-binding heat-shock protein

DELLA protein GAI

E3 ubiquitin-protein ligase SDIR1 isoform X1

protein phosphatase 2C 37

ultraviolet-B receptor UVR8-like

wall-associated receptor kinase-like 20

---NA---

uncharacterized protein LOC101504080

purple acid phosphatase 3 isoform X1

uncharacterized protein LOC101493228

thaumatin-like protein

probable calcium-binding protein CML27

For Review Only

golgin candidate 2 isoform X1
AET05524.1 late embryogenesis abundant protein
nuclear poly(A) polymerase 1-like isoform X1
uncharacterized protein LOC101509529
disease resistance-responsive dirigent domain protein
probable glutathione peroxidase 3, mitochondrial-like
---NA---
adagio protein 3
nudix hydrolase 18, mitochondrial-like
protein TIFY 6B-like isoform X1
protein SAR DEFICIENT 1-like isoform X1
LEAF RUST 10 DISEASE-RESISTANCE LOCUS RECEPTOR-LIKE PROTEIN KINASE-like 2.3
phosphoglucomutase, cytoplasmic
endoglucanase 8
uncharacterized protein LOC101513508
arabinogalactan protein
uncharacterized protein LOC101493656
uncharacterized protein LOC101509321
probable protein phosphatase 2C 6
---NA---
ATP-dependent 6-phosphofructokinase 3-like
uncharacterized protein LOC101492105
vacuolar protein sorting-associated protein 32 homolog 2-like
auxin-induced protein 6B
uncharacterized protein LOC101502258
putative GEM-like protein 8
---NA---
transmembrane protein, putative
B2 protein-like
phosphatidylinositol 4-kinase gamma 2-like
uncharacterized protein LOC101498634 isoform X1
long chain acyl-CoA synthetase 4-like
subtilisin-like protease SBT2.5
uncharacterized protein LOC101502259
probable WRKY transcription factor 41
disease resistance response protein 206
protein LURP-one-related 17
1-aminocyclopropane-1-carboxylate synthase
E3 ubiquitin-protein ligase RHA1B
BTB/POZ domain-containing protein At5g41330
uncharacterized protein LOC101497539 isoform X2
tubulin beta-1 chain
protein LURP-one-related 4
tetraspanin-10 isoform X2
hypothetical protein CR513_29970, partial
thaumatin-like pathogenesis-related protein 4-like precursor

For Review Only

bifunctional UDP-glucose 4-epimerase and UDP-xylose 4-epimerase 1
uncharacterized protein LOC101506441
NAC domain-containing protein 83
acyl carrier protein 1, chloroplastic isoform X1
---NA---
uncharacterized protein LOC113855720
AET04880.1 Zn-dependent alcohol dehydrogenase family protein
RING/FYVE/PHD zinc finger superfamily protein
amino acid permease 4-like
NAC transcription factor 25-like
---NA---
probable pectinesterase/pectinesterase inhibitor 51
AET04723.1 DUF506 family protein
RING-H2 finger protein ATL78-like
RING-H2 finger protein ATL78-like
probable mannitol dehydrogenase
hypothetical protein glysoja_008476
ethylene-responsive transcription factor ABR1-like
ubiquitin carboxyl-terminal hydrolase 27 isoform X1
UDP-rhamnose/UDP-galactose transporter 4-like
protein CELLULOSE SYNTHASE INTERACTIVE 1
nitrate regulatory gene2 protein
dehydration responsive element binding protein
probable trehalose-phosphate phosphatase J
PLAT domain-containing protein 3
protein spinster-like
KEH16213.1 DUF4057 family protein
ras-related protein RABH1e-like
calmodulin-binding transcription activator 1
protein SENSITIVE TO PROTON RHIZOTOXICITY 1-like
UDP-URONIC ACID TRANSPORTER 1-like
---NA---
serine/threonine-protein kinase SAPK2 isoform X1
KEH20776.1 beta-1,4-N-acetylglucosaminyltransferase-like protein
E3 ubiquitin-protein ligase RGLG5-like isoform X1
AET04507.1 CAAX amino terminal protease family protein
Basic blue protein
protein BPS1, chloroplastic
ferredoxin, leaf L-A
uncharacterized protein LOC101502040
uncharacterized protein LOC101505243 isoform X1
ubiquitin 5
GDSL esterase/lipase At1g29670-like
fasciclin-like arabinogalactan protein 12
uncharacterized protein LOC101494080
cellulose synthase A catalytic subunit 8 [UDP-forming]

For Review Only

uncharacterized protein LOC113787212

Mitochondrial dicarboxylate/tricarboxylate transporter DTC

bZIP transcription factor 44-like

peptidyl-tRNA hydrolase, mitochondrial isoform X2

KFB-type F-box protein

UV radiation resistance associated protein isoform X1

70 kDa peptidyl-prolyl isomerase-like

15-cis-zeta-carotene isomerase, chloroplastic

probable DNA primase large subunit isoform X2

F-box/kelch-repeat protein At1g67480

uncharacterized protein LOC101501830

transcription factor TCP2-like

uncharacterized protein At4g22758

RHN42375.1 putative small subunit of serine palmitoyltransferase

putative receptor-like protein kinase At4g00960 isoform X2

zinc finger protein 4-like

purple acid phosphatase 8

protein SRG1-like

probable 2-oxoglutarate/Fe(II)-dependent dioxygenase

zinc finger protein SHOOT GRAVITROPISM 5

protein SLOW GREEN 1, chloroplastic

transcription factor EAT1-like

phosphatidate cytidylyltransferase, mitochondrial isoform X2

RING-H2 finger protein ATL73-like

1-Cys peroxiredoxin isoform X1

cytochrome b561 domain-containing protein At4g18260

LRR receptor-like serine/threonine-protein kinase FLS2

small RNA 2'-O-methyltransferase

dihydroceramide fatty acyl 2-hydroxylase FAH2

chaperone protein dnaJ 20, chloroplastic-like

calmodulin-binding transcription activator 2-like isoform X2

probable protein phosphatase 2C 9

outer envelope protein 64, mitochondrial

7-deoxyloganetin glucosyltransferase

protein EXORDIUM-like 2

bifunctional riboflavin biosynthesis protein RIBA 1, chloroplastic-like

---NA---

protein DMR6-LIKE OXYGENASE 2-like

AES90613.1 transmembrane protein, putative

hypothetical protein TSUD_65690

coiled-coil-helix-coiled-coil-helix domain-containing protein 10, mitochondrial

---NA---

squalene epoxidase

protein HEAT-STRESS-ASSOCIATED 32

lysine-specific demethylase JMJ25-like isoform X1

uncharacterized protein LOC25493221

For Review Only

serine carboxypeptidase-like 31
KEH31059.1 plant/F8B4-180 protein
MACPF domain-containing protein NSL1
18.5 kDa class I heat shock protein-like
18.5 kDa class I heat shock protein-like
FHA domain-containing protein At4g14490-like
cellulose synthase-like protein G2
isoflavone 4'-O-methyltransferase-like
2-hydroxyisoflavanone synthase-like
---NA---
GDSL esterase/lipase At5g45960-like
(+)-neomenthol dehydrogenase isoform X1
CCG-binding protein 1
kinesin-like protein KIN-12D isoform X1
uncharacterized protein LOC101490629
uncharacterized protein LOC101490954
putative cupredoxin
Nodulation protein H
probable carboxylesterase 12
probable carboxylesterase 12
cyclin-dependent kinase inhibitor 4
uncharacterized protein LOC101496349
cell number regulator 2-like isoform X1
protein CYPRO4
PI-PLC X domain-containing protein At5g67130
40S ribosomal protein S16-like
polyadenylate-binding protein 2
scopoletin glucosyltransferase
DnaJ heat shock amino-terminal domain protein
formin-like protein 10
pentatricopeptide repeat-containing protein At2g01860
uncharacterized protein LOC101489326 isoform X1
transcription termination factor family protein
10 kDa chaperonin, mitochondrial
phytanoyl-CoA dioxygenase isoform X1
calcyclin-binding protein-like
protein NARROW LEAF 1-like isoform X2
probable inorganic phosphate transporter 1-9
zinc finger protein ZPR1-like isoform X1
BOI-related E3 ubiquitin-protein ligase 1-like
UDP-glycosyltransferase 708D1
probable WRKY transcription factor 4 isoform X2
integrin-linked protein kinase 1
TPR domain protein
alpha-expansin 4 precursor
myb family transcription factor PHL8-like

For Review Only

G-type lectin S-receptor-like serine/threonine-protein kinase
uncharacterized protein LOC101513513
dihydroflavonol 4-reductase
thioredoxin-like protein CXXS1
RING finger protein
senescence-specific cysteine protease SAG39-like isoform X1
TMV resistance protein N
probable alpha,alpha-trehalose-phosphate synthase [UDP-forming] 7
phospholipase A1-IIdelta
F-box protein MAX2
DUF724 domain-containing protein 2-like
phosphoenolpyruvate carboxylase
senescence-specific cysteine protease SAG39-like
protein trichome birefringence-like 40 isoform X1
BnaCnng12640D
ras-related protein RABA1f
lysine-specific histone demethylase-like protein
zinc finger protein MAGPIE-like
uncharacterized protein LOC101510728
geraniol 8-hydroxylase-like
TOM1-like protein 1
cellulose synthase-like protein D3
phospholipase A1-Igamma3, chloroplastic
glucan endo-1,3-beta-glucosidase
beta-adaptin-like protein C
L-ascorbate oxidase homolog
translocon-associated protein subunit alpha-like
proline-rich protein 4-like
serine carboxypeptidase-like 34
H/ACA ribonucleoprotein complex subunit 4
gibberellin 2-beta-dioxygenase 6-like
eukaryotic translation initiation factor 4B3 isoform X1
dirigent protein 23-like
putative disease resistance RPP13-like protein 3
subtilisin-like protease SBT1.6
protein EARLY-RESPONSIVE TO DEHYDRATION 7, chloroplastic-like
probable receptor-like protein kinase At3g17420
shikimate O-hydroxycinnamoyltransferase-like
lamin-like protein
cinnamoyl-CoA reductase 2-like
uncharacterized protein LOC101488406 isoform X2
putative UPF0481 protein At3g02645
VQ motif-containing protein 11
probable zinc metallopeptidase EGY3, chloroplastic
protein LURP-one-related 12-like
methyl-CpG-binding domain-containing protein 11 isoform X1

For Review Only

AES86605.1 transmembrane protein, putative

KEH28697.1 lactoylglutathione lyase/glyoxalase I family protein

peptide deformylase 1A, chloroplastic-like

molybdate transporter 2

---NA---

pleiotropic drug resistance protein 1

probable 2-oxoglutarate-dependent dioxygenase AOP1

malonate--CoA ligase-like

protein TIC 20, chloroplastic isoform X2

probable sulfate transporter 3.4

mitochondrial arginine transporter BAC2

protein SCAR2 isoform X1

probable WRKY transcription factor 33

macrophage migration inhibitory factor homolog

PRA1 family protein B4-like

probably inactive leucine-rich repeat receptor-like protein kinase IMK2

ferritin-3, chloroplastic

AES86816.1 laccase/diphenol oxidase family protein

transcription factor DIVARICATA-like

protein ENHANCED PSEUDOMONAS SUSCEPTIBILTY 1

probable 2-oxoglutarate-dependent dioxygenase At5g05600

GDSL esterase/lipase CPRD49-like isoform X1

vacuolar cation/proton exchanger 3

pyruvate decarboxylase 2

geraniol 8-hydroxylase-like

receptor-like protein 7

zinc finger protein ZPR1-like

receptor-like protein 7

KEH28951.1 laccase/diphenol oxidase family protein

transcription factor MYB1

probable glutathione S-transferase

probable glutathione S-transferase

serine/threonine-protein kinase BSK6

disease resistance protein TAO1 isoform X5

UPF0160 protein C27H6.8

zinc finger protein 10-like

hsp70 nucleotide exchange factor fes1-like isoform X2

uncharacterized protein At3g17950-like

peroxidase 15-like

O-fucosyltransferase 20-like

cytochrome P450 83B1-like

SPX domain-containing protein 3

---NA---

serine/threonine-protein kinase STY13-like

UDP-glucuronate 4-epimerase 3

hypothetical protein VIGAN_10080400

For Review Only

KEH29092.1 DUF1442 family protein
cyclin-U2-1-like
ruvB-like protein 1 isoform X2
B3 domain-containing transcription repressor VAL2 isoform X1
uncharacterized protein LOC101515306 isoform X1
---NA---
non-specific lipid-transfer protein 1-like
cationic peroxidase 1-like
DEAD-box ATP-dependent RNA helicase 52C-like isoform X2
peroxidase P7-like
transcription repressor OFP1
non-specific lipid-transfer protein precursor
cytochrome P450 72A15-like
UDP-glucose flavonoid 3-O-glucosyltransferase 7
proteinase inhibitor PSI-1.2
NAD(P)H-quinone oxidoreductase subunit T, chloroplastic
serine/threonine-protein kinase-like protein CCR4
---NA---
ethylene-responsive transcription factor ERF043-like
AES65997.1 polygalacturonase
putative glycerol-3-phosphate transporter 1
uncharacterized protein LOC105852351
protein HIGH ARSENIC CONTENT 1, mitochondrial
uncharacterized protein LOC101493605 isoform X3
ubiquitin-like modifier-activating enzyme atg7
50S ribosomal protein L1, chloroplastic
uncharacterized protein LOC101488893
RecName: Full=Protein PROPEP890; Short=GmPROPEP890; Contains: RecName: Full=Peptide GmPep890; Flags: Precursor
RALF-like protein
RALF-like protein
RALF protein
21 kDa protein-like
pectinesterase inhibitor 4-like
CASP-like protein 1C1
probable polyol transporter 4
uncharacterized GPI-anchored protein At4g28100
KEH16437.1 transmembrane protein, putative
calcium-binding protein KRP1-like
KEH29555.1 beta-amylase
RecName: Full=Beta-amylase; AltName: Full=1,4-alpha-D-glucan maltohydrolase
xyloglucan endotransglucosylase/hydrolase protein 9-like isoform X1
uncharacterized protein LOC101515637
MYB-like transcription factor ETC1
uncharacterized protein LOC101514172
dnaJ protein homolog
---NA---

For Review Only

transcription factor LHW-like
NDR1/HIN1-like protein 1
transcription repressor OFP4
late embryogenesis abundant protein
transcription factor TGA9 isoform X2
zinc finger protein WIP3
cytochrome P450 94C1
AES88439.2 transmembrane protein, putative
glucomannan 4-beta-mannosyltransferase 2
putative phospholipid:diacylglycerol acyltransferase 2
protein argonaute 5
E3 ubiquitin-protein ligase RZFP34 isoform X4
uncharacterized protein LOC101509644
probable xyloglucan endotransglucosylase/hydrolase protein 32
serine carboxypeptidase-like
serine carboxypeptidase-like
heavy metal-associated isoprenylated plant protein 3-like
alpha carbonic anhydrase 7-like
alpha carbonic anhydrase 7-like
E3 ubiquitin-protein ligase WAV3-like
KEH30024.1 enoyl-(acyl carrier) reductase
cysteine synthase-like
uncharacterized protein LOC101510502
probable glutathione S-transferase
fasciclin-like arabinogalactan protein 12
fasciclin-like arabinogalactan protein 12
fasciclin-like arabinogalactan protein 12
auxin response factor 4 isoform X2
mitogen-activated protein kinase
protein SODIUM POTASSIUM ROOT DEFECTIVE 2
dirigent protein 10-like
aquaporin TIP1-3-like
transcription factor MYB48-like
protein UPSTREAM OF FLC
osmotin-like protein
osmotin-like protein
CLAVATA3/ESR (CLE)-related protein 46
heat shock cognate 70 kDa protein 2
probable serine/threonine-protein kinase PBL10 isoform X1
scarecrow-like protein 34
ubiquitin recognition factor in ER-associated degradation protein 1-like
ripening-related hydrolase-like protein
---NA---
sterile nodes
---NA---
UDP-glucose iridoid glucosyltransferase-like

For Review Only

extensin-2-like
serine/threonine-protein kinase UCN
transcription factor bHLH122
non-cyanogenic beta-glucosidase-like
non-cyanogenic beta-glucosidase-like
probable E3 ubiquitin-protein ligase ARI7
calmodulin-like protein 3
B3 domain-containing protein Os11g0197600-like
B3 domain-containing transcription factor VRN1-like isoform X1
54s ribosomal protein mitochondrial-like
hypothetical protein MTR_4g068560
allene oxide synthase 3-like
cold-regulated 413 plasma membrane protein 1
KEH30373.1 DUF4228 domain protein
uncharacterized protein LOC101514616 isoform X1
organ-specific protein P4-like
uncharacterized LOC101495589 precursor
phenylalanine--tRNA ligase alpha subunit, cytoplasmic
isoflavone reductase homolog PCBER
---NA---
probable nucleolar protein 5-1
Squalene synthase
putative arginine decarboxylase
probable transcription factor PosF21
probable xyloglucan glycosyltransferase 5
---NA---
KEH28522.1 lipid-binding serum glycoprotein-like protein
auxin-induced protein 6B
beta-amyrin synthase
uncharacterized protein At5g08430-like isoform X2
uncharacterized protein LOC11411232
FACT complex subunit SPT16
AES70911.1 TPX2 (targeting protein for Xklp2) family protein
KEH34623.1 senescence-associated protein, putative
inositol 1,3,4-trisphosphate 5/6-kinase 4 isoform X1
telomere repeat-binding protein 4-like
aspartic proteinase PCS1
L-type lectin-domain containing receptor kinase S.4-like
ribulose bisphosphate carboxylase/oxygenase activase 2, chloroplastic
probable transcriptional regulator RABBIT EARS
ARF guanine-nucleotide exchange factor GNOM-like
AES71067.2 lipase
coronatine-insensitive protein 1
putative Hs1pro-1-like protein
NF-X1-type zinc finger protein NFXL1-like
phosphoethanolamine N-methyltransferase

For Review Only

E3 ubiquitin-protein ligase SIRP1-like isoform X1
hypothetical protein MtrunA17_Chr8g0391661
protein PLANT CADMIUM RESISTANCE 11-like
protein PLANT CADMIUM RESISTANCE 2-like
---NA---
uncharacterized CRM domain-containing protein At3g25440, chloroplastic
---NA---
transcription repressor OFP8 isoform X1
protein SYM1-like
KEH21341.1 transmembrane protein, putative
AET05187.1 nucleotide-diphospho-sugar transferase domain protein
probable pectinesterase 53
inositol oxygenase 2-like
protein NDL1 isoform X1
short-chain dehydrogenase TIC 32, chloroplastic
luminal-binding protein
luminal-binding protein
UDP-glucose 4-epimerase GEPI48
putative RING-H2 finger protein ATL21A
uncharacterized protein LOC101498634 isoform X1
KEH21203.1 plant/K16L22-9 protein
6-phosphogluconate dehydrogenase, decarboxylating 1, chloroplastic
KEH21157.1 late embryogenesis abundant protein
respiratory burst oxidase homolog protein A
probable WRKY transcription factor 29
heavy metal-associated isoprenylated plant protein 39-like
homeobox protein BEL1 homolog
probable aquaporin PIP1-2
UDP-N-acetylmuramoyl-L-alanyl-D-glutamate--2,6-diaminopimelate ligase MurE homolog, chloroplastic
probable phospholipid hydroperoxide glutathione peroxidase
KEH36410.1 core-2/I-branching enzyme
gamma carbonic anhydrase-like 2, mitochondrial
auxin-responsive protein SAUR32-like
bidirectional sugar transporter SWEET13-like
patellin-3-like protein
MACPF domain-containing protein At4g24290-like
diacylglycerol kinase 2
NAC domain-containing protein 2
probable methyltransferase PMT26
uncharacterized protein LOC11442614 isoform X1
AET04733.1 cysteine desulfurylase
protein trichome berefringence-like 7
nodulation-signaling pathway 2 protein-like
WAT1-related protein At5g64700
probable ATP synthase 24 kDa subunit, mitochondrial
stress enhanced protein 2, chloroplastic

For Review Only

cytochrome P450 CYP736A12-like
L-ascorbate oxidase
inositol-3-phosphate synthase-like
acyl-coenzyme A oxidase 2, peroxisomal
KEH16213.1 DUF4057 family protein
annexin D2
zeatin O-glucosyltransferase
haloacid dehalogenase-like hydrolase domain-containing protein At4g39970
serine/threonine-protein kinase SAPK2
LMBR1 domain-containing protein 2 homolog A-like isoform X1
putative clathrin assembly protein At4g40080
KEH20773.1 lysine ketoglutarate reductase trans-splicing protein
glycosyl hydrolase 5 family protein-like
major pollen allergen Ole e 10-like isoform X1
beta-keto reductase
uncharacterized protein LOC101509749
11S globulin seed storage protein 2-like
transmembrane protein, putative
kxDL motif-containing protein 1
putative aldo/keto reductase, NADP-dependent oxidoreductase domain-containing protein
protein NRT1/ PTR FAMILY 4.5-like isoform X2
probable LRR receptor-like serine/threonine-protein kinase RFK1 isoform X3
XP_004504264.1 nodulin-26-like
AAL32128.1 multifunctional aquaporin
heat stress transcription factor A-4a
glutathione transferase GST 23
probable glutathione S-transferase
hypothetical protein MTR_8g087320
AET04135.1 transcription regulators protein
RHN42646.1 putative actinidain
transcription factor HRS1-like isoform X1
CBL-interacting serine/threonine-protein kinase 12
uncharacterized protein LOC101499907
heavy metal-associated isoprenylated plant protein 28-like
thioredoxin-like fold domain-containing protein MRL7, chloroplastic
probable polyamine transporter At3g13620
chromatin-remodeling complex subunit ies6
chalcone synthase
probable magnesium transporter NIPA1 isoform X2
DNA ligase 1
serine hydroxymethyltransferase 4-like
protein RALF-like 32
probable acyl-activating enzyme 16, chloroplastic isoform X1
receptor-like kinase TMK4
xyloglucan endotransglucosylase/hydrolase 2
probable amidase At4g34880 isoform X2

For Review Only

Stress-associated endoplasmic reticulum protein 2
F-box protein At1g67340-like
cytochrome P450 78A5-like
---NA---
mannosyl-oligosaccharide glucosidase GCS1-like isoform X4
putative ABC transporter C family member 15
---NA---
flocculation protein FLO11 isoform X1
uncharacterized protein LOC101501830
protein TIFY 5A-like
putative Acid phosphatase
receptor-like protein kinase FERONIA
uncharacterized protein LOC101492109
serine/threonine-protein kinase BRI1-like 2
protein XRI1
Protein NRT1/ PTR FAMILY 8.3
remorin 4.1
isoflavone 2'-hydroxylase
polygalacturonase At1g48100-like
eukaryotic peptide chain release factor subunit 1-3
KEH31377.1 thiamine monophosphate synthase
uncharacterized protein LOC101495357
uncharacterized protein LOC105852274
chlorophyll a-b binding protein
WRKY transcription factor 71
berberine bridge enzyme-like 21
polygalacturonase inhibitor 2-like
protein LIGHT-DEPENDENT SHORT HYPOCOTYLS 4-like
uncharacterized protein LOC101506443
vacuolar iron transporter-like protein
beta-xylosidase/alpha-L-arabinofuranosidase 2
chaperone protein dnaJ 20, chloroplastic-like
---NA---
tobamovirus multiplication protein 1-like
uncharacterized protein LOC101505572 isoform X1
18.2 kDa class I heat shock protein-like
glutamyl-tRNA reductase 2, chloroplastic-like isoform X1
receptor-like serine/threonine-protein kinase SD1-8 isoform X2
external alternative NAD(P)H-ubiquinone oxidoreductase B2, mitochondrial
F-box protein At1g61340
calmodulin-binding protein 60 D-like
sugar carrier protein C-like isoform X1
EPIDERMAL PATTERNING FACTOR-like protein 4
AES90378.1 transmembrane protein, putative
dof zinc finger protein DOF1.4-like
Splicing factor 3B subunit 2

For Review Only

cytochrome P450 71A3-like isoform X2
stem-specific protein TSJT1-like isoform X1
heat stress transcription factor B-2a
GTP-binding protein At2g22870
18.5 kDa class I heat shock protein-like
18.5 kDa class I heat shock protein-like
cytochrome P450 CYP82D47
triose phosphate/phosphate translocator, chloroplastic
isoflavone 4'-O-methyltransferase
isoflavone synthase precursor
histone H3-like centromeric protein CSE4
transcription factor bHLH106-like
(+)-neomenthol dehydrogenase isoform X1
probable 2-oxoglutarate-dependent dioxygenase At5g05600
AES90217.1 calmodulin-binding protein
ethylene-responsive transcription factor ERF109-like
ferredoxin--nitrite reductase, chloroplastic
LRR receptor-like serine/threonine-protein kinase FLS2
AES90113.2 Serine/Threonine kinase, plant-type protein, putative
homeobox-leucine zipper protein HOX3
probable sulfate transporter 3.3
---NA---
probable inorganic phosphate transporter 1-9
glucan endo-1,3-beta-glucosidase isoform X1
LOB domain-containing protein 4
cyclic dof factor 1
G-type lectin S-receptor-like serine/threonine-protein kinase At4g27290 isoform X1
AES89818.1 DUF1685 family protein
serine/threonine-protein kinase EDR1 isoform X2
F-box protein MAX2
peroxidase 17-like
protein LIGHT-DEPENDENT SHORT HYPOCOTYLS 10
BnaCnng12640D
probable O-methyltransferase 3
dnaJ homolog subfamily B member 13-like
switch 2 isoform X1
AES89662.1 DUF506 family protein
probable serine/threonine-protein kinase At1g54610 isoform X1
geraniol 8-hydroxylase-like
---NA---
isoflavone 2'-hydroxylase-like
isoflavone 2'-hydroxylase-like
cinnamoyl-CoA reductase 1
glucan endo-1,3-beta-glucosidase
scarecrow-like protein 3
NAC domain-containing protein

For Review Only

expansin-like A2
KEH30613.1 late embryogenesis abundant protein
serine carboxypeptidase-like 34
putative C2 domain-containing protein
gibberellin 2-beta-dioxygenase 6-like
protein NUCLEAR FUSION DEFECTIVE 4-like
calcium-transporting ATPase 9, plasma membrane-type
KH domain-containing protein HEN4 isoform X1
---NA---
uncharacterized protein LOC101497985
aspartyl protease family protein At5g10770
shikimate O-hydroxycinnamoyltransferase-like
probable WRKY transcription factor 40
cinnamoyl-CoA reductase 2
transcription factor bHLH18
transcription factor bHLH25-like isoform X1
putative UPF0481 protein At3g02645
---NA---
putative sterile alpha motif/pointed domain-containing protein
F-box/LRR-repeat protein 14-like isoform X3
AES86570.1 post-illumination chlorophyll fluorescence increase
methyl-CpG-binding domain-containing protein 11 isoform X1
AES86581.1 TPR repeat protein
DUF4050 family protein
putative protein TPRXL
Retrovirus-related Pol polyprotein from transposon TNT 1-94
MAPK kinase substrate protein At1g80180-like
uncharacterized RNA-binding protein C17H9.04c
auxin-induced protein IAA6 isoform X1
uncharacterized protein LOC101513959
uncharacterized protein LOC101513630
KEH28820.1 SEC14 cytosolic factor family protein
dirigent protein 19-like
dirigent protein 22-like
dirigent protein 22-like
sulfoquinovosyl transferase SQD2
uncharacterized protein LOC105852408
---NA---
chlorophyll a-b binding protein CP29.1, chloroplastic
E3 ubiquitin-protein ligase AIRP2-like isoform X1
trihelix transcription factor GT-3b
uncharacterized protein LOC101492243
heme-binding-like protein At3g10130, chloroplastic
O-fucosyltransferase 19
protein NRT1/ PTR FAMILY 5.6-like
serine/threonine-protein kinase BSK6

For Review Only

NADPH-dependent aldo-keto reductase, chloroplastic-like
Avr9/Cf-9 rapidly elicited protein
---NA---
uncharacterized protein At2g37660, chloroplastic-like
putative E3 ubiquitin-protein ligase XBAT31
---NA---
BTB/POZ domain-containing protein At5g48800
3-ketoacyl-CoA thiolase 2, peroxisomal
myeloid leukemia factor 1 isoform X1
probable protein S-acyltransferase 14 isoform X2
protein transport Sec1a-like
SPX domain-containing protein 3
---NA---
pectinesterase 2
---NA---
pectinesterase inhibitor 9-like
AES87301.1 DUF1442 family protein
ribonuclease H
protein LURP-one-related 15-like isoform X1
transcription repressor OFP1
KEH29320.1 patellin-like protein
GDSL esterase/lipase At1g71250
probable inactive purple acid phosphatase 27
heat shock 22 kDa protein, mitochondrial isoform X2
uncharacterized protein LOC105852351
acyl-CoA--sterol O-acyltransferase 1
uncharacterized protein LOC101488216
protein EXORDIUM-like 3
KEH16659.1 expansin-like protein B1
uncharacterized protein LOC101514389
uncharacterized protein LOC101508901
transcription factor MYB13-like
transcription factor WER-like
transcription factor MYB30-like
---NA---
CAP2 protein
uncharacterized protein LOC101509648 isoform X1
peroxidase 3-like
ribonuclease H
vascular-related unknown protein 1-like
AES88116.1 DUF4228 domain protein
probable WRKY transcription factor 31
protein STRICTOSIDINE SYNTHASE-LIKE 3-like
uncharacterized protein LOC101502581
secoisolariciresinol dehydrogenase-like
photosystem I subunit O

For Review Only

F-box protein At2g27310-like
RING-H2 finger protein ATL57
zinc finger protein ZAT5
probable xyloglucan endotransglucosylase/hydrolase protein 32
high-affinity nitrate transporter 2.1-like
F-box/kelch-repeat protein At3g06240-like
yrdC domain-containing protein, mitochondrial isoform X1
fasciclin-like arabinogalactan protein 12
fasciclin-like arabinogalactan protein 12
18.5 kDa class I heat shock protein-like
LOB domain-containing protein 1-like
AES88695.1 tetraspanin family protein
dof zinc finger protein DOF2.1
mitogen-activated protein kinase
L-ascorbate peroxidase, cytosolic
ethylene-responsive transcription factor RAP2-7
phototropin-1-like isoform X2
receptor-like protein kinase HERK 1 isoform X2
dirigent protein 10-like
glutelin type-D 1-like
very-long-chain (3R)-3-hydroxyacyl-CoA dehydratase 2
transcription factor MYB48-like
histone H2B.11-like
KEH30169.1 expansin A10
scarecrow-like protein 33
scarecrow-like protein 30
ATP-dependent zinc metalloprotease FTSH 10, mitochondrial-like
Ras-related protein Rab11C
UDP-glucose iridoid glucosyltransferase-like
extensin-2-like
extensin-2-like
extensin-2-like
DNA damage-repair/toleration protein DRT100-like
lysine-specific demethylase JMJ30
cyanogenic beta-glucosidase
cyanogenic beta-glucosidase-like
non-cyanogenic beta-glucosidase-like
non-cyanogenic beta-glucosidase-like
non-cyanogenic beta-glucosidase-like
non-cyanogenic beta-glucosidase-like
non-cyanogenic beta-glucosidase-like
non-cyanogenic beta-glucosidase-like
protein FAR1-related sequence 6-like
AES89016.1 calreticulin
uncharacterized protein LOC101502998
transmembrane protein 45A

For Review Only

protein JINGUBANG isoform X2
uncharacterized protein LOC101500782
plastid division protein PDV2
uncharacterized protein LOC101514616 isoform X1
uncharacterized protein LOC101490202
UPF0496 protein
dolichyl-diphosphooligosaccharide--protein glycosyltransferase 48 kDa subunit
BURP domain protein USPL1-like
uncharacterized LOC101495589 precursor
glucan endo-1,3-beta-glucosidase 7
fructose-bisphosphate aldolase 1, chloroplastic
auxin-responsive protein SAUR50
4-coumarate--CoA ligase 1-like
---NA---
hypothetical protein MANES_06G077400, partial
hypothetical protein MANES_06G077400, partial
---NA---
---NA---
uncharacterized protein LOC11437166
---NA---
---NA---
uncharacterized protein LOC101504831
---NA---
---NA---
---NA---
---NA---
---NA---
hypothetical protein TSUD_321200
auxin-induced protein 10A5-like
---NA---
uncharacterized protein LOC101513630
hypothetical protein MTR_4g014400
uncharacterized protein LOC101494040
legume-specific protein
putative NADH:ubiquinone reductase (H(+)-translocating) (mitochondrion)
ATP synthase subunit 9 (mitochondrion)
---NA---
PAX-interacting protein 1
---NA---
---NA---
---NA---
---NA---
---NA---
---NA---
---NA---
transmembrane protein, putative

For Review Only

hypothetical protein KK1_012296
non-lysosomal glucosylceramidase isoform X1
uncharacterized protein LOC101496765
protein root UVB sensitive 6
START domain-containing protein 10 isoform X1
phosphatidylinositol/phosphatidylcholine transfer protein SFH2-like
nudix hydrolase 2-like isoform X1
KEH32686.1 pollen Ole e I family allergen
scarecrow-like protein 13
cytochrome b561 and DOMON domain-containing protein At4g17280-like isoform X1
histone H2B type 1-A-like
calcium permeable stress-gated cation channel 1-like
probable WRKY transcription factor 31
S-type anion channel SLAH1-like
probable F-box protein At4g22030
arogenate dehydratase/prephenate dehydratase 1, chloroplastic
cationic peroxidase precursor
uncharacterized protein LOC101501534
polyadenylate-binding protein RBP45 isoform X1
NADP-dependent glyceraldehyde-3-phosphate dehydrogenase
omega-6 fatty acid desaturase, chloroplastic isoform X1
heat shock 70 kDa protein
cellulose synthase A catalytic subunit 7 [UDP-forming]
---NA---
---NA---
AFG1-like ATPase isoform X2
tetraspanin-8-like
protein ECERIFERUM 3
transcription factor IBH1-like 1
VAN3-binding protein
uncharacterized protein LOC101506460
probable alpha,alpha-trehalose-phosphate synthase [UDP-forming] 11
---NA---
pathogenesis-related protein PR-1
pathogenesis-related protein PR-1
calmodulin-binding protein 60 B isoform X1
probable xyloglucan endotransglucosylase/hydrolase protein 23 isoform X2
AES92343.1 DUF761 domain protein
replication protein A 32 kDa subunit B isoform X2
VIN3-like protein 2 isoform X2
LOW QUALITY PROTEIN: uncharacterized protein LOC101502059
putative inactive cadmium/zinc-transporting ATPase HMA3
probable WRKY transcription factor 11
homeobox-leucine zipper protein HAT22-like
probable xyloglucan endotransglucosylase/hydrolase protein 23
pathogen-associated molecular patterns-induced protein A70

For Review Only

IQ domain-containing protein IQM1
IQ domain-containing protein IQM1
U-box domain-containing protein 34
protein ALP1-like
uncharacterized protein LOC101502176
ACO24551.1 chloroplast photosystem II subunit X
12-oxophytodienoate reductase 3
serine/threonine-protein kinase RIPK-like
subtilisin-like protease SBT1.8
inter alpha-trypsin inhibitor, heavy chain 4-like isoform X1
protein TIFY 10A-like
3-oxoacyl-[acyl-carrier-protein] synthase II, chloroplastic-like
RZB55236.1 Indole-3-acetic acid-induced protein ARG7 isoform A
sucrose synthase
putative pectinesterase/pectinesterase inhibitor 45
alpha-aminoadipic semialdehyde synthase isoform X1
guanosine deaminase
CBL-interacting serine/threonine-protein kinase 1
probable protein phosphatase 2C 13
transcription factor MYB108
alkaline/neutral invertase A, mitochondrial isoform X1
serine carboxypeptidase-like 26
hypothetical protein (mitochondrion)
cell wall / vacuolar inhibitor of fructosidase 1
cell wall / vacuolar inhibitor of fructosidase 1-like precursor
cell wall / vacuolar inhibitor of fructosidase 1-like
hypothetical protein GLYMA_07G240800
acetate/butyrate--CoA ligase AAE7, peroxisomal isoform X2
COBRA-like protein 7
ABA-responsive protein ABR18-like
uncharacterized protein LOC101512882
probable protein phosphatase 2C 40
CASP-like protein 1D2
uncharacterized protein At4g15545-like, partial
protein DMP2-like
protein DMP2-like
UDP-glucose flavonoid 3-O-glucosyltransferase 7
serine/threonine-protein kinase STN8, chloroplastic-like
---NA---
copper amine oxidase precursor
---NA---
heavy metal-associated isoprenylated plant protein 32
basic 7S globulin-like
stem-specific protein TSJT1
transmembrane 9 superfamily member 11
uncharacterized protein LOC101506464

For Review Only

hypothetical protein E2542_SST19178
monogalactosyldiacylglycerol synthase 2, chloroplastic-like
putative chloride channel-like protein CLC-g
probable glycosyltransferase At3g42180 isoform X1
probable glycosyltransferase At5g20260 isoform X1
36.4 kDa proline-rich protein
unknown
calmodulin-like protein 7
blue copper protein-like
SPX domain-containing protein 1
peroxidase 25
AES91487.1 lactoylglutathione lyase/glyoxalase I family protein
transcriptional corepressor LEUNIG-like
protein PGR
uncharacterized protein LOC101507638
putative phospholipid-transporting ATPase 9
protein CHROMATIN REMODELING 24
SNAP25 homologous protein SNAP33
uncharacterized protein At5g01610-like
uncharacterized protein LOC101503663
transcription factor TCP14
transcription factor bHLH47
zinc finger protein BRUTUS-like At1g18910
uncharacterized protein LOC25493783
transcription factor MYB1R1
AES91124.1 UVI1, putative
metal tolerance protein 4-like
---NA---
snakin-2-like precursor
calmodulin-binding protein 60 A isoform X1
mannosyltransferase family protein
protease Do-like 9
ubiquitin-conjugating enzyme E2-23 kDa-like isoform X1
aldehyde dehydrogenase family 2 member B7, mitochondrial isoform X1
U-box domain-containing protein 19-like
RHN63486.1 putative neprosin
tryptophan aminotransferase-related protein 2-like
L-type lectin-domain containing receptor kinase S.1-like
PB1 domain protein
hypothetical protein MtrunA17_Chr4g0058531
KEH31766.1 protein phosphatase 2c, putative
cytochrome P450 71A26-like
hypothetical protein GLYMA_06G171100
probable inactive purple acid phosphatase 27
molybdenum cofactor sulfurase
ethylene-responsive transcription factor ERF026-like

For Review Only

dehydration-responsive element-binding protein 1E-like
geraniol 8-hydroxylase-like
DUF1442 family protein
AES90982.2 acytochrome-C oxidase/electron carrier protein
protein YLS3-like
14 kDa proline-rich protein DC2.15-like
lipid transfer protein EARLI 1-like
14 kDa proline-rich protein DC2.15
KEH31647.1 transmembrane protein, putative
ethylene-responsive transcription factor ERF039
deSI-like protein At4g17486
ethylene-responsive transcription factor 1A
phospholipase A1-Ibeta2, chloroplastic
bax inhibitor 1-like
potassium transporter 5
probable serine/threonine-protein kinase PBL19
FCS-Like Zinc finger 2-like
expansin-like B1
two-component response regulator-APRR2-like protein
type I inositol polyphosphate 5-phosphatase 2-like
plant intracellular Ras-group-related LRR protein 5-like isoform X1
transcription factor NAI1-like
transcription factor bHLH19
---NA---
LOB domain-containing protein 38
scarecrow-like protein 32
uncharacterized protein LOC101510644
lysine-rich arabinogalactan protein 18-like
cytochrome P450 81E8-like
PAMP-induced secreted peptide 2
floral homeotic protein APETALA 2
protein ENHANCED PSEUDOMONAS SUSCEPTIBILTY 1
precursor of CEP9-like
putative encoded peptide
L-ascorbate oxidase homolog
L-ascorbate oxidase homolog
methylesterase 1-like
GDSL esterase/lipase At2g23540 isoform X2
wall-associated receptor kinase-like 14
oxygen-evolving enhancer protein 1, chloroplastic
uncharacterized protein LOC101502801
STS14 protein-like
elongation of fatty acids protein 3-like
phosphatidylinositol transfer protein 3-like
protein FAR1-RELATED SEQUENCE 5-like
KEH20419.1 plant/F18B3-190 protein, putative

For Review Only

KEH20411.1 transmembrane protein, putative
peroxidase 72-like
AET03861.1 50S ribosomal protein L7/L12
cytochrome P450 84A1-like
uncharacterized protein LOC101515606
DNA-(apurinic or apyrimidinic site) lyase 2 isoform X1
thaumatin-like protein 1
thiosulfate sulfurtransferase 18
peroxidase 64
probable serine/threonine-protein kinase PIX13 isoform X2
MLO-like protein 10
ruBisCO-associated protein-like
3-hydroxy-3-methylglutaryl-coenzyme A reductase 3-like
---NA---
glycine-rich protein A3-like
abscisic acid 8'-hydroxylase CYP707A2-like
---NA---
mechanosensitive ion channel protein 10
isoflavone reductase homolog
---NA---
LOW QUALITY PROTEIN: uncharacterized protein LOC101496266
cysteine-rich knottin fold-containing protein precursor
NADPH-dependent aldo-keto reductase, chloroplastic-like
phosphate transporter PHO1 homolog 1 isoform X2
putative phospholipid-transporting ATPase 9
alpha-glucan water dikinase, chloroplastic isoform X2
cyclin-dependent protein kinase inhibitor SMR6-like
---NA---
uncharacterized protein LOC101497902
E3 ubiquitin-protein ligase WAVH1-like
probable ADP-ribosylation factor GTPase-activating protein AGD8 isoform X1
AET03272.1 PLATZ transcription factor family protein
putative DNA-3-methyladenine glycosylase I
hypothetical protein MTR_8g066155
probable xyloglucan endotransglucosylase/hydrolase protein 33
---NA---
---NA---
cellulose synthase A catalytic subunit 7 [UDP-forming]-like isoform X2
nucleobase-ascorbate transporter 6
60S ribosomal protein L27a-2-like
---NA---
---NA---
F-box/kelch-repeat protein At1g26930-like
PH, RCC1 and FYVE domains-containing protein 1-like isoform X1
delta(8)-fatty-acid desaturase 1-like
---NA---

For Review Only

KEH19737.1 CAAX amino terminal protease family protein
amino acid transporter AVT1I
diacylglycerol kinase 5-like
indole-3-acetic acid-amido synthetase GH3.6
dynein light chain 2, cytoplasmic
myosin-2 isoform X2
cyclic nucleotide-gated ion channel 4-like isoform X1
uncharacterized protein LOC105852351
metallothionein-like protein type 3
NADH-ubiquinone oxidoreductase chain 5
probable protein phosphatase 2C 39
uncharacterized protein LOC101494249
protein transport protein Sec61 subunit alpha-like isoform X1
cold-responsive protein kinase 1-like
phosphoribulokinase/uridine kinase-like protein
acid phosphatase 1-like
RHN40162.1 putative isopenicillin N synthase
auxin response factor 2 isoform X2
---NA---
uncharacterized protein LOC113787691
ALA-interacting subunit 3-like
putative cyclin-B3-1 isoform X2
protein MAIN-LIKE 1-like
MLP-like protein 28
embryonic abundant protein VF30.1-like
MLP-like protein 34
uncharacterized protein LOC101499126
protein ZINC INDUCED FACILITATOR-LIKE 1-like isoform X1
subtilisin-like serine protease
---NA---
protein ENHANCED PSEUDOMONAS SUSCEPTIBILTY 1-like
---NA---
two-component response regulator ORR9-like
annexin-like protein RJ4
flavin-containing monooxygenase FMO GS-OX5-like
protein CASPARIAN STRIP INTEGRITY FACTOR 1
---NA---
uncharacterized protein LOC101513856
transcription factor bHLH130-like
LOB domain-containing protein 30-like
---NA---
4-hydroxy-tetrahydrodipicolinate synthase, chloroplastic-like
KEH18942.1 BON1-associated-like protein
gibberellin 20 oxidase 2-like isoform X1
MADS-box protein SOC1
transcription factor TCP9

For Review Only

ethylene-responsive transcription factor ERF012-like
shaggy-related protein kinase theta-like
inositol polyphosphate multikinase beta
remorin-like
FT-interacting protein 7-like
gibberellin 20-oxidase-like protein
TMV resistance protein N
---NA---
heavy metal-associated isoprenylated plant protein 39-like isoform X1
WRKY transcription factor 22-like
RING-H2 finger protein ATL67 isoform X1
F-box/kelch-repeat protein At3g61590-like
BTB/POZ domain-containing protein POB1-like
uncharacterized protein LOC101509356
ribonuclease 2
cytochrome b561 and DOMON domain-containing protein At3g07570
mediator of RNA polymerase II transcription subunit 19a-like
jasmonic acid-amido synthetase JAR1-like
glycosyltransferase family 28 protein
cyclic nucleotide-gated ion channel 1
protein yippee-like At4g27745
ras-related protein RABA3-like isoform X1
transcription factor bHLH14
---NA---
CBL-interacting serine/threonine-protein kinase 9
cyclic nucleotide-gated ion channel 1-like
chlorophyll a-b binding protein 7, chloroplastic
tyrosine-sulfated glycopeptide receptor 1
protein STRUBBELIG-RECEPTOR FAMILY 6
RING-H2 finger protein ATL60
pectinesterase 3-like isoform X1
pectinesterase 3
uncharacterized protein LOC101515722 isoform X1
protein LURP-one-related 11-like
FCS-Like Zinc finger 17-like
GDSL esterase/lipase 5-like
auxin-responsive protein SAUR50
chaperone DnaJ domain protein
dormancy-associated protein homolog 4 isoform X1
trihelix transcription factor ASIL2
uncharacterized protein LOC101512363
linoleate 13S-lipoxygenase 3-1, chloroplastic
flavonoid 3'-monooxygenase-like
E3 ubiquitin-protein ligase SPL2
disease resistance protein RML1A
calumenin-B-like isoform X1

For Review Only

disease resistance protein LAZ5
protein BIG GRAIN 1-like B
formin-like protein 18
disease resistance-like protein DSC1
protein indeterminate-domain 11 isoform X1
inorganic pyrophosphatase 2-like
dof zinc finger protein DOF1.7
60S ribosomal protein L28-2 isoform B
transcription factor bHLH87-like isoform X1
stem 28 kDa glycoprotein-like
auxin-responsive protein IAA26-like
GATA transcription factor 9-like
CASP-like protein 3A1
aquaporin TIP2-1
LON peptidase N-terminal domain and RING finger protein 2-like
acid phosphatase 1-like
beta-amyrin 11-oxidase-like
membrane protein of ER body-like protein
defensin-like protein 19
hydroquinone glucosyltransferase-like
mediator-associated protein 2-like
---NA---
peptidyl-prolyl cis-trans isomerase FKBP62-like
40S ribosomal protein S8
uncharacterized protein LOC101513106
serine/threonine-protein kinase OXI1-like
MLO-like protein 1
RALF-like protein
---NA---
---NA---
---NA---
putative ABC transporter C family member 15 isoform X1
UMP-CMP kinase 3-like
uncharacterized protein LOC101512034
AET01279.1 ferredoxin-like protein
FCS-Like Zinc finger 3
methylesterase 3-like isoform X1
ethylene-responsive transcription factor 13-like
ranBP2-type zinc finger protein At1g67325
KEH17830.1 calcium ion-binding protein, putative
hydroquinone glucosyltransferase
oxygen-evolving enhancer protein 3, chloroplastic-like
oxygen-evolving enhancer protein 3, chloroplastic-like
beta-centractin-like
UPF0481 protein At3g47200-like
uncharacterized protein LOC101502605 isoform X2

For Review Only

acid phosphatase 1-like isoform X1
NADPH-dependent pterin aldehyde reductase
NADH dehydrogenase [ubiquinone] 1 beta subcomplex subunit 3-B
KEH32730.1 YacP-like NYN domain protein
KEH32700.1 universal stress family protein
U-box domain-containing protein 35-like
nuclear transcription factor Y subunit B-3-like protein
oligopeptide transporter 3
E3 ubiquitin-protein ligase UPL5-like
E3 ubiquitin-protein ligase COP1
probable transcription repressor OFP9
---NA---
L-ascorbate oxidase homolog
beta-glucosidase 46-like
BTB/POZ domain-containing protein At2g24240
translation initiation factor IF3-4, chloroplastic-like
Transcription regulatory protein SNF5
WAT1-related protein At4g30420-like
zinc finger protein CONSTANS-LIKE 5 isoform X1
pathogenesis-related protein PR-1
xyloglucan endotransglucosylase/hydrolase protein 22-like
probable xyloglucan endotransglucosylase/hydrolase protein 23 isoform X2
AES92334.2 galactosyltransferase family protein
AAA-ATPase At5g57480
uncharacterized protein LOC101495164
NADPH--cytochrome P450 reductase
peroxidase 16-like
protein CHLOROPLAST IMPORT APPARATUS 2-like
protein SMAX1-LIKE 4
caffeoylshikimate esterase
auxin efflux carrier component 2
probable serine/threonine-protein kinase PIX13 isoform X2
lysine-specific demethylase NO66
receptor-like serine/threonine-protein kinase ALE2 isoform X2
DNA repair protein recA homolog 3, mitochondrial-like isoform X1
KEH32499.1 DUF3511 domain protein
peroxidase N
uncharacterized protein LOC101500266
uncharacterized membrane protein C776.05-like isoform X2
thiol protease aleurain-like
FCS-Like Zinc finger 13
Stress-response A/B barrel domain-containing protein HS1
pectinesterase inhibitor 3-like
auxin-responsive protein SAUR78-like
probable pectinesterase/pectinesterase inhibitor 12 isoform X1
pectinesterase/pectinesterase inhibitor PPE8B

For Review Only

protein FATTY ACID EXPORT 2, chloroplastic
uncharacterized protein LOC101505378
probable GTP diphosphokinase CRSH, chloroplastic
probable sugar phosphate/phosphate translocator At3g17430 isoform X1
pto-interacting protein 1
S-adenosylmethionine synthase
gibberellin 2-beta-dioxygenase 2
---NA---
AES92006.2 transmembrane protein, putative
pentatricopeptide repeat-containing protein At1g59720, chloroplastic/mitochondrial-like
calcium-binding protein CP1
dirigent protein 11-like
ABA-responsive protein ABR18-like
ABA-responsive protein ABR18-like
J protein JJJ2
TPR repeat protein
uncharacterized protein LOC101489667
RING-H2 finger protein ATL2-like
late embryogenesis abundant protein Lea5
flowering-promoting factor 1-like
protein SRC2-like
acyl-CoA-binding domain-containing protein 6-like isoform X1
AES91778.1 F-box-like protein
probable 1-deoxy-D-xylulose-5-phosphate synthase, chloroplastic
sugar transporter ERD6-like 16
isoflavonoid glucosyltransferase
UDP-glucose flavonoid 3-O-glucosyltransferase 7
copper amine oxidase precursor
dicer-like protein 4 isoform X5
AES91712.1 stress induced protein
uncharacterized protein LOC105852471
F-box/LRR-repeat protein 3 isoform X2
putative clathrin assembly protein At5g35200
zinc finger BED domain-containing protein DAYSLEEPER isoform X1
hypothetical protein PHAVU_003G1683001g
hypothetical protein VIGAN_01430300
blue copper protein-like
cucumber peeling cupredoxin-like protein
transmembrane protein, putative
protein NRT1/ PTR FAMILY 6.2
MAPK kinase substrate protein At1g80180
early nodulin-93-like
probable receptor-like protein kinase At5g20050
cytochrome P450 734A1
allene oxide cyclase, chloroplastic-like
protein MIZU-KUSSEI 1

For Review Only

protein Dr1 homolog isoform X1
probable calcium-binding protein CML15
ethylene-responsive transcription factor RAP2-9-like
chaperone protein ClpB1
probable LRR receptor-like serine/threonine-protein kinase At1g74360
probable xyloglucan 6-xylosyltransferase 5
two-component response regulator-like APRR1 isoform X1
cyclic dof factor 3
probable WRKY transcription factor 72 isoform X2
protein QUIRKY isoform X1
zinc finger protein BRUTUS-like At1g18910
---NA---
two-component response regulator ARR5-like
AES91124.1 UVI1, putative
---NA---
uncharacterized protein LOC101514073
uncharacterized protein LOC101489558
probable inactive poly [ADP-ribose] polymerase SRO5 isoform X1
LRR receptor-like serine/threonine-protein kinase GSO1
VAMP-like protein YKT61
short-chain dehydrogenase TIC 32, chloroplastic-like isoform X1
armadillo repeat only 1 protein
pentatricopeptide repeat-containing protein At3g48250, chloroplastic
cytochrome P450 71A1-like
---NA---
proline transporter 2-like
BTB/POZ and TAZ domain-containing protein 2-like
probable pectate lyase 18
nucleoside diphosphate kinase 2
adenylate kinase 4
ribonuclease J
subtilisin-like protease SBT1.2
probable sugar phosphate/phosphate translocator At1g12500
copper chaperone for superoxide dismutase, chloroplastic/cytosolic
protein YLS3-like
NAC domain-containing protein 7-like
RGS1-HXK1-interacting protein 1
monocopper oxidase-like protein SKU5
probable trehalose-phosphate phosphatase F
protein NRT1/ PTR FAMILY 6.3
14 kDa proline-rich protein DC2.15-like
14 kDa proline-rich protein DC2.15
14 kDa proline-rich protein DC2.15-like
protein transport protein SEC16B homolog
transcription factor MYB114-like
AES90886.1 transmembrane protein, putative

For Review Only

homeobox-leucine zipper protein HAT4-like
glutamic acid-rich protein
---NA---
pentatricopeptide repeat-containing protein At4g17616
beta-amylase 3, chloroplastic-like
expansin-like B1
probable purine permease 11 isoform X1
protein NRT1/ PTR FAMILY 7.3-like
AT-hook motif nuclear-localized protein 23
leucine-rich repeat receptor-like serine/threonine-protein kinase BAM1
AP2-like ethylene-responsive transcription factor ANT
short-chain dehydrogenase cctT-like
basic leucine zipper 4-like
VQ motif-containing protein 29
histone H3.3
probable N-acetyltransferase HLS1
subtilisin-like protease SBT1.7
metal transporter Nramp3-like
zinc finger protein 7-like
protein IRX15-LIKE
kinesin-like protein KIN-4A isoform X2
putative mitogen-activated protein kinase kinase kinase STE-STE11 family
uncharacterized protein LOC101506676 isoform X1
uncharacterized protein LOC101508500 isoform X1
phosphoglycerate mutase-like protein 4
probable disease resistance protein At5g66900
dof zinc finger protein DOF5.3-like
GDSL esterase/lipase At2g23540 isoform X1
wall-associated receptor kinase-like 14
wall-associated receptor kinase-like 14
probable galacturonosyltransferase-like 1
calcium uniporter protein 2, mitochondrial
protein N-lysine methyltransferase METTL21A
abscisic acid 8'-hydroxylase 3-like
KEH20402.1 epoxide hydrolase
beta-galactosidase 3-like
cytochrome P450 84A1-like
Heterogeneous nuclear ribonucleoprotein Q
mediator of RNA polymerase II transcription subunit 4-like
chaperone protein dnaJ 11, chloroplastic-like
probable serine/threonine-protein kinase PBL11
IST1-like protein
AET03679.1 DUF241 domain protein
AET03677.1 DUF241 domain protein
AET03677.1 DUF241 domain protein
SNQ45215.1 Pathogenesis related protein

For Review Only

probable acyl-activating enzyme 2 isoform X1
dirigent protein 19-like
dehydrodolichyl diphosphate synthase 6 isoform X1
plant intracellular Ras-group-related LRR protein 5
diacylglycerol O-acyltransferase 2D-like isoform X2
pectin acetylesterase 8-like
ubiquitin-conjugating enzyme E2 28-like
plant/MCA23-20 protein
putative bifunctional inhibitor/plant lipid transfer protein/seed storage helical
high affinity nitrate transporter 2.5
heme-binding protein 2-like
pentatricopeptide repeat-containing protein At1g60770-like
---NA---
Mitochondrial uncoupling protein 5
transcription factor MYC2-like
glycine-rich cell wall structural protein 1.0-like
pantothenate kinase 1
gibberellin-regulated protein 3
phospholipid-transporting ATPase 3-like
---NA---
actin cross-linking protein
receptor kinase-like protein Xa21
putative TIR domain-containing protein
DNA-binding protein HEXBP isoform X2
AET02842.2 transmembrane protein
RING/FYVE/PHD zinc finger protein
diacylglycerol kinase 5-like
phytohormone-binding protein-like
NAC domain-containing protein 72
metallothionein-like protein type 3
probable aminotransferase TAT2
NADH dehydrogenase subunit 5 (mitochondrion)
early nodulin-like protein 2
---NA---
transcription factor TCP4-like
peroxidase P7
protein LIGHT-DEPENDENT SHORT HYPOCOTYLS 10-like isoform X1
rhamnose biosynthetic-like enzyme
transcription factor HEC2
pectin acetylesterase 12-like
AES87268.1 DUF1499 family protein
cytochrome P450 monooxygenase
QCD86001.1 DNA-3-methyladenine glycosylase I
organic cation/carnitine transporter 3
MLP-like protein 43
MLP-like protein 43

For Review Only

caffeoylshikimate esterase-like isoform X1
cyclic dof factor 1-like isoform X1
calcium and calcium/calmodulin-dependent serine/threonine-protein kinase DMI-3
staphylococcal nuclease domain-containing protein 1
Clavata3/ESR (CLE) gene family member
cytosolic sulfotransferase 15
UPF0481 protein At3g47200-like
11-oxo-beta-amyrin 30-oxidase-like isoform X1
cysteine-rich receptor-like protein kinase 10 isoform X1
ABC transporter A family member 8-like
WAT1-related protein At5g07050-like
protein LNK3
uncharacterized protein LOC101493795
nudix hydrolase 16, mitochondrial isoform X1
U-box domain-containing protein 4
putative disease resistance protein At3g14460
E3 ubiquitin-protein ligase SINAT5-like isoform X1
annexin D4
protein DETOXIFICATION 43-like
---NA---
DNA damage-repair/toleration protein DRT100
LOB domain-containing protein 19-like
peptidyl-prolyl cis-trans isomerase CYP18-1
MADS-box protein SOC1
transcription factor TCP9
UDP-glycosyltransferase 1
plant UBX domain-containing protein 9
BON1-associated protein 2-like
BON1-associated protein 2-like
KEH18862.1 acyl-CoA thioesterase
protein KTI12 homolog
KEH18823.1 transferring glycosyl group transferase
mitogen-activated protein kinase homolog MMK2
BAG family molecular chaperone regulator 6
probable glutathione S-transferase
probable WRKY transcription factor 53
centromeric histone 3
cyclic nucleotide-gated ion channel 1
putative RING-H2 finger protein ATL21A
transcription factor bHLH13-like
transcription factor MYB8-like
cytochrome P450 78A3
KEH18596.1 methyltransferase, putative
transmembrane protein, putative
uncharacterized protein LOC101488730
cyclic nucleotide-gated ion channel 1-like

For Review Only

NAC domain-containing protein 69-like

uncharacterized calcium-binding protein At1g02270-like isoform X1

---NA---

KEH18523.1 K+-H+ exchange-like protein

uncharacterized protein LOC101490868 isoform X1

pectinesterase 3-like

---NA---

---NA---

17.4 kDa class III heat shock protein

protein TIFY 11B

F-box protein SKP2B-like isoform X2

putative receptor-like protein kinase At1g72540

probable GTP diphosphokinase RSH2, chloroplastic

germin-like protein 8-14

calumenin-B-like isoform X1

calumenin-B-like isoform X1

protein BIG GRAIN 1-like B

protein Brevis radix-like 2

---NA---

putative PGG domain-containing protein

uncharacterized protein LOC101494327 isoform X2

aminopeptidase M1

linoleate 9S-lipoxygenase-like

coatomer subunit zeta-3-like

beta-fructofuranosidase, cell wall isozyme-like

NDR1/HIN1-like protein 26

inorganic pyrophosphatase 2-like

mediator of RNA polymerase II transcription subunit 6 isoform X1

nuclear poly(A) polymerase 4-like

uncharacterized protein LOC101504211

AET01486.1 Avr9/Cf-9 rapidly elicited protein

---NA---

membrane protein of ER body-like protein

vacuolar sorting protein 3 isoform X1

UV-B-induced protein At3g17800, chloroplastic

probable glucan endo-1,3-beta-glucosidase A6

transcription factor bHLH62-like

transmembrane protein, putative

probably inactive leucine-rich repeat receptor-like protein kinase At5g48380 isoform X1

FRIGIDA-like protein 3

---NA---

KEH18002.1 stress-induced protein, putative

putative RNA helicase

dynamin-related protein 1E

putative invertase inhibitor

actin-related protein 4

For Review Only

KEH17921.1 cyclin p4

probable WRKY transcription factor 12

KEH17865.1 plastocyanin-like domain protein

ethylene-responsive transcription factor 2-like

protein PIN-LIKES 3-like isoform X1

hydroquinone glucosyltransferase

uncharacterized protein LOC101500803

---NA---

mannan endo-1,4-beta-mannosidase 6-like

hypothetical protein MTR_4g133627

uncharacterized protein LOC101491481 isoform X1

---NA---

hypothetical protein MTR_4g129580

Ribosomal protein S10

hypothetical protein MtrunA17_Chr4g0065281

---NA---

---NA---

ABC transporter B family member 9-like isoform X2

---NA---

uncharacterized protein LOC101495054

auxin-responsive protein SAUR50

---NA---

---NA---

---NA---

---NA---

---NA---

---NA---

---NA---

---NA---

---NA---

hypothetical protein MTR_4g133627

---NA---

---NA---

---NA---

---NA---

O-acyltransferase WSD1-like

---NA---

---NA---

leucine-rich repeat protein 2-like

dual-specificity RNA methyltransferase RlmN 1-like protein

uncharacterized protein LOC101500065

uncharacterized GPI-anchored protein At3g06035-like

---NA---

heat shock 70 kDa protein 8

putative 12-oxophytodienoate reductase 11

type IV inositol polyphosphate 5-phosphatase 7

For Review Only

AES93764.1 PLATZ transcription factor family protein
4-coumarate--CoA ligase 2
ERAD-associated E3 ubiquitin-protein ligase HRD1B isoform X1
protein EXPORTIN 1A-like
AES93837.2 rubredoxin family protein
Cytochrome c-2
ethylene-responsive transcription factor ERF021-like
probable acyl-activating enzyme 1, peroxisomal
AES93923.1 hexokinase
ethylene-responsive transcription factor ERF061
---NA---
ribosome biogenesis ATPase RIX7-like isoform X2
AES94019.1 glycosyl hydrolase family 9 protein
uncharacterized protein LOC101492610
hypothetical protein MTR_5g010060
probable E3 ubiquitin-protein ligase RHA1A
transcription factor MYB3R-3-like isoform X1
F-box protein PP2-A12-like
protein DETOXIFICATION 49-like
dehydration-responsive element-binding protein 1E-like
hydroxymethylglutaryl-CoA synthase isoform X1
probable sodium/metabolite cotransporter BASS5, chloroplastic
putative glucuronosyltransferase PGSIP8
probable aquaporin TIP2-2
formyltetrahydrofolate deformylase 1, mitochondrial-like isoform X1
homeobox-leucine zipper protein HAT4-like
ethylene-responsive transcription factor 1A-like
phospholipase A1-Ibeta2, chloroplastic-like
protein TIFY 10a-like
probable serine/threonine-protein kinase PBL19
FCS-Like Zinc finger 2-like
haloacid dehalogenase-like hydrolase domain-containing protein Sgpp
type I inositol polyphosphate 5-phosphatase 2-like
uncharacterized protein LOC101504661
transcription factor bHLH18-like
transcription factor bHLH18-like
leucine-rich repeat receptor-like serine/threonine-protein kinase BAM1
LRR receptor-like serine/threonine-protein kinase HSL2
phytosulfokines 3
uncharacterized protein LOC101514532
probable amino-acid acetyltransferase NAGS2, chloroplastic isoform X1
calcium-transporting ATPase 2, plasma membrane-type-like
---NA---
glucan endo-1,3-beta-glucosidase 12
queuosine salvage protein-like
protein CONSERVED IN THE GREEN LINEAGE AND DIATOMS 27, chloroplastic isoform X2

For Review Only

cytochrome P450 81E8-like
microtubule-associated protein RP/EB family member 1C
SUMO-conjugating enzyme SCE1
floral homeotic protein APETALA 2-like isoform X4
AES94691.1 triacylglycerol lipase-like protein
zeaxanthin epoxidase, chloroplastic-like isoform X1
heat stress transcription factor B-1
AES94720.1 BEST plant protein match is: (TAIR:plant.1) protein, putative
methionine aminopeptidase 1D, chloroplastic/mitochondrial
AES94732.1 P-loop nucleoside triphosphate hydrolase superfamily protein
RHN54051.1 putative encoded peptide
LOB domain-containing protein 36-like
---NA---
Peroxidase 7
L-ascorbate oxidase homolog
hypothetical protein L195_g005878
branched-chain amino acid aminotransferase
uncharacterized protein LOC101511835
uncharacterized protein LOC105851093
cytochrome P450 71D8-like
cytochrome P450 71D8-like
cytochrome P450 71D8-like
SYM10 protein
seven transmembrane protein 1
BES1/BZR1 homolog protein 2
UDP-glycosyltransferase 72E1
homeobox-leucine zipper protein ATHB-40
AES94931.1 plant/T3A5-60 protein
cytochrome P450 90B1-like
AES94993.1 laccase/diphenol oxidase family protein
putative glucose-6-phosphate 1-epimerase
isoflavone reductase
floral homeotic protein DEFICIENS-like isoform X1
uncharacterized protein LOC101498564
calcium-dependent protein kinase 28
vacuolar protein
ferredoxin--NADP reductase, leaf isozyme, chloroplastic
thaumatin-like protein 1
thiosulfate sulfurtransferase 18-like isoform X1
histidine kinase 1 isoform X2
rac-like GTP-binding protein ARAC1-like
uncharacterized protein LOC101489897
ganglioside-induced differentiation-associated protein 2
probable serine/threonine-protein kinase PIX13
HMG1/2-like protein
RING-H2 finger protein ATL8

For Review Only

3-hydroxy-3-methylglutaryl-coenzyme A reductase 1-like
hypothetical protein L195_g029980
protein PIN-LIKES 3-like isoform X1
subtilisin-like protease SBT3.9 isoform X1
probable carotenoid cleavage dioxygenase 4, chloroplastic
ABC transporter G family member 14
abscisic acid 8'-hydroxylase CYP707A2-like
AES95465.1 transmembrane protein, putative
fatty alcohol:caffeoyl-CoA acyltransferase
SH3 domain-containing protein 2
uncharacterized protein LOC101505738 isoform X1
AES95555.1 kinesin-like protein
protein CYCLOPS isoform X2
probable methyltransferase PMT21
transmembrane protein, putative
lysine-specific demethylase JMJ706 isoform X1
transcription factor MYB39
cinnamoyl-CoA reductase-like SNL6
protein TORNADO 2-like
putative gamma-glutamylcyclotransferase At3g02910
hydroxyproline-rich glycoprotein
probable mannitol dehydrogenase
receptor-like cytosolic serine/threonine-protein kinase RBK1
BTB/POZ domain-containing protein At1g67900-like
lysM domain receptor-like kinase 3
AES96182.1 transmembrane protein, putative
AMSH-like ubiquitin thioesterase 2 isoform X2
polygalacturonase-1 non-catalytic subunit beta-like
sucrose transport protein SUC8-like
putative xenobiotic-transporting ATPase
---NA---
tubby-like F-box protein 8
uncharacterized protein LOC101494894
steroid 5-alpha-reductase DET2-like
very-long-chain enoyl-CoA reductase-like
mitochondrial phosphate carrier protein 3, mitochondrial-like
KEH25656.1 tetraspanin family protein
MLO-like protein 2
chlorophyll a-b binding protein 6, chloroplastic isoform X1
UDP-glycosyltransferase 1-like
UDP-glycosyltransferase 1
---NA---
heavy metal-associated isoprenylated plant protein 39-like
beta-carotene hydroxylase 2, chloroplastic-like isoform X1
UDP-glycosyltransferase 1
silicon efflux transporter LSI2

For Review Only

heavy metal-associated isoprenylated plant protein 20-like
tripeptidyl-peptidase 2-like isoform X3
cytokinin hydroxylase
heavy metal-associated isoprenylated plant protein 39-like isoform X3
---NA---
uncharacterized protein LOC101506614
CEN-like protein 1 isoform X2
pentatricopeptide repeat-containing protein At4g32430, mitochondrial-like
F-box protein PP2-A13-like
gibberellin 20 oxidase 1-like
---NA---
uncharacterized protein LOC101508329
APO protein 1, chloroplastic
PHD finger protein At1g33420-like
probable carboxylesterase 18
uncharacterized oxidoreductase At4g09670-like
Pentatricopeptide repeat-containing protein DOT4, chloroplastic
alcohol dehydrogenase-like 6
uncharacterized protein LOC101496282
KEH21082.1 hydroxyproline-rich glycoprotein family protein
kunitz trypsin inhibitor 2-like
probable ADP-ribosylation factor GTPase-activating protein AGD15
early nodulin-55-1-like
transcription factor MYB52
TMV resistance protein N-like isoform X1
methylecgonone reductase-like
ethylene-responsive transcription factor ERN2-like
U-box domain-containing protein 21-like
mitogen-activated protein kinase kinase 9
chromosome transmission fidelity protein 8 homolog
protein TIFY 6B isoform X1
putative tagatose-bisphosphate aldolase
transcription factor MYB21-like
uncharacterized protein LOC101495844
AES76695.1 plant synaptotagmin
twinkle homolog protein, chloroplastic/mitochondrial
---NA---
probable ADP-ribosylation factor GTPase-activating protein AGD14 isoform X3
multiprotein-bridging factor 1c
hypothetical protein TSUD_141100
proline-rich receptor-like protein kinase PERK1
probable protein ABIL5 isoform X2
hypothetical protein L195_g039649
protein SPEAR3-like
E3 ubiquitin-protein ligase ATL42
probable glycosyltransferase At3g07620

For Review Only

dehydration-responsive element-binding protein 3-like
lachrymatory-factor synthase-like
KEH27184.1 Avr9/Cf-9 rapidly elicited protein
---NA---
protein DETOXIFICATION 51-like
AES76974.1 DUF1005 family protein
zinc finger CCCH domain-containing protein 20
transcription factor MYBS3-like
1-aminocyclopropane-1-carboxylate oxidase 1
---NA---
pentatricopeptide repeat-containing protein At4g20740-like
---NA---
uncharacterized protein LOC101493476
cysteine-rich and transmembrane domain-containing protein WIH2
putative receptor-like protein kinase At4g00960 isoform X1
putative receptor-like protein kinase At4g00960 isoform X1
probable membrane-associated kinase regulator 1
sugar transport protein 13
thermospermine synthase ACAULIS5-like
RHN53309.1 putative ribosomal protein L18
geraniol 8-hydroxylase-like
AES93673.1 Serine/Threonine-kinase STN7, related protein, putative
TOM1-like protein 1
chalcone synthase 1
chalcone synthase
endoplasmic reticulum oxidoreductin-1-like
magnesium-chelatase subunit ChlI chloroplastic-like
MYB-like transcription factor ETC3
probable inactive receptor kinase At4g23740 isoform X1
Sec-independent protein translocase protein TatB
probable aquaporin PIP1-2
probable copper-transporting ATPase HMA5
thaumatin-like protein 1
protein P21-like
heat stress transcription factor B-2b
phospholipase D beta 1
SNARE-interacting protein KEULE-like isoform X1
non-specific lipid transfer protein GPI-anchored 1-like
syntaxin-related protein KNOLLE
hsp70-Hsp90 organizing protein 3-like
L-lactate dehydrogenase A-like
probable aspartyl protease At4g16563
metalloendoproteinase 4-MMP
VAN3-binding protein
peroxisomal acyl-coenzyme A oxidase 1
homeobox-leucine zipper protein HAT4-like

For Review Only

50S ribosomal protein L19, chloroplastic
expansin-like B1
AES94355.1 peptidyl-prolyl cis-trans isomerase
beta-amylase 3, chloroplastic
FCS-Like Zinc finger 2-like
---NA---
two-component response regulator-like APRR2 isoform X1
myosin-G heavy chain
protein NRT1/ PTR FAMILY 7.3
glycine cleavage system H protein, mitochondrial
malate dehydrogenase, glyoxysomal isoform X2
1-aminocyclopropane-1-carboxylate synthase 3-like
bZIP transcription factor RISBZ3-like
protein PHLOEM PROTEIN 2-LIKE A10-like
VQ motif-containing protein 29-like
adenylyl-sulfate kinase
mitochondrial outer membrane protein porin 2
calcium-transporting ATPase 2, plasma membrane-type
peroxidase 51-like
probable indole-3-acetic acid-amido synthetase GH3.1
ethylene-responsive transcription factor ERF011
soyasapogenol B glucuronide galactosyltransferase
patatin-like protein 5
peroxidase 10
methylesterase 3-like
synechocystis YCF37-like protein
BEL1-like homeodomain protein 4
cytochrome P450 71D8-like
---NA---
putative galacturonosyltransferase 1-like protein
heterodimeric geranylgeranyl pyrophosphate synthase large subunit 1, chloroplastic
AES94929.1 polyol transporter-like protein, putative
cytochrome P450 71D8-like
plant/MUD21-2 protein
hypothetical protein MTR_5g020090
putative major facilitator, sugar transporter, major facilitator superfamily
MYB family transcription factor
---NA---
AAA-ATPase At5g17760-like isoform X2
protein RTE1-HOMOLOG
beta-galactosidase 3
protein FMP32, mitochondrial-like
zinc finger CCCH domain-containing protein 44-like isoform X1
B-box zinc finger protein 18
AES95089.2 lactoylglutathione lyase/glyoxalase I family protein
histone H1

For Review Only

uncharacterized protein LOC101501661
---NA---
TLC domain-containing protein At5g14285
protein NUCLEAR FUSION DEFECTIVE 4-like
---NA---
histidine kinase 1 isoform X1
SKP1-like protein 1A isoform B
aspartyl protease family protein 1-like
DVL family protein
hypothetical protein CCACVL1_09133
thiosulfate sulfurtransferase 16, chloroplastic-like isoform X1
lysine histidine transporter-like 8
lysine histidine transporter-like 8
probable serine/threonine-protein kinase PIX13
---NA---
protein PIN-LIKES 7-like
monosaccharide-sensing protein 2-like
diacylglycerol O-acyltransferase 2D
uncharacterized protein LOC101509048
kinesin-like protein KIN-14I
heavy metal-associated isoprenylated plant protein 22-like
AES95460.1 transmembrane protein, putative
pectin acetylesterase 8-like isoform X1
probable serine/threonine-protein kinase PBL15
hypothetical protein TSUD_236100
SH3 domain-containing protein 2
growth-regulating factor 5-like
elongation factor 1-alpha-like
cellulose synthase-like protein D3
AES95795.2 transmembrane protein, putative
unnamed protein product
TPR domain protein
cinnamoyl-CoA reductase 1-like protein
uncharacterized protein LOC113788058
mitochondrial carnitine/acylcarnitine carrier-like protein
phosphoenolpyruvate carboxykinase (ATP)
---NA---
transcription factor bHLH93-like isoform X1
serine hydroxymethyltransferase, mitochondrial
agamous-like MADS-box protein AGL21 isoform X2
probable mannitol dehydrogenase
probable mannitol dehydrogenase
probable mannitol dehydrogenase
AP2-like ethylene-responsive transcription factor AIL6
uncharacterized protein LOC101496149
O-fucosyltransferase 39

For Review Only

mitochondrial uncoupling protein 5

---NA---

uncharacterized protein LOC11407074

AES96075.1 PB1 domain protein

glutamate--glyoxylate aminotransferase 2

alpha/beta fold hydrolase

homeobox protein knotted-1-like 6

pentatricopeptide repeat-containing protein At2g40240, mitochondrial-like isoform X1

vacuolar iron transporter homolog 2-like

abscisic stress-ripening protein 3-like

UDP-glycosyltransferase 1

UDP-glycosyltransferase 1-like

---NA---

heavy metal-associated isoprenylated plant protein 39-like

heavy metal-associated isoprenylated plant protein 39-like

heavy metal-associated isoprenylated plant protein 39-like

heavy metal-associated domain protein

heat shock protein 83

---NA---

---NA---

transcription factor LAF1-like

E3 ubiquitin-protein ligase ATL6-like

probable methyltransferase PMT16

serine/arginine-rich splicing factor RS40-like isoform X2

protein DETOXIFICATION 49-like

ubiquitin carboxyl-terminal hydrolase 6 isoform X1

---NA---

probable fructokinase-7 isoform X1

transcription factor bHLH118-like

CDGSH iron-sulfur domain-containing protein NEET

NAD-dependent protein deacetylase HST1

chlorophyll a-b binding protein CP26, chloroplastic

kunitz-type trypsin inhibitor-like 2 protein-like precursor

kunitz-type trypsin inhibitor-like 1 protein

extradiol ring-cleavage dioxygenase-like

pre-mRNA-splicing factor SPF27 homolog

TMV resistance protein N

probable serine/threonine-protein kinase DDB_G0282963

beta-glucosidase 12-like

beta-glucosidase 12-like

PNX86176.1 miraculin

kunitz trypsin inhibitor 2-like

GEM-like protein 4

protein REVEILLE 7-like

beta-amylase 1, chloroplastic

probable cyclic nucleotide-gated ion channel 20, chloroplastic

For Review Only

protein-S-isoprenylcysteine O-methyltransferase A-like

ethylene-responsive transcription factor ERN2-like

purple acid phosphatase 17-like

KEH26746.1 carbohydrate-binding protein of the ER protein

NDR1/HIN1-like protein 10

phosphoethanolamine N-methyltransferase-like isoform X1

beta-glucosidase 44-like

ras-related protein RABA6a-like

zinc finger protein VAR3, chloroplastic

Multi antimicrobial extrusion protein

---NA---

reticulon-like protein B9

LEAF RUST 10 DISEASE-RESISTANCE LOCUS RECEPTOR-LIKE PROTEIN KINASE-like 1.2

LEAF RUST 10 DISEASE-RESISTANCE LOCUS RECEPTOR-LIKE PROTEIN KINASE-like 2.3 isoform X2

transcription factor BEE 3-like

uncharacterized protein LOC101495844

aspartyl protease APCB1

cytochrome P450 CYP736A12-like

cytochrome P450 CYP736A12-like

strigolactone esterase D14

leucine-rich repeat extensin-like protein 4

probable sulfate transporter 3.5

aldehyde dehydrogenase family 2 member C4

putative arabinogalactan peptide, AGP

polyol transporter 5 isoform X1

uncharacterized protein LOC105852725

hypothetical protein MtrunA17_Chr6g0486621

lachrymatory-factor synthase-like

mitochondrial import inner membrane translocase subunit TIM17-2-like

2-oxoglutarate-Fe(II) type oxidoreductase hxnY-like isoform X2

putative ABC transporter B family member 8

programmed cell death protein 4-like

uncharacterized protein LOC101493710

acid phosphatase 1

probable caffeoyl-CoA O-methyltransferase At4g26220

sericin 1

probable phospholipase A2 homolog 1

hypothetical protein MTR_5g010580

---NA---

hypothetical protein MTR_5g016470

---NA---

---NA---

---NA---

---NA---

---NA---

---NA---

For Review Only

NADH dehydrogenase subunit 5 (mitochondrion)
---NA---
hypothetical protein PHAVU_004G148400g
---NA---
transmembrane protein, putative
E3 ubiquitin-protein ligase ATL42
---NA---
orf187 (mitochondrion)
cytochrome c oxidase subunit 1 (mitochondrion)
hypothetical protein DEO72_LG2g3846
---NA---
---NA---
aquaporin NIP6-1-like
ribosomal protein S3 (mitochondrion)
hypothetical protein MTR_1g060240
E3 ubiquitin-protein ligase DIS1
probable terpene synthase 2
---NA---
SKP1-like protein 14
protein trichome birefringence-like 19
anamorsin homolog
pentatricopeptide repeat-containing protein At1g63330-like
DUF241 domain protein
hypothetical protein L195_g051726
---NA---
---NA---
ribosomal protein S3 (mitochondrion)
NADH dehydrogenase subunit 9 (mitochondrion)
polyadenylate-binding protein-interacting protein 4-like
E3 ubiquitin-protein ligase MARCH8-like
hypothetical protein CR513_00088, partial
---NA---
splicing factor 3B subunit 2
cellulose synthase-like protein E1
serine carboxypeptidase-like 45 isoform X2
---NA---
F-box protein At5g51380-like
---NA---
60S acidic ribosomal protein P1-like
cytochrome P450 82C4-like
isoflavone 7-O-glucosyltransferase 1-like
putative MO25-like protein At5g47540
Enod93 protein
TOM1-like protein 5 isoform X1
RING-finger ubiquitin ligase
RZB97188.1 DNA-3-methyladenine glycosylase 1

For Review Only

peroxisomal 2,4-dienoyl-CoA reductase
hypothetical protein TSUD_86250
ATP-dependent 6-phosphofructokinase 6-like
tonoplast dicarboxylate transporter-like
probable calcium-binding protein CML27
probable serine/threonine-protein kinase PBL7
indole-3-acetic acid-induced protein ARG7-like
'photosystem I P700 apoprotein A1 (chloroplast)
acetyl-CoA carboxylase carboxyltransferase beta subunit
hypothetical chloroplast RF34 (chloroplast)
photosystem II CP47 chlorophyll apoprotein
hypothetical protein DEO72_LG7g1332
60S acidic ribosomal protein P1-like
---NA---
granule-bound starch synthase 1, chloroplastic/amyloplastic-like
NADH dehydrogenase subunit 7
suppressor of mec-8 and unc-52 protein homolog 1-like
uncharacterized protein LOC101501044 isoform X2
dirigent protein 9-like
peroxidase 3
phenolic glucoside malonyltransferase 1-like
---NA---
uncharacterized protein LOC100194361
---NA---
cytochrome c oxidase subunit 6b-2
---NA---
protein SODIUM POTASSIUM ROOT DEFECTIVE 2
ras-related protein RHN1 isoform X1
hypothetical protein L195_g030538
alcohol dehydrogenase-like 5 isoform X1
kinesin-like protein KIN-8A isoform X1
ABC transporter B family member 1
uncharacterized protein LOC113786764
probable protein phosphatase 2C 42

For Review Only

Supplemental Table S3. Functional annotation (Biological Process Category) of DEGs identified f chickpea genotypes under control and stress conditions.

GO ID	Term
GO:0050896	response to stimulus
GO:0042221	response to chemical
GO:0006355	regulation of transcription, DNA-templated
GO:1903506	regulation of nucleic acid-templated transcription
GO:2001141	regulation of RNA biosynthetic process
GO:0070887	cellular response to chemical stimulus
GO:0051252	regulation of RNA metabolic process
GO:0019219	regulation of nucleobase-containing compound metabolic process
GO:0042743	hydrogen peroxide metabolic process
GO:0042744	hydrogen peroxide catabolic process
GO:0006351	transcription, DNA-templated
GO:0097659	nucleic acid-templated transcription
GO:0032774	RNA biosynthetic process
GO:0072593	reactive oxygen species metabolic process
GO:0007154	cell communication
GO:0050794	regulation of cellular process
GO:0009725	response to hormone
GO:0009719	response to endogenous stimulus
GO:0007165	signal transduction
GO:0023052	signaling
GO:0050789	regulation of biological process
GO:0006952	defense response
GO:0010033	response to organic substance
GO:0097237	cellular response to toxic substance
GO:1990748	cellular detoxification
GO:0006979	response to oxidative stress
GO:0098754	detoxification
GO:0009755	hormone-mediated signaling pathway
GO:0006950	response to stress
GO:0009694	jasmonic acid metabolic process
GO:0015698	inorganic anion transport
GO:0098869	cellular oxidant detoxification
GO:0032870	cellular response to hormone stimulus
GO:0071495	cellular response to endogenous stimulus
GO:1901700	response to oxygen-containing compound
GO:1901362	organic cyclic compound biosynthetic process
GO:0015979	photosynthesis
GO:0065007	biological regulation
GO:0071310	cellular response to organic substance
GO:0051716	cellular response to stimulus
GO:0042537	benzene-containing compound metabolic process
GO:0019438	aromatic compound biosynthetic process

For Review Only

GO:0034654	nucleobase-containing compound biosynthetic process
GO:0009636	response to toxic substance
GO:0031323	regulation of cellular metabolic process
GO:0009889	regulation of biosynthetic process
GO:0009696	salicylic acid metabolic process
GO:0031326	regulation of cellular biosynthetic process
GO:2000112	regulation of cellular macromolecule biosynthetic process
GO:0055085	transmembrane transport
GO:0009733	response to auxin
GO:0010556	regulation of macromolecule biosynthetic process
GO:0006629	lipid metabolic process
GO:0018130	heterocycle biosynthetic process
GO:0018958	phenol-containing compound metabolic process
GO:0080090	regulation of primary metabolic process
GO:0051171	regulation of nitrogen compound metabolic process
GO:0071365	cellular response to auxin stimulus
GO:0009734	auxin-activated signaling pathway
GO:0015977	carbon fixation
GO:0009607	response to biotic stimulus
GO:0010167	response to nitrate
GO:0022900	electron transport chain
GO:0051346	negative regulation of hydrolase activity
GO:0043086	negative regulation of catalytic activity
GO:0019222	regulation of metabolic process
GO:0044092	negative regulation of molecular function
GO:1901615	organic hydroxy compound metabolic process
GO:0071554	cell wall organization or biogenesis
GO:0008202	steroid metabolic process
GO:0006091	generation of precursor metabolites and energy
GO:0051259	protein complex oligomerization
GO:0009768	photosynthesis, light harvesting in photosystem I
GO:0015706	nitrate transport
GO:0006817	phosphate ion transport
GO:0009266	response to temperature stimulus
GO:0009695	jasmonic acid biosynthetic process
GO:0006720	isoprenoid metabolic process
GO:0060255	regulation of macromolecule metabolic process
GO:0009808	lignin metabolic process
GO:0010468	regulation of gene expression
GO:0008610	lipid biosynthetic process
GO:0006721	terpenoid metabolic process
GO:0009765	photosynthesis, light harvesting
GO:0032515	negative regulation of phosphoprotein phosphatase activity
GO:0010923	negative regulation of phosphatase activity
GO:0006468	protein phosphorylation
GO:1901606	alpha-amino acid catabolic process

For Review Only

GO:0098542	defense response to other organism
GO:0035305	negative regulation of dephosphorylation
GO:0035308	negative regulation of protein dephosphorylation
GO:0032875	regulation of DNA endoreduplication
GO:0006694	steroid biosynthetic process
GO:0010563	negative regulation of phosphorus metabolic process
GO:0045936	negative regulation of phosphate metabolic process
GO:0016054	organic acid catabolic process
GO:0044419	biological process involved in interspecies interaction between organisms
GO:0033993	response to lipid
GO:0006793	phosphorus metabolic process
GO:1901701	cellular response to oxygen-containing compound
GO:0042542	response to hydrogen peroxide
GO:0043207	response to external biotic stimulus
GO:0051707	response to other organism
GO:0010035	response to inorganic substance
GO:0008299	isoprenoid biosynthetic process
GO:0098661	inorganic anion transmembrane transport
GO:0072330	monocarboxylic acid biosynthetic process
GO:0044282	small molecule catabolic process
GO:0019748	secondary metabolic process
GO:1902358	sulfate transmembrane transport
GO:0009408	response to heat
GO:0008272	sulfate transport
GO:0006749	glutathione metabolic process
GO:0006796	phosphate-containing compound metabolic process
GO:0045229	external encapsulating structure organization
GO:0016311	dephosphorylation
GO:0071396	cellular response to lipid
GO:0071555	cell wall organization
GO:0006556	S-adenosylmethionine biosynthetic process
GO:0042126	nitrate metabolic process
GO:0042128	nitrate assimilation
GO:2001057	reactive nitrogen species metabolic process
GO:0010337	regulation of salicylic acid metabolic process
GO:0046189	phenol-containing compound biosynthetic process
GO:0009697	salicylic acid biosynthetic process
GO:0010411	xyloglucan metabolic process
GO:0009644	response to high light intensity
GO:0080142	regulation of salicylic acid biosynthetic process
GO:0016125	sterol metabolic process
GO:1901617	organic hydroxy compound biosynthetic process
GO:0009698	phenylpropanoid metabolic process
GO:0120254	olefinic compound metabolic process
GO:0019684	photosynthesis, light reaction
GO:0042023	DNA endoreduplication

For Review Only

GO:0032787	monocarboxylic acid metabolic process
GO:0016114	terpenoid biosynthetic process
GO:0000041	transition metal ion transport
GO:0009628	response to abiotic stimulus
GO:0009685	gibberellin metabolic process
GO:0009605	response to external stimulus
GO:0016310	phosphorylation
GO:0009645	response to low light intensity stimulus
GO:0006970	response to osmotic stress
GO:0097577	sequestering of iron ion
GO:0006880	intracellular sequestering of iron ion
GO:0051238	sequestering of metal ion
GO:0015689	molybdate ion transport
GO:0009074	aromatic amino acid family catabolic process
GO:0009065	glutamine family amino acid catabolic process
GO:0031407	oxylipin metabolic process
GO:0031408	oxylipin biosynthetic process
GO:0046395	carboxylic acid catabolic process
GO:0042546	cell wall biogenesis
GO:0016036	cellular response to phosphate starvation
GO:0046274	lignin catabolic process
GO:0046271	phenylpropanoid catabolic process
GO:0071669	plant-type cell wall organization or biogenesis
GO:0009738	abscisic acid-activated signaling pathway
GO:0016101	diterpenoid metabolic process
GO:0016042	lipid catabolic process
GO:0005975	carbohydrate metabolic process
GO:0052548	regulation of endopeptidase activity
GO:0010466	negative regulation of peptidase activity
GO:0045861	negative regulation of proteolysis
GO:0010951	negative regulation of endopeptidase activity
GO:0032973	amino acid export across plasma membrane
GO:1903789	regulation of amino acid transmembrane transport
GO:0042402	cellular biogenic amine catabolic process
GO:0032890	regulation of organic acid transport
GO:0097306	cellular response to alcohol
GO:0051955	regulation of amino acid transport
GO:0051952	regulation of amine transport
GO:0071215	cellular response to abscisic acid stimulus
GO:0009310	amine catabolic process
GO:0080143	regulation of amino acid export
GO:0015837	amine transport
GO:0044036	cell wall macromolecule metabolic process
GO:0015671	oxygen transport
GO:0015669	gas transport
GO:0009809	lignin biosynthetic process

For Review Only

GO:0009642	response to light intensity
GO:0080163	regulation of protein serine/threonine phosphatase activity
GO:0044273	sulfur compound catabolic process
GO:0071941	nitrogen cycle metabolic process
GO:0051248	negative regulation of protein metabolic process
GO:0032269	negative regulation of cellular protein metabolic process
GO:0009617	response to bacterium
GO:0030001	metal ion transport
GO:0009063	cellular amino acid catabolic process
GO:0052547	regulation of peptidase activity
GO:0016102	diterpenoid biosynthetic process
GO:0009686	gibberellin biosynthetic process
GO:0006012	galactose metabolic process
GO:0043666	regulation of phosphoprotein phosphatase activity
GO:0006790	sulfur compound metabolic process
GO:0034220	ion transmembrane transport
GO:0035304	regulation of protein dephosphorylation
GO:0010921	regulation of phosphatase activity
GO:0097305	response to alcohol
GO:0090329	regulation of DNA-dependent DNA replication
GO:0006828	manganese ion transport
GO:0009737	response to abscisic acid
GO:0009813	flavonoid biosynthetic process
GO:0005992	trehalose biosynthetic process
GO:0071421	manganese ion transmembrane transport
GO:0009627	systemic acquired resistance
GO:0010410	hemicellulose metabolic process
GO:0010200	response to chitin
GO:0009651	response to salt stress
GO:0042436	indole-containing compound catabolic process
GO:0006562	proline catabolic process
GO:0006569	tryptophan catabolic process
GO:0046218	indolalkylamine catabolic process
GO:0034329	cell junction assembly
GO:0007043	cell-cell junction assembly
GO:0006308	DNA catabolic process
GO:0019685	photosynthesis, dark reaction
GO:0019253	reductive pentose-phosphate cycle
GO:0019441	tryptophan catabolic process to kynurenine
GO:0034755	iron ion transmembrane transport
GO:0006022	aminoglycan metabolic process
GO:0009299	mRNA transcription
GO:0010089	xylem development
GO:1903959	regulation of anion transmembrane transport
GO:0015849	organic acid transport
GO:0009739	response to gibberellin

For Review Only

GO:0005991	trehalose metabolic process
GO:0120255	olefinic compound biosynthetic process
GO:0016143	S-glycoside metabolic process
GO:0019760	glucosinolate metabolic process
GO:0019757	glycosinolate metabolic process
GO:0035435	phosphate ion transmembrane transport
GO:0010565	regulation of cellular ketone metabolic process
GO:0051336	regulation of hydrolase activity
GO:0098660	inorganic ion transmembrane transport
GO:0016128	phytosteroid metabolic process
GO:0016129	phytosteroid biosynthetic process
GO:0016131	brassinosteroid metabolic process
GO:0016132	brassinosteroid biosynthetic process
GO:0016108	tetraterpenoid metabolic process
GO:0016116	carotenoid metabolic process
GO:0120178	steroid hormone biosynthetic process
GO:0035303	regulation of dephosphorylation
GO:0009409	response to cold
GO:0071249	cellular response to nitrate
GO:1901699	cellular response to nitrogen compound
GO:1905156	negative regulation of photosynthesis
GO:0010270	photosystem II oxygen evolving complex assembly
GO:0072526	pyridine-containing compound catabolic process
GO:0006842	tricarboxylic acid transport
GO:0072348	sulfur compound transport
GO:0042538	hyperosmotic salinity response
GO:0006972	hyperosmotic response
GO:0010110	regulation of photosynthesis, dark reaction
GO:0010196	nonphotochemical quenching
GO:0005976	polysaccharide metabolic process
GO:1990066	energy quenching
GO:0080152	regulation of reductive pentose-phosphate cycle
GO:0080153	negative regulation of reductive pentose-phosphate cycle
GO:1902170	cellular response to reactive nitrogen species
GO:0015746	citrate transport
GO:1901071	glucosamine-containing compound metabolic process
GO:0046500	S-adenosylmethionine metabolic process
GO:0031324	negative regulation of cellular metabolic process
GO:0006816	calcium ion transport
GO:0070588	calcium ion transmembrane transport
GO:0010383	cell wall polysaccharide metabolic process
GO:0009611	response to wounding
GO:1901072	glucosamine-containing compound catabolic process
GO:0070973	protein localization to endoplasmic reticulum exit site
GO:0010207	photosystem II assembly
GO:0046348	amino sugar catabolic process

For Review Only

GO:1902644	tertiary alcohol metabolic process
GO:0009687	abscisic acid metabolic process
GO:0043288	apocarotenoid metabolic process
GO:0006026	aminoglycan catabolic process
GO:0006032	chitin catabolic process
GO:0006030	chitin metabolic process
GO:0006575	cellular modified amino acid metabolic process
GO:0042742	defense response to bacterium
GO:0000302	response to reactive oxygen species
GO:0030417	nicotianamine metabolic process
GO:0030418	nicotianamine biosynthetic process
GO:0016122	xanthophyll metabolic process
GO:0016103	diterpenoid catabolic process
GO:0034330	cell junction organization
GO:0072351	tricarboxylic acid biosynthetic process
GO:0030497	fatty acid elongation
GO:0009664	plant-type cell wall organization
GO:0009800	cinnamic acid biosynthetic process
GO:0009803	cinnamic acid metabolic process
GO:0045487	gibberellin catabolic process
GO:0045216	cell-cell junction organization
GO:1903409	reactive oxygen species biosynthetic process
GO:0009699	phenylpropanoid biosynthetic process
GO:0009812	flavonoid metabolic process
GO:1901698	response to nitrogen compound
GO:0016145	S-glycoside catabolic process
GO:0019759	glycosinolate catabolic process
GO:0019762	glucosinolate catabolic process
GO:0006073	cellular glucan metabolic process
GO:0018298	protein-chromophore linkage
GO:0044255	cellular lipid metabolic process

For Review Only

from different

P-value
5.81E-17
2.04E-15
1.82E-13
2.36E-13
2.36E-13
1.38E-12
1.63E-12
8.12E-12
1.50E-10
1.68E-10
3.96E-10
4.10E-10
4.46E-10
6.75E-10
9.27E-10
1.09E-09
2.52E-09
2.69E-09
2.91E-09
3.31E-09
1.20E-08
1.41E-08
1.99E-08
5.04E-08
5.04E-08
5.24E-08
5.98E-08
6.01E-08
6.19E-08
6.43E-08
6.58E-08
9.11E-08
1.02E-07
1.08E-07
1.12E-07
1.56E-07
2.12E-07
2.30E-07
3.01E-07
3.88E-07
4.00E-07
5.68E-07

For Review Only

6.73E-07
8.45E-07
1.00E-06
1.43E-06
1.48E-06
1.94E-06
2.06E-06
2.30E-06
2.38E-06
2.43E-06
3.07E-06
5.01E-06
5.39E-06
1.25E-05
1.32E-05
1.50E-05
1.50E-05
1.58E-05
1.90E-05
3.22E-05
4.30E-05
4.33E-05
7.67E-05
8.07E-05
8.23E-05
8.82E-05
9.23E-05
9.53E-05
1.12E-04
1.46E-04
1.51E-04
1.51E-04
1.66E-04
1.69E-04
1.89E-04
1.97E-04
2.00E-04
2.94E-04
3.60E-04
3.87E-04
4.67E-04
5.75E-04
5.93E-04
5.93E-04
6.05E-04
7.14E-04

For Review Only

8.18E-04
8.62E-04
8.62E-04
9.55E-04
0.00106089
0.00106089
0.00106089
0.00107989
0.00137441
0.00137821
0.00138782
0.00149885
0.00163329
0.00168625
0.00168625
0.00172034
0.00176006
0.00184516
0.00187498
0.00187498
0.00191064
0.00224007
0.00224007
0.00224007
0.00226163
0.00227108
0.00230034
0.00250461
0.00255224
0.00259911
0.00273055
0.00273055
0.00273055
0.00273055
0.00273055
0.00273055
0.00273055
0.00272255
0.00273055
0.00273055
0.002796
0.00306382
0.00324118
0.00332124
0.00336039
0.00355662

For Review Only

0.00366269
0.00408985
0.00408985
0.00448656
0.0045243
0.00503739
0.00532061
0.00565008
0.00587338
0.0062122
0.0062122
0.0062122
0.0062122
0.00648285
0.00648285
0.00668031
0.00668031
0.00722317
0.00776634
0.00806915
0.00806915
0.00806915
0.00853284
0.00882808
0.00954325
0.00999613
0.00999998
0.01053598
0.01053598
0.01053598
0.01053598
0.01111092
0.01111092
0.01111092
0.01111092
0.01091846
0.01111092
0.01111092
0.01091846
0.01111092
0.01111092
0.01111092
0.01162954
0.01193584
0.01193584
0.01193584

For Review Only

0.01193584
0.01193584
0.01179171
0.01193584
0.01236927
0.01236927
0.01247365
0.01260479
0.01325583
0.01325583
0.01444657
0.01444657
0.01444657
0.01468398
0.01569117
0.01597494
0.01610701
0.01610701
0.01659415
0.01663664
0.01660726
0.01659415
0.01663664
0.01663664
0.01660726
0.01663664
0.01733968
0.01743008
0.01831043
0.01958216
0.01958216
0.01958216
0.01958216
0.01958216
0.01958216
0.01958216
0.01958216
0.01958216
0.01958216
0.02040421
0.02040421
0.02228473
0.02228473
0.02228473
0.02229169
0.02276362

For Review Only

0.02276362
0.02598282
0.02598282
0.02598282
0.02598282
0.02598282
0.02598282
0.02712597
0.02848099
0.02898001
0.02898001
0.02898001
0.02898001
0.02898001
0.02898001
0.02898001
0.02928333
0.02931241
0.03172701
0.03172701
0.03172701
0.03172701
0.03172701
0.03172701
0.03129195
0.03172701
0.03172701
0.03172701
0.03172701
0.03062573
0.03172701
0.03172701
0.03172701
0.03172701
0.03172701
0.03200534
0.03200534
0.03305385
0.03361649
0.03361649
0.03634944
0.03691854
0.03835726
0.03835726
0.03835726
0.03835726

For Review Only

0.03835726
0.03835726
0.03835726
0.03835726
0.03835726
0.03835726
0.03850034
0.03908256
0.03996632
0.0424847
0.0424847
0.0424847
0.0424847
0.0424847
0.0424847
0.0424847
0.04156006
0.0424847
0.0424847
0.0424847
0.0424847
0.0424847
0.043653
0.043653
0.04495584
0.04698991
0.04698991
0.04698991
0.04777194
0.04875583
0.04909655

For Review Only

Supplemental Table S4. Functional annotation (Cellular Component Category) of DEGs identified from different chickpea genotypes under control and stress conditions.

GO ID	Term	P-value
GO:0005576	extracellular region	7.77E-28
GO:0016021	integral component of membrane	1.72E-22
GO:0031224	intrinsic component of membrane	1.06E-19
GO:0016020	membrane	1.14E-17
GO:0048046	apoplast	1.13E-11
GO:0110165	cellular anatomical entity	3.54E-10
GO:0071944	cell periphery	5.51E-10
GO:0009521	photosystem	8.63E-08
GO:0009522	photosystem I	1.04E-06
GO:0031226	intrinsic component of plasma membrane	2.39E-06
GO:0005886	plasma membrane	2.83E-06
GO:0009505	plant-type cell wall	2.95E-06
GO:0009523	photosystem II	5.34E-06
GO:0030312	external encapsulating structure	1.48E-05
GO:0005615	extracellular space	3.47E-05
GO:0005618	cell wall	4.25E-05
GO:0046658	anchored component of plasma membrane	8.94E-05
GO:0030054	cell junction	4.25E-04
GO:0070161	anchoring junction	4.25E-04
GO:0009506	plasmodesma	4.25E-04
GO:0005911	cell-cell junction	4.25E-04
GO:0055044	symplast	4.25E-04
GO:0010287	plastoglobule	0.0019661
GO:0005887	integral component of plasma membrane	0.006222
GO:0034357	photosynthetic membrane	0.0077654
GO:0009538	photosystem I reaction center	0.0111109
GO:0009579	thylakoid	0.0207411
GO:0009535	chloroplast thylakoid membrane	0.0326647
GO:0042651	thylakoid membrane	0.0359278
GO:0055035	plastid thylakoid membrane	0.0430273

For Review Only

Supplemental Table S5. Functional annotation (Molecular Function Category) of DEGs identified from different chickpea genotypes under control and stress conditions.

GO ID	Term	P-value
GO:0016491	oxidoreductase activity	7.88E-35
GO:0046906	tetrapyrrole binding	1.82E-29
GO:0020037	heme binding	4.74E-27
GO:0003700	DNA-binding transcription factor activity	6.03E-24
GO:0140110	transcription regulator activity	7.95E-20
GO:0016705	oxidoreductase activity, acting on paired donors, with incorpo	6.91E-17
GO:0005506	iron ion binding	2.36E-13
GO:0004497	monooxygenase activity	4.02E-12
GO:0004601	peroxidase activity	5.54E-11
GO:0016684	oxidoreductase activity, acting on peroxide as acceptor	7.34E-11
GO:0043565	sequence-specific DNA binding	1.79E-09
GO:0016209	antioxidant activity	1.18E-07
GO:0005215	transporter activity	9.29E-07
GO:0022857	transmembrane transporter activity	1.66E-06
GO:0003824	catalytic activity	1.77E-06
GO:0009055	electron transfer activity	1.94E-06
GO:0016679	oxidoreductase activity, acting on diphenols and related subst	5.65E-06
GO:0015103	inorganic anion transmembrane transporter activity	1.11E-05
GO:0005507	copper ion binding	1.20E-05
GO:0051213	dioxygenase activity	1.35E-05
GO:0016682	oxidoreductase activity, acting on diphenols and related subst	1.57E-05
GO:0033293	monocarboxylic acid binding	1.88E-05
GO:0015318	inorganic molecular entity transmembrane transporter activity	2.25E-05
GO:0008374	O-acyltransferase activity	2.98E-05
GO:0004857	enzyme inhibitor activity	4.27E-05
GO:0016773	phosphotransferase activity, alcohol group as acceptor	6.56E-05
GO:0004672	protein kinase activity	7.02E-05
GO:0015112	nitrate transmembrane transporter activity	9.95E-05
GO:0016420	malonyltransferase activity	9.95E-05
GO:0003993	acid phosphatase activity	1.12E-04
GO:0008194	UDP-glycosyltransferase activity	1.66E-04
GO:0080031	methyl salicylate esterase activity	1.79E-04
GO:0080032	methyl jasmonate esterase activity	1.79E-04
GO:0009815	1-aminocyclopropane-1-carboxylate oxidase activity	1.89E-04
GO:0042562	hormone binding	1.93E-04
GO:0008271	secondary active sulfate transmembrane transporter activity	1.94E-04
GO:0016765	transferase activity, transferring alkyl or aryl (other than meth	2.49E-04
GO:0008447	L-ascorbate oxidase activity	2.52E-04
GO:0045735	nutrient reservoir activity	3.17E-04
GO:0016709	oxidoreductase activity, acting on paired donors, with incorpo	3.85E-04
GO:0047164	isoflavone-7-O-beta-glucoside 6''-O-malonyltransferase activit	4.10E-04

For Review Only

GO:0050736	O-malonyltransferase activity	4.10E-04
GO:0016168	chlorophyll binding	5.21E-04
GO:0019840	isoprenoid binding	5.75E-04
GO:0010427	abscisic acid binding	6.11E-04
GO:0016701	oxidoreductase activity, acting on single donors with incorpora	6.46E-04
GO:0004674	protein serine/threonine kinase activity	6.52E-04
GO:0016757	transferase activity, transferring glycosyl groups	6.74E-04
GO:0043178	alcohol binding	8.12E-04
GO:0004864	protein phosphatase inhibitor activity	8.62E-04
GO:0019212	phosphatase inhibitor activity	8.62E-04
GO:0004611	phosphoenolpyruvate carboxykinase activity	9.55E-04
GO:0016641	oxidoreductase activity, acting on the CH-NH2 group of donors	0.0010272
GO:0016706	2-oxoglutarate-dependent dioxygenase activity	0.0013866
GO:0015075	ion transmembrane transporter activity	0.0014573
GO:0043169	cation binding	0.0015457
GO:0043621	protein self-association	0.0016333
GO:0008964	phosphoenolpyruvate carboxylase activity	0.0016333
GO:0046872	metal ion binding	0.0016393
GO:0008378	galactosyltransferase activity	0.0016496
GO:0016762	xyloglucan:xyloglucosyl transferase activity	0.0017061
GO:0016702	oxidoreductase activity, acting on single donors with incorpora	0.001782
GO:0016638	oxidoreductase activity, acting on the CH-NH2 group of donors	0.0021055
GO:0015116	sulfate transmembrane transporter activity	0.0022401
GO:0004478	methionine adenosyltransferase activity	0.0027306
GO:0070330	aromatase activity	0.0027306
GO:0052634	C-19 gibberellin 2-beta-dioxygenase activity	0.0027306
GO:0052691	UDP-arabinopyranose mutase activity	0.0027306
GO:0030247	polysaccharide binding	0.002796
GO:0015267	channel activity	0.0028159
GO:0022803	passive transmembrane transporter activity	0.0028159
GO:0005315	inorganic phosphate transmembrane transporter activity	0.0029506
GO:0052716	hydroquinone:oxygen oxidoreductase activity	0.0029506
GO:0015301	anion:anion antiporter activity	0.0031384
GO:0140323	solute:anion antiporter activity	0.0031384
GO:0046915	transition metal ion transmembrane transporter activity	0.0032412
GO:0016798	hydrolase activity, acting on glycosyl bonds	0.0033471
GO:0042578	phosphoric ester hydrolase activity	0.0035955
GO:0004867	serine-type endopeptidase inhibitor activity	0.0043138
GO:0046983	protein dimerization activity	0.0044374
GO:0005509	calcium ion binding	0.0050513
GO:0016829	lyase activity	0.0053576
GO:0016301	kinase activity	0.0054997
GO:0019904	protein domain specific binding	0.0056501
GO:0035671	enone reductase activity	0.0056501
GO:0047751	cholestenone 5-alpha-reductase activity	0.0056501
GO:0046423	allene-oxide cyclase activity	0.0056501

For Review Only

GO:0016707	gibberellin 3-beta-dioxygenase activity	0.0056501
GO:0005516	calmodulin binding	0.0057343
GO:0046527	glucosyltransferase activity	0.0059252
GO:0015098	molybdate ion transmembrane transporter activity	0.0062122
GO:0016712	oxidoreductase activity, acting on paired donors, with incorpo	0.0062122
GO:0060089	molecular transducer activity	0.0069552
GO:0004364	glutathione transferase activity	0.0070138
GO:0038023	signaling receptor activity	0.0077207
GO:0008889	glycerophosphodiester phosphodiesterase activity	0.0080692
GO:0015926	glucosidase activity	0.0081503
GO:0019888	protein phosphatase regulator activity	0.0082249
GO:0019208	phosphatase regulator activity	0.0082249
GO:0004553	hydrolase activity, hydrolyzing O-glycosyl compounds	0.009168
GO:0004568	chitinase activity	0.0095792
GO:0005504	fatty acid binding	0.0095792
GO:0030414	peptidase inhibitor activity	0.010536
GO:0004866	endopeptidase inhibitor activity	0.010536
GO:0061135	endopeptidase regulator activity	0.010536
GO:0016161	beta-amylase activity	0.0110269
GO:0102229	amylopectin maltohydrolase activity	0.0110269
GO:0016854	racemase and epimerase activity	0.0108606
GO:0008198	ferrous iron binding	0.0111109
GO:0004766	spermidine synthase activity	0.0111109
GO:0033883	pyridoxal phosphatase activity	0.0111109
GO:0046873	metal ion transmembrane transporter activity	0.0116639
GO:0047172	shikimate O-hydroxycinnamoyltransferase activity	0.0119358
GO:0005344	oxygen carrier activity	0.0119358
GO:0019825	oxygen binding	0.0119358
GO:0045543	gibberellin 2-beta-dioxygenase activity	0.0119358
GO:0005216	ion channel activity	0.0126601
GO:0061134	peptidase regulator activity	0.0132558
GO:0008422	beta-glucosidase activity	0.0141956
GO:0005384	manganese ion transmembrane transporter activity	0.0166073
GO:0016746	transferase activity, transferring acyl groups	0.0168177
GO:0080030	methyl indole-3-acetate esterase activity	0.0167944
GO:0016758	transferase activity, transferring hexosyl groups	0.0170059
GO:0016229	steroid dehydrogenase activity	0.0174301
GO:0047274	galactinol-sucrose galactosyltransferase activity	0.0195822
GO:0102499	SHG alpha-glucan phosphorylase activity	0.0195822
GO:0102250	linear malto-oligosaccharide phosphorylase activity	0.0195822
GO:0004657	proline dehydrogenase activity	0.0195822
GO:0008184	glycogen phosphorylase activity	0.0195822
GO:0008171	O-methyltransferase activity	0.0198601
GO:0004620	phospholipase activity	0.0198826
GO:0005381	iron ion transmembrane transporter activity	0.0204042
GO:0050737	O-hydroxycinnamoyltransferase activity	0.0204042

For Review Only

GO:0050734	hydroxycinnamoyltransferase activity	0.0204042
GO:0004089	carbonate dehydratase activity	0.0204042
GO:0102811	geraniol 10-hydroxylase activity	0.0204042
GO:0008725	DNA-3-methyladenine glycosylase activity	0.0204042
GO:0016791	phosphatase activity	0.0207917
GO:0016655	oxidoreductase activity, acting on NAD(P)H, quinone or similar	0.0221586
GO:0050328	tetrahydroberberine oxidase activity	0.0222847
GO:0015291	secondary active transmembrane transporter activity	0.023009
GO:0015085	calcium ion transmembrane transporter activity	0.0235367
GO:0004180	carboxypeptidase activity	0.0242445
GO:1901682	sulfur compound transmembrane transporter activity	0.0271292
GO:0015293	symporter activity	0.027137
GO:0016857	racemase and epimerase activity, acting on carbohydrates anc	0.0272149
GO:0008137	NADH dehydrogenase (ubiquinone) activity	0.0272149
GO:0004164	diphthine synthase activity	0.031727
GO:0047216	inositol 3-alpha-galactosyltransferase activity	0.031727
GO:0047594	6-beta-hydroxyhyoscyamine epoxidase activity	0.031727
GO:0015137	citrate transmembrane transporter activity	0.031727
GO:0015142	tricarboxylic acid transmembrane transporter activity	0.031727
GO:0102966	arachidoyl-CoA:1-dodecanol O-acyltransferase activity	0.031727
GO:0047689	aspartate racemase activity	0.031727
GO:0061929	gamma-glutamylaminecyclotransferase activity	0.031727
GO:0016298	lipase activity	0.0315479
GO:0047918	GDP-mannose 3,5-epimerase activity	0.031727
GO:0052631	sphingolipid delta-8 desaturase activity	0.031727
GO:0010487	thermospermine synthase activity	0.031727
GO:0046509	1,2-diacylglycerol 3-beta-galactosyltransferase activity	0.031727
GO:0008892	guanine deaminase activity	0.031727
GO:0102003	Delta8-sphingolipid desaturase activity	0.031727
GO:0043733	DNA-3-methylbase glycosylase activity	0.0320053
GO:0003905	alkylbase DNA N-glycosylase activity	0.0320053
GO:0031406	carboxylic acid binding	0.035802
GO:0008081	phosphoric diester hydrolase activity	0.035802
GO:0034485	phosphatidylinositol-3,4,5-trisphosphate 5-phosphatase activi	0.0383573
GO:0045551	cinnamyl-alcohol dehydrogenase activity	0.0383573
GO:0003854	3-beta-hydroxy-delta5-steroid dehydrogenase activity	0.0383573
GO:0030410	nicotianamine synthase activity	0.0424847
GO:0004096	catalase activity	0.0424847
GO:0036361	racemase activity, acting on amino acids and derivatives	0.0424847
GO:0102802	thebaine 6-O-demethylase activity	0.0424847
GO:0102804	oripavine 6-O-demethylase activity	0.0424847
GO:0047661	amino-acid racemase activity	0.0424847
GO:0008199	ferric iron binding	0.0424847
GO:0004792	thiosulfate sulfurtransferase activity	0.0424847
GO:0003933	GTP cyclohydrolase activity	0.0424847
GO:0016853	isomerase activity	0.0442732

For Review Only

GO:0042973	glucan endo-1,3-beta-D-glucosidase activity	0.0449558
GO:0035250	UDP-galactosyltransferase activity	0.0469899
GO:0003677	DNA binding	0.0479342
GO:0050136	NADH dehydrogenase (quinone) activity	0.0487558

For Review Only

Supplemental Table S6. List of differentially expressed transcription factor encoding genes.

XLOC ID	Ca ID	Transcription factor family
XLOC_004915	Ca_v2.0_03915	RAV
XLOC_011668	Ca_v2.0_09339	RAV
XLOC_000110	Ca_v2.0_00199	bHLH
XLOC_000127	Ca_v2.0_00225	bHLH
XLOC_000182	Ca_v2.0_00332	Dof
XLOC_000183	Ca_v2.0_00334	SBP
XLOC_000193	Ca_v2.0_00358	NAC
XLOC_000238	Ca_v2.0_00441	WRKY
XLOC_000273	Ca_v2.0_00502	TCP
XLOC_000397	Ca_v2.0_00753	NF-YB
XLOC_000651	Ca_v2.0_01173	WRKY
XLOC_000670	Ca_v2.0_01198	bHLH
XLOC_000832	Ca_v2.0_01513	GRAS
XLOC_000891	Ca_v2.0_01636	Dof
XLOC_000991	Ca_v2.0_01820	NAC
XLOC_001006	Ca_v2.0_01847	NAC
XLOC_001059	Ca_v2.0_01954	WRKY
XLOC_001089	Ca_v2.0_01999	TCP
XLOC_001131	Ca_v2.0_02078	NAC
XLOC_001185	Ca_v2.0_02175	bHLH
XLOC_001409	Ca_v2.0_02575	Dof
XLOC_001583	Ca_v2.0_02890	bHLH
XLOC_001589	Ca_v2.0_02905	WRKY
XLOC_001667	Ca_v2.0_00113	NAC
XLOC_001781	Ca_v2.0_00333	Dof
XLOC_001817	Ca_v2.0_00404	bHLH
XLOC_001818	Ca_v2.0_00409	Dof
XLOC_002088	Ca_v2.0_00921	WRKY
XLOC_002189	Ca_v2.0_01155	bHLH
XLOC_002625	Ca_v2.0_01951	BES1
XLOC_002709	Ca_v2.0_02114	bHLH
XLOC_002713	Ca_v2.0_02119	GRAS
XLOC_002770	Ca_v2.0_02230	bZIP
XLOC_002826	Ca_v2.0_02330	bHLH
XLOC_002870	Ca_v2.0_02440	TCP
XLOC_003383	Ca_v2.0_03230	bZIP
XLOC_003723	Ca_v2.0_03858	bZIP
XLOC_003754	Ca_v2.0_03920	GRAS
XLOC_003988	Ca_v2.0_04335,Ca_v2.0_(	WRKY
XLOC_004273	Ca_v2.0_04904	NAC
XLOC_004519	Ca_v2.0_03133	ZF-HD
XLOC_004597	Ca_v2.0_03308	LBD

For Review Only

XLOC_004646	Ca_v2.0_03414	Dof
XLOC_004771	Ca_v2.0_03660	bHLH
XLOC_004798	Ca_v2.0_03704	NAC
XLOC_004825	Ca_v2.0_03751	WRKY
XLOC_005138	Ca_v2.0_04349	WRKY
XLOC_005148	Ca_v2.0_04368	LBD
XLOC_005534	Ca_v2.0_05043	GRAS
XLOC_005580	Ca_v2.0_05140,Ca_v2.0_(	WRKY
XLOC_005777	Ca_v2.0_05345	Dof
XLOC_006180	Ca_v2.0_06160	bHLH
XLOC_006348	Ca_v2.0_06485	Trihelix
XLOC_006363	Ca_v2.0_06513	GRAS
XLOC_006443	Ca_v2.0_06648	GRAS
XLOC_006512	Ca_v2.0_06787	WRKY
XLOC_006552	Ca_v2.0_06864	bHLH
XLOC_006636	Ca_v2.0_07039	NAC
XLOC_006638	Ca_v2.0_07041	NAC
XLOC_006749	Ca_v2.0_07252	bHLH
XLOC_006773	Ca_v2.0_07291	HSF
XLOC_006795	Ca_v2.0_07340	bHLH
XLOC_006927	Ca_v2.0_07590	bHLH
XLOC_007038	Ca_v2.0_07799	bZIP
XLOC_007138	Ca_v2.0_08015	GRAS
XLOC_007139	Ca_v2.0_08017	WRKY
XLOC_007145	Ca_v2.0_08025	WRKY
XLOC_007165	Ca_v2.0_08058	bHLH
XLOC_007230	Ca_v2.0_08194	bHLH
XLOC_007255	Ca_v2.0_08251	bZIP
XLOC_007292	Ca_v2.0_08313	bZIP
XLOC_007312	Ca_v2.0_08347	Dof
XLOC_007508	Ca_v2.0_05501	bZIP
XLOC_007822	Ca_v2.0_06054	bHLH
XLOC_008098	Ca_v2.0_06550,Ca_v2.0_(	WRKY
XLOC_008127	Ca_v2.0_06608	WRKY
XLOC_008537	Ca_v2.0_07349	bZIP
XLOC_008608	Ca_v2.0_07505	LBD
XLOC_009070	Ca_v2.0_08341	bHLH
XLOC_009279	Ca_v2.0_08505	NAC
XLOC_009291	Ca_v2.0_08528	LBD
XLOC_009762	Ca_v2.0_09393	GATA
XLOC_009821	Ca_v2.0_09524	Trihelix
XLOC_010287	Ca_v2.0_10379	HSF
XLOC_010384	Ca_v2.0_10584	bZIP
XLOC_010497	Ca_v2.0_10837	bHLH
XLOC_010628	Ca_v2.0_11069	bHLH
XLOC_010772	Ca_v2.0_11341	GRAS

For Review Only

XLOC_010858	Ca_v2.0_11497	WRKY
XLOC_011039	Ca_v2.0_11827	bHLH
XLOC_011118	Ca_v2.0_11969	BBR-BPC
XLOC_011250	Ca_v2.0_08568	HSF
XLOC_011458	Ca_v2.0_08931	bHLH
XLOC_011547	Ca_v2.0_09121	bZIP
XLOC_011580	Ca_v2.0_09189	NF-YB
XLOC_011706	Ca_v2.0_09422	LBD
XLOC_011841	Ca_v2.0_09659	bHLH
XLOC_011937	Ca_v2.0_09832	FAR1
XLOC_012917	Ca_v2.0_11643	WRKY
XLOC_013066	Ca_v2.0_11957	SRS
XLOC_013404	Ca_v2.0_12309	Trihelix
XLOC_013488	Ca_v2.0_12464	bZIP
XLOC_013633	Ca_v2.0_12712	Dof
XLOC_013974	Ca_v2.0_13353	LBD
XLOC_014055	Ca_v2.0_13527	LBD
XLOC_014410	Ca_v2.0_14173,Ca_v2.0_	bZIP
XLOC_014417	Ca_v2.0_14185	NAC
XLOC_014467	Ca_v2.0_14260	GRAS
XLOC_014486	Ca_v2.0_14293	bHLH
XLOC_014512	Ca_v2.0_14345	Dof
XLOC_014585	Ca_v2.0_14482	WRKY
XLOC_014636	Ca_v2.0_14570	NAC
XLOC_014664	Ca_v2.0_14641	bHLH
XLOC_014793	Ca_v2.0_14872	HSF
XLOC_014906	Ca_v2.0_15095	bZIP
XLOC_015036	Ca_v2.0_15355	Nin-like
XLOC_015816	Ca_v2.0_13374	WRKY
XLOC_015902	Ca_v2.0_13517	LBD
XLOC_015943	Ca_v2.0_13576	GRAS
XLOC_016299	Ca_v2.0_14294	WRKY
XLOC_016394	Ca_v2.0_14479	WRKY
XLOC_016489	Ca_v2.0_14652	NAC
XLOC_016508	Ca_v2.0_14694	bHLH
XLOC_016553	Ca_v2.0_14784	HSF
XLOC_016612	Ca_v2.0_14885	WRKY
XLOC_016644	Ca_v2.0_14943	WRKY
XLOC_016710	Ca_v2.0_15072	WRKY
XLOC_016774	Ca_v2.0_15189	bHLH
XLOC_017159	Ca_v2.0_15564	GRAS
XLOC_017378	Ca_v2.0_15967	WRKY
XLOC_017449	Ca_v2.0_16087	NAC
XLOC_017503	Ca_v2.0_16191	NAC
XLOC_017743	Ca_v2.0_16663	TCP
XLOC_017783	Ca_v2.0_16739	bHLH

For Review Only

XLOC_017851	Ca_v2.0_16892	CAMTA
XLOC_018104	Ca_v2.0_17370	WRKY
XLOC_018414	Ca_v2.0_17947	WRKY
XLOC_018914	Ca_v2.0_18872	bZIP
XLOC_019057	Ca_v2.0_19108	GRAS
XLOC_019089	Ca_v2.0_19166	bHLH
XLOC_019175	Ca_v2.0_19347	bZIP
XLOC_019280	Ca_v2.0_15651,Ca_v2.0_'	NF-X1
XLOC_019458	Ca_v2.0_16001	WRKY
XLOC_019545	Ca_v2.0_16173	NAC
XLOC_019573	Ca_v2.0_16220	GRAS
XLOC_019876	Ca_v2.0_16806	WRKY
XLOC_020019	Ca_v2.0_17058	Dof
XLOC_020077	Ca_v2.0_17171	bHLH
XLOC_020186	Ca_v2.0_17394	Dof
XLOC_020301	Ca_v2.0_17612	GRAS
XLOC_020304	Ca_v2.0_17618	NAC
XLOC_020393	Ca_v2.0_17793	WRKY
XLOC_020410	Ca_v2.0_17829	bHLH
XLOC_020515	Ca_v2.0_18015	Trihelix
XLOC_020922	Ca_v2.0_18791,Ca_v2.0_'	WRKY
XLOC_021037	Ca_v2.0_19028	LBD
XLOC_021039	Ca_v2.0_19032	Dof
XLOC_021077	Ca_v2.0_19112	GRAS
XLOC_021135	Ca_v2.0_19201	FAR1
XLOC_021447	Ca_v2.0_19474	GRAS
XLOC_021467	Ca_v2.0_19511	WRKY
XLOC_021917	Ca_v2.0_20356	TCP
XLOC_022146	Ca_v2.0_20746	bHLH
XLOC_022148	Ca_v2.0_20748	bHLH
XLOC_022167	Ca_v2.0_20786	GRAS
XLOC_022268	Ca_v2.0_20947	ZF-HD
XLOC_022815	Ca_v2.0_22005	bHLH
XLOC_022827	Ca_v2.0_22023	LBD
XLOC_022923	Ca_v2.0_22192	WRKY
XLOC_023038	Ca_v2.0_22393	Trihelix
XLOC_023111	Ca_v2.0_22530	Dof
XLOC_023120	Ca_v2.0_22547	bHLH
XLOC_023137	Ca_v2.0_22579	GATA
XLOC_023328	Ca_v2.0_19457	NF-YB
XLOC_023801	Ca_v2.0_20340	Dof
XLOC_023820	Ca_v2.0_20370	WRKY
XLOC_023903	Ca_v2.0_20543	Trihelix
XLOC_024066	Ca_v2.0_20879	Dof
XLOC_024296	Ca_v2.0_21308	bHLH
XLOC_024421	Ca_v2.0_21566	NAC

For Review Only

XLOC_024465	Ca_v2.0_21621	TCP
XLOC_024567	Ca_v2.0_21826	Dof
XLOC_024671	Ca_v2.0_22022	LBD
XLOC_024686	Ca_v2.0_22068	TCP
XLOC_024773	Ca_v2.0_22260	WRKY
XLOC_024787	Ca_v2.0_22280	bHLH
XLOC_024821	Ca_v2.0_22342	NAC
XLOC_024976	Ca_v2.0_22641	bHLH
XLOC_025038	Ca_v2.0_22769	WRKY
XLOC_025412	Ca_v2.0_23230	bHLH
XLOC_025414	Ca_v2.0_23232	bHLH
XLOC_025469	Ca_v2.0_23343	HSF
XLOC_025480	Ca_v2.0_23360	LBD
XLOC_025508	Ca_v2.0_23410	BES1
XLOC_026411	Ca_v2.0_23248	bZIP
XLOC_026720	Ca_v2.0_23815	bHLH
XLOC_027102	Ca_v2.0_24521	bHLH
XLOC_006274	Ca_v2.0_06339	ARF
XLOC_009763	Ca_v2.0_09394	ARF
XLOC_019005	Ca_v2.0_19018	ARF
XLOC_022695	Ca_v2.0_21760	ARF
XLOC_011066	.	B3
XLOC_012661	Ca_v2.0_11144	B3
XLOC_014737	Ca_v2.0_14769,Ca_v2.0_	B3
XLOC_018616	Ca_v2.0_18346	B3
XLOC_019114	Ca_v2.0_19227	B3
XLOC_014780	Ca_v2.0_14844	AP2
XLOC_021043	Ca_v2.0_19040	AP2
XLOC_022192	Ca_v2.0_20829	AP2
XLOC_024010	Ca_v2.0_20761	AP2
XLOC_025456	Ca_v2.0_23321	AP2
XLOC_026738	Ca_v2.0_23850	AP2
XLOC_000203	Ca_v2.0_00376	ERF
XLOC_000611	Ca_v2.0_01103	ERF
XLOC_000719	Ca_v2.0_01277	ERF
XLOC_001314	Ca_v2.0_02386	ERF
XLOC_002244	Ca_v2.0_01276	ERF
XLOC_002247	Ca_v2.0_01278	ERF
XLOC_003510	Ca_v2.0_03448	ERF
XLOC_003527	Ca_v2.0_03480	ERF
XLOC_004920	Ca_v2.0_03926	ERF
XLOC_004973	Ca_v2.0_04028	ERF
XLOC_005335	Ca_v2.0_04674	ERF
XLOC_005873	Ca_v2.0_05544	ERF
XLOC_006573	Ca_v2.0_06909	ERF
XLOC_006882	Ca_v2.0_07510	ERF

For Review Only

XLOC_007310	Ca_v2.0_08342	ERF
XLOC_007835	Ca_v2.0_06075	ERF
XLOC_008378	Ca_v2.0_07065	ERF
XLOC_008604	Ca_v2.0_07496	ERF
XLOC_009285	Ca_v2.0_08517	ERF
XLOC_009363	Ca_v2.0_08664	ERF
XLOC_009365	Ca_v2.0_08666	ERF
XLOC_009888	Ca_v2.0_09639	ERF
XLOC_010499	Ca_v2.0_10841	ERF
XLOC_010581	Ca_v2.0_10985	ERF
XLOC_010812	Ca_v2.0_11413	ERF
XLOC_010921	Ca_v2.0_11614	ERF
XLOC_011224	Ca_v2.0_08516	ERF
XLOC_011225	Ca_v2.0_08518	ERF
XLOC_011560	Ca_v2.0_09152,Ca_v2.0_(	ERF
XLOC_011613	Ca_v2.0_09242	ERF
XLOC_011759	Ca_v2.0_09512	ERF
XLOC_012982	Ca_v2.0_11773	ERF
XLOC_013829	Ca_v2.0_13077	ERF
XLOC_014075	Ca_v2.0_13571	ERF
XLOC_014484	Ca_v2.0_14291	ERF
XLOC_016472	Ca_v2.0_14624	ERF
XLOC_016559	Ca_v2.0_14789	ERF
XLOC_016629	Ca_v2.0_14916	ERF
XLOC_016653	Ca_v2.0_14957	ERF
XLOC_017527	Ca_v2.0_16238	ERF
XLOC_017561	Ca_v2.0_16298	ERF
XLOC_020106	Ca_v2.0_17233	ERF
XLOC_020892	Ca_v2.0_18723	ERF
XLOC_022050	Ca_v2.0_20575	ERF
XLOC_022051	Ca_v2.0_20576	ERF
XLOC_022102	Ca_v2.0_20675	ERF
XLOC_022869	Ca_v2.0_22086	ERF
XLOC_023266	Ca_v2.0_22788	ERF
XLOC_023751	Ca_v2.0_20244	ERF
XLOC_025049	Ca_v2.0_22787	ERF
XLOC_025295	Ca_v2.0_22999	ERF
XLOC_025307	Ca_v2.0_23024	ERF
XLOC_025340	Ca_v2.0_23084	ERF
XLOC_025375	Ca_v2.0_23166	ERF
XLOC_026048	Ca_v2.0_24420	ERF
XLOC_026158	Ca_v2.0_24626	ERF
XLOC_001052	Ca_v2.0_01938	DBB
XLOC_008325	Ca_v2.0_06964	DBB
XLOC_015080	Ca_v2.0_15440	DBB
XLOC_026537	Ca_v2.0_23493	DBB

For Review Only

XLOC_003929	Ca_v2.0_04240	CO-like
XLOC_023420	Ca_v2.0_19632	CO-like
XLOC_022134	Ca_v2.0_20724	ARR-B
XLOC_004668	Ca_v2.0_03466	G2-like
XLOC_004789	Ca_v2.0_03692	G2-like
XLOC_008084	Ca_v2.0_06509	G2-like
XLOC_010374	Ca_v2.0_10569	G2-like
XLOC_011068	Ca_v2.0_11874	G2-like
XLOC_011667	Ca_v2.0_09337	G2-like
XLOC_013560	Ca_v2.0_12586	G2-like
XLOC_018115	Ca_v2.0_17395	G2-like
XLOC_019066	Ca_v2.0_19124	G2-like
XLOC_019715	Ca_v2.0_16495	G2-like
XLOC_003880	Ca_v2.0_04154	MIKC_MADS
XLOC_014712	Ca_v2.0_14726	MIKC_MADS
XLOC_024683	Ca_v2.0_22063	MIKC_MADS
XLOC_025553	Ca_v2.0_23484	MIKC_MADS
XLOC_026726	Ca_v2.0_23825	MIKC_MADS
XLOC_007031	Ca_v2.0_07782	GRF
XLOC_026657	Ca_v2.0_23715	GRF
XLOC_015041	Ca_v2.0_15367	WOX
XLOC_010335	Ca_v2.0_10482	TALE
XLOC_026485	Ca_v2.0_23393	TALE
XLOC_004768	Ca_v2.0_03657	HD-ZIP
XLOC_004512	Ca_v2.0_03119	HD-ZIP
XLOC_012342	Ca_v2.0_10553	HD-ZIP
XLOC_025373	Ca_v2.0_23162	HD-ZIP
XLOC_014383	Ca_v2.0_14126	HD-ZIP
XLOC_016336	Ca_v2.0_14366	HD-ZIP
XLOC_020131	Ca_v2.0_17283	HD-ZIP
XLOC_025510	Ca_v2.0_23414	HD-ZIP
XLOC_014786	Ca_v2.0_14850	HD-ZIP
XLOC_021586	Ca_v2.0_19714	HD-ZIP
XLOC_006818	Ca_v2.0_07384	HD-ZIP
XLOC_010991	Ca_v2.0_11746	TALE
XLOC_026766	Ca_v2.0_23903	TALE
XLOC_001511	Ca_v2.0_02775	HD-ZIP
XLOC_014932	Ca_v2.0_15145	HD-ZIP
XLOC_001186	Ca_v2.0_02177	C2H2
XLOC_001187	Ca_v2.0_02178	C2H2
XLOC_001436	Ca_v2.0_02621	C2H2
XLOC_001784	Ca_v2.0_00338	C3H
XLOC_002861	Ca_v2.0_02428	C2H2
XLOC_003111	Ca_v2.0_02898	C2H2
XLOC_004141	Ca_v2.0_04647,Ca_v2.0_(	C3H
XLOC_004721	Ca_v2.0_03571	C2H2

For Review Only

XLOC_006789	Ca_v2.0_07327	C2H2
XLOC_008670	Ca_v2.0_07616	C2H2
XLOC_008671	Ca_v2.0_07617	C2H2
XLOC_009994	Ca_v2.0_09825	C2H2
XLOC_010966	Ca_v2.0_11698	C2H2
XLOC_011017	Ca_v2.0_11793	C2H2
XLOC_011128	Ca_v2.0_11985	C2H2
XLOC_011982	Ca_v2.0_09918	C3H
XLOC_012035	Ca_v2.0_10029	C2H2
XLOC_012699	Ca_v2.0_11213	C2H2
XLOC_013931	Ca_v2.0_13279	C2H2
XLOC_017772	Ca_v2.0_16710	C2H2
XLOC_018174	Ca_v2.0_17502	C2H2
XLOC_018916	Ca_v2.0_18876	C2H2
XLOC_019249	Ca_v2.0_15593	C2H2
XLOC_020990	Ca_v2.0_18934	C2H2
XLOC_023096	Ca_v2.0_22500	C2H2
XLOC_000489	Ca_v2.0_00908	MYB
XLOC_001144	Ca_v2.0_02102	MYB
XLOC_001734	Ca_v2.0_00246	MYB
XLOC_003332	Ca_v2.0_03145	MYB
XLOC_005765	Ca_v2.0_05314	MYB
XLOC_006888	Ca_v2.0_07521	MYB
XLOC_007272	Ca_v2.0_08280	MYB
XLOC_007761	Ca_v2.0_05951	MYB
XLOC_008408	Ca_v2.0_07126	MYB
XLOC_008716	Ca_v2.0_07706	MYB
XLOC_008924	Ca_v2.0_08078	MYB
XLOC_009568	Ca_v2.0_09047	MYB
XLOC_009598	Ca_v2.0_09099	MYB
XLOC_009599	Ca_v2.0_09100	MYB
XLOC_010095	Ca_v2.0_10007	MYB
XLOC_010992	Ca_v2.0_11748	MYB
XLOC_011288	Ca_v2.0_08633	MYB
XLOC_012570	Ca_v2.0_10962	MYB
XLOC_013926	Ca_v2.0_13270	MYB
XLOC_014119	Ca_v2.0_13646	MYB
XLOC_016275	Ca_v2.0_14245	MYB
XLOC_018439	Ca_v2.0_17993	MYB
XLOC_018496	Ca_v2.0_18100	MYB
XLOC_020881	Ca_v2.0_18709	MYB
XLOC_020882	Ca_v2.0_18710	MYB
XLOC_021666	Ca_v2.0_19873	MYB
XLOC_023965	Ca_v2.0_20661	MYB
XLOC_024792	Ca_v2.0_22292	MYB
XLOC_025336	Ca_v2.0_23076	MYB

For Review Only

XLOC_025719	Ca_v2.0_23782	MYB
XLOC_026041	Ca_v2.0_24413	MYB
XLOC_026898	Ca_v2.0_24150	MYB
XLOC_002514	Ca_v2.0_01744	MYB_related
XLOC_002944	Ca_v2.0_02558	MYB_related
XLOC_003591	Ca_v2.0_03600	MYB_related
XLOC_005200	Ca_v2.0_04442	MYB_related
XLOC_007333	Ca_v2.0_08387,Ca_v2.0_(	MYB_related
XLOC_010584	Ca_v2.0_10990	MYB_related
XLOC_012386	Ca_v2.0_10626,Ca_v2.0_'	MYB_related
XLOC_014707	Ca_v2.0_14717	MYB_related
XLOC_016800	Ca_v2.0_15232	MYB_related
XLOC_017152	.	MYB_related
XLOC_018849	Ca_v2.0_18748	MYB_related
XLOC_021063	Ca_v2.0_19075	MYB_related
XLOC_026189	Ca_v2.0_24685	MYB_related
XLOC_027034	Ca_v2.0_24401	MYB_related
XLOC_002489	Ca_v2.0_01694	C2H2

For Review Only
